# α-Aminophosphonate inhibitors of metallo-β-lactamases NDM-1 and VIM-2†

**DOI:** 10.1039/d3md00286a

**Published:** 2023-08-02

**Authors:** Katarzyna Palica, Fritz Deufel, Susann Skagseth, Gabriela Paula Di Santo Metzler, Johannes Thoma, Anna Andersson Rasmussen, Arto Valkonen, Per Sunnerhagen, Hanna-Kirsti S. Leiros, Hanna Andersson, Mate Erdelyi

**Affiliations:** a Department of Chemistry – BMC, Organic Chemistry, Uppsala University Husargatan 3 752 37 Uppsala Sweden hanna.andersson@redglead.com mate.erdelyi@kemi.uu.se; b Department of Chemistry, Faculty of Science and Technology, UiT The Arctic University of Norway N-9037 Tromsø Norway; c Department of Chemistry & Molecular Biology, University of Gothenburg Medicinaregatan 9C 413 90 Göteborg Sweden; d Center for Antibiotics Resistance Research (CARe) at University of Gothenburg 413 90 Göteborg Sweden; e Department of Chemistry, University of Jyvaskyla Survontie 9B 40014 Finland

## Abstract

The upswing of antibiotic resistance is an escalating threat to human health. Resistance mediated by bacterial metallo-β-lactamases is of particular concern as these enzymes degrade β-lactams, our most frequently prescribed class of antibiotics. Inhibition of metallo-β-lactamases could allow the continued use of existing β-lactam antibiotics, such as penicillins, cephalosporins and carbapenems, whose applicability is becoming ever more limited. The design, synthesis, and NDM-1, VIM-2, and GIM-1 inhibitory activities (IC_50_ 4.1–506 μM) of a series of novel non-cytotoxic α-aminophosphonate-based inhibitor candidates are presented herein. We disclose the solution NMR spectroscopic and computational investigation of their NDM-1 and VIM-2 binding sites and binding modes. Whereas the binding modes of the inhibitors are similar, VIM-2 showed a somewhat higher conformational flexibility, and complexed a larger number of inhibitor candidates in more varying binding modes than NDM-1. Phosphonate-type inhibitors may be potential candidates for development into therapeutics to combat metallo-β-lactamase resistant bacteria.

## Abstract 2

α-Aminophosphonate inhibitors of the bacterial metallo-β-lactamases NDM-1 and VIM-2 have been developed and studied.

## Graphical abstract

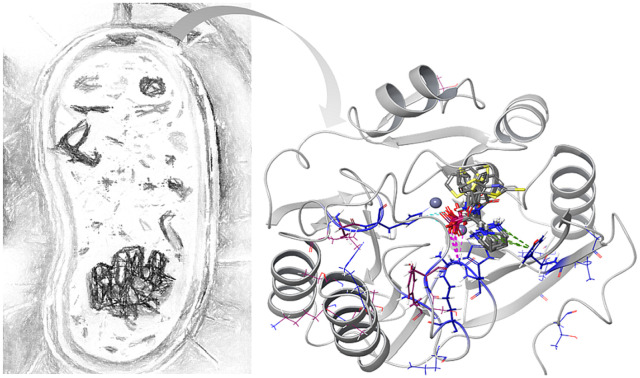

## Introduction

**Fig. 1:**
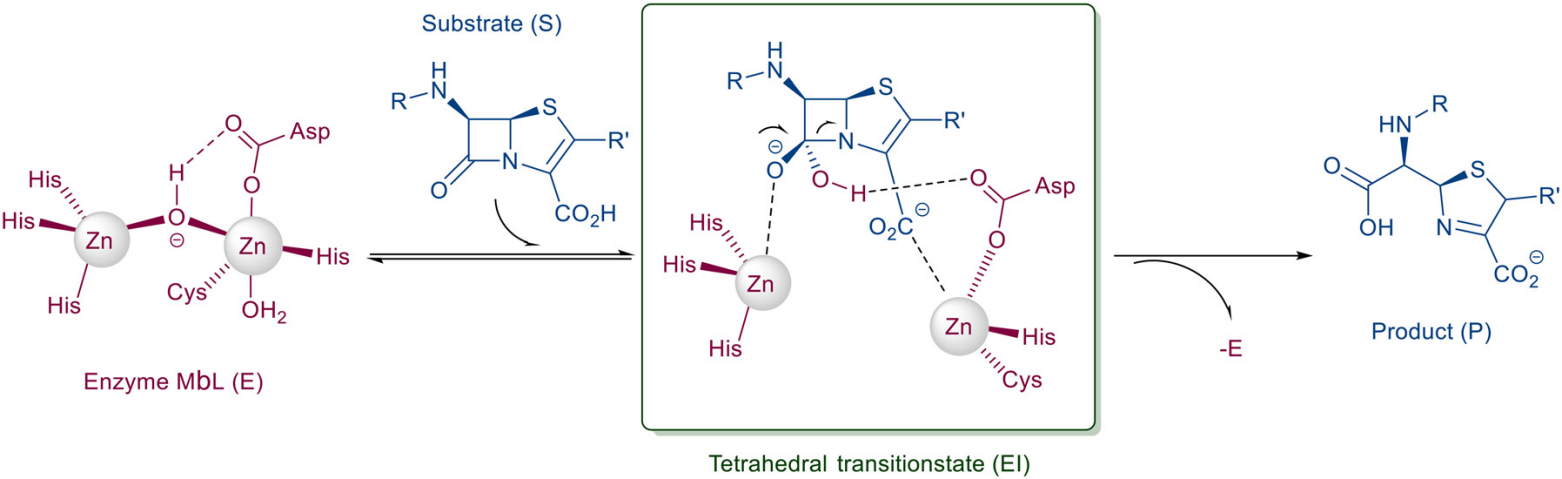
The schematic mechanism of β-lactam hydrolysis facilitated by metallo-β-lactamases.^5^

Antimicrobial resistance is a major threat to human health that already causes over a million deaths annually.^1^ It is expected to become the leading cause of mortality by 2050.^2^ Out of the multiple resistance mechanisms that bacteria possess,^3^ metallo-β-lactamase mediated degradation pathways are currently the most worrisome^4^ as they render β-lactam antibiotics inactive (Fig. 1),^5^ including also carbapenems, a class of drugs of last-resort.^6,7^ Historically, β-lactams constitute the most widely used, cheapest to produce and most prescribed class of antibiotics.^4,8^ The New Delhi metallo-β-lactamase 1 (NDM-1)^9^ and the Verona integron-encoded metallo-β-lactamase 2 (VIM-2)^10^ are of particular concern as they are commonly found in clinical isolates and are spread worldwide.^11^ The transmutation of the plasmid-borne β-lactamase genes (*bla*) between Gram-negative bacteria^12^ made *bla*_NDM-1_ the predominant β-lactamase in Europe and endemic in the Indian subcontinent.^4^

The mutation rate of metallo-β-lactamases outnumbers that of antibiotic discovery by far, leading to a rapid increase in the number of bacteria insensitive to β-lactam antibiotics. Out of the 7279 β-lactamases reported so far, 38 are NDM-1 and 74 are VIM-2 variants,^13^ with further β-lactamases being continuously identified.^14^

Besides the design of novel β-lactams reluctant to hydrolysis by metallo-β-lactamases, the development of metallo-β-lactamase inhibitors is the most promising strategy to counteract metallo-β-lactamase-based antibiotic resistance.^15^ These inhibitors, when combined with antibiotics and formulated into a single product, may prolong the usefulness of existing β-lactam antibiotics. This strategy was demonstrated to be successful by the introduction of serine-β-lactamase inhibitors, such as tazobactam^16^ and clavulanic acid.^17^

Metallo-β-lactamases do not share an evolutionary relationship with serine-β-lactamases. They possess different structures, active sites and catalytic mechanisms. Consequently, serine-β-lactamase inhibitors are inactive against metallo-β-lactamases, except for the recently developed taniborbactam.^18^ Captopril derivatives, thiorphan, tiopronin, 2,3-dimercaprol, ML302F, L-CS319, 2-(4-fluorobenzoyl)benzoic acid, 3-formylchromone, cyclic boronates and ANT-2681 are examples of compounds that act by ligand replacement, whereas SIT-Z5 and aspergillomarasmine A^19^ act by metal sequestration. These are both Zn-dependent strategies.^20,21^ Schofield's isoquinolines (IC_50_ 10–1000 μM)^22^ are the most successful Zn-independent inhibitors. Most inhibitors discovered so far have IC_50_s in the μM range, although a few compounds with nM potency have also been reported.^23^ The field has been extensively reviewed.^24–27^ Importantly, no clinically applicable metallo-β-lactamase inhibitors are available to date, and merely two candidates have yet been reported to have reached clinical trials.^8^ Consequently, the development of new classes of metallo-β-lactamase inhibitors that provide protection to some of our clinically most precious antibiotics is expected to be one of the possibly most valuable contributions to combating antibiotic resistance.^28^

## Results and discussion

### Design

**Fig. 2:**
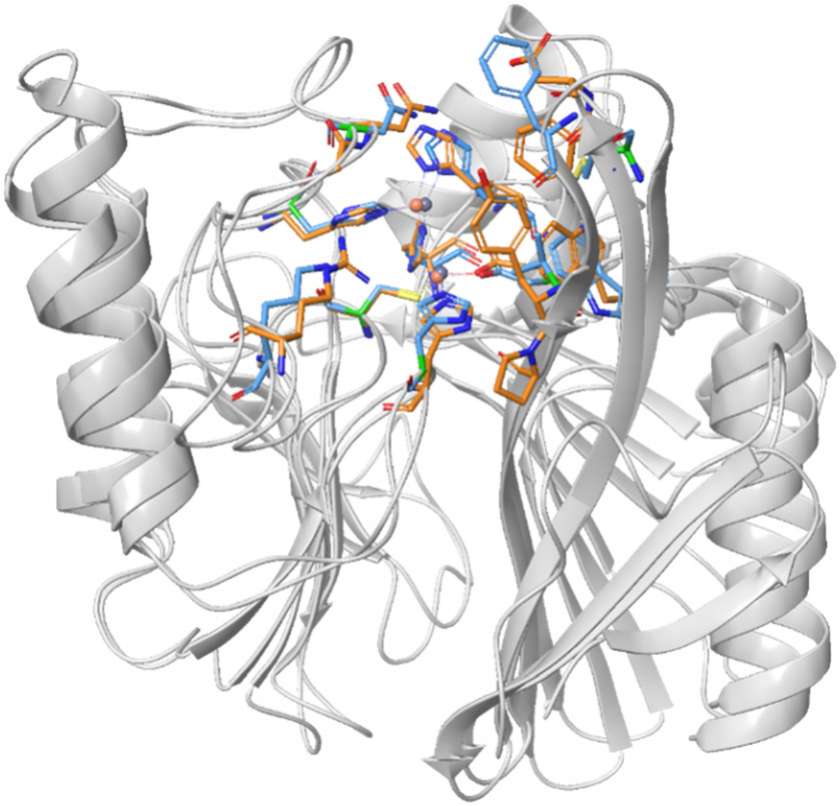
Superimposed structures of NDM-1 (blue, pdb: 6NY7) and VIM-2 (orange, pdb: 6O5T).^29^ The amino acid residues that are most important for binding of β-lactam antibiotics are highlighted to indicate the conserved part of the active site.

Mimetics of the tetrahedral transition state of β-lactam hydrolysis^29^ (Fig. 1) are expected to yield potential metallo-β-lactamase inhibitors. The active site of NDM-1 and of VIM-2 are structurally similar (Fig. 2, and section S3 of ESI†), and involve the same amino acid residues to facilitate zinc coordination. In addition, both enzymes use flexible loops to allow for adjustment of the active site to enable the binding of a wide range of β-lactam antibiotics.

**Scheme 1:**
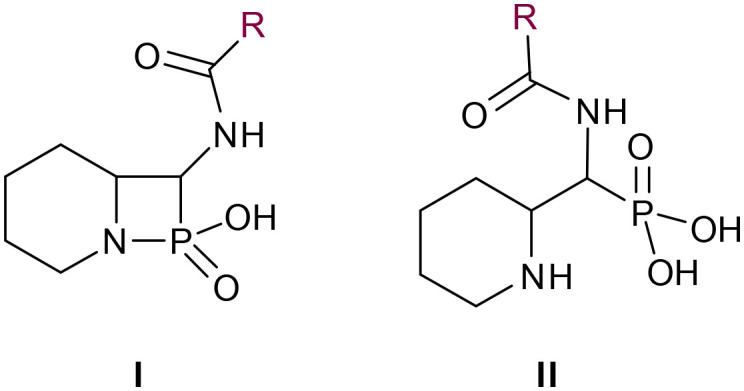
Piperidine substituted phospholactams and α-aminophosphonates are expected to bind metallo-β-lactamases as the geometry of their P=O motif resembles the tetrahedral intermediate of β-lactam hydrolysis.

In our design of novel inhibitors, we introduced a phosphonic acid and a piperidine ring as key binding motives (Scheme 1). Phosphonic acid derivatives^30^ and phosphonamidates^31^ have previously been studied in the context of metallo-β-lactamase inhibition. The phosphonate group was incorporated to mimic the amide carbonyl of β-lactams and is expected to coordinate the zinc ions of metallo-β-lactamases.^32,33^ The piperidine possibly interacts with the catalytic site of metallo-β-lactamases. Initially, phospholactams (**I**) showed low stability in aqueous solutions in preliminary experiments,^34^ so we turned to their chemically stable analogues **II**, as their tetrahedral phosphonate motif resembles the tetrahedral transition state of hydrolysed β-lactams (Scheme 1). The phosphonic acid core is thus expected to interact within the active site, by strongly coordinating the zinc ions with its oxygens, analogous to that reported for structurally related phosphonate-based metallo-β-lactamase inhibitors (see for example pdb: 5HH6).^32,35^

**Scheme 2:**
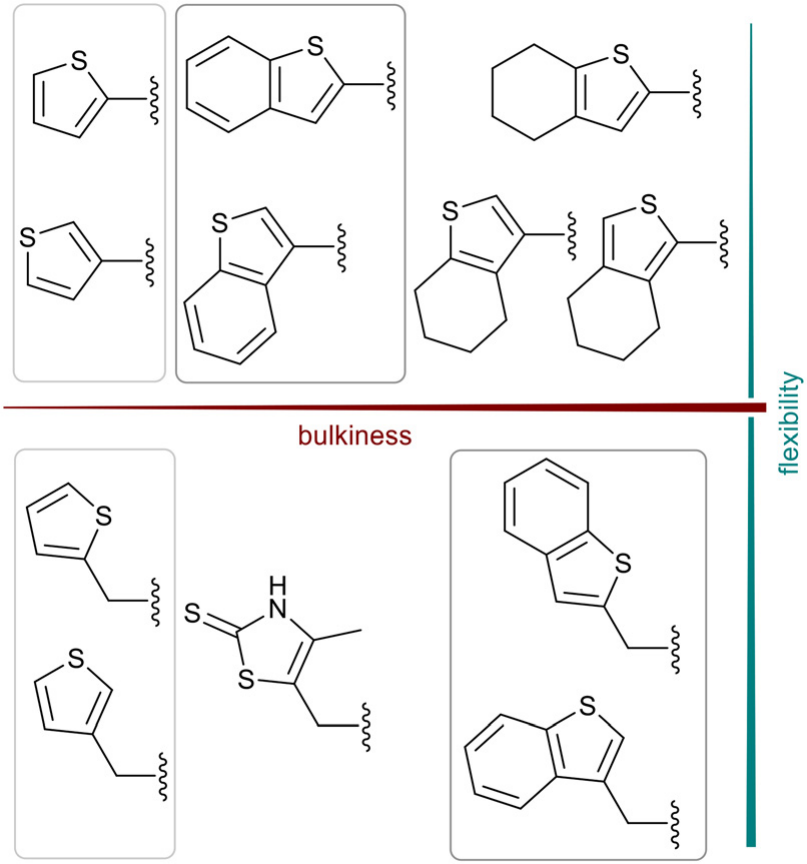
Thiophenes with varying regioisomer patterns, steric bulk, polarity and flexibility have been used as the “*R*”-substituents (Scheme 1) of the α-aminophosphonate-type metallo-β-lactamase inhibitors developed herein.

We further introduced thiophene moieties with variation in their regioisomeric pattern, steric bulk and flexibility (Scheme 2) as sulfur substitution is common in several classes of β-lactams (penams, penems, cephems, *etc.*). The (thiophene-2-yl)acet-amido and thiophene moieties, which are bioisosteres of benzyl and phenyl groups, respectively,^36^ were anticipated to interact with the hydrophobic and aromatic amino acid residues of loop 3 (Phe70/Tyr65). These sidechains vary in hydrophobicity, which we deemed worth exploring. In SAR studies of β-lactamase inhibitors, the properties of aromatic moieties were found important as they contribute to crucial π–π interactions.^37^ The structural variation of the aromatic moiety in the present series is also expected to affect the solubility of the compounds.

### Synthesis

**Scheme 3:**
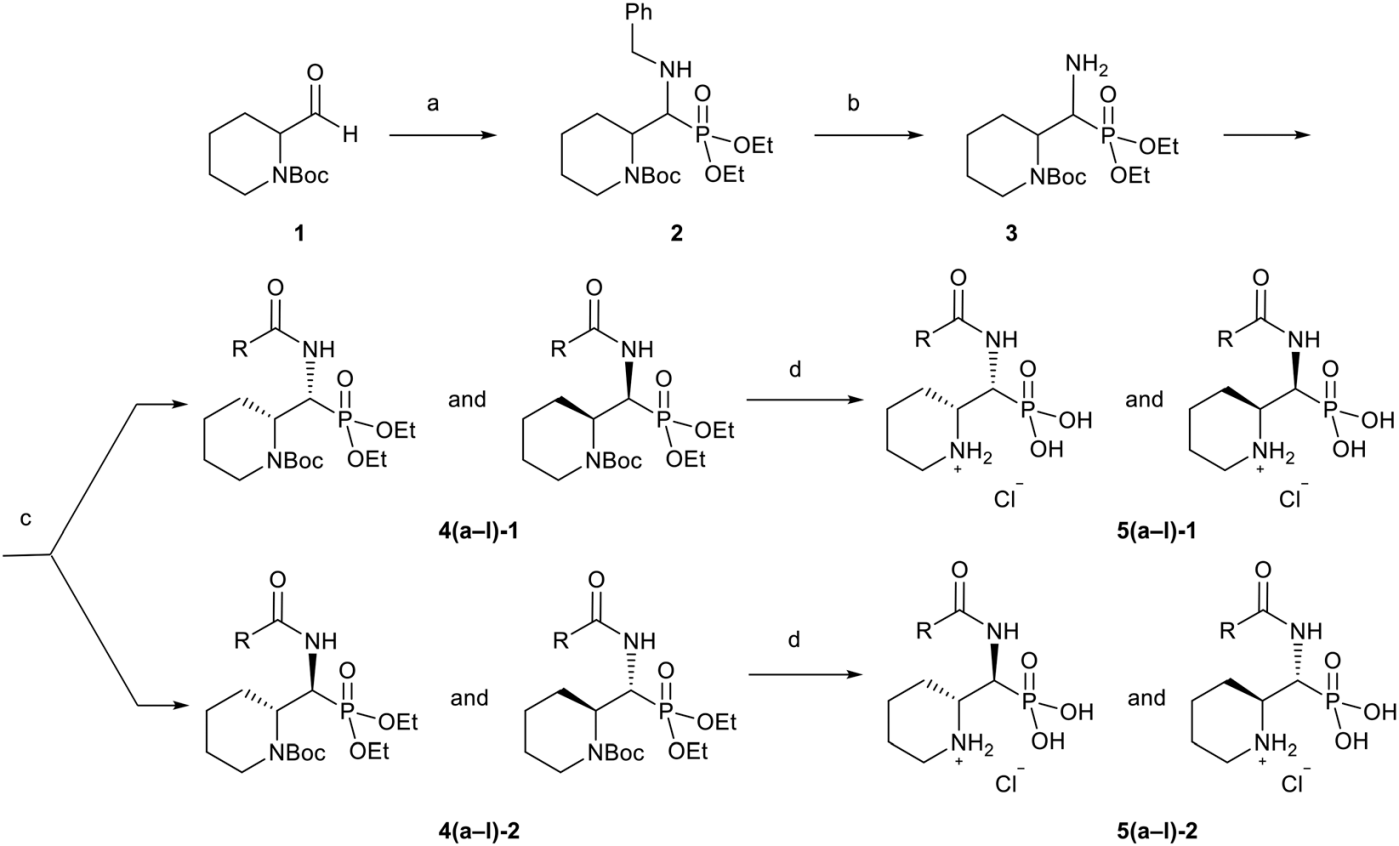
Synthesis of the target compounds. Reagents and conditions: (a) BnNH_2_, HP(O)(OEt)_2_, HfCl_4_ (cat), EtOH, 60 °C, 12 h, 32%; (b) H_2_ (1 atm), Pd(OH_2_)/C (cat), EtOH, r.t., 16 h, 99%; (c) R–COOH, HATU, DIPEA, dry EtOAc, r.t., 12–24 h, 60–80%; (d) TMSBr, 3-mercaptopropyl functionalized silica gel, Et_3_N, MeCN, r.t., 12–48 h, 19–94%. For the full structures of 5(a–l), and hence of the R-groups, see Table 1 below.

To synthesize 5a–l, we used a pathway (Scheme 3) that relies on the hafnium chloride catalyzed Kabachnik–Fields reaction,^38^ which simultaneously introduces the phosphonic acid and the amine functional groups, in its key first step. Next, the benzyl protecting group of 2 was hydrogenated under mild conditions using Pearlman's catalyst,^39^ Pd(OH)_2_/C and H_2_ under atmospheric pressure, yielding 3. Subsequently, a variety of amides 4a–l were generated from this intermediate with a range of carboxylic acids (Scheme 3). Out of the variety of coupling reagents and solvents examined, HATU in combination with dry EtOAc as solvent provided the highest yields.^40^ The diastereomers of 4a–l were separated using preparative HPLC, and one set of enantiomeric pairs was crystalized to identify the relative configuration of the intermediates by single-crystal X-ray diffraction (section 5, ESI†). The phosphonic ester was then hydrolyzed with McKenna's method using TMSBr, resulting in simultaneous Boc-deprotection of 4a–l. However, it was observed that ethyl bromide liberated as a side product led to unwanted *N*-alkylation of the target compounds 5a–l. This could be avoided by the addition of Et_3_N in combination with 3-mercaptopropyl functionalized silica gel to the reaction mixture. To enhance the aqueous solubility, the final products were converted to their hydrochloric salts (5a–l). In our hands, the compounds were chemically stable in solution for a prolonged time (weeks to months, Fig. S187–S192, ESI†).

### Metallo-β-lactamase inhibition and cytotoxic activities

The 24 synthesized compounds 5a–l were tested for their inhibitory activities against NDM-1, VIM-2 (Table 1), and GIM-1 in an *in vitro* enzymatic assay (ESI†).^41^ The inhibitory activity was measured using enzyme concentrations of 0.1 nM VIM-2, 10 nM NDM-1 or 1 nM GIM-1, with an excess Zn(ii) (100 μM, see Experimental methods), with inhibitor compounds with concentrations from 0 to 800 μM. Each data point was measured in triplicates. Almost all compounds showed metallo-β-lactamase inhibitory activities with 5g-2 being the most active (IC_50_ = 4.1 μM, VIM-2). A larger number of compounds inhibited VIM-2 than NDM-1 or GIM-1, and those with *RR*/*SS* configuration typically inhibited VIM-2 at lower concentrations. Our observation that a larger number of compounds inhibited VIM-2 than NDM-1 may be unexpected as both enzymes belong to the B1-subclass of metallo-β-lactamases. This is in line with previous reports.^42,43^ In general, the more rigid compounds showed higher affinity (compare 5b to 5j, for instance). Accordingly, upon introduction of a flexible methylene group, inhibitors with a 3-thiophene ring showed a 10-fold reduction of activity, whilst 2-thiophene substituted ones showed up to 45-fold decreased activity. Importantly, none of the compounds showed cytotoxicity against human MCF-7 or HepG cells (section 2, ESI†).

**Table 1:** The NDM-1 and VIM-2 inhibitory activities of compounds 5a–l

No.	Compound	Stereochemistry^a^	Enzyme IC_50_^c^ (μM)	
			NDM-1	VIM-2
5a-1	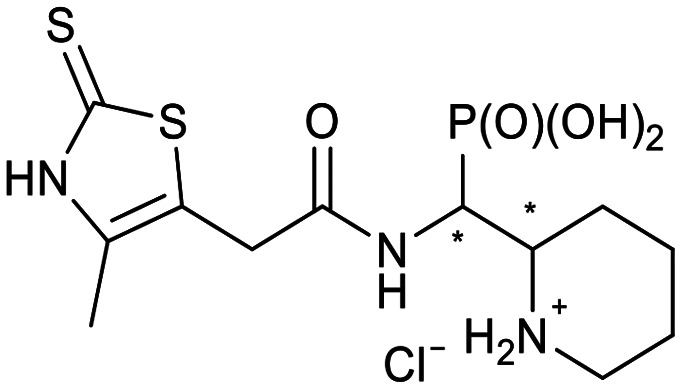	*SR*/*RS*	N.I.^b^	353 ± 62
5a-2		*RR*/*SS*	N.I.	N.I.
5b-1	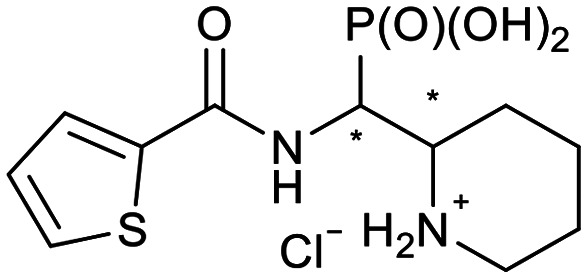	*SR*/*RS*	230 ± 28	118 ± 60
5b-2		*RR*/*SS*	N.I.	7.2 ± 0.4
5c-1	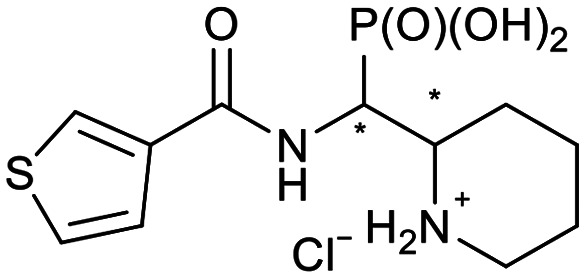	*SR*/*RS*	219 ± 14	33 ± 3
5c-2		*RR*/*SS*	N.I.	14.6 ± 2.3
5d-1	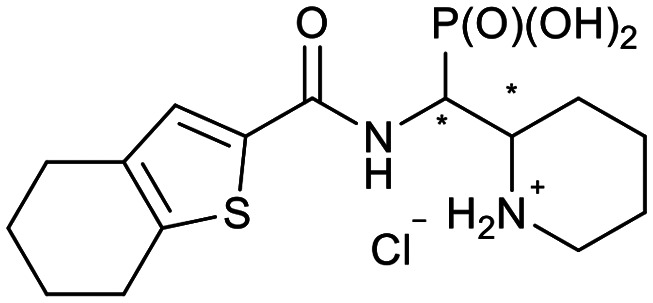	*SR*/*RS*	N.I.	244 ± 35
5d-2		*RR*/*SS*	N.I.	4.5 ± 0.8
5e-1	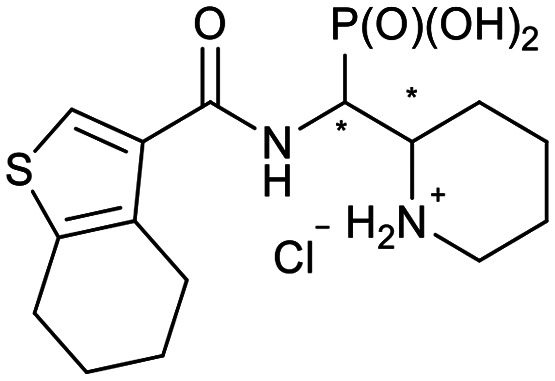	*SR*/*RS*	76 ± 12	69.7 ± 26.2
5e-2		*RR*/*SS*	N.I.	46.1 ± 15.6
5f-1	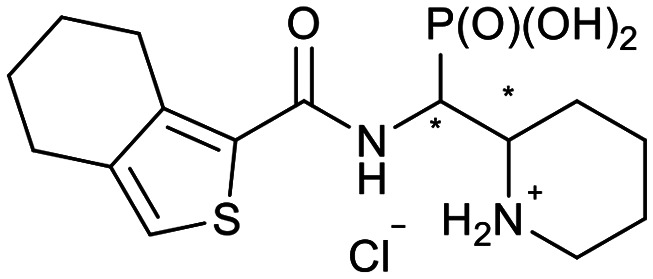	*SR*/*RS*	N.I.	37 ± 3
5f-2		*RR*/*SS*	N.I.	18.8 ± 0.5
5g-1	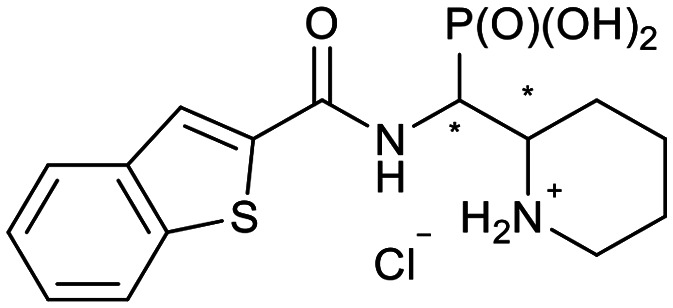	*SR*/*RS*	46.7 ± 9.7	45 ± 5
5g-2		*RR*/*SS*	46.8 ± 5.5	4.1 ± 2.1
5h-1	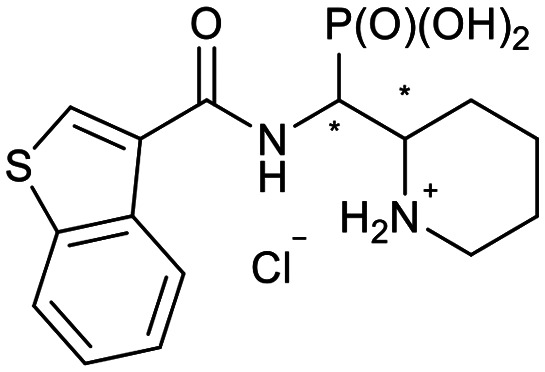	*SR*/*RS*	N.I.	40.6 ± 3.9
5h-2		*RR*/*SS*	N.I.	11.6 ± 4.7
5i-1	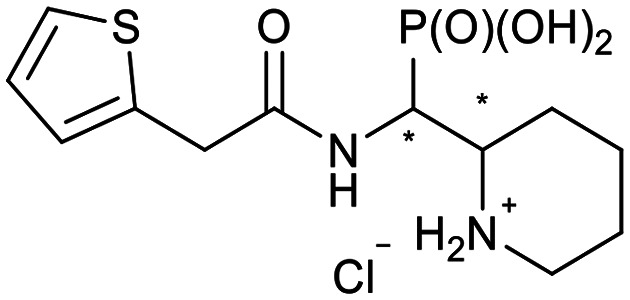	*SR*/*RS*	296 ± 87	N.I.
5i-2		*RR*/*SS*	271 ± 34	328 ± 116
5j-1	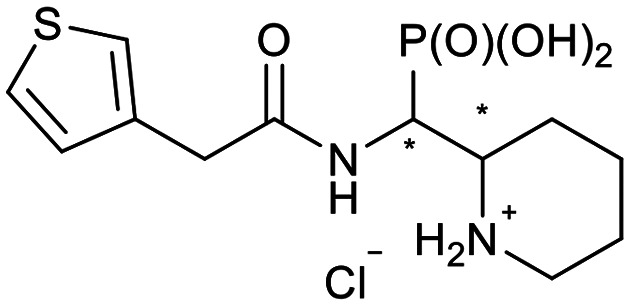	*RS*/*SR*	506 ± 333	319 ± 69
5j-2		*RR*/*SS*	N.I.	N.I.
5k-1	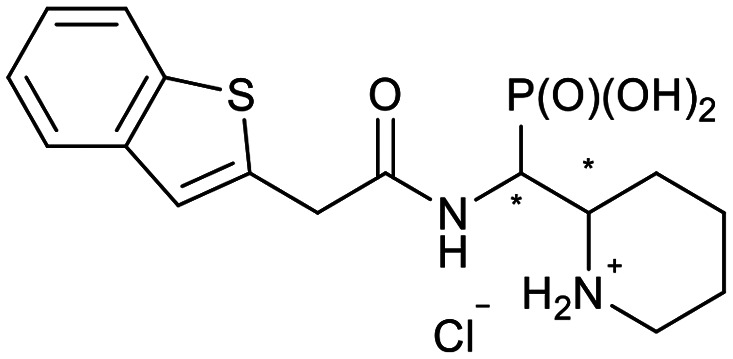	*SR*/*RS*	N.I.	56.6 ± 1.0
5k-2		*RR*/*SS*	22.4 ± 6.3	28.7 ± 10.1
5l-1	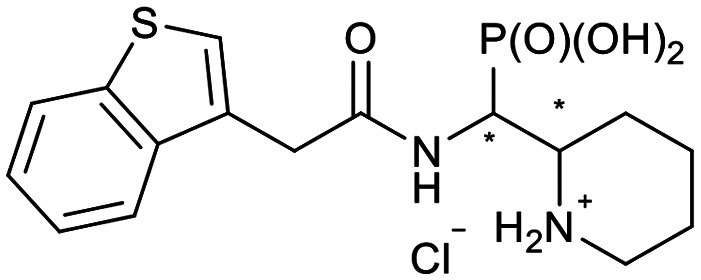	*RR*/*SS*	7.9 ± 1.6	N.I.
5l-2		*SR*/*RS*	27.8 ± 21.0	94.4 ± 5.8

a The compounds were separated as diastereomeric pairs, which are shown here with the stereocenters being highlighted with *.

b N.I. means no enzyme inhibitory activity was observed.

c IC_50_ values were calculated from 3 parallel measurements.

### Bacterial membrane permeability

**Fig. 3:**
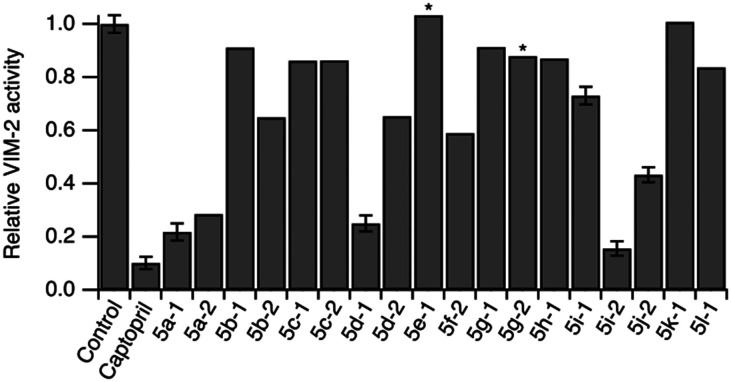
Normalized relative activity of VIM-2 encapsulated in outer membrane vesicles in absence (control) and presence of compounds. Error bars represent S.D. of 3 measurements. * indicates ambiguous readout due to compound precipitation. Compound concentrations are specified in Table S189.†

To evaluate the compounds' ability to cross bacterial outer membranes we prepared outer membrane vesicles carrying VIM-2 inside their lumen.^44^ Outer membrane vesicles maintain the protein as well as the lipid composition of the bacterial membrane they originate from, and thus serve as an *in situ* model system for assessing transmembrane uptake.^45^ The activity of VIM-2 encapsulated inside the outer membrane vesicles was determined using the colorimetric β-lactamase substrate CENTA in presence and absence of the compounds.^46^ We observed a reduction in VIM-2 activity for most compounds (Fig. 3), in agreement with the *in vitro* assay, indicating that they effectively cross the Gram-negative outer membrane.

### Protein–inhibitor interaction studies

Solution NMR spectroscopy is a powerful tool to assess protein–ligand interactions under close to physiological conditions.^47^ We, therefore, performed NMR chemical shift perturbation (CSP, for details, see section S8, ESI†) experiments to identify the amino acid residues of NDM-1 and VIM-2 that are involved in inhibitor binding, making use of the previously published protein assignments.^48,49^ Titrations were conducted using ^15^N-labelled proteins with 0, 0.5, 1, 2, 4, 8, and 16 equiv. of 5 inhibitors for NDM-1 and of 7 inhibitors for VIM-2.

As performing NMR titrations for all inhibitors in combination with both proteins was deemed unrealistic, we selected compounds based on their inhibitory activity and aqueous solubility. Titrations with concentrated ligand samples were preferred to minimize dilution-induced chemical shift changes. Titrations were carried out with NDM-1 and VIM-2 due to their high clinical relevance.

**Fig. 4:**
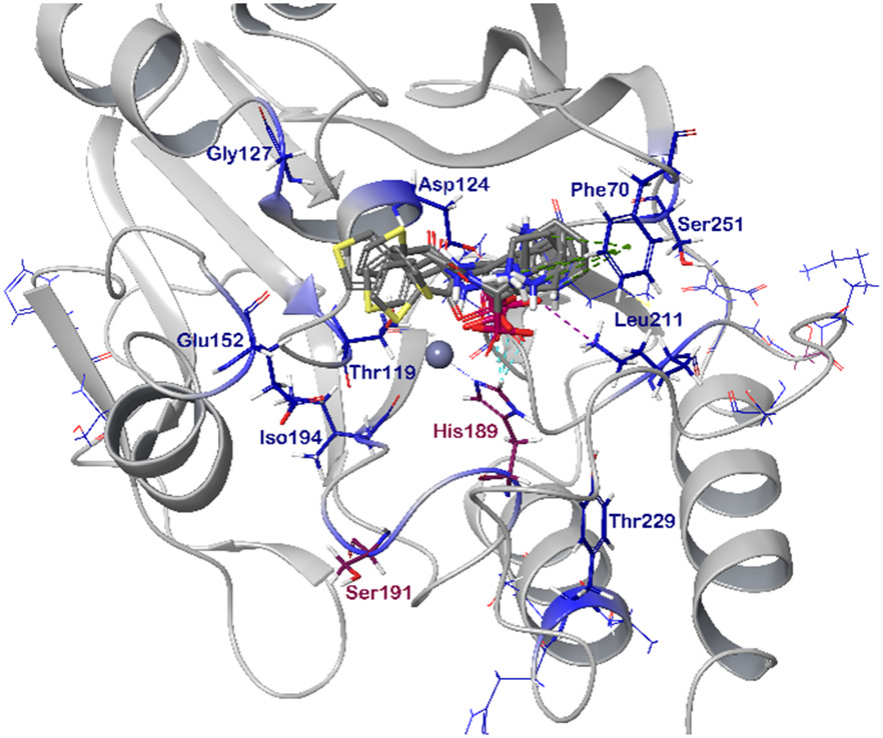
The chemical shift perturbation of the backbone amides (CSP) of ^15^N-labelled NDM-1 (pdb: 6O3R) upon addition of 16 equivalents of 5b-1/2, 5c-1/2, 5i-2 and 5j-2 (grey). CSPs greater than the population mean and one standard deviation (*μ* + 1*σ*) were considered significant and are colored. Amino acid residues highlighted in blue were influenced by binding of each inhibitor and thus are essential for binding. Those highlighted in purple were affected in the majority of the titrations.

Throughout the titrations of NDM-1 with selected inhibitors (Table 2), Phe70, Asp124, and Lys211 surrounding the suggested β-lactam binding site showed significant chemical shift perturbations (Fig. 4 and S164–S168, ESI†). We also observed large chemical shift perturbations for Ser191, which has previously been reported to take part in substrate binding (numbered as Ser198).^44,50^ The observed spectral changes are characteristic for binding whereas not compatible with zinc(ii)-depletion of the enzyme.^44,51^ Some amino acid residues in the C-terminal increased in intensity with increasing concentration of the added inhibitor, suggesting binding-induced alteration corresponding to the rate of the conformational dynamics of this enzyme.^52^

**Table 2:** NDM-1 and VIM-2 binding affinities as determined *via* NMR chemical shift perturbation experiments^a^

Protein	NDM-1 *K*_d_ [mM]	VIM-2 *K*_d_ [mM]
Compound		
5a-1	n.d.	0.9 ± 0.1
5b-1	2.0 ± 0.1	0.4 ± 0.04
5b-2	n.d.	1.4 ± 0.1
5c-1	3.1 ± 0.3	0.4 ± 0.03
5c-2	n.d.	n.d.
5i-1	0.5 ± 0.1	1.5 ± 0.2
5i-2	1.4 ± 0.1	1.1 ± 0.1
5j-1	2.2 ± 0.2	0.9 ± 0.1

a For details, see section S8 of the ESI;† n.d., not determined.

**Fig. 5:**
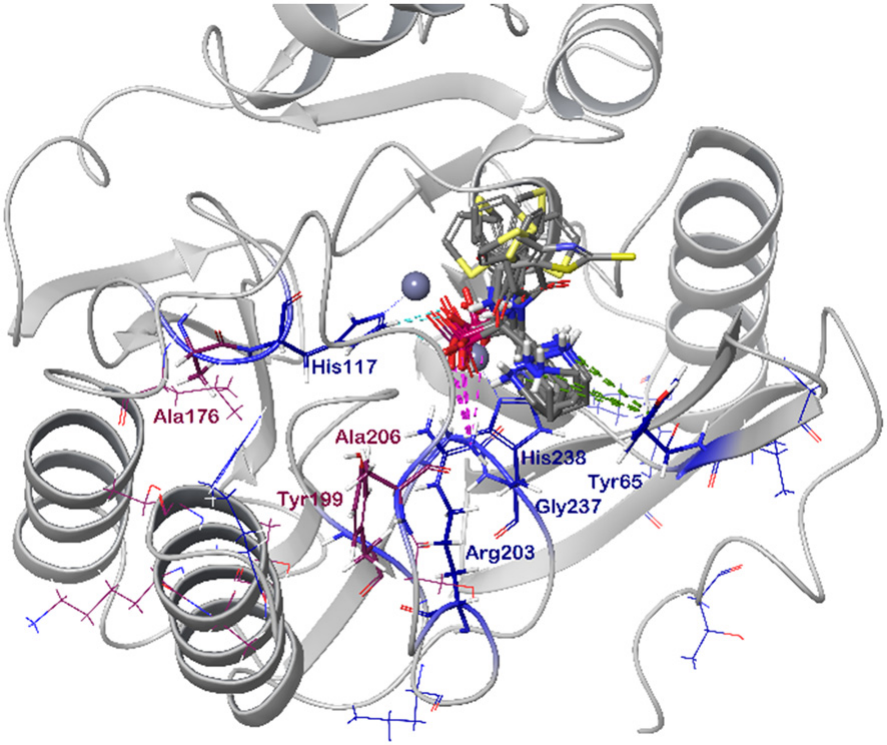
The chemical shift perturbation (CSP) of the backbone amides of ^15^N-labelled VIM-2 (pdb: 6O5T) upon titration with ligands 5b-1/2, 5c-1/2, 5i-1/2 and 5j-1 (grey). CSPs greater than the population mean plus one standard deviation (*μ* + 1*σ*) were considered significant and are colored as for Fig. 4.

We observed significant chemical shift perturbations for Tyr65, His177, Tyr199, Arg203, Ser205, and His238 of VIM-2, in the expected binding site (Fig. 5 and S169–S174, section S7, ESI†), and for a few additional amino acid residues at the N-terminal, the latter presumably due to binding induced conformational change of the enzyme. The line broadening was observed for Tyr65, Gly69, Thr83, His177, Tyr199, Ser202, Ser205, Ala206, His238, and Gly239 throughout at least one of the titrations, indicating the presence of a process with intermediate rate exchange, possibly related to the binding of the inhibitor and/or a binding induced conformational change.

**Fig. 6:**
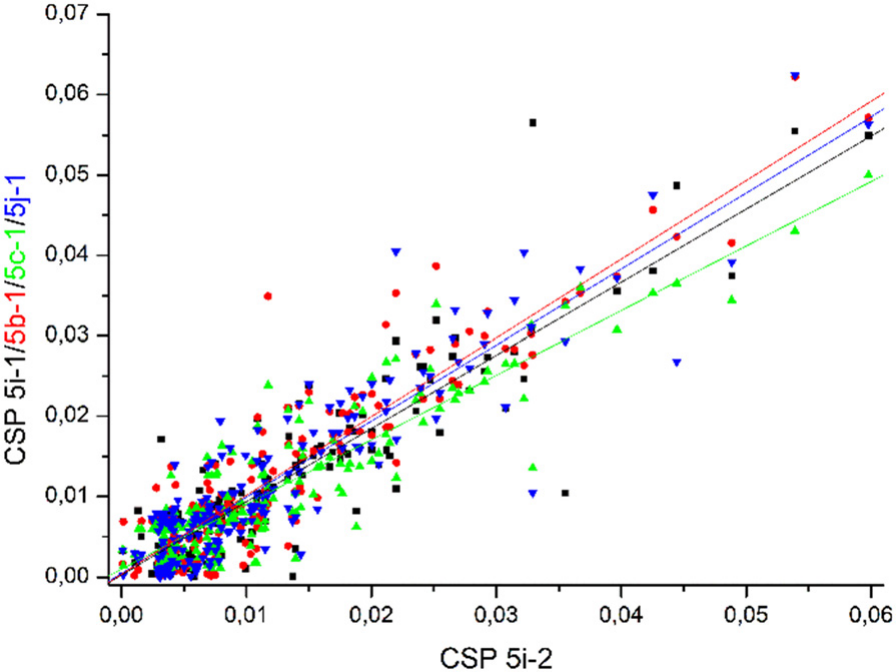
The chemical shift perturbation of NDM-1 upon titration with 5b-1, 5c-1, 5i-1, and 5j-1 correlates to that of 5i-2 with *R*^2^ ∼ 0.81–0.84, suggesting comparable binding modes of all ligands. For details, see Fig. S175–S178, ESI.†

**Fig. 7:**
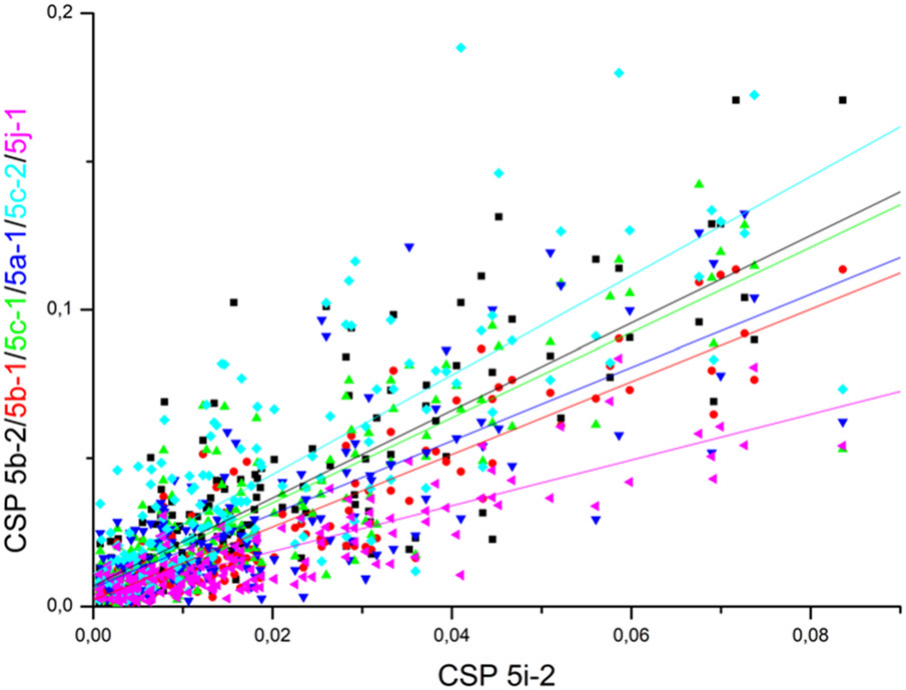
The chemical shift perturbation of VIM-2 upon addition of 16 equivalents of 5b-1/5b-2, 5c-1, 5a-1, 5c-2 and 5j-1 correlate to that observed upon titration with 5i-2 (*R*^2^ ∼ 0.61–0.82), suggesting comparable binding modes for the ligands. For details, see Fig. S179–S184, ESI.†

Comparable chemical shift perturbations were observed for NDM-1 upon titrations with 5i-1, 5b-1, 5c-1 and 5j-1 to that observed for 5i-2 (*R*^2^ ∼ 0.81–0.84, Fig. 6), a compound that inhibits both NDM-1 and VIM-2, and revealed that the α-amino-phosphonate inhibitors bind to the same binding cleft and have comparable binding modes. Due to the structural differences leading to certain amino acid residues being involved in the binding of one but not of another inhibitor, the chemical shift perturbations are not expected to show perfect correlation. However, a reasonable correlation reveals the involvement of certain amino acid residues in the binding of all inhibitors studied herein. We also observed correlation of the chemical perturbations of the ^1^H,^15^N resonances of the amino acid residues of VIM-2 upon titration with 5b-1/5b-2, 5c-1, 5a-1, 5c-2 and 5j-1 to that observed for 5i-2 (Fig. 7).

**Fig. 8:**
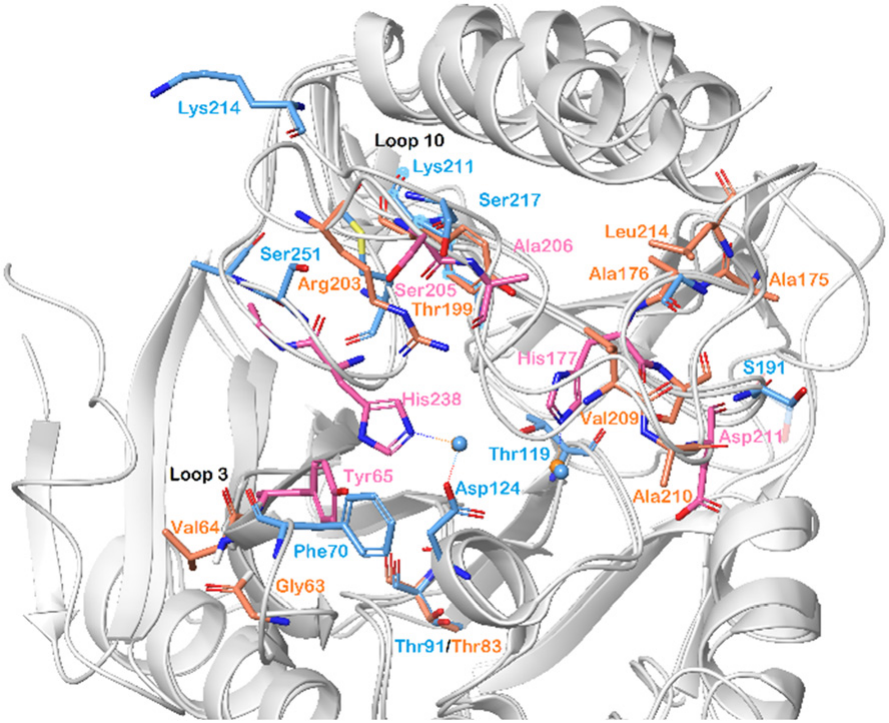
Chemical shift perturbations observed for NDM-1 (blue, pdb: 6NY7) and VIM-2 (pink and orange, pdb: 6O5T), shown superimposed, upon titration with 5b-1 revealed the amino acid residues that are important for α-aminophosphonate-type inhibitor binding for both metallo-β-lactamases. The zinc ions are shown as blue spheres.

By superimposing the chemical shift perturbations observed for NDM-1 and VIM-2 upon titration with 5b-1, the amino acid residues important for binding of these compounds to both enzymes were identified (Fig. 8). Hence, we observed that His177 and His238 of VIM-2, and Asp124 of NDM-1, holding the zinc ions of the catalytic sites, showed large chemical shift perturbations. Moreover, Phe70/Tyr65 (NDM-1/VIM-2) of the lipophilic loop 3 and Lys214/Arg203 (NDM-1/VIM-2) of loop 10 are also pointed out as key residues for inhibitor binding.

The sequestration of Zn^2+^ ions from NDM-1 or VIM-2 were reported to cause extensive changes in their ^1^H,^15^N HSQC spectrum.^44^ Importantly, such large spectral alterations have not been observed at the titrations described above, which indicates that 5a–j do not act by metal sequestration, but rather by ligand replacement in the active site.

### Computational docking

**Fig. 9:**
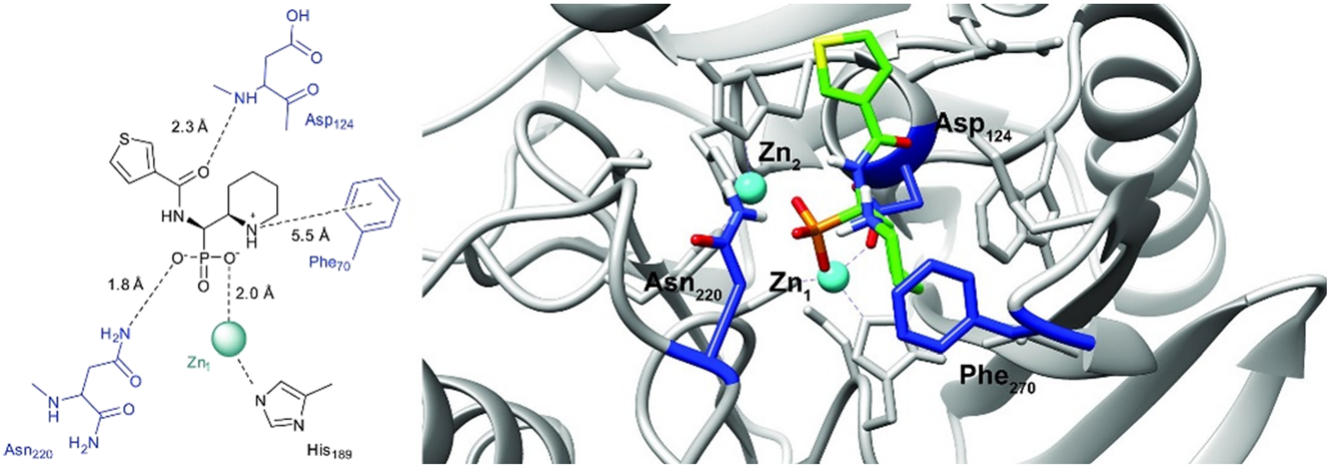
The interactions of (*RS*)-5c-1 with the active site of NDM-1 (pdb: 6NY7), as determined by computational docking directed by NMR (ligand induced protein chemical shift perturbation). The inhibitor is shown in green, the amino acids involved in the interaction in blue whereas the zinc(ii) ions in cyan. The lowest Δ*G*_bind_ docked positions of all inhibitor candidates (pdb) are provided as ESI, uploaded to the open access repository Zenodo (DOI: https://doi.org/10.5281/zenodo.6971841).

**Fig. 10:**
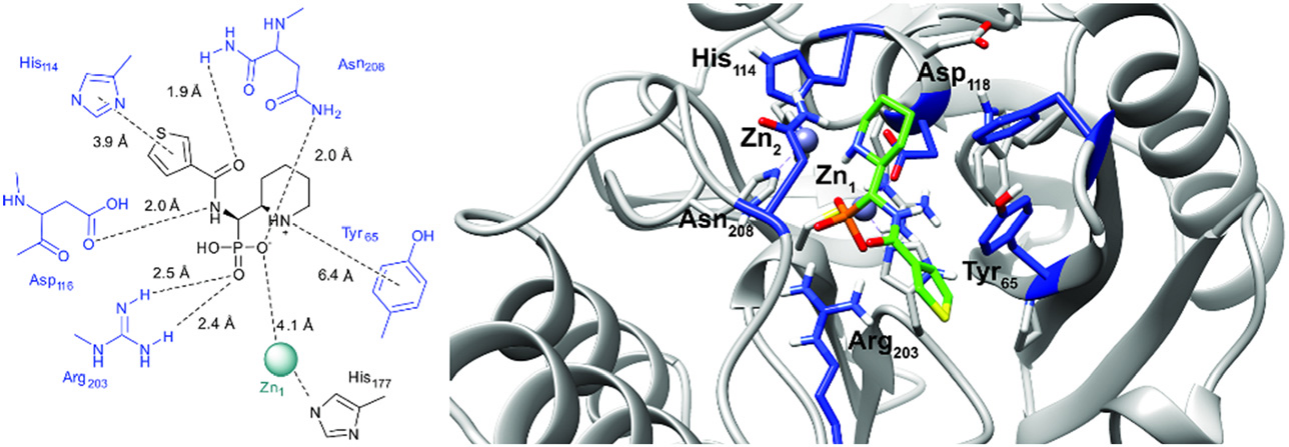
The interactions of (*RS*)-5c-1 with the active site of VIM-2 (pdb: 6O5T), as determined by computational docking directed by NMR (ligand induced protein chemical shift perturbation). The inhibitor is shown in green, the amino acids involved in the interaction in blue whereas the zinc(ii) ions in cyan. The lowest Δ*G*_bind_ docked positions of all inhibitor candidates (pdb) are provided as ESI, uploaded to the open access repository Zenodo (DOI: https://doi.org/10.5281/zenodo.6971841).

In order to visualize the binding of the studied inhibitors, we performed flexible docking for the compounds that have been selected for NMR titration above, using the software Glide (Schrödinger Inc.). The calculations were started from the experimental protein structures pdb 6O3R^32^ and pdb 6O5T,^32^ followed by MM-GBSA rescoring, which was selected as it is known as the quickest force-field based method for calculation of the free energy of binding.^53^ Ligands were prepared with LigPrep (Schrödinger Inc.), generating all possible stereoisomers and reasonable protonation states at pH 7 ± 2. Factors such as tautomerisation, metal binding, and the presence or absence of salts were also taken into account. To avoid bias or forced positions, docking was performed up to 40 times for each generated state for each ligand without experimental restraints. The resulting docking modes were evaluated in comparison to the experimentally observed ^1^H,^15^N NMR chemical shift perturbations (see the ESI†) and those compatible with the experimental data were kept. Next, they were ranked according to their binding free energies (Δ*G*) obtained by MM-GBSA rescoring, and the most feasible binding pose was selected. The stronger binding of the *RS* as compared to the *SR*, and of the *RR* as compared to the *SS* diastereomers of the studied inhibitors to both VIM-2 and NDM-1 (Tables S8 and S9, ESI†) was observed, as a general trend. This suggests that these stereoisomers (*RS*, *RR*) are primarily responsible for the enzyme inhibitory activities are shown in Table 1. The superposition of the binding modes (Fig. 4, VIM-2, and Fig. 5, NDM-1, pdb coordinates are provided as ESI†) indicates that the α-aminophosphonate-type inhibitors coordinate Zn1 of both NDM-1 (Fig. 9) and VIM-2 (Fig. 10) *via* an oxygen of the phosphonic acid. This computational observation is corroborated by the line broadening of the ^1^H,^15^N HSQC signal of His177 that directly coordinates Zn-1, and is in agreement with our design (inhibition by a tetrahedral phosphonate). Moreover, another oxygen of the phosphonic acid forms a hydrogen bond with Asn220 of NDM-1 or a salt bridge to Arg203 of VIM-2. The piperidine nitrogen of the inhibitors is involved in a π–cation interaction^53,54^ with Phe70 of NDM-1 or Tyr65 in case of VIM-2, whereas the thiophene ring of the inhibitors is oriented towards loop 10 of the enzyme. The binding poses of the inhibitors are similar, as revealed by the average RMSD^55^ of 2.6 ± 0.1 Å for NDM-1, and 2.8 ± 0.4 Å for VIM-2 binding (Fig. 4 and 5). The latter similarity is expected for structurally related inhibitors, and is supported by the good correlation of the NMR chemical shift perturbations observed for the same protein upon titration with different α-aminophosphonates (Fig. 6 and 7). Overall, the energetically preferred binding modes (Fig. 9 and 10) are in good agreement with the NMR chemical shift perturbations.

**Fig. 11:**
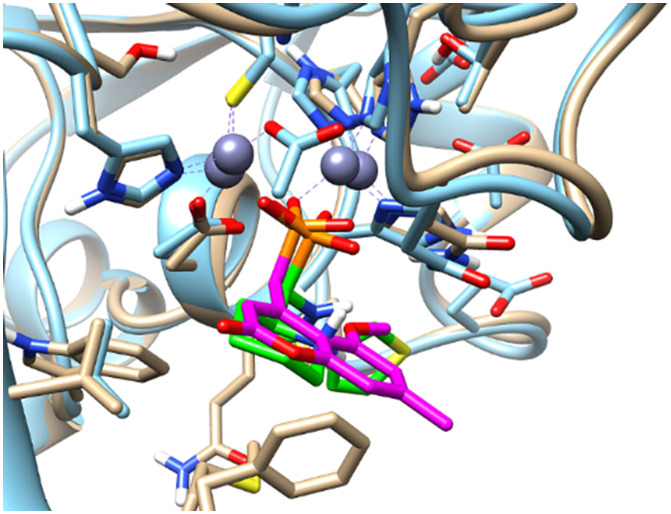
Superimposition of the computationally predicted binding mode of (*RS*)-5c-1 (green) to NDM-1 (grey) with the X-ray structure of [(5-methoxy-7-methyl-2-oxo-2*H*-chromen-4-yl)methyl]-phosphonic acid (magenta) in complex with NDM-1 (blue, PDB code 6D1E).^32^

Pemberton *et al.*^32^ have shown that the binding pose of phosphonate-bearing coumarin inhibitors of NDM-1 may be pH dependent. At pH 7.5, the active site hydroxide is retained, and the phosphonate group interacts with this hydroxide and Zn2, rather than with both Zn(ii) ions as at lower pH. The NMR titrations reported herein were performed at pH 7, however, based on the ^15^N NMR chemical shift perturbation data the possibility that 5a–l could bind *via* a bridging hydroxide ion cannot be excluded. The binding poses of phosphonates presented herein and of the coumarin phosphonate derivatives of Pemberton, an example being depicted in Fig. 11, show noticeable similarities.

Comparing our data, we note that the ligand-induced chemical shift perturbation observed upon titration with 5a–l showed a wider spread of chemical shift changes for VIM-2 (Fig. 6) as compared to NDM-1 (Fig. 7), and that the binding modes of the inhibitors to VIM-2 are being slightly more dissimilar (Fig. 4) as compared to those of NDM-1 (Fig. 5). This may indicate somewhat higher flexibility of VIM-2 as compared to NDM-1, which may allow it to offer a larger degree of substrate promiscuity. This is corroborated by the observation of a larger number of α-aminophosphonate inhibitors showing activity against VIM-2 than NDM-1 in this study.

## Conclusions

We designed and synthesized a series of α-aminophosphonate-type inhibitors for NDM-1 and VIM-2. Their evaluation in enzyme assays revealed several compounds to possess <10 μM inhibitory activity (IC_50_) against these metallo-β-lactamases, while showing no cytotoxicity, indicating their potential for further development. These compounds were designed to enhance the activity of β-lactam antibiotics, such as meropenem. Being the first members of a new type of inhibitors, they showed moderate to low inhibitory potency and are obviously not directly applicable in the clinical treatment of NDM-1 and VIM-2 expressing bacterial strains. As none of the enantiomers of 5a–l can be expected to have nM activity, it was not worth to separate or selectively generate their isomers. The development of stereoselective synthetic routes to α-aminophosphonates will therefore be the task for upcoming studies at the identification of more active members of this structural group. This may be achieved, for instance, by the structural modifications of the piperidine moiety of 5a–l. Hence, whereas these compounds aren't clinically applicable, their future structural modifications may yield compounds applicable for the treatment of NDM-1 and VIM-2 expressing bacterial strains. The inhibitor binding sites were indicated by ^1^H/^15^N NMR chemical shift perturbation experiments, evaluating the data of selected compounds in the series. Comparable magnitudes of chemical shift perturbation on the same amino acid residues upon titration with differently substituted inhibitors indicated that the α-aminophosphonic acid derivatives have a common binding site on both enzymes. Computational docking revealed that the *RS* and *RR* stereoisomers of the synthesized inhibitors are primarily responsible for NDM-1 and VIM-2 binding (*K*_d_ = 0.4–3.1 mM). A stronger correlation of the inhibitor induced chemical shift perturbations on NDM-1 as compared to that observed for VIM-2 may indicate a lower substrate specificity of VIM-2. This was corroborated by the observation of VIM-2 binding a larger number of phosphonates with more different binding poses, as determined by NMR guided computational docking. The amino acid residues Phe70, Asp124, Lys211, Ser191 and Ser249 for NDM-1, and Tyr65, His177, Tyr199, Arg203, Ser205, and His238 for VIM-2 Phe70/Tyr65 (VIM-2/NDM-1) were suggested to be necessary for inhibitor binding. This structural knowledge is expected to be useful for the development of future wide spectrum metallo-β-lactamase inhibitors. Accordingly, the compounds presented herein may be useful in fragment-based drug discovery, in addition to possibly providing a starting point for the development clinically applicable metallo-β-lactamase inhibitors.

## Experimental methods

### General methods

Reagents and reactants for synthesis were purchased from commercial suppliers (Sigma-Aldrich, Merck, Fluorochem, VWR, Arcos, Fischer Scientific) and were used without further purification. Reactions were monitored by LCMS (Agilent 1100 Series) equipped with an ESI-MS detector (Waters Micromass ZQ 2000) with a UV range of 200–600 nm, and an analytical column (Phenomenex, Gemini C18 column, 5 μm, 110 Å, ∅ 3.00 mm, L 50 mm) or by TLC-MS (API, Advion Express). Purification of the compounds was performed on Merck silica gel (0.035–0.079 mm, 5 Å) by manual column chromatography or by using Biotage Isolera One flash column chromatography system with solvent gradients. Additionally, diastereomers were separated using preparative RP-HPLC (VWR LaPrep P110) with single wavelength detection, using a Kromasil C8 column (10 μm, 100 Å, ∅ 39 mm, L 250 mm), and chiral column Phenomenex Lux Amylose-1 (5 μm, 1000 Å, ∅ 21.2 mm, L 250 mm) and gradients of CH_3_CN/H_2_O with/without 0.1% formic acid as mobile phase at 10/15 mL min^−1^ flow rate. NMR spectra of the synthetic intermediates were recorded at room temperature on a Varian Agilent MR400-DD2 with OneNMR probe (^1^H, 400 MHz), and a Bruker Avance Neo with TXO cryogenic probe (^1^H, 500 MHz). In order to assign diastereomers and rotamers, diastereomers were separated by preparative chiral HPLC. Protein-related spectra (series of ^1^H–^15^N HSQC spectra was used for the CSP experiment) were recorded in 90% H_2_O/10% D_2_O + 2.5% DMSO-*d*_6_ with a protein concentration of 0.25 mM at a Bruker Avance Neo (^1^H, 600 MHz) with TCI cryogenic probe, using 3 mm NMR tubes.

### Crystallography

Crystallization of 4a–l was done by vapour diffusion or by layering. Layering dioxane over H_2_O at 12 °C provided single crystals suitable for X-ray diffraction analysis. Vapour diffusion was performed with THF/cyclohexane or methylformate/*n*-pentane at −20 °C, 12 °C, or at r.t. The crystallographic data were collected with either a Rigaku SuperNova single-source diffractometer equipped with an Eos CCD detector using mirror-monochromated Mo Kα (*λ* = 0.71073 Å) or a Rigaku SuperNova dual wave-length diffractometer equipped with an Atlas CCD area detector with Cu-Kα radiation (*λ* = 1.54184 Å). Data collection, reduction, and Gaussian or analytical face-index based absorption correction were performed with CrysAlisPro.^56^ Structures were solved using SHELXT^57^ and refined by full-matrix least-squares on *F*^2^ using SHELXL.^58^ In all data, anisotropic displacement parameters were introduced for all atoms except hydrogens, which were calculated or refined into their ideal positions using isotropic displacement parameters of 1.2 or 1.5 times that of the host atom. The figures were made using the Mercury program.^59^ The structures have been deposited to CCDC with codes 2203659 (4a-2), 2203660 (4b-2), 2203661 (4c-2), 2203662 (4d-2), 2203663 and 2203670 (4e-2), 2203664 (4f-1), 2203665 (4g-1), 2203666 (4h-2), 2209101 (4i-1), 2203667 (4j-1), 2203668 (4k-1), and 2203669 (4l-1). For a detailed description of the crystallographic data, see section S5 of the ESI.†

### Synthetic procedures

The synthetic route towards 5a–l-1/2 is summarized in Scheme 3, and the details are given below.

### *tert*-Butyl 2-((benzylamino)(diethoxyphosphoryl)methyl)-piperidine-1-carboxylate (2)

Compound 1 (3.55 g, 16.7 mmol), benzylamine (1.84 mL, 16.8 mmol, 1.0 equiv.) and diethyl phosphite (2.19 mL, 17.0 mmol, 1.0 equiv.) were dissolved in absolute EtOH (17 mL). HfCl_4_ (107 mg, 2 mol%) was added in one portion and the reaction was stirred at 60 °C for 12 h under an inert atmosphere. The solvent was removed under reduced pressure and the crude product was purified by FCC (SiO_2_, gradient EtOAc in hexane 80% to 100%), *R*_f_ (EtOAc : Hex 4 : 1) = 0.47. The product was obtained as a pale yellow solid (2.36 g, 5.35 mmol, 32%). HRESIMS *m*/*z* 441.2529 [M + H]^+^ (441.2513 calcd for C_22_H_38_N_2_O_5_P).

### Compound 2, diastereomer 1 (rotamer ratio 1 : 1)

^1^H NMR (500 MHz, CDCl_3_) *δ* 7.31–7.27 (m, 4H, 11-H, 12-H, 14-H, 15-H), 7.22 (m, 1H, 13-H), 4.53 (br. s, 1H, 2-H), 4.26–4.16 (m, 4H, 1-P(OCH_2_)_2_), 4.07 (d, ^2^*J* = 12.9 Hz, 1H, part A of AB, 9-H), 3.98 (br. d, *J* = 14.1 Hz, 1H, 4-H), 3.78 (d, ^2^*J* = 12.9 Hz, 1H, part B of AB, 9-H), 3.24 (dd, ^2^*J*_*HP*_ =10.6 Hz, *J* = 10.6 Hz, 1H, 1-H), 2.27 (m, 1H, 4-H), 2.14 (br. d, *J* = 13.8 Hz, 1H, 7-H), 1.71–1.42 (m, 5H, 5-H_2_, 6-H_2_, 7-H), 1.38 (s, 1H, 9H, 2-NC_q_(O)OC_q_–(CH_3_)_3_), 1.37–1.32 (m, 6H, 1-P(OCH_2_CH_3_)_2_). ^13^C NMR (126 MHz, CDCl_3_) *δ* 155.5 (2-N–C_q_(O)–O), 140.0 (10-C_q_), 128.7 (11-CH, 15-CH or 12-CH, 14-CH), 128.5 (11-CH, 15-CH or 12-CH, 14-CH), 127.3 (13-CH), 79.9 (2-NC_q_(O)O–C_q_–(CH_3_)_3_), 62.6 (d, ^2^*J*_*CP*_ = 7.4 Hz, 1-POCH_2_), 62.1 (br. s., 1-POCH_2_), 53.8 (9-CH_2_), 53.4 (d, ^1^*J*_*CP*_ = 152.4 Hz, 1-CH), 51.0 (d, ^2^*J*_*CP*_ = 13.8 Hz, 2-CH), 38.9 (4-CH_2_), 28.6 (2-NC_q_(O)OC_q_–(CH_3_)_3_), 27.1 (7-CH_2_), 25.2 (5-CH_2_), 19.5 (6-CH_2_), 16.8 (d, ^3^*J*_*CP*_ = 5.4 Hz, 1-POCH_2_CH_3_), 16.8 (d, ^3^*J*_*CP*_ = 4.8 Hz, 1-POCH_2_CH_3_). ^31^P NMR (162 MHz, CDCl_3_) *δ* 27.02.

### Compound 2, diastereomer 2

^1^H NMR (500 MHz, CDCl_3_) *δ* 7.37–7.27 (m, 4H, 11-H, 12-H, 14-H, 15-H), 7.25 (m, 1H, 13-H), 4.33 (ddd, *J* = 13.7, 4.4, 4.4 Hz, 1H, 2-H), 4.22–4.07 (m, 4H, 1-P(OCH_2_)_2_), 4.06 (dd, ^2^*J* = 12.7 Hz, *J* = 1.7 Hz, 1H, part A of AB, 9-H), 3.98 (m, 1H, 4-H), 3.86 (dd, ^2^*J* = 12.7 Hz, *J* = 2.2 Hz, 1H, part B of AB, 9-H), 3.33 (dd, *J* = 14.0, 8.7 Hz, 1H, 1-H), 2.96 (dd, *J* = 11.8, 11.8 Hz, 1H, 4-H), 2.09 (m, 1H, 7-H), 1.59–1.37 (m, 5H, 5-H_2_, 6-H_2_, 7-H), 1.44 (s, 9H, 2-NC_q_(O)OC_q_–(CH_3_)_3_), 1.34 (t, *J* = 7.1 Hz, 3H, 1-POCH_2_CH_3_), 1.33 (t, *J* = 7.0 Hz, 3H, 1-POCH_2_CH_3_). ^13^C NMR (126 MHz, CDCl_3_) *δ* 154.8 (2-N–C_q_(O)–O), 140.2 (10-C_q_), 128.7 (11-CH, 15-CH or 12-CH, 14-CH), 128.5 (11-CH, 15-CH or 12-CH, 14-CH), 127.28 (13-CH), 79.4 (2-NC_q_(O)O–C_q_–(CH_3_)_3_), 61.9 (d, ^2^*J*_*CP*_ = 8.1 Hz, 1-POCH_2_), 61.8 (d, ^2^*J*_*CP*_ = 8.1 Hz, 1-POCH_2_), 54.3 (d, ^1^*J*_*CP*_ = 139.6 Hz, 1-CH), 53.3 (9-CH_2_), 51.7 (br. s, 2-CH), 40.2 (br. s, 4-CH_2_), 28.6 (2-NC_q_(O)OC_q_–(CH_3_)_3_), 24.8 (5-CH_2_), 24.7 (d, ^3^*J*_*CP*_ = 7.9 Hz, 7-CH_2_), 19.4 (6-CH_2_), 16.7 (d, ^3^*J*_*CP*_ = 5.8 Hz, 1-POCH_2_CH_3_), 16.7 (d, ^3^*J*_*CP*_ = 5.7 Hz, 1-POCH_2_CH_3_). ^31^P NMR (162 MHz, CDCl_3_) *δ* 27.35.

### *tert*-Butyl 2-(amino(diethoxyphosphoryl)methyl)piperi-dine-1-carboxylate (3)

Compound 2 (2.36 g, 5.35 mmol) and Pd(OH)_2_/C, 50% wet (10% w/w, 1.28 g, 0.91 mmol, 0.17 equiv.) were dissolved in EtOH (54 mL). A strong argon flow was used to bubble argon through the reaction mixture for 5 min. Subsequently, H_2_ was bubbled through the solution for 30 s and the reaction vial was equipped with an H_2_-filled balloon. The reaction was stirred at r.t. for 16 h. The reaction mixture was filtered through a plug of Celite and a syringe filter to remove Pd traces. The solvent was removed under reduced pressure. The product was obtained as a colorless liquid (1.87 g, 5.33 mmol, 99%). HRESIMS *m*/*z* 351.2066 [M + H]^+^ (351.2043 calcd for C_15_H_32_N_2_O_5_P).

### Compound 3, diastereomer 1

^1^H NMR (400 MHz, CDCl_3_) *δ* 4.32 (m, 1H, 2-H), 4.13 (q, *J* = 7.1 Hz, 2H, 1-POCH_2_ or 1-P(OCH)_2_), 4.13 (q, *J* = 7.1 Hz, 2H, 1-POCH_2_ or 1-P(OCH)_2_), 4.00 (m, 1H, 4-H), 3.42 (dd, ^1^*J*_*HP*_ = 10.4 Hz, *J* = 10.4 Hz, 1H, 1-H), 2.89 (dd, *J* = 12.1, 12.1 Hz, 1H, 4-H), 2.09 (br. d, *J* = 11.7 Hz, 1H, 7-H), 1.67–1.37 (m, 5H, 5-CH_2_, 6-H_2_, 7-H), 1.45 (s, 9H, 2-NC_q_(O)OC_q_–(CH_3_)_3_), 1.31 (t, *J* = 6.8 Hz, 6H, 1-P(OCH_2_CH_3_)_2_).^13^C NMR (101 MHz, CDCl_3_) *δ* 154.7 (2-N–C_q_(O)–O), 79.6 (2-NC_q_(O)O–C_q_–(CH_3_)_3_), 62.3 (d, ^2^*J*_*CP*_ = 7.3 Hz, 1-POCH_2_), 62.1 (d, ^2^*J*_*CP*_ = 7.3 Hz, 1-POCH_2_), 51.9 (d, ^2^*J*_*CP*_ = 2.5 Hz, 2-CH), 48.2 (d, ^1^*J*_*CP*_ = 147.0 Hz, 1-CH), 40.3 (br. s, 4-CH_2_), 28.6 (2-NC_q_(O)OC_q_–(CH_3_)_3_), 25.0 (5-CH_2_), 24.8 (d, ^3^*J*_*CP*_ = 9.4 Hz, 7-CH_2_), 19.3 (6-CH_2_), 16.7 (d, ^3^*J*_*CP*_ = 3.8 Hz, 1-POCH_2_CH_3_), 16.6 (d, ^3^*J*_*CP*_ = 4.1 Hz, 1-POCH_2_CH_3_). ^31^P NMR (162 MHz, CDCl_3_) *δ* 27.42.

### Compound 3, diastereomer 2 (rotamer ratio 1 : 1.3)

^1^H NMR (400 MHz, CDCl_3_) *δ* 4.36 (br. s, 1H, 2-H), 4.24–4.08 (m, 5H, 1-P(OCH_2_)_2_, 4-H), 3.37 (dd, ^2^*J*_*HP*_ = 11.6 Hz, *J* = 11.6 Hz, 1H, 1-H), 2.59 (br. dd, *J* = 12.7, 12.7 Hz, 1H, 4-H), 2.16 (br. d, *J* = 13.0 Hz, 1H, 7-H), 1.73–1.50 (m, 5H, 5-H_2_, 6-CH_2_, 7-H), 1.45 (s, 9H), 1.34 (dd, *J* = 7.1, 7.1 Hz, 3H, 1-POCH_2_CH_3_), 1.33 (dd, *J* = 7.0, 7.0 Hz, 3H, 1-POCH_2_CH_3_). ^13^C NMR (126 MHz, CDCl_3_) *δ* 155.7 (2-N–C_q_(O)–O), 80.0 (2-NC_q_(O)O–C_q_–(CH_3_)_3_), 62.5 (d, ^2^*J*_*CP*_ = 7.2 Hz, 1-POCH_2_), 62.3 (d, ^2^*J*_*CP*_ = 6.9 Hz, 1-POCH_2_), 52.4 (d, ^2^*J*_*CP*_ = 10.5 Hz, 2-H), 47.4 (d, ^1^*J*_*CP*_ = 149.5 Hz, 1-H), 38.8 (4-CH), 28.6 (2-NC_q_(O)OC_q_–(CH_3_)_3_), 26.9 (7-CH_2_), 25.2 (5-CH_2_), 19.4 (6-CH_2_), 16.7 (d, ^3^*J*_*CP*_ = 5.5 Hz, 1-P(OCH_2_CH_3_)_2_). ^31^P NMR (162 MHz, CDCl_3_) *δ* (27.83), 27.62.

### General procedure A for 4a–l

The carboxylic acid (2.0 equiv.) and HATU (2.1 equiv.) were dissolved in ethyl acetate. DIPEA (4.0 equiv.) was added dropwise and the reaction mixture was stirred at r.t. for at least 10 min. The reaction mixture was added to a solution of 3 in EtOAc. The reaction was stirred at r.t. for 12–24 h. The reaction mixture was then diluted with EtOAc and washed (3×) with a sat. solution of NaHCO_3_ (or alternatively, with a mixture of NaHCO_3_ and brine). The aqueous layer was re-extracted with EtOAc (2×) and the combined organic layers were washed with brine and dried over Na_2_SO_4_. The solvent was removed under reduced pressure. The crude product was purified using preparative HPLC (C8 column, MeCN:H_2_O). The product was obtained as separated diastereomers (in a total yield of 60–80%).

*tert*-Butyl 2-((diethoxyphosphoryl)(2-(4-methyl-2-thioxo-2,3-dihydrothiazol-5-yl)acetamido)methyl)piperidine-1-carboxylate (4a). The synthesis was performed according to general procedure A, 3 (316 mg, 0.902 mmol) and 2-mercapto-4-methyl-5-thiazoleacetic acid (341 mg, 1.80 mmol, 2.0 equiv.) gave the total product (330 mg, 0.632 mmol, 70%). 4a-1 was obtained as a light orange solid (152 mg, 0.291 mmol, 32%). 4a-2 was obtained as a light orange solid (178 mg, 0.341 mmol, 38%).

### Compound 4a-1, diastereomer 1

^1^H NMR (500 MHz, DMSO-*d*_6_) *δ* 12.93 (s, 1H, 13-NH), 8.67 (d, *J* = 9.9 Hz, 1H, 1-NH), 4.71 (ddd, ^2^*J*_*HP*_ = 15.7 Hz, *J* = 10.2, 10.2 Hz, 1H, 1-H), 4.42 (m, 1H, 2-H), 4.09–3.69 (m, 5H, 4-H, 1-P(OCH_2_)_2_), 3.46 (s, 2H, 10-H_2_), 2.74 (m, 1H, 4-H), 2.06 (s, 3H, 15-C_q_CH_3_), 1.61 (br. d, *J* = 13.4 Hz, 1H), 1.53–1.43 (m, 3H, 5-H, 6-H_2_), 1.40 (s, 9H, 2-NC_q_(O)OC_q_–(CH_3_)_3_), 1.36–1.19 (m, 2H, 5-H, 7-H), 1.17 (dd, *J* = 7.1 Hz, 3H, 1-POCH_2_CH_3_), 1.15 (dd, *J* = 7.1 Hz, 3H, 1-POCH_2_CH_3_). ^13^C NMR (126 MHz, DMSO-*d*_6_) *δ* 186.9 (13-CH), 168.2 (d, ^3^*J*_*CP*_ = 4.8 Hz), 153.5 (2-N–C_q_(O)–O), 135.0 (15-C_q_), 117.0 (11-C_q_), 78.7 (2-NC_q_(O)O–C_q_–(CH_3_)_3_), 62.1 (br. s, 1-POCH_2_), 61.4 (br. s, 1-POCH_2_), 50.2 (br. s, 2-CH), 43.6 (d, ^1^*J*_*CP*_ = 156.7 Hz, 1-CH), 38.5 (br. s, 4-CH), 32.3 (10-CH_2_), 28.1 (2-NC_q_(O)OC_q_–(CH_3_)_3_), 24.7 (br. s, 7-CH_2_), 24.5 (br. s, 5-CH_2_), 18.8 (6-CH_2_), 16.3 (d, ^3^*J*_*CP*_ = 5.5 Hz, 1-POCH_2_CH_3_), 16.1 (d, ^3^*J*_*CP*_ = 6.0 Hz, 1-POCH_2_CH_3_), 11.3 (15-C_q_CH_3_). ^31^P NMR (162 MHz, DMSO-*d*_6_) *δ* 22.48. HRESIMS *m*/*z* 522.2179 [M + H]^+^ (calcd 522.1856 for C_21_H_37_N_3_O_6_PS_2_).

### Compound 4a-2, diastereomer 2 (rotamer ratio 2.3 : 1)

^1^H NMR (500 MHz, DMSO-*d*_6_) *δ* 12.89 (s, 1H, 13-NH), 8.45 (d, *J* = 10.2 Hz, 1H, 1-NH), 4.71 (ddd, ^2^*J*_*HP*_ = 17.4 Hz, *J* = 10.9, 10.9 Hz, 1H, 1-H), 4.46 (m, 1H, 2-H), 4.10–3.95 (m, 4H, 1-P(OCH_2_)_2_), 3.74–3.65 (m, 1H, 4-CH), 3.47 (d, ^2^*J* = 15.9 Hz, 1H, part A of AB, 10-H), 3.33 (d, ^2^*J* = 15.9 Hz, part B of AB, 10-H), 2.71 (dd, *J* = 13.7, 13.7 Hz, 1H, 4-CH), 2.05 (m, 1H, 7-CH), 2.03 (s, 3H, 15-C_q_CH_3_), 1.58–1.47 (m, 4H, 5-CH, 6-CH_2_, 7-CH), 1.36 (s, 9H, 2-NC_q_(O)OC_q_–(CH_3_)_3_), 1.24 (m, 1H, 5-CH), 1.19 (t, *J* = 7.1 Hz, 6H, 1-(POCH_2_CH_3_)_2_). ^13^C NMR (101 MHz, DMSO-*d*_6_) *δ* 187.4 (13-C_q_), 168.8 (d, ^3^*J*_*CP*_ = 4.9 Hz, 9-C_q_), 154.6 (2-N–C_q_(O)–O), 135.1 (15-C_q_), 117.5 (11-C_q_), 79.1 (2-NC_q_(O)O–C_q_–(CH_3_)_3_), 62.9 (d, ^2^*J*_*CP*_ = 7.0 Hz, 1-POCH_2_), 62.1 (d, ^2^*J*_*CP*_ = 6.7 Hz, 1-POCH_2_), 50.1 (d, ^2^*J*_*CP*_ = 13.6 Hz, 2-CH), 44.1 (d, ^1^*J*_*CP*_ = 151.3 Hz, 1-CH), 39.8 (4-CH_2_), 32.5 (10-CH_2_), 28.5 (2-NC_q_(O)OC_q_–(CH_3_)_3_), 26.4 (7-CH_2_), 25.3 (5-CH_2_), 19.2 (6-CH_2_), 16.7 (d, ^3^*J*_*CP*_ = 5.5 Hz, 1-POCH_2_CH_3_), 16.5 (d, ^3^*J*_*CP*_ = 6.0 Hz, 1-POCH_2_CH_3_), 11.7 (15-C_q_CH_3_). ^31^P NMR (162 MHz, DMSO-*d*_6_) *δ* 22.92 (22.71). HRESIMS *m*/*z* 522.2255 [M + H]^+^ (calcd for C_21_H_37_N_3_O_6_PS_2_ 522.18559). The solution tautomer was confirmed by ^1^H–^15^N HSQC, and by X-ray diffraction.

### *tert*-Butyl 2-((diethoxyphosphoryl)(thiophene-2-carbox-amido)methyl)piperidine-1-carboxylate (4b)

The synthesis was performed according to general procedure A, 3 (211 mg, 0.603 mmol) and thiophene-2-carboxylic acid (154 mg, 1.19 mmol, 2.0 equiv.) gave the total product (212 mg, 0.460 mmol, 76%). 4b-1 was obtained as a colorless solid (100 mg, 0.217 mmol, 36%). 4b-2 was obtained as a colorless solid (112 mg, 0.243 mmol, 40%).

### Compound 4b-1, diastereomer 1

^1^H NMR (500 MHz, CD_3_CN) *δ* 7.71 (d, *J* = 3.8 Hz, 1H, 14-H), 7.64 (d, *J* = 5.0 Hz, 1H, 12-H), 7.35 (br. m, 1H, 1-NH), 7.12 (dd, *J* = 5.0, 3.8 Hz, 1H, 13-H), 5.04 (ddd, ^2^*J*_*HP*_ = 15.7 Hz, *J* = 10.5, 10.5 Hz, 1H), 4.74 (m, 1H, 2-H), 4.12–3.89 (m, 5H, 1-P(OCH_2_)_2_, 4-H), 2.94 (m, 1H, 4-H), 1.74 (br. d, *J* = 11.5 Hz, 1H, 7-H), 1.69–1.49 (m, 4H, 5-H, 6-H_2_, 7-H), 1.44 (s, 9H, 2-NC_q_(O)OC_q_–(CH_3_)_3_), 1.34 (m, 1H, 5-H), 1.24 (t, *J* = 7.1 Hz, 3H, 1-POCH_2_CH_3_), 1.19 (t, *J* = 7.1 Hz, 3H, 1-POCH_2_CH_3_). ^13^C NMR (101 MHz, CD_3_CN) *δ* 162.3 (d, *J* = 4.8 Hz, 9-C_q_), 155.2 (2-N–C_q_(O)–O), 139.9 (10-C_q_), 132.1 (12-CH), 129.5 (14-CH), 129.0 (13-CH), 79.9 (2-NC_q_(O)O–C_q_–(CH_3_)_3_), 63.5 (d, ^2^*J*_*CP*_ = 8.1 Hz, 1-POCH_2_), 63.0 (d, ^2^*J*_*CP*_ = 6.7 Hz, 1-POCH_2_), 51.1 (2-CH), 45.4 (d, ^1^*J*_*CP*_ = 157.2 Hz, 1-CH), 41.4 (4-CH_2_), 28.6 (2-NC_q_(O)OC_q_–(CH_3_)_3_), 26.0 (d, ^3^*J*_*CP*_ = 10.0 Hz, 7-CH_2_), 25.8 (5-CH_2_), 20.1 (6-CH_2_), 16.8 (d, ^3^*J*_*CP*_ = 5.7 Hz, 1-POCH_2_CH_3_), 16.7 (d, ^3^*J*_*CP*_ = 6.1 Hz, 1-POCH_2_CH_3_). ^31^P NMR (162 MHz, CDC_3_N) *δ* (21.77), 21.67. HRESIMS *m*/*z* 461.1854 [M + H]^+^ (calcd for C_20_H_34_N_2_O_6_PS 461.18697).

### Compound 4b-2, diastereomer 2 (rotamer ratio 2.3 : 1)

^1^H NMR (500 MHz, CD_3_CN) *δ* 7.61 (d, *J* = 4.8 Hz, 1H, 14-H), 7.55 (d, *J* = 3.8 Hz, 1H, 12-H), 7.08 (dd, *J* = 4.8, 3.8 Hz, 1H, 13-), 6.88 (d, *J* = 9.6 Hz, 1H, 1-NH), 4.95 (ddd, ^2^*J*_*HP*_ = 16.0 Hz, *J* = 10.7, 10.7 Hz, 1H, 1-H), 4.60 (m, 1H, 2-H), 4.18–4.02 (m, 4H, 1-P(OCH_2_)_2_), 3.84 (dd, *J* = 13.5, 3.4 Hz, 1H, 4-H), 2.77 (ddd, *J* = 13.2, 13.2, 1.9 Hz, 1H, 4-H), 2.26 (br. d, *J* = 11.9 Hz, 1H, 7-H), 1.68–1.58 (m, 4H, 5-H, 6-H_2_, 7-H), 1.34 (m, 1H, 5-H), 1.25 (t, *J* = 7.0 Hz, 3H, 1-POCH_2_CH_3_), 1.25 (t, *J* = 7.1 Hz, 3H, 1-POCH_2_CH_3_), 1.20 (s, 9H, 2-NC_q_(O)OC_q_–(CH_3_)_3_). ^13^C NMR (101 MHz, CD_3_CN) *δ* 162.0 (9-C_q_), 157.0 (2-N–C_q_(O)–O), 139.4 (10-C_q_), 132.1 (12-CH), 129.5 (14-CH), 128.9 (13-CH), 80.5 (2-NC_q_(O)O–C_q_–(CH_3_)_3_), 63.8 (d, ^2^*J*_*CP*_ = 7.0 Hz, 1-POCH_2_), 63.3 (d, ^2^*J*_*CP*_ = 6.5 Hz, 1-POCH_2_), 51.0 (d, ^3^*J*_*CP*_ = 12.4 Hz, 2-CH), 46.5 (d, ^1^*J*_*CP*_ = 153.3 Hz, 1-CH), 40.8 (4-CH_2_), 28.3 (2-NC_q_(O)OC_q_–(CH_3_)_3_), 26.9 (7-CH_2_), 25.87 (5-CH_2_), 19.8 (6-CH_2_), 16.8 (d, ^3^*J*_*CP*_ = 5.7 Hz, 1-POCH_2_CH_3_), 16.7 (d, ^3^*J*_*CP*_ = 6.0 Hz, 1-POCH_2_CH_3_). ^31^P NMR (162 MHz, CD_3_CN) *δ* (21.78), 21.74. HRESIMS *m*/*z* 461.2128 [M + H]^+^ (461.18697 calcd for C_20_H_34_N_2_O_6_PS).

### *tert*-Butyl 2-((diethoxyphosphoryl)(thiophene-3-carbox-amido)methyl)piperidine-1-carboxylate (4c)

The synthesis was performed according to general procedure A, 3 (211 mg, 0.601 mmol) and thiophene-3-carboxylic acid (153 mg, 1.20 mmol, 2.0 equiv.) gave the total product (219 mg, 0.476 mmol, 79%). 4c-1 was obtained as a colorless solid (101 mg, 0.219 mmol, 37%); 4c-2 was obtained as a colorless solid (118 mg, 0.256 mmol, 43%).

### Compound 4c-1, diastereomer 1

^1^H NMR (400 MHz, CDCl_3_) *δ* 7.98 (d, *J* = 2.2 Hz, 1H, 11-H), 7.46 (dd, *J* = 5.1, 1.3 Hz, 1H, 13-H), 7.34 (dd, *J* = 5.1, 3.0 Hz, 1H, 14-H), 6.96 (m, 1H, 1-NH), 5.08 (m, 1H, 1-H), 4.60 (m, 1H, 2-H), 4.17–4.04 (m, 5H, 1-P(OCH_2_)_2_, 4-H), 3.01 (m, 1H, 4-H), 1.85–1.68 (m, 2H, 6-H, 7-H), 1.63–1.53 (m, 3H, 5-H, 6-H, 7-H), 1.48 (s, 9H, 2-NC_q_(O)OC_q_–(CH_3_)_3_), 1.40 (m, 1H, 5-H), 1.29 (t, *J* = 7.1 Hz, 3H, 1-POCH_2_CH_3_), 1.23 (t, *J* = 7.1 Hz, 3H, 1-POCH_2_CH_3_). ^13^C NMR (101 MHz, CDCl_3_) *δ* 162.4 (d, ^3^*J*_*CP*_ = 4.8 Hz, 9-C_q_), 154.8 (2-N–C_q_(O)–O), 136.8 (10-C_q_), 129.1 (11-CH), 126.7 (13-CH), 126.4 (14-CH), 79.9 (2-NC_q_(O)O–C_q_–(CH_3_)_3_), 63.1 (d, ^2^*J*_*CP*_ = 5.5 Hz, 1-POCH_2_), 62.5 (d, ^2^*J*_*CP*_ = 6.9 Hz, 1-POCH_2_), 51.4 (2-CH), 45.1 (d, ^1^*J*_*CP*_ = 156.7 Hz, 1-CH), 40.4 (4-CH_2_), 28.6 (2-NC_q_(O)OC_q_–(CH_3_)_3_), 25.4 (d, ^3^*J*_*CP*_ = 9.7 Hz, 7-CH_2_), 24.8 (5-CH_2_), 19.5 (6-CH_2_), 16.6 (d, ^3^*J*_*CP*_ = 5.6 Hz, 1-POCH_2_CH_3_), 16.5 (d, ^3^*J*_*CP*_ = 6.2 Hz, 1-POCH_2_CH_3_). ^31^P NMR (162 MHz, CDCl_3_) *δ* (23.06), 22.83. HRESIMS *m*/*z* 461.1781 [M + H]^+^ (461.1870 calcd for C_20_H_34_N_2_O_6_PS).

### Compound 4c-2, diastereomer 2 (rotamer ratio 6.6 : 1)

^1^H NMR (400 MHz, CDCl_3_) *δ* 7.89 (d, *J* = 2.8 Hz, 1H, 11-H), 7.40 (m, 1H, 13-H), 7.28 (dd, *J* = 4.2, 3.0 Hz, 1H, 14-H), 6.72 (d, *J* = 9.6 Hz, 1H, 1-NH), 5.03 (ddd, ^2^*J*_*HP*_ = 15.4, *J* = 10.7, 10.7 Hz, 1H, 1-H), 4.70 (m, 1H, 2-H), 4.19–4.09 (m, 4H, 1-P(OCH_2_)_2_), 3.85 (m, 1H, 4-H), 2.80 (ddd, *J* = 13.4, 13.4, 2.9 Hz, 1H, 4-H), 2.44–2.30 (m, 1H, 7-H), 1.73–1.55 (m, 4H, 5-H, 6-H_2_, 7-H), 1.37 (m, 1H, 5-H), 1.30 (t, *J* = 6.9 Hz, 1H, 1-POCH_2_CH_3_), 1.28 (t, *J* = 6.8 Hz, 1H, 1-POCH_2_CH_3_), 1.23 (s, 9H, 2-NC_q_(O)OC_q_–(CH_3_)_3_). ^13^C NMR (101 MHz, CDCl_3_) *δ* 162.3 (d, ^3^*J*_*CP*_ = 3.8 Hz, 9-C_q_), 156.7 (2-N–C_q_(O)–O), 136.8 (10-C_q_), 129.0 (11-CH), 126.5 (13-CH), 126.4 (14-CH), 80.1 (2-NC_q_(O)O–C_q_–(CH_3_)_3_), 62.9 (d, ^2^*J*_*CP*_ = 5.6 Hz, 1-POCH_2_), 62.9 (d, *J* = 5.5 Hz, 1-POCH_2_), 50.1 (d, ^2^*J*_*CP*_ = 12.0 Hz, 2-CH), 45.6 (d, ^1^*J*_*CP*_ = 152.8 Hz, 1-CH), 40.2 (4-CH_2_), 28.3 (2-NC_q_(O)OC_q_–(CH_3_)_3_), 26.3 (7-CH_2_), 25.2 (5-CH_2_), 19.2 (6-CH_2_), 16.6 (d, ^3^*J*_*CP*_ = 5.4 Hz, 1-POCH_2_CH_3_), 16.4 (d, ^3^*J*_*CP*_ = 6.2 Hz, 1-POCH_2_CH_3_). ^31^P NMR (162 MHz, CDCl_3_) *δ* (23.43), 23.10. HRESIMS *m*/*z* 461.1836 [M + H]^+^ (461.1870 calcd for C_20_H_34_N_2_O_6_PS).

### *tert*-Butyl 2-((diethoxyphosphoryl)(4,5,6,7-tetrahydro-benzo-[*b*]thiophene-2-carboxamido)methyl)piperidine-1-carboxylate (4d)

The synthesis was performed according to general procedure A, 3 (241 mg, 0.689 mmol) and 4,5,6,7-tetrahydrobenzo[*b*]thiophene-2-carboxylic acid (251 mg, 1.38 mmol, 2.0 equiv.) gave the total product (269 mg, 0.523 mmol, 76%). 4d-1 was obtained as a colorless solid (127 mg, 0.247 mmol, 36%). 4d-2 was obtained as a colorless solid (142 mg, 0.256 mmol, 43%).

### Compound 4d-1, diastereomer 1

^1^H NMR (400 MHz, CDCl_3_) *δ* 7.26 (s, 1H, 18-H), 6.23 (br. s, 1H, 1-NH), 5.06 (ddd, ^2^*J*_*HP*_ = 16.7 Hz, *J* = 9.9, 9.9 Hz, 1H, 1-H), 4.57 (br. s, 1H, 2-H), 4.19–3.98 (m, 5H, 1-P(OCH_2_)_2_, 4-H), 2.94 (br. s, 1H, 4-H), 2.78 (dd, *J* = 6.1, 6.1 Hz, 1H, 16-H_2_), 2.60 (dd, *J* = 6.1, 6.1 Hz, 1H, 13-H_2_), 1.91–1.66 (m, 8H, 6-H, 7-H, 14-H_2_, 15-H_2_), 1.64–1.53 (m, 3H, 5-H, 6-H, 7-H), 1.49 (s, 9H, 2-NC_q_(O)OC_q_–(CH_3_)_3_), 1.40 (m, 1H, 5-H), 1.29 (t, ^3^*J*_*HP*_ = 6.9 Hz, 3H, 1-POCH_2_CH_3_), 1.26 (t, ^3^*J*_*HP*_ = 7.2 Hz, 3H, 1-POCH_2_CH_3_). ^13^C NMR (126 MHz, CDCl_3_) *δ* 161.6 (d, ^3^*J*_*CP*_ = 4.8 Hz, 9-C_q_), 154.7 (2-N–C_q_(O)–O), 142.4 (17-C_q_), 136.6 (12-C_q_), 133.5 (10-C_q_), 129.8 (18-CH), 79.9 (2-NC_q_(O)O–C_q_–(CH_3_)_3_), 63.1 (br. s, 1-POCH_2_), 62.5 (d, ^2^*J*_*CP*_ = 6.9 Hz, 1-POCH_2_), 51.2 (2-CH), 45.0 (d, ^1^*J*_*CP*_ = 159.9 Hz, 1-CH), 40.7 (4-CH_2_), 28.6 (2-NC_q_(O)OC_q_–(CH_3_)_3_), 25.6 (13-CH_2_), 25.5 (16-CH_2_), 25.4 (d, ^3^*J*_*CP*_ = 8.5 Hz, 7-CH_2_), 24.9 (5-CH_2_), 23.4 (15-CH_2_), 22.7 (14-CH_2_), 19.5 (6-CH_2_), 16.6 (d, ^3^*J*_*CP*_ = 5.5 Hz, 1-POCH_2_CH_3_), 16.4 (d, ^3^*J*_*CP*_ = 6.3 Hz, 1-POCH_2_CH_3_). ^31^P NMR (162 MHz, CDCl_3_) *δ* 22.72. HRESIMS *m*/*z* 515.2324 [M + H]^+^ (515.2339 calcd for C_22_H_38_N_2_O_5_P).

### Compound 4d-2, diastereomer 2 (rotamer ratio 4.9 : 1)

^1^H NMR (400 MHz, CDCl_3_) *δ* 7.12 (s, 1H, 18-H), 6.55 (d, *J* = 9.6 Hz, 1H, 1-NH), 4.99 (ddd, ^2^*J*_*HP*_ = 15.0 Hz, *J* = 11.6, 9.6 Hz, 1H, 1-H), 4.68 (m, 1H, 2-H), 4.19–4.10 (m, 4H, 1-P(OCH_2_)_2_), 3.84 (m, 1H, 4-H), 2.78 (ddd, *J* = 13.3, 13.3, 2.9 Hz, 1H, 4-H), 2.76–2.72 (m, 2H, 13-H_2_ or 16-H_2_), 2.64–2.44 (m, 2H, 16-H_2_ or 13-H_2_), 2.37 (br. d, *J* = 11.5 Hz, 1H, 7-H), 1.87–1.72 (m, 4H, 14-H_2_, 15-H_2_), 1.69–1.56 (m, 4H, 5-H, 6-H_2_, 7-H), 1.40 (m, 1H, 5-H), 1.31 (t, ^3^*J*_*CP*_ = 7.1 Hz, 3H, 1-POCH_2_CH_3_), 1.28 (s, 9H, 2-NC_q_(O)OC_q_–(CH_3_)_3_), 1.28 (t, *J* = 7.3 Hz, 3H, 1-POCH_2_CH_3_). ^13^C NMR (101 MHz, CDCl_3_) *δ* 161.7 (d, *J* = 3.8 Hz, 9-C_q_), 156.7 (2-N–C_q_(O)–O), 142.1 (17-C_q_), 136.2 (12-C_q_), 133.9 (10-C_q_), 129.1 (18-CH), 80.1 (2-NC_q_(O)O–C_q_–(CH_3_)_3_), 62.9 (d, *J* = 5.5 Hz, 1-POCH_2_), 62.8 (d, ^2^*J*_*CP*_ = 6.8 Hz, 1-POCH_2_), 50.0 (d, ^2^*J*_*CP*_ = 12.2 Hz, 2-CH), 45.7 (d, ^1^*J*_*CP*_ = 152.7 Hz, 1-CH), 40.2 (4-CH_2_), 28.3 (2-NC_q_(O)OC_q_–(CH_3_)_3_), 26.3 (7-CH_2_), 25.5 (13-CH_2_ or 16-CH_2_), 25.4 (13-CH_2_ or 16-CH_2_), 25.2 (5-CH_2_), 23.4 (15-CH_2_), 22.8 (14-CH_2_), 19.2(6-CH_2_), 16.7 (d, ^3^*J*_*CP*_ = 5.3 Hz, 1-POCH_2_CH_3_), 16.5 (d, ^3^*J*_*CP*_ = 6.3 Hz, 1-POCH_2_CH_3_). ^31^P NMR (162 MHz, CDCl_3_) *δ* (23.42), 23.05. HRESIMS *m*/*z* 515.2316 [M + H]^+^ (515.2339 calcd for C_22_H_38_N_2_O_5_P).

### *tert*-Butyl 2-((diethoxyphosphoryl)(4,5,6,7-tetrahydro-benzo-[*b*]thiophene-3-carboxamido)methyl)piperidine-1-carboxylate (4e)

Synthesis was performed according to the general procedure A, 3 (322 mg, 0.919 mmol) and 4,5,6,7-tetrahydrobenzo[*b*]thiophene-3-carboxylic acid (336 mg, 1.84 mmol) gave the total product (317 mg, 0.616 mmol, 67%). 4e-1 was obtained as a colorless solid (117 mg, 0.227 mmol, 25%). 4e-2 was obtained as a colorless solid (201 mg, 0.391 mmol, 43%).

### Compound 4e-1, diastereomer 1

^1^H NMR (400 MHz, CDCl_3_) *δ* 7.50 (s, 1H, 11-H), 6.35 (br. s, 1H, 1-NH), 5.07 (ddd, ^2^*J*_*HP*_ = 16.8 Hz, *J* = 9.9, 9.9 Hz, 1H, 1-H), 4.55 (m, 1H, 2-H), 4.18–4.03 (m, 4H, 1-P(OCH_2_)_2_), 3.97 (m, 1H, 4-H), 3.00 (m, 1H, 4-H), 2.88–2.70 (m, 4H, 14-CH_2_, 17-CH_2_), 1.90–1.72 (m, 6H, 6-H, 7-H, 15-CH_2_, 16-CH_2_), 1.65–1.53 (m, 4H, 5-H, 6-H, 7-H), 1.48 (s, 9H, 2-NC_q_(O)OC_q_–(CH_3_)_3_), 1.40 (m, 1H, 5-H), 1.29 (m, 6H, 1-P(OCH_2_CH_3_)_2_). ^13^C NMR (126 MHz, CDCl_3_) *δ* 163.9 (d, *J* = 4.7 Hz, 9-C_q_), 154.8 (2-N–C_q_(O)–O), 138.3 (13-C_q_ or 18-C_q_), 135.6 (10-C_q_), 134.7 (13-C_q_ or 18-C_q_), 124.8 (11-CH), 79.8 (2-NC_q_(O)O–C_q_–(CH_3_)_3_), 62.8 (br. s, 1-POCH_2_), 62.5 (d, ^2^*J*_*CP*_ = 6.6 Hz, 1-POCH_2_), 51.2 (2-CH), 44.4 (d, ^1^*J*_*CP*_ = 156.2 Hz, 1-CH), 40.6 (4-CH_2_), 28.5 (2-NC_q_(O)OC_q_–(CH_3_)_3_), 25.7 (14-CH_2_ or 17-CH_2_), 25.5 (14-CH_2_ or 17-CH_2_), 25.5 (d, ^3^*J*_*CP*_ = 8.1 Hz, 7-CH_2_), 24.9 (5-CH_2_), 23.2 (15-CH_2_ or 16-CH_2_), 22.7 (15-CH_2_ or 16-CH_2_), 19.5 (6-CH_2_), 16.6 (d, ^3^*J*_*CP*_ = 5.7 Hz, 1-POCH_2_CH_3_), 16.5 (d, ^3^*J*_*CP*_ = 6.1 Hz, 1-POCH_2_CH_3_). ^31^P NMR (162 MHz, CDCl_3_) *δ* (22.91), 22.54. HRESIMS *m*/*z* 515.2349 [M + H]^+^ (515.2339 calcd for C_22_H_38_N_2_O_5_P).

### Compound 4e-2, diastereomer 2 (rotamer ratio 3.6 : 1)

^1^H NMR (400 MHz, CDCl_3_) *δ* 7.48 (s, 1H, 11-H), 6.39 (d, *J* = 9.9 Hz, 1H, 1-NH), 5.02 (ddd, ^2^*J*_*HP*_ = 15.4 Hz, *J* = 11.6, 9.9 Hz, 1H, 1-H), 4.67 (m, 1H, 2-H), 4.27–4.08 (m, 4H, 1-P(OCH_2_)_2_), 3.85 (br. dd, *J* = 13.52, 3.7 Hz, 1H, 4-H), 2.88–2.59 (m, 5H, 4-H, 14-H_2_, 17-H_2_), 2.39 (br. d, *J* = 10.1 Hz 1H, 7-H), 1.92–1.72 (m, 4H, 15-H_2_, 16-H_2_), 1.70–1.56 (m, 4H, 5-H, 6-H_2_, 7-H), 1.40 (m, 1H, 5-H), 1.34 (t, *J* = 7.2 Hz, 3H, 1-POCH_2_CH_3_), 1.29 (t, *J* = 7.1 Hz, 3H, 1-POCH_2_CH_3_), 1.24 (s, 9H, 2-NC_q_(O)OC_q_–(CH_3_)_3_). ^13^C NMR (101 MHz, CDCl_3_) *δ* 163.7 (d, *J* = 4.0 Hz, 9-C_q_), 156.4 (2-N–C_q_(O)–O), 137.8 (13-C_q_ or 18-C_q_), 135.3 (10-C_q_), 134.3 (13-C_q_ or 18-C_q_), 124.9 (11-CH), 79.8 (2-NC_q_(O)O–C_q_–(CH_3_)_3_), 62.7 (d, ^2^*J*_*CP*_ = 6.0 Hz, 1-POCH_2_), 62.5 (d, ^2^*J*_*CP*_ = 7.2 Hz, 1-POCH_2_), 49.8 (d, ^2^*J*_*CP*_ = 12.4 Hz, 2-H), 44.8 (d, ^1^*J*_*CP*_ = 152.2 Hz, 1-H), 40.0 (4-CH_2_), 28.1 (2-NC_q_(O)OC_q_–(CH_3_)_3_), 26.1 (7-CH_2_), 25.5 (14-CH_2_ or 17-CH_2_), 25.3 (14-CH_2_ or 17-CH_2_), 25.1 (5-CH_2_), 23.0 (15-CH_2_ or 16-CH_2_), 22.6 (15-CH_2_ or 16-CH_2_), 19.00 (6-CH_2_), 16.5 (d, ^3^*J*_*CP*_ = 5.4 Hz, 1-POCH_2_CH_3_), 16.3 (d, ^3^*J*_*CP*_ = 6.2 Hz, 1-POCH_2_CH_3_). ^31^P NMR (162 MHz, CDCl_3_) *δ* (23.57), 23.27. HRESIMS *m*/*z* 515.2302 [M + H]^+^ (515.2339 calcd for C_22_H_38_N_2_O_5_P).

### *tert*-Butyl 2-((diethoxyphosphoryl)(4,5,6,7-tetrahydro-benzo[*c*]thiophene-1-carboxamido)methyl)piperidine-1-carboxylate (4f)

The synthesis was performed according to general procedure A, 3 (303 mg, 0.865 mg) and 4,5,6,7-tetrahydrobenzo[*c*]thiophene-1-carboxylic acid (336 mg, 1.84 mg, 2.1 equiv.) gave the total product (275 mg, 0.534 mmol, 62%). 4f-1 was obtained as a colorless solid (121 mg, 0.235 mmol, 27%). 4f-2 was obtained as a colorless solid (201 mg, 0.391 mmol, 45%).

### Compound 4f-1, diastereomer 1

^1^H NMR (400 MHz, CDCl_3_) *δ* 6.99 (s, 1H, 12-H), 6.18 (s, 1H, 1-NH), 5.08 (ddd, ^2^*J*_*HP*_ = 16.4 Hz, *J* = 9.5, 9.5 Hz, 1H, 1-H), 4.55 (m, 1H, 2-H), 4.27–3.86 (m, 5H, 1-P(OCH_2_)_2_), 3.10–2.86 (m, 3H, 4-H, 17-H_2_), 2.71 (br. dd, *J* = 6.0, 6.0 Hz, 2H, 14-H_2_), 1.85–1.70 (m, 6H, 6-H, 7-H, 15-H_2_, 16-H_2_), 1.58 (m, 3H, 5-H, 6-H, 7-H), 1.48 (s, 9H, 2-NC_q_(O)OC_q_–(CH_3_)_3_), 1.40 (m, 1H, 5-H), 1.29 (t, *J* = 7.0 Hz, 6H, 1-POCH_2_CH_3_). ^13^C NMR (126 MHz, CDCl_3_) *δ* 162.4 (d, *J* = 4.6 Hz, 9-C_q_), 154.9 (2-N–C_q_(O)–O), 141.9 (13-C_q_), 140.7 (18-C_q_), 129.0 (10-C_q_), 122.7 (12-CH), 79.8 (2-NC_q_(O)O–C_q_–(CH_3_)_3_), 62.8 (br. s, 1-POCH_2_), 62.6 (d, *J* = 6.6 Hz, 1-POCH_2_), 51.3 (d, ^2^*J*_*CP*_ = 2.9 Hz, 2-CH), 44.8 (d, ^1^*J*_*CP*_ = 155.3 Hz, 1-CH), 40.6 (4-CH_2_), 28.6 (2-NC_q_(O)OC_q_–(CH_3_)_3_), 27.0 (17-CH_2_), 26.6 (14-CH_2_), 25.5 (d, ^3^*J*_*CP*_ = 9.5 Hz, 7-CH_2_), 24.8 (5-CH_2_), 23.1 (16-CH_2_), 22.9 (15-CH_2_), 19.5 (6-CH_2_), 16.6 (d, ^3^*J*_*CP*_ = 5.7 Hz, 1-POCH_2_CH_3_), 16.5 (d, ^3^*J*_*CP*_ = 6.0 Hz, 1-POCH_2_CH_3_). ^31^P NMR (162 MHz, CDCl_3_) *δ* 22.81, 22.42. HRESIMS *m*/*z* 515.2312 [M + H]^+^ (515.2339 calcd for C_22_H_38_N_2_O_5_P).

### Compound 4f-2, diastereomer 2 (rotamer ratio 3.5 : 1)

^1^H NMR (400 MHz, CDCl_3_) *δ* 6.96 (s, 1H, 12-H), 6.34 (d, *J* = 9.8 Hz, 1H, 1-NH), 5.06 (ddd, ^2^*J*_*HP*_ = 15.3 Hz, *J* = 11.6, 9.8 Hz, 1H, 1-H), 4.68 (m, 1H, 2-H), 4.25–4.09 (m, 4H, 1-P(OCH_2_)_2_), 3.85 (br. dd, *J* = 13.6, 2.6 Hz, 1H, 4-H), 3.08–2.91 (m, 2H, 14-CH_2_ or 17-CH_2_), 2.87 (ddd, *J* = 13.4, 13.4, 2.8 Hz, 1H, 4-H), 2.75–2.57 (m, 2H, 14-CH_2_ or 17-CH_2_), 2.41 (m, *J* = 9.0 Hz 1H, 7-H), 1.91–1.56 (m, 8H, 5-H, 6-H_2_, 7-H, 15-CH_2_, 16-CH_2_), 1.39 (m, 1H, 5-H), 1.34 (t, *J* = 7.1 Hz, 3H, 1-POCH_2_CH_3_), 1.28 (t, *J* = 7.1 Hz, 3H, 1-POCH_2_CH_3_), 1.20 (s, 9H, 2-NC_q_(O)OC_q_–(CH_3_)_3_). ^13^C NMR (101 MHz, CDCl_3_) *δ* 162.6 (d, *J* = 4.4 Hz, 9-C_q_), 156.6 (2-N–C_q_(O)–O), 140.8 (13-C_q_ or 18-C_q_), 140.2 (13-C_q_ or 18-C_q_), 130.9 (10-C_q_), 123.1 (12-CH), 79.9 (2-NC_q_(O)O–C_q_–(CH_3_)_3_), 62.9 (d, ^2^*J*_*CP*_ = 6.1 Hz, 1-POCH_2_), 62.7 (d, ^2^*J*_*CP*_ = 7.2 Hz, 1-POCH_2_), 49.9 (d, ^2^*J*_*CP*_ = 12.3 Hz, 2-CH), 45.3 (d, ^1^*J*_*CP*_ = 151.9 Hz, 1-CH), 40.2 (4-CH_2_), 28.1 (2-NC_q_(O)OC_q_–(CH_3_)_3_), 26.7 (14-CH_2_ or 17-CH_2_), 26.6 (14-CH_2_ or 17-CH_2_), 26.2 (7-CH_2_), 25.2 (5-CH_2_), 23.2 (15-CH_2_ or 16-CH_2_), 22.9 (15-CH_2_ or 16-CH_2_), 19.2 (6-CH_2_), 16.7 (d, ^3^*J*_*CP*_ = 5.5 Hz, 1-POCH_2_CH_3_), 16.5 (d, ^3^*J*_*CP*_ = 6.0 Hz, 1-POCH_2_CH_3_). ^31^P NMR (162 MHz, CDCl_3_) *δ* (23.42), 23.15. HRESIMS *m*/*z* 515.2329 [M + H]^+^ (515.2339 calcd for C_22_H_38_N_2_O_5_P).

### *tert*-Butyl 2-((benzo[*b*]thiophene-2-carboxamido)(di-ethoxyphosphoryl)methyl)piperidine-1-carboxylate (4g)

The synthesis was performed according to the general procedure A, 3 (236 mg, 0.673 mmol) and benzo[*b*]thiophene-2-carboxylic acid (238 mg, 1.34 mmol, 2.0 equiv.) gave the total product (270 mg, 0.529 mmol, 79%). 4g-1 was obtained as a colorless solid (110 mg, 0.215 mmol, 32%). 4g-2 was obtained as a colorless solid (153 mg, 0.300 mmol, 44%).

### Compound 4g-1, diastereomer 1

^1^H NMR (400 MHz, CDCl_3_) *δ* 7.90 (s, 1H, 18-H), 7.86 (dd, *J* = 8.1, 1.1 Hz, 1H, 13-H), 7.84 (dd, *J* = 7.7, 1.7 Hz, 1H, 16-H), 7.43 (ddd, *J* = 7.1, 7.1, 1.7 Hz, 1H, part C of ABCD, 14-CH), 7.40 (ddd, *J* = 7.1, 7.1, 1.6 Hz, 1H, part D of ABCD, 15-CH), 7.00 (s, 1H, 1-NH), 5.14 (m, 1H, 1-H), 4.65 (m, 1H, 2-H), 4.22–3.91 (m, 5H, 1-P(OCH_2_)_2_, 4-H), 2.92 (m, 1H, 4-H), 1.85 (m, 1H, 7-H), 1.75 (m, 1H, 6-H), 1.66–1.54 (m, 3H, 5-H, 6-H, 7-H), 1.49 (s, 9H, 2-NC_q_(O)OC_q_–(CH_3_)_3_), 1.42 (m, 1H, 5-H), 1.31 (t, *J* = 7.1 Hz, 3H, 1-POCH_2_CH_3_), 1.24 (t, *J* = 7.2 Hz, 3H, 1-POCH_2_CH_3_). ^13^C NMR (126 MHz, CDCl_3_) *δ* 160.8 (d, *J* = 5.0 Hz, 9-C_q_), 153.9 (2-N–C_q_(O)–O), 140.2 (12-C_q_), 138.1 (17-C_q_), 136.8 (10-C_q_), 125.5 (14-CH), 124.7 (18-CH), 124.2 (16-CH), 123.9 (15-CH), 121.7 (13-CH), 78.8 (2-NC_q_(O)O–C_q_–(CH_3_)_3_), 62.2 (br s, 1-POCH_2_), 61.5 (br s, 1-POCH_2_), 50.1 (2-CH), 44.7 (d, ^1^*J*_*CP*_ = 148.9 Hz, 1-CH), 39.4 (4-CH_2_), 27.4 (2-NC_q_(O)OC_q_–(CH_3_)_3_), 24.3 (d, ^3^*J*_*CP*_ = 9.8 Hz, 7-CH_2_), 23.7 (5-CH_2_), 18.3 (6-CH_2_), 15.5 (d, ^3^*J*_*CP*_ = 5.6 Hz, 1-POCH_2_CH_3_), 15.3 (d, ^3^*J*_*CP*_ = 6.3 Hz, 1-POCH_2_CH_3_). ^31^P NMR (162 MHz, CDCl_3_) *δ* 22.79, 22.43. HRESIMS *m*/*z* 511.2017 [M + H]^+^ (511.2026 calcd for C_24_H_36_N_2_O_6_PS).

### Compound 4g-2, diastereomer 2 (rotamer ratio 6.6 : 1)

^1^H NMR (400 MHz, CDCl_3_) *δ* 7.84 (d, *J* = 7.9, 1.2 Hz, 1H, 13-CH), 7.78 (dd, *J* = 7.1, 1.6 Hz, 1H, 16-H), 7.73 (s, 1H, 18-H), 7.41 (ddd, *J* = 7.4, 7.4, 1.4 Hz, 1H, part C of ABCD 14-CH), 7.37 (ddd, *J* = 7.4, 7.4, 1.4 Hz, 1H, part D of ABCD, 15-CH), 6.90 (d, *J* = 9.5 Hz, 1H, 1-NH), 5.05 (ddd, ^2^*J*_*HP*_ = 15.0 Hz, *J* = 11.6, 9.5 Hz, 1H, 1-H), 4.75 (m, 1H, 2-H), 4.32–4.14 (m, 4H, 1-P(OCH_2_)_2_), 3.89 (dd, *J* = 14.0, 4.0 Hz, 1H, 4-H), 2.82 (ddd, *J* = 13.3, 13.3, 2.7 Hz, 1H, 4-H), 2.38 (br. d, *J* = 12.4 Hz, 1H, 7-H), 1.80–1.58 (m, 4H, 5-H, 5-H_2_, 7-H), 1.41 (m, 1H, 5-H), 1.33 (t, *J* = 7.1 Hz, 3H, 1-POCH_2_CH_3_), 1.30 (t, *J* = 7.1 Hz, 3H, 1-POCH_2_CH_3_), 1.23 (s, 9H, 2-NC_q_(O)OC_q_–(CH_3_)_3_). ^13^C NMR (126 MHz, CDCl_3_) *δ* 161.8 (d, *J* = 3.6 Hz, 9-C_q_), 156.9 (2-N–C_q_(O)–O), 141.4 (12-C_q_), 139.2 (17-C_q_), 138.2 (10-C_q_), 126.5 (14-CH), 125.3 (18-CH), 125.1 (16-CH), 125.0 (15-CH), 122.8 (13-CH), 80.3 (2-NC_q_(O)O–C_q_–(CH_3_)_3_), 63.1 (d, ^2^*J*_*CP*_ = 6.3 Hz, 1-POCH_2_), 63.0 (d, ^2^*J*_*CP*_ = 7.5 Hz, 1-POCH_2_), 50.0 (d, ^2^*J*_*CP*_ = 11.7 Hz, 2-H), 46.2 (d, ^1^*J*_*CP*_ = 152.7 Hz, 1-H), 40.2 (4-CH_2_), 28.3 (2-NC_q_(O)OC_q_–(CH_3_)_3_), 26.3 (7-CH_2_), 25.2 (5-CH_2_), 19.2 (7-CH_2_), 16.7 (d, ^3^*J*_*CP*_ = 5.4 Hz, 1-POCH_2_CH_3_), 16.5 (d, ^3^*J*_*CP*_ = 6.3 Hz, 1-POCH_2_CH_3_). ^31^P NMR (162 MHz, CDCl_3_) *δ* (22.97), 22.53. HRESIMS *m*/*z* 511.2029 [M + H]^+^ (511.2026 calcd for C_24_H_36_N_2_O_6_PS).

### *tert*-Butyl 2-((benzo[*b*]thiophene-3-carboxamido)(di-ethoxyphosphoryl)methyl)piperidine-1-carboxylate (4h)

Synthesis was performed according to the general procedure A, 3 (241 mg, 0.689 mmol) and benzo[*b*]thiophene-3-carboxylic acid (245 mg, 1.38 mmol, 2.0 equiv.) gave the total product (257 mg, 0.503 mmol, 73%). 4h-1 was obtained as a colorless solid (104 mg, 0.204 mmol, 30%). 4h-2 was obtained as a colorless solid (153 mg, 0.300 mmol, 44%).

### Compound 4h-1, diastereomer 1

^1^H NMR (400 MHz, CDCl_3_) *δ* 8.43 (d, *J* = 7.8 Hz, 1H, 17-H), 8.07 (br. s, 1H, 11-H), 7.87 (d, *J* = 7.8 Hz, 1H, 14-H), 7.45 (ddd, *J* = 7.6, 7.6, 1.3 Hz, 1H, 16-CH), 7.40 (ddd, *J* = 7.6, 7.2, 1.4 Hz, 1H, 15-CH), 7.11 (m, 1H, 1-NH), 5.20 (ddd, ^2^*J*_*HP*_ = 17.7 Hz, *J* = 9.9, 9.9 Hz, 1H, 1-H), 4.66 (m, 1H, 2-H), 4.25–3.92 (m, 5H, 1-P(OCH_2_)_2_, 4-H), 3.05 (m, 1H, 4-H), 2.13–1.72 (m, 2H, 7-H, 6-H), 1.67–1.55 (m, 3H, 5-H, 6-H, 7-H), 1.48 (s, 9H, 2-NC_q_(O)OC_q_–(CH_3_)_3_), 1.40 (m, 1H, 5-H), 1.31 (t, *J* = 7.1 Hz, 3H, 1-POCH_2_CH_3_), 1.24 (t, *J* = 6.9 Hz, 3H, 1-POCH_2_CH_3_). ^13^C NMR (126 MHz, CDCl_3_) *δ* 163.4 (d, *J* = 4.7 Hz, 9-C_q_), 155.0 (2-N–C_q_(O)–O), 140.3 (13-C_q_), 137.1 (18-C_q_), 131.1 (10-C_q_), 130.6 (br. s, 11-C_q_), 125.4 (16-CH), 125.3 (15-CH), 124.5 (17-CH), 122.6 (14-CH), 79.9 (2-NC_q_(O)O–C_q_–(CH_3_)_3_), 63.1 (d, ^2^*J*_*CP*_ = 4.4 Hz, 1-POCH_2_), 62.6 (d, ^2^*J*_*CP*_ = 5.5 Hz, 1-POCH_2_), 51.2 (2-CH), 44.9 (d, ^1^*J*_*CP*_ = 155.7 Hz, 1-CH), 40.6 (4-CH_2_), 28.6 (2-NC_q_(O)OC_q_–(CH_3_)_3_), 25.5 (d, ^3^*J*_*CP*_ = 10.1 Hz, 7-CH_2_), 24.9 (5-CH_2_), 19.5 (6-CH_2_), 16.6 (d, ^3^*J*_*CP*_ = 5.6 Hz, 1-POCH_2_CH_3_), 16.5 (d, ^3^*J*_*CP*_ = 6.2 Hz, 1-POCH_2_CH_3_). ^31^P NMR (162 MHz, CDCl_3_) *δ* 22.71. HRESIMS *m*/*z* 511.2016 [M + H]^+^ (511.2026 calcd for C_24_H_36_N_2_O_6_PS).

### Compound 4h-2, diastereomer 2 (rotamer ratio 4.8 : 1)

^1^H NMR (400 MHz, CDCl_3_) *δ* 8.46 (d, *J* = 7.9 Hz, 1H, 17-H), 7.92 (s, 1H, 11-H), 8.46 (d, *J* = 7.9 Hz, 1H, 14-H), 7.45 (ddd, *J* = 8.2, 7.1, 1.2 Hz, 1H, 16-H), 7.39 (ddd, *J* = 8.0, 6.9, 1.2 Hz, 1H, 15-H), 6.66 (d, *J* = 9.9 Hz, 1H, 1-NH), 5.13 (ddd, ^2^*J*_*HP*_ = 15.4 Hz, *J* = 11.6, 9.8 Hz, 1H, 1-H), 4.74 (m, 1H, 2-H), 4.27–4.15 (m, 4H, 1-P(OCH_2_)_2_), 3.89 (ddd, *J* = 13.6, 4.7, 2.1 Hz, 1H, 4-H), 2.90 (ddd, *J* = 13.4, 13.4, 2.8 Hz, 1H, 4-H), 2.42 (m, 1H, 7-H), 1.77–1.58 (m, 4H, 5-H, 6-H_2_, 7-H), 1.43 (m, 1H, 5-H), 1.35 (t, *J* = 7.1 Hz, 3H, 1-POCH_2_CH_3_), 1.31 (t, *J* = 7.2 Hz, 3H, 1-POCH_2_CH_3_), 1.16 (s, 9H, 2-NC_q_(O)OC_q_–(CH_3_)_3_) ppm. ^31^P NMR (162 MHz, CDCl_3_) *δ* (23.40), 22.99 ppm. HRESIMS *m*/*z* 511.2008 [M + H]^+^ (511.2026 calcd for C_24_H_36_N_2_O_6_PS).

### *tert*-Butyl 2-((diethoxyphosphoryl)(2-(thiophen-2-yl)-acetamido)methyl)piperidine-1-carboxylate (4i)

The synthesis was performed according to general procedure A, 3 (255 mg, 0.897 mmol) and 2-(thiophen-2-yl)acetic acid (255 mg, 1.79 mmol, 2.0 equiv.) gave the total product (256 mg, 0.539 mmol, 60%). 4i-1 was obtained as a colorless solid (125 mg, 0.238 mmol, 27%). 4i-2 was obtained as a colorless solid (139 mg, 0.293 mmol, 33%).

### Compound 4i-1, diastereomer 1

^1^H NMR (400 MHz, CDCl_3_) *δ* 7.23 (dd, *J* = 4.7, 1.7 Hz, 1H, 13-CH), 6.98 (dd, *J* = 3.5, 3.5 Hz, 1H, 14-H), 6.97 (m, 1H, 15-H), 6.46 (m, 1H, 1-NH), 4.90 (ddd, ^2^*J*_*HP*_ = 16.0 Hz, *J* = 10.2, 10.2 Hz, 1H, 1-H), 4.41 (m, 1H, 2-H), 4.16–3.88 (m, 5H, 1-P(OCH_2_)_2_, 4-H), 3.83 (s, 2H, 10-H_2_), 2.85 (m, 1H, 4-H), 1.69–1.42 (m, 5H, 5-H, 6-H_2_, 7-H_2_), 1.44 (s, 9H, 2-NC_q_(O)OC_q_–(CH_3_)_3_), 1.37 (m, 1H, 5-H), 1.27 (t, *J* = 7.1 Hz, 3H, 1-POCH_2_CH_3_), 1.22 (t, *J* = 7.1 Hz, 3H, 1-POCH_2_CH_3_). ^13^C NMR (101 MHz, CDCl_3_) *δ* 169.5 (d, *J* = 3.7 Hz, 9-C_q_), 154.6 (2-N–C_q_(O)–O), 135.9 (11-C_q_), 127.4 (14-CH or 15-CH), 127.4 (14-CH or 15-CH), 125.6 (13-CH), 79.8 (2-NC_q_(O)O–C_q_–(CH_3_)_3_), 62.9 (d, ^2^*J*_*CP*_ = 7.2 Hz, 1-POCH_2_), 62.4 (d, ^2^*J*_*CP*_ = 6.5 Hz, 1-POCH_2_), 50.7 (2-CH), 44.8 (d, ^1^*J*_*CP*_ = 155.2 Hz, 1-CH), 40.6 (4-CH_2_), 37.5 (10-CH_2_), 28.5 (2-NC_q_(O)OC_q_–(CH_3_)_3_), 25.2 (d, ^3^*J*_*CP*_ = 9.8 Hz, 7-CH_2_), 24.9 (5-CH_2_), 19.4 (6-CH_2_), 16.5 (d, ^3^*J*_*CP*_ = 6.0 Hz, 1-POCH_2_CH_3_), 16.5 (d, ^3^*J*_*CP*_ = 6.5 Hz, 1-POCH_2_CH_3_). ^31^P NMR (162 MHz, CDCl_3_) *δ* 22.15. HRESIMS *m*/*z* 475.1967 [M + H]^+^ (475.2026 calcd for C_21_H_36_N_2_O_6_PS).

### Compound 4i-2, diastereomer 2 (rotamer ratio 3.2 : 1)

^1^H NMR (400 MHz, CDCl_3_) *δ* 7.21 (d, *J* = 5.0 Hz, 1H, 13-H), 6.96 (dd, *J* = 5.0, 3.5 Hz, 1H, 14-H), 6.93 (m, 1H, 15-H), 6.10 (m, 1H, 1-NH), 4.84 (ddd, ^2^*J*_*HP*_ = 15.4 Hz, *J* = 11.5, 10.1 Hz, 1H, 1-H), 4.55 (m, 1H, 2-H), 4.19–3.98 (m, 4H, 1-P(OCH_2_)_2_), 3.88 (m, 1H, 4-H), 3.69 (s, 2H, 10-H_2_), 2.76 (m, 1H, 4-H), 2.30 (m, 1H, 7-H), 1.70–1.53 (m, 4H, 5-H, 6-H_2_, 7-H), 1.45 (s, 9H, 2-NC_q_(O)OC_q_–(CH_3_)_3_), 1.35 (m, 1H, 5-H), 1.25 (t, *J* = 7.1 Hz, 6H, 1-P(OCH_2_CH_3_)_2_). ^13^C NMR (101 MHz, CDCl_3_) *δ* 169.5 (d, *J* = 3.2 Hz, 9-C_q_), 156.3 (2-N–C_q_(O)–O), 135.8 (11-C_q_), 127.2 (14-CH), 127.1 (15-CH), 125.4 (13-CH), 80.0 (2-NC_q_(O)O–C_q_–(CH_3_)_3_), 62.9 (br. s, 1-POCH_2_), 62.6 (br. s, 1-POCH_2_), 49.9 (2-CH), 45.5 (d, ^1^*J*_*CP*_ = 163.1 Hz, 1-CH), 40.1 (4-CH_2_), 37.7 (10-CH_2_), 28.6 (2-NC_q_(O)OC_q_–(CH_3_)_3_), 26.3 (7-CH_2_), 25.2 (5-CH_2_), 19.1 (6-CH_2_), 16.5 (d, ^3^*J*_*CP*_ = 5.6 Hz, 1-POCH_2_CH_3_), 16.4 (d, ^3^*J*_*CP*_ = 6.2 Hz, 1-POCH_2_CH_3_). ^31^P NMR (162 MHz, CDCl_3_) *δ* (22.97), 22.44. HRESIMS *m*/*z* 475.1997 [M + H]^+^ (475.2026 calcd for C_21_H_36_N_2_O_6_PS).

### *tert*-Butyl 2-((diethoxyphosphoryl)(2-(thiophen-3-yl)-acetamido)methyl)piperidine-1-carboxylate (4j)

The synthesis was performed according to general procedure A, 3 (235 mg, 0.670 mmol) and 2-(thiophene-3-yl)acetic acid (191 mg, 1.34 mmol, 2.0 equiv.) gave the total product (254 mg, 0.535 mmol, 80%). 4j-1 was obtained as s slightly yellow solid (109 mg, 0.230 mmol, 34%). 4j-2 was obtained as a slightly yellow solid (145 mg, 0.306 mmol, 46%).

### Compound 4j-1, diastereomer 1

^1^H NMR (400 MHz, CDCl_3_) *δ* 7.32 (dd, *J* = 5.0, 2.9 Hz, 1H, 14-H), 7.18 (d, *J* = 2.2 Hz, 1H, 12-H), 7.01 (d, *J* = 5.0 Hz, 1H, 15-H), 6.09 (br. s, 1H, 1-NH), 4.90 (ddd, ^2^*J*_*CP*_ = 17.2 Hz, *J* = 10.2, 10.2 Hz, 1H, 1-H), 4.37 (m, 1H, 2-H), 4.14–3.89 (m, 5H, 1-P(OCH_2_)_2_, 4-H), 3.63 (s, 2H, 10-H_2_), 2.85 (m, 1H, 4-H), 1.63–1.48 (m, 5H, 5-H, 6-H_2_, 7-H_2_), 1.45 (s, 9H, 2-NC_q_(O)OC_q_–(CH_3_)_3_), 1.35 (m, 1H, 5-H), 1.27 (t, *J* = 7.1 Hz, 3H, 1-POCH_2_CH_3_), 1.22 (t, *J* = 7.1 Hz, 3H, 1-POCH_2_CH_3_). ^13^C NMR (126 MHz, CDCl_3_) *δ* 170.0 (9-C_q_), 154.6 (2-N–C_q_(O)–O), 134.5 (11-C_q_), 128.5 (15-CH), 126.8 (14-CH), 123.5 (12-CH), 79.8 (2-NC_q_(O)O–C_q_–(CH_3_)_3_), 62.7 (br. s, 1-POCH_2_), 62.5 (d, ^2^*J*_*CP*_ = 6.6 Hz, 1-POCH_2_), 50.8 (2-CH), 44.7 (d, ^1^*J*_*CP*_ = 154.7 Hz, 1-CH), 40.5 (4-CH_2_), 38.2 (10-CH_2_), 28.5 (2-NC_q_(O)OC_q_–(CH_3_)_3_), 25.2 (d, ^3^*J*_*CP*_ = 9.7 Hz, 7-CH_2_), 24.8 (5-CH_2_), 19.4 (6-CH_2_), 16.5 (d, ^3^*J*_*CP*_ = 5.9 Hz, 1-POCH_2_CH_3_), 16.5 (d, ^3^*J*_*CP*_ = 6.6 Hz, 1-POCH_2_CH_3_). ^31^P NMR (162 MHz, CDCl_3_) *δ* 22.37. HRESIMS *m*/*z* 475.2032 [M + H]^+^ (475.2026 calcd for C_21_H_36_N_2_O_6_PS).

### Compound 4j-2, diastereomer 2 (rotamer ratio 2.8 : 1)

^1^H NMR (400 MHz, CDCl_3_) *δ* 7.27 (dd, *J* = 5.0, 3.1 Hz, 1H, 14-H), 7.15–7.12 (m, 1H, 12-H), 7.00 (dd, *J* = 5.0, 1.3 Hz, 1H, 15-H), 6.12 (d, *J* = 9.9 Hz, 1H, 1-NH), 4.84 (ddd, ^2^*J*_*CP*_ = 15.5 Hz, *J* = 11.6, 10.0 Hz, 1H, 1-H), 4.56 (m, 1H, 2-H), 4.19–3.95 (m, 4H, 1-P(OCH_2_)_2_), 3.88 (m, 1H, 4-H), 3.50 (s, 2H, 10-H), 2.78 (dd, *J* = 13.4, 13.4, 2.6 Hz, 1H, 4-H), 2.32 (m, 1H, 7-H), 1.67–1.55 (m, 4H, 5-H, 6-H_2_, 7-H), 1.44 (s, 9H, 2-NC_q_(O)OC_q_–(CH_3_)_3_), 1.36 (m, 1H, 5-H), 1.23 (t, *J* = 6.9 Hz, 3H, 1-POCH_2_CH_3_), 1.22 (t, *J* = 6.7 Hz, 3H, 1-POCH_2_CH_3_). ^13^C NMR (126 MHz, CDCl_3_) *δ* 169.9 (d, ^3^*J*_*CP*_ = 3.6 Hz, 9-C_q_), 156.4 (2-N–C_q_(O)–O), 134.5 (11-C_q_), 128.5 (15-CH), 126.2 (14-CH), 123.0 (12-CH), 80.0 (2-NC_q_(O)O–C_q_–(CH_3_)_3_), 62.8 (d, ^2^*J*_*CP*_ = 6.0 Hz, 1-POCH_2_), 62.6 (d, ^2^*J*_*CP*_ = 7.2 Hz, 1-POCH_2_), 49.8 (d, ^2^*J*_*CP*_ = 12.0 Hz, 2-CH), 45.3 (d, ^1^*J*_*CP*_ = 151.5 Hz, 1-CH), 40.1 (4-CH_2_), 38.3 (10-CH_2_), 28.6 (2-NC_q_(O)OC_q_–(CH_3_)_3_), 26.2 (7-CH_2_), 25.2 (5-CH_2_), 19.2 (6-CH_2_), 16.5 (d, ^3^*J*_*CP*_ = 5.4 Hz, 1-POCH_2_CH_3_), 16.4 (d, ^3^*J*_*CP*_ = 6.2 Hz, 1-POCH_2_CH_3_). ^31^P NMR (162 MHz, CDCl_3_) *δ* (23.29), 22.69. HRESIMS *m*/*z* 475.2012 [M + H]^+^ (475.2026 calcd for C_21_H_36_N_2_O_6_PS).

### *tert*-Butyl 2-((2-(benzo[*b*]thiophen-2-yl)acetamido)(di-ethoxyphosphoryl)methyl)piperidine-1-carboxylate (4k)

The synthesis was performed according to general procedure A, 3 (308 mg, 0.879 mmol) and 2-(benzo[*b*]thiophen-2-yl)acetic acid (330 mg, 1.72 mmol, 2.0 equiv.) gave the total product (280 mg, 0.534 mmol, 61%). 4k-1 was obtained as a slightly orange solid (125 mg, 0.238 mmol, 27%). 4k-2 was obtained as a slightly orange solid (154 mg, 0.294 mg, 33%).

### Compound 4k-1, diastereomer 1

^1^H NMR (400 MHz, CDCl_3_) *δ* 7.74 (dd, *J* = 7.8, 1.0 Hz, 1H, 14-H), 7.67 (dd, *J* = 6.9, 1.1 Hz, 1H, 17-H), 7.30 (ddd, *J* = 7.3, 7.3, 1.3 Hz, 1H, part C of ABCD, 16-H), 7.24 (ddd, *J* = 7.3, 7.3, 1.3 Hz, 1H, part D of ABCD, 15-H), 7.18 (d, *J* = 1.1 Hz, 1H, 19-H), 7.00 (br. s, 1H, 1-NH), 4.89 (m, 1H, 1-H), 4.44 (m, 1H, 2-H), 4.16–3.90 (m, 5H, 1-P(OCH_2_)_2_, 4-H), 3.88 (s, 2H, 10-H_2_), 2.99–2.69 (m, 1H, 4-H), 1.73–1.41 (m, 5H, 5-H, 6-H_2_, 7-H_2_), 1.40 (s, 9H, 2-NC_q_(O)OC_q_–(CH_3_)_3_), 1.34 (m, 1H, 5-H), 1.24 (t, *J* = 7.1 Hz, 3H, 1-POCH_2_CH_3_), 1.12 (t, *J* = 7.1 Hz, 3H, 1-POCH_2_CH_3_). ^13^C NMR (126 MHz, CDCl_3_) *δ* 168.9 (9-C_q_), 154.5 (2-N–C_q_(O)–O), 140.1 (13-C_q_), 139.8 (18-C_q_), 137.2 (11-C_q_), 124.4 (16-CH), 124.2 (14-CH), 123.8 (19-CH), 123.3 (17-CH), 122.2 (14-CH), 79.6 (2-NC_q_(O)O–C_q_–(CH_3_)_3_), 62.8 (d, ^2^*J*_*CP*_ = 7.1 Hz, 1-POCH_2_), 62.3 (d, ^2^*J*_*CP*_ = 6.7 Hz, 1-POCH_2_), 50.7 (2-CH), 44.7 (d, ^1^*J*_*CP*_ = 156.0 Hz, 1-CH), 40.4 (4-CH_2_), 38.28 (10-CH_2_), 28.4 (2-NC_q_(O)OC_q_–(CH_3_)_3_), 25.2 (d, ^3^*J*_*CP*_ = 10.0 Hz, 7-CH_2_), 24.8 (5-CH_2_), 19.3 (6-CH_2_), 16.4 (d, ^3^*J*_*CP*_ = 5.9 Hz, 1-P(OCH_2_CH_3_)_2_). ^31^P NMR (162 MHz, CDCl_3_) *δ* 22.30. HRESIMS *m*/*z* 525.2177 [M + H]^+^ (525.2183 calcd for C_25_H_38_N_2_O_6_PS).

### Compound 4k-2, diastereomer 2 (rotamer ratio 2.7 : 1)

^1^H NMR (500 MHz, CDCl_3_) *δ* 7.69 (d, *J* = 7.8 Hz, 1H, 14-H), 7.62 (d, *J* = 7.5 Hz, 1H, 17-H), 7.24 (m, 1H, 16-H), 7.21 (m, 1H, 15-H), 7.09 (s, 1H, 19-H), 6.43 (m, 1H, 1-NH), 4.80 (ddd, ^2^*J*_*HP*_ = 15.5 Hz, *J* = 10.7, 10.7 Hz, 1H, 1-H), 4.51 (m, 1H, 2-H), 4.10–3.89 (m, 4H, 1-P(OCH_2_)_2_), 3.83 (m, 1H, 4-H), 3.70 (d, ^2^*J* = 15.9 Hz, 1H, part A of AB, 10-H), 3.68 (d, ^2^*J* = 15.9 Hz, 1H, part B of AB, 10-H), 2.74 (ddd, *J* = 13.4, 13.4, 2.6 Hz, 1H, 4-H), 2.25 (br. d, *J* = 10.5 Hz, 1H, 7-H), 1.60–1.49 (m, 4H, 5-H, 6-H_2_, 7-H), 1.37 (s, 9H, 2-NC_q_(O)OC_q_–(CH_3_)_3_), 1.32 (m, 1H, 5-H), 1.15 (t, *J* = 7.1 Hz, 3H, 1-POCH_2_CH_3_), 1.08 (t, *J* = 7.0 Hz, 3H, 1-POCH_2_CH_3_). ^13^C NMR (126 MHz, CDCl_3_) *δ* 168.5 (d, *J* = 3.3 Hz, 9-C_q_), 156.2 (2-N–C_q_(O)–O), 140.0 (13-C_q_), 139.7 (18-C_q_), 137.1 (11-C_q_), 124.3 (16-CH), 124.1 (15-CH), 123.3 (19-CH), 123.2 (17-CH), 122.0 (14-CH), 79.9 (2-NC_q_(O)O–C_q_–(CH_3_)_3_), 62.7 (d, ^2^*J*_*CP*_ = 5.2 Hz, 1-POCH_2_), 62.6 (d, ^2^*J*_*CP*_ = 7.3 Hz, 1-POCH_2_), 49.7 (d, ^2^*J*_*CP*_ = 11.9 Hz, 2-CH), 45.4 (d, ^1^*J*_*CP*_ = 151.6 Hz, 1-CH), 40.0 (4-CH_2_), 38.3 (10-CH_2_), 28.4 (2-NC_q_(O)OC_q_–(CH_3_)_3_), 26.1 (7-CH_2_), 25.1 (5-CH_2_), 19.0 (6-CH_2_), 16.3 (d, ^3^*J*_*CP*_ = 3.9 Hz, 1-POCH_2_CH_3_), 16.2 (d, ^3^*J*_*CP*_ = 4.9 Hz, 1-POCH_2_CH_3_). ^31^P NMR (162 MHz, CDCl_3_) *δ* (22.87), 22.42. HRESIMS *m*/*z* 525.2191 [M + H]^+^ (525.2183 calcd for C_25_H_38_N_2_O_6_PS).

### *tert*-Butyl 2-((2-(benzo[*b*]thiophen-3-yl)acetamido)(di-ethoxyphosphoryl)methyl)piperidine-1-carboxylate (4l)

The synthesis was performed according to general procedure A, 3 (301 mg, 0.860 mmol) and 2-(benzo[*b*]thiophen-3-yl)acetic acid (330 mg, 1.72 mmol, 2.0 equiv.) gave the total product (304 mg, 0.579 mg, 68%). 4l-1 was obtained as a colorless solid (141 mg, 0.269 mmol, 31%). 4l-2 was obtained as a colorless solid (163 mg, 0.311 mmol, 36%).

### Compound 4l-1, diastereomer 1

^1^H NMR (400 MHz, CDCl_3_) *δ* 7.86 (m, 1H, 15-H), 7.78 (m, 1H, 18-H), 7.44–7.32 (m, 3H, 16-H, 17-H, 12-H), 6.30 (br. s, 1H, 1-NH), 4.88 (ddd,^2^*J*_*PH*_ = 18.8 Hz, *J* = 10.5, 10.5 Hz, 1H, 1-H), 4.32 (m, 1H, 2-H), 4.07–3.88 (m, 4H, 1-P(OCH_2_)_2_), 3.86 (s, 2H, 10-H_2_), 3.83 (m, 1H, 4-H), 2.82 (m, 1H, 4-H), 1.61–1.47 (m, 4H, 5-H, 6-H_2_, 7-H), 1.41 (s, 9H, 2-NC_q_(O)OC_q_–(CH_3_)_3_), 1.38–1.27 (m, 2H, 5-H, 7-H), 1.23 (t, *J* = 7.1 Hz, 3H, 1-POCH_2_CH_3_), 1.09 (t, *J* = 7.1 Hz, 3H, 1-POCH_2_CH_3_). ^13^C NMR (126 MHz, CDCl_3_) *δ* 169.5 (9-C_q_), 154.7 (2-N–C_q_(O)–O), 140.6 (14-C_q_), 138.4 (19-C_q_), 129.2 (11-C_q_), 125.3 (12-CH), 124.9 (16-CH), 124.5 (17-CH), 123.0 (15-CH), 122.1 (18-CH), 79.7 (2-NC_q_(O)O–C_q_–(CH_3_)_3_), 62.6 (d, ^2^*J*_*CP*_ = 4.3 Hz, 1-POCH_2_), 62.4 (d, ^2^*J*_*CP*_ = 6.5 Hz, 1-POCH_2_), 50.7 (2-CH), 44.8 (d, ^1^*J*_*CP*_ = 154.6 Hz, 1-CH), 40.4 (4-CH_2_), 36.89 (10-CH_2_), 28.5 (2-NC_q_(O)OC_q_–(CH_3_)_3_), 25.2 (d, ^3^*J*_*CP*_ = 9.4 Hz, 7-CH_2_), 24.7 (5-CH_2_), 19.27 (6-CH_2_), 16.4 (d, ^3^*J*_*CP*_ = 6.1 Hz, 1-POCH_2_CH_3_), 16.4 (d, ^3^*J*_*CP*_ = 5.9 Hz, 1-POCH_2_CH_3_). ^31^P NMR (162 MHz, CDCl_3_) *δ* 22.05. HRESIMS *m*/*z* 525.2181 [M + H]^+^ (525.2183 calcd for C_25_H_38_N_2_O_6_PS).

### Compound 4l-2, diastereomer 2 (rotamer ratio 2.5 : 1)

^1^H NMR (400 MHz, CDCl_3_) *δ* 7.84 (d, *J* = 7.0 Hz, 1H, 15-H), 7.74 (d, *J* = 7.5 Hz, 1H, 18-H), 7.38 (dd, *J* = 7.0, 7.0 Hz, 1H, 17-H), 7.35 (m, 1H, 12-H), 7.33 (m, 1H, 16-H), 6.17 (d, *J* = 9.9 Hz, 1H, 1-NH), 4.84 (ddd, ^2^*J*_*HP*_ = 15.4 Hz, *J* = 11.7, 9.9 Hz, 1H, 1-H), 4.55 (m, 1H, 2-H), 4.14–3.80 (m, 5H, 1-P(OCH_2_)_2_, 4-H), 3.71 (s, 2H, 10-H_2_), 2.82 (ddd, *J* = 13.5, 13.5, 2.6 Hz, 1H, 4-H), 2.34 (m, 1H, 7-H), 1.67–1.55 (m, 4H, 5-H, 6-H_2_, 7-H), 1.46 (s, 9H, 2-NC_q_(O)OC_q_–(CH_3_)_3_), 1.43 (m, 1H, 5-H), 1.14 (t, *J* = 7.1 Hz, 3H, 1-POCH_2_CH_3_), 1.08 (t, *J* = 7.1 Hz, 3H, 1-POCH_2_CH_3_).^13^C NMR (126 MHz, CDCl_3_) *δ* 169.2 (d, *J* = 3.6 Hz, 9-C_q_), 156.3 (2-N–C_q_(O)–O), 140.3 (14-C_q_), 138.4 (19-C_q_), 129.1 (11-C_q_), 124.5 (16-CH or 12-CH), 124.5 (16-CH or 12-CH), 124.2 (17-CH), 122.8 (15-CH), 121.9 (18-CH), 79.9 (2-NC_q_(O)O–C_q_–(CH_3_)_3_), 62.7 (d, ^2^*J*_*CP*_ = 6.0 Hz, 1-POCH_2_), 62.4 (d, ^2^*J*_*CP*_ = 7.3 Hz, 1-POCH_2_), 49.7 (d, ^2^*J*_*CP*_ = 11.9 Hz, 2-CH), 45.3 (d, ^1^*J*_*CP*_ = 151.5 Hz, 1-CH), 40.1 (4-CH_2_), 36.8 (10-CH_2_), 28.5 (OC_q_–(CH_3_)_3_), 26.1 (7-CH_2_), 25.1 (5-CH_2_), 19.1 (6-CH_2_), 16.2 (d, ^3^*J*_*CP*_ = 1.6 Hz, 1-POCH_2_CH_3_), 16.2 (d, ^3^*J*_*CP*_ = 2.2 Hz, 1-POCH_2_CH_3_). ^31^P NMR (162 MHz, CDCl_3_) *δ* (22.93), 22.32. HRESIMS *m*/*z* 525.2153 [M + H]^+^ (525.2183 calcd for C_25_H_38_N_2_O_6_PS).

### General procedure B for 5a–l

Corresponding compounds 4 and 3-mercaptopropyl-functionalized silica gel (3.5 equiv., 1.2 mmol g^−1^ loading) were dried under high vacuum for at least 30 min. Dry MeCN and Et_3_N (6.0 equiv.) were added, and the mixture was stirred at r.t. for 5 min. The reaction mixture was subsequently cooled to 0 °C. TMSBr (5.0 equiv.) in MeCN was added dropwise. The reaction was stirred for 12–48 h. All the compounds were converted to HCl salts by adding 0.1 M HCl solution (aq) and the solvent was removed by freezedrying, due to poor solubility of either the free base or formate salts.

### ((2-(4-Methyl-2-thioxo-2,3-dihydrothiazol-5-yl)acet-amido)(piperidin-2-yl)methyl)phosphonic acid hydro-chloride (5a)

The synthesis was performed according to general procedure B. Compound 4a-1 (50.8 mg, 0.0974 mmol) gave 5a-1 as a slightly orange solid (18.8 mg, 0.0515 mmol, 48%). Compound 4a-2 (50.6 mg, 0.0970 mmol) gave 5a-2 as a slightly orange solid (8.6 mg, 0.0235 mmol, 22%).

### Compound 5a-1, diastereomer 1

^1^H NMR (500 MHz, DMSO-*d*_6_) *δ* 12.94 (s, 1H, 15-NH), 8.32 (s, 1H, 1-NH), 3.96 (dd, ^2^*J*_*HP*_ = 15.9 Hz, *J* = 8.5, 8.5 Hz, 1H, 1-H), 3.55 (d, ^2^*J* = 17.3 Hz, 1H, part A of AB, 10-H), 3.54 (d, ^2^*J* = 17.3 Hz, 1H, part B of AB, 10-H), 3.23 (br. d, 12.3 Hz, 1H, 4-H), 3.09 (ddd, *J* = 8.8, 8.8, 8.8 Hz, 1H, 2-H), 2.84 (ddd, *J* = 12.0, 12.0, 2.3 Hz, 1H, 4-H), 2.06 (s, 3H, 15-CH_3_), 1.81 (br. d, *J* = 10.5 Hz, 1H, 7-H), 1.76–1.59 (m, 2H, 5-H, 6-H), 1.52 (m, 1H, 6-H), 1.45–1.30 (m, 2H, 5-H, 7-H). ^13^C NMR (126 MHz, DMSO-*d*_6_) *δ* 186.9 (13-C_q_), 168.9 (d, ^3^*J*_*CP*_ = 6.2 Hz, 9-C_q_), 134.7 (15-C_q_), 117.5 (11-C_q_), 57.3 (2-CH), 48.6 (d, ^1^*J*_*CP*_ = 134.7 Hz, 1-CH), 43.7 (4-CH_2_), 32.2 (10-CH_2_), 25.0 (d, ^3^*J*_*CP*_ = 7.6 Hz, 7-CH_2_), 22.0 (5-CH_2_), 21.6 (6-CH_2_), 11.4 (15-CH_3_). ^31^P NMR (162 MHz, DMSO-*d*_6_) *δ* 11.71. HRESIMS *m*/*z* 366.0718 [M + H–Cl]^+^ (366.0706 calcd for C_12_H_21_N_3_O_4_PS_2_).

### Compound 5a-2, diastereomer 2

^1^H NMR (500 MHz, DMSO-*d*_6_) *δ* 12.92 (s, 1H, 15-NH), 8.69 (s, 1H, 1-NH), 4.01 (ddd, ^2^*J*_*HP*_ = 17.1 Hz, *J* = 8.8, 3.5 Hz, 1H, 1-H), 3.61 (d, ^2^*J* = 16.7 Hz, 1H, part A of AB, 10-H), 3.56 (d, ^2^*J* = 16.7 Hz, 1H, part B of AB, 10-H), 3.34–3.13 (m, 2H, 2-H, 4-H), 2.82 (dd, *J* = 12.4, 12.4 Hz, 1H, 4-H), 2.07 (s, 3H, 15-CH_3_), 1.82–1.70 (m, 2H, 6-H, 7-H), 1.69–1.47 (m, 3H, 5-H_2_, 7-H), 1.41 (m, 1H, 6-H). ^13^C NMR (126 MHz, DMSO-*d*_6_) *δ* 186.9 (13-C_q_), 168.8 (d, ^3^*J*_*CP*_ = 6.7 Hz, 9-C_q_), 134.7 (15-CH), 117.5 (11-CH), 57.1 (2-CH), 48.9 (d, ^1^*J*_*CP*_ = 134.7 Hz, 1-CH), 44.3 (4-CH_2_), 32.3 (10-CH_2_), 25.6 (d, ^3^*J*_*CP*_ = 5.2 Hz, 7-CH_2_), 22.1 (5-CH_2_), 21.8 (6-CH_2_), 11.4 (15-CH_3_). ^31^P NMR (162 MHz, DMSO-*d*_6_) *δ* 11.79. HRESIMS *m*/*z* 366.0699 [M + H–Cl]^+^ (calcd for C_12_H_21_N_3_O_4_PS_2_).

### (Piperidin-2-yl(thiophene-2-carboxamido)methyl)phos-phonic acid hydrochloride (5b)

The synthesis was performed according to general procedure B. Compound 4b-1 (31.5 mg, 0.0684 mmol) gave 5b-1 as a colorless solid (15.8 mg, 0.0464 mmol, 68%). Compound 4b-2 (40.1 mg, 0.0871 mmol) gave 5b-2 as a colorless solid (22.5 mg, 0.0660 mmol, 76%).

### Compound 5b-1, diastereomer 1

^1^H NMR (400 MHz, DMSO-*d*_6_) *δ* 8.53 (m, 1H, 1-NH), 8.10 (m, 1H, 14-H), 7.80 (d, *J* = 4.9 Hz, 1H, 12-H), 7.16 (dd, *J* = 5.0, 3.7 Hz, 1H, 13-H), 4.57 (m, 1H, 1-H), 3.42 (m, 1H, 2-H), 3.24 (m, 1H, 4-H), 2.94 (m, 1H, 4-H), 2.06 (br. d, *J* = 13.5 Hz, 1H, 7-H), 1.78–1.38 (m, 1H, 5-H_2_, 6-H_2_), 1.57 (m, 1H, 7-H). ^13^C NMR (126 MHz, DMSO-*d*_6_) *δ* 161.7 (d, ^3^*J*_*CP*_ = 5.6 Hz, 9-C_q_), 139.2 (10-C_q_), 131.3 (12-CH), 129.4 (14-CH), 127.9 (13-CH), 57.1 (d, ^2^*J*_*CP*_ = 8.1 Hz, 2-CH), 48.6 (d, ^1^*J*_*CP*_ = 145.5 Hz, 1-CH), 44.8 (4-CH_2_), 24.6 (7-CH_2_), 21.5 (5-CH_2_, 6-CH_2_). ^31^P NMR (162 MHz, DMSO-*d*_6_) *δ* 14.26. HRESIMS *m*/*z* 305.0751 [M + H–Cl]^+^ (305.0719 calcd for C_11_H_18_N_2_O_4_PS).

### Compound 5b-2, diastereomer 2

^1^H NMR (400 MHz, DMSO-*d*_6_) *δ* 8.68 (d, *J* = 9.7 Hz, 1H, 1-NH), 8.14 (d, *J* = 3.7, 1.1 Hz, 1H, 14-H), 7.79 (dd, *J* = 5.0, 1.1 Hz, 1H, 12-H), 7.17 (dd, *J* = 5.0, 3.7 Hz, 1H, 13-H), 4.44 (ddd, ^2^*J*_*HP*_ = 17.7 Hz, *J* = 9.7, 7.2 Hz, 1H, 1-H), 3.45 (m, 1H, 2-H), 3.28 (br. d, *J* = 12.2 Hz, 1H, 4-H), 2.86 (dd, *J* = 11.3, 11.3 Hz, 1H, 4-H), 2.07 (br. d, *J* = 12.1 Hz 1H, 7-H), 1.81–1.72 (m, 1H, 6-H or 5-H), 1.68 (m, 1H, 5-H or 6-H), 1.65–1.55 (m, 2H, 7-H, 5-H or 6-H), 1.47 (m, 1H, 5-H or 6-H). ^13^C NMR (126 MHz, DMSO-*d*_6_) *δ* 161.3 (d, *J* = 4.0 Hz, 9-C_q_), 139.2 (10-CH), 131.2 (12-CH), 129.5 (14-CH), 127.9 (13-CH), 56.3 (d, ^2^*J*_*CP*_ = 7.1 Hz, 2-CH), 49.1 (d, ^1^*J*_*CP*_ = 147.1 Hz, 1-CH), 44.9 (4-CH_2_), 26.1 (d, ^3^*J*_*CP*_ = 4.5 Hz, 7-CH_2_), 21.7 (5-CH_2_ or 6-CH_2_), 21.5 (6-CH_2_ or 5-CH_2_). ^31^P NMR (162 MHz, DMSO-*d*_6_) *δ* 14.67. HRESIMS *m*/*z* 305.0761 [M + H–Cl]^+^ (305.0719 calcd for C_11_H_18_N_2_O_4_PS).

### (Piperidin-2-yl(thiophene-3-carboxamido)methyl)phos-phonic acid hydrochloride (5c)

The synthesis was performed according to general procedure B. Compound 4c-1 (32.6 mg, 0.0708 mmol) gave 5c-1 as a colorless solid (22.6 mg, 0.0663 mmol, 94%). Compound 4c-1 (32.6 mg, 0.0974 mmol) gave 5c-2 as a colorless solid (21.8 mg, 0.0640 mmol, 90%).

### Compound 5c-1, diastereomer 1

^1^H NMR (400 MHz, DMSO-*d*_6_) *δ* 8.37 (dd, *J* = 2.9, 1.4 Hz, 1H, 11-H), 8.23 (d, *J* = 8.1 Hz, 1H, 1-NH), 7.58 (m, 1H, 14-H), 7.56 (m, 1H, 13-H), 4.48 (ddd, ^2^*J*_*HP*_ = 16.7 Hz, *J* = 9.0, 6.5 Hz, 1H, 1-H), 3.35 (m, 1H, 2-H), 2.89 (ddd, *J* = 12.3, 12.3, 3.5 Hz, 1H, 4-H), 2.01 (br. d, *J* = 12.9 Hz, 1H, 7-H), 1.78–1.61 (m, 2H, 5-H, 6-H), 1.61–1.47 (m, 1H, 5-H or 6-H, 7-H), 1.40 (m, 1H, 5-H or 6-H). ^13^C NMR (126 MHz, DMSO-*d*_6_) *δ* 162.4 (d, ^3^*J*_*CP*_ = 5.7 Hz, 9-C_q_), 137.1 (10-C_q_), 129.5 (11-CH), 127.4 (14-CH), 126.4 (13-CH), 57.3 (d, ^2^*J*_*CP*_ = 6.2 Hz, 2-CH), 48.4 (d, ^1^*J*_*CP*_ = 141.9 Hz, 1-CH), 44.5 (4-CH_2_), 24.8 (d, ^3^*J*_*CP*_ = 5.2 Hz, 7-CH_2_), 21.7 (5-CH_2_ or 6-CH_2_), 21.6 (5-CH_2_ or 6-CH_2_). ^31^P NMR (162 MHz, DMSO-*d*_6_) *δ* 13.77. HRESIMS *m*/*z* 305.0737 [M + H–Cl]^+^ (305.0719 calcd for C_11_H_18_N_2_O_4_PS).

### Compound 5c-2, diastereomer 2

^1^H NMR (500 MHz, DMSO-*d*_6_) *δ* 8.44 (m, 1H, 11-H), 7.65 (d, *J* = 5.0 Hz, 1H, 14-H), 7.58 (dd, *J* = 5.1, 2.9 Hz, 1H, 13-H), 4.47 (ddd, ^2^*J*_*HP*_ = 17.7 Hz, *J* = 9.7, 6.9 Hz, 1H, 1-H), 3.44 (m, 1H, 2-H), 3.29 (br. d, *J* = 12.9 Hz, 1H, 4-H), 2.86 (dd, *J* = 12.6, 12.6 Hz, 1H, 4-H), 2.06 (br. d, *J* = 11.9 Hz, 1H, 7-H), 1.81–1.39 (m, 4H, 5-H_2_, 6-H_2_), 1.61 (m, 1H, 7-H). ^13^C NMR (126 MHz, DMSO-*d*_6_) *δ* 162.2 (d, ^3^*J*_*CP*_ = 4.0 Hz, 9-C_q_), 137.1 (10-C_q_), 129.8 (11-CH), 127.5 (14-CH), 126.3 (13-CH), 56.4 (d, ^2^*J*_*CP*_ = 6.9 Hz, 2-CH), 48.8 (d, ^1^*J*_*CP*_ = 147.0 Hz, 1-CH), 44.9 (4-CH_2_), 26.2 (d, ^3^*J*_*CP*_ = 4.8 Hz, 7-CH_2_), 21.7 (5-CH_2_ or 6-CH_2_), 21.5 (5-CH_2_ or 6-CH_2_). ^31^P NMR (162 MHz, DMSO-*d*_6_) *δ* 12.03. HRESIMS *m*/*z* 305.0745 [M + H–Cl]^+^ (305.07194 calcd for C_11_H_18_N_2_O_4_PS).

### (Piperidin-2-yl(4,5,6,7-tetrahydrobenzo[*b*]thiophene-2-carboxamido)methyl)phosphonic acid hydrochloride (5d)

The synthesis was performed according to general procedure B. Compound 4d-1 (52.1 mg, 0.101 mmol) gave 5d-1 as a slightly yellow solid (26.8 mg, 0.0679 mmol, 67%). Compound 4d-2 (45.8 mg, 0.0890 mmol) gave 5d-2 as a colorless solid (21.4 mg, 0.0542 mmol, 61%).

### Compound 5d-1, diastereomer 1

^1^H NMR (500 MHz, DMSO-*d*_6_) *δ* 8.30 (d, *J* = 9.7 Hz, 1H, 1-NH), 7.74 (s, 1H, 18-H), 4.53 (ddd, ^2^*J*_*HP*_ = 19.4 Hz, *J* = 9.5, 5.3 Hz, 1H, 1-H), 3.40 (m, 1H, 2-H), 3.26 (br. d, *J* = 12.4 Hz, 1H, 4-H), 2.97–2.88 (m, 1H, 4-H), 2.76–2.69 (m, 2H, 13-H_2_), 2.62–2.53 (m, 2H, 16-H_2_), 2.03 (br. d, *J* = 12.3 Hz, 1H, 7-H), 1.84–1.63 (m, 5H, 14-H_2_, 15-H_2_, 5-H or 6-H), 1.61–1.49 (m, 2H, 7-H, 5-H or 6-H), 1.42 (m, 1H, 5-H or 6-H). ^13^C NMR (126 MHz, DMSO-*d*_6_) *δ* 161.9 (d, ^3^*J*_*CP*_ = 5.6 Hz, 9-C_q_), 140.9 (12-C_q_), 135.8 (17-C_q_), 134.7 (10-C_q_), 130.1 (18-CH), 57.1 (d, ^2^*J*_*CP*_ = 8.3 Hz, 2-CH), 48.5 (d, ^1^*J*_*CP*_ = 145.5 Hz, 1-CH), 44.8 (4-CH_2_), 25.0 (16-CH_2_), 24.7 (13-CH_2_), 24.5 (d, ^3^*J*_*CP*_ = 4.3 Hz, 7-CH_2_), 22.9 (14-CH_2_), 22.2 (15-CH_2_), 21.5 (6-CH_2_, 5-CH_2_). ^31^P NMR (162 MHz, DMSO-*d*_6_) *δ* 14.58. HRESIMS *m*/*z* 359.1207 [M + H–Cl]^+^ (359.1189 calcd for C_15_H_24_N_2_O_4_PS).

### Compound 5d-2, diastereomer 2

^1^H NMR (500 MHz, DMSO-*d*_6_) *δ* 8.42 (d, *J* = 9.5 Hz, 1H, 1-NH), 7.75 (s, 1H, 18-H), 4.40 (ddd, ^2^*J*_*HP*_ = 17.7 Hz, *J* = 9.7, 7.4 Hz, 1H, 1-H), 3.41 (m, 1H, 2-H), 3.27 (br d, *J* = 10.6 Hz, 1H, 4-H), 2.84 (dd, *J* = 12.6, 12.6 Hz, 1H, 4-H), 2.76–2.68 (m, 2H, 13-H_2_), 2.66–2.53 (m, 2H, 16-H_2_), 2.08 (m, 1H, 7-H), 1.85–1.65 (m, 5H, 14-H_2_, 15-H_2_, 5-H or 6-H), 1.63–1.53 (m, 2H, 7-H, 5-H or 6-H), 1.44 (m, 1H, 5-H or 6-H). ^13^C NMR (126 MHz, DMSO-*d*_6_) *δ* 160.9 (d, ^3^*J*_*CP*_ = 3.9 Hz, 9-C_q_), 140.3 (12-C_q_), 135.2 (17-C_q_), 134.2 (10-C_q_), 129.5 (18-CH), 55.7 (d, ^2^*J*_*CP*_ = 7.2 Hz, 2-CH), 48.4 (d, ^1^*J*_*CP*_ = 147.1 Hz, 1-CH), 44.3 (4-CH_2_), 25.6 (d, ^3^*J*_*CP*_ = 4.2 Hz, 7-CH_2_), 24.5 (16-CH_2_), 24.1 (13-CH_2_), 22.3 (14-CH_2_), 21.7 (15-CH_2_), 21.1 (5-CH_2_ or 6-CH_2_), 20.9 (5-CH_2_ or 6-CH_2_). ^31^P NMR (162 MHz, DMSO-*d*_6_) *δ* 15.36. HRESIMS *m*/*z* 359.1219 [M + H–Cl]^+^ (359.1189 calcd for C_15_H_24_N_2_O_4_PS).

### (Piperidin-2-yl(4,5,6,7-tetrahydrobenzo[*b*]thiophene-3-carboxamido)methyl)phosphonic acid hydrochloride (5e)

The synthesis was performed according to the general procedure B. Compound 4e-1 (59.4 mg, 0.115 mmol) gave 5e-1 as a colorless solid (17.6 mg, 0.0446 mmol, 39%). Compound 4e-2 (68.5 mg, 0.133 mmol) gave 5e-2 as a colorless solid (11.5 mg, 0.0291 mmol, 22%).

### Compound 5e-1, diastereomer 1

^1^H NMR (400 MHz, DMSO-*d*_6_) *δ* 8.07 (s, 1H, 11-H), 7.95 (dd, *J* = 9.7, 3.5 Hz, 1H, 1-NH), 4.54 (ddd, ^2^*J*_*HP*_ = 19.2 Hz, *J* = 9.5, 5.5 Hz, 1H, 1-H), 3.39 (m, 1H, 2-H), 3.26 (br. d, *J* = 11.7 Hz, 1H, 4-H), 2.93 (ddd, *J* = 11.7, 11.7, 3.6 Hz, 1H, 1-H), 2.84–2.65 (m, 4H, 14-H_2_, 17-H_2_), 2.01 (br. d, *J* = 12.2 Hz, 1H, 7-H), 1.83–1.64 (m, 5H, 15-H_2_, 16-H_2_, 5-H or 6-H), 1.63–1.51 (m, 2H, 7-H, 5-H or 6-H), 1.44 (m, 1H, 5-H or 6-H). ^13^C NMR (101 MHz, DMSO-*d*_6_) *δ* 163.8 (9-C_q_), 136.0 (10-C_q_ or 13-C_q_ or 18-C_q_), 134.9 (10-C_q_ or 13-C_q_ or 18-C_q_), 134.8 (10-C_q_ or 13-C_q_ or 18-C_q_), 125.9 (11-CH), 57.1 (d, ^2^*J*_*CP*_ = 7.3 Hz, 2-CH), 47.9 (d, ^1^*J*_*CP*_ = 145.4 Hz, 1-CH), 44.7 (4-CH_2_), 25.4 (14-CH_2_ or 17-CH_2_), 24.7 (14-CH_2_ or 17-CH_2_), 24.5 (d, ^3^*J*_*CP*_ = 3.8 Hz, 7-CH_2_), 22.7 (14-CH_2_ or 17-CH_2_), 22.1 (14-CH_2_ or 17-CH_2_), 21.5 (5-CH_2_, 6-CH_2_). ^31^P NMR (162 MHz, DMSO-*d*_6_) *δ* 14.73. HRESIMS *m*/*z* 359.1161 [M + H–Cl]^+^ (359.1189 calcd for C_15_H_24_N_2_O_4_PS).

### Compound 5e-2, diastereomer 2

^1^H NMR (500 MHz, DMSO-*d*_6_) *δ* 8.22 (s, 1H, 11-H), 8.17 (d, *J* = 9.9 Hz, 1H, 1-NH), 4.45 (ddd, ^2^*J*_*HP*_ = 17.9 Hz, *J* = 8.8, 8.8 Hz, 1H, 1-H), 3.45 (m, 1H, 2-H), 3.26 (br. d, *J* = 12.4 Hz, 1H, 4-H), 2.85 (m, 1H, 4-H), 2.81–2.67 (m, 4H, 14-H_2_, 17-H_2_), 2.10 (br. d, *J* = 13.9 Hz, 1H, 7-H), 1.79–1.36 (m, 9H, 5-H_2_, 6-H_2_, 7-H, 15-H_2_, 16-H_2_). ^13^C NMR (126 MHz, DMSO-*d*_6_) *δ* 163.5 (d, ^3^*J*_*CP*_ = 3.4 Hz, 9-C_q_), 135.9 (10-C_q_ or 13-C_q_ or 18-C_q_), 135.2 (10-C_q_ or 13-C_q_ or 18-C_q_), 135.0 (10-C_q_ or 13-C_q_ or 18-C_q_), 126.0 (11-CH), 55.9 (d, ^2^*J*_*CP*_ = 8.5 Hz, 2-CH), 48.4 (d, ^1^*J*_*CP*_ = 147.7 Hz, 1-CH), 44.7 (4-CH_2_), 26.0 (7-CH_2_), 25.4 (14-CH_2_ or 17-CH_2_), 24.7 (14-CH_2_ or 17-CH_2_), 22.8 (15-CH_2_ or 16-CH_2_), 22.2 (15-CH_2_ or 16-CH_2_), 21.6 (5-CH_2_ or 6-CH_2_), 21.5 (5-CH_2_ or 6-CH_2_). ^31^P NMR (162 MHz, DMSO-*d*_6_) *δ* 14.34. HRESIMS *m*/*z* 3 591 170 [M + H–Cl]^+^ (359.1189 calcd for C_15_H_24_N_2_O_4_PS).

### (Piperidin-2-yl(4,5,6,7-tetrahydrobenzo[*c*]thiophene-1-carboxamido)methyl)phosphonic acid hydrochloride (5f)

The synthesis was performed according to general procedure B. Compound 4f-1 (45.3 mg, 0.0880 mmol) gave 5f-1 as a colorless solid (28.9 mg, 0.0732 mmol, 83%). Compound 4f-2 (56.1 mg, 0.109 mmol) gave 5f-2 as a colorless solid (32.2 mg, 0.0815 mmol, 75%).

### Compound 5f-1, diastereomer 1

^1^H NMR (500 MHz, DMSO-*d*_6_) *δ* 7.32 (s, 1H, 12-H), 7.02 (m, 1H, 1-NH), 4.33 (m, 1H, 1-H), 3.32 (m, 1H, 2-H), 3.28 (m, 1H, 4-H), 3.00–2.92 (m, 2H, 17-CH_2_), 2.88 (m, 1H, 4-H), 2.72–2.62 (m, 2H, 14-H_2_), 1.95 (br. d, *J* = 13.2 Hz, 1H, 7-H), 1.81–1.63 (m, 6H, 15-H_2_, 16-H_2_, 5-H, 6-H), 1.61–1.48 (m, 2H, 7-H, 5-H or 6-H), 1.43 (m, 1H, 5-H or 6-H). ^13^C NMR (126 MHz, DMSO-*d*_6_) *δ* 162.5 (d, ^3^*J*_*CP*_ = 6.2 Hz, 9-C_q_), 140.2 (13-C_q_), 139.5 (18-C_q_), 131.0 (10-C_q_), 123.5 (12-CH), 57.4 (d, ^2^*J*_*CP*_ = 5.7 Hz, 2-CH), 48.5 (d, ^1^*J*_*CP*_ = 139.5 Hz, 1-CH), 44.4 (4-CH_2_), 26.2 (17-CH_2_), 26.0 (14-CH_2_), 24.8 (d, ^3^*J*_*CP*_ = 5.5 Hz, 7-CH_2_), 22.7 (15-CH_2_ or 16-CH_2_), 22.4 (15-CH_2_ or 16-CH_2_), 21.7 (5-CH_2_ or 6-CH_2_), 21.6 (5-CH_2_ or 6-CH_2_). ^31^P NMR (162 MHz, DMSO-*d*_6_) *δ* 13.89. HRESIMS *m*/*z* 359.1149 [M + H–Cl]^+^ (359.1189 calcd for C_15_H_24_N_2_O_4_PS).

### Compound 5f-2, diastereomer 2

^1^H NMR (500 MHz, DMSO-*d*_6_) *δ* 7.30 (s, 1H, 12-H), 4.33 (m, 1H, 1-H), 3.42 (m, 1H, 2-H), 3.28 (br. d, *J* = 12.1 Hz, 1H, 4-H), 2.98 (m, 2H, 17-H_2_), 2.83 (dd, *J* = 12.7, 12.7, 3.0 Hz, 1H, 4-H), 2.66 (m, 2H, 14-CH_2_), 1.99 (m, 1H, 7-H), 1.81–1.49 (m, 6H, 15-CH_2_, 16-CH_2_, 7-H, 5-H′, 6H', 5-H′′ or 6-H′′), 1.42 (m, 1H, 5-H′′ or 6-H′′). ^13^C NMR (126 MHz, DMSO-*d*_6_) *δ* 162.3 (d, ^3^*J*_*CP*_ = 5.4 Hz, 9-C_q_), 140.6 (13-C_q_), 139.3 (18-C_q_), 130.7 (10-C_q_), 123.3 (12-C_q_), 56.3 (d, ^2^*J*_*CP*_ = 4.8 Hz, 2-CH), 49.0 (d, ^1^*J*_*CP*_ = 142.7 Hz, 1-CH), 44.7 (4-CH_2_), 26.2 (17-CH_2_), 26.0 (14-CH_2_), 25.9 (d, ^3^*J*_*CP*_ = 4.8 Hz, 7-CH_2_), 22.7 (15-CH_2_ or 16-CH_2_), 22.4 (15-CH_2_ or 16-CH_2_), 21.9 (5-CH_2_ or 6-CH_2_), 21.6 (5-CH_2_ or 6-CH_2_). ^31^P NMR (162 MHz, DMSO-*d*_6_) *δ* 13.37. HRESIMS *m*/*z* 359.1155 [M + H–Cl]^+^ (359.1189 calcd for C_15_H_24_N_2_O_4_PS).

### ((Benzo[*b*]thiophene-2-carboxamido)(piperidin-2-yl)methyl)phosphonic acid hydrochloride (5g)

The synthesis was performed according to general procedure B. Compound 4g-1 (49.7 mg, 0.0973 mmol) gave 5g-1 as a colorless solid (19.7 mg, 0.0504 mmol, 52%). Compound 4g-2 (51.5 mg, 0.101 mmol) gave 5g-2 as a colorless solid (20.8 mg, 0.0532 mmol, 53%).

### Compound 5g-1, diastereomer 1

^1^H NMR (400 MHz, DMSO-*d*_6_) *δ* 8.81 (m, 1H, 1-NH), 8.47 (s, 1H, 18-H), 8.02 (d, *J* = 7.7 Hz, 1H, 13-H), 7.95 (d, *J* = 7.8 Hz, 1H, 18-H), 7.47 (ddd, *J* = 8.1, 8.1, 1.5 Hz, 1H, part C of ABCD, 14-H), 7.44 (ddd, *J* = 8.1, 8.1, 1.6 Hz, 1H, part D of ABCD, 15-H), 4.66 (ddd, ^2^*J*_*HP*_ = 19.7 Hz, *J* = 9.9, 5.0 Hz, 1H, 1-H), 3.49 (m, 1H, 2-H), 3.28 (br. d, *J* = 12.7 Hz, 1H, 4-H), 2.95 (br. dd, *J* = 11.9, 11.9 Hz, 1H, 4-H), 2.11 (br. d, *J* = 13.6 Hz, 1H, 7-H), 1.76 (m, 1H, 5-H), 1.71–1.53 (m, 4H, 6-H_2_, 7-H), 1.45 (m, 1H, 5-H). ^13^C NMR (126 MHz, DMSO-*d*_6_) *δ* 161.7 (d, ^3^*J*_*CP*_ = 5.5 Hz, 10-C_q_), 139.8 (12-C_q_), 138.6 (10-C_q_), 138.5 (17-C_q_), 125.8 (18-CH), 125.8 (14-CH), 124.8 (16-CH), 124.4 (15-CH), 122.2 (13-CH), 56.5 (d, ^2^*J*_*CP*_ = 8.5 Hz, 2-H), 48.3 (d, ^1^*J*_*CP*_ = 146.0 Hz, 1-H), 44.3 (4-CH_2_), 23.9 (7-CH_2_), 21.0 (5-CH_2_ or 6-CH_2_), 21.0 (5-CH_2_ or 6-CH_2_). ^31^P NMR (162 MHz, DMSO-*d*_6_) *δ* 14.19. HRESIMS *m*/*z* 355.0803 [M + H–Cl]^+^ (355.0876 calcd for C_15_H_20_N_2_O_4_PS).

### Compound 5g-2, diastereomer 2

^1^H NMR (400 MHz, DMSO-*d*_6_) *δ* 8.93 (d, *J* = 9.8 Hz, 1H, 1-NH), 8.42 (s, 1H, 18-CH), 8.03 (dd, *J* = 6.7, 1.7 Hz, 1H, 13-H), 7.96 (dd, *J* = 7.6, 1.7 Hz, 1H, 6-H), 7.48 (ddd, *J* = 5.8, 5.8, 1.7 Hz, 1H, part C of ABCD, 14-H), 7.45 (ddd, *J* = 5.8, 5.8, 1.7 Hz, 1H, part D of ABCD, 15-H), 4.48 (ddd, ^3^*J*_*CP*_ = 17.8 Hz, *J* = 9.6, 8.0 Hz, 1H, 1-H), 3.48 (m, 1H, 2-H), 3.31 (m, 1H, 4-H), 2.87 (br. dd, *J* = 10.1, 10.1 Hz, 1H, 4-H), 2.23–2.13 (br. d, *J* = 12.0 Hz, 1H, 7-H), 1.86–1.55 (m, 4H, 7-H, 5′-H, 6′-H, 5′′-H or 6′′-H), 1.48 (m, 1H, 5′′-H or 6′′-H). ^13^C NMR (126 MHz, DMSO-*d*_6_) *δ* 161.8 (d,^3^*J*_*CP*_ = 3.8 Hz, 9-C_q_), 140.3 (12-C_q_), 139.2 (10-C_q_), 139.1 (17-C_q_), 126.3 (14-H), 126.2 (18-H), 125.2 (16-H), 125.0 (15-H), 122.8 (13-H), 56.3 (d, ^2^*J*_*CP*_ = 7.7 Hz, 2-CH), 49.4 (d, ^1^*J*_*CP*_ = 147.4 Hz, 1-CH), 44.8 (4-CH_2_), 26.2 (d, ^3^*J*_*CP*_ = 3.5 Hz, 7-CH_2_), 21.7 (5-CH_2_ or 6-CH_2_), 21.5 (5-CH_2_ or 6-CH_2_). ^31^P NMR (162 MHz, DMSO-*d*_6_) *δ* 14.41. HRESIMS *m*/*z* 355.0806 [M + H–Cl]^+^ (355.0876 calcd for C_15_H_20_N_2_O_4_PS).

### ((Benzo[*b*]thiophene-3-carboxamido)(piperidin-2-yl)met-hyl)phosphonic acid hydrochloride (5h)

The synthesis was performed according to general procedure B. Compound 4h-1 (49.4 mg, 0.0973 mmol) gave 5h-1 as a colorless solid (25.8 mg, 0.0660 mmol, 68%). Compound 4h-2 (39.3 mg, 0.0770 mmol) gave 5h-2 as a colorless solid (15.2 mg, 0.0389 mmol, 51%).

### Compound 5h-1, diastereomer 1

^1^H NMR (400 MHz, DMSO-*d*_6_) *δ* 8.70 (s, 1H, 11-H), 8.48 (d, *J* = 7.5 Hz, 1H, 17-H), 8.04 (dd, *J* = 7.5, 1.6 Hz, 1H, 13-H), 7.45 (ddd, *J* = 7.5, 7.5, 1.3 Hz, 1H, part C of ABCD, 16-H), 7.42 (ddd, *J* = 7.5, 7.5, 1.6 Hz, 1H, part D of ABCD, 15-H), 4.71 (ddd, ^2^*J*_HP_ = 19.5 Hz, *J* = 9.5 Hz, 4.8 Hz, 1H, 1-H), 3.46 (m, 1H, 2-H), 3.29 (br. d, *J* = 12.5 Hz, 1H, 4-H), 2.96 (ddd, *J* = 12.5, 12.5, 2.08 Hz, 1H, 4-H), 2.09 (br. d, *J* = 12.1 Hz, 1H), 1.81–1.38 (m, 5H, 5-H_2_, 6-H_2_, 7-H). ^13^C NMR (126 MHz, DMSO-*d*_6_) *δ* 163.5 (d, ^3^*J*_CP_ = 5.4 Hz, 9-Cq), 139.2 (13-Cq), 137.3 (18-Cq), 132.4 (11-CH), 130.0 (10-Cq), 124.9 (15-CH), 124.8 (16-CH), 124.6 (17-CH), 122.8 (14-CH), 57.2 (d, ^2^*J*_CP_ = 8.7 Hz, 2-CH), 48.2 (d, ^1^*J*_CP_ = 146.3 Hz, 1-CH), 44.8 (4-CH_2_), 24.5 (d, ^3^*J*_CP_ = 3.8 Hz, 7-CH_2_), 21.6 (5-CH_2_, 6-CH_2_). ^31^P NMR (162 MHz, DMSO-*d*_6_) *δ* 14.68. HRESIMS *m*/*z* 355.0807 [M + H–Cl]^+^ (355.0876 calcd for C_15_H_19_N_2_O_4_PS).

### Compound 5h-2, diastereomer 2

^1^H NMR (500 MHz, DMSO-*d*_6_) *δ* 8.75 (s, 1H, 11-H), 8.54 (d, *J* = 9.8 Hz, 1H, 1-NH), 8.47 (d, *J* = 7.2 Hz, 1H, 17-H), 8.06 (d, *J* = 7.0 Hz, 1H, 14-H), 7.46 (ddd, *J* = 7.3, 7.3, 1.2 Hz, 1H, part C of ABCD, 16-H), 7.45 (ddd, *J* = 7.3, 7.3, 1.2 Hz, 1H, part D of ABCD, 15-H), 4.56 (ddd, ^2^*J*_*HP*_ = 18.0 Hz, *J* = 9.1 Hz, 1H), 3.47 (m, 1H, 2-H), 3.30 (br. d, *J* = 12.0 Hz, 1H, 4-H), 2.87 (ddd, *J* = 11.9, 11.9, 11.9 Hz, 1H, 4-H), 2.23 (d, *J* = 13.3 Hz, 1H, 7-H), 1.85–1.42 (m, 5H, 5-H_2_, 6-H_2_, 7-H). ^13^C NMR (126 MHz, DMSO-*d*_6_) *δ* 163.0 (d, ^3^*J*_*CP*_ = 3.3 Hz), 139.3 (13-C_q_), 137.3 (18-C_q_), 132.2 (11-CH), 130.2 (10-C_q_), 124.9 (15-CH or 15-CH), 124.8 (15-CH or 15-CH), 124.6 (17-CH), 122.7 (14-CH), 56.2 (d, ^2^*J*_*CP*_ = 8.8 Hz, 2-H), 48.7 (d, ^1^*J*_*CP*_ = 148.0 Hz, 1-H), 44.7 (4-H_2_), 26.1 (7-CH_2_), 21.7 (5-CH_2_ or 6-CH_2_), 21.5 (5-CH_2_ or 6-CH_2_). ^31^P NMR (162 MHz, DMSO-*d*_6_) *δ* 14.94. HRESIMS *m*/*z* 355.0849 [M + H–Cl]^+^ (355.0876 calcd for C_15_H_20_N_2_O_4_PS).

### (Piperidin-2-yl(2-(thiophen-2-yl)acetamido)methyl)phos-phonic acid hydrochloride (5i)

The synthesis was performed according to general procedure B. Compound 4i-1 (60.8 mg, 0.128 mmol) gave 5i-1 as a colorless solid (21.3 mg, 0.0600 mmol, 47%). Compound 4i-2 (61.1 mg, 0.129 mmol) gave 5i-2 as a colorless solid (31.0 mg, 0.0874 mmol, 68%).

### Compound 5i-1, diastereomer 1

^1^H NMR (400 MHz, DMSO-*d*_6_) *δ* 8.36 (d, *J* = 9.8 Hz, 1H, 1-NH), 7.36 (dd, *J* = 4.9, 1.6 Hz, 1H, 13-H), 7.04–6.89 (m, 2H, 14-H, 15-H), 4.43 (ddd, ^3^*J*_*HP*_ = 20.1 Hz, *J* = 9.7, 4.5 Hz, 1H, 1-H), 3.90 (d, ^2^*J* = 16.1 Hz, 1H, part A of B, 10-H), 3.79 (d, ^2^*J* = 16.1 Hz, 1H, part B of AB, 10-H), 3.37 (m, 1H, 2-H), 3.24 (br. d, *J* = 12.4 Hz, 1H, 4-H), 2.93 (dd, *J* = 11.5, 11.5 Hz, 1H, 4-H), 2.01 (br. d, *J* = 12.6 Hz, 1H, 7-H), 1.81–1.37 (m, 5H, 5-H_2_, 6-H_2_, 7-H). ^13^C NMR (126 MHz, DMSO-*d*_6_) *δ* 170.1 (d, ^3^*J*_*CP*_ = 5.6 Hz, 9-C_q_), 137.2 (11-C_q_), 126.4 (14-CH), 126.2 (15-CH), 124.9 (13-CH), 56.9 (d, ^2^*J*_*CP*_ = 10.0 Hz, 2-CH), 48.1 (d, ^1^*J*_*CP*_ = 148.5 Hz, 1-CH), 44.8 (4-CH_2_), 36.00 (10-CH_2_), 24.0 (d, ^3^*J*_*CP*_ = 3.4 Hz, 7-CH_2_), 21.5 (5-CH_2_, 6-CH_2_). ^31^P NMR (162 MHz, DMSO-*d*_6_) *δ* 14.76. HRESIMS *m*/*z* 319.0848 [M + H–Cl]^+^ (319.0876 calcd for C_12_H_20_N_2_O_4_PS).

### Compound 5i-2, diastereomer 2

^1^H NMR (400 MHz, DMSO-*d*_6_) *δ* 8.49 (m, 1H, 1-NH), 7.35 (dd, *J* = 4.6, 1.7 Hz, 1H, 13-H), 6.97–6.92 (m, 2H, 14-H, 15-H), 4.22 (ddd, ^2^*J*_*HP*_ = 17.8 Hz, *J* = 9.6, 6.4 Hz, 1H, 1-H), 3.83 (s, 2H, 10-CH_2_), 3.35–3.21 (m, 2H, 2-H, 4-H), 2.87 (br. dd, *J* = 11.4, 11.4 Hz, 1H, 4-H), 2.00 (br. d, *J* = 13.2 Hz, 1H, 7-H), 1.79–1.39 (m, 5H, 5-H_2_, 6-H_2_, 7-H). ^13^C NMR (126 MHz, DMSO-*d*_6_) *δ* 169.5 (d, ^3^*J*_*CP*_ = 5.1 Hz, 9-C_q_), 137.2 (11-C_q_), 126.4 (14-CH), 126.3 (15-CH), 124.9 (13-CH), 56.8 (d, ^2^*J*_*CP*_ = 5.7 Hz, 2-CH), 48.4 (d, ^1^*J*_*CP*_ = 146.4 Hz, 1-CH), 44.9 (4-CH_2_), 36.1 (10-CH_2_), 25.8 (d, ^3^*J*_*CP*_ = 4.9 Hz, 7-CH_2_), 21.8 (5-CH_2_ or 6-CH_2_), 21.5 (5-CH_2_ or 6-CH_2_). ^31^P NMR (162 MHz, DMSO-*d*_6_) *δ* 15.26. HRESIMS *m*/*z* 319.0833 [M + H–Cl]^+^ (319.0876 calcd for C_12_H_20_N_2_O_4_PS).

### (Piperidin-2-yl(2-(thiophen-3-yl)acetamido)methyl)phos-phonic acid hydrochloride (5j)

The synthesis was performed according to general procedure B. Compound 4j-1 (48.0 mg, 0.101 mmol) gave 5j-1 as a colorless solid (24.4 mg, 0.0688 mmol, 68%). Compound 4j-2 (58.2 mg, 0.122 mmol) gave 5j-2 as a slightly yellow solid (26.8 mg, 0.0755, 62%).

### Compound 5j-1, diastereomer 1

^1^H NMR (400 MHz, DMSO-*d*_6_) *δ* 8.30 (dd, *J* = 9.9, 3.2 Hz, 1H, 1-NH), 7.44 (dd, *J* = 4.9, 2.9 Hz, 1H, 14-H), 7.27 (dd, *J* = 2.9, 1.2 Hz, 1H, 12-H), 7.07 (dd, *J* = 4.9, 1.2 Hz, 1H, 15-H), 4.46 (ddd, ^3^*J*_*HP*_ = 20.4 Hz, *J* = 9.9, 4.3 Hz, 1H, 1-H), 3.69 (d, ^2^*J* = 15.2 Hz, 1H, part A of AB, 10-H), 3.56 (d, ^2^*J* = 15.2 Hz, 1H, part B of AB, 10-H), 3.39 (m, 1H, 2-H), 3.24 (br. d, *J* = 12.3 Hz, 1H, 4-H), 2.93 (m, 1H, 4-H), 2.00 (br. d, *J* = 13.5 Hz, 1H, 7-H), 1.81–1.35 (m, 4H, 5-H_2_, 6-H_2_, 7-H). ^13^C NMR (126 MHz, DMSO-*d*_6_) *δ* 170.8 (d, ^3^*J*_*CP*_ = 5.5 Hz, 9-C_q_), 136.0 (11-C_q_), 129.0 (15-CH), 125.4 (14-CH), 122.3 (12-CH), 56.9 (d, ^2^*J*_*CP*_ = 10.3 Hz, 2-CH), 48.0 (d, ^1^*J*_*CP*_ = 148.9 Hz, 1-CH), 44.8 (4-CH_2_), 36.7 (10-CH_2_), 24.0 (d, ^3^*J*_*CP*_ = 3.3 Hz, 7-CH_2_), 21.5 (5-CH_2_ or 6-CH_2_), 21.4 (5-CH_2_ or 6-CH_2_). ^31^P NMR (162 MHz, DMSO-*d*_6_) *δ* 15.07. ^15^N NMR (51 MHz, DMSO-*d*_6_) *δ* −272.06 (1-NH), −331.80 (2-NH_2_). HRESIMS *m*/*z* 319.0801 [M + H–Cl]^+^ (319.0876 calcd for C_12_H_20_N_2_O_4_PS).

### Compound 5j-2, diastereomer 2

^1^H NMR (400 MHz, DMSO-*d*_6_) *δ* 8.40 (d, *J* = 7.1 Hz, 1H, 1-NH), 7.44 (dd, *J* = 4.9, 3.0 Hz, 1H, 14-H), 7.30 (d, *J* = 3.0 Hz, 1H, 12-H), 7.08 (d, *J* = 4.9 Hz, 1H, 15-H), 4.27 (ddd, ^2^*J*_*HP*_ = 17.3 Hz, *J* = 9.7, 7.1 Hz, 1H), 3.60 (s, 2H, 10-H_2_), 3.38–3.22 (m, 2H, 2-H, 4-H), 2.89 (dd, *J* = 12.1, 12.1 Hz, 1H, 4-H), 2.06 (br. d, *J* = 13.8 Hz, 1H, 7-H), 1.78–1.36 (m, 5H, 5-H_2_, 6-H_2_, 7-H). ^13^C NMR (126 MHz, DMSO-*d*_6_) *δ* 169.5 (d, *J* = 4.4 Hz, 9-C_q_), 135.3 (11-C_q_), 128.5 (15-CH), 124.8 (14-CH), 121.8 (12-CH), 55.9 (d, ^2^*J*_*CP*_ = 6.8 Hz, 2-CH), 47.7 (d, ^1^*J*_*CP*_ = 148.3 Hz, 1-CH), 44.2 (4-CH_2_), 36.3 (10-CH_2_), 25.3 (d, ^3^*J*_*CP*_ = 4.1 Hz, 7-CH_2_), 21.2 (5-CH_2_ or 6-CH_2_), 20.9 (5-CH_2_ or 6-CH_2_). ^31^P NMR (162 MHz, DMSO-*d*_6_) *δ* 15.26. HRESIMS *m*/*z* 319.0885 [M + H–Cl]^+^ (319.0876 calcd for C_12_H_20_N_2_O_4_PS).

### ((2-(Benzo[*b*]thiophen-2-yl)acetamido)(piperidin-2-yl)methyl)phosphonic acid hydrochloride (5k)

The synthesis was performed according to general procedure B. Compound 4k-1 (52.2 mg, 0.100 mmol) gave 5k-1 as a slightly orange solid (17.5 mg, 0.0432 mmol, 43%). Compound 4k-2 (53.7 mg, 0.102 mmol) gave 5k-2 as a colorless solid (13.4 mg, 0.0331 mmol, 32%).

### Compound 5k-1, diastereomer 1

^1^H NMR (400 MHz, DMSO-*d*_6_) *δ* 8.45 (d, *J* = 9.3 Hz, 1H, 1-NH), 7.87 (d, *J* = 7.7 Hz, 1H, 14-H), 7.80–7.69 (dd, *J* = 7.8, 1.0 Hz, 1H, 17-H), 7.32 (ddd, *J* = 7.4, 7.4, 1.3 Hz, 1H, part C of ABCD, 16-H), 7.28 (ddd, *J* = 7.4, 7.4, 1.3 Hz, 1H, part D of ABCD, 15-H), 7.25 (s, 1H, 19-H), 4.39 (ddd, ^2^*J*_*HP*_ = 19.5 Hz, *J* = 9.7, 4.9 Hz, 1H, 1-H), 4.01 (d, ^2^*J* = 16.0 Hz, 1H, part A of AB, 10-H), 3.90 (d, ^2^*J* = 16.0 Hz, 1H, part B of AB,10-H), 3.35 (m, 1H, 2-H), 3.25 (br. d, *J* = 12.4 Hz, 1H, 4-H), 2.92 (br. dd, *J* = 11.7, 11.7 Hz, 1H, 4-H), 2.02 (br. d, *J* = 13.1 Hz, 1H, 7-H), 1.80–1.35 (m, 5H, 5-H_2_, 6-H_2_, 7-H). ^13^C NMR (126 MHz, DMSO-*d*_6_) *δ* 169.6 (d, ^3^*J*_*CP*_ = 5.8 Hz, 9-C_q_), 139.4 (13-C_q_), 139.3 (18-C_q_), 138.9 (11-C_q_), 124.1 (16-CH), 123.7 (15-CH), 122.9 (17-CH), 122.7 (19-CH), 122.1 (14-CH), 57.0 (d, ^2^*J*_*CP*_ = 8.7 Hz, 2-H), 48.2 (d, ^1^*J*_*CP*_ = 146.6 Hz, 1-CH), 44.7 (4-CH_2_), 36.9 (10-CH_2_), 24.2 (d, ^3^*J*_*CP*_ = 3.8 Hz, 7-CH_2_), 21.6 (5-CH_2_ or 6-CH_2_), 21.5 (5-CH_2_ or 6-CH_2_). ^31^P NMR (162 MHz, DMSO-*d*_6_) *δ* 14.27. HRESIMS *m*/*z* 369.1008 [M + H–Cl]^+^ (369.1032 calcd for C_16_H_22_N_2_O_4_PS).

### Compound 5k-2, diastereomer 2

^1^H NMR (500 MHz, DMSO-*d*_6_) *δ* 8.57 (d, *J* = 10.0 Hz, 1H, 1-NH), 7.88 (d, *J* = 7.8 Hz, 1H, 14-H), 7.75 (d, *J* = 7.7 Hz, 1H, 17-H), 7.33 (dd, *J* = 7.4, 7.4 Hz, 1H, 16-H), 7.29 (dd, *J* = 7.4, 7.4 Hz, 1H, 15-H), 7.27 (s, 1H, 19-H), 4.29 (ddd, ^2^*J*_*HP*_ = 17.3 Hz, *J* = 9.6, 7.4 Hz, 1H, 1-H), 3.97 (d, ^2^*J* = 16.2 Hz, 1H, part A of AB, 10-H), 3.93 (d, ^2^*J* = 16.2 Hz, 1H, part A of AB, 10-H), 3.34–3.25 (m, 2H, 2-H, 4-H), 2.88 (br. dd, *J* = 12.2, 11.5 Hz, 1H, 4-H), 2.11 (br. d, *J* = 13.9 Hz, 1H, 7-H), 1.85–1.40 (m, 5H, 5-H_2_, 6-H_2_, 7-H). ^13^C NMR (126 MHz, DMSO-*d*_6_) *δ* 168.9 (d, ^3^*J*_*CP*_ = 4.2 Hz, 9-C_q_), 139.5 (13-C_q_ or 18-C_q_), 139.4 (18-C_q_ or 13-C_q_), 138.8 (11-C_q_), 124.2 (16-CH), 123.8 (15-CH), 123.0 (17-CH), 122.9 (19-CH), 122.2 (14-CH), 56.2 (d, ^2^*J*_*CP*_ = 7.6 Hz, 2-CH), 48.5 (d, ^1^*J*_*CP*_ = 148.5 Hz, 1-CH), 44.7 (4-CH_2_), 37.2 (10-CH_2_), 25.9 (7-CH_2_), 21.7 (5-CH_2_ or 6-CH_2_), 21.5 (5-CH_2_ or 6-CH_2_). ^31^P NMR (162 MHz, DMSO-*d*_6_) *δ* 13.32. HRESIMS *m*/*z* 369.1052 [M + H–Cl]^+^ (369.1032 calcd for C_16_H_22_N_2_O_4_PS).

### ((2-(Benzo[*b*]thiophen-3-yl)acetamido)(piperidin-2-yl)methyl)phosphonic acid hydrochloride (5l)

The synthesis was performed according to general procedure B. Compound 4l-1 (53.0 mg, 0.101 mmol) gave 5l-1 as a colorless solid (7.9 mg, 0.0195 mmol, 19%). Compound 4l-2 (50.7 mg, 0.0966 mmol) gave 5l-2 as a colorless solid (15.9 mg, 0.0393 mmol, 41%).

### Compound 5l-1, diastereomer 1

^1^H NMR (400 MHz, DMSO-*d*_6_) *δ* 8.49 (d, *J* = 9.5 Hz, 1H, 1-NH), 7.97 (d, *J* = 6.7 Hz, 1H, 15-H), 7.88 (d, *J* = 6.9 Hz, 1H, 18-H), 7.54 (s, 1H, 12-H), 7.39 (m, 1H, 17-H), 7.36 (m, 1H, 16-H), 4.46 (m, 1H, 1-H), 3.93 (d, ^2^*J* = 15.6 Hz, 1H, part A of AB, 10-H), 3.83 (d, ^2^*J* = 15.6 Hz, 1H, part B of AB, 10-H), 3.39 (m, 1H, 2-H), 3.25 (br. d, *J* = 12.1 Hz, 1H, 4-H), 2.93 (br. dd, *J* = 12.6, 12.6 Hz, 1H, 4-H), 2.03 (br. d, *J* = 13.0 Hz, 1H, 7-H), 1.86–1.35 (m, 5H, 5-H_2_, 6-H_2_, 7-H). ^13^C NMR (126 MHz, DMSO-*d*_6_) *δ* 170.4 (d, ^3^*J*_*CP*_ = 5.5 Hz, 9-C_q_), 139.3 (14-C_q_), 138.9 (19-C_q_), 130.6 (11-C_q_), 124.3 (16-CH), 124.1 (12-CH), 124.0 (17-CH), 122.7 (15-CH), 122.4 (18-CH), 56.9 (d, ^2^*J*_*CP*_ = 9.9 Hz, 2-CH), 48.1 (d, ^1^*J*_*CP*_ = 148.4 Hz, 1-CH), 44.8 (4-CH_2_), 35.1 (10-CH_2_), 24.1 (d, ^3^*J*_*CP*_ = 2.5 Hz, 7-CH_2_), 21.5 (5-CH_2_ or 6-CH_2_), 21.5 (5-CH_2_ or 6-CH_2_). ^31^P NMR (162 MHz, DMSO-*d*_6_) *δ* 14.97. HRESIMS *m*/*z* 3 690 859 [M + H–Cl]^+^ (369.1032 calcd for C_16_H_22_N_2_O_4_PS).

### Compound 5l-2, diastereomer 2

^1^H NMR (400 MHz, DMSO-*d*_6_) *δ* 8.56 (d, *J* = 9.3 Hz, 1H, 1-NH), 7.96 (m, 1H, 15-H), 7.90 (m, 1H, 18-H), 7.57 (s, 1H, 12-H), 7.38 (m, 1H, 17-H), 7.36 (m, 1H, 16-H), 4.28 (ddd, ^3^*J*_*HP*_ = 17.1 Hz, *J* = 9.7, 6.7 Hz, 1H, 1-H), 3.88 (d, ^2^*J* = 16.3 Hz, 1H, part A of AB, 10-H), 3.86 (dd, *J* = 16.3 Hz, 1H, part B of AB, 10-H), 3.36–3.22 (m, 2H, 2-H, 4-H), 2.88 (dd, *J* = 11.8, 11.8 Hz, 1H, 4-H), 2.04 (d, *J* = 13.8 Hz, 1H, 7-H), 1.82–1.38 (m, 5H, 5-H_2_, 6-H_2_, 7-H). ^13^C NMR (126 MHz, DMSO-*d*_6_) *δ* 169.8 (d, ^3^*J*_*CP*_ = 4.7 Hz, 9-C_q_), 139.3 (14-C_q_), 138.9 (19-C_q_), 130.5 (11-C_q_), 124.3 (16-CH), 124.3 (12-CH), 123.9 (17-CH), 122.7 (15-CH), 122.4 (18-CH), 56.7 (d, ^2^*J*_*CP*_ = 6.4 Hz, 2-CH), 48.4 (d, ^1^*J*_*CP*_ = 147.5 Hz, 1-CH), 44.9 (4-CH_2_), 35.2 (10-CH_2_), 25.8 (d, ^3^*J*_*CP*_ = 4.3 Hz, 7-CH_2_), 21.8 (5-CH_2_ or 6-CH_2_), 21.5 (5-CH_2_ or 6-CH_2_). ^31^P NMR (162 MHz, DMSO-*d*_6_) *δ* 15.13. HRESIMS *m*/*z* 369.0975 [M + H–Cl]^+^ (369.10324 calcd for C_16_H_22_N_2_O_4_PS).

### ^1^H,^15^N HSQC titration experiments

For the titrations, ^15^N-labelled NDM-1 and ^15^N-labelled VIM-2 (250 μM), and titrated ligands (0.5 equiv. in 1 μL) were prepared in the same buffer as the protein (20 mM KPO_4_, 0.1 mM ZnCl_2_, pH 7.0 with 10% D_2_O and 2.5% DMSO-*d*_6_). ^1^H,^15^N HSQC spectra were acquired with 128 × 1024 complex points (F1 × F2) and spectral width of 9090 × 2740 Hz on ^15^N-labeled NDM-1/VIM-2 and with every titration step up to 1 : 16 ratio between protein : ligand (0, 0.5, 1, 2, 4, 8, and 16 equiv. of ligand). All experiments were recorded on a Bruker 600 MHz spectrometer at 310 K equipped with a 5 mm TCI cryogenic probe. The NMR data were processed on the MestReNova software with the Mnova Binding plugin. The weighted average chemical shift perturbations (CSP) for the backbone amides were calculated from the observed chemical shift differences in the proton and nitrogen dimensions using the equation (chemical shift scaling factors: *F*_H_ = 1, *F*_N_ = 0.156) CSP = Δ*δ*_1H,15N_ = 
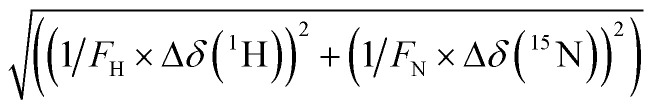
.

### Molecular docking

Schrödinger software (Schrödinger, LLC, New York, NY, 2019) was used and unless otherwise stated, default settings were used in the computations. The crystal structures of VIM-2 (pdb 6O5T) and NDM-1 (pdb 6NY7) were prepared using the Protein Preparation Wizard;^60^ involving Prime^52^ to fill in missing side chains and loops, Epik^61,62^ to generate heteroatom states (pH 7.0 ± 2.0), PropKa (pH 7) to optimize the hydrogen bond network (sampling of water orientations when appropriate), and the OPLS3e force field^63^ in the restrained minimization (hydrogens only). The Receptor Grid Generation module in Glide^64–66^ was used to define the active site by creating a rectangular box centered on the native ligand (crystal structure) and extending in all directions to encompass ligands of similar size (*i.e.* 16.5 Å) or ligands <22 Å. Grids with no water molecules in the active site were generated and validated. The ligands were converted to 3D all atom structures using LigPrep. Epik was used to generate possible ionization states at target pH 7 ± 2, including metal binding states. Tautomers were selected to be generated. The molecular docking was performed using Glide. The inhibitors were docked using the flexible docking mode, with sampling of nitrogen inversions and ring conformations, and with extra precision (XP) rescoring. Biased sampling of torsions was selected for amides by penalizing non-planar conformations. Epik state penalties was selected to be added to the docking score. A maximum of 40 poses were selected to be generated for each ligand. Post-docking minimization was used, and per-residue interaction scores were written for residues within 10 Å from the grid center. Prime MM-GBSA rescoring of docking poses (VSGB 2.0 solvation model and OPLS3e force field) were performed providing poses ranked based on free energy of binding (Δ*G*_bind_ in kcal mol^−1^). For these calculations, flexibility was allowed for residues within 10 Å from the ligands, and minimization was selected as sampling method. Structural interaction fingerprints (SIFts) in Canvas was used for representation and analysis of the docking poses. Side chain, zinc ion, and hydrogen bonding interactions were used to generate the SIFts. The similarity between the SIFts were evaluated using the Tanimoto similarity metric, and clustering was performed using the Flexible Beta linkage method. Epik (Schrödinger, LLC, New York, NY, 2020) was used for empirical predictions of aqueous p*K*_a_ values.

### Dose rate inhibition studies for IC_50_ determination

The half-maximal inhibitory concentration (IC_50_) against the NDM-1, VIM-2 and GIM-1 enzymes were determined by using twelve different concentration of inhibitor compounds ranging from 0 μM to 800 μM. A 100 μl solution with buffer (50 mM HEPES (pH 7.2), 100 μM ZnCl_2_, 2.5% DMSO), purified enzyme (0.1 nM VIM-2, 10 nM NDM-1 or 1 nM GIM-1) and 800–0 μM inhibitor was incubated in a 96 well plate at 25 °C for 5 min. In addition, the enzyme buffer contained 400 μg ml^−1^ bovine serum albumin (BSA) to prevent protein unfolding and loss of activity due to low concentrations.^67,68^ 100 μM of the reporter substrate nitrocefin (VIM-2, GIM-1) or imipenem (NDM-1) was added to the enzyme–inhibitor solution and the increase in absorbance at 482 nm (nitrocefin) or 300 nm (imipenem) was recorded on a Spectramax M4 spectrophotometer (Molecular Devices). Each data point was performed in triplicates and the initial velocity for each inhibitor concentration was analyzed by a log [inhibitor] *vs.* response curve fitting to calculate IC_50_ in GraphPad Prism 9.2 software.

### Cytotoxicity assay

MCF-7 and HepG2 cells were cultured in Dulbecco's modified Eagle's medium supplemented with 10% fetal bovine serum and 1% of penicillin–streptomycin antibiotics (Sigma-Aldrich). Cells were cultured in a permanent exponential growth at 37 °C, 5% CO_2_.^69^ Briefly, cells were seeded in 96-well plates (TPP® tissue culture plates 92097) at a density that allowed continued exponential growth. Stock solutions of the compounds dissolved in DMSO were added to a final DMSO concentration of 0.3% v/v in the cell culture medium. Two-fold serial dilutions were made to estimate EC_50_. After 24 h of incubation in the presence of the compounds, cell viability was determined using PrestoBlue cell viability reagent (ThermoFisher). POLARstar Omega plate reader (BMG Lab Tech) was used to measure resorufin fluorescence (544 nm/590 nm). Cells with no treatment, cells treated only with DMSO 0.3% v/v, and cells treated with SDS 1% w/v were used as controls. Three independent replicate experiments were used to calculate the EC_50_. The statistical analysis and plots were performed using R version 3.6.3 (R core team).^70^ The viability of the cells was plotted using ggplot 2,^71^ as the fluorescence of treated cells/fluorescence of the lowest concentration of the compound.

### Protein expression

The expression and purification of uniformly [^15^N]-labelled NDM-1 and VIM-2 were performed according to the procedure reported previously.^48,49^ For enzymatic assays, NDM-1, VIM-2 and GIM-1 were expressed and purified as published earlier.^41^

### Molecular cloning

The full-length *bla*_VIM-2_ gene was amplified using primers ATA CAT ATG TTC AAA CTT TTG AGT AAG TTA TTG and TCA CTC GAG CTA CTC AAC GAC TGA GCG ATT TG and subcloned into a pET30a vector between the NdeI and XhoI restriction sites resulting in plasmid pY339.

### Preparation of outer membrane vesicles (OMVs) and *in situ* activity testing

OMVs carrying active VIM-2 inside their lumen were prepared as described.^72^ Briefly, *E. coli* strain BL21(DE3)ΔompA carrying plasmid pY339 was grown at 37 °C in LB media supplemented with 10 μM ZnCl_2_ and 50 μg mL^−1^ Kanamycin. When cultures reached an optical density of 0.4 protein expression was induced by addition of 50 μM IPTG and growth was maintained for 2 h. Cells were removed by centrifugation at 10 000 × *g* for 10 min and cleared supernatants were filtered using 0.45 μm bottle top filters. OMVs were collected by centrifugation at 38 400 × *g* for 2 h, washed in OMV buffer (DPBSS, Sigma-Aldrich D8662, supplemented with 10 μM ZnCl_2_), filtered using 0.45 μm syringe filters, recollected by centrifugation at 74 500 × *g* for 30 min and resuspended in OMV buffer.

Activity of the encapsulated VIM-2 in presence of the compounds was determined in clear flat bottom half area 96 well microplates (Corning 3696) in a total volume of 50 μL containing 400 μg ml^−1^ OMVs, 250 μg ml^−1^ CENTA β-lactamase substrate (Sigma-Aldrich 219475), and 2.5% DMSO in OMV buffer using a Fluostar Omega plate reader (BMG Lab Tech) by measuring the increase in absorbance at 405 nm 10 minutes after addition of the substrate.

## Author contributions

KP and FD contributed equally. FD and KP synthesized the molecules, KP performed the NMR titrations and computational docking, SS and HKSL determined IC_50_s, GPDSM and PS performed the cytotoxicity assay, JT determined membrane permeability, AAR expressed and purified proteins, and AV determined X-ray structures. HA, KP and ME designed the structures. The synthesis was developed by HA, KP and FD. PS, HKSL, KP, HA and ME analysed and discussed the data. The manuscript was written with the contributions of all authors, and all authors have approved the final version of the manuscript.

## Conflicts of interest

There are no conflicts to declare.

## Supplementary Material

MD-014-D3MD00286A-s001

MD-014-D3MD00286A-s002
