# Supplementary material for: α-Aminophosphonate inhibitors of metallo-β-lactamases NDM-1 and VIM-2
Source: RSC Med Chem. 2023 Aug 2;14(11):2277–300. doi: 10.1039/d3md00286a (PMC10650955; doi:10.1039/d3md00286a)
Supplement: MD-014-D3MD00286A-s001 [file MD-014-D3MD00286A-s001.pdf]

Electronic Supplementary Material (ESI) for RSC Medicinal Chemistry.
This journal is © The Royal Society of Chemistry 2023

Electronic Supplementary Material

α-Aminophosphonate inhibitors of metallo-β-lactamases NDM-1 and VIM-2

Katarzyna Palica,[‡a] Fritz Deufel,[‡a] Susann Skagseth,[b] Gabriela Paula Di Santo Metzler,[c,d] Johannes Thoma,[c] Anna Andersson Rasmussen,[a] Arto Valkonen,[e] Per Sunnerhagen,[c] Hanna-Kirsti S. Leiros,[b] Hanna Andersson,[*a] and Mate Erdelyi[*a]

[†] Department of Chemistry - BMC, Organic Chemistry; Uppsala University, Husargatan 3, 752 37 Uppsala, Sweden

[‡] Department of Chemistry, Faculty of Science and Technology, UiT The Arctic University of Norway, N-9037 Tromsø, Norway

[§] Department of Chemistry & Molecular Biology, University of Gothenburg, Medicinaregatan 9C, 413 90 Göteborg, Sweden

[¶] Center for Antibiotics Resistance Research (CARe) at University of Gothenburg, Medicinaregatan 9C, 413 90 Göteborg, Sweden

[Δ] Department of Chemistry, University of Jyvaskyla, Survontie 9B, 40014 University of Jyväskylä, Finland

* E-mail: mate.erdelyi@kemi.uu.se, hanna.andersson@redglead.com

Table of contents

1. Dose-response graphs S2
2. Cytotoxicity assay S14
3. Protein sequence alignment[1] S18
4. NMR spectra of the synthesized compounds S19
5. Crystallographic data S197
6. Chemical shift perturbation throughout the titration of NDM-1 and of VIM-2 with inhibitors S209
7. Chemical shift perturbation histograms S230
8. Estimation of binding constants (K_d) S243
9. Linear correlation of chemical shift perturbation S245
10. Evaluation of the binding energies of the docking poses S257
11. The RMSD of atomic positions of the binding energies of the docking poses S260
12. SDS-Page Analysis for ^{15}N-labelled NDM-1 and VIM-2 .... S261
13. Long term stability S264
14. References S268

1. Dose-response graphs

Table S1. The dose-response graphs for the enzymatic assay of the inhibitors against NDM-1, GIM-1 and VIM-2. For details, see the experimental section in the main text.

Inhibitors	NDM-1	GIM-1	VIM-2
5a-1	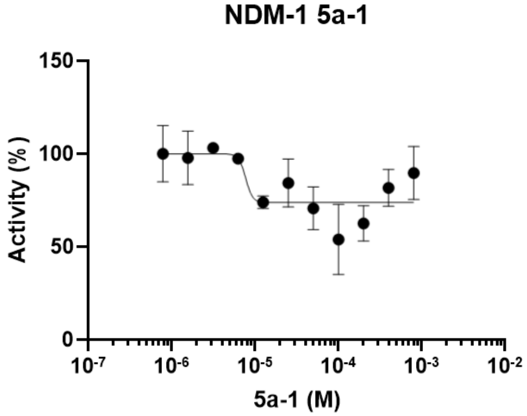	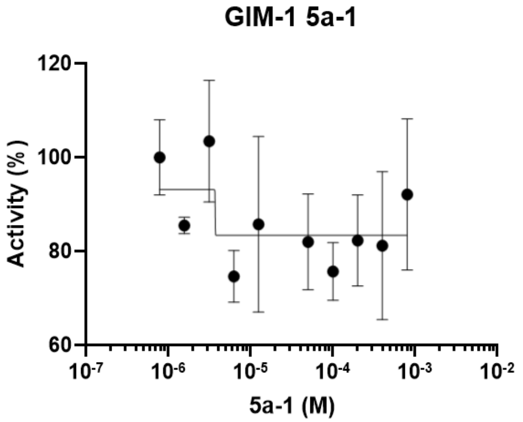	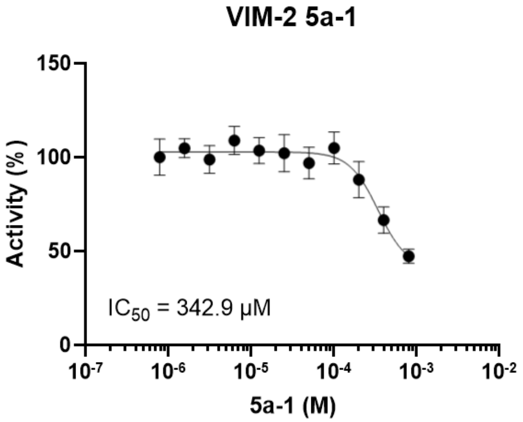
5a-2	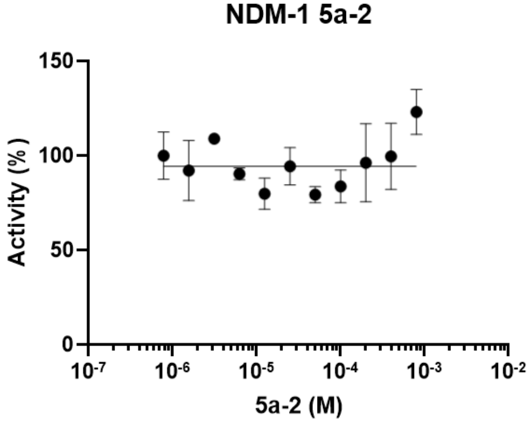	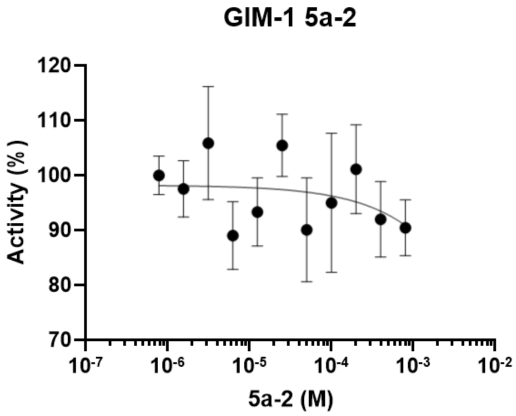	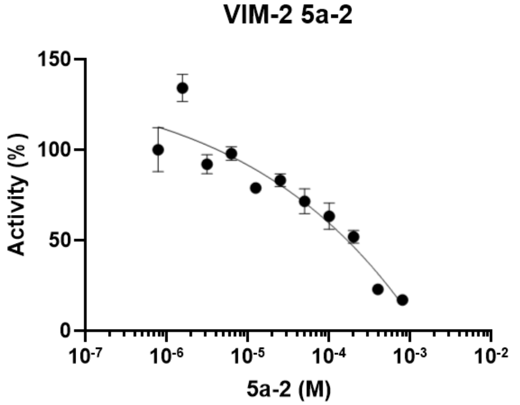

5b-1

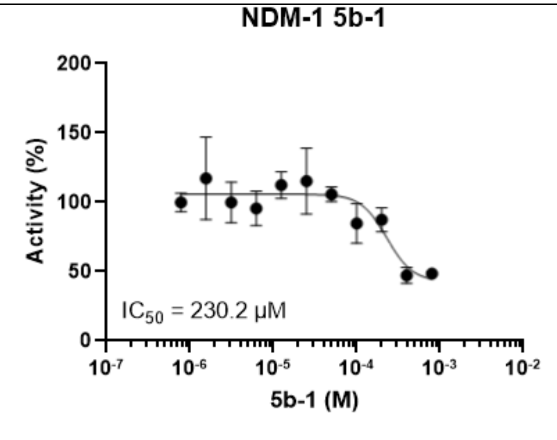
NDM-1 5b-1
Activity (%)
IC_{50} = 230.2 μM
5b-1 (M)

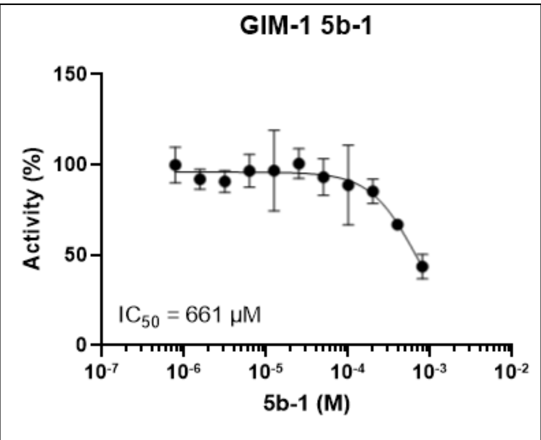
GIM-1 5b-1
Activity (%)
IC_{50} = 661 μM
5b-1 (M)

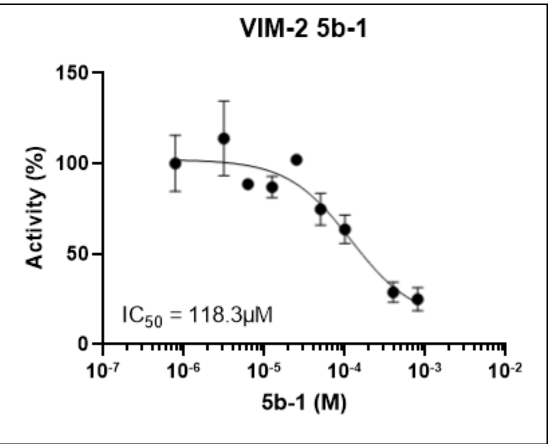
VIM-2 5b-1
Activity (%)
IC_{50} = 118.3μM
5b-1 (M)

5b-2

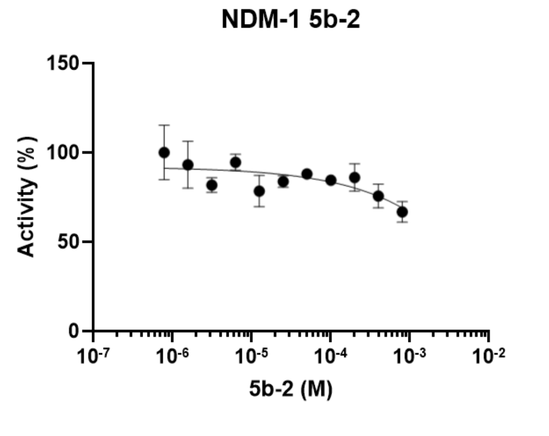
NDM-1 5b-2
Activity (%)
5b-2 (M)

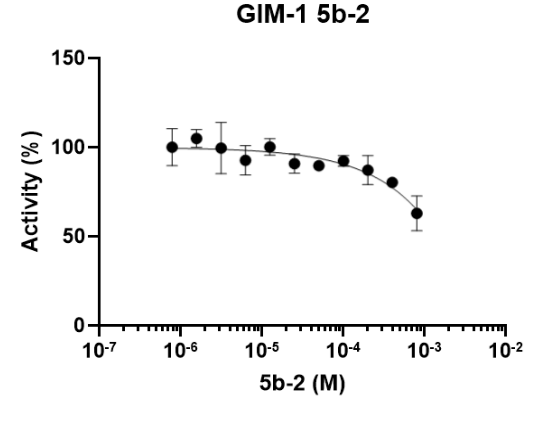
GIM-1 5b-2
Activity (%)
5b-2 (M)

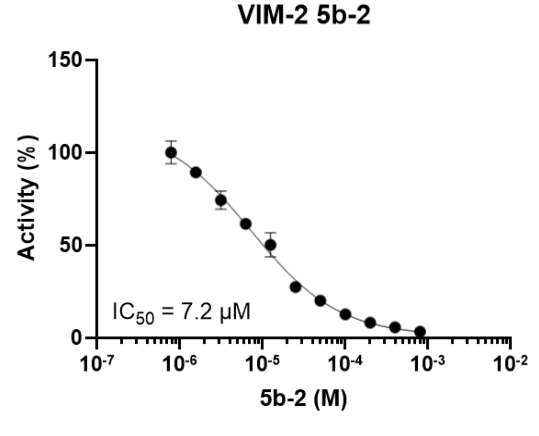
VIM-2 5b-2
Activity (%)
IC_{50} = 7.2 μM
5b-2 (M)

**5c-1**	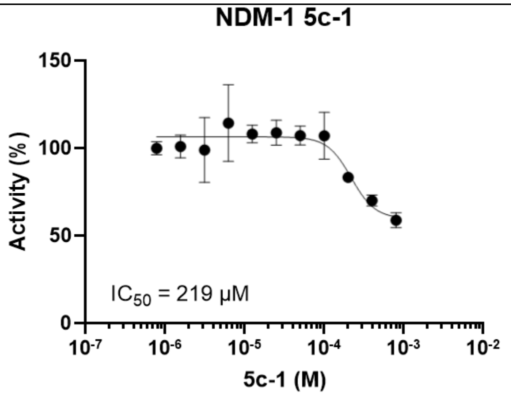NDM-1 5c-1<br>Activity (%)<br>IC50 = 219 µM<br>5c-1 (M)	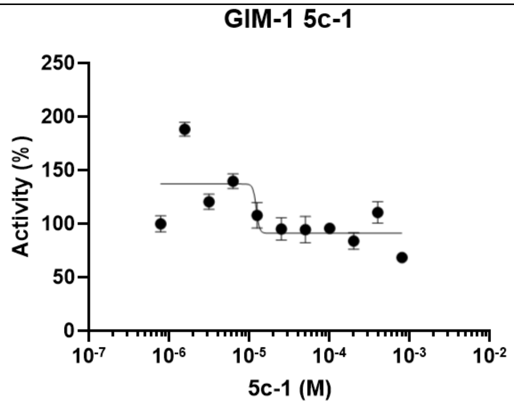GIM-1 5c-1<br>Activity (%)<br>5c-1 (M)	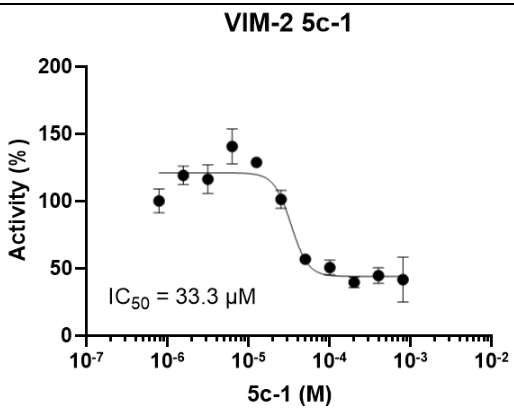VIM-2 5c-1<br>Activity (%)<br>IC50 = 33.3 µM<br>5c-1 (M)
**5c-2**	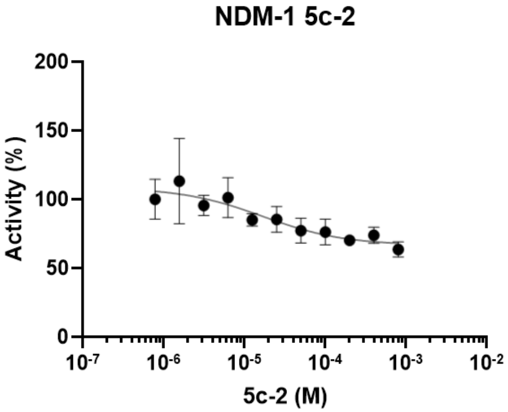NDM-1 5c-2<br>Activity (%)<br>5c-2 (M)	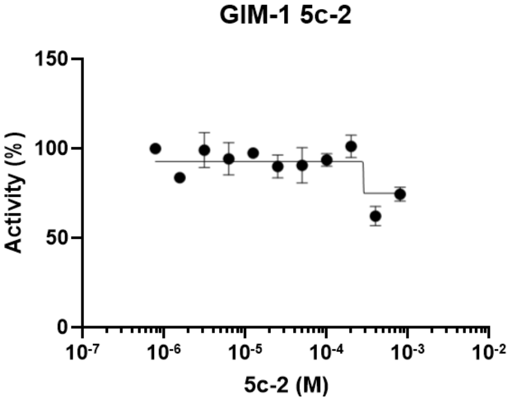GIM-1 5c-2<br>Activity (%)<br>5c-2 (M)	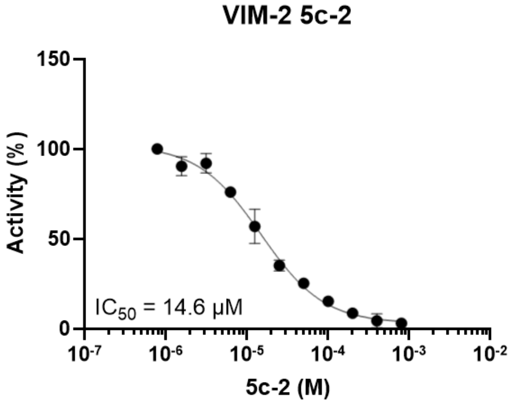VIM-2 5c-2<br>Activity (%)<br>IC50 = 14.6 µM<br>5c-2 (M)

5d-1

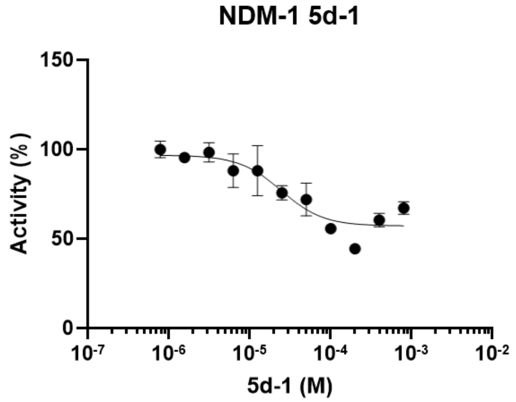
NDM-1 5d-1
Activity (%)
5d-1 (M)

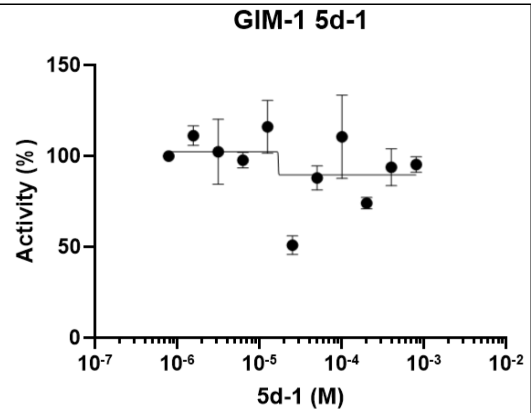
GIM-1 5d-1
Activity (%)
5d-1 (M)

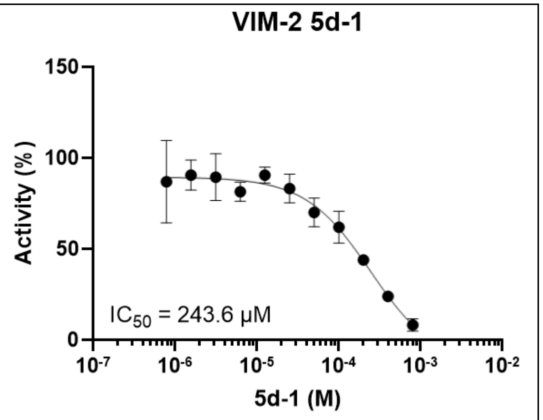
VIM-2 5d-1
Activity (%)
IC_{50} = 243.6 μM
5d-1 (M)

5d-2

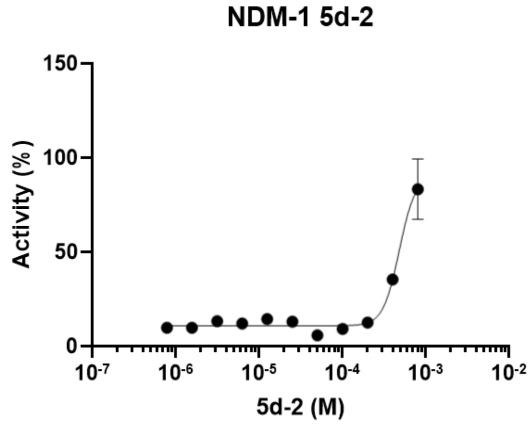
NDM-1 5d-2
Activity (%)
5d-2 (M)

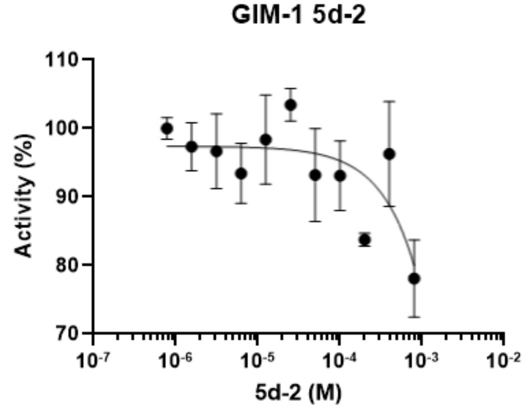
GIM-1 5d-2
Activity (%)
5d-2 (M)

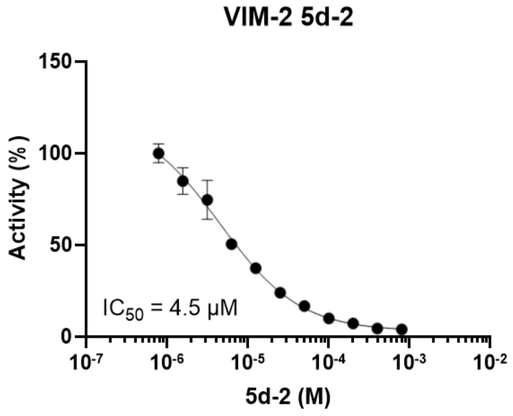
VIM-2 5d-2
Activity (%)
IC_{50} = 4.5 μM
5d-2 (M)

**5e-1**	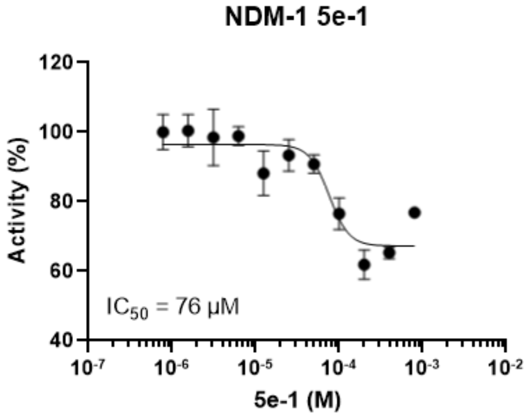 NDM-1 5e-1<br>Activity (%)<br>5e-1 (M)<br>IC50 = 76 μM	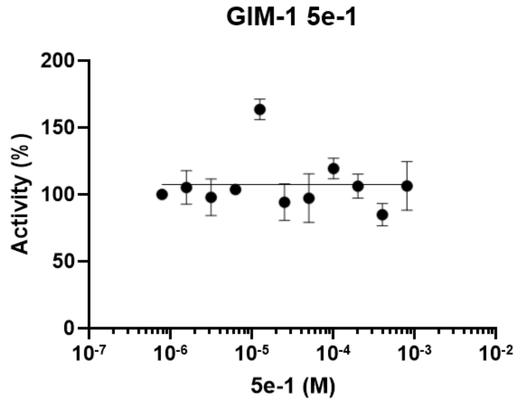 GIM-1 5e-1<br>Activity (%)<br>5e-1 (M)	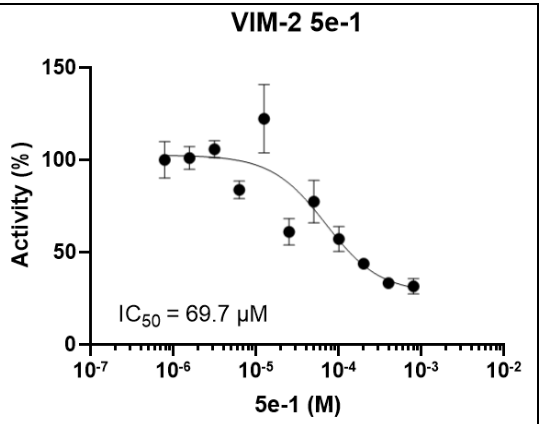 VIM-2 5e-1<br>Activity (%)<br>5e-1 (M)<br>IC50 = 69.7 μM
**5e-2**	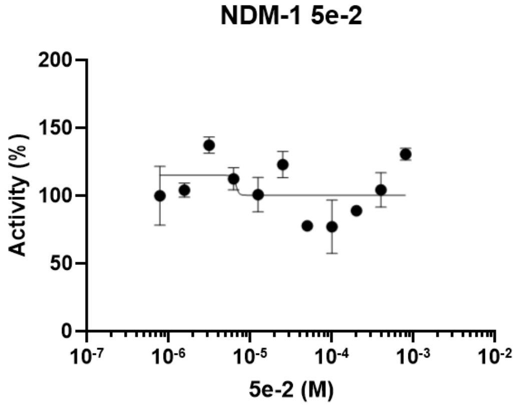 NDM-1 5e-2<br>Activity (%)<br>5e-2 (M)	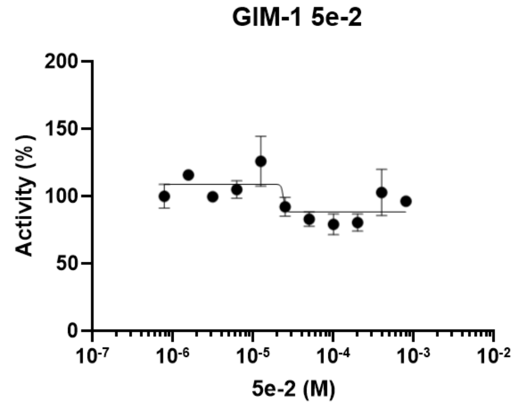 GIM-1 5e-2<br>Activity (%)<br>5e-2 (M)	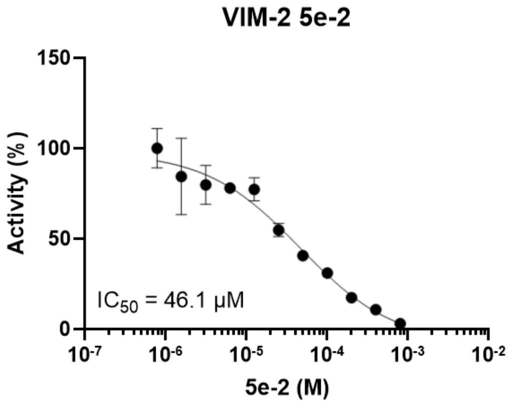 VIM-2 5e-2<br>Activity (%)<br>5e-2 (M)<br>IC50 = 46.1 μM

**5f-1**	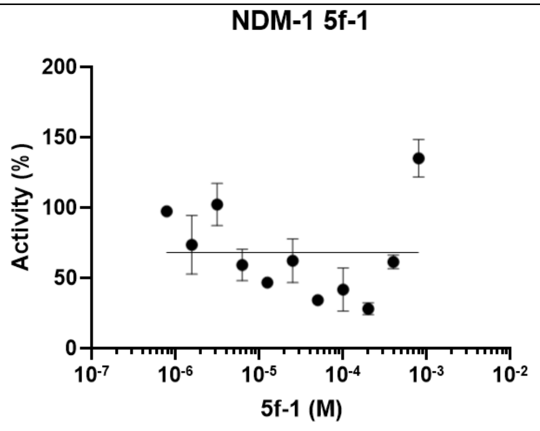 NDM-1 5f-1<br>Activity (%)<br>5f-1 (M)	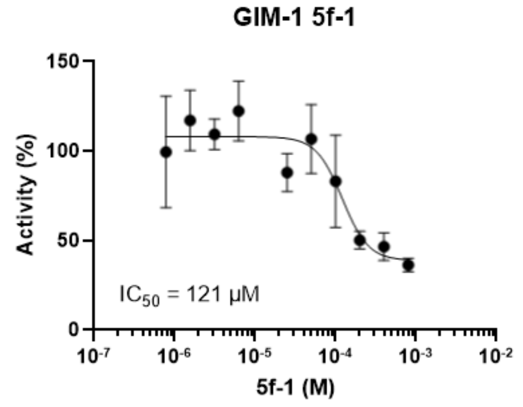 GIM-1 5f-1<br>Activity (%)<br>IC_{50} = 121 μM<br>5f-1 (M)	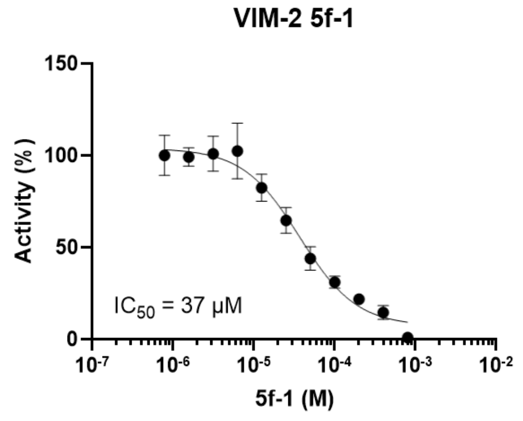 VIM-2 5f-1<br>Activity (%)<br>IC_{50} = 37 μM<br>5f-1 (M)
**5f-2**	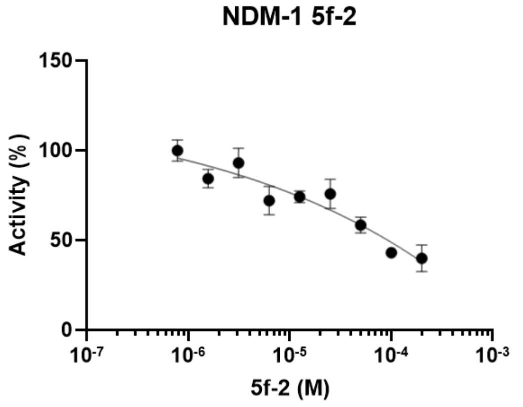 NDM-1 5f-2<br>Activity (%)<br>5f-2 (M)	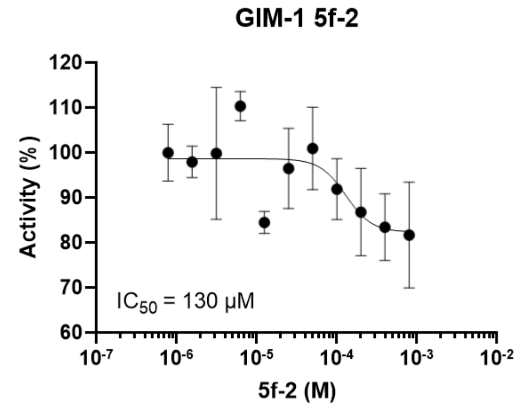 GIM-1 5f-2<br>Activity (%)<br>IC_{50} = 130 μM<br>5f-2 (M)	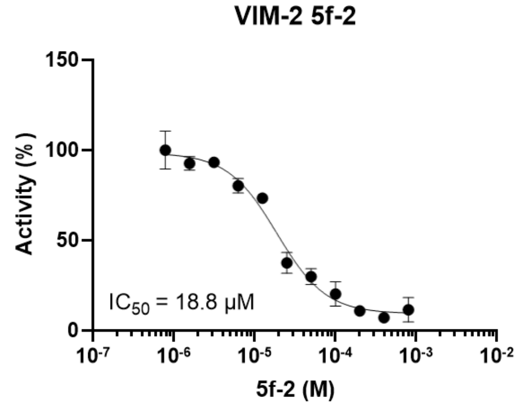 VIM-2 5f-2<br>Activity (%)<br>IC_{50} = 18.8 μM<br>5f-2 (M)

5g-1

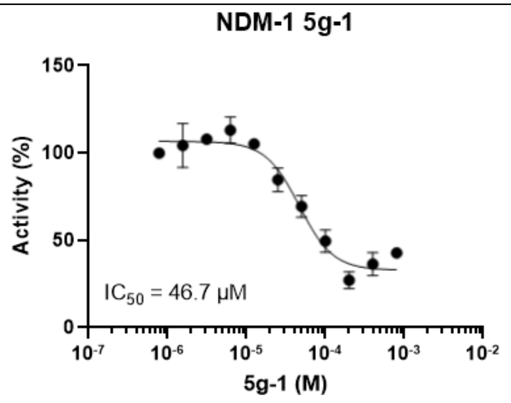
NDM-1 5g-1
Activity (%)
5g-1 (M)
IC50 = 46.7 μM

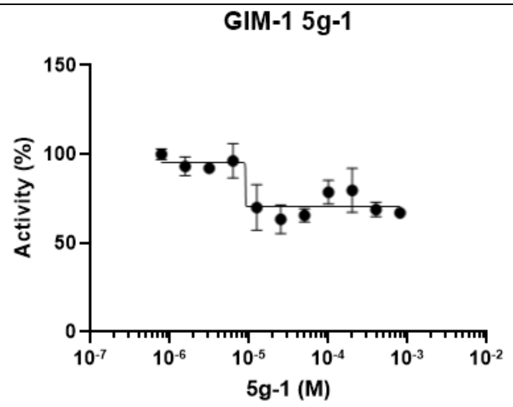
GIM-1 5g-1
Activity (%)
5g-1 (M)

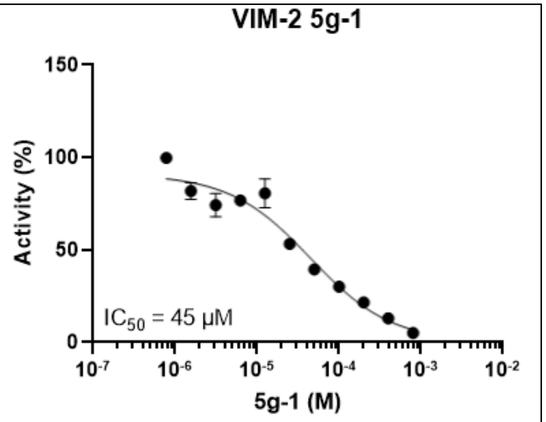
VIM-2 5g-1
Activity (%)
5g-1 (M)
IC50 = 45 μM

5g-2

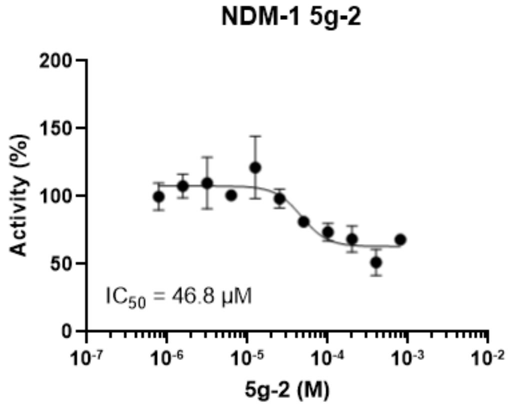
NDM-1 5g-2
Activity (%)
5g-2 (M)
IC50 = 46.8 μM

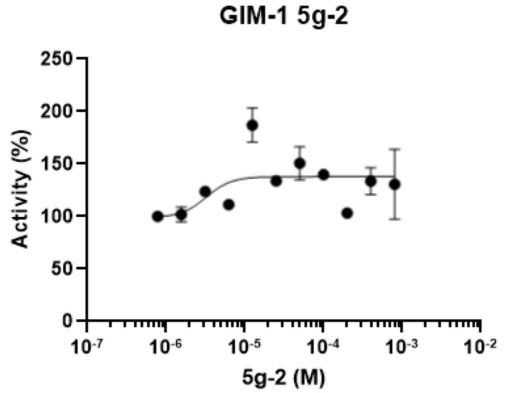
GIM-1 5g-2
Activity (%)
5g-2 (M)

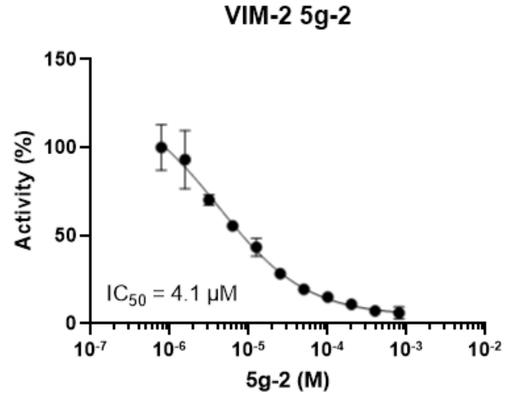
VIM-2 5g-2
Activity (%)
5g-2 (M)
IC50 = 4.1 μM

5h-1

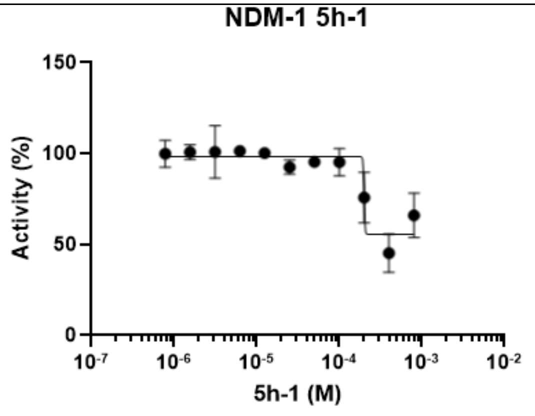
NDM-1 5h-1
Activity (%)
5h-1 (M)

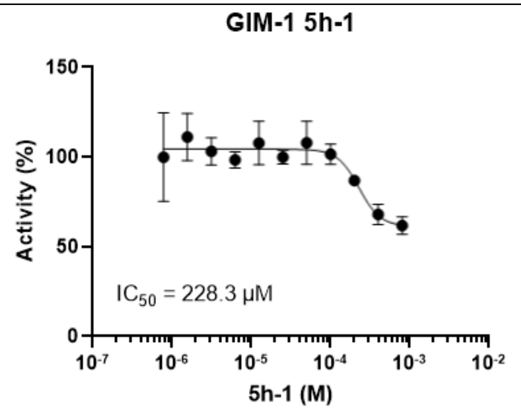
GIM-1 5h-1
Activity (%)
IC_{50} = 228.3 μM
5h-1 (M)

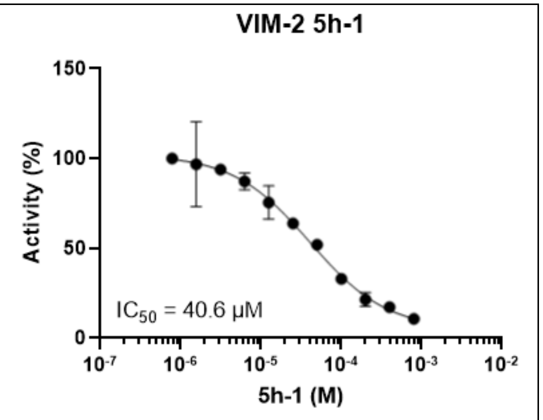
VIM-2 5h-1
Activity (%)
IC_{50} = 40.6 μM
5h-1 (M)

5h-2

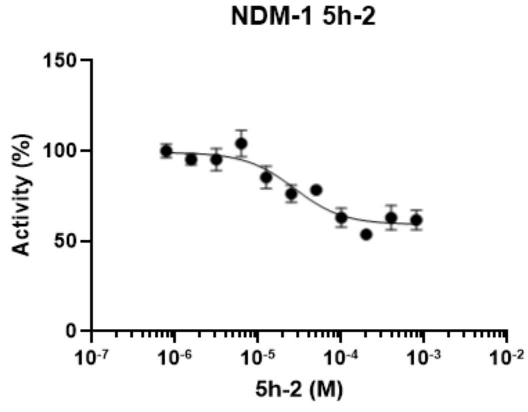
NDM-1 5h-2
Activity (%)
5h-2 (M)

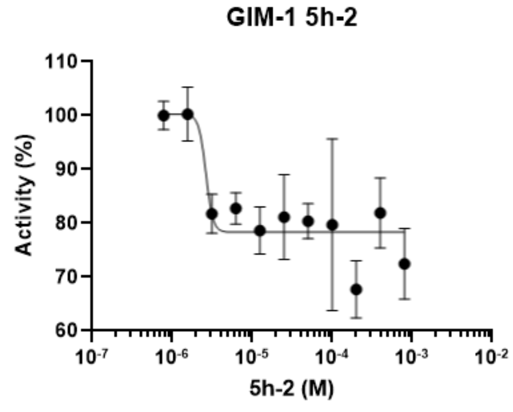
GIM-1 5h-2
Activity (%)
5h-2 (M)

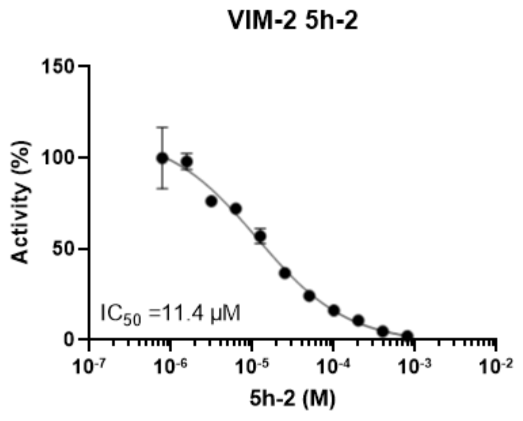
VIM-2 5h-2
Activity (%)
IC_{50} =11.4 μM
5h-2 (M)

5i-1

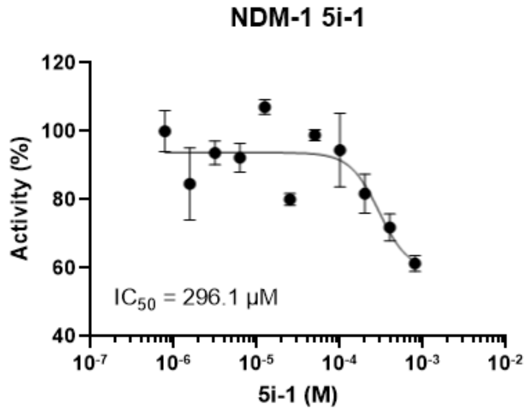
NDM-1 5i-1
Activity (%)
IC50 = 296.1 μM
5i-1 (M)

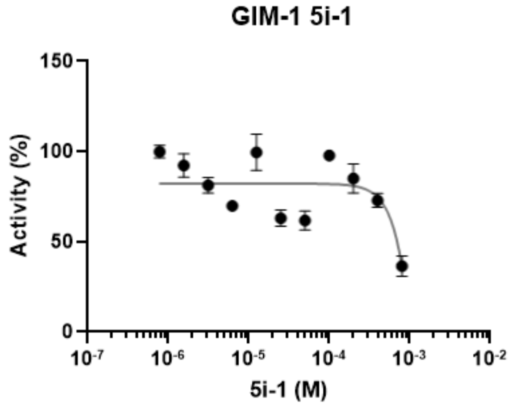
GIM-1 5i-1
Activity (%)
5i-1 (M)

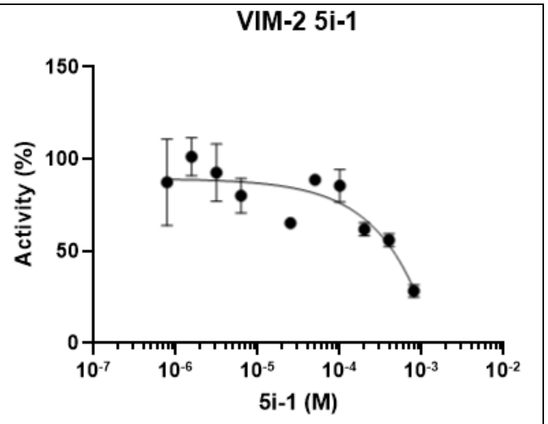
VIM-2 5i-1
Activity (%)
5i-1 (M)

5i-2

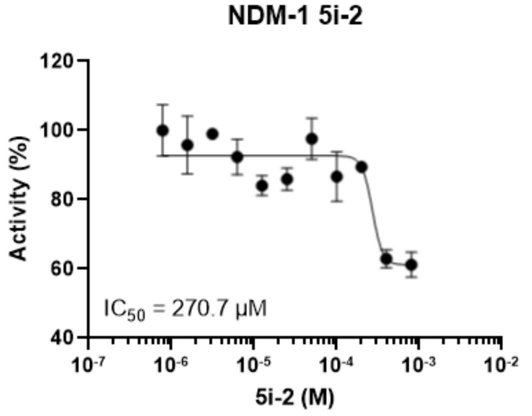
NDM-1 5i-2
Activity (%)
IC50 = 270.7 μM
5i-2 (M)

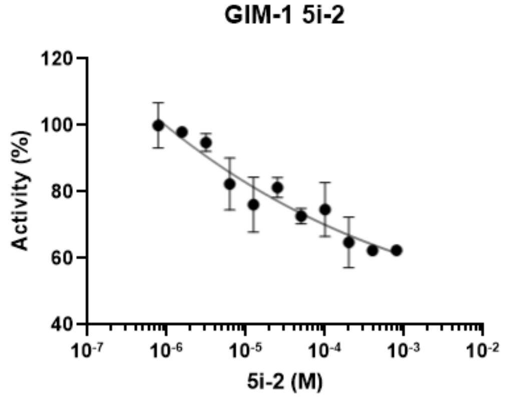
GIM-1 5i-2
Activity (%)
5i-2 (M)

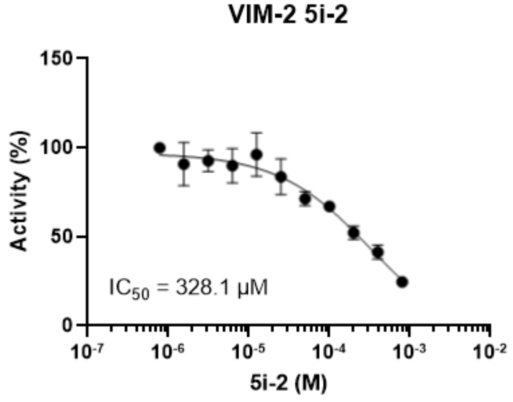
VIM-2 5i-2
Activity (%)
IC50 = 328.1 μM
5i-2 (M)

**5j-1**	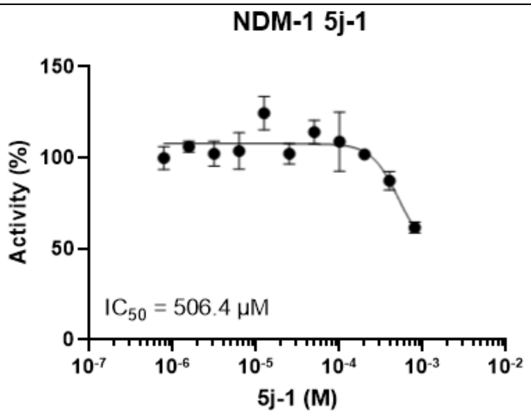NDM-1 5j-1<br>Activity (%)<br>5j-1 (M)<br>IC_{50} = 506.4 μM	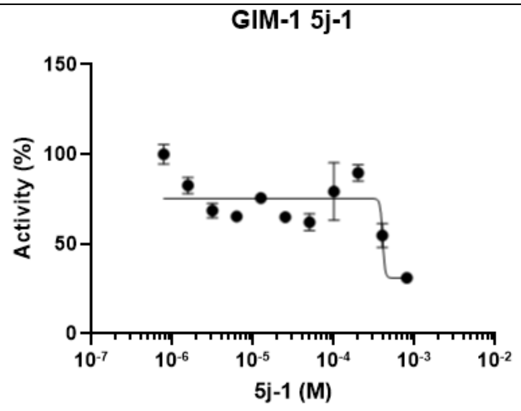GIM-1 5j-1<br>Activity (%)<br>5j-1 (M)	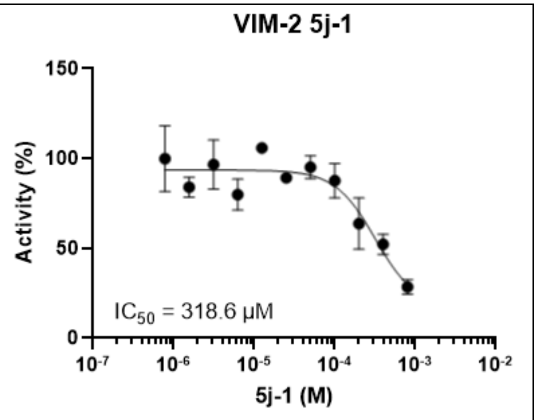VIM-2 5j-1<br>Activity (%)<br>5j-1 (M)<br>IC_{50} = 318.6 μM
**5j-2**	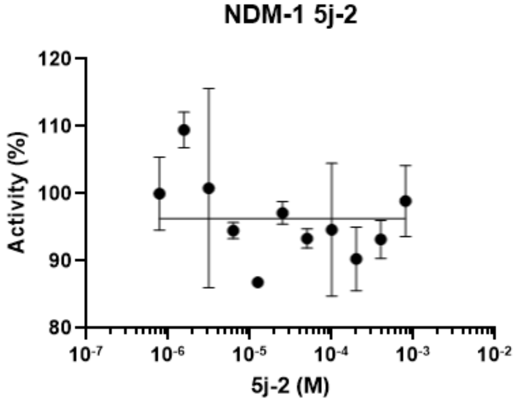NDM-1 5j-2<br>Activity (%)<br>5j-2 (M)	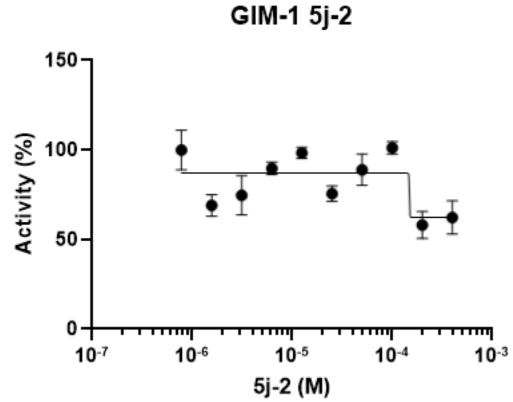GIM-1 5j-2<br>Activity (%)<br>5j-2 (M)	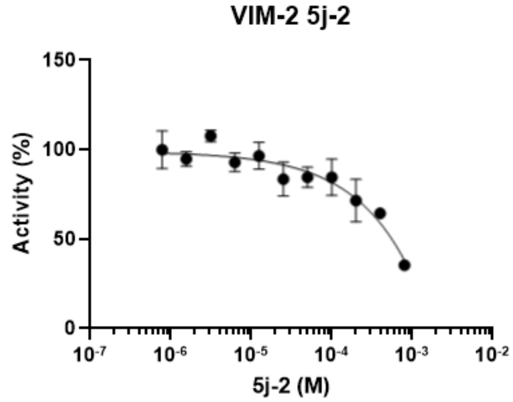VIM-2 5j-2<br>Activity (%)<br>5j-2 (M)

<table>
<tr>
<td>5k-1</td>
<td>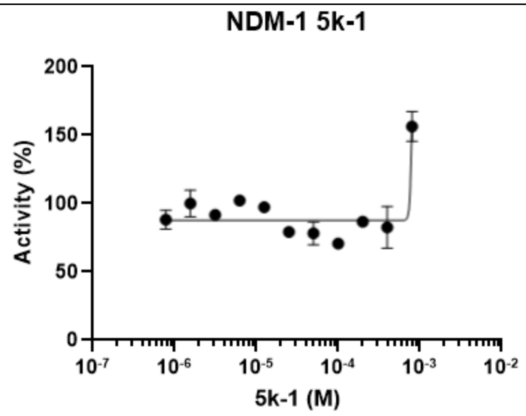
</td>
<td>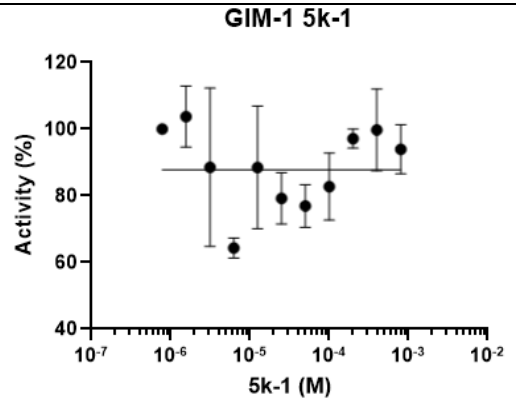
</td>
<td>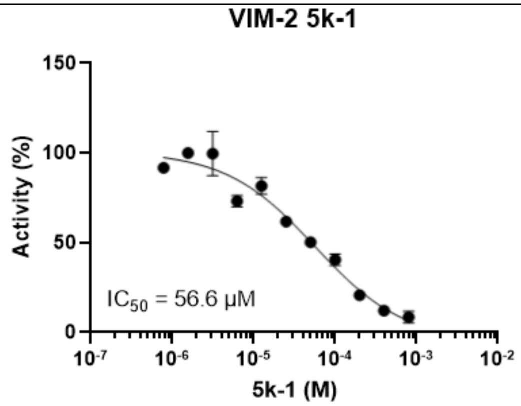
</td>
</tr>
<tr>
<td>5k-2</td>
<td>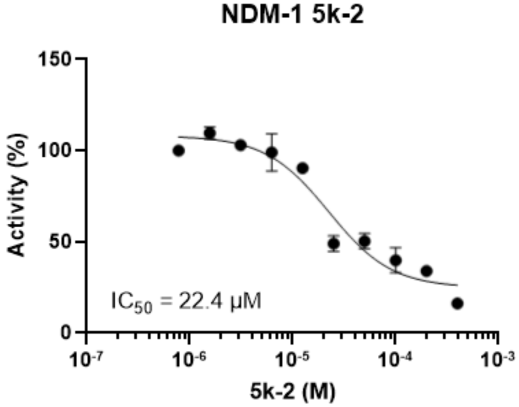
</td>
<td>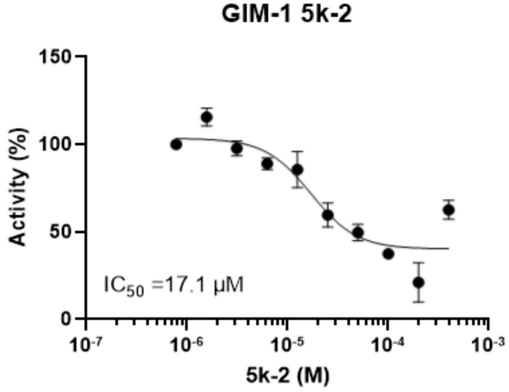
</td>
<td>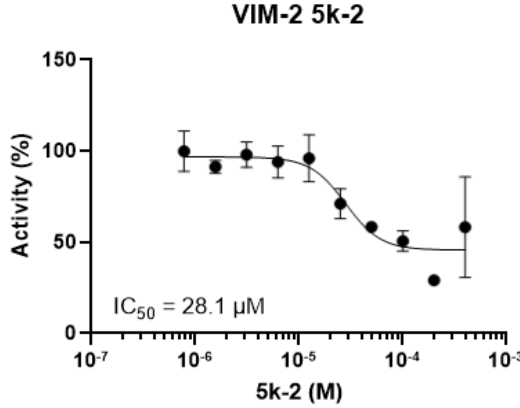
</td>
</tr>
</table>

5l-1

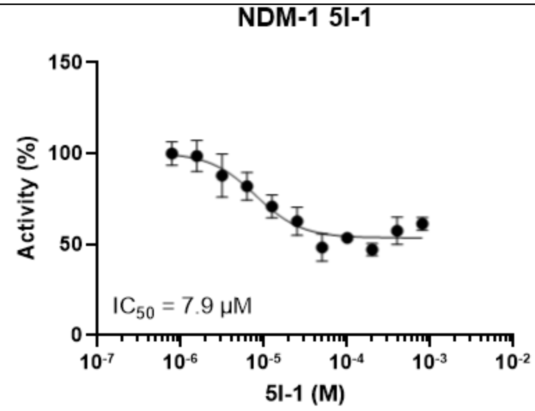

NDM-1 5l-1
Activity (%)
5l-1 (M)
IC_{50} = 7.9 μM

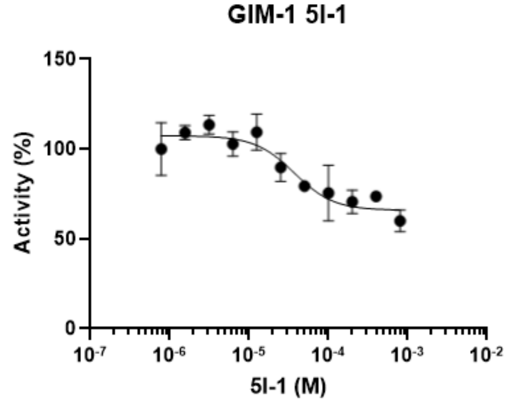

GIM-1 5l-1
Activity (%)
5l-1 (M)

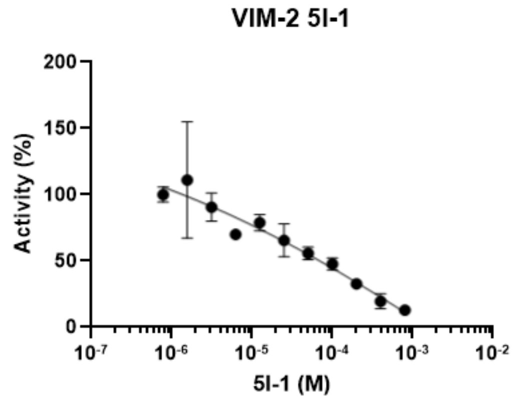

VIM-2 5l-1
Activity (%)
5l-1 (M)

5l-2

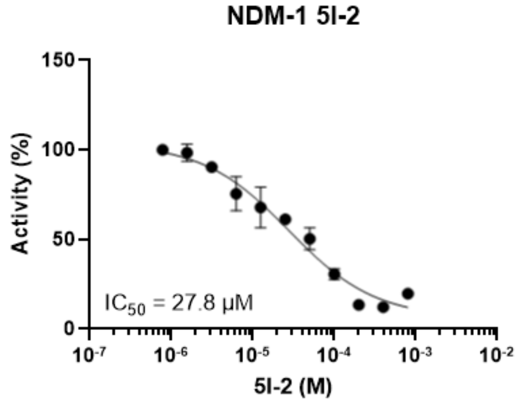

NDM-1 5l-2
Activity (%)
5l-2 (M)
IC_{50} = 27.8 μM

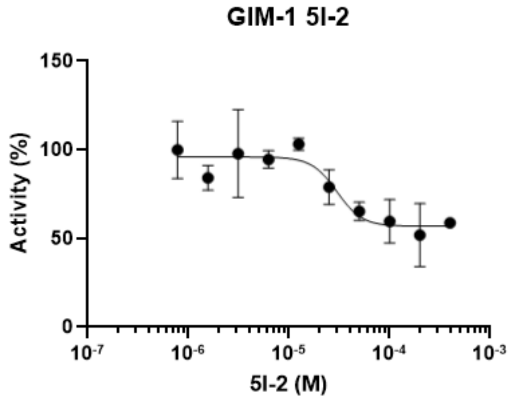

GIM-1 5l-2
Activity (%)
5l-2 (M)

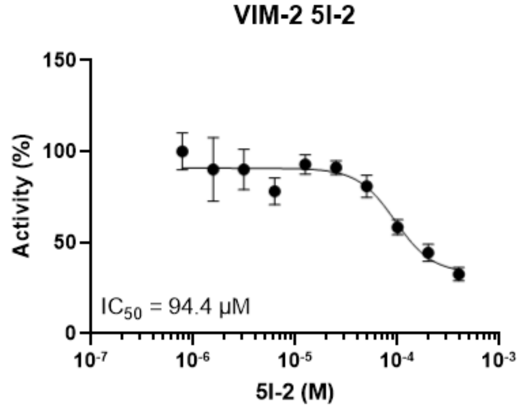

VIM-2 5l-2
Activity (%)
5l-2 (M)
IC_{50} = 94.4 μM

2. Cytotoxicity assay

Table S2. The dose-response graphs for the cytotoxicity testing of the inhibitors on MCF-7 cells. For details, see the experimental section in the main text. No toxicity has been observed at the tested concentrations for any of the compounds.

Inhibitors	MCF-7	Inhibitors	MCF-7
**5a-1**	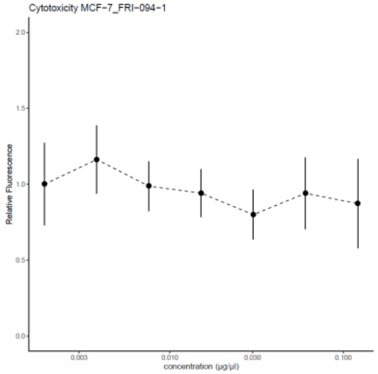	**5g-1**	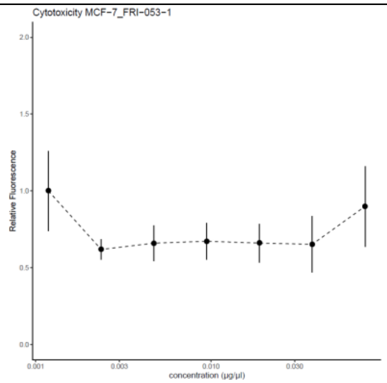
**5a-2**	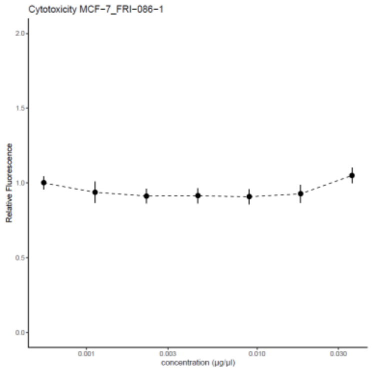	**5g-2**	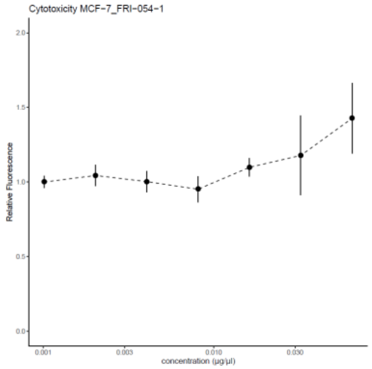

5b-1

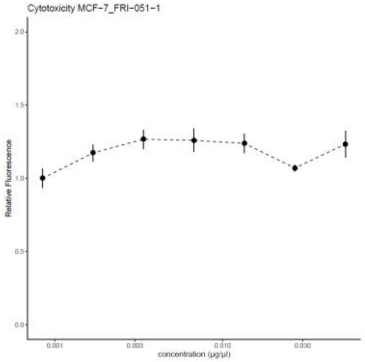

5h-1

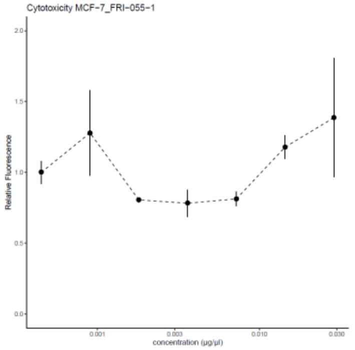

5b-2

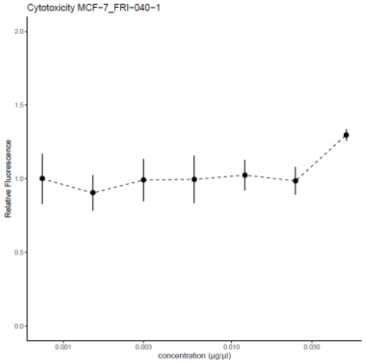

5i-1

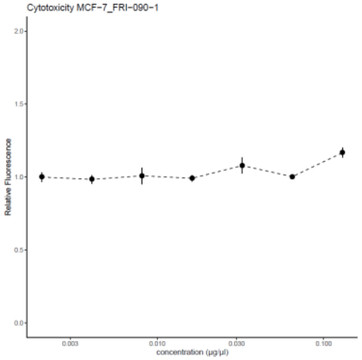

5c-1

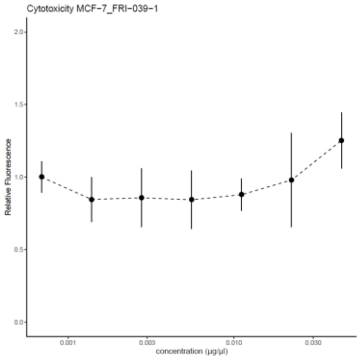

5i-2

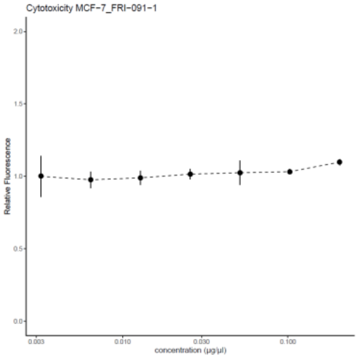

**5c-2**	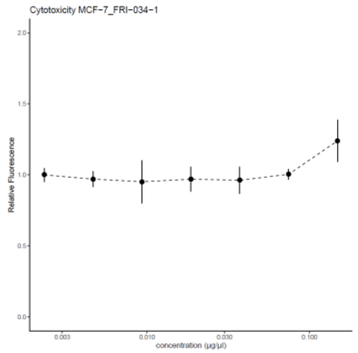	**5j-1**	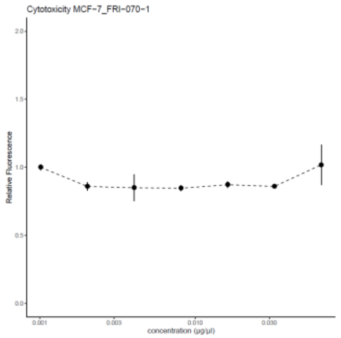
**5d-1**	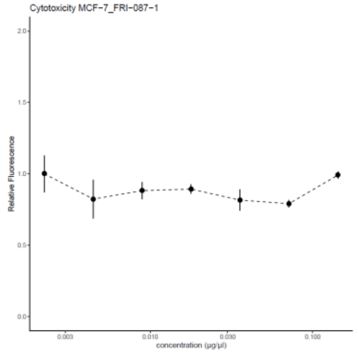	**5j-2**	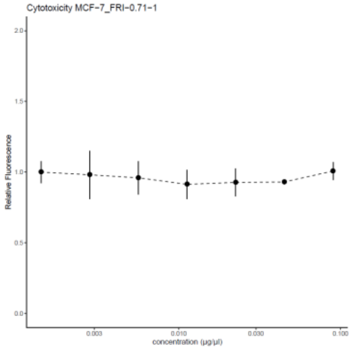
**5d-2**	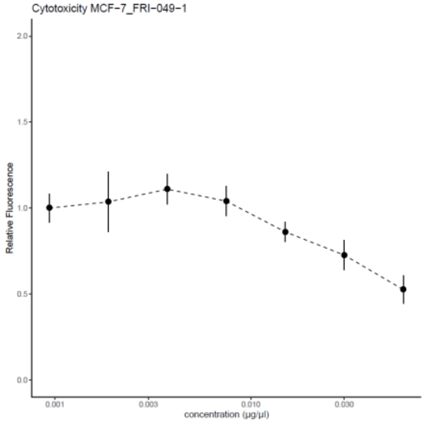	**5k-1**	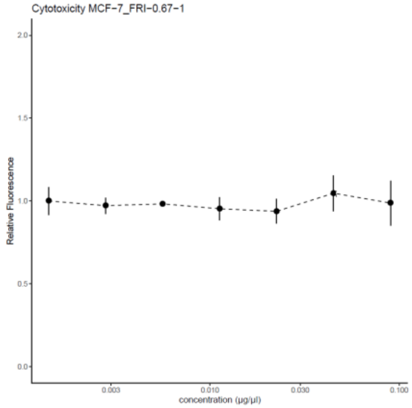

**5f-1**	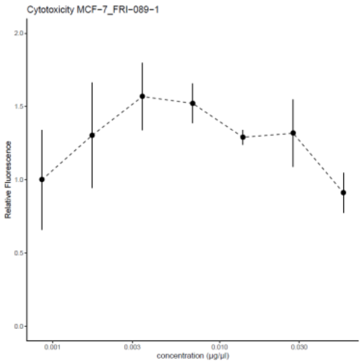	**5l-1**	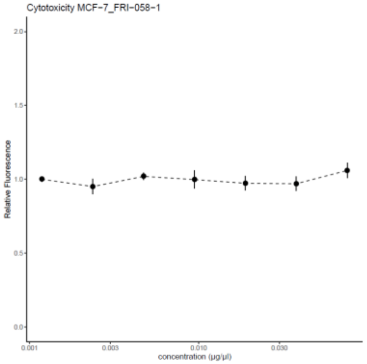
**5f-2**	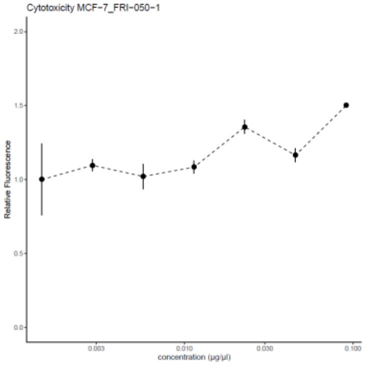	**5l-2**	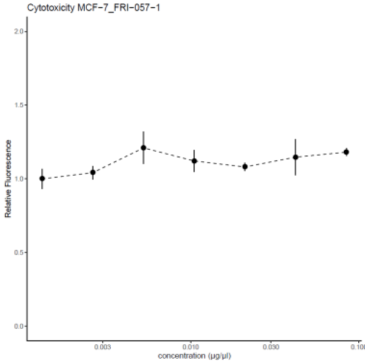

3. Protein sequence alignment[1]

The sequence of NDM-1, VIM-2 and GIM-1 aligned, to show similarities and differences.

'.' Semi-conservative amino acids

'*' Conservative (identical) amino acids

':' Conservative (similar) amino acids

```
CLUSTAL O(1.2.4) multiple sequence alignment


gi|953357714|gb|ALO69078.1|GIM-1|      ------MK---NVLVFLI---LLV-ALPA------------LAQGHKPLEVIKIEDGVYL 35
gi|595266892|gb|AHM26723.1|NDM-1|      MELPNIMHPVAKLSTALAAALMLSGCMPGEIRPTIGQQMETGDQRFGDLVFRQLAPNVWQ 60
gi|28569602|gb|AAO43978.1|VIM-2|       -----MFKLLSKLLVYLTASIMAIASPLAFSVDSSGEYPTVSEIPVGEVRLYQIADGVWS 55
                                             ::   :: . *    :   .  .                   : . ::  .*:

gi|953357714|gb|ALO69078.1|GIM-1|      HTSFKNIEGYGLVDSNGLVVLDNNQAYIIDTPWSEEDTKLLLSWATD-RGYQVMASISTH 94
gi|595266892|gb|AHM26723.1|NDM-1|      HTSYLDMPGFGAVASNGLIVRDGGRVLVVDTAWTDDQTAQILNWIKQEINLPVALAVVTH 120
gi|28569602|gb|AAO43978.1|VIM-2|       HIATQSFDG-AVYPSNGLIVRDGDELLLIDTAWGAKNTAALLAEIEKQIGLPVTRAVSTH 114
                                       * :  .: * .   ****:* *...  ::** *  .:*  :*    .  .  *  :: **

gi|953357714|gb|ALO69078.1|GIM-1|      SHEDRTAGIKLLNSKSIPTYTSELTKKLLAREGKPVPTHYFKD-------DEFTLGNGLI 147
gi|595266892|gb|AHM26723.1|NDM-1|      AHQDKMGGMDALHAAGIATYANALSNQLAPQEGMVAAQHSLTFAANGWVEPATAPNFGPL 180
gi|28569602|gb|AAO43978.1|VIM-2|       FHDDRVGGVDVLRAAGVATYASPSTRRLAEVEGNEIPTHSL----EGLSSSGDAVRFGPV 170
                                        *:*: .*:. *.: .: **:.  :.:*   **     * :            :   * :

gi|953357714|gb|ALO69078.1|GIM-1|      ELYYPGAGHTEDNIVAWLPKSKILFGGCLVRSHEWEGLGYVGDASISSWADSIKNIVSKK 207
gi|595266892|gb|AHM26723.1|NDM-1|      KVFYPGPGHTSDNITVGIDGTDIAFGGCLIKDSKAKSLGNLGDADTEHYAASARAFGAAF 240
gi|28569602|gb|AAO43978.1|VIM-2|       ELFYPGAAHSTDNLVVYVPSASVLYGGCAIYELSRTSAGNVADADLAEWPTSIERIQQHY 230
                                       :::*** .*: **:.. :  :.: :*** : . .  . * :.**.   :  * . :

gi|953357714|gb|ALO69078.1|GIM-1|      YPIQMVVPGHGKVGSSDILDHTIDLAESASNKLMQPTAEASAD  250
gi|595266892|gb|AHM26723.1|NDM-1|      PKASMIVMSHSAPDSRAAITHTARMADKLR-------------  270
gi|28569602|gb|AAO43978.1|VIM-2|       PEAQFVIPGHGLPGGLDLLKHTTNVVKAHTNRSVVE-------  266
```

Identical positions: 45, similar positions: 47

4. NMR spectra of the synthesized compounds

Tert-butyl 2-((benzylamino)(diethoxyphosphoryl)methyl)piperidine-1-carboxylate **(2)**

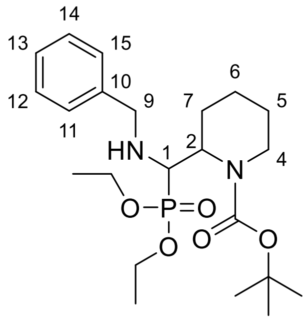

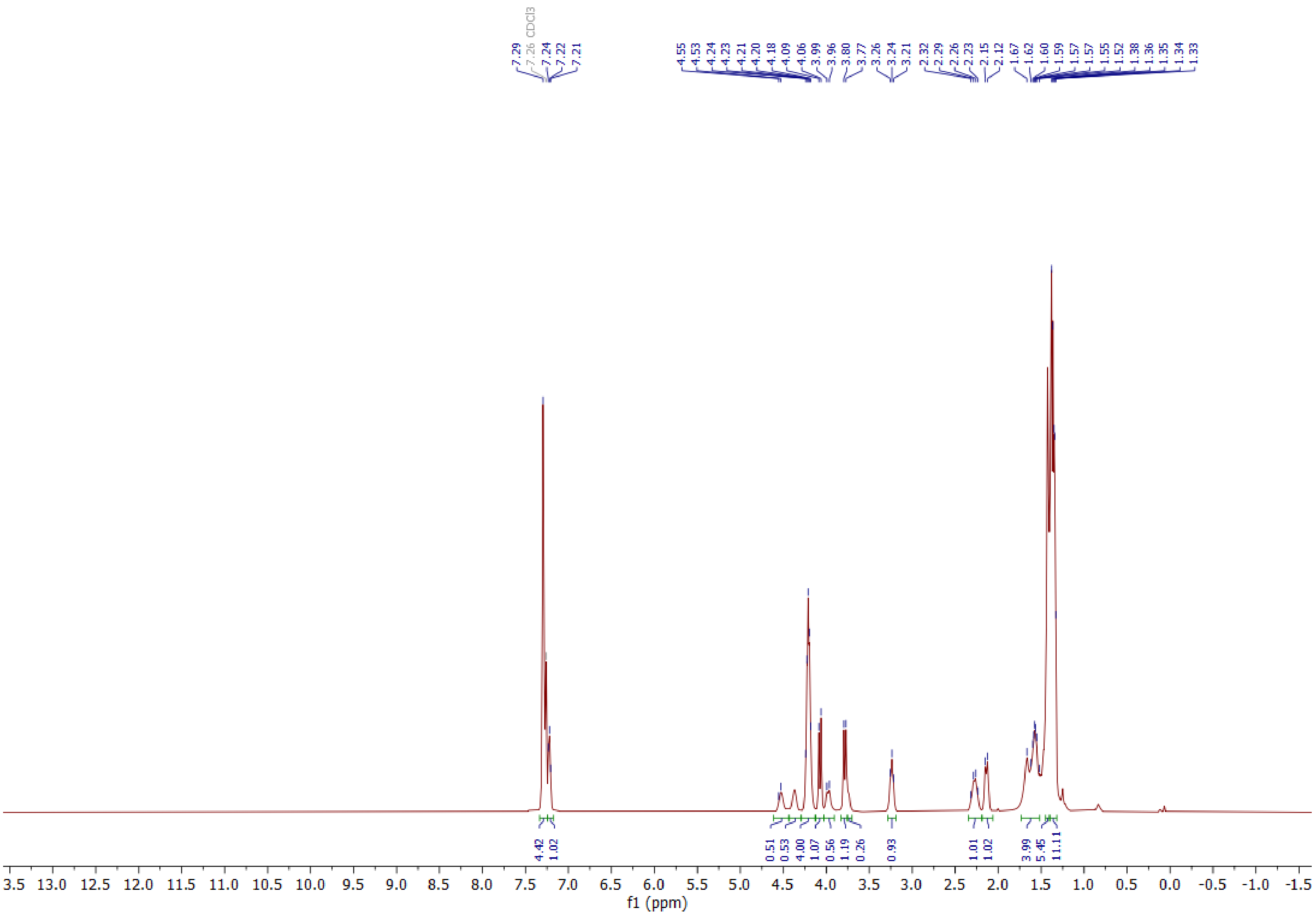

Figure S1. ^{1}H NMR spectrum of **2** diastereomer 1.

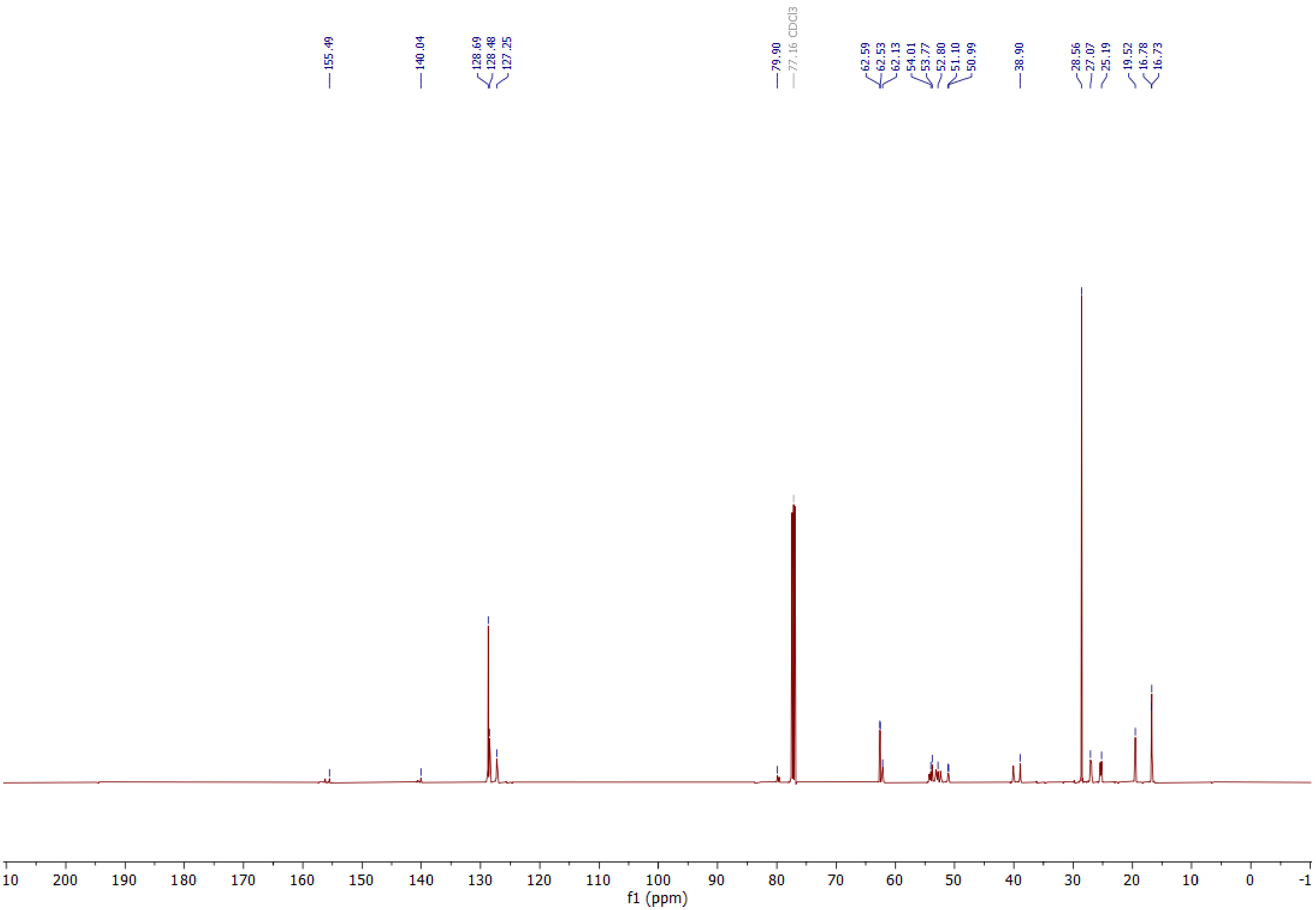

Figure S2. ^{13}C NMR spectrum of **2** diastereomer 1.

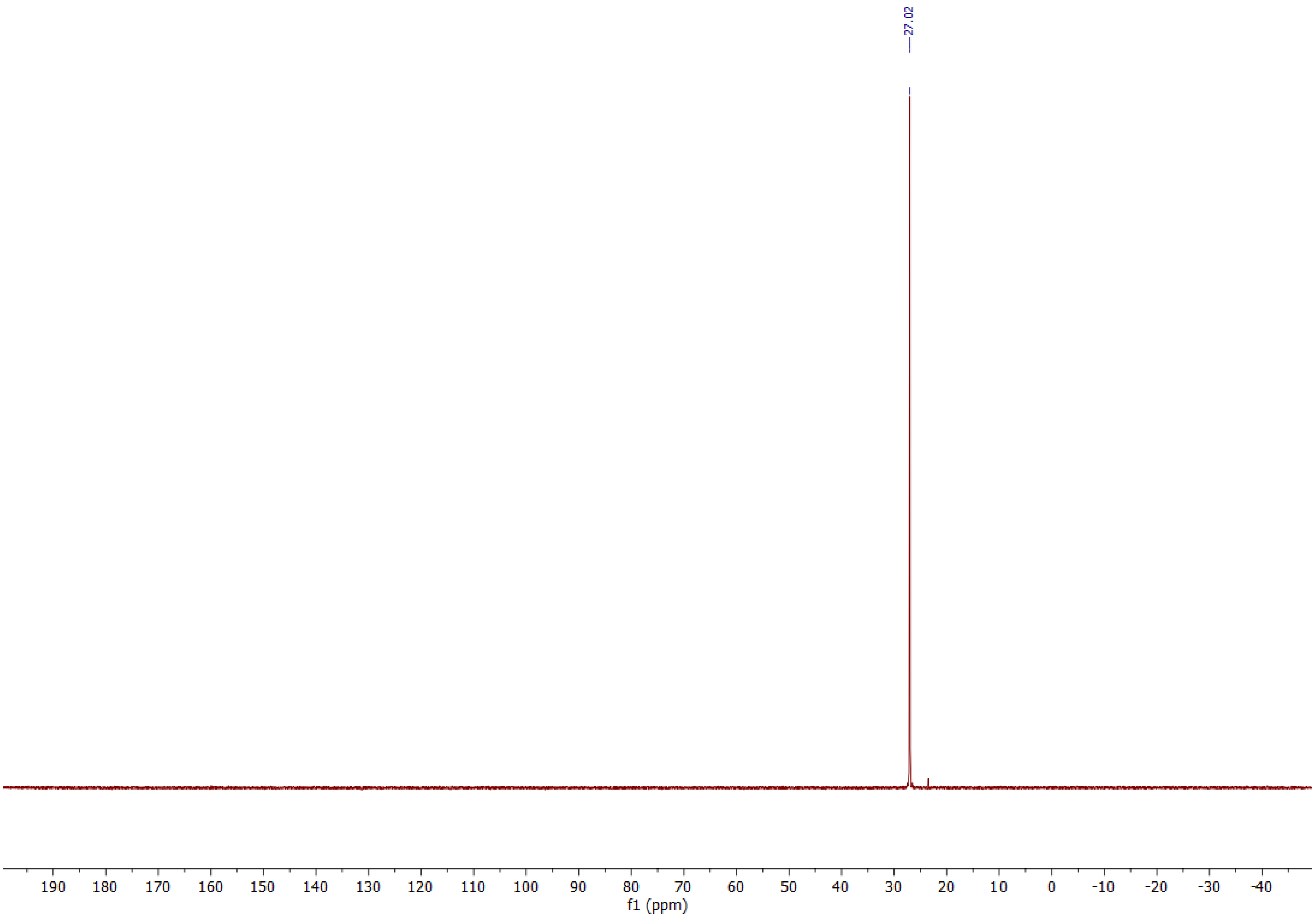

Figure S3. ^{31}P NMR spectrum of **2** diastereomer 1.

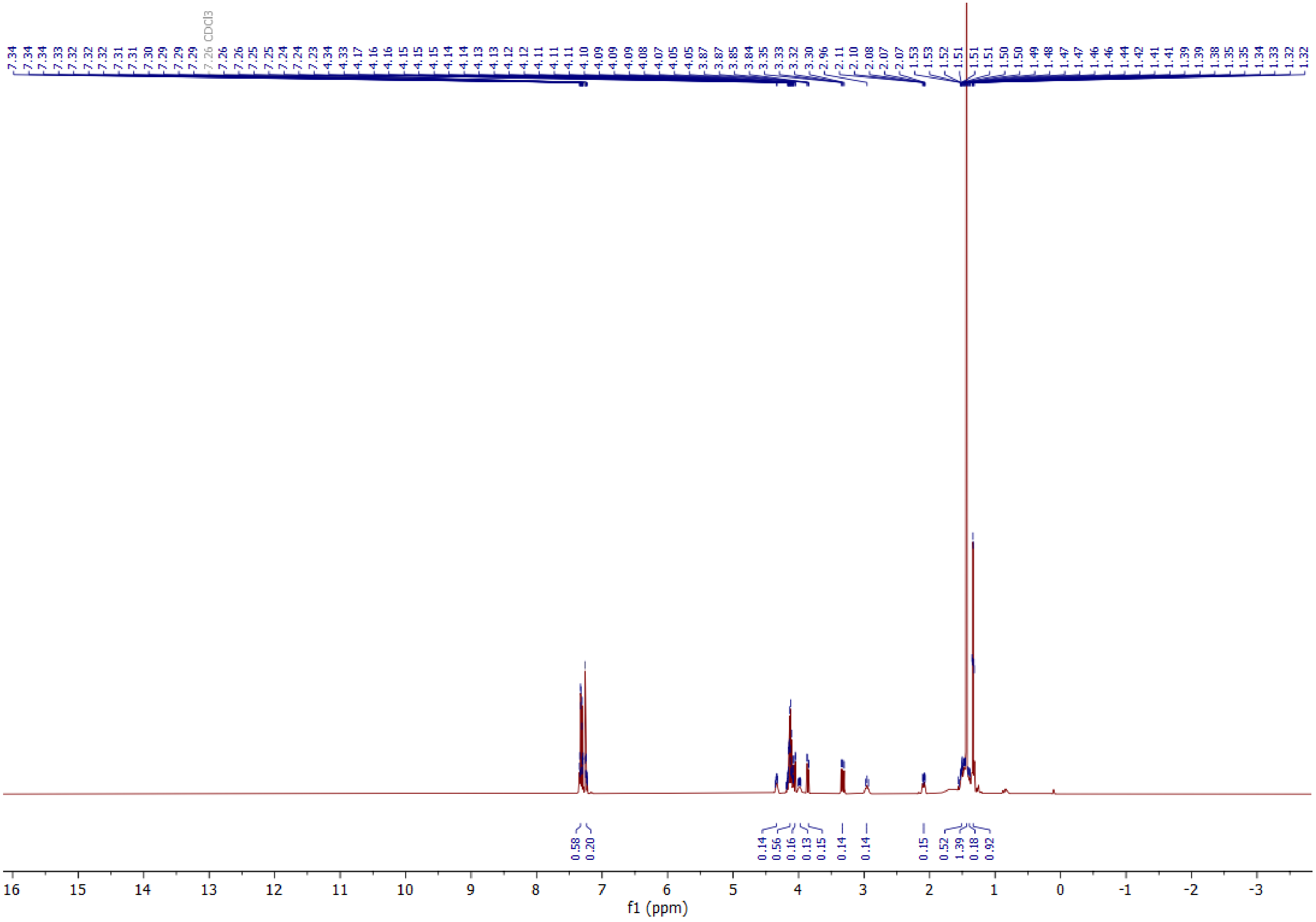

Figure S4. ^{1}H NMR spectrum of **2** diastereomer 2.

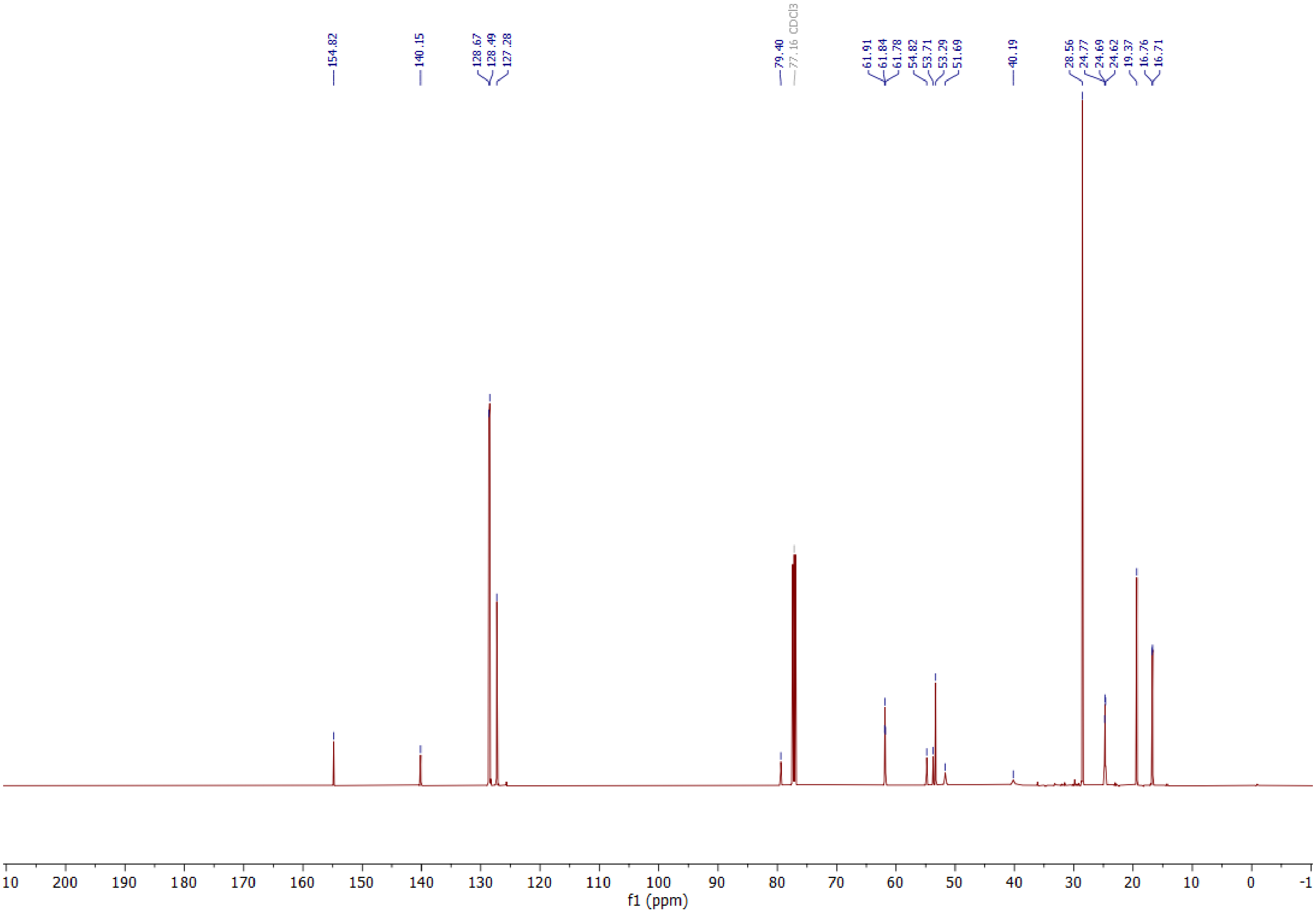

Figure S5. ^{13}C NMR spectrum of **2** diastereomer 2.

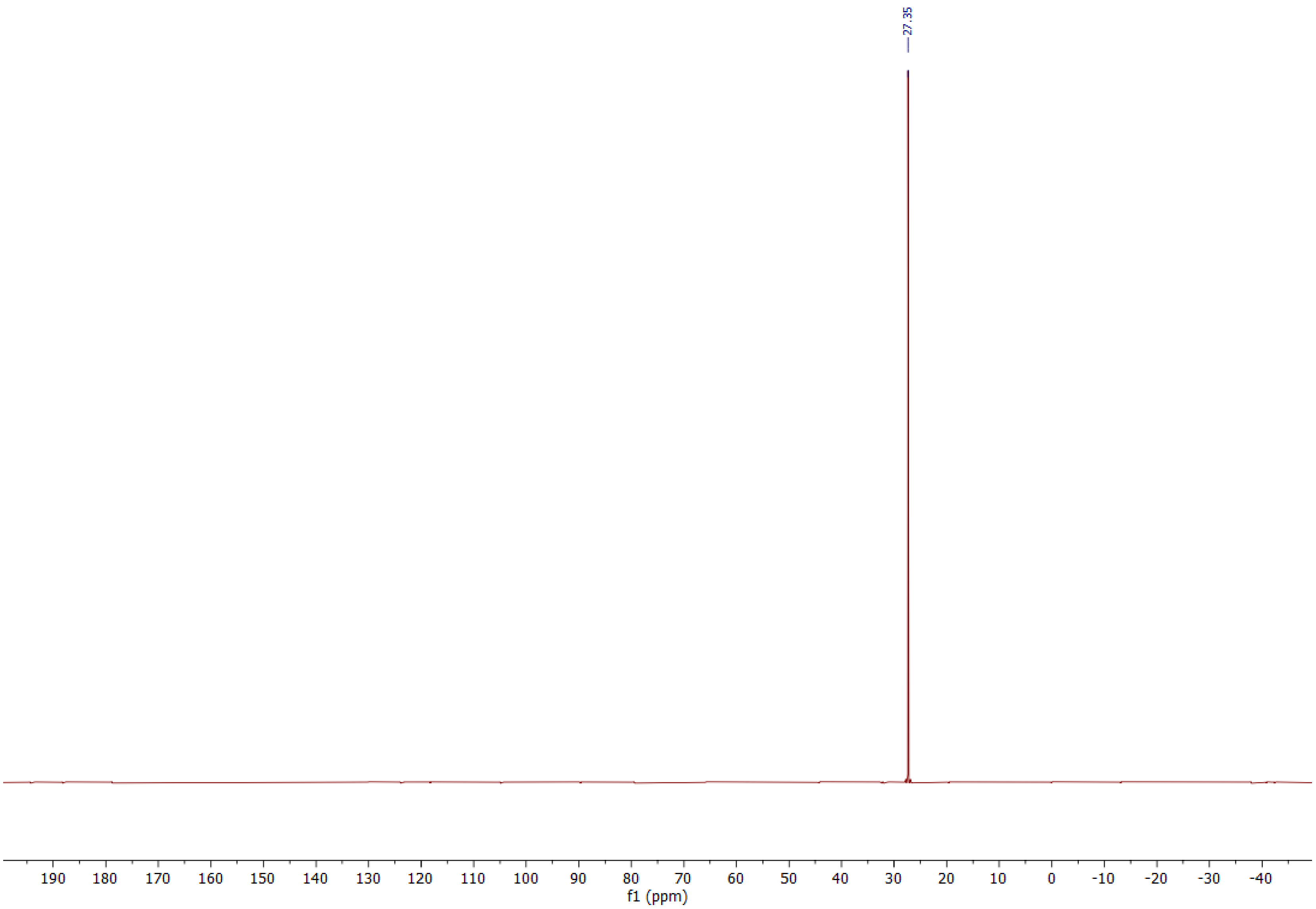

Figure S6. ^{31}P NMR spectrum of **2** diastereomer 2.

Tert-butyl 2-(amino(diethoxyphosphoryl)methyl)piperidine-1-carboxylate **(3)**

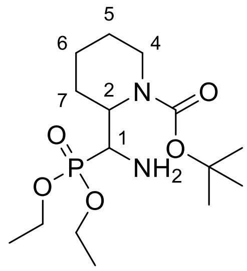

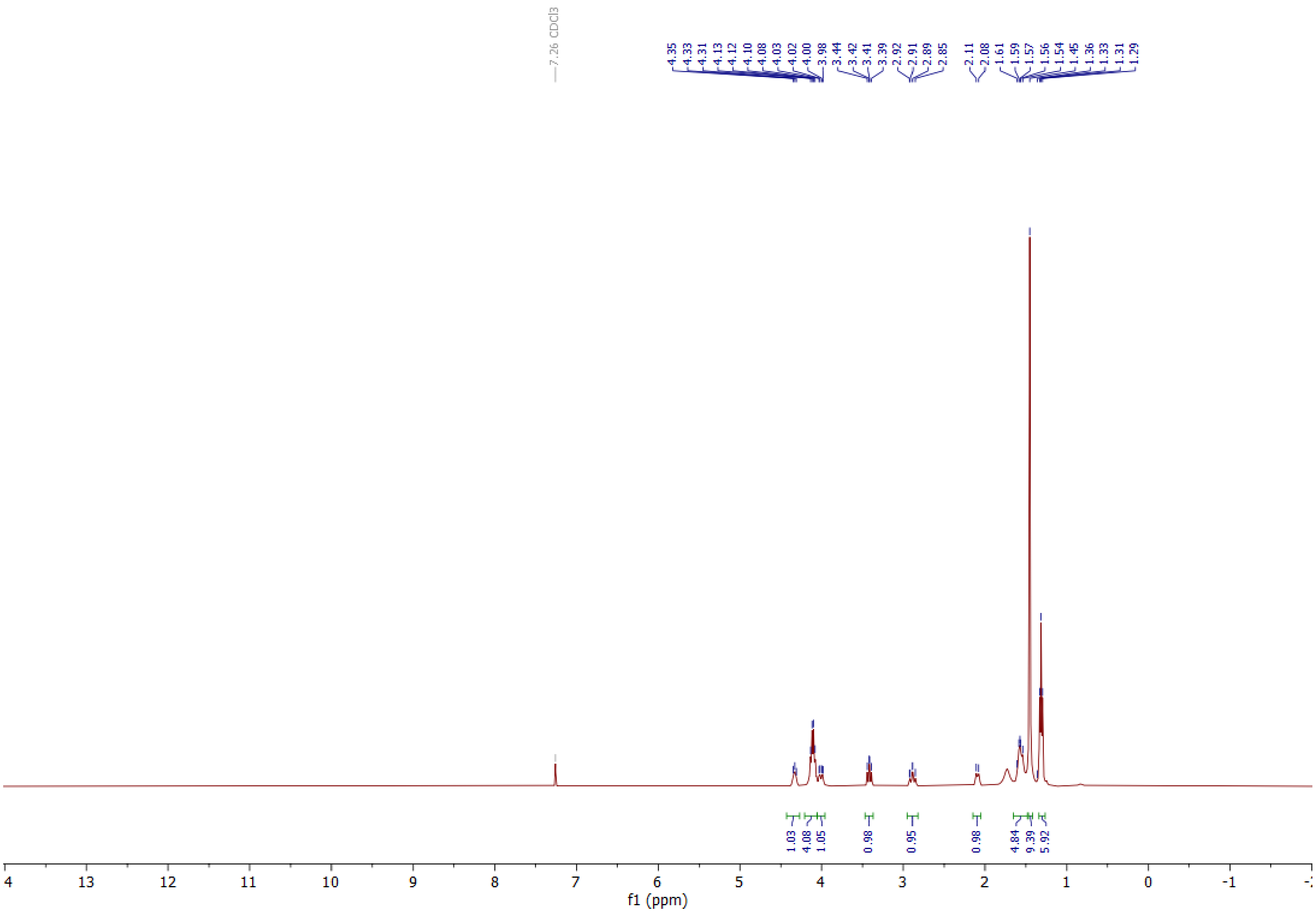

Figure S7. ^{1}H NMR spectrum of **3** diastereomer 1.

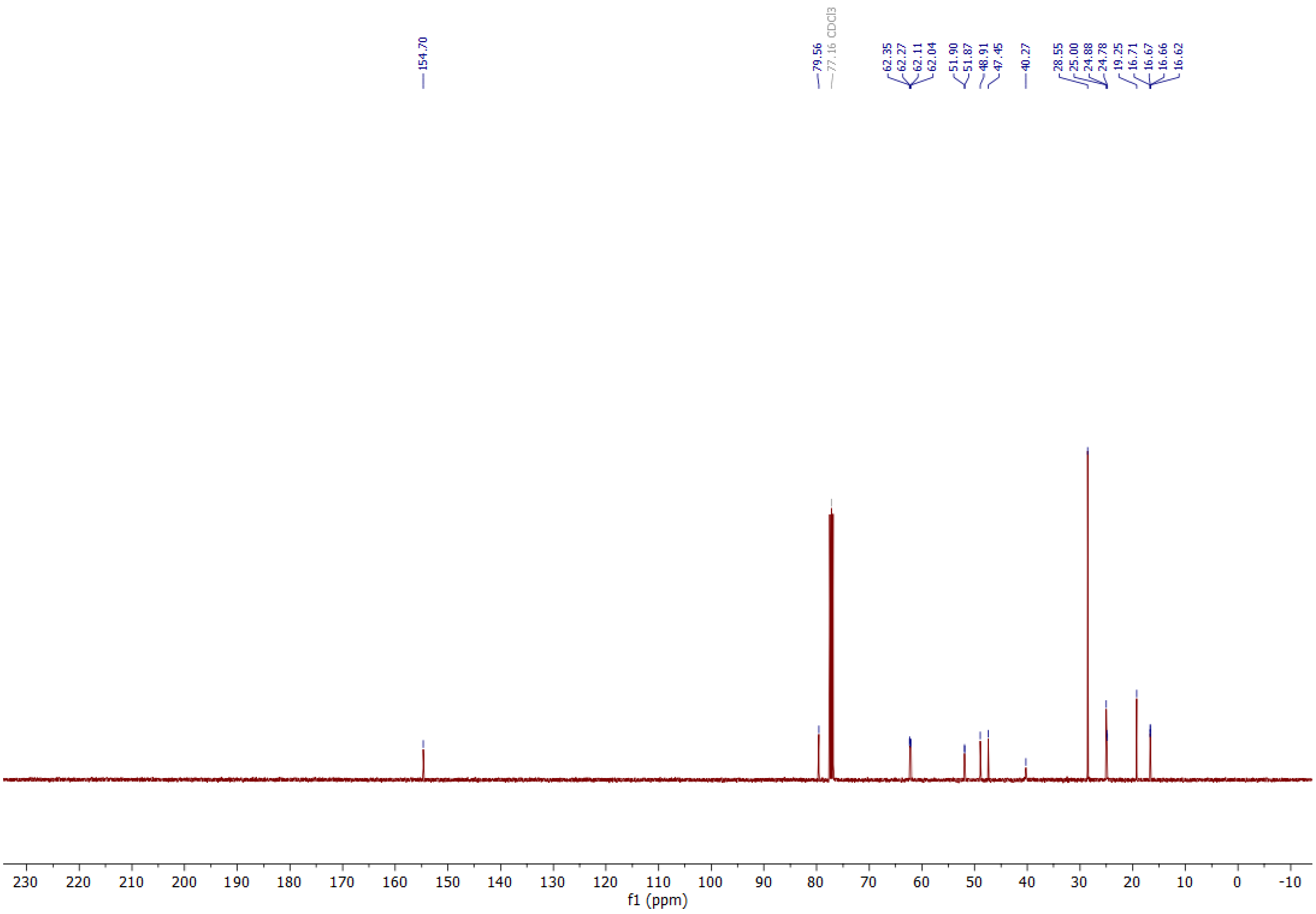

Figure S8. ^{13}C NMR spectrum of **3** diastereomer 1.

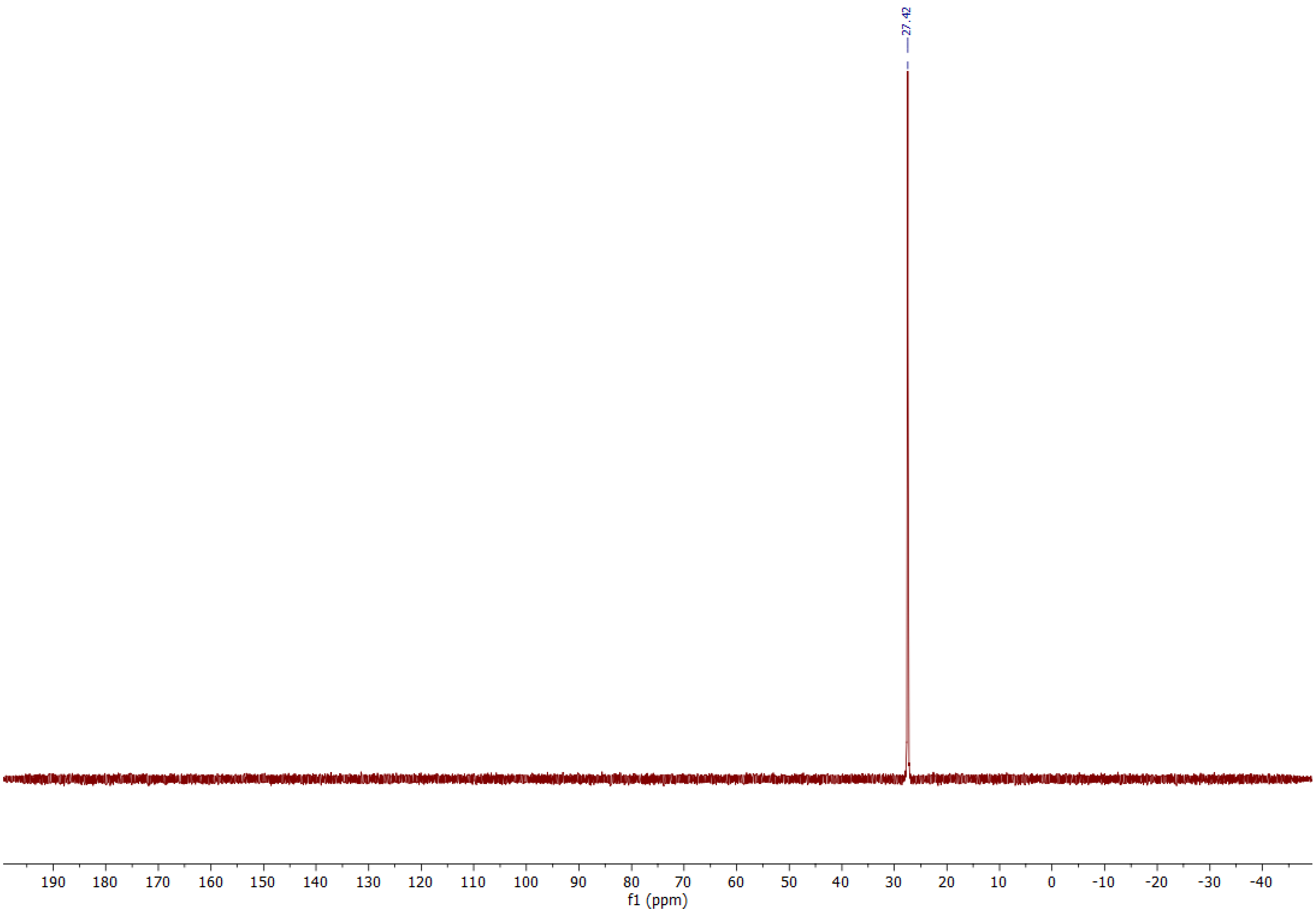

Figure S9. ^{31}P NMR spectrum of **2** diastereomer 1.

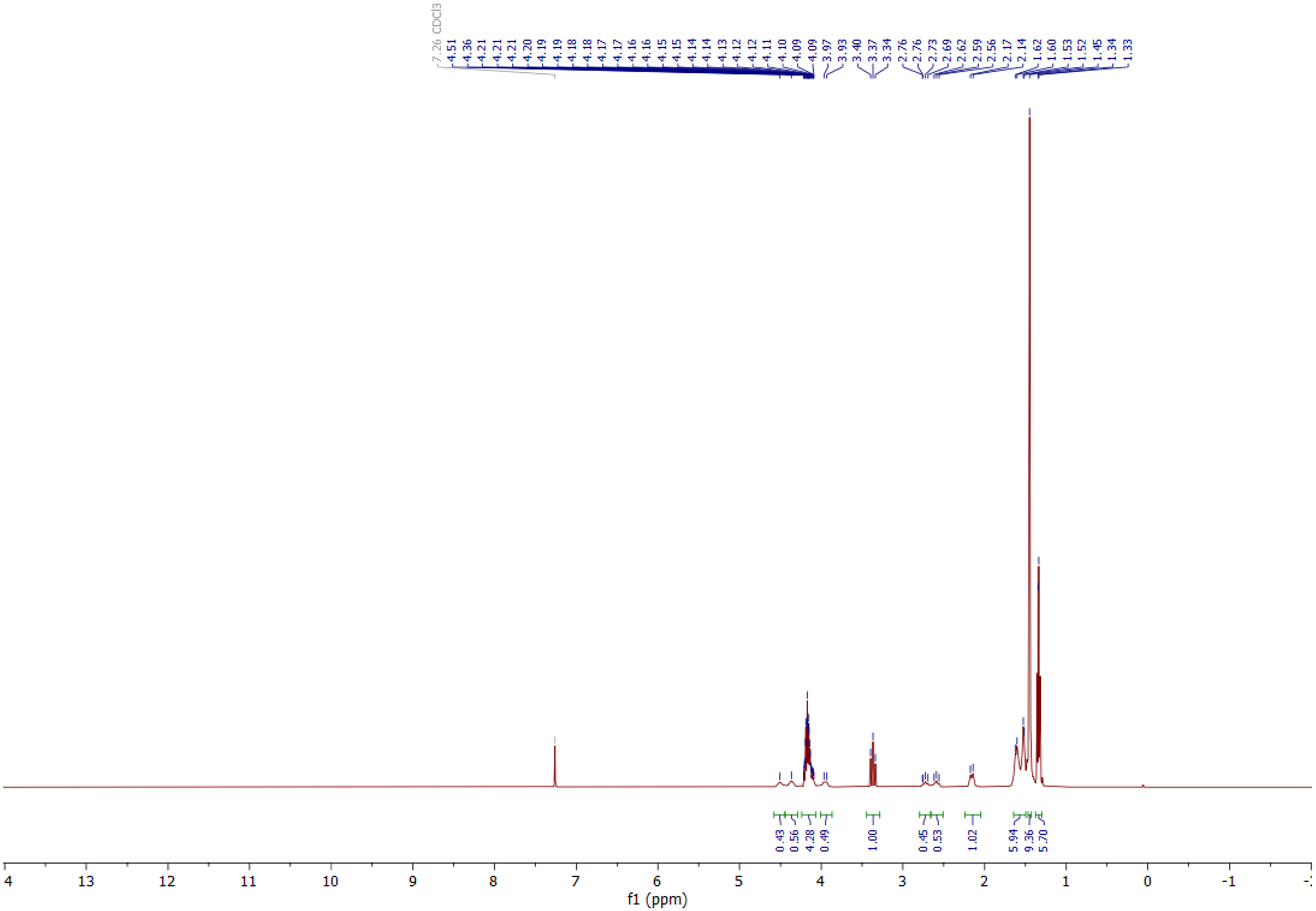

Figure S10. ^{1}H NMR spectrum of **3** diastereomer 2.

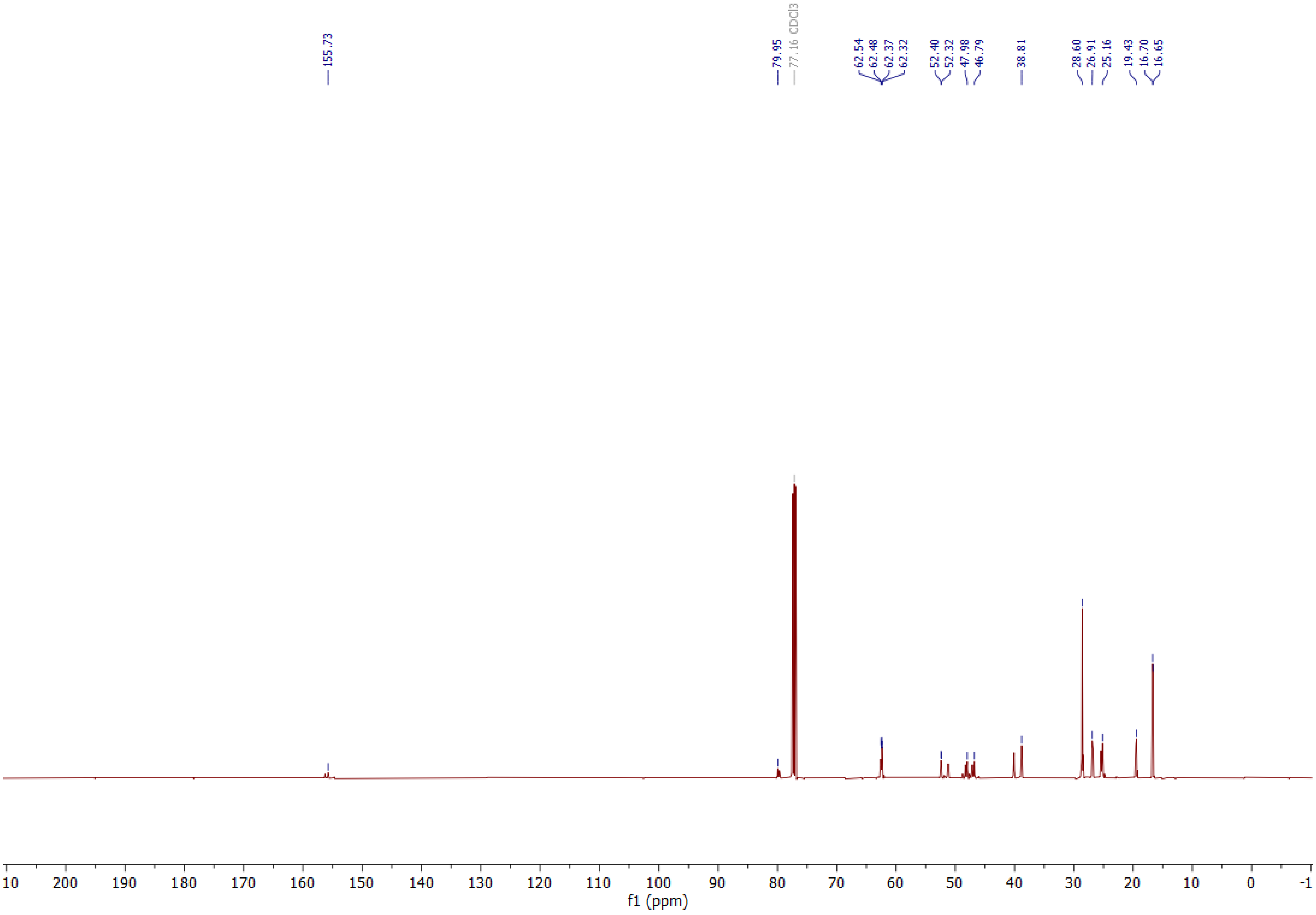

Figure S11. ^{13}C NMR spectrum of **3** diastereomer 2.

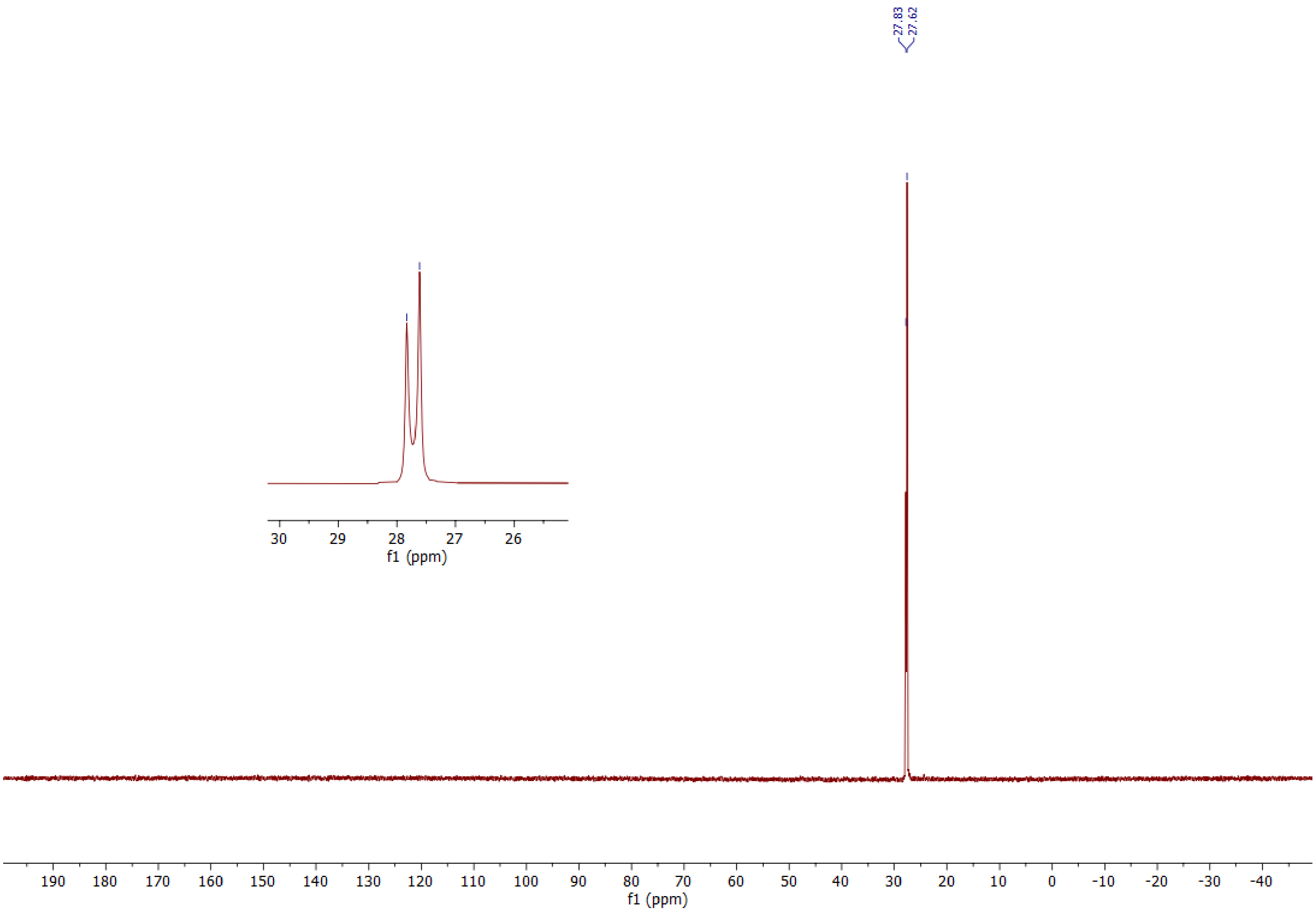

Figure S12. ^{31}P NMR spectrum of **3** diastereomer 2.

Tert-butyl 2-((diethoxyphosphoryl)(2-(4-methyl-2-thioxo-2,3-dihydrothiazol-5- yl)acetamido)methyl)piperidine-1-carboxylate **(4a)**

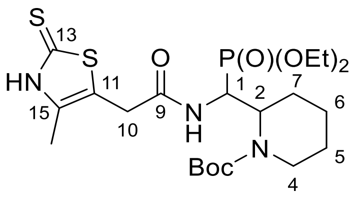

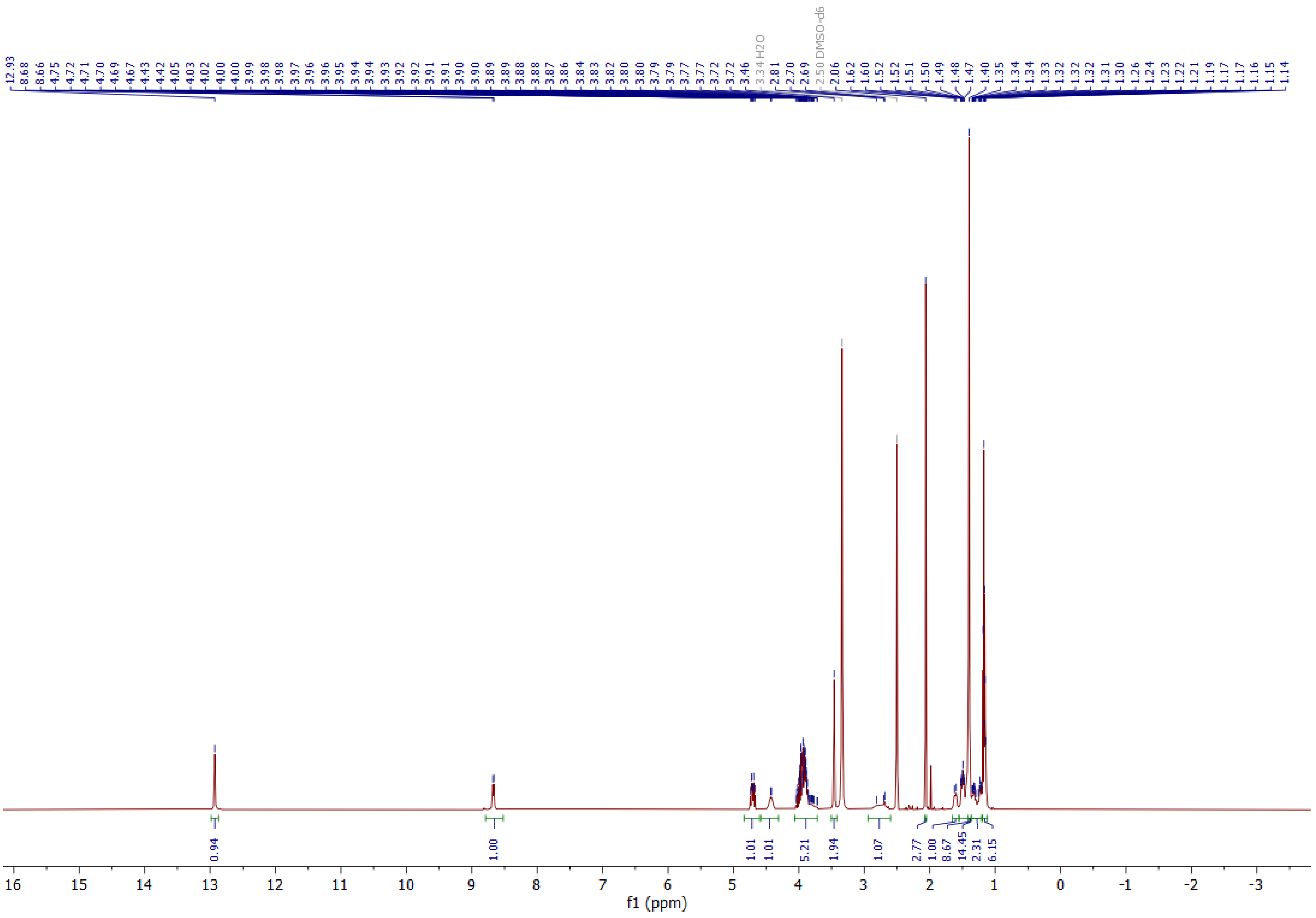

Figure S13. ^{1}H NMR spectrum of **4a** diastereomer 1.

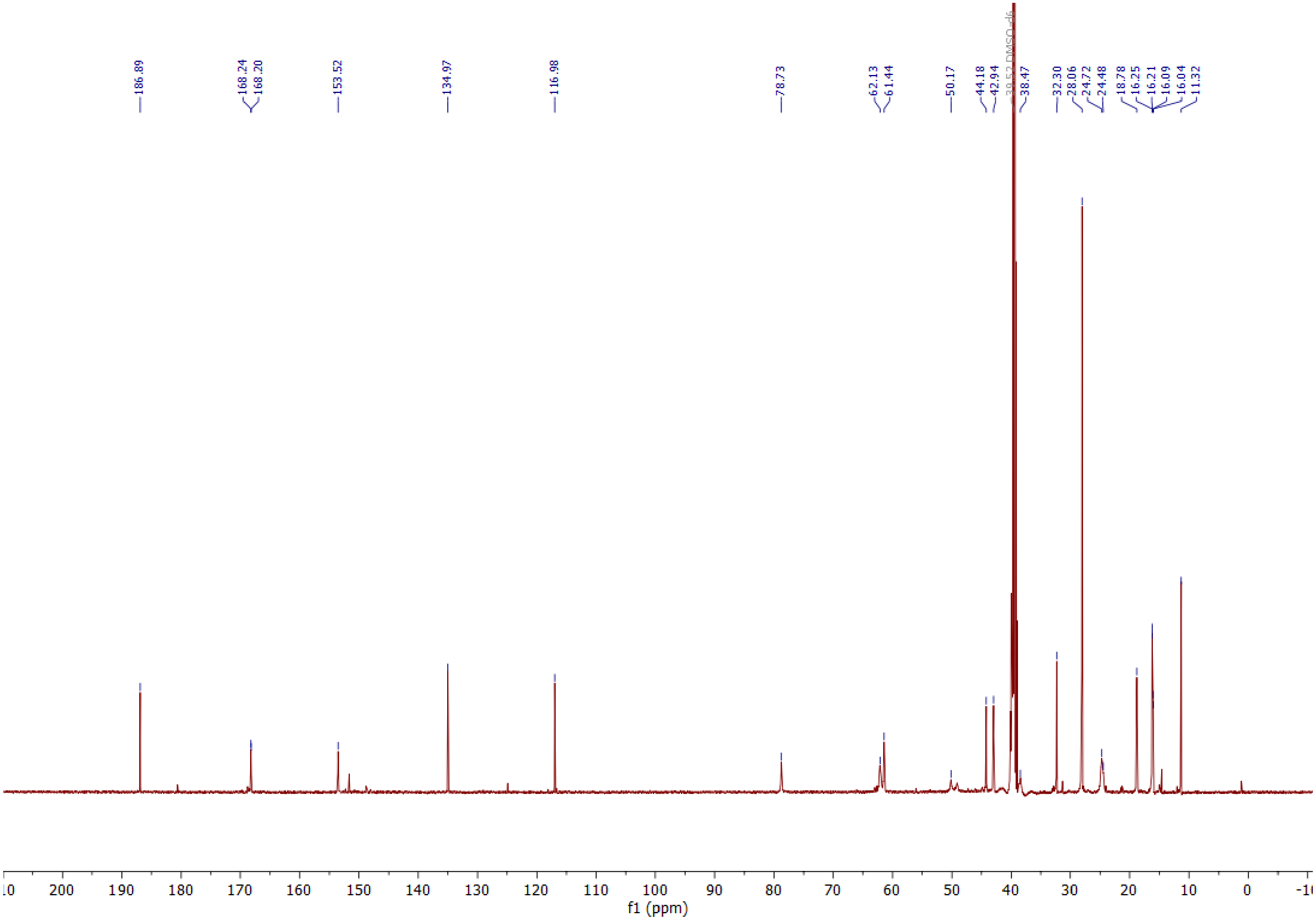

Figure S14. ^{13}C NMR spectrum of **4a** diastereomer 1.

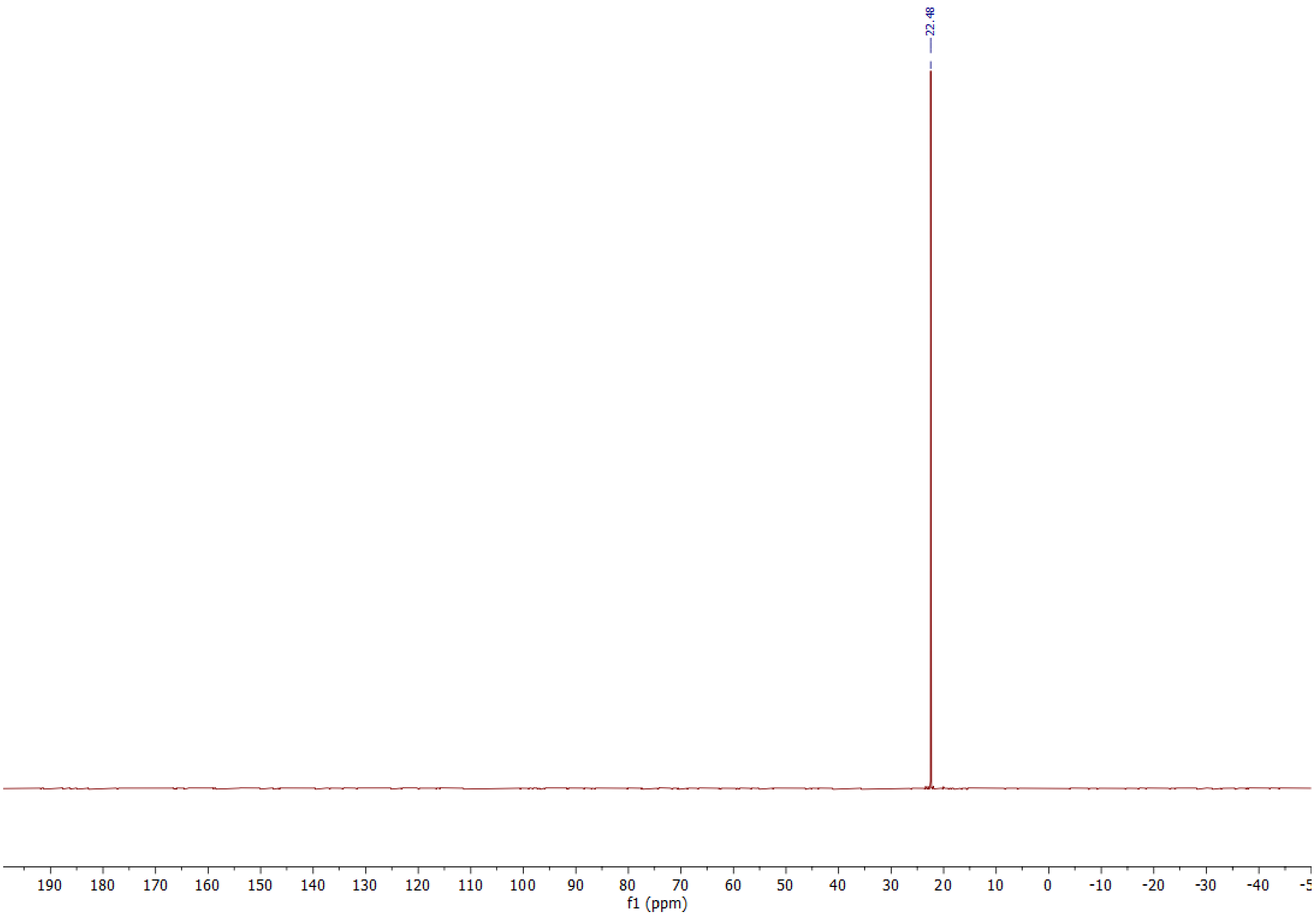

Figure S15. ^{31}P NMR spectrum of **4a** diastereomer 1.

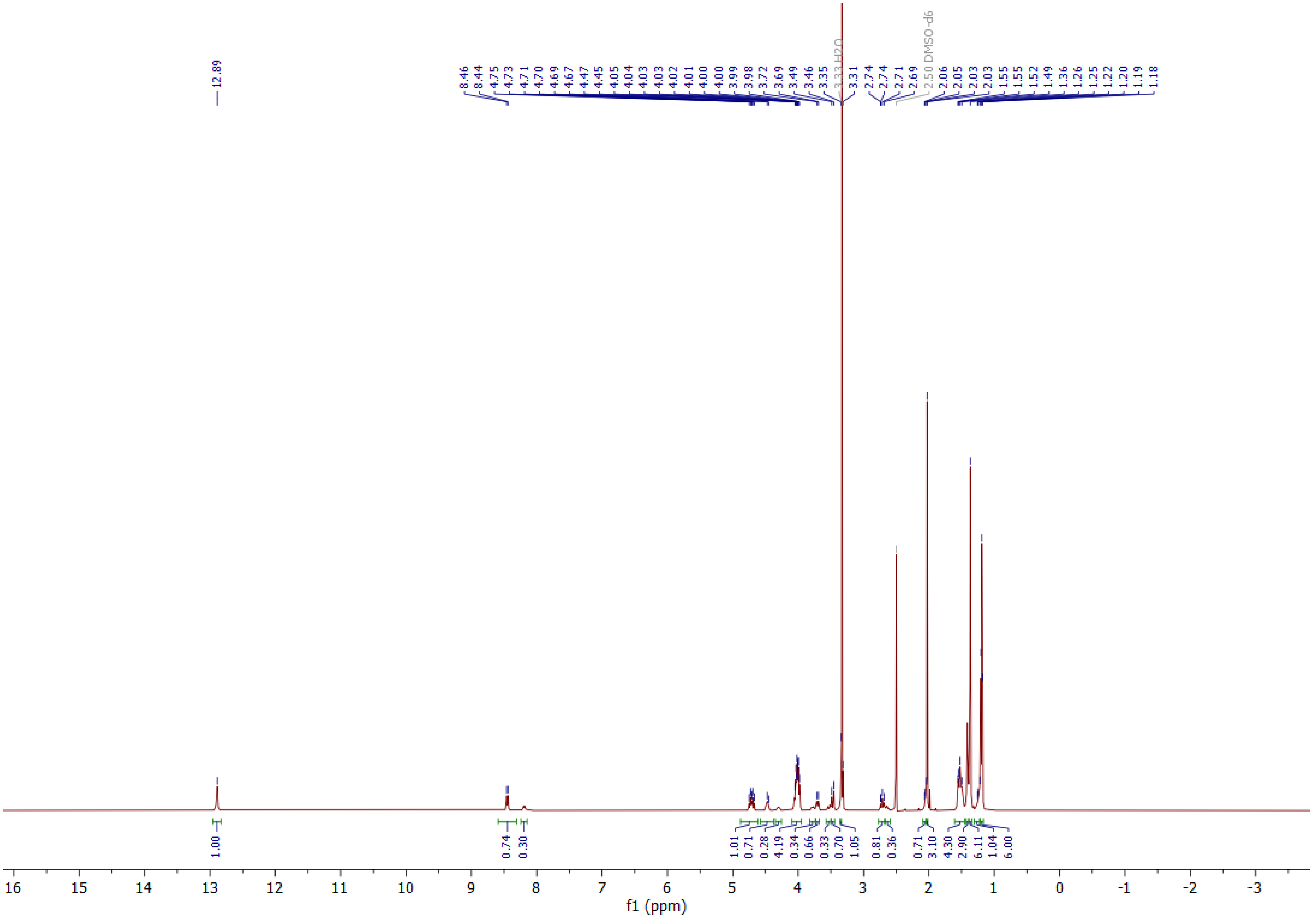

Figure S16. ^{1}H NMR spectrum of **4a** diastereomer 2.

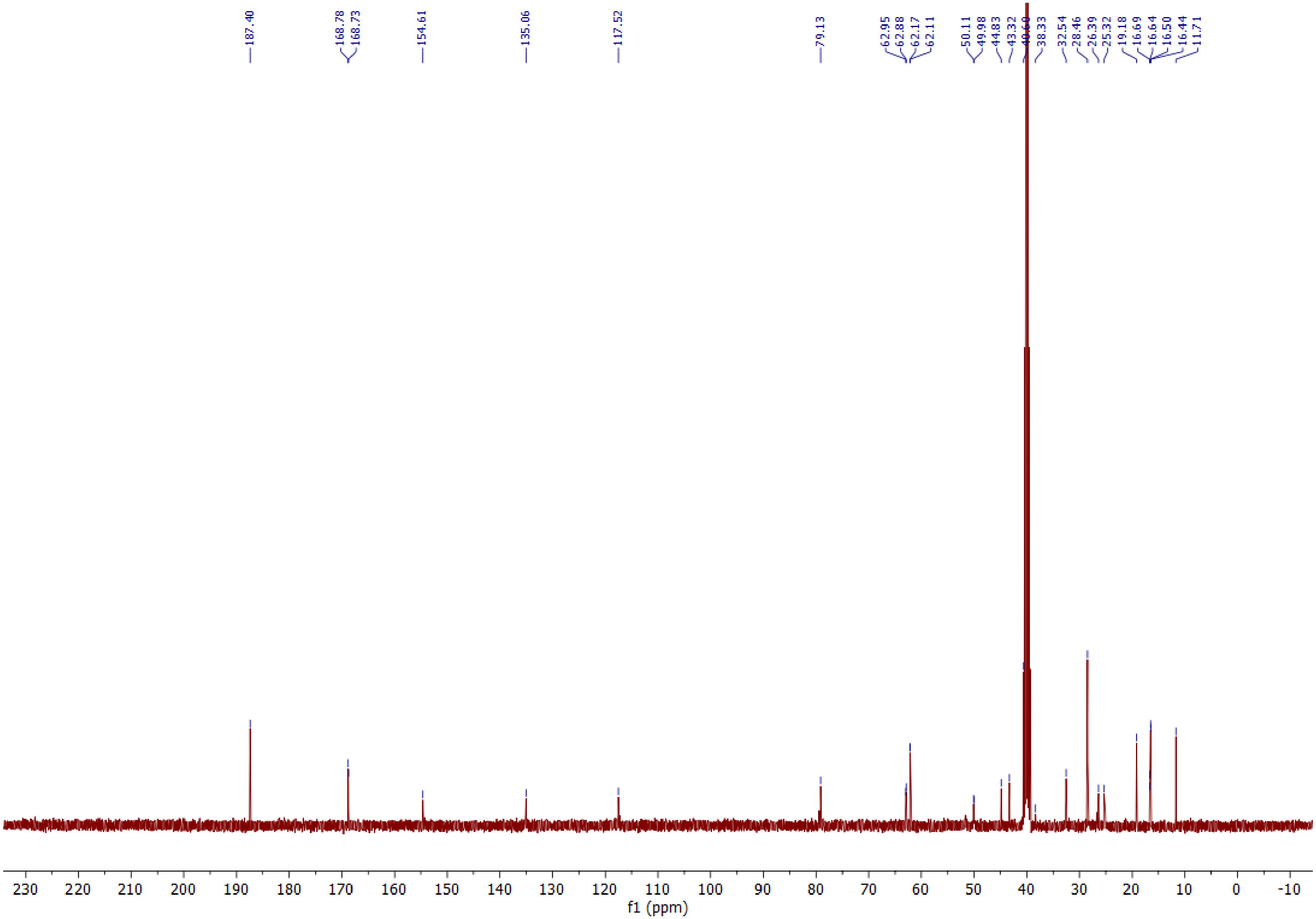

Figure S17. ^{13}C NMR spectrum of **4a** diastereomer 2.

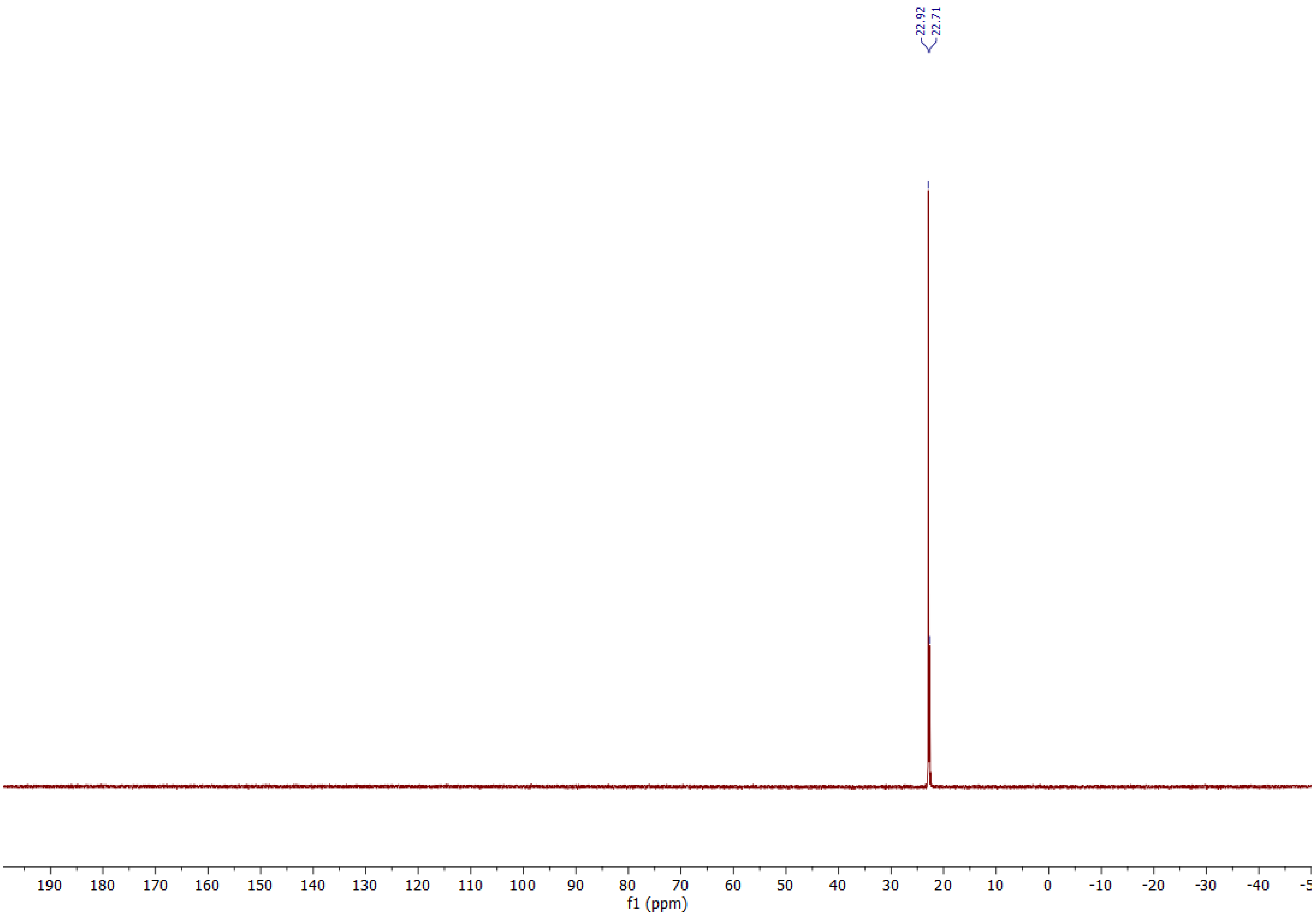

Figure S18. ^{31}P NMR spectrum of **4a** diastereomer 2.

Tert-butyl 2-((diethoxyphosphoryl)(thiophene-2-carboxamido)methyl)piperidine-1-carboxylate **(4b)**

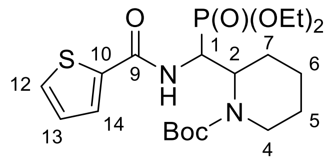

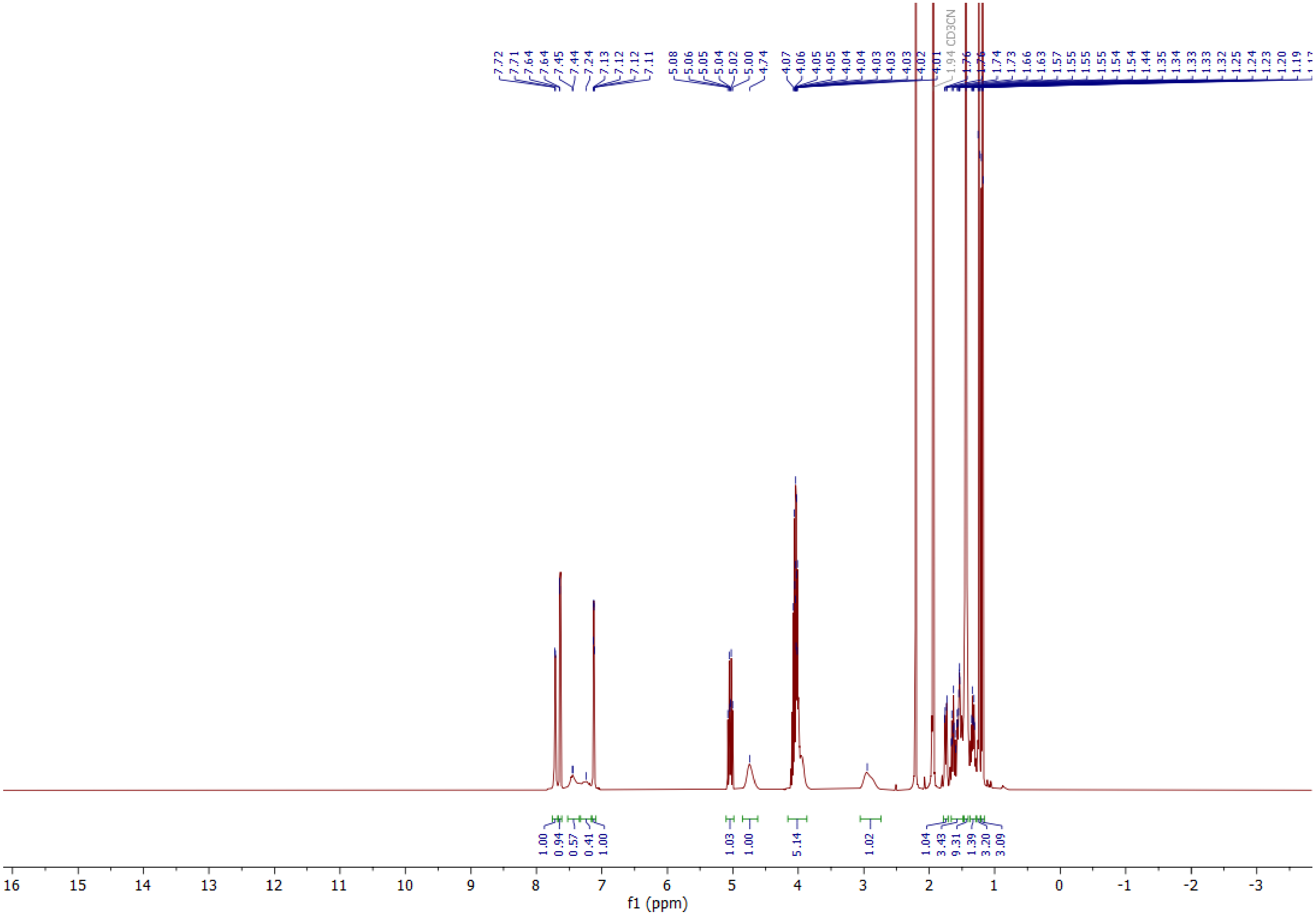

Figure S19. ^{1}H NMR spectrum of **4b** diastereomer 1.

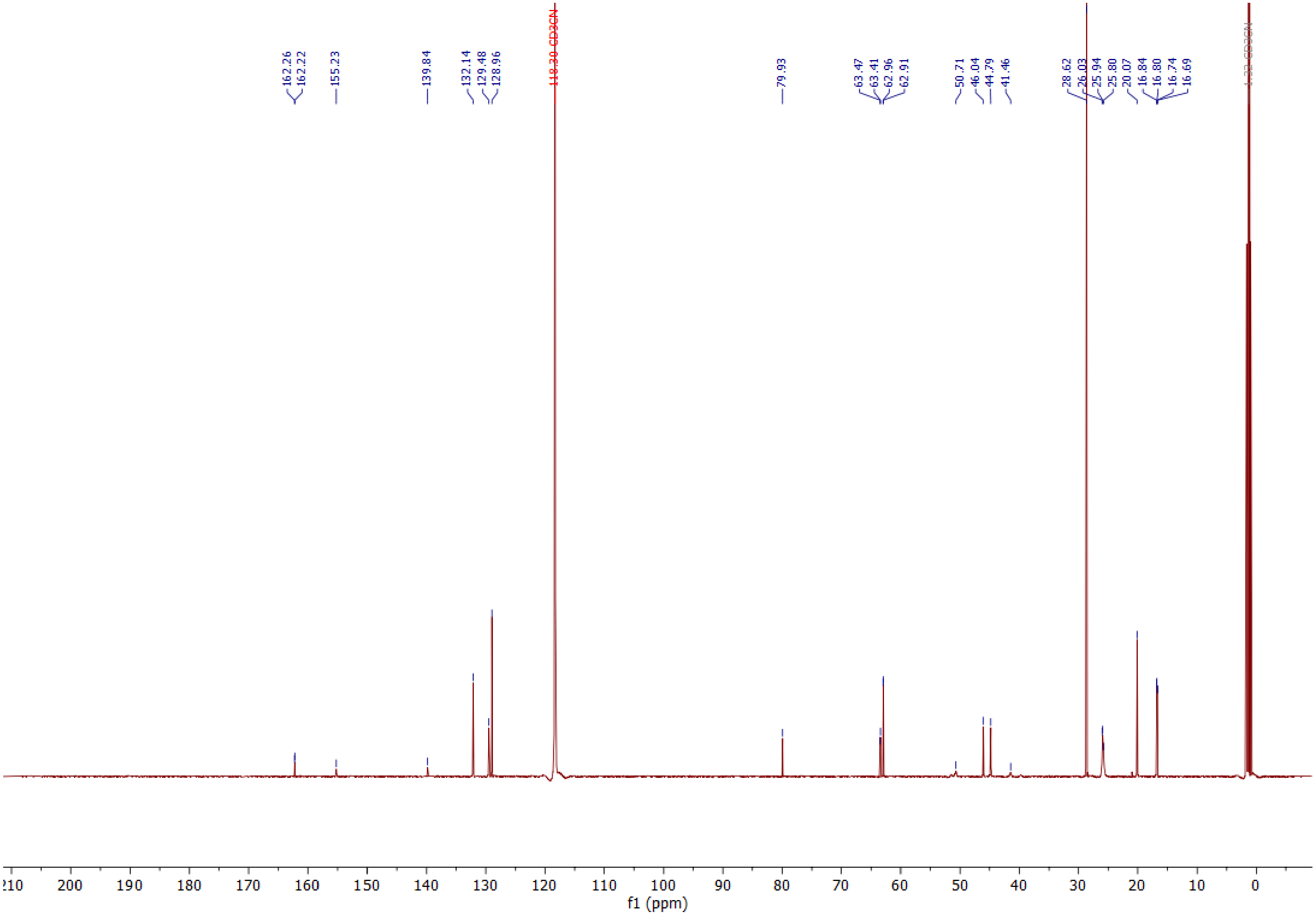

Figure S20. ^{13}C NMR spectrum of **4b** diastereomer 1.

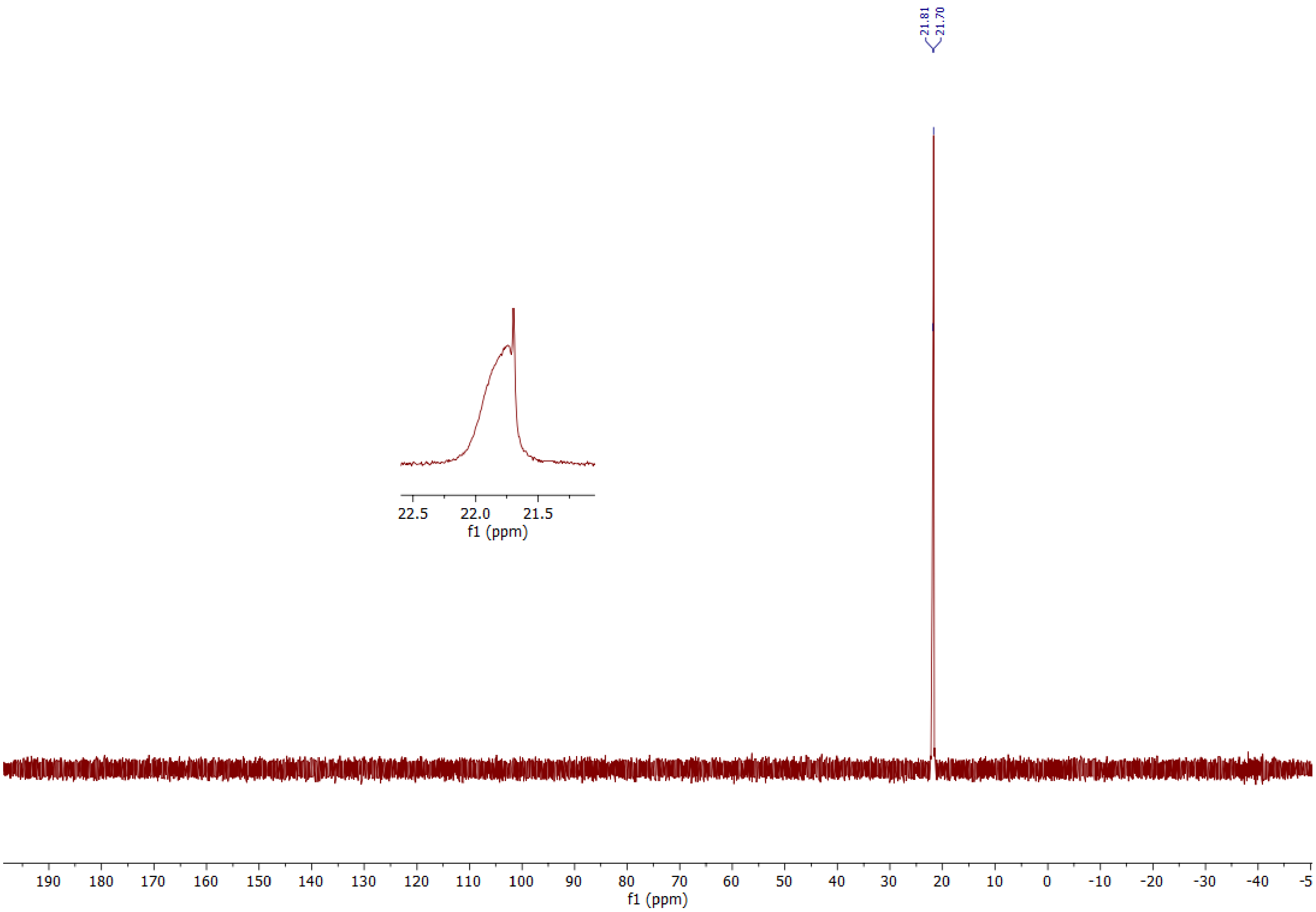

Figure S21. ^{31}P NMR spectrum of **4b** diastereomer 1.

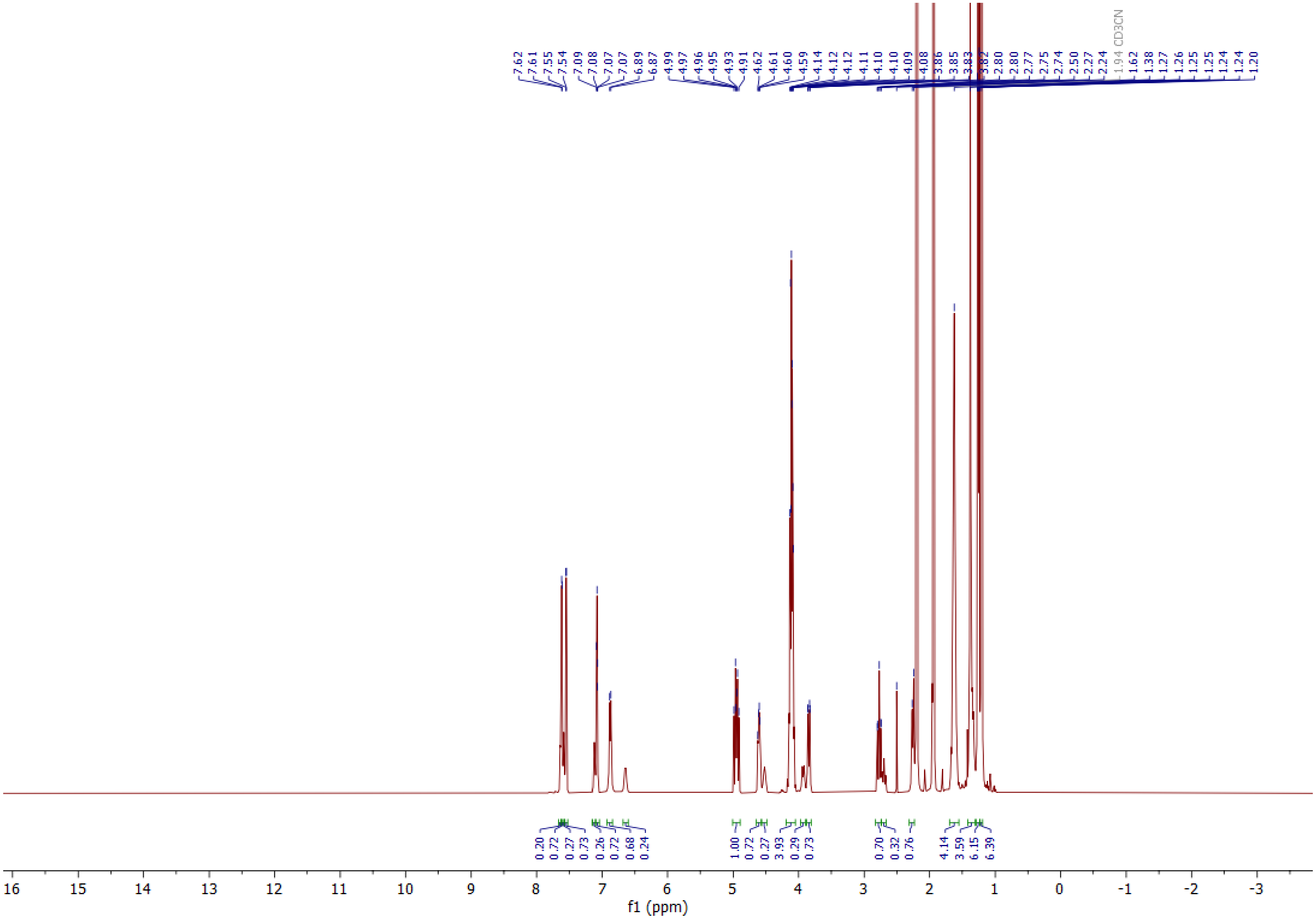

Figure S22. ^{1}H NMR spectrum of **4b** diastereomer 2.

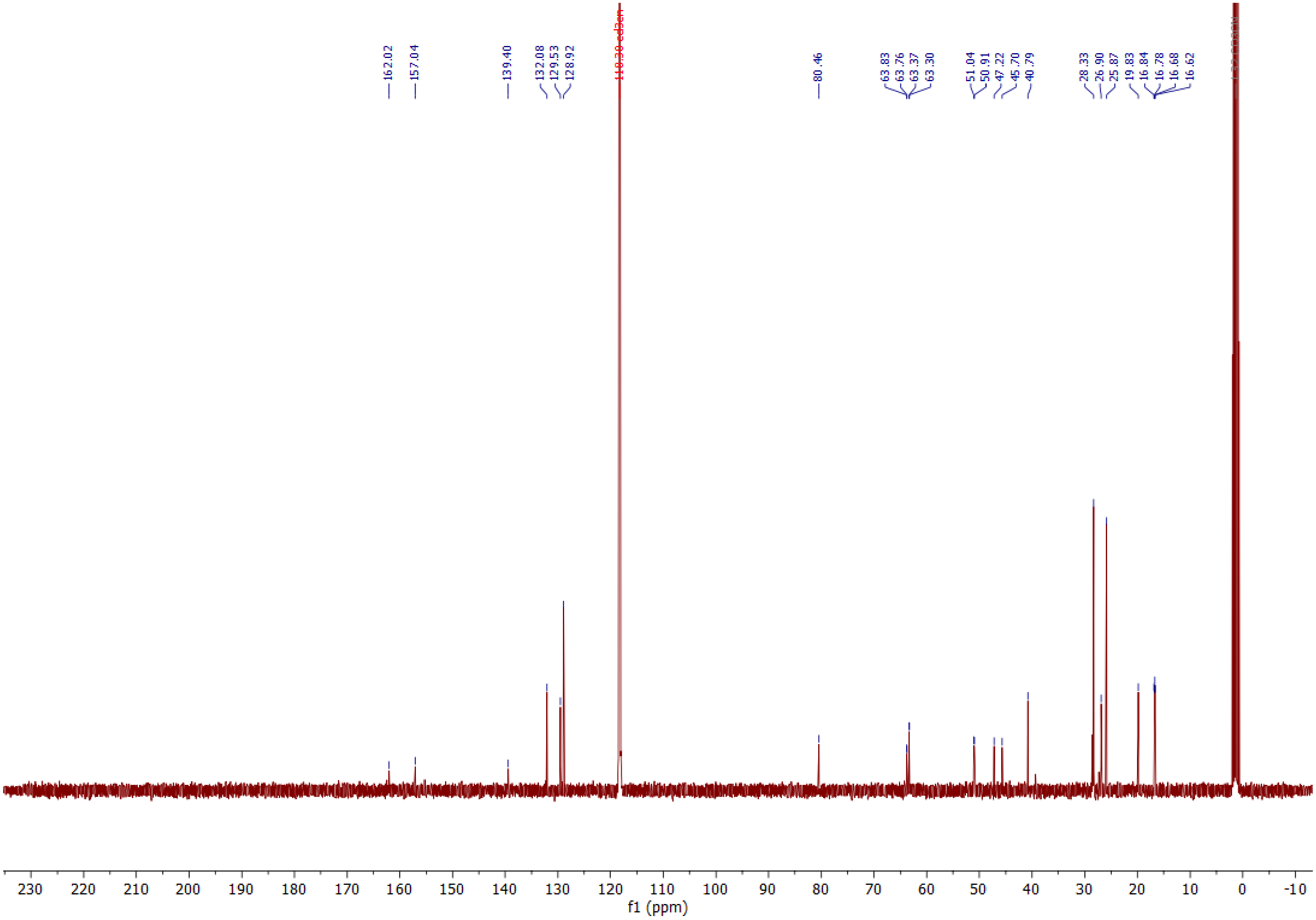

Figure S23. ^{13}C NMR spectrum of **4b** diastereomer 2.

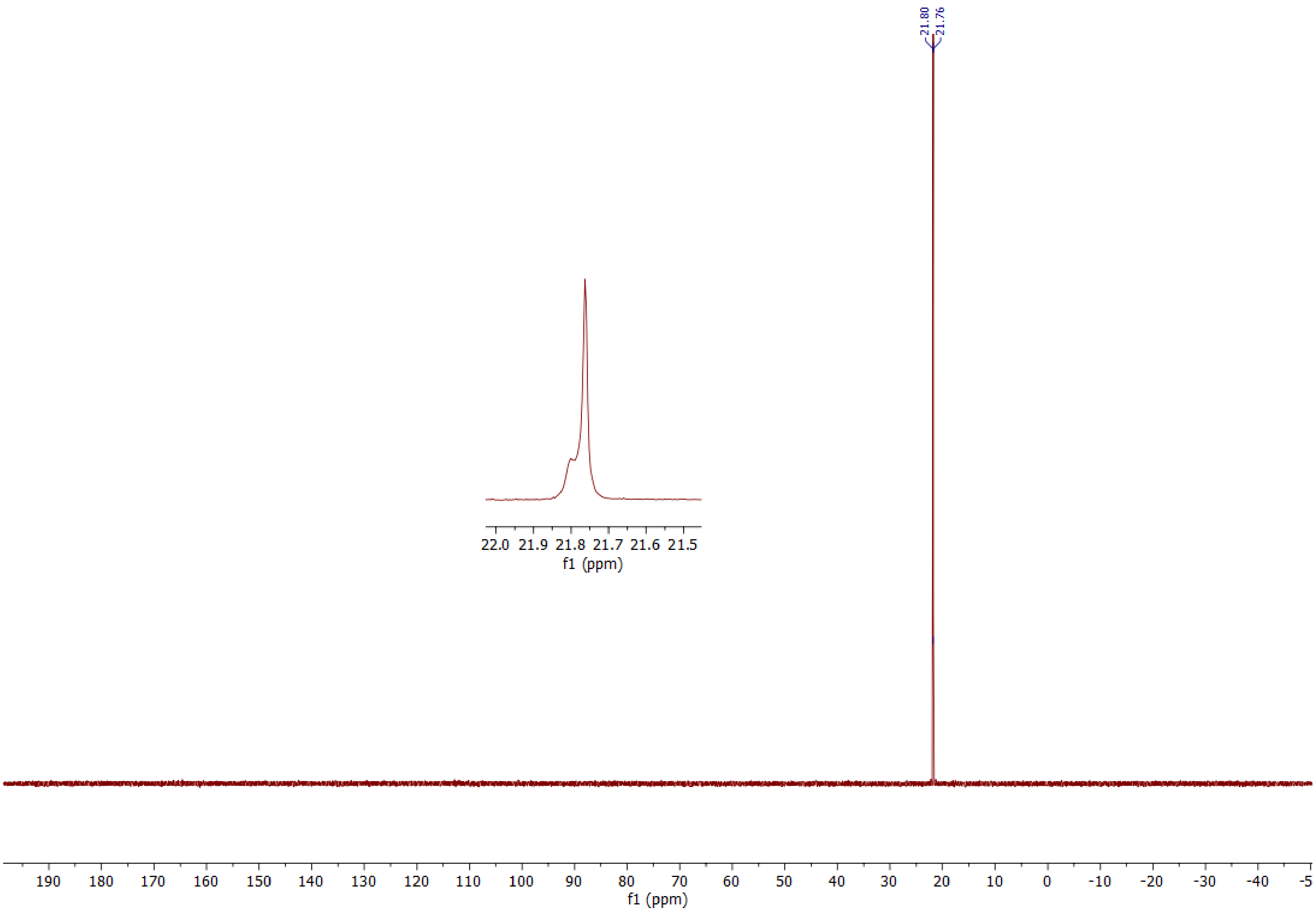

Figure S24. ^{31}P NMR spectrum of **4b** diastereomer 2.

Tert-butyl 2-((diethoxyphosphoryl)(thiophene-3-carboxamido)methyl)piperidine-1-carboxylate **(4c)**

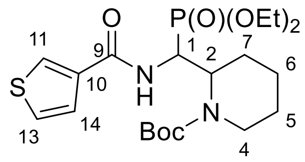

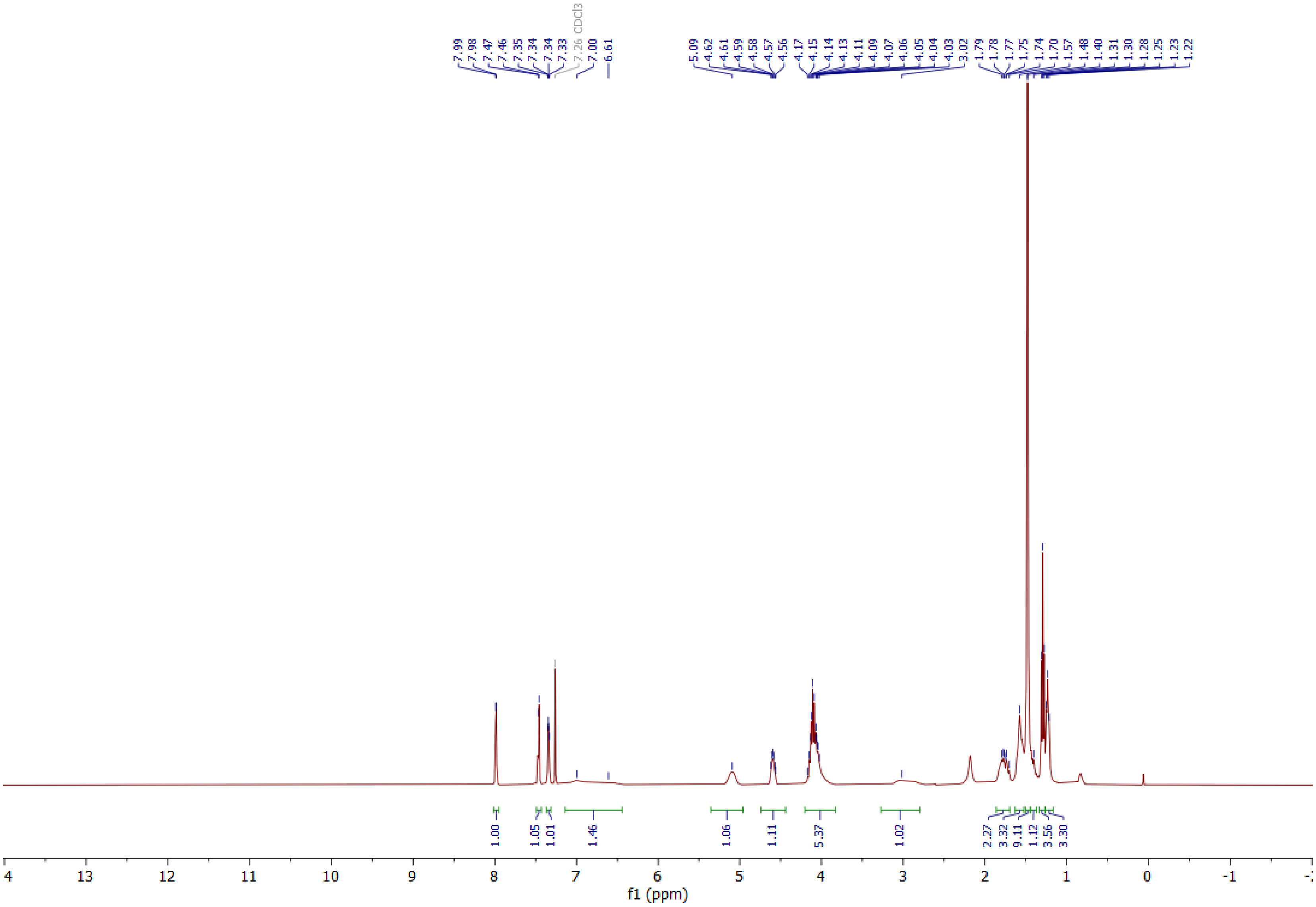

Figure S25. ^{1}H NMR spectrum of **4c** diastereomer 1.

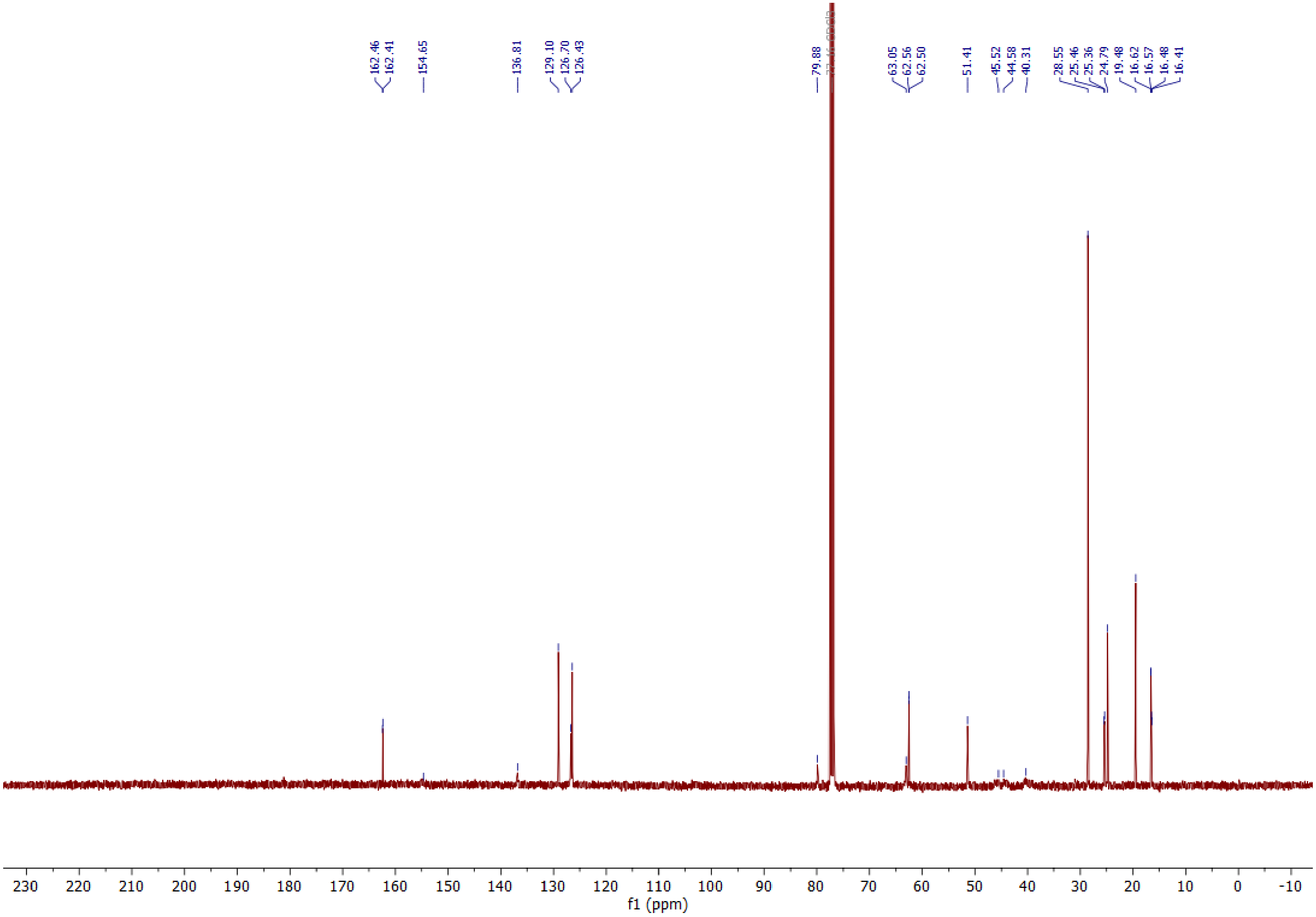

Figure S26. ^{13}C NMR spectrum of **4c** diastereomer 1.

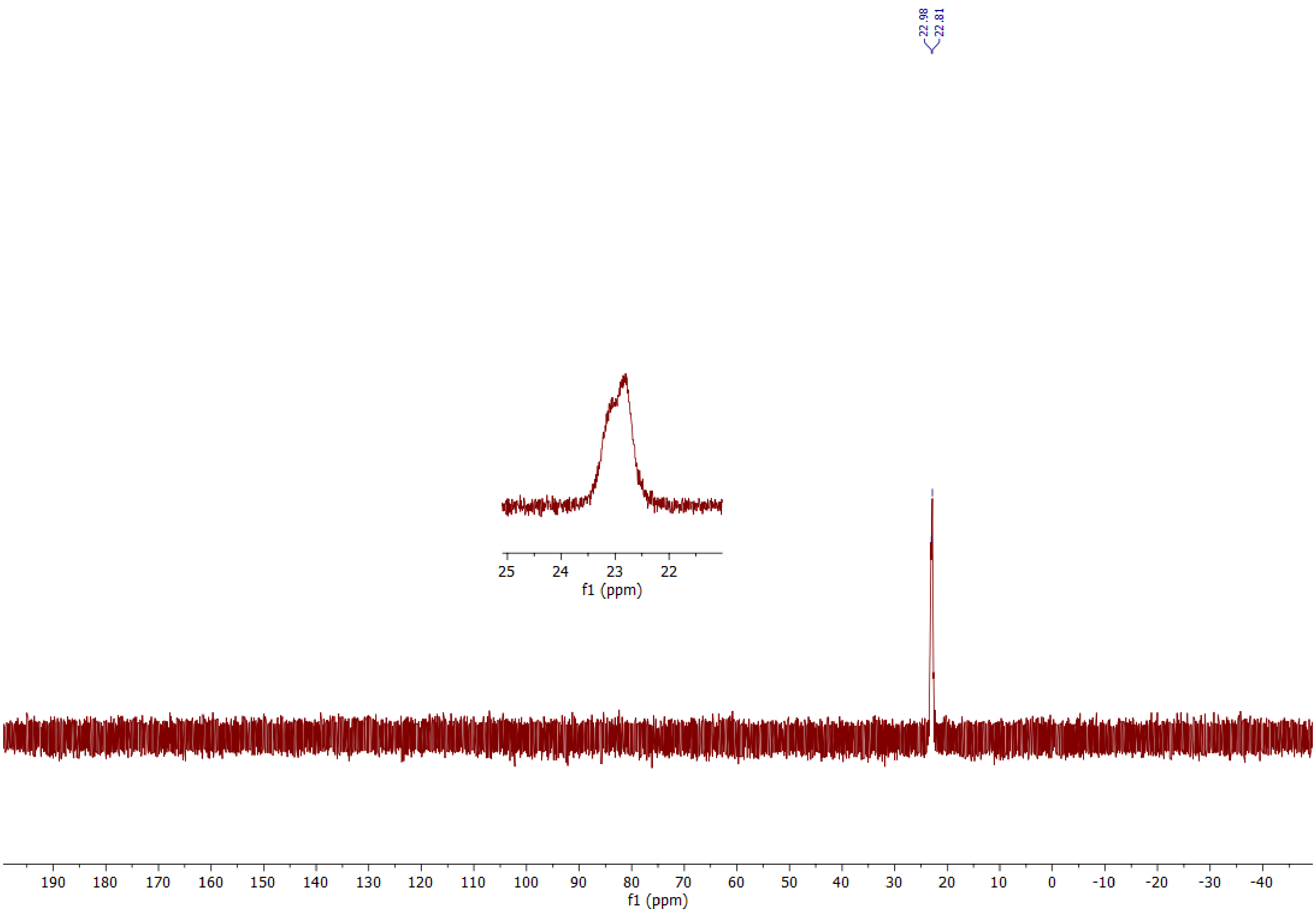

Figure S27. ^{31}P NMR spectrum of **4c** diastereomer 1.

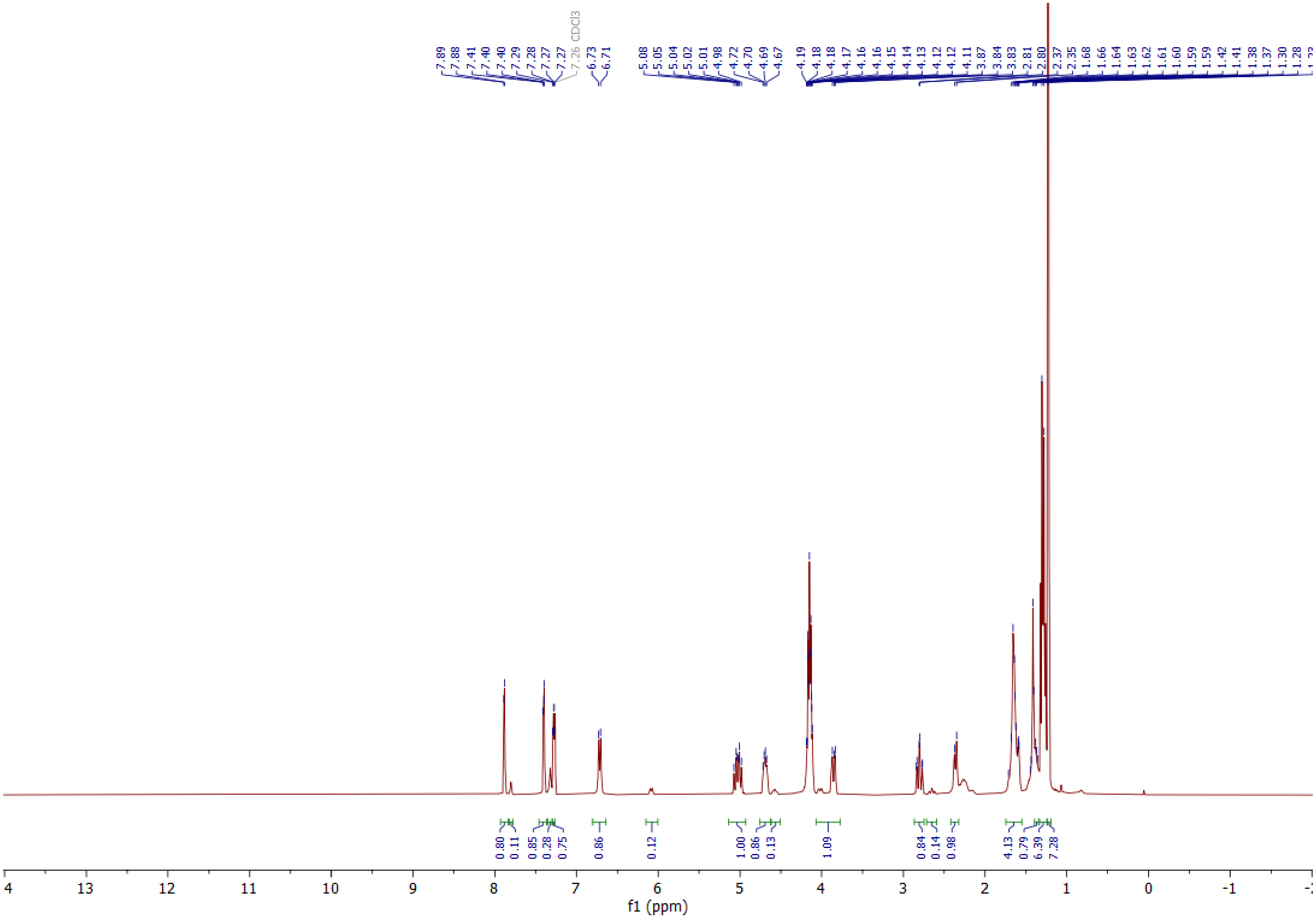

Figure S28. ^{1}H NMR spectrum of **4c** diastereomer 2.

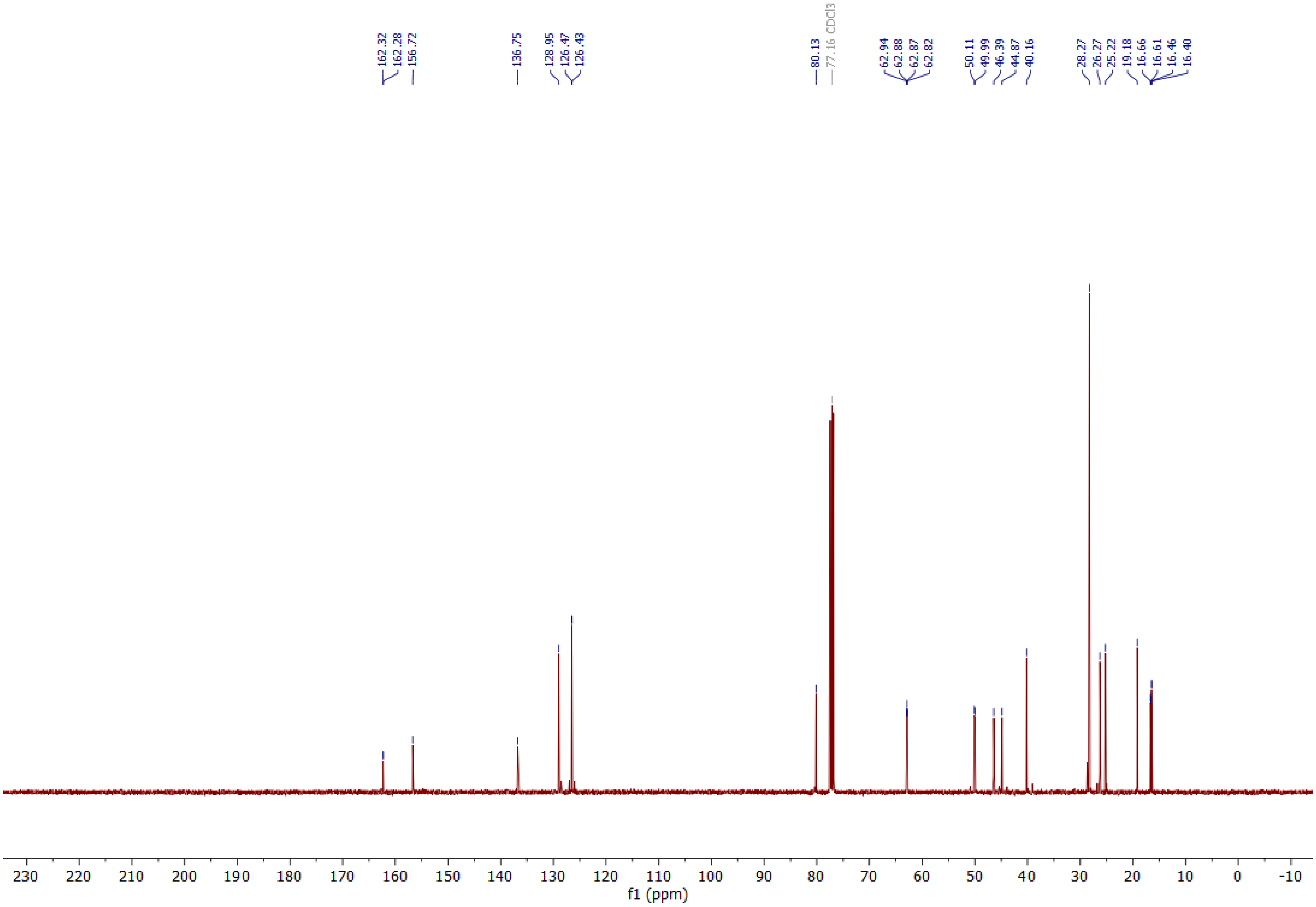

Figure S29. ^{13}C NMR spectrum of **4c** diastereomer 2.

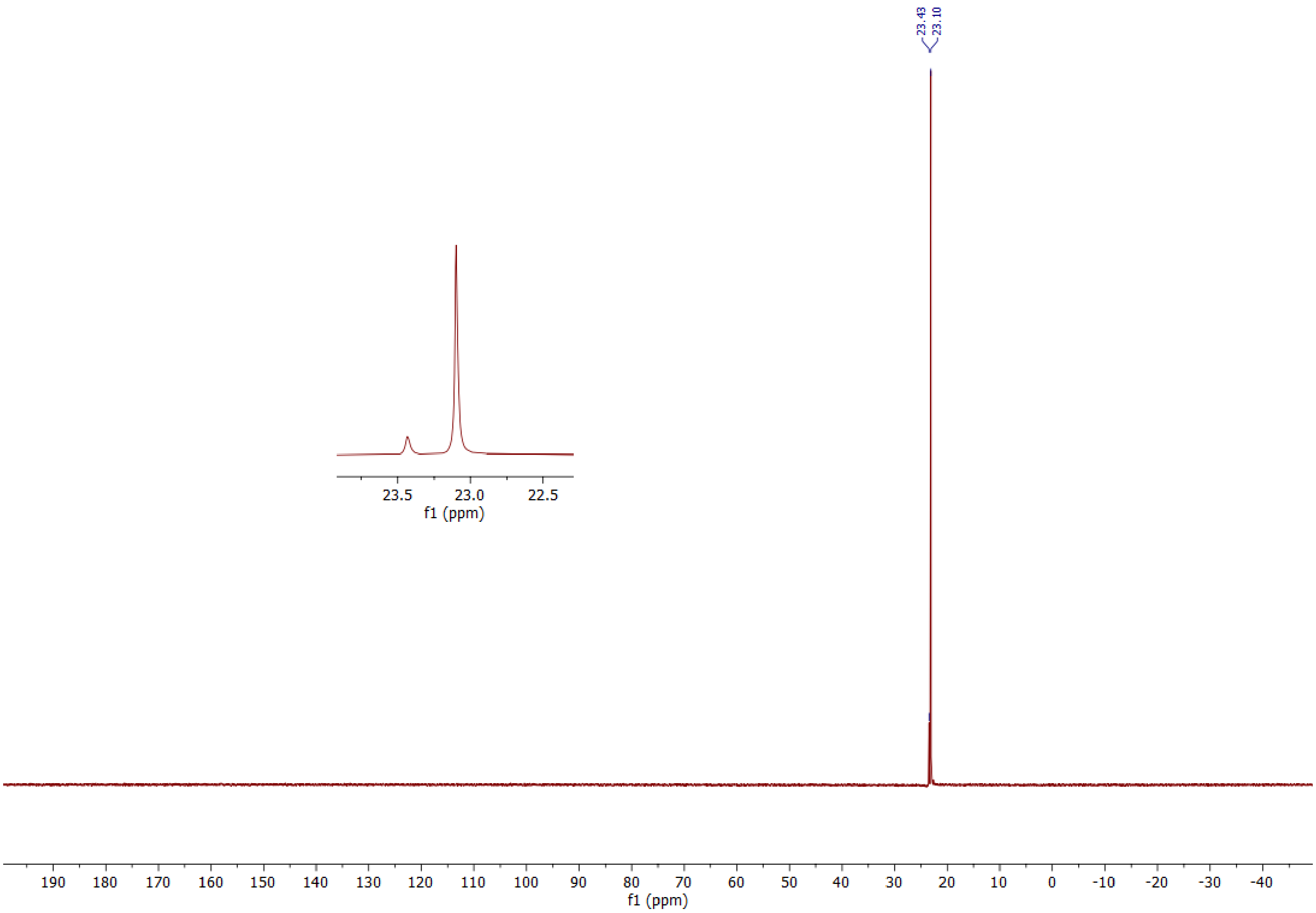

Figure S30. ^{31}P NMR spectrum of **4c** diastereomer 2.

Tert-butyl 2-((diethoxyphosphoryl)(4,5,6,7-tetrahydrobenzo[*b*]thiophene-2-carboxamido)methyl)piperidine-1-carboxylate (**4d**)

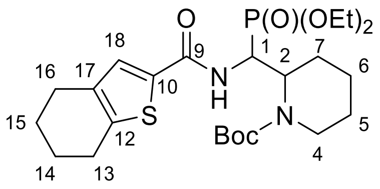

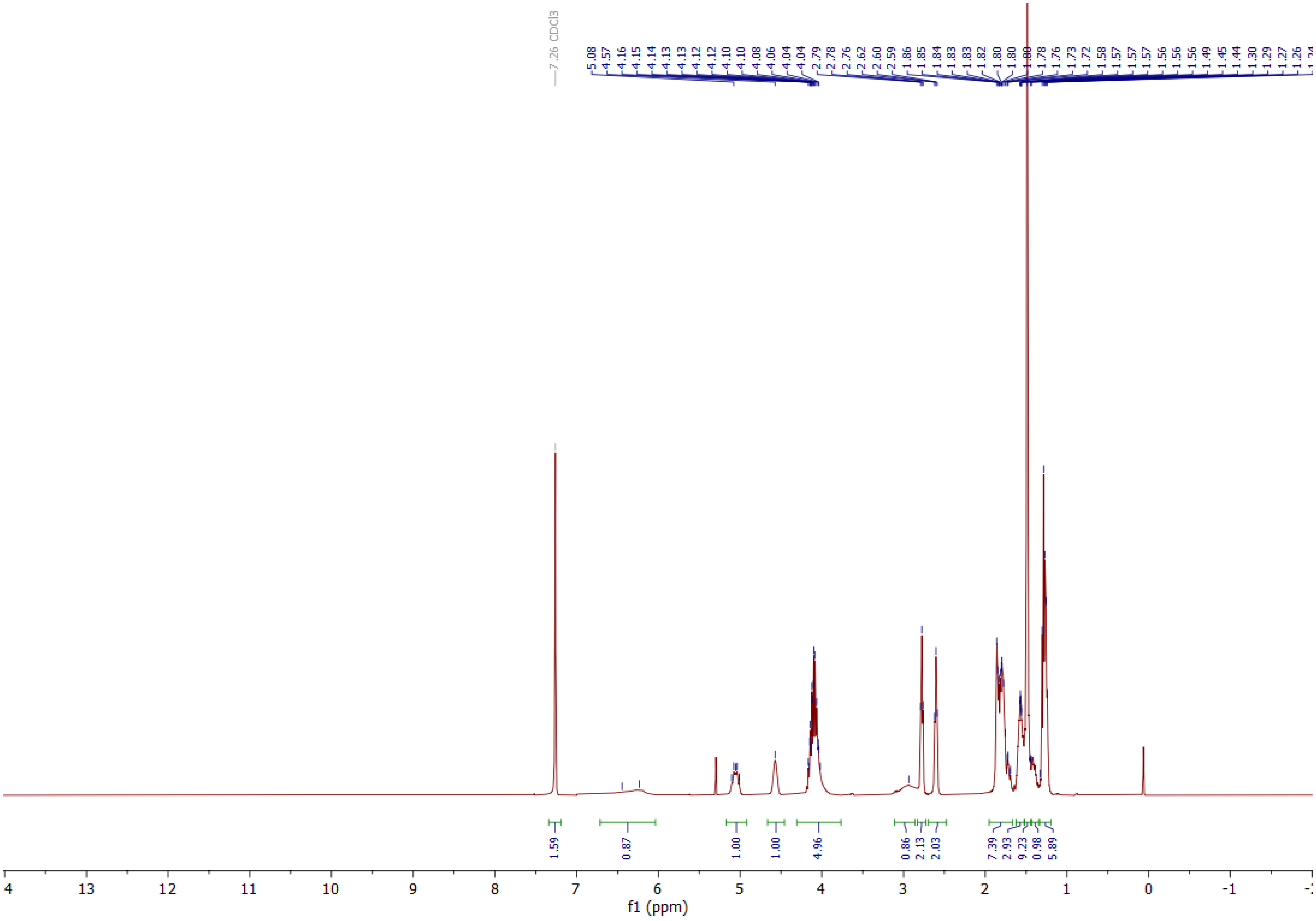

Figure S31. ^{1}H NMR spectrum of **4d** diastereomer 1.

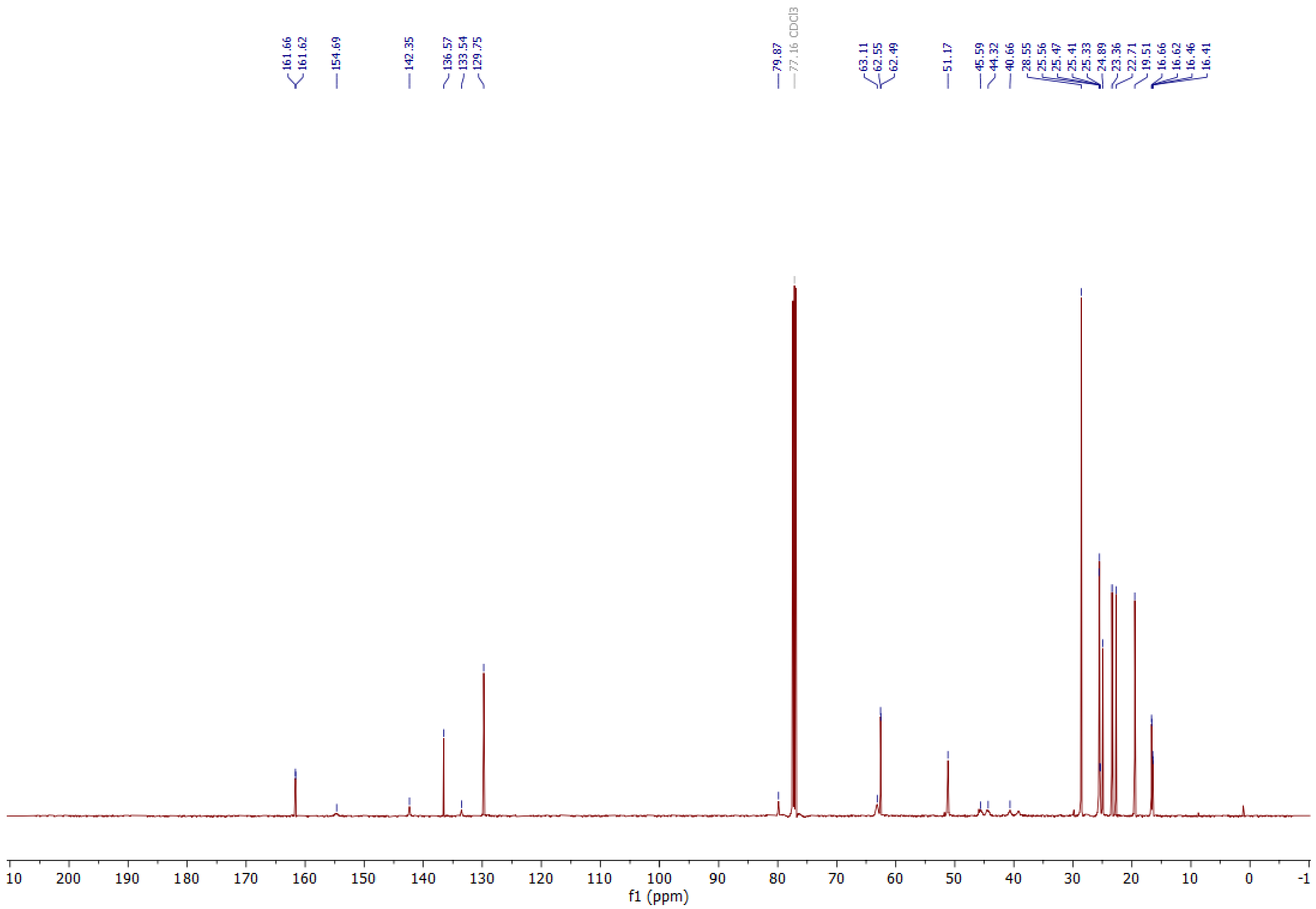

Figure S32. ^{13}C NMR spectrum of **4d** diastereomer 1.

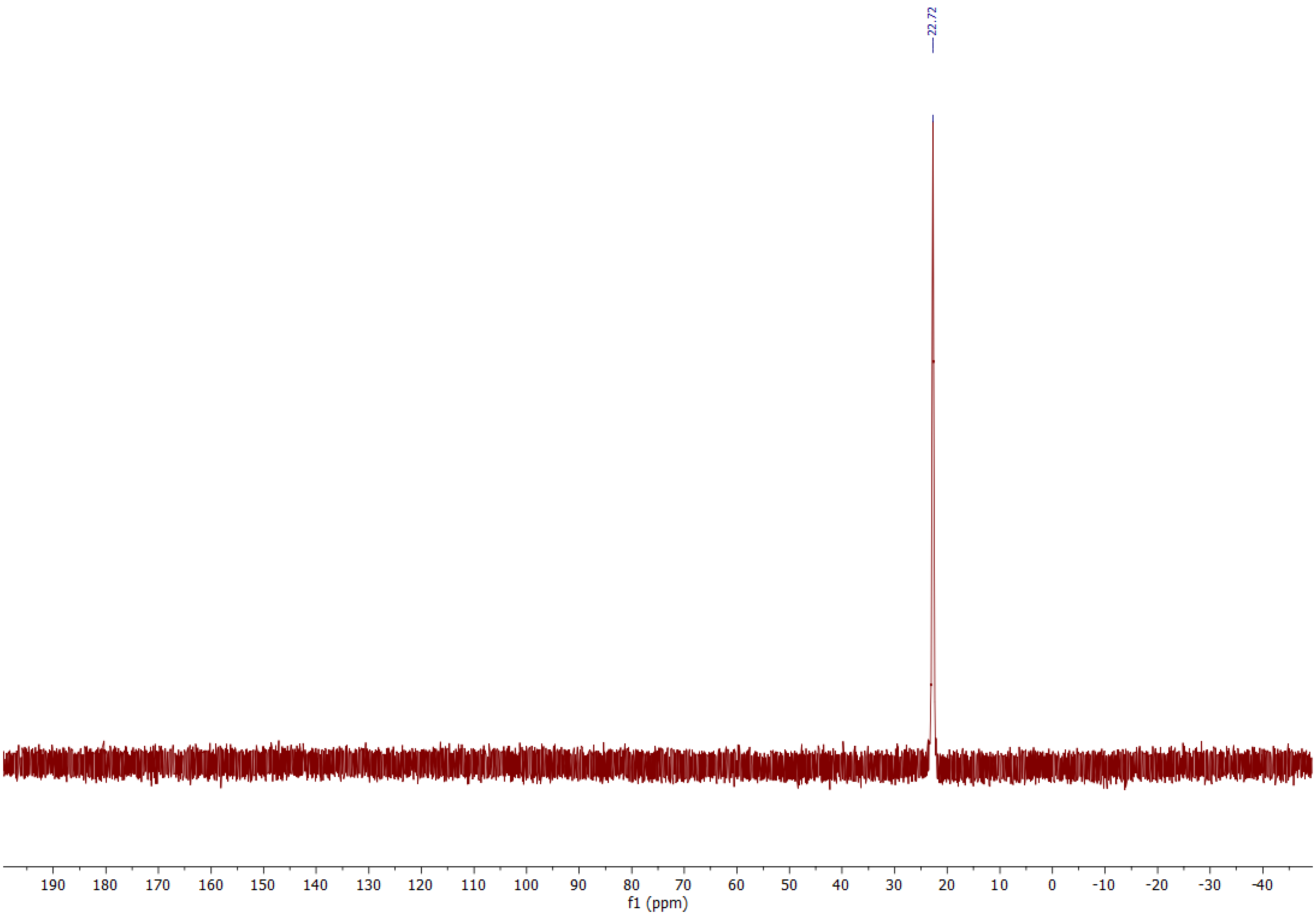

Figure S33. ^{31}P NMR spectrum of **4d** diastereomer 1.

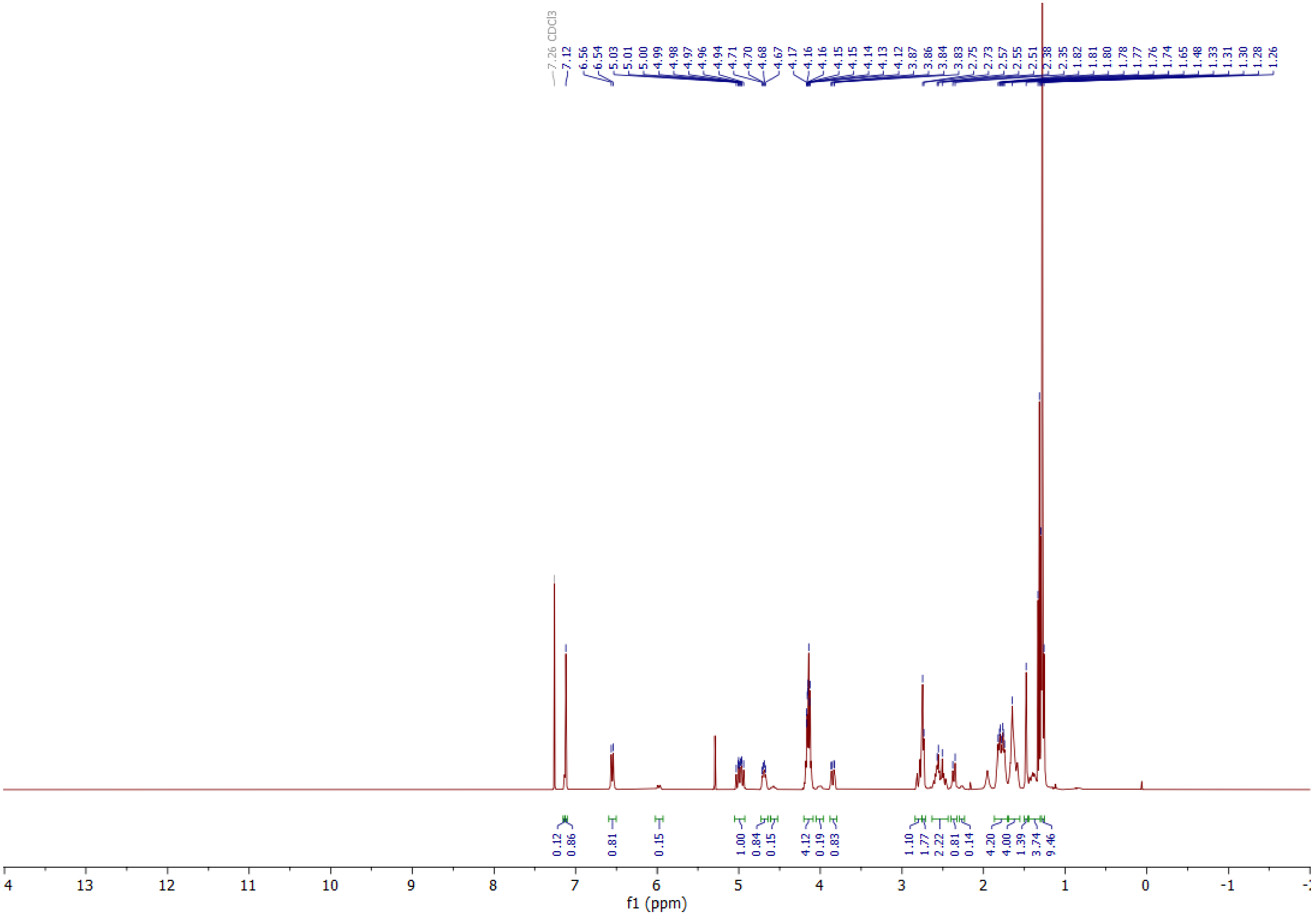

Figure S34. ^{1}H NMR spectrum of **4d** diastereomer 2.

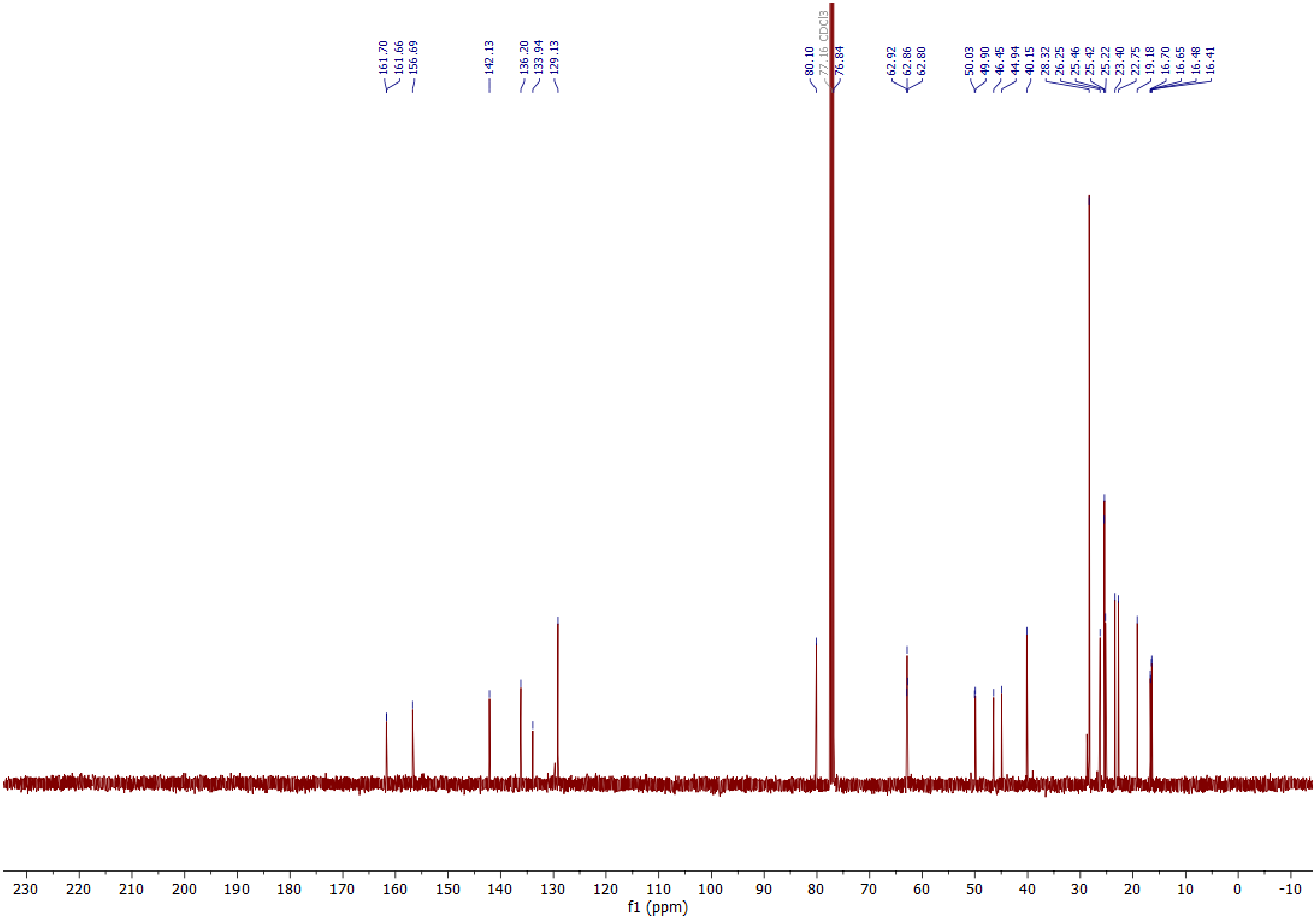

Figure S35. ^{13}C NMR spectrum of **4d** diastereomer 2.

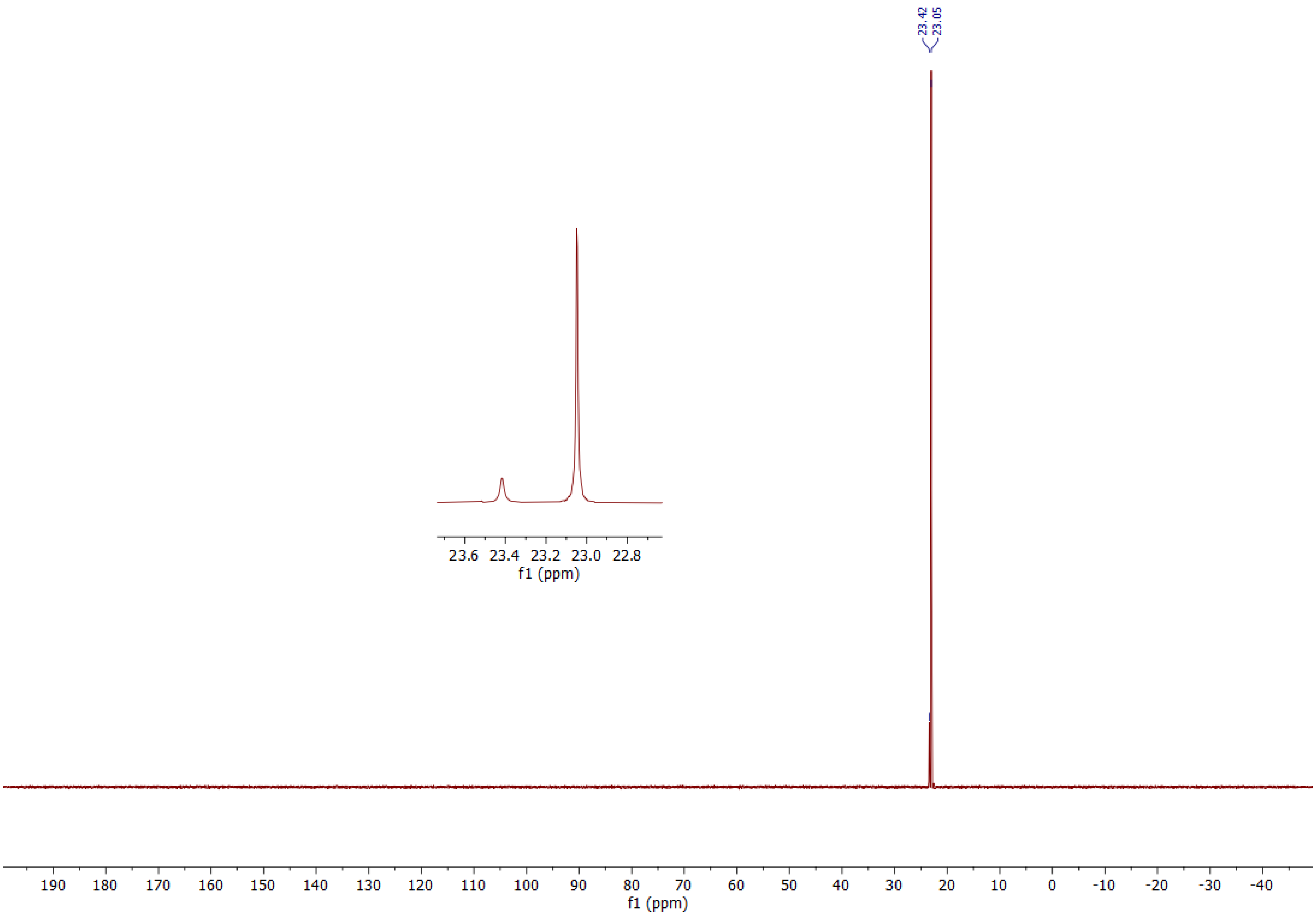

Figure S36. ^{31}P NMR spectrum of **4d** diastereomer 2.

Tert-butyl 2-((diethoxyphosphoryl)(4,5,6,7-tetrahydrobenzo[*b*]thiophene-3-carboxamido)methyl)piperidine-1-carboxylate (**4e**)

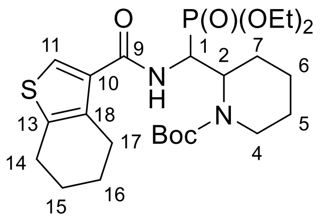

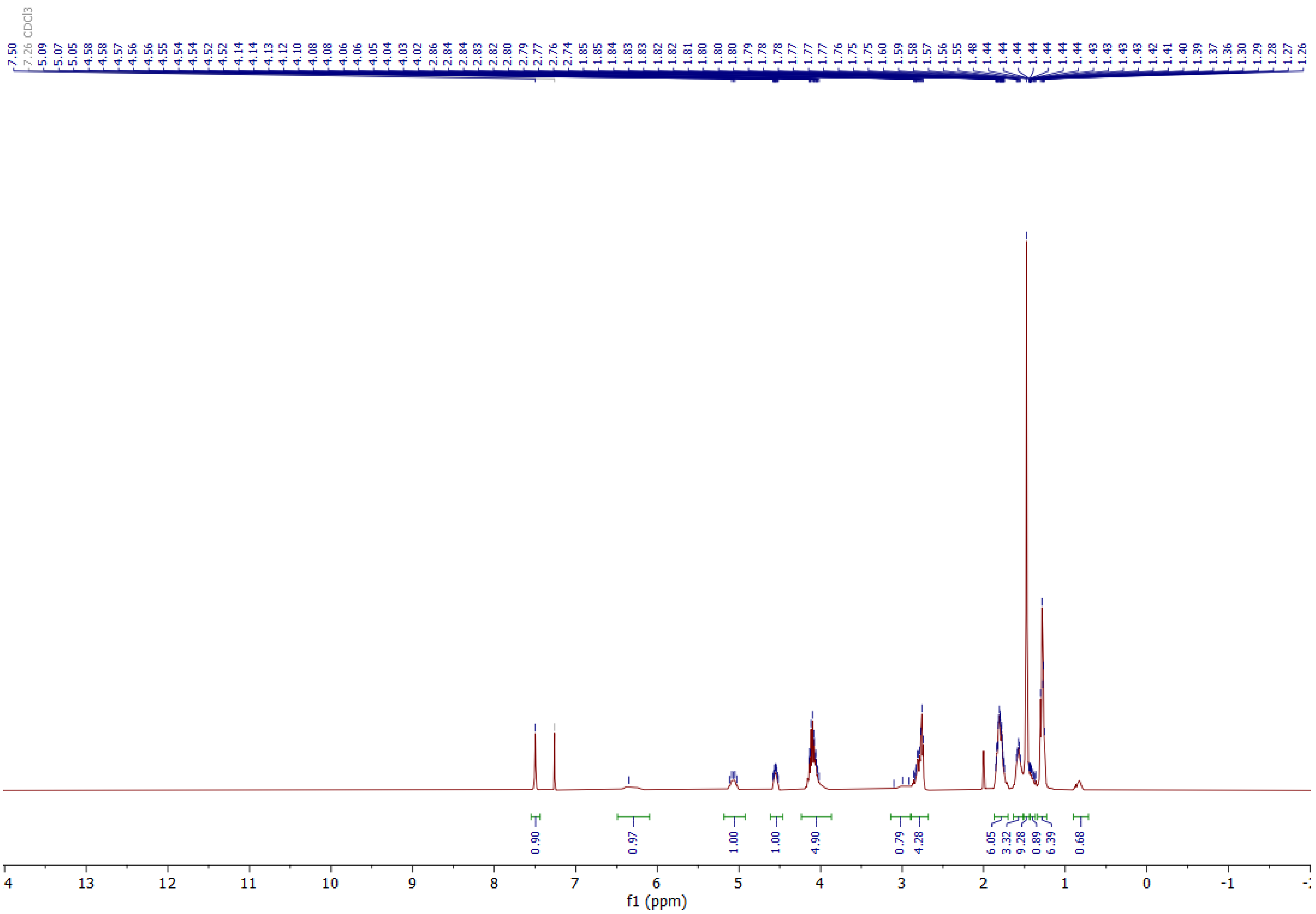

Figure S37. ^{1}H NMR spectrum of **4e** diastereomer 1.

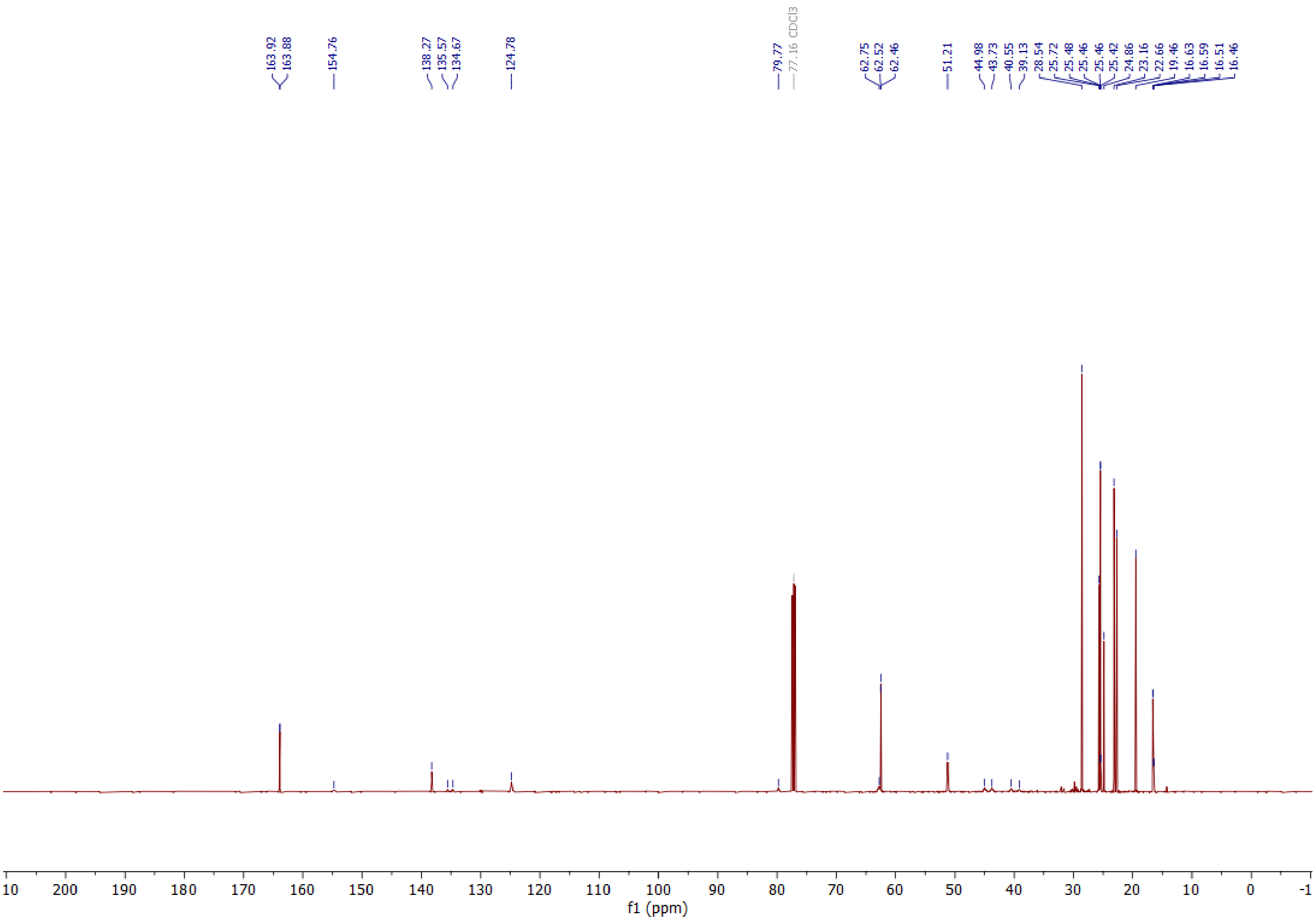

Figure S38. ^{13}C NMR spectrum of **4e** diastereomer 1.

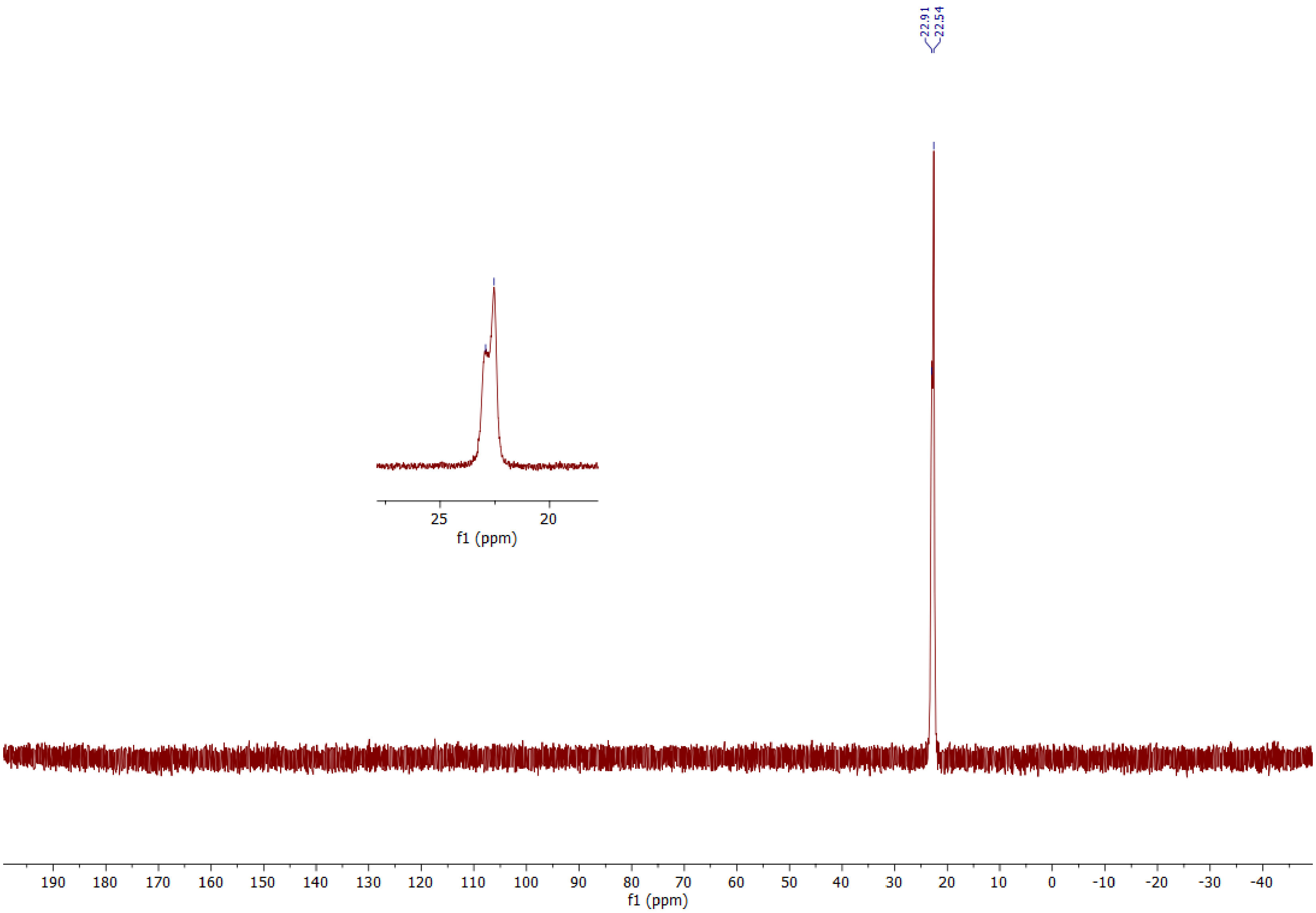

Figure S39. ^{31}P NMR spectrum of **4e** diastereomer 1.

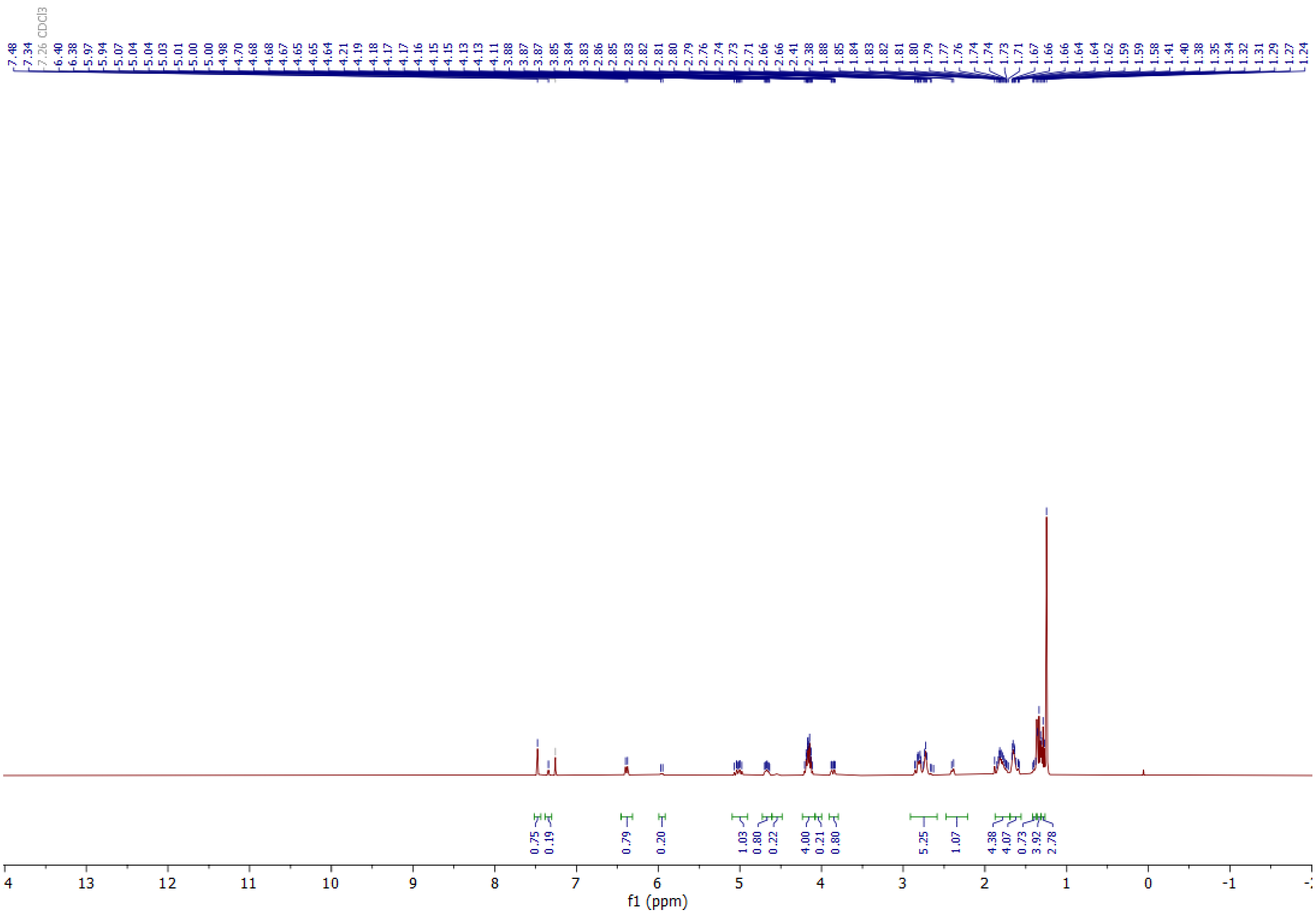

Figure S40. ^{1}H NMR spectrum of **4e** diastereomer 2.

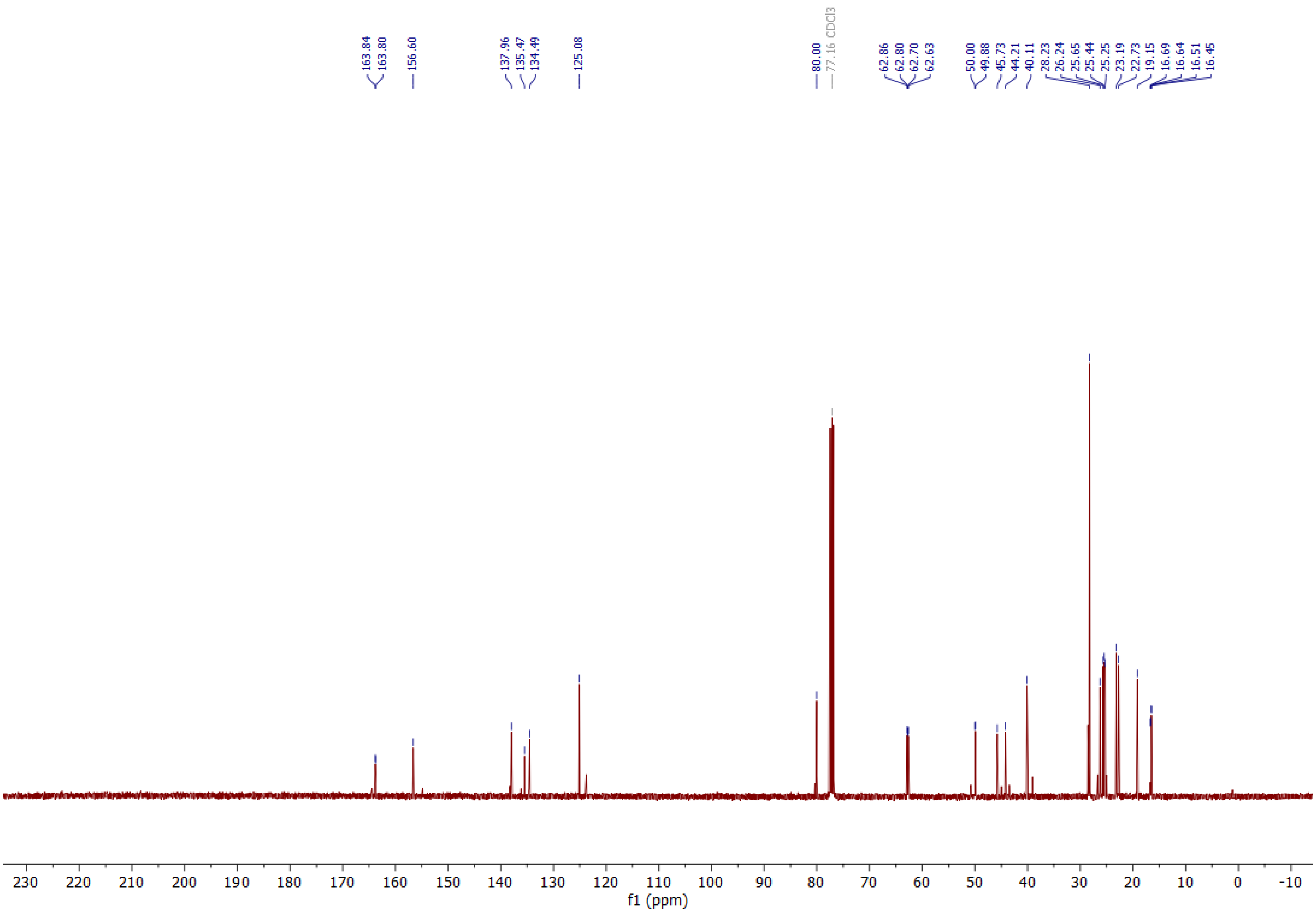

Figure S41. ^{13}C NMR spectrum of **4e** diastereomer 2.

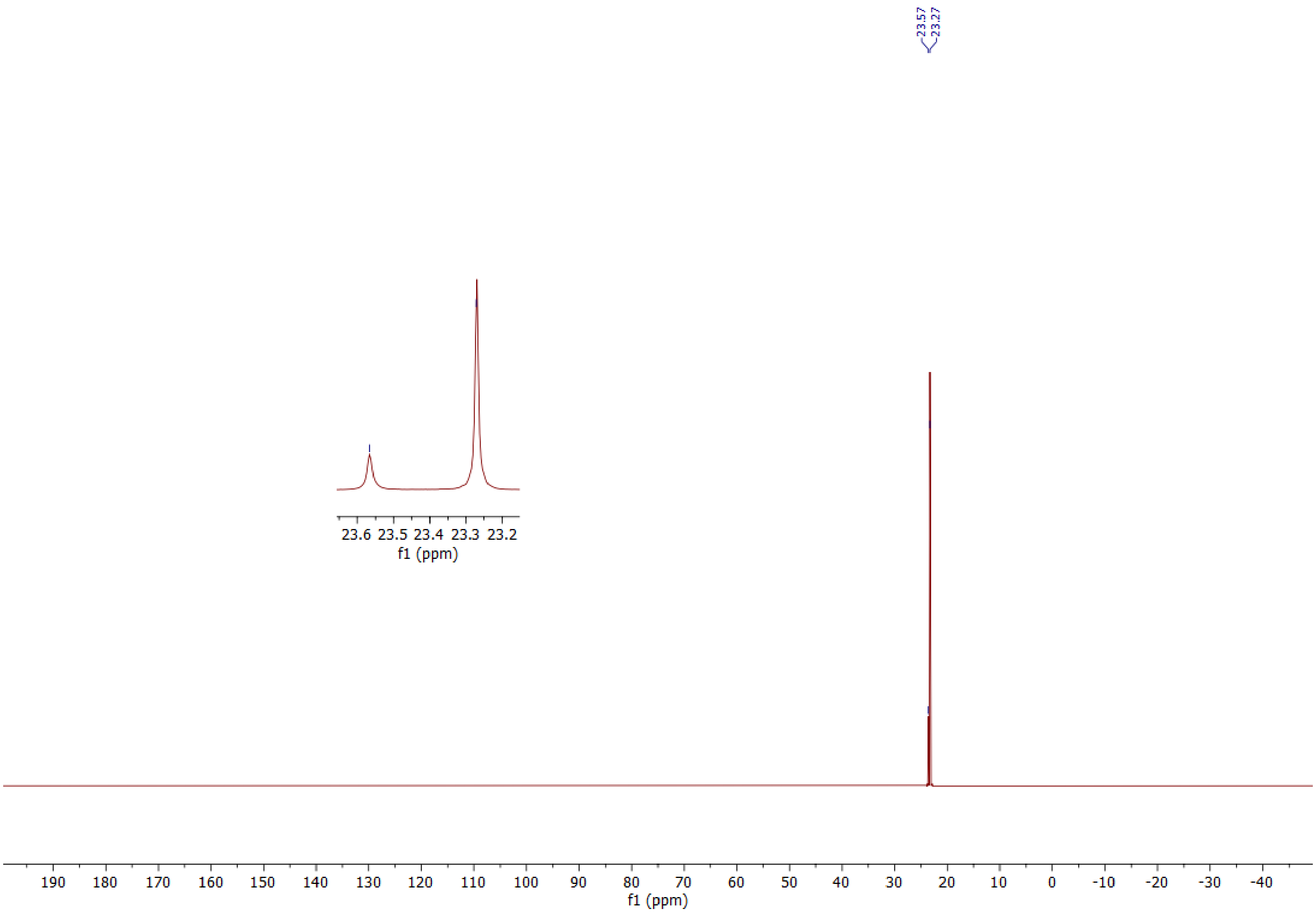

Figure S42. ^{31}P NMR spectrum of **4e** diastereomer 2.

Tert-butyl 2-((diethoxyphosphoryl)(4,5,6,7-tetrahydrobenzo[*c*]thiophene-1-carboxamido)methyl)piperidine-1-carboxylate (**4f**)

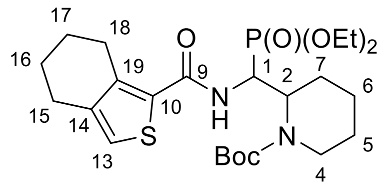

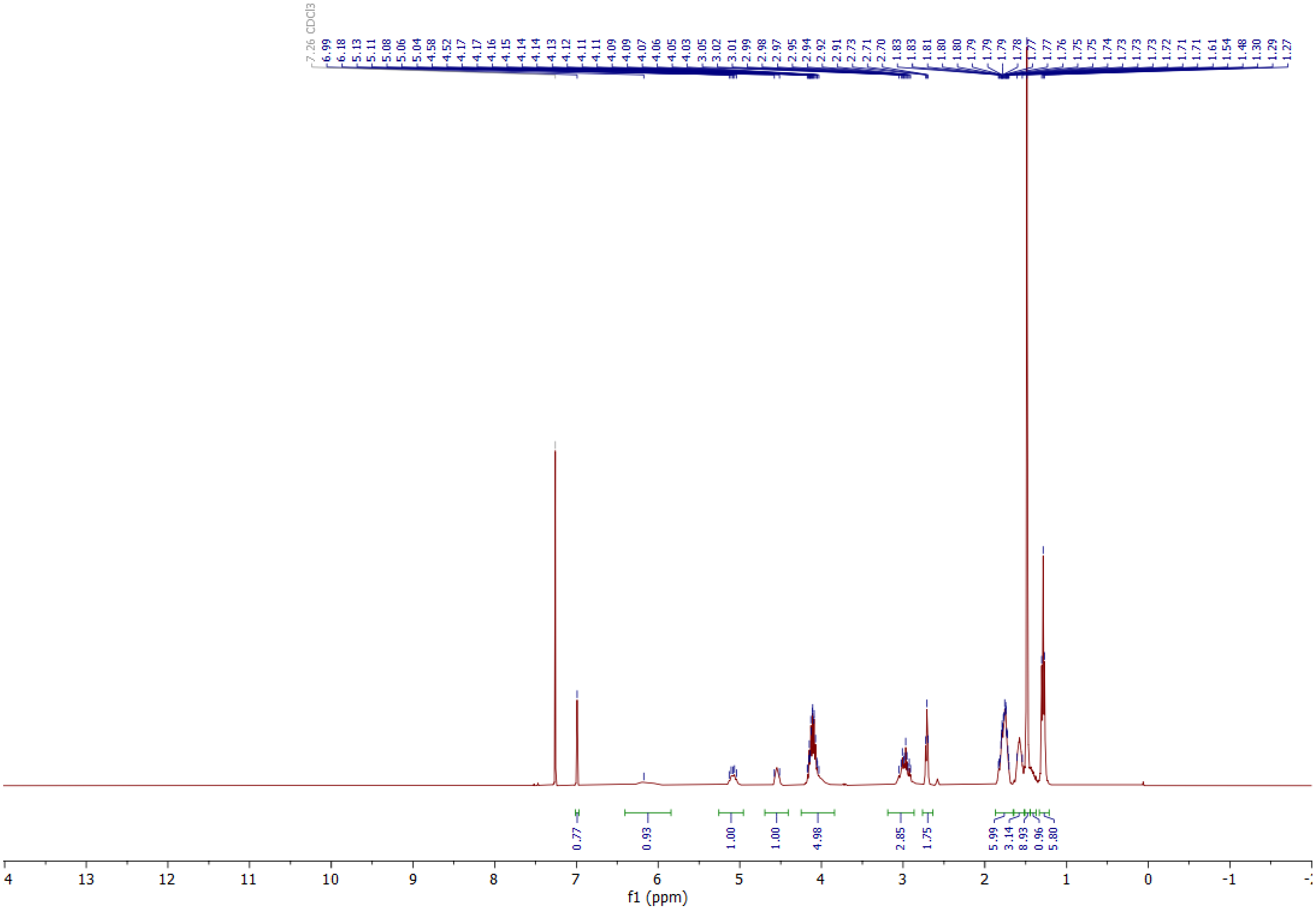

Figure S43. ^{1}H NMR spectrum of **4f** diastereomer 1.

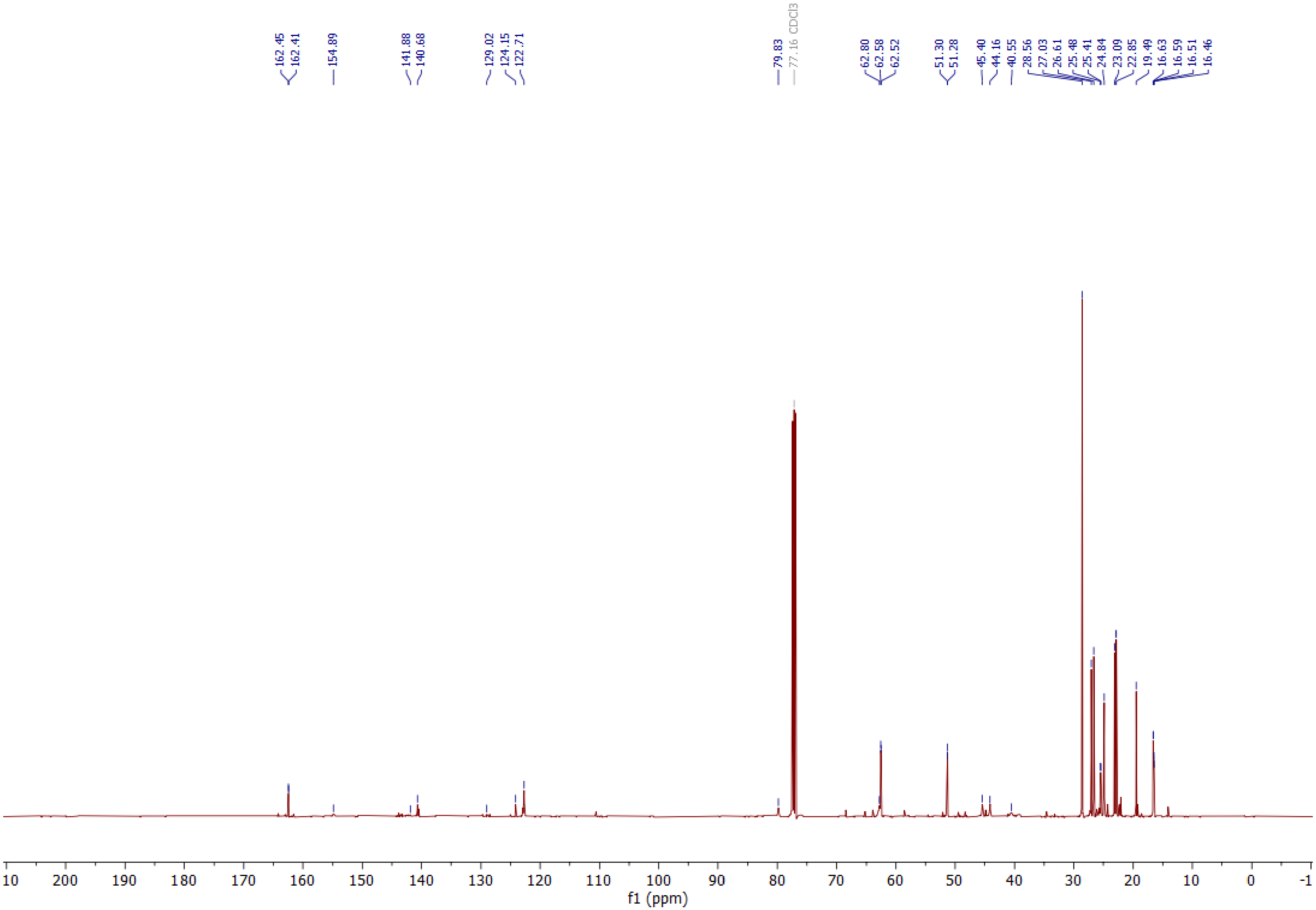

Figure S44. ^{13}C NMR spectrum of **4f** diastereomer 1.

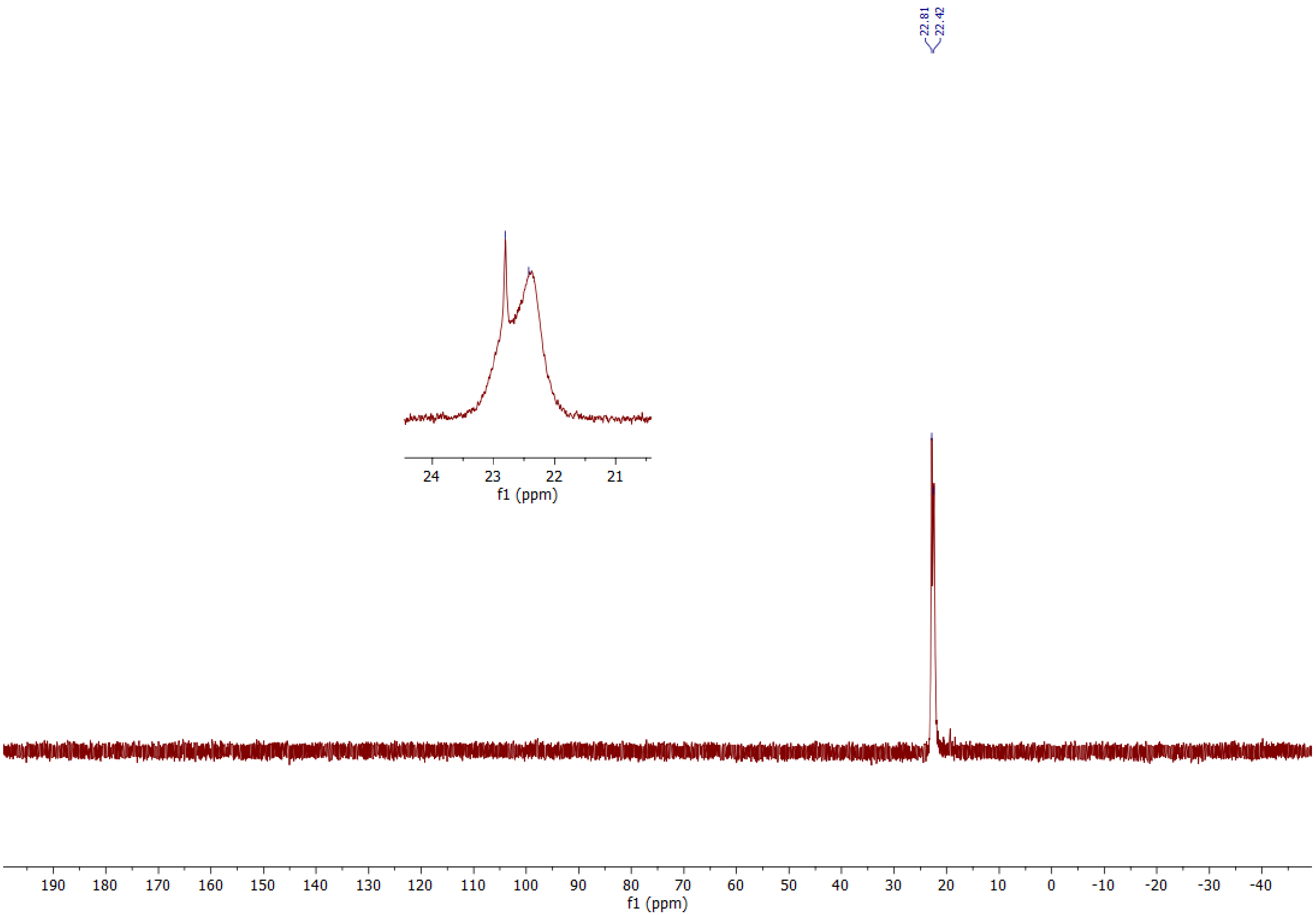

Figure S45. ^{31}P NMR spectrum of **4f** diastereomer 1.

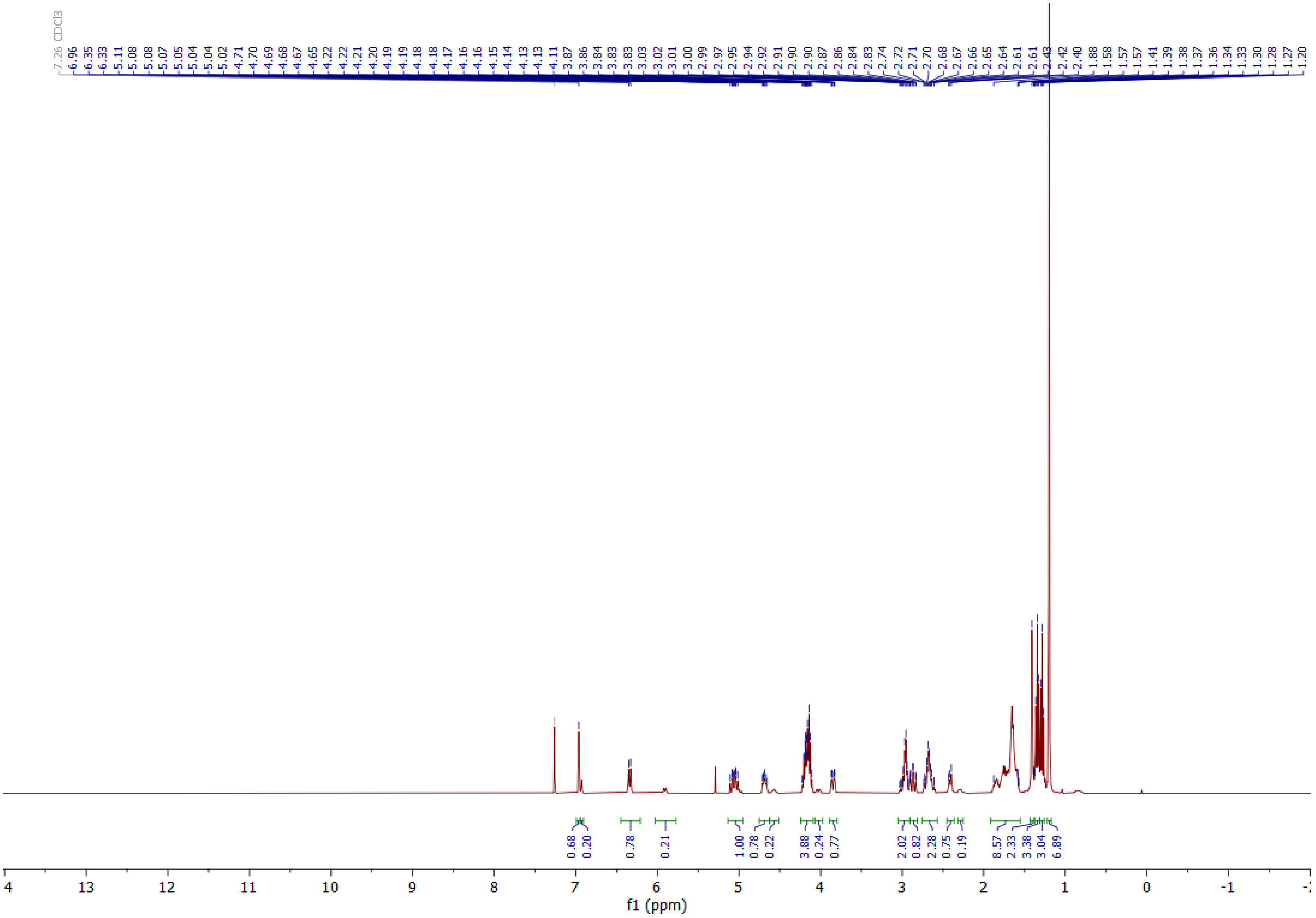

Figure S46. ^{1}H NMR spectrum of **4f** diastereomer 2.

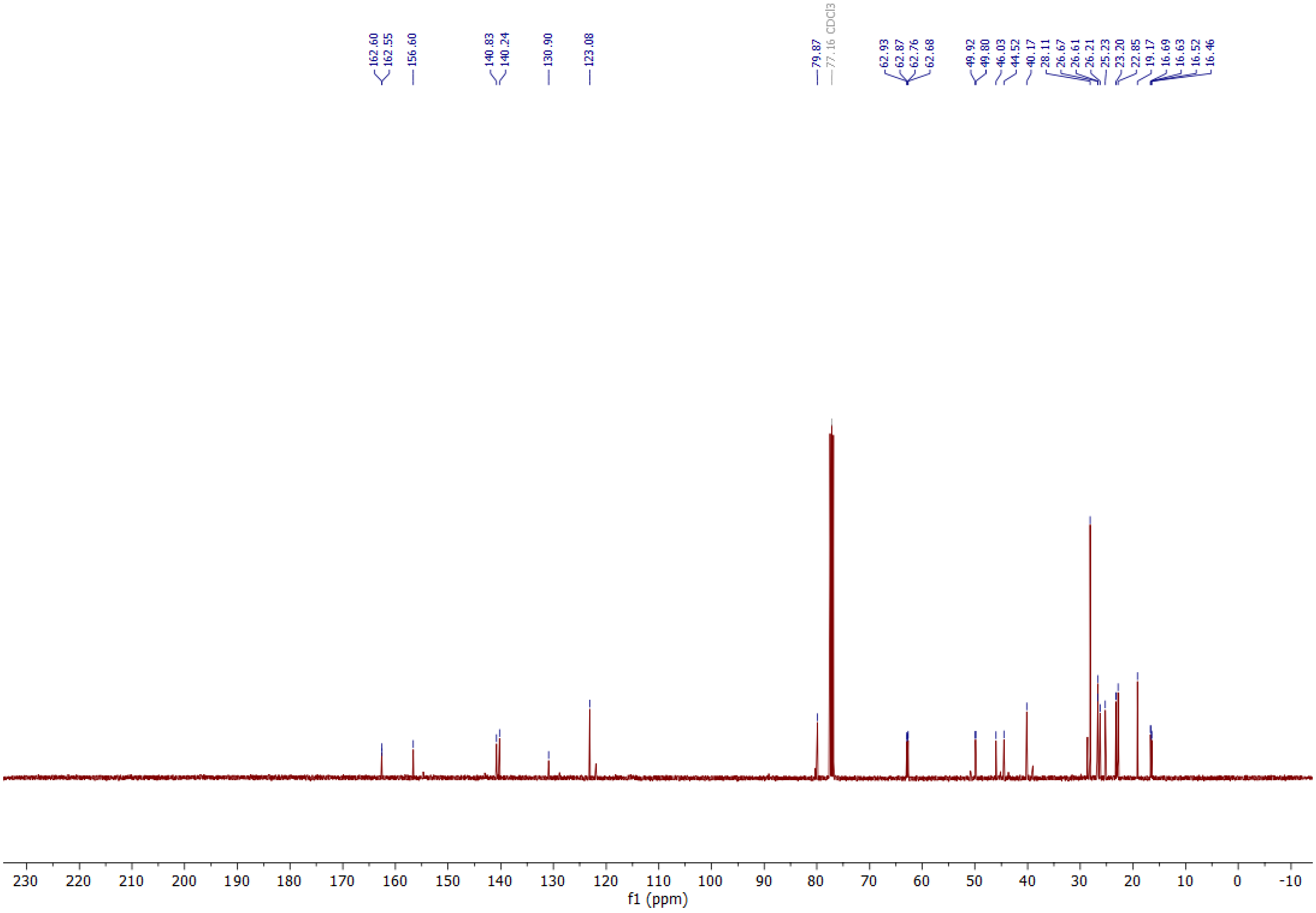

Figure S47. ^{13}C NMR spectrum of **4f** diastereomer 2.

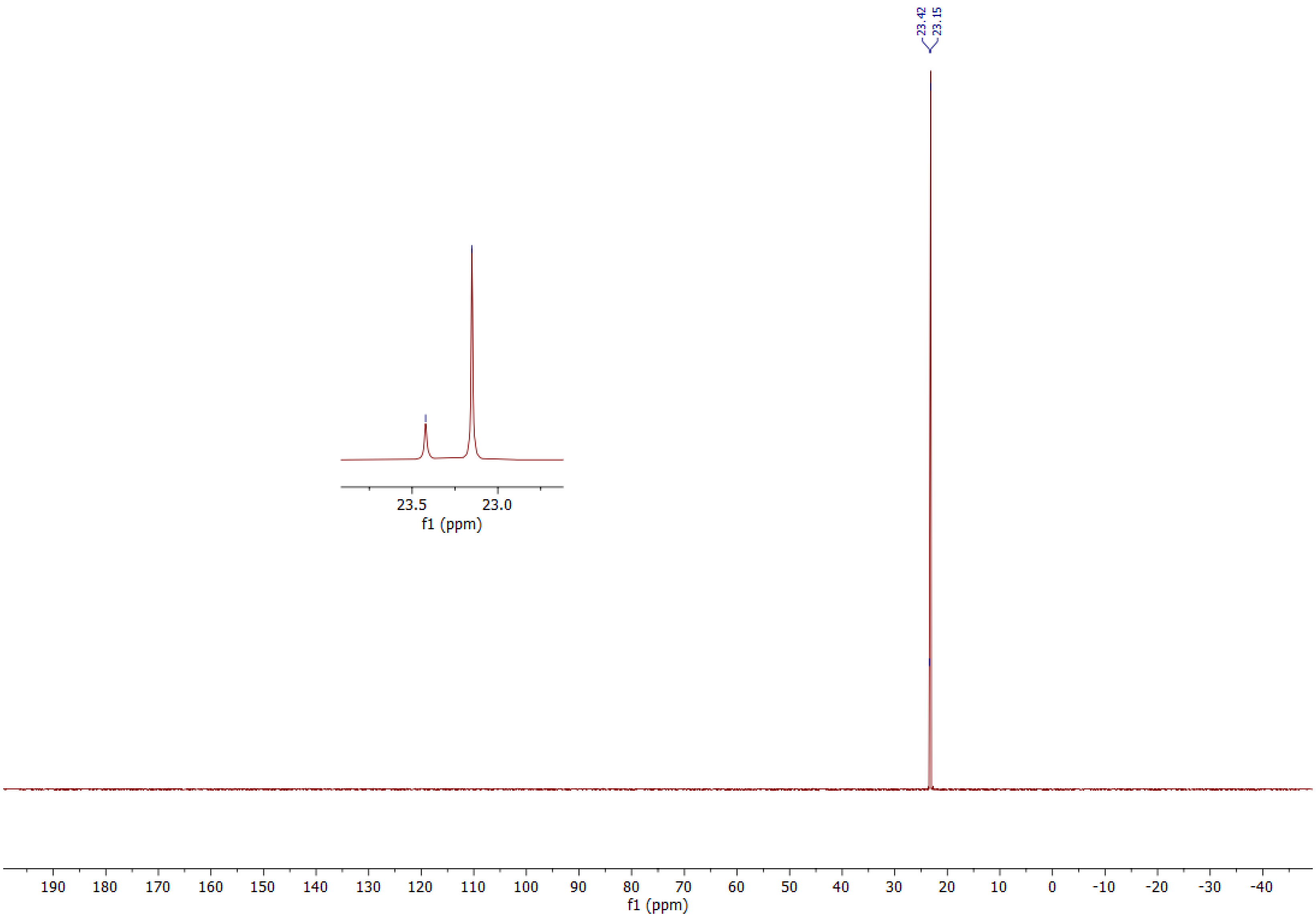

Figure S48. ^{31}P NMR spectrum of **4f** diastereomer 2.

Tert-butyl 2-((benzo[*b*]thiophene-2-carboxamido)(diethoxyphosphoryl)methyl)piperidine-1-carboxylate (**4g**)

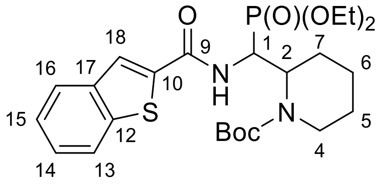

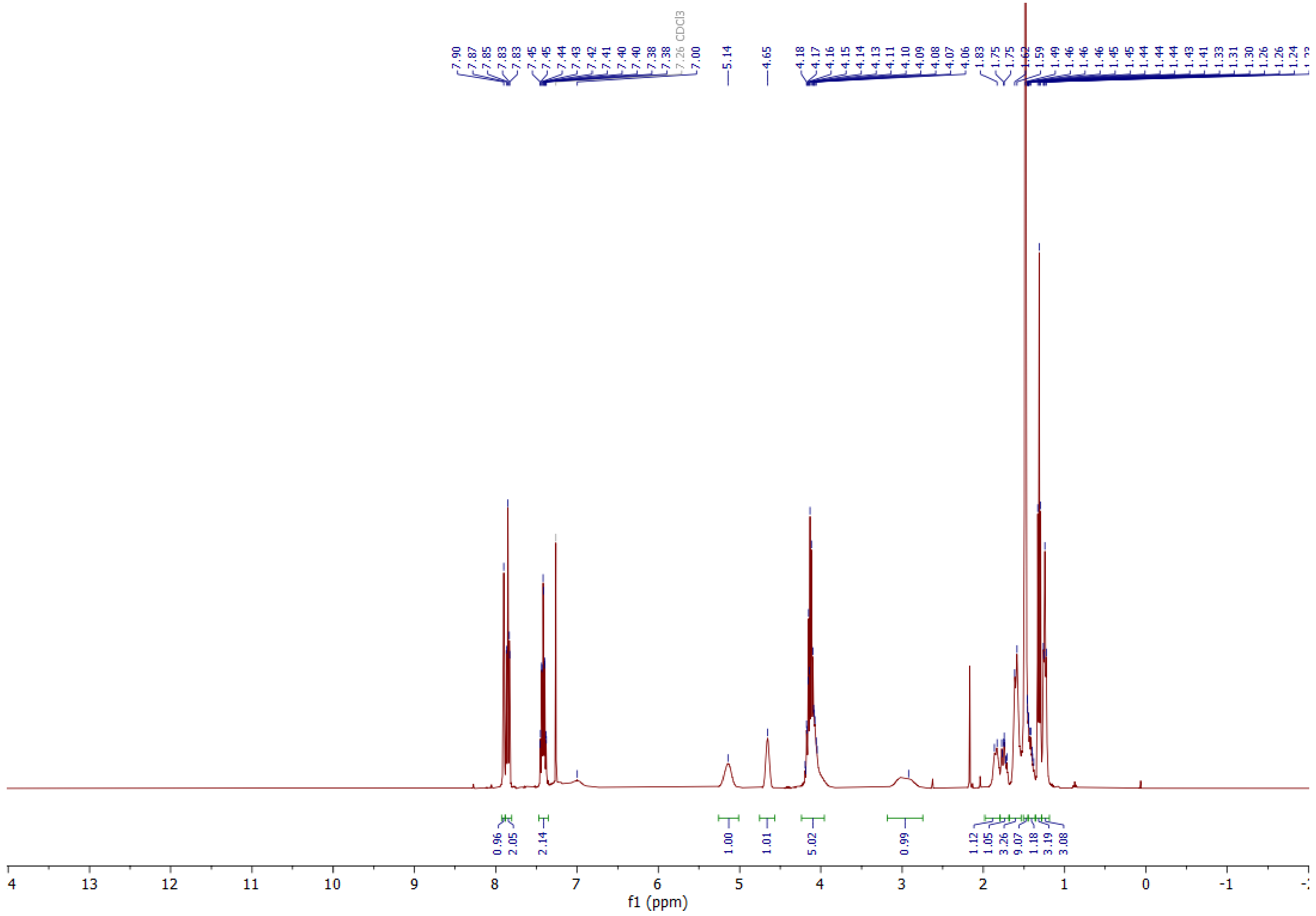

Figure S49. ^{1}H NMR spectrum of **4g** diastereomer 1.

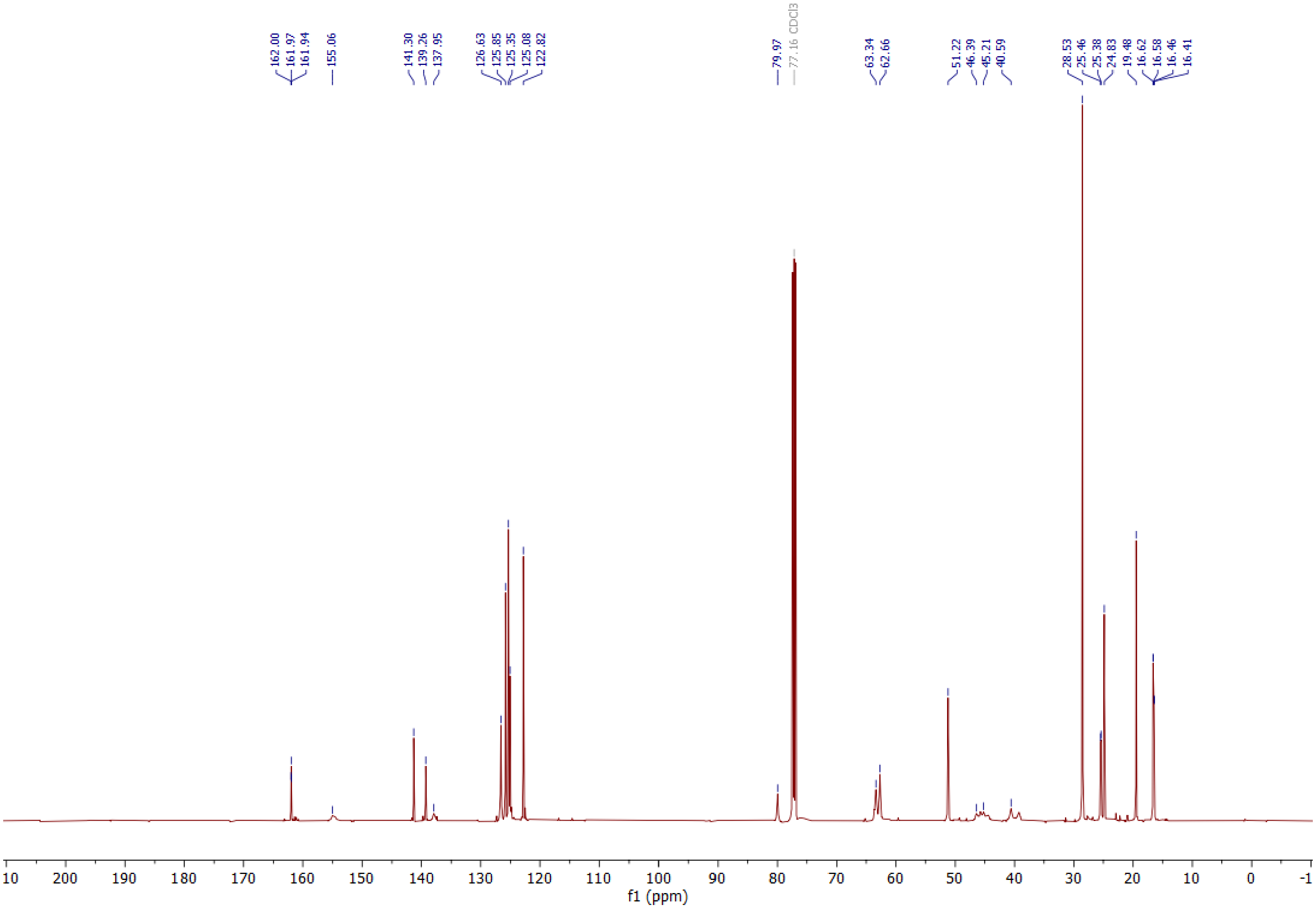

Figure S50. ^{13}C NMR spectrum of **4g** diastereomer 1.

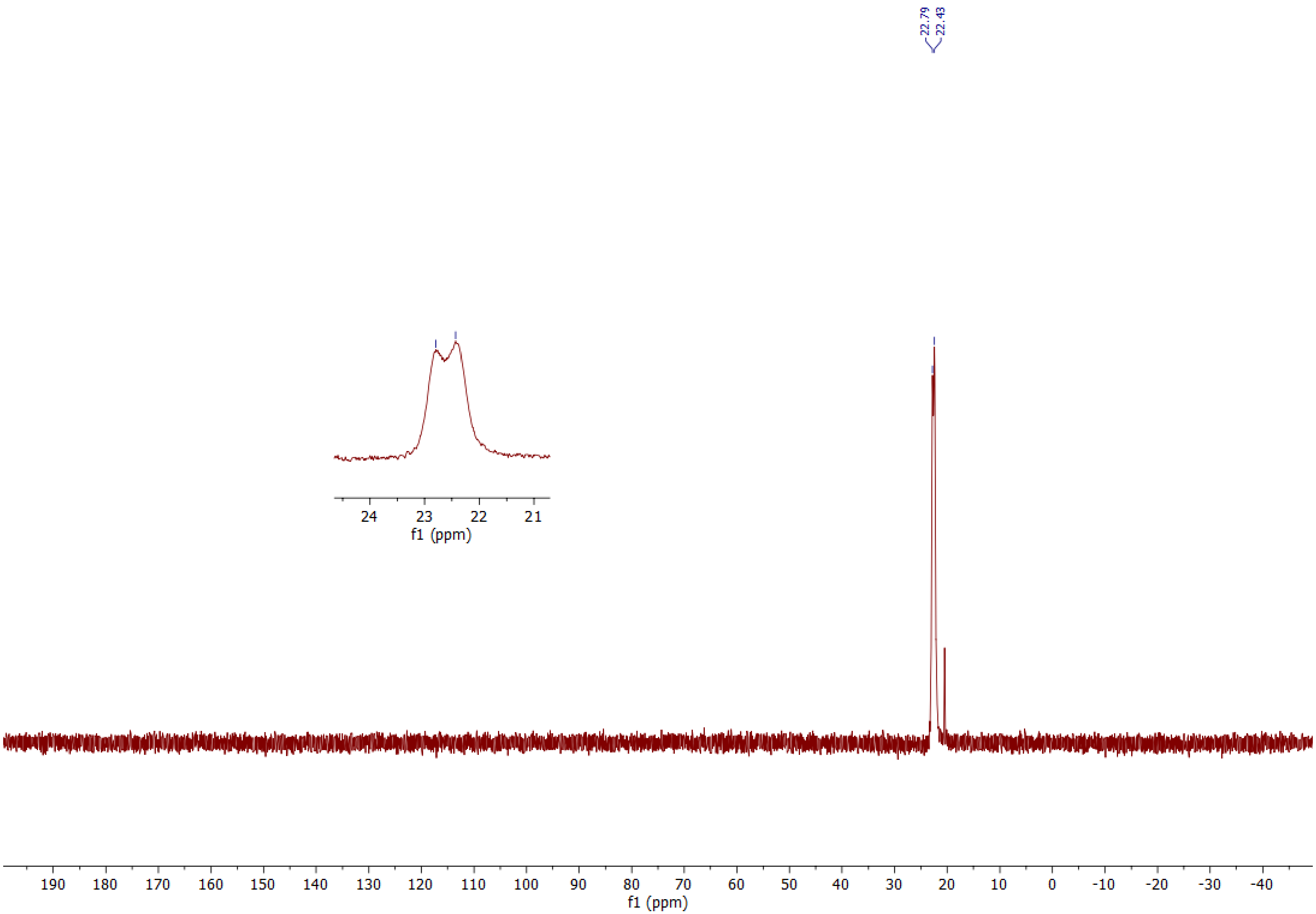

Figure S51. ^{31}P NMR spectrum of **4g** diastereomer 1.

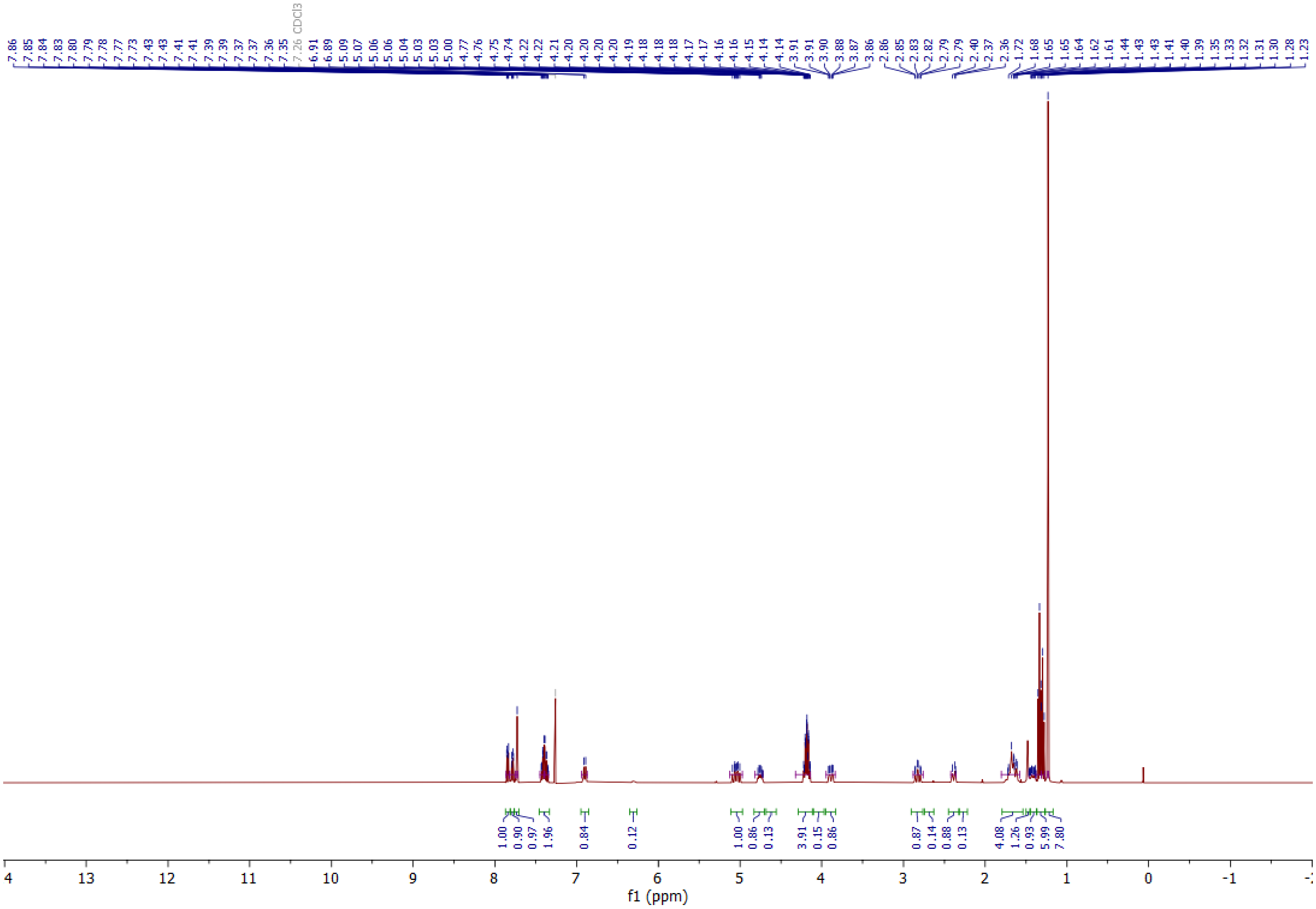

Figure S52. ^{1}H NMR spectrum of **4g** diastereomer 2.

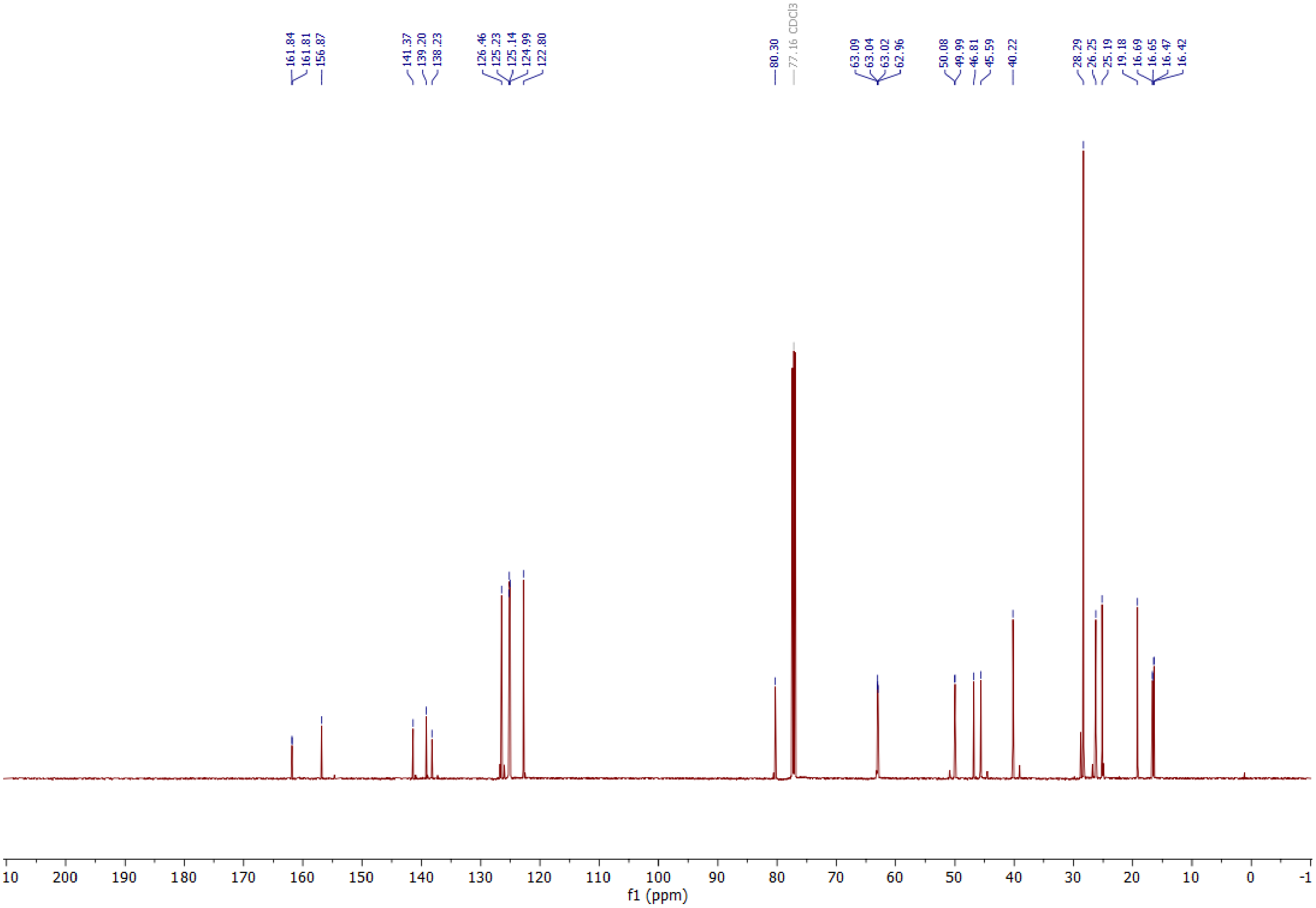

Figure S53. ^{13}C NMR spectrum of **4g** diastereomer 2.

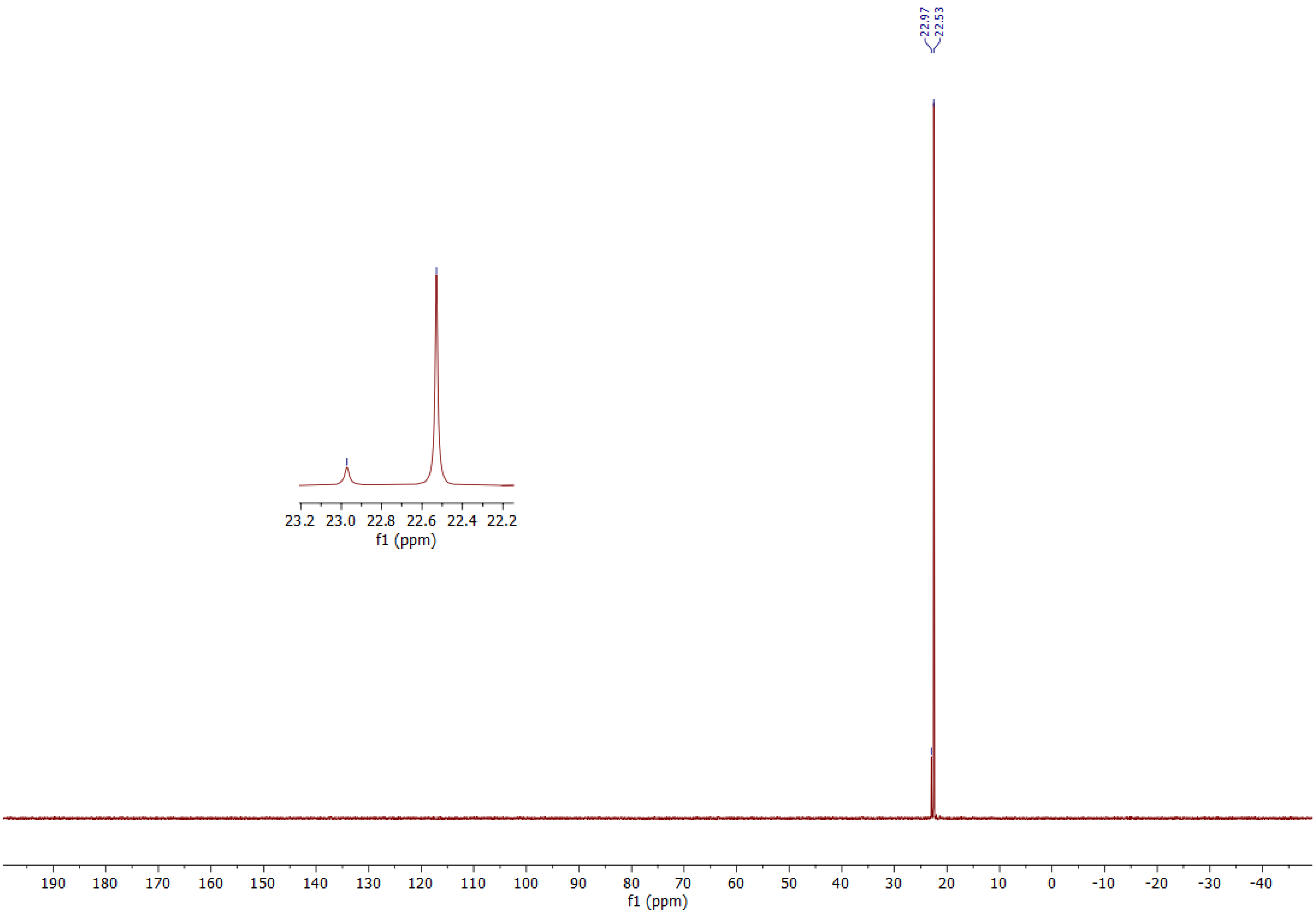

Figure S54. ^{31}P NMR spectrum of **4g** diastereomer 2.

Tert-butyl 2-((benzo[*b*]thiophene-3-carboxamido)(diethoxyphosphoryl)methyl)piperidine-1-carboxylate (**4h**)

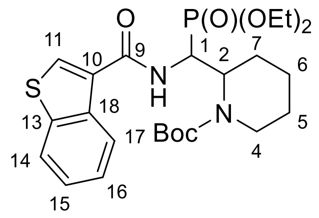

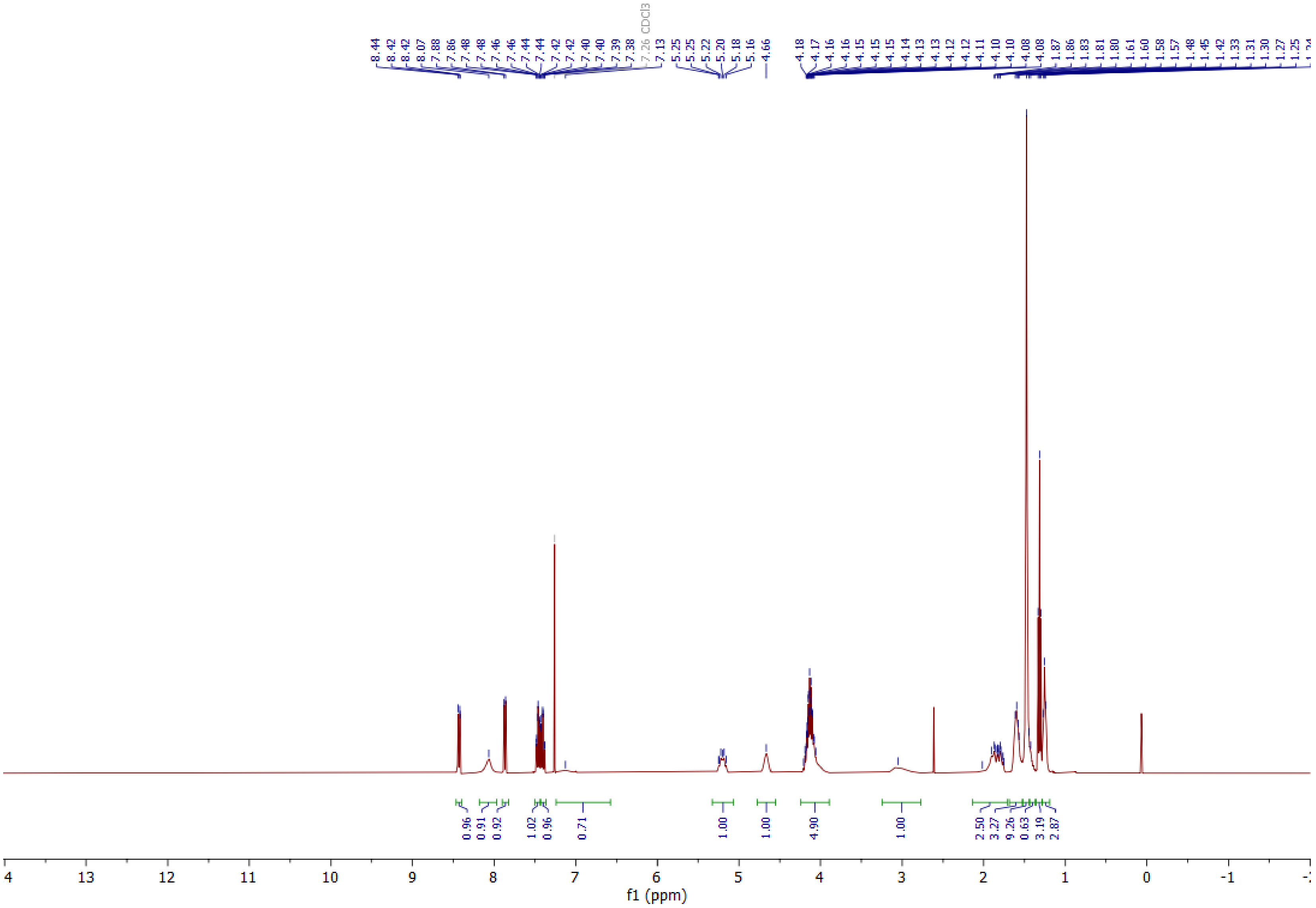

Figure S55. ^{1}H NMR spectrum of **4h** diastereomer 1.

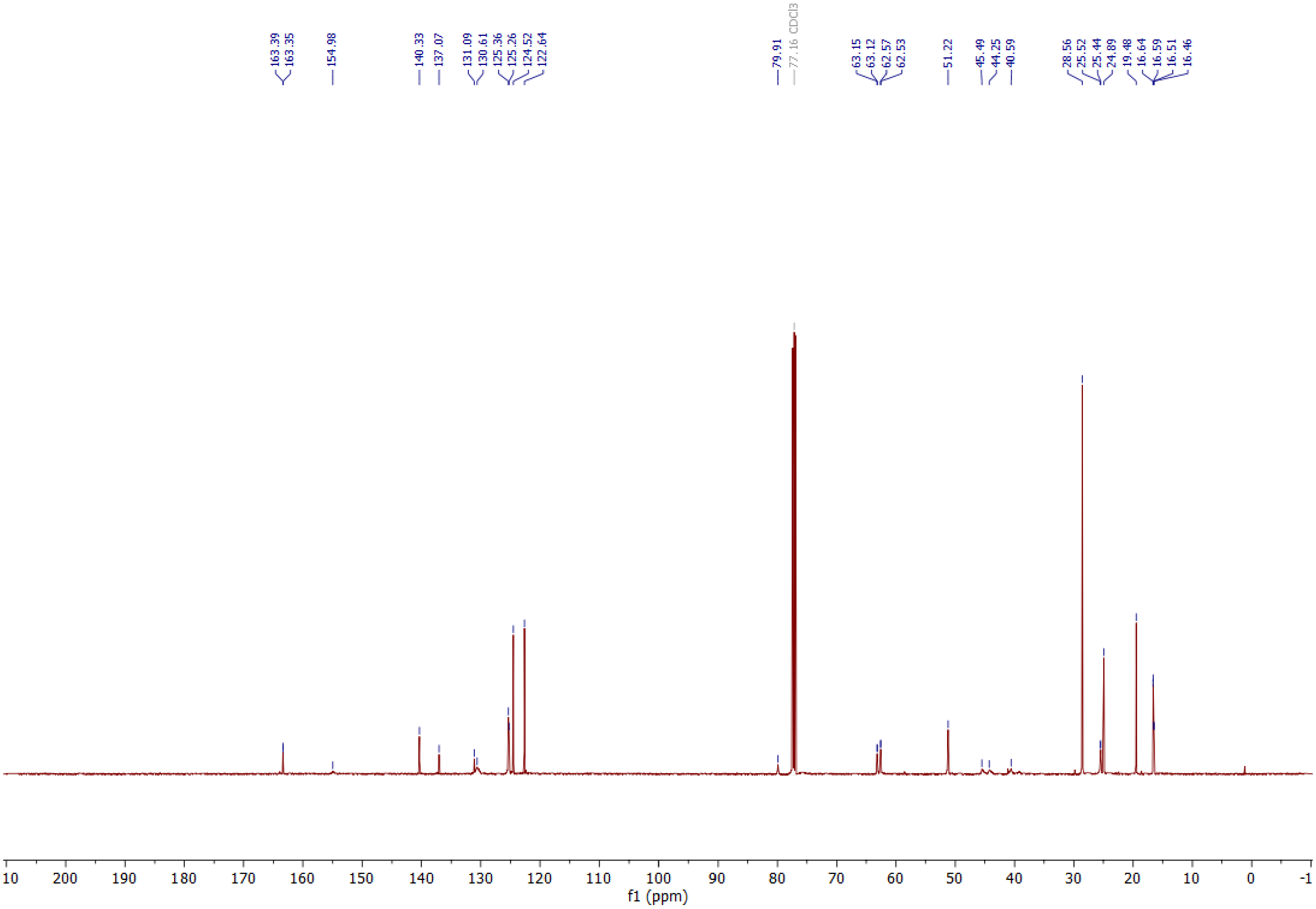

Figure S56. ^{13}C NMR spectrum of **4h** diastereomer 1.

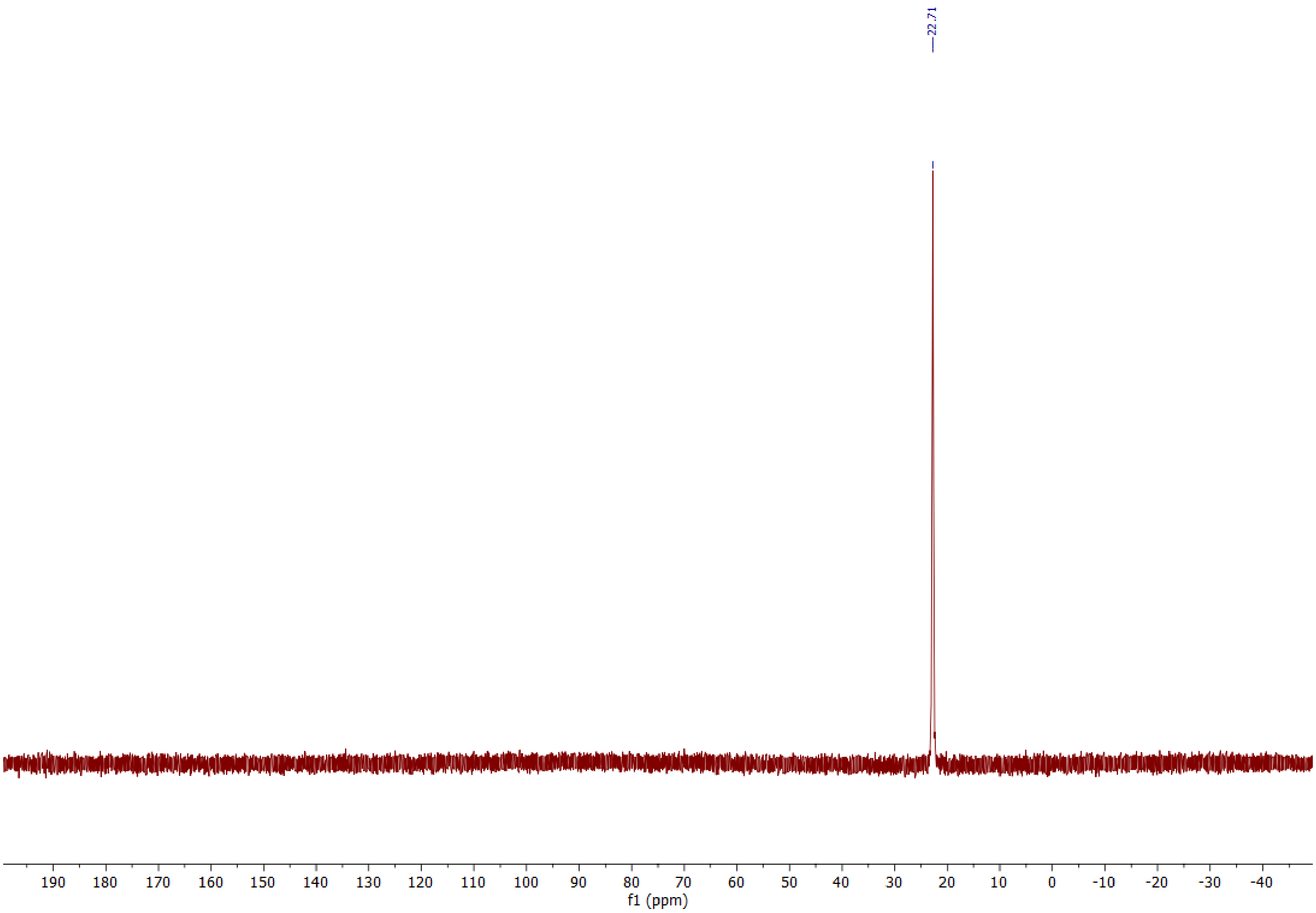

Figure S57. ^{31}P NMR spectrum of **4h** diastereomer 1.

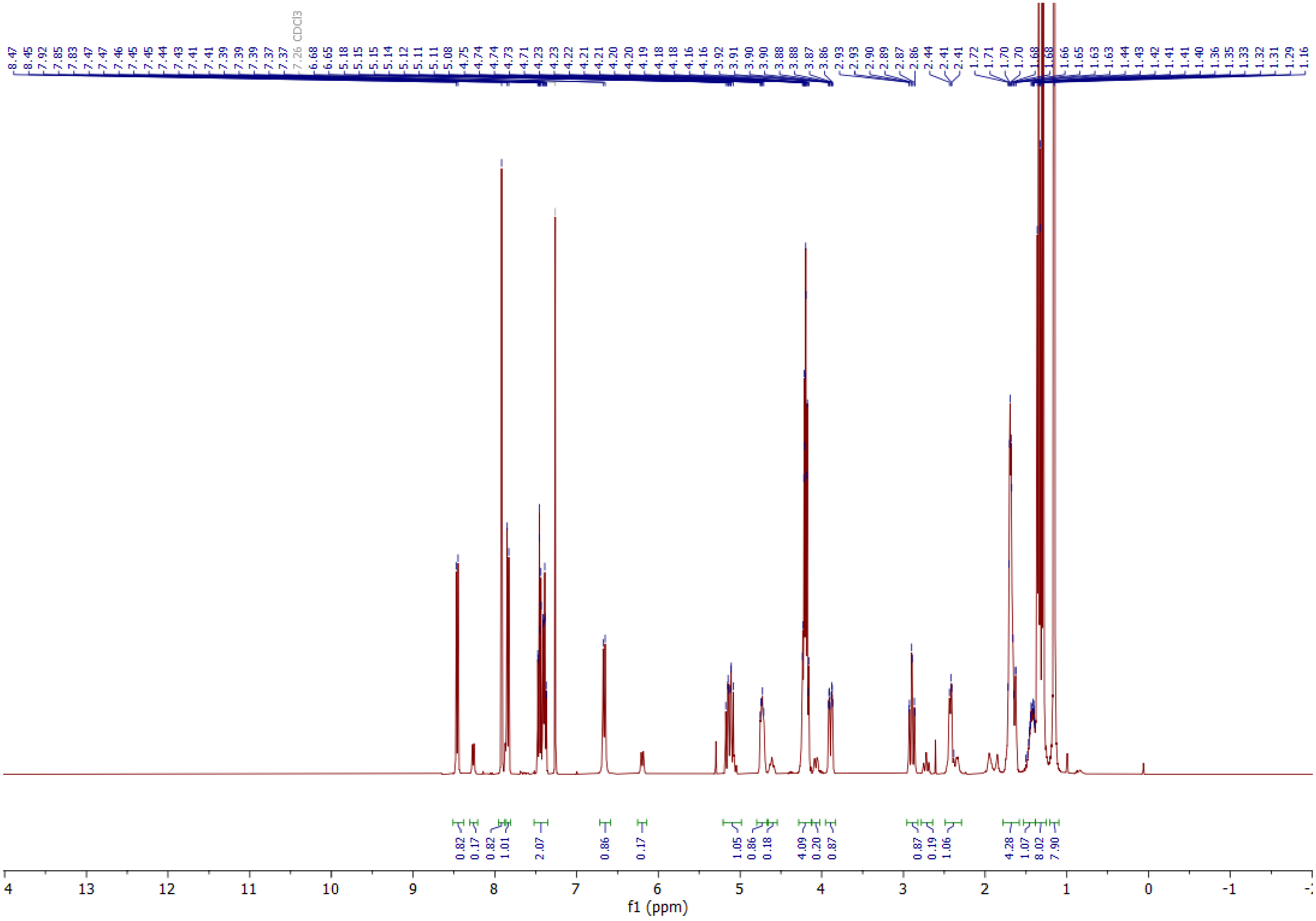

Figure S58. ^{1}H NMR spectrum of **4h** diastereomer 2.

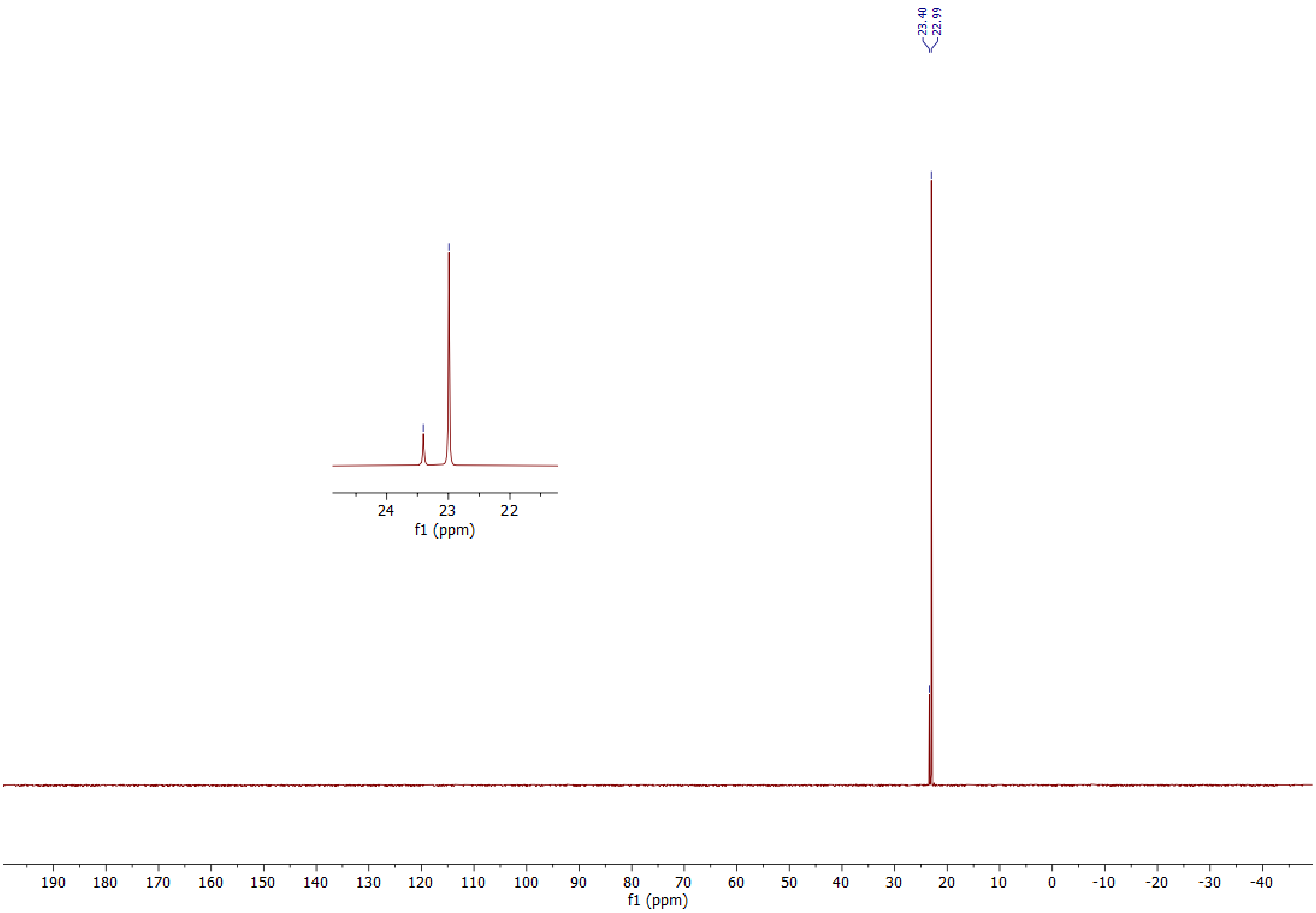

Figure S59. ^{31}P NMR spectrum of **4h** diastereomer 2.

Tert-butyl 2-((diethoxyphosphoryl)(2-(thiophen-2-yl)acetamido)methyl)piperidine-1-carboxylate (**4i**)

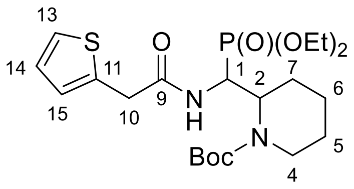

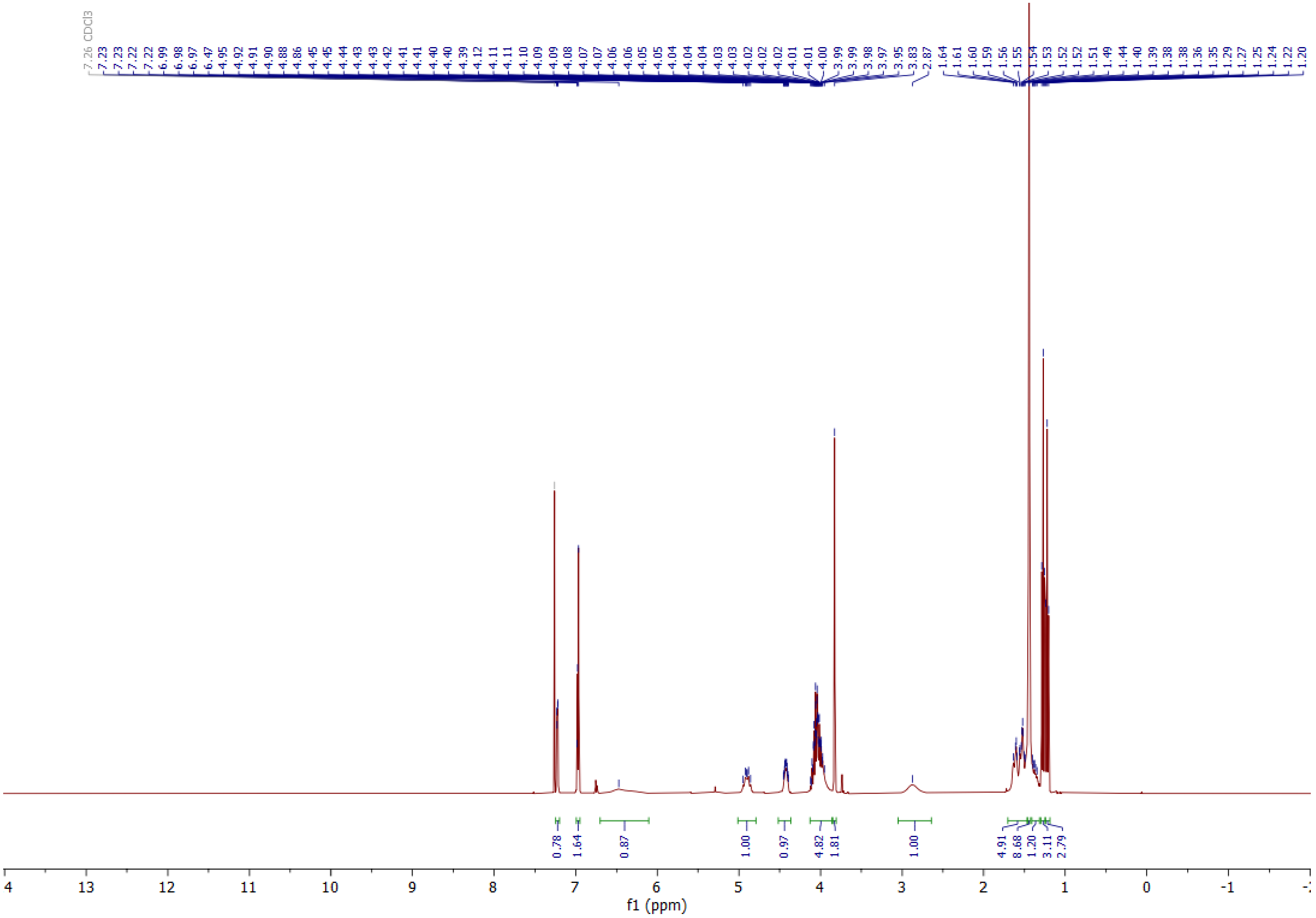

Figure S60. ^{1}H NMR spectrum of **4i** diastereomer 1.

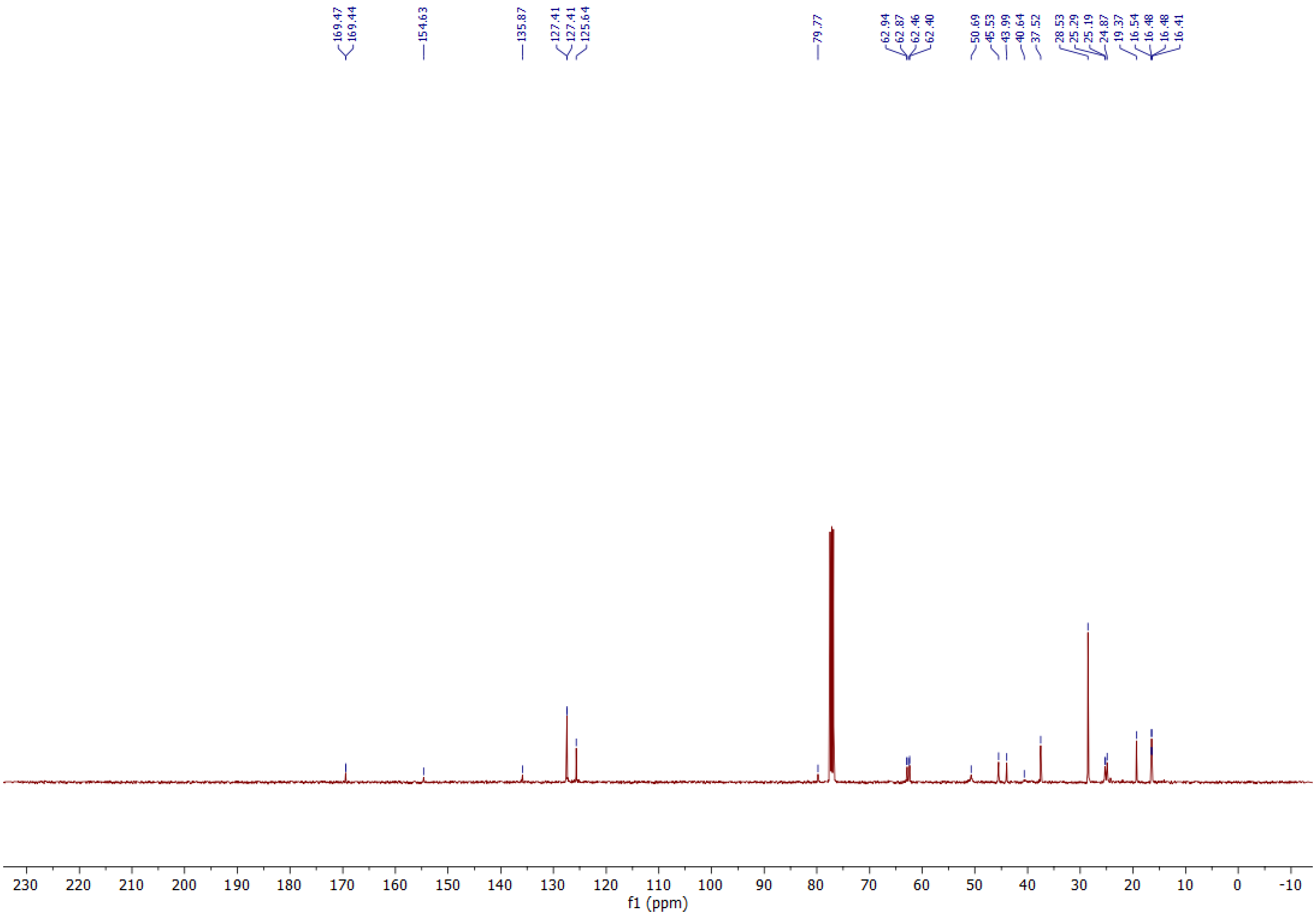

Figure S61. ^{13}C NMR spectrum of **4i** diastereomer 1.

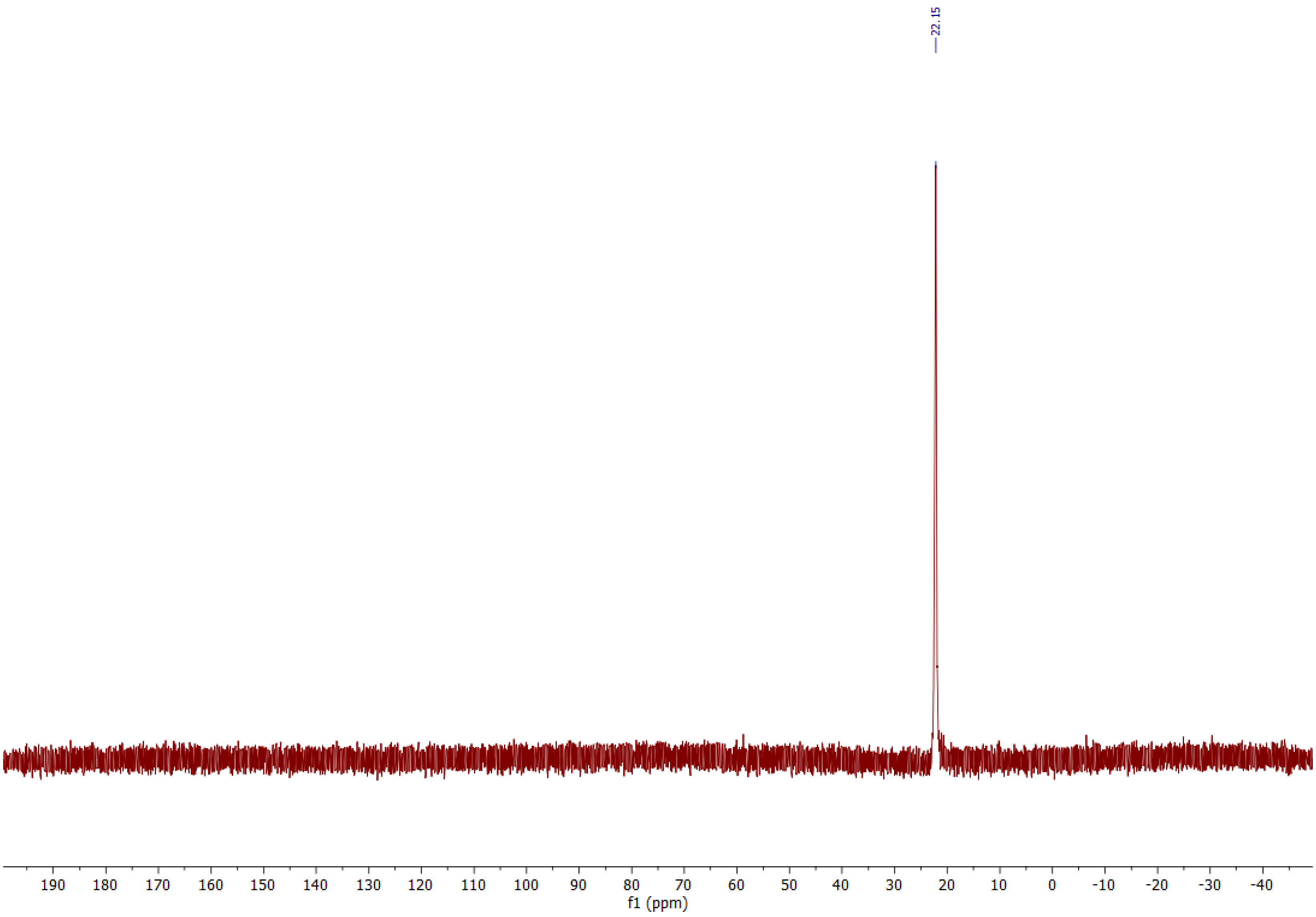

Figure S62. ^{31}P NMR spectrum of **4i** diastereomer 1.

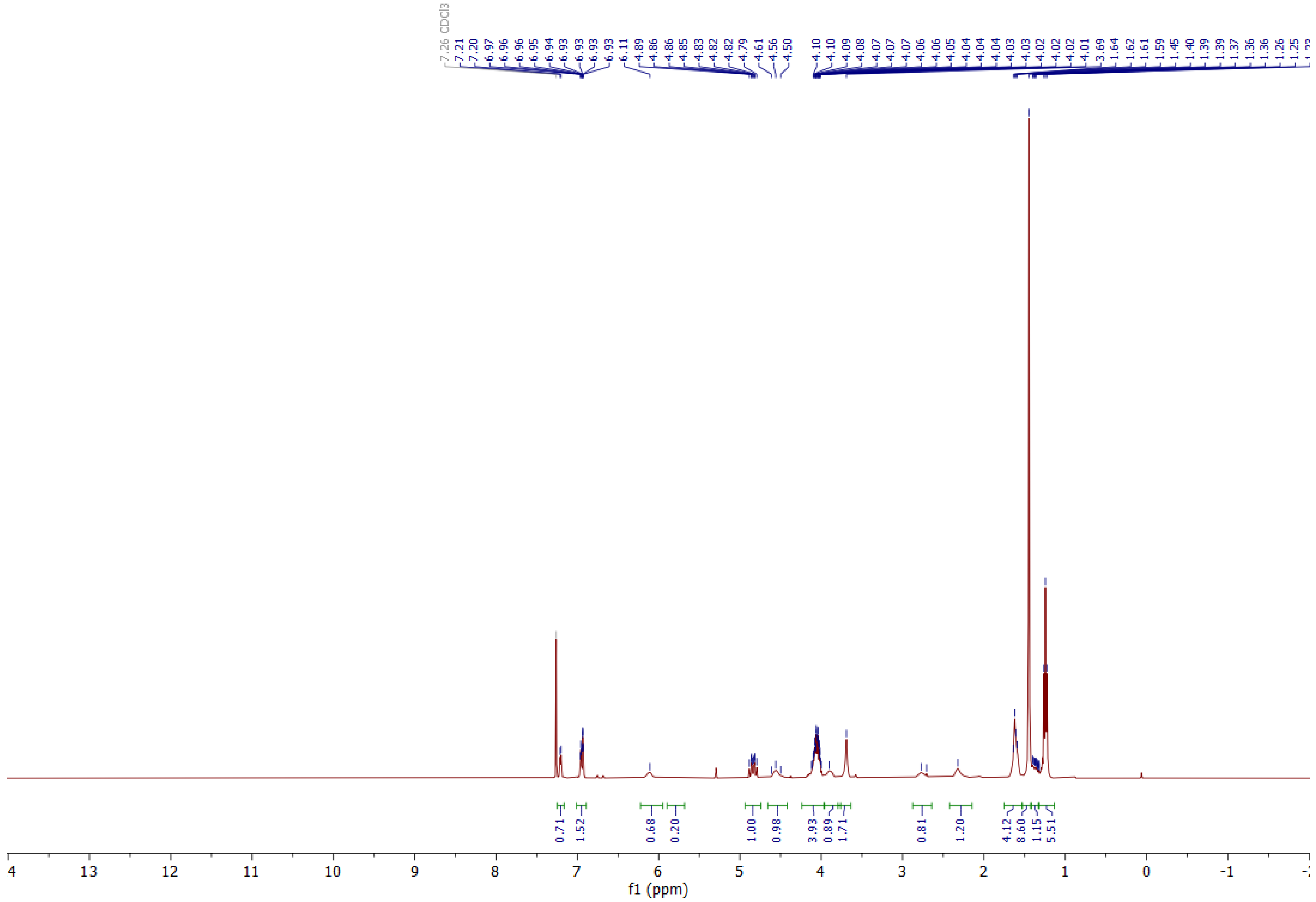

Figure S63. ^{1}H NMR spectrum of **4i** diastereomer 2.

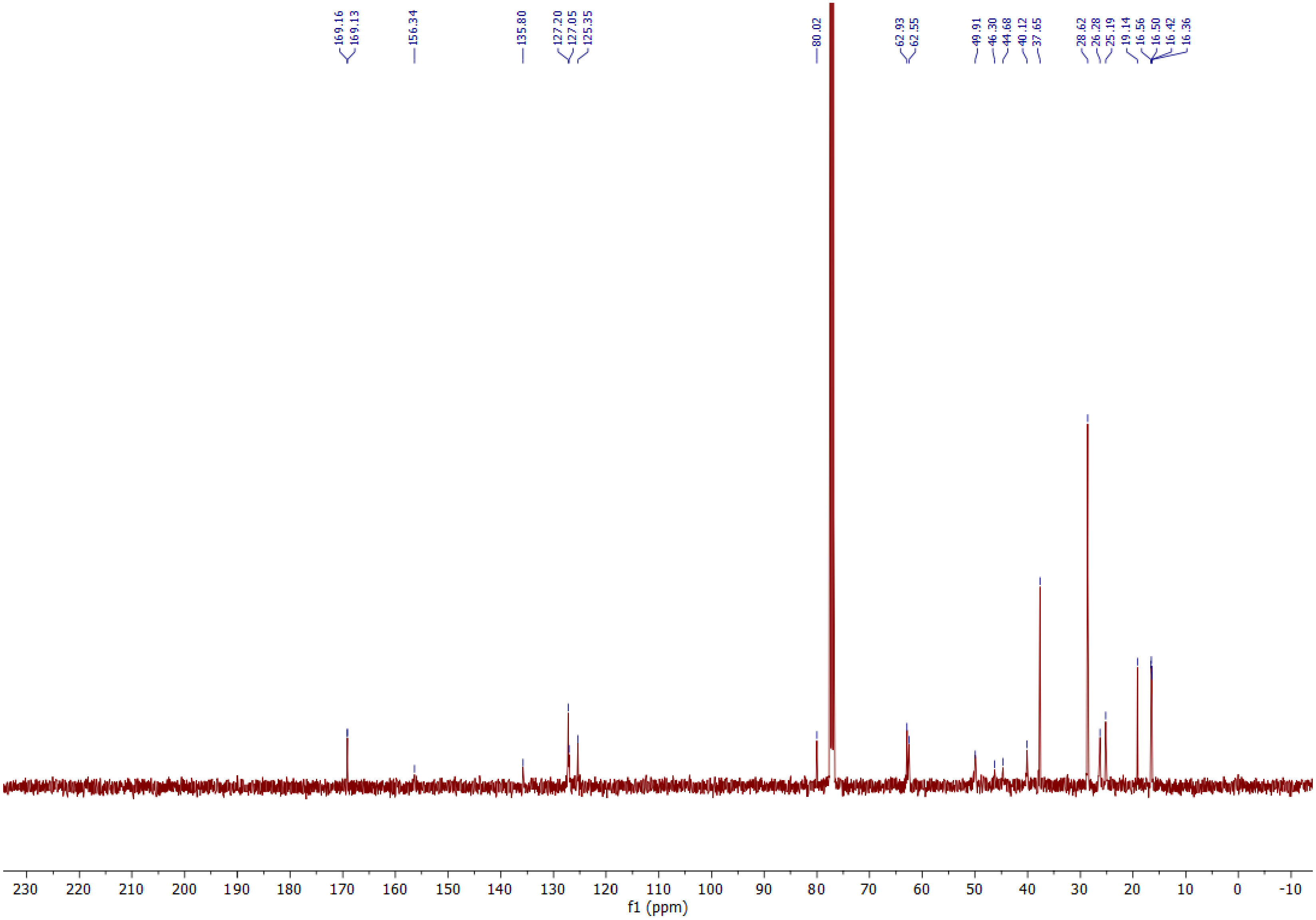

Figure S64. ^{13}C NMR spectrum of **4i** diastereomer 2.

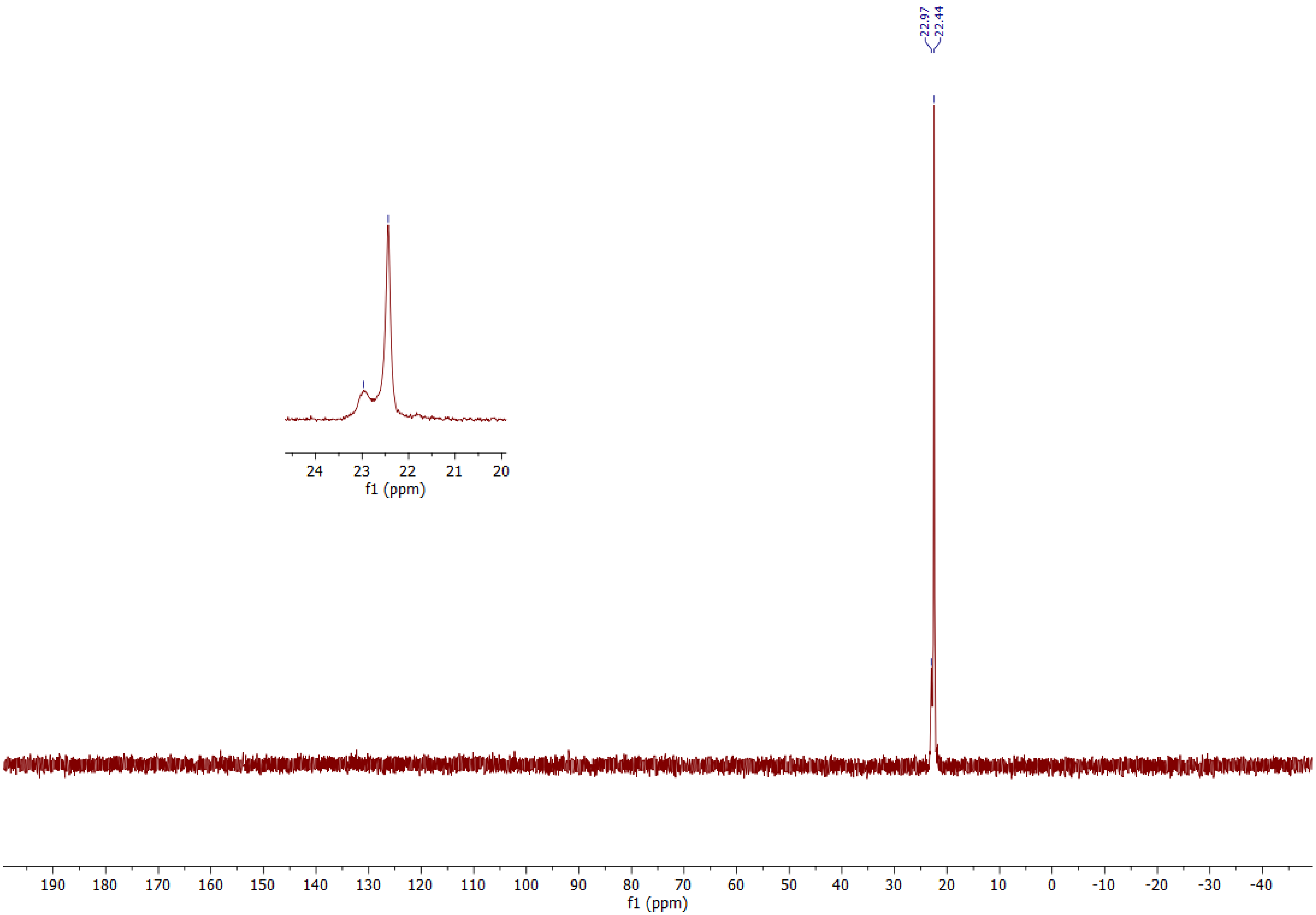

Figure S65. ^{31}P NMR spectrum of **4i** diastereomer 2.

Tert-butyl 2-((diethoxyphosphoryl)(2-(thiophen-3-yl)acetamido)methyl)piperidine-1-carboxylate (**4j**)

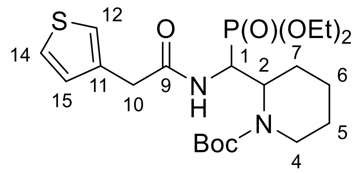

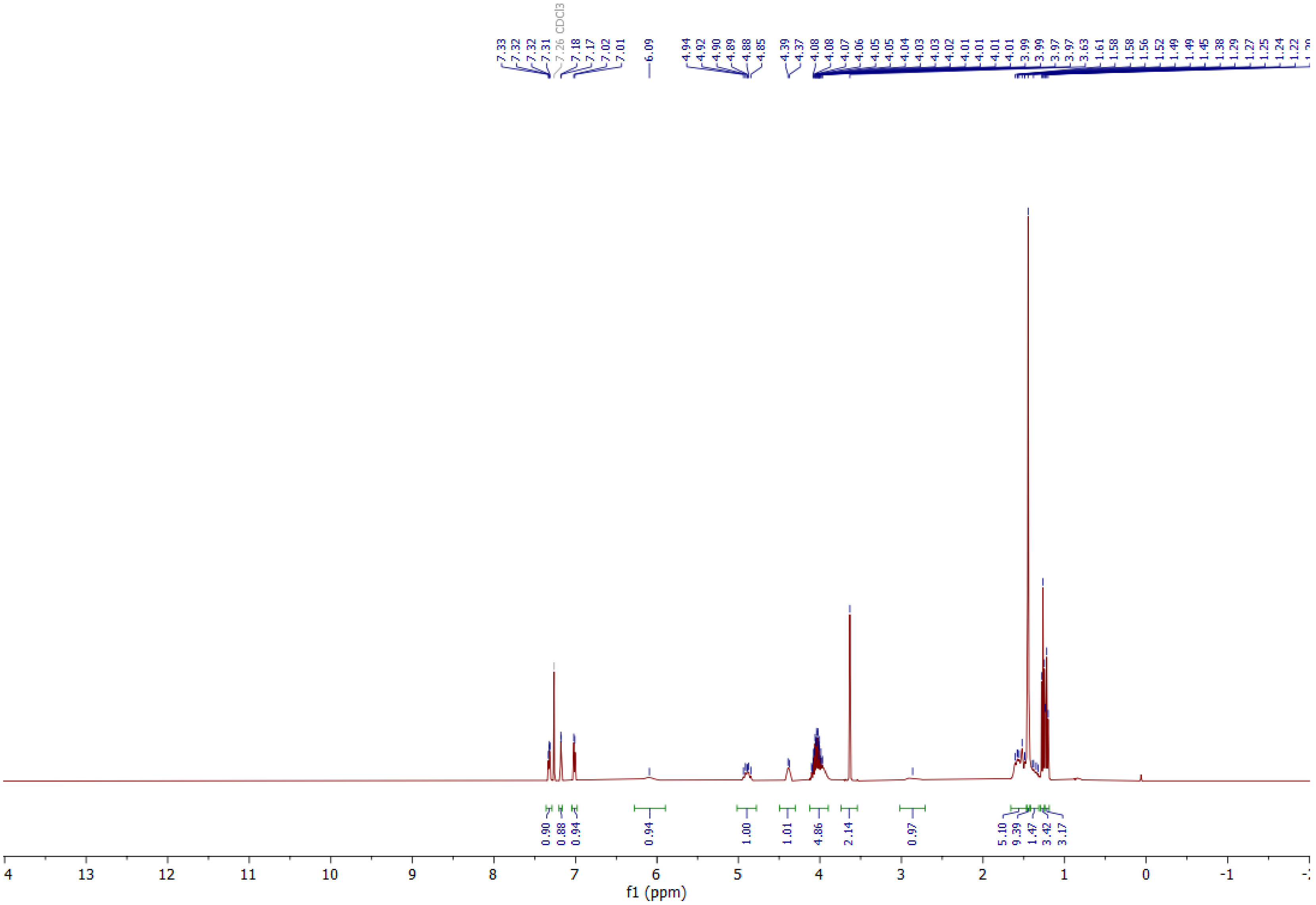

Figure S66. ^{1}H NMR spectrum of **4j** diastereomer 1.

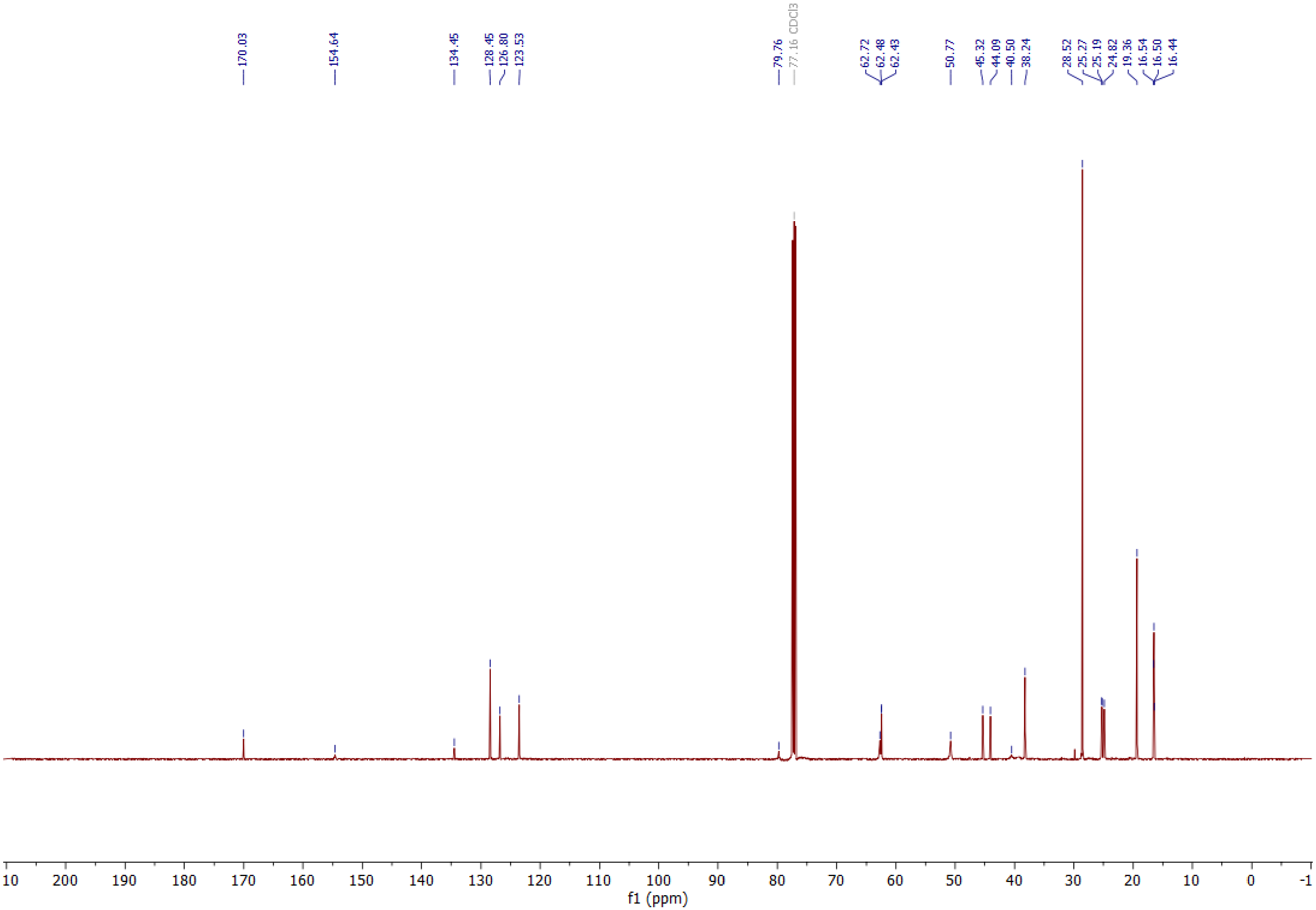

Figure S67. ^{13}C NMR spectrum of **4j** diastereomer 1.

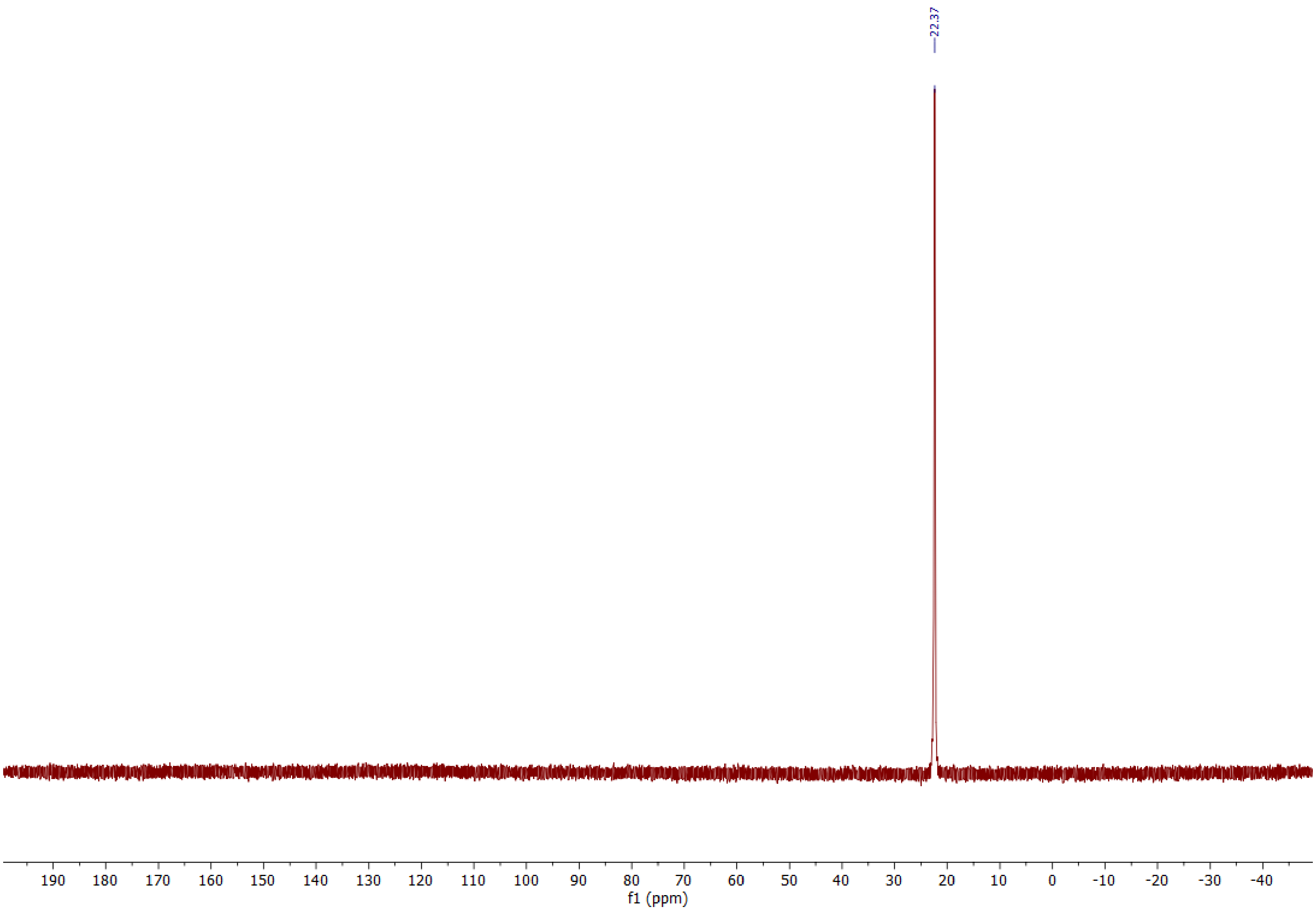

Figure S68. ^{31}P NMR spectrum of **4j** diastereomer 1.

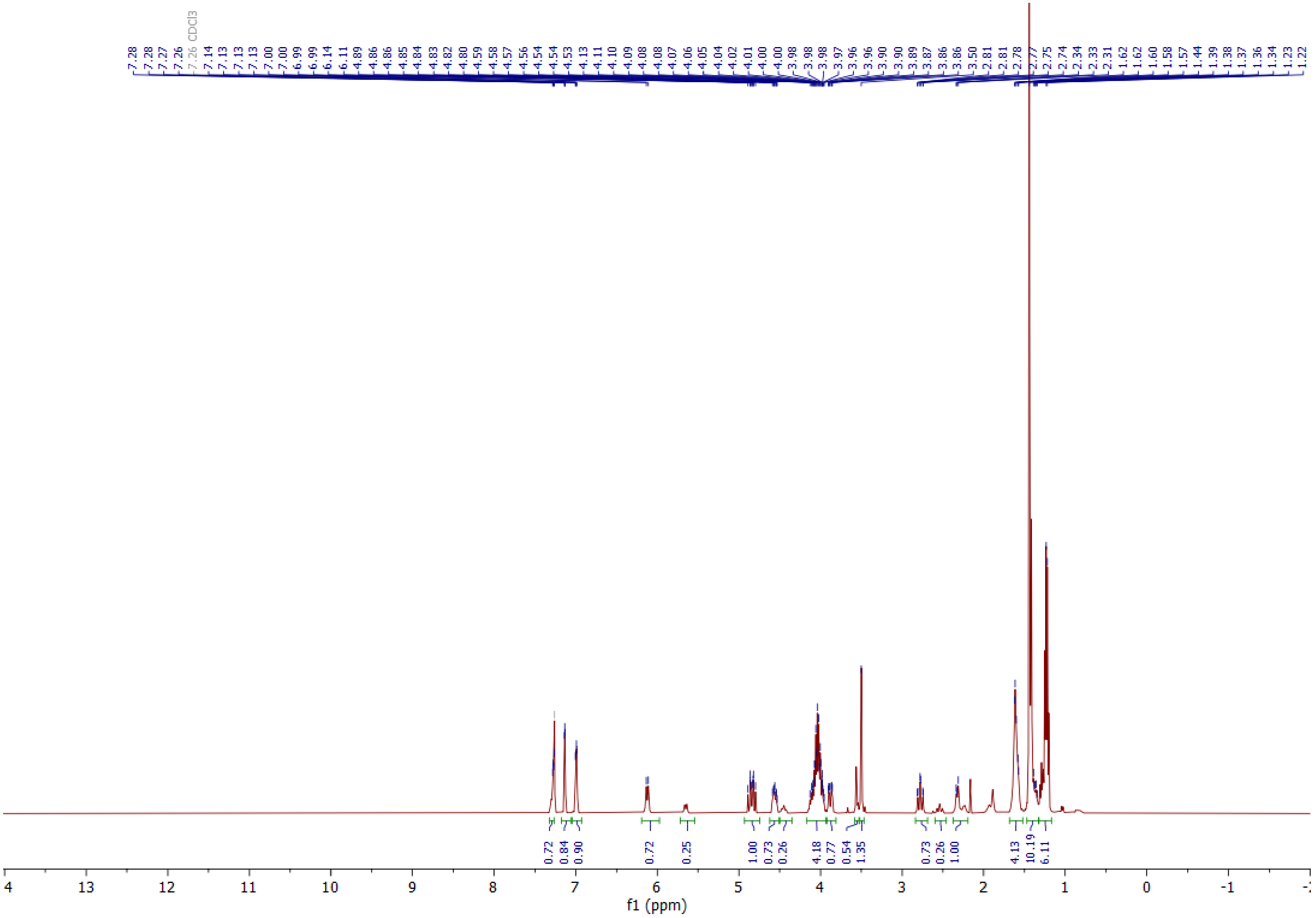

Figure S69. ^{1}H NMR spectrum of **4j** diastereomer 2.

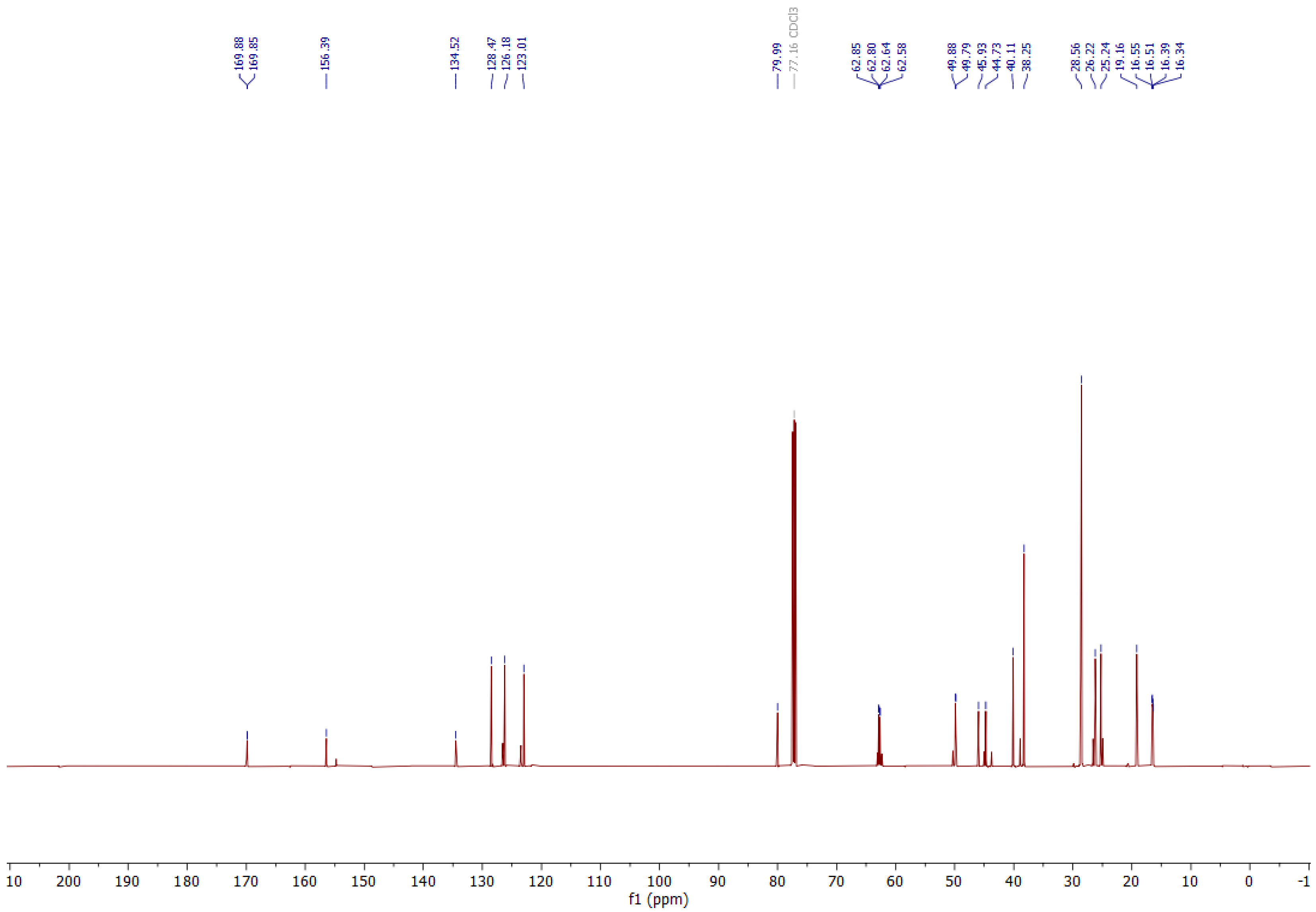

Figure S70. ^{13}C NMR spectrum of **4j** diastereomer 2.

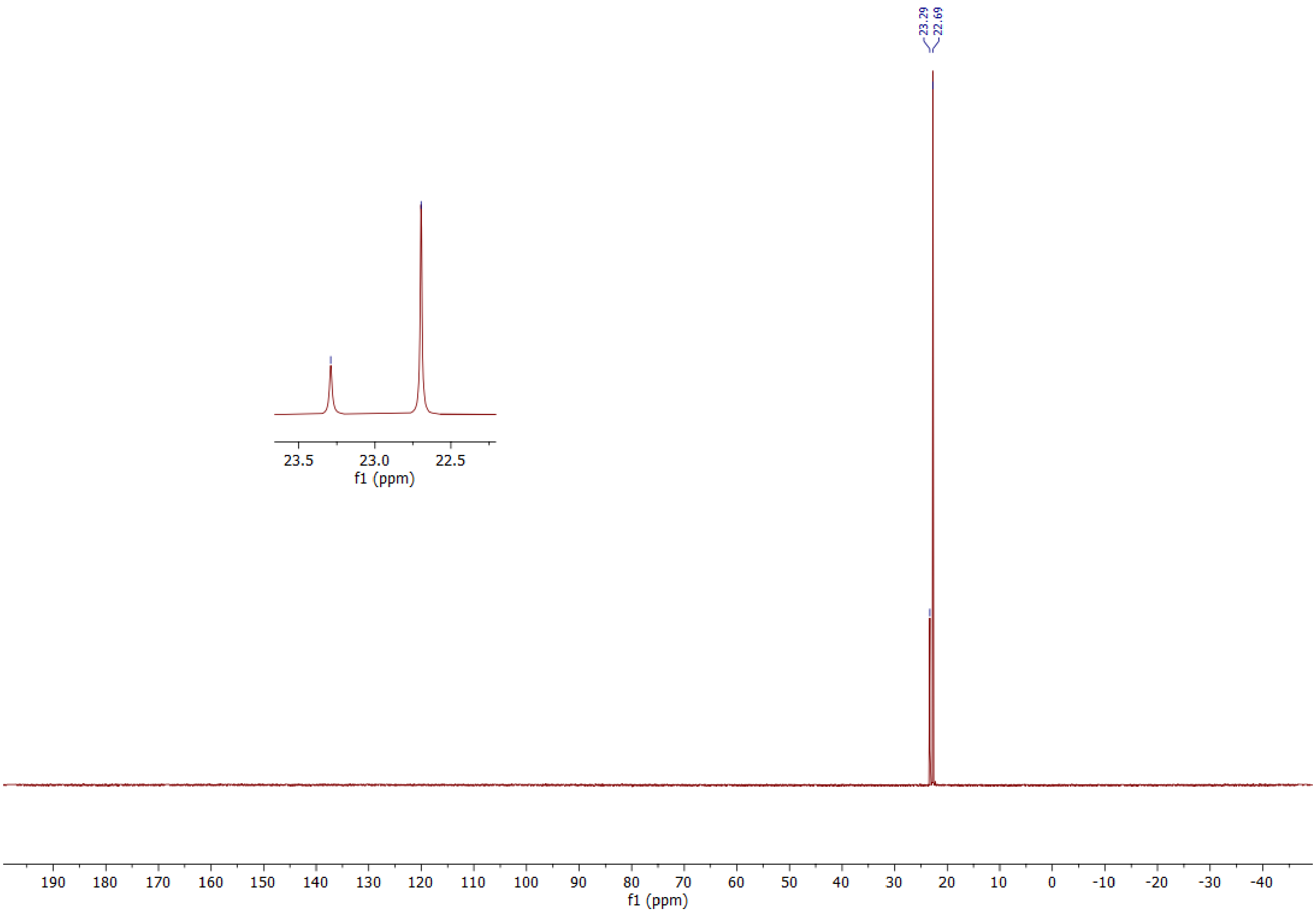

Figure S71. ^{31}P NMR spectrum of **4j** diastereomer 2.

Tert-butyl 2-((2-(benzo[*b*]thiophen-2-yl)acetamido)(diethoxyphosphoryl)methyl)piperidine-1-carboxylate (**4k**)

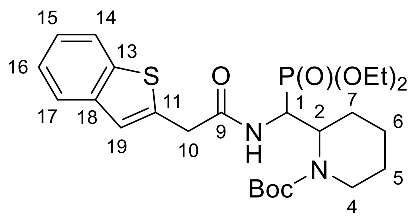

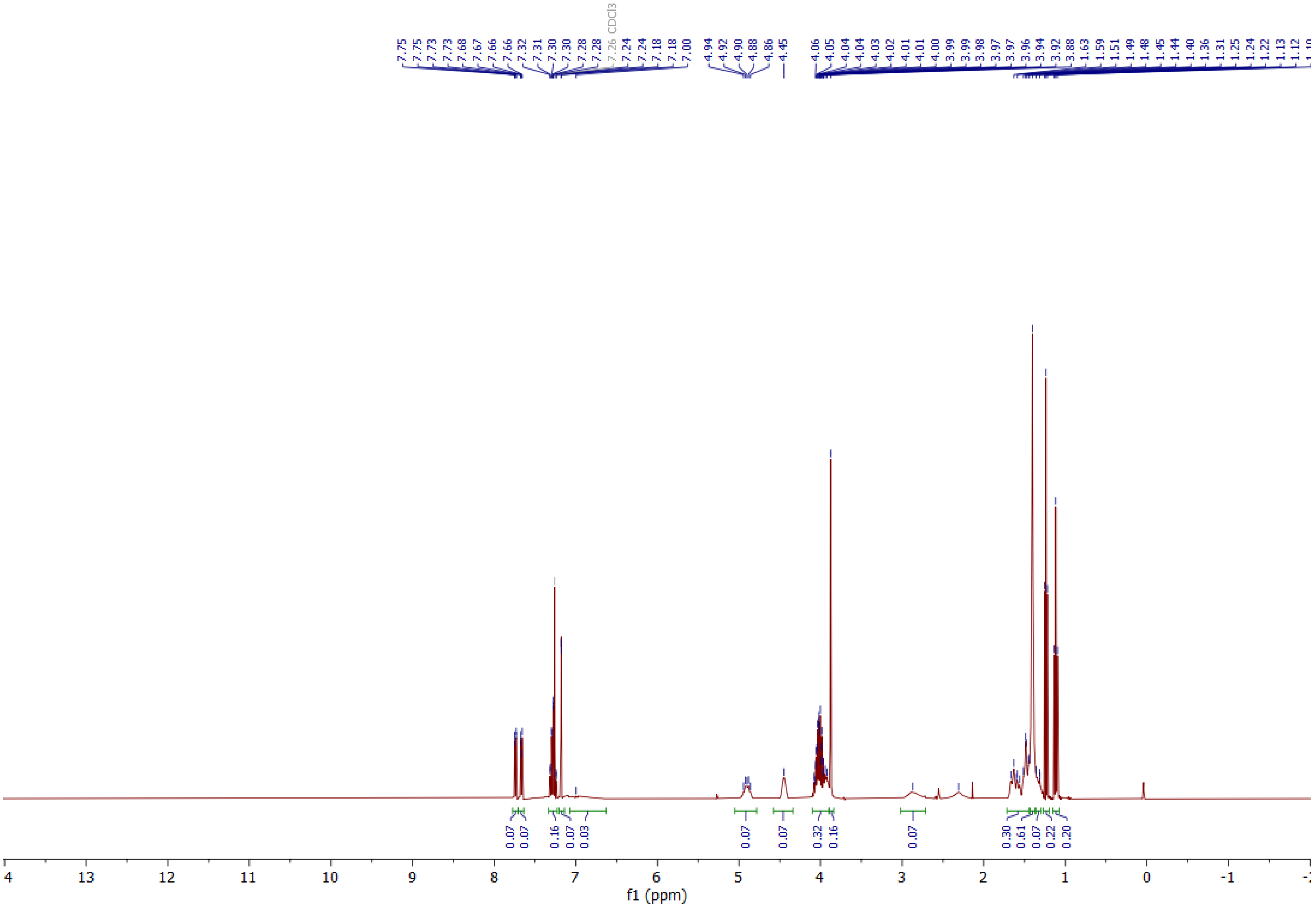

Figure S72. ^{1}H NMR spectrum of **4k** diastereomer 1.

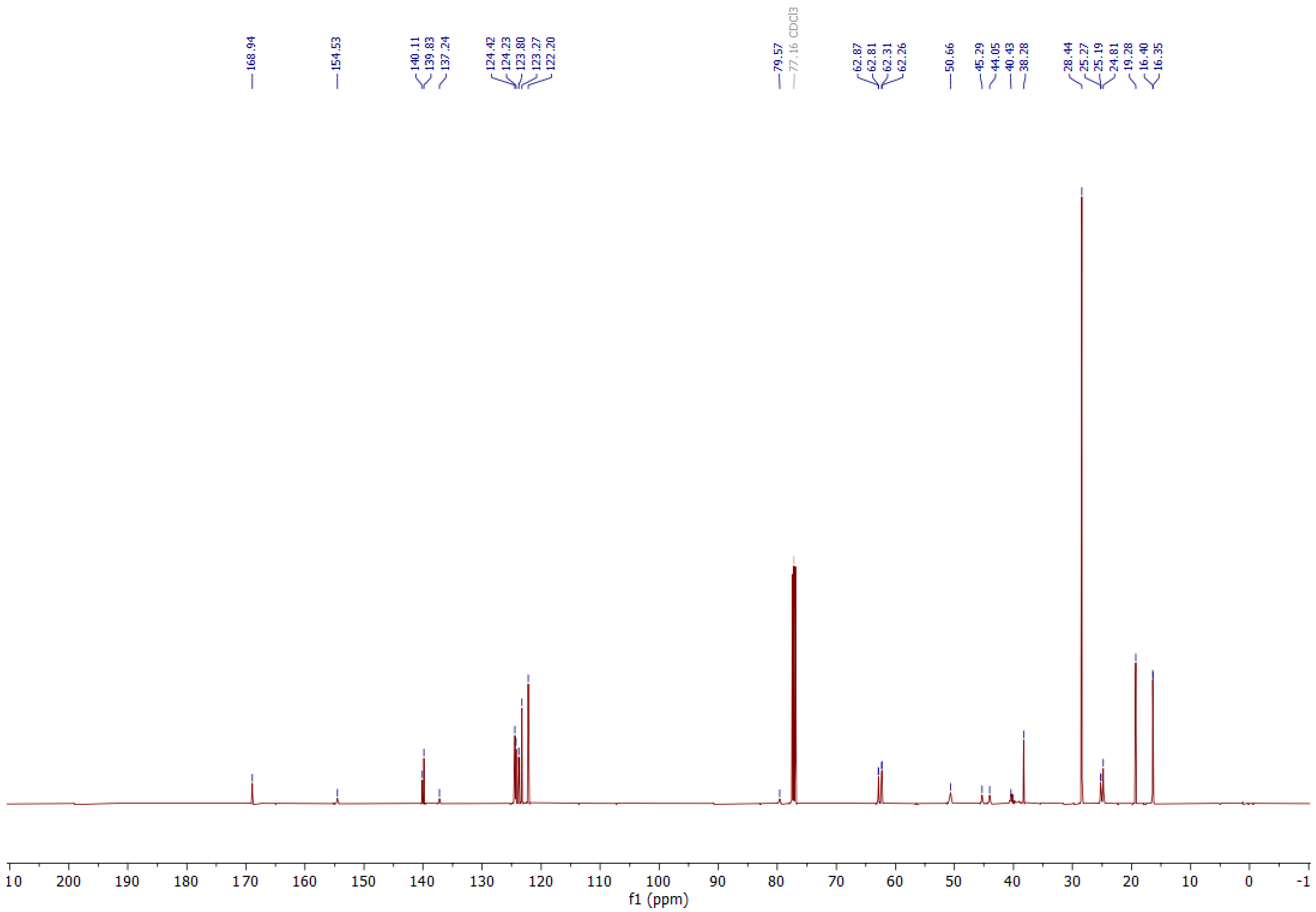

Figure S73. ^{13}C NMR spectrum of **4k** diastereomer 1.

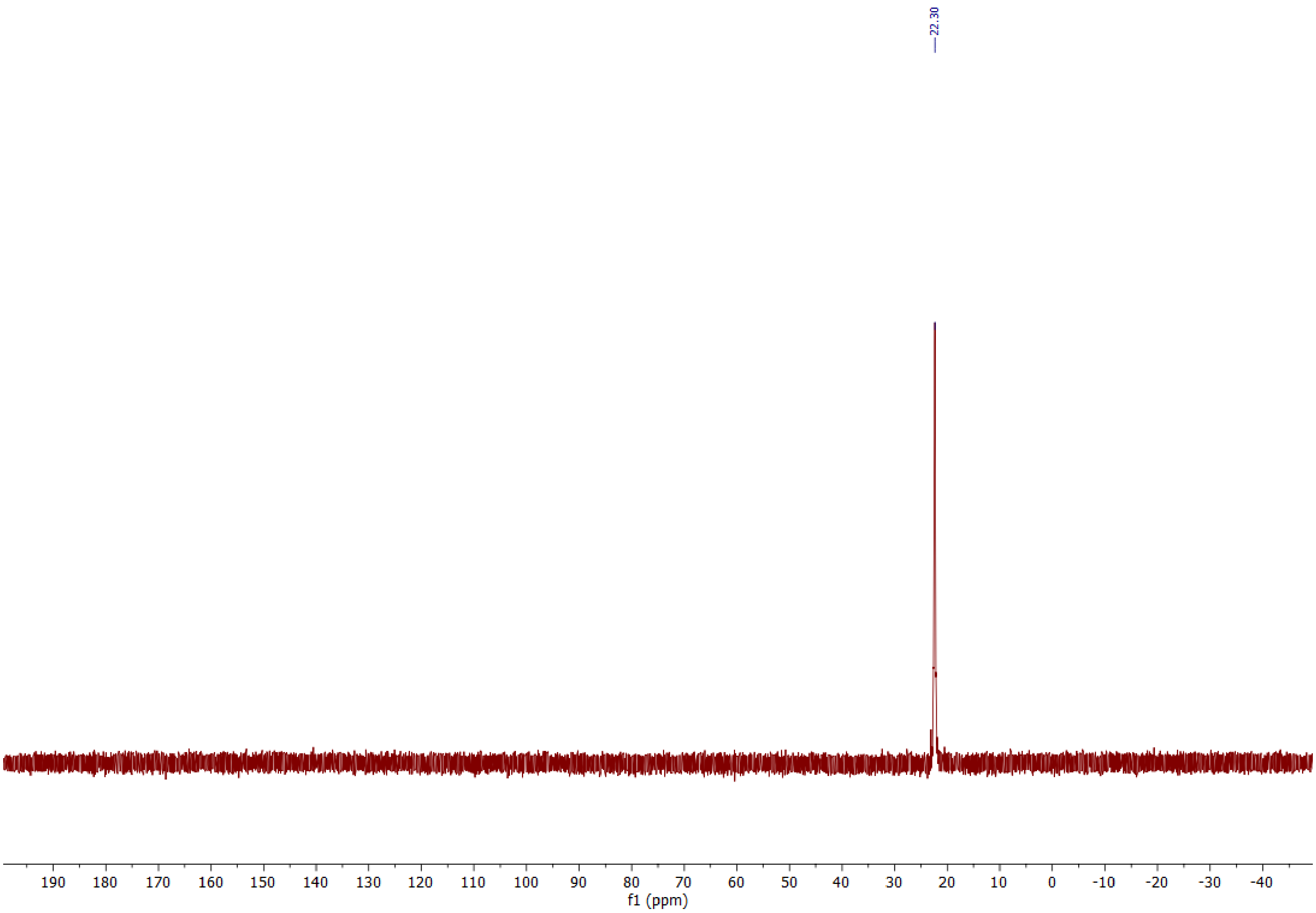

Figure S74. ^{31}P NMR spectrum of **4k** diastereomer 1.

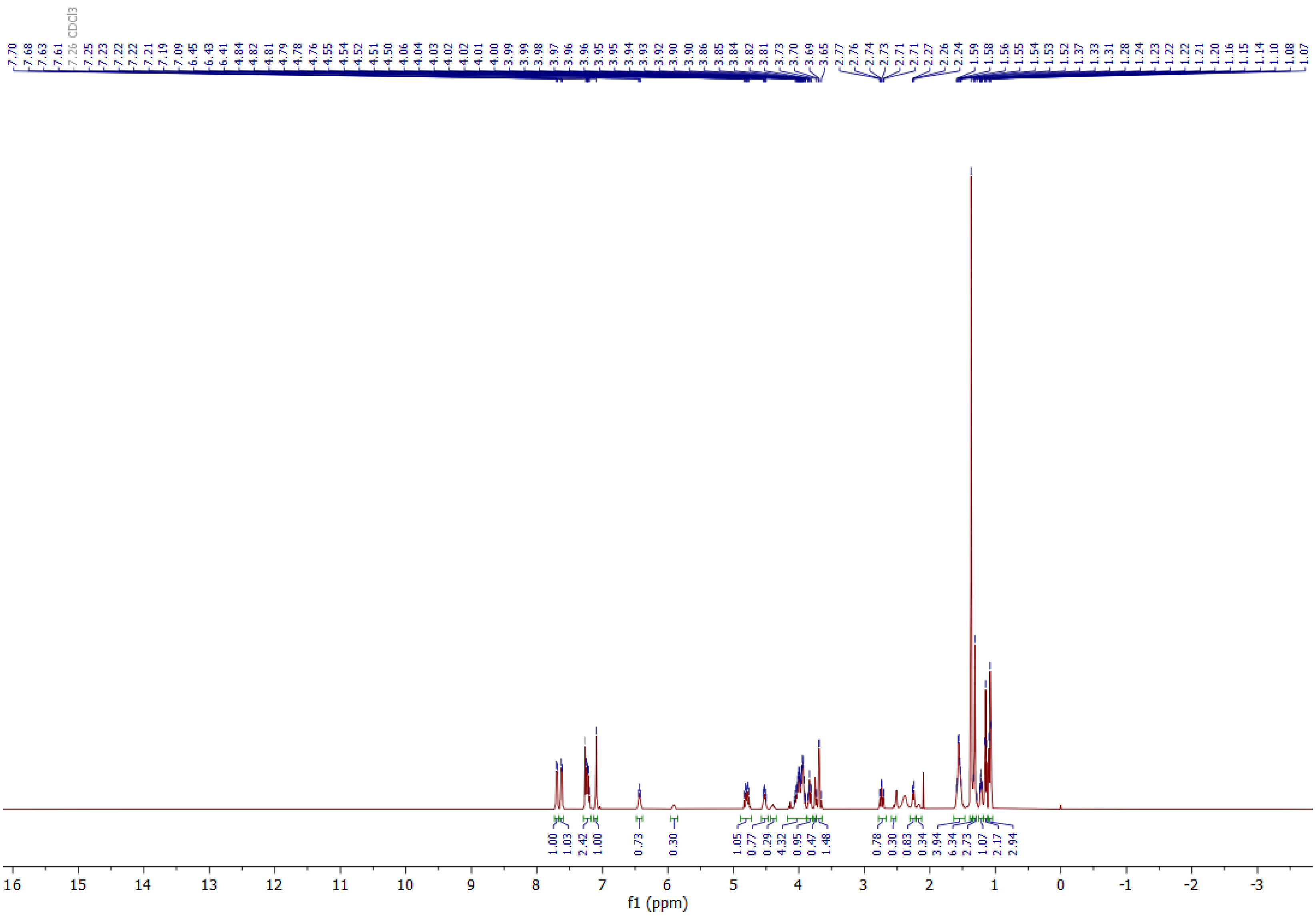

Figure S75. ^{1}H NMR spectrum of **4k** diastereomer 2.

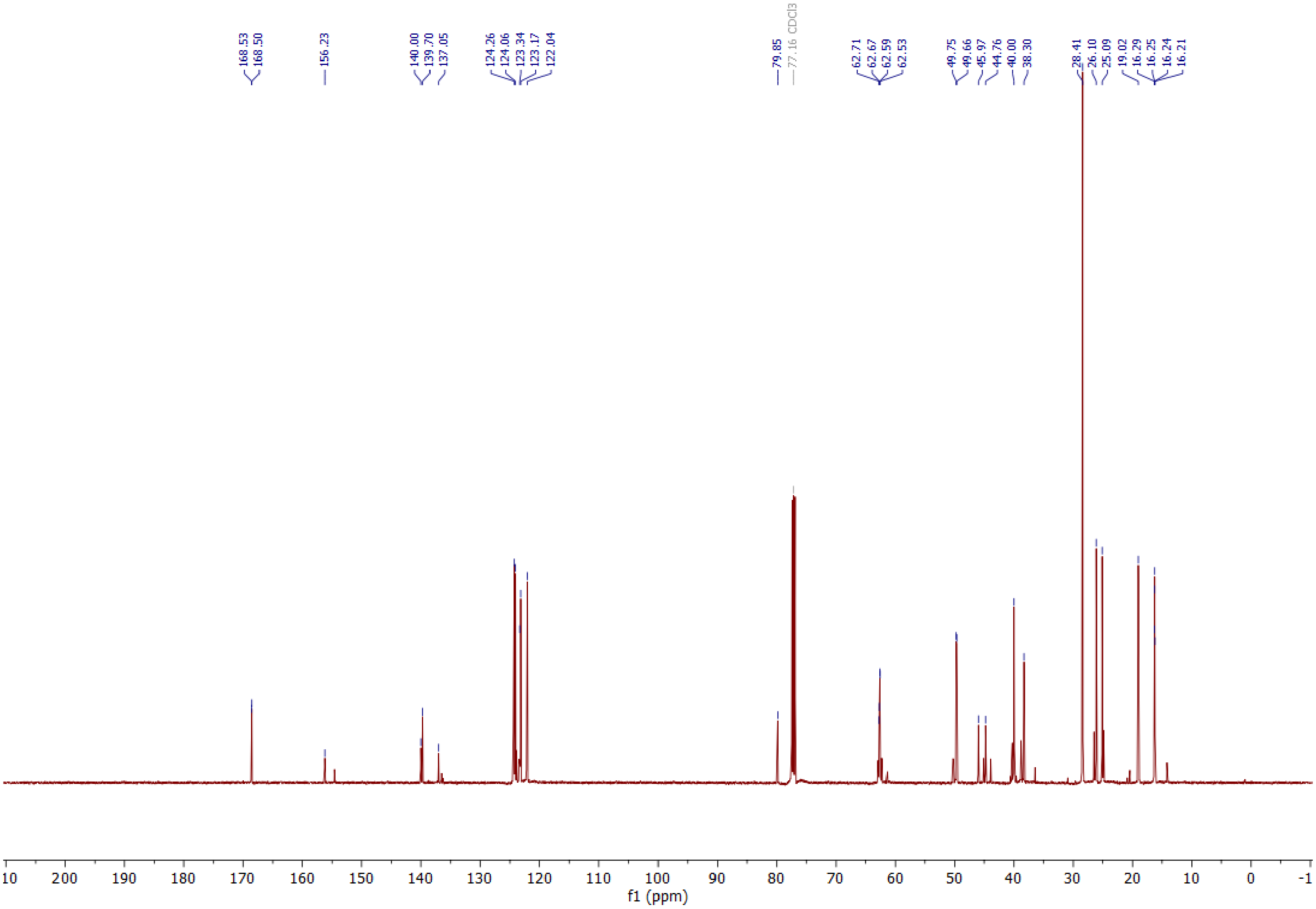

Figure S76. ^{13}C NMR spectrum of **4k** diastereomer 2.

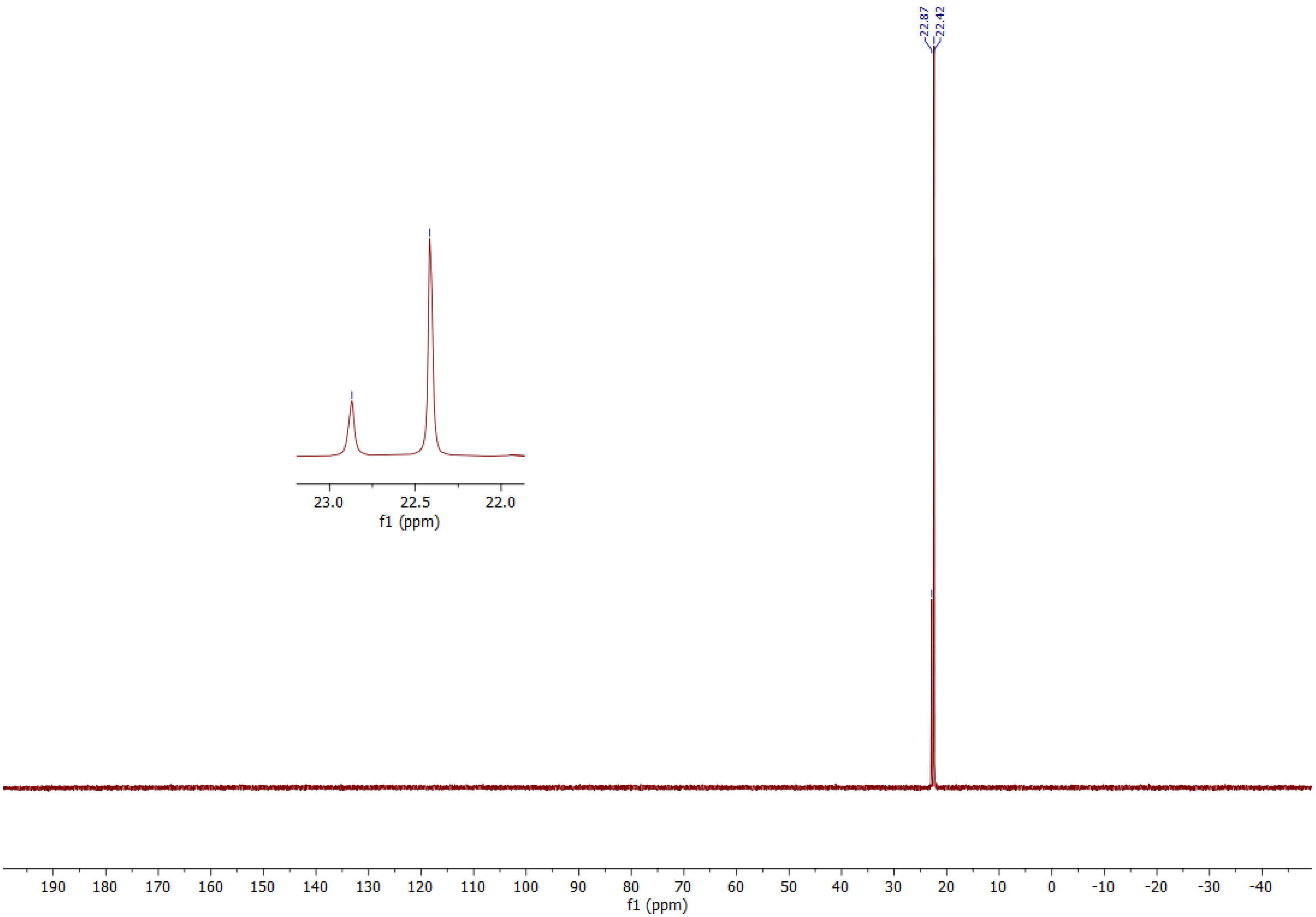

Figure S77. ^{31}P NMR spectrum of **4k** diastereomer 2.

Tert-butyl 2-((2-(benzo[*b*]thiophen-3-yl)acetamido)(diethoxyphosphoryl)methyl)piperidine-1-carboxylate (**4l**)

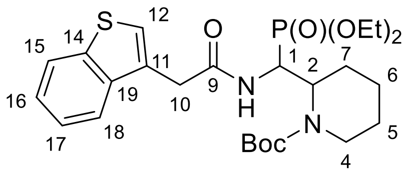

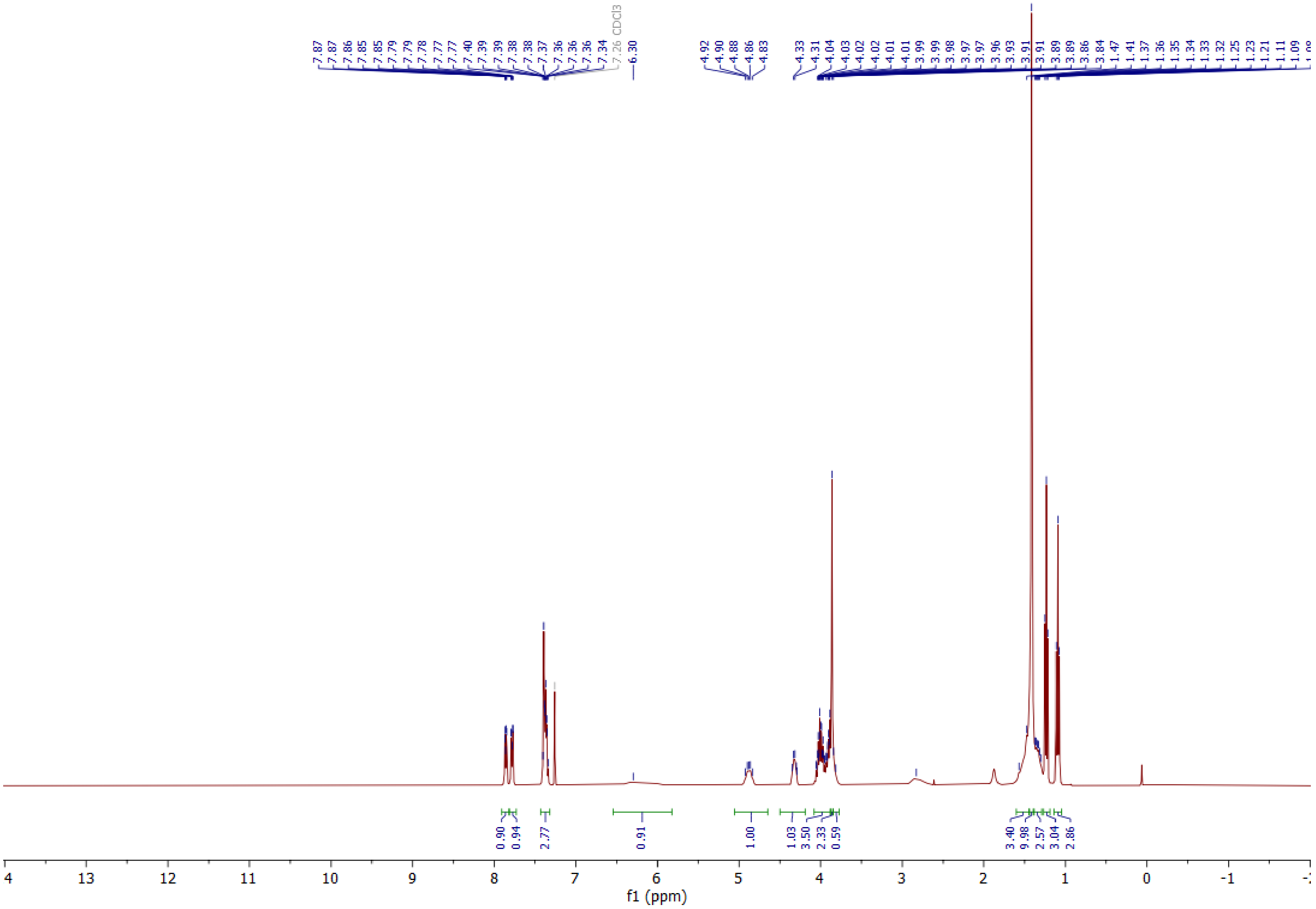

Figure S78. ^{1}H NMR spectrum of **4l** diastereomer 1.

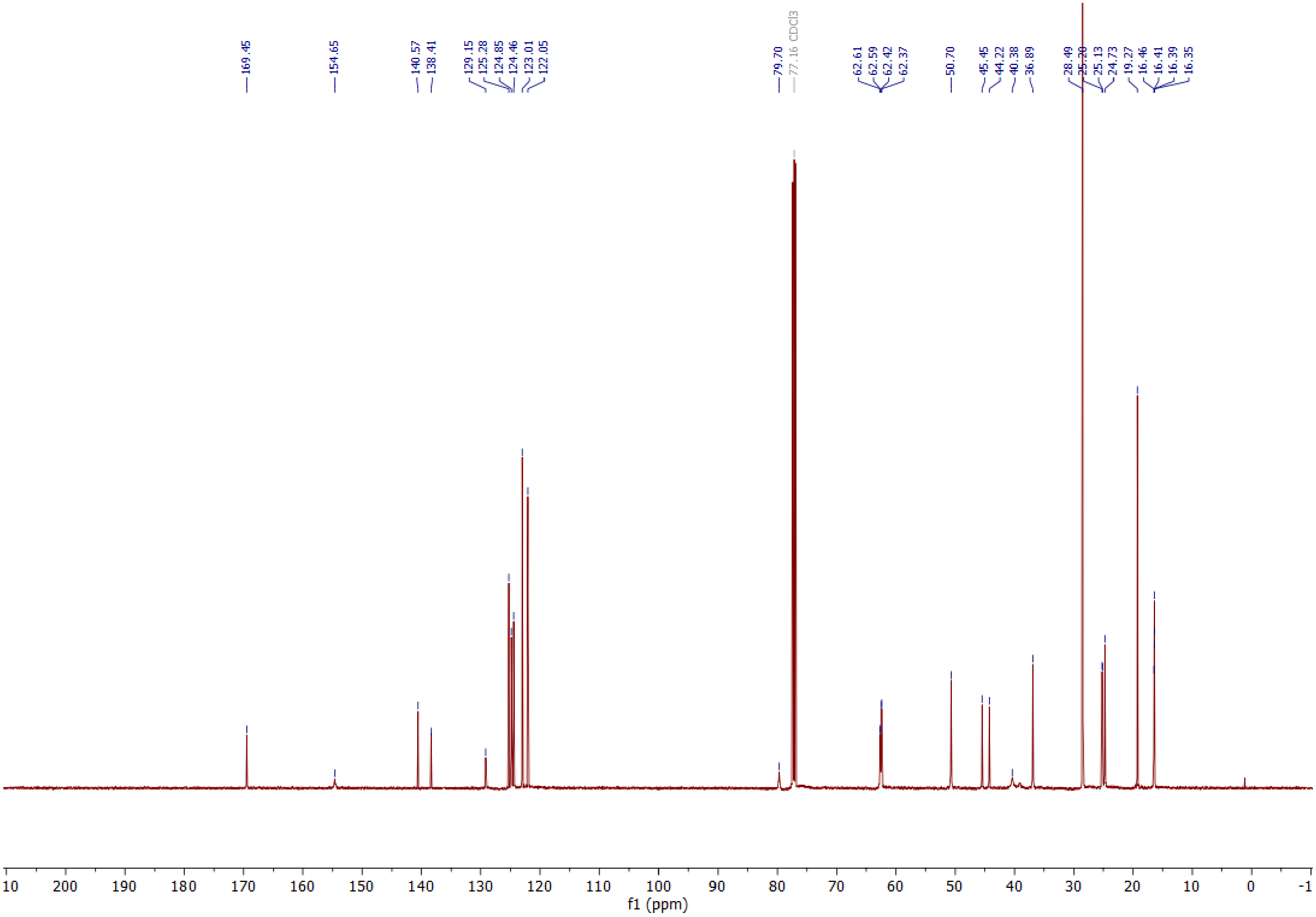

Figure S79. ^{13}C NMR spectrum of **4l** diastereomer 1.

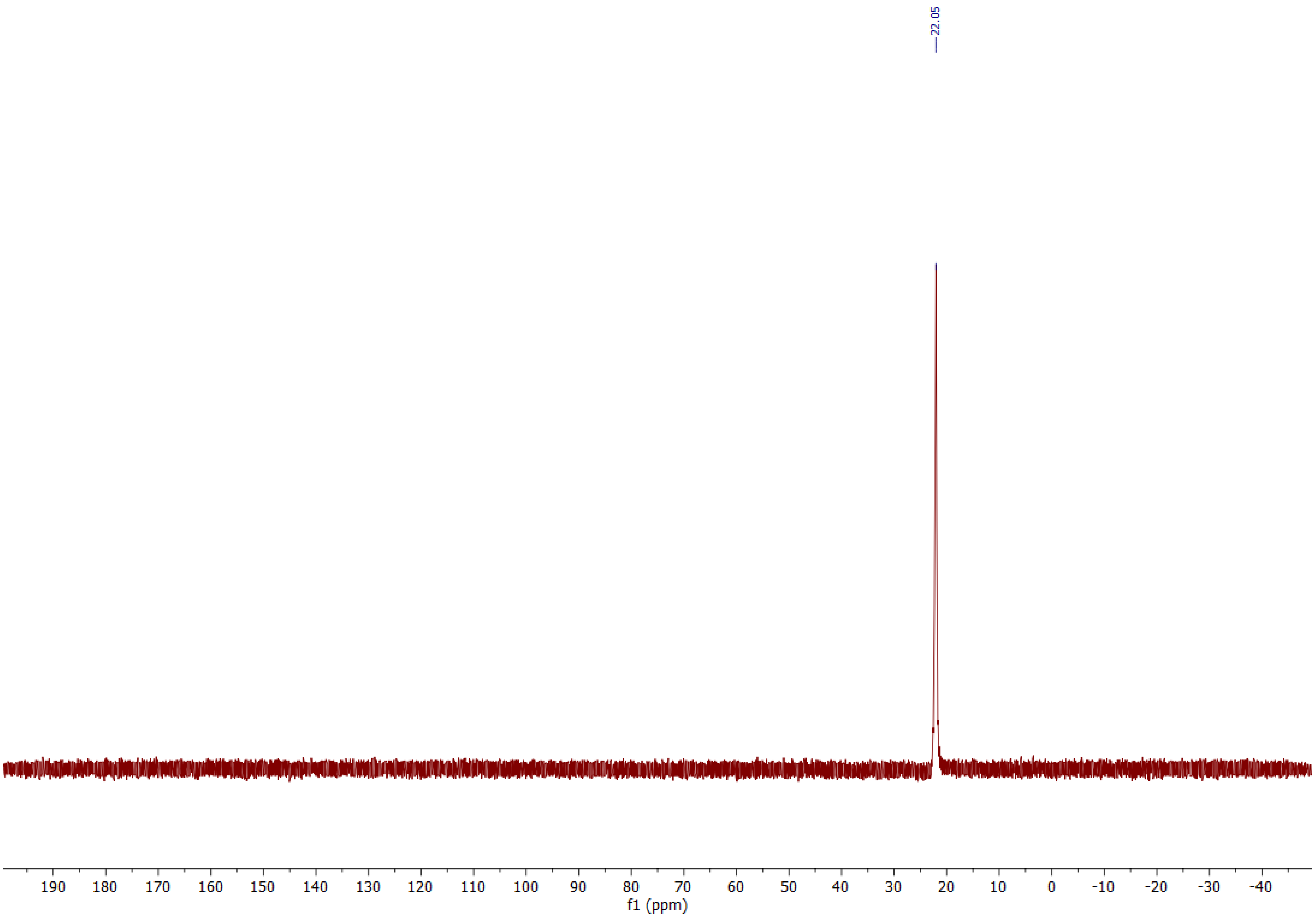

Figure S80. ^{31}P NMR spectrum of **4l** diastereomer 1.

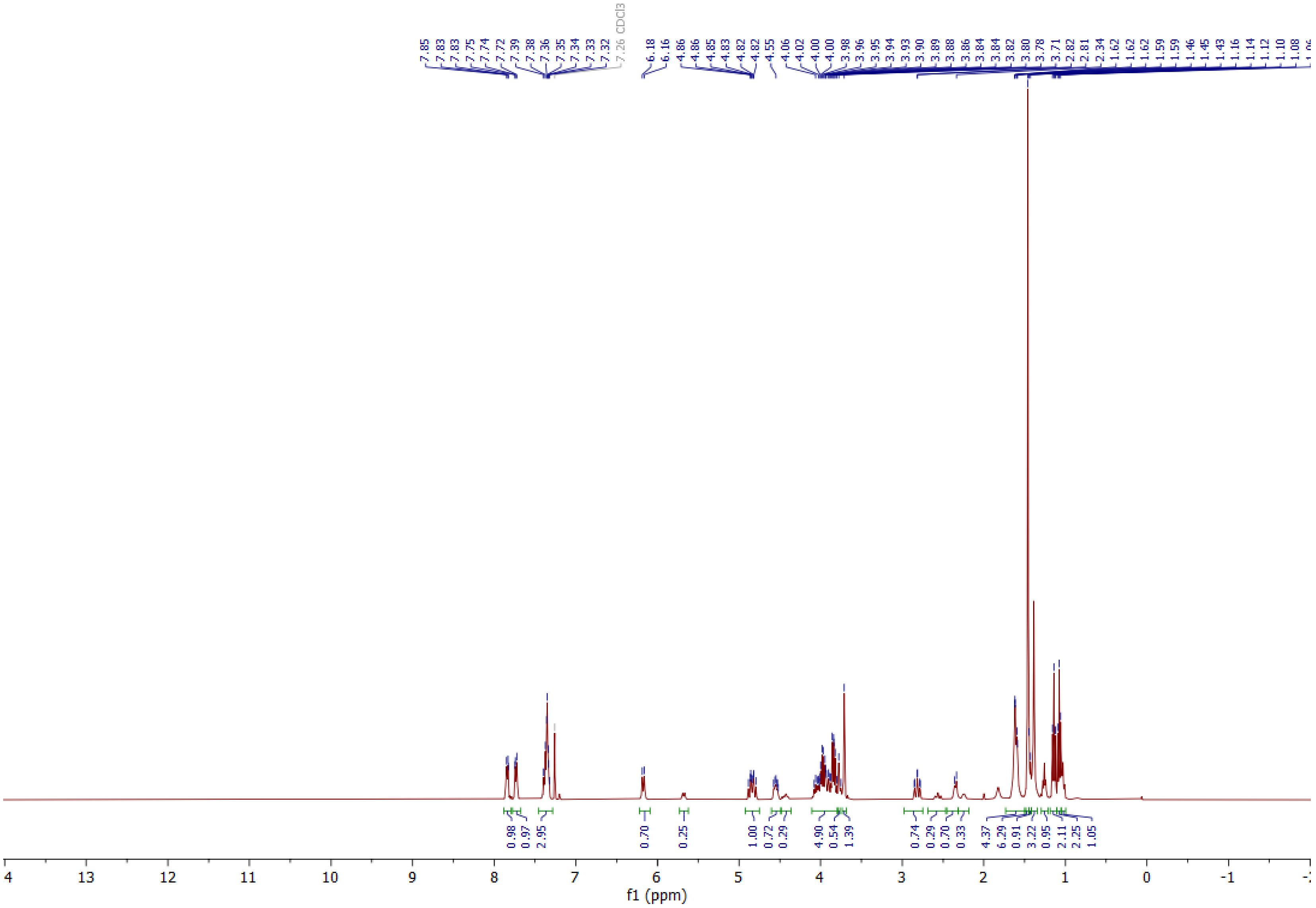

Figure S81. ^{1}H NMR spectrum of **4l** diastereomer 2.

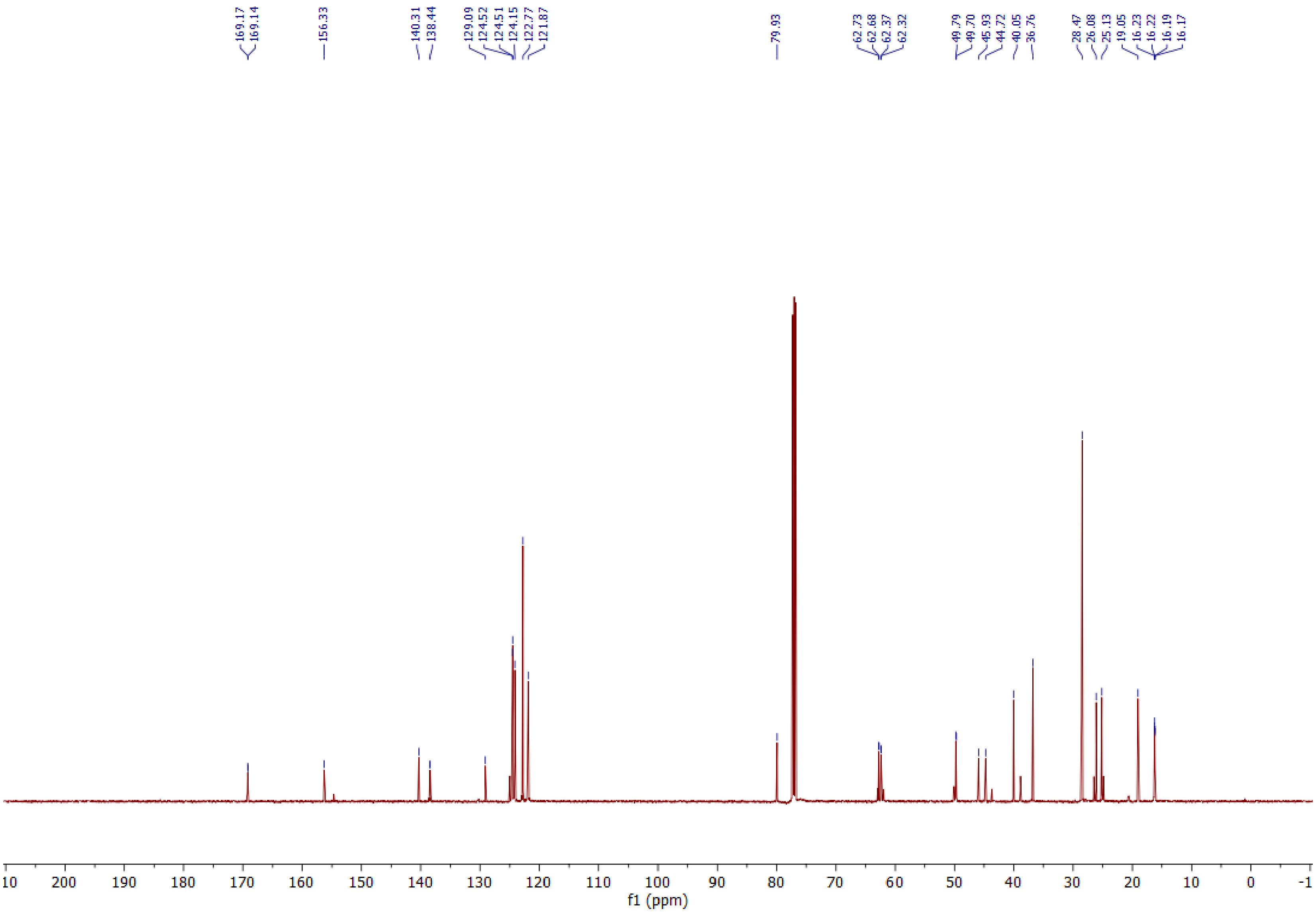

Figure S82. ^{13}C NMR spectrum of **4l** diastereomer 2.

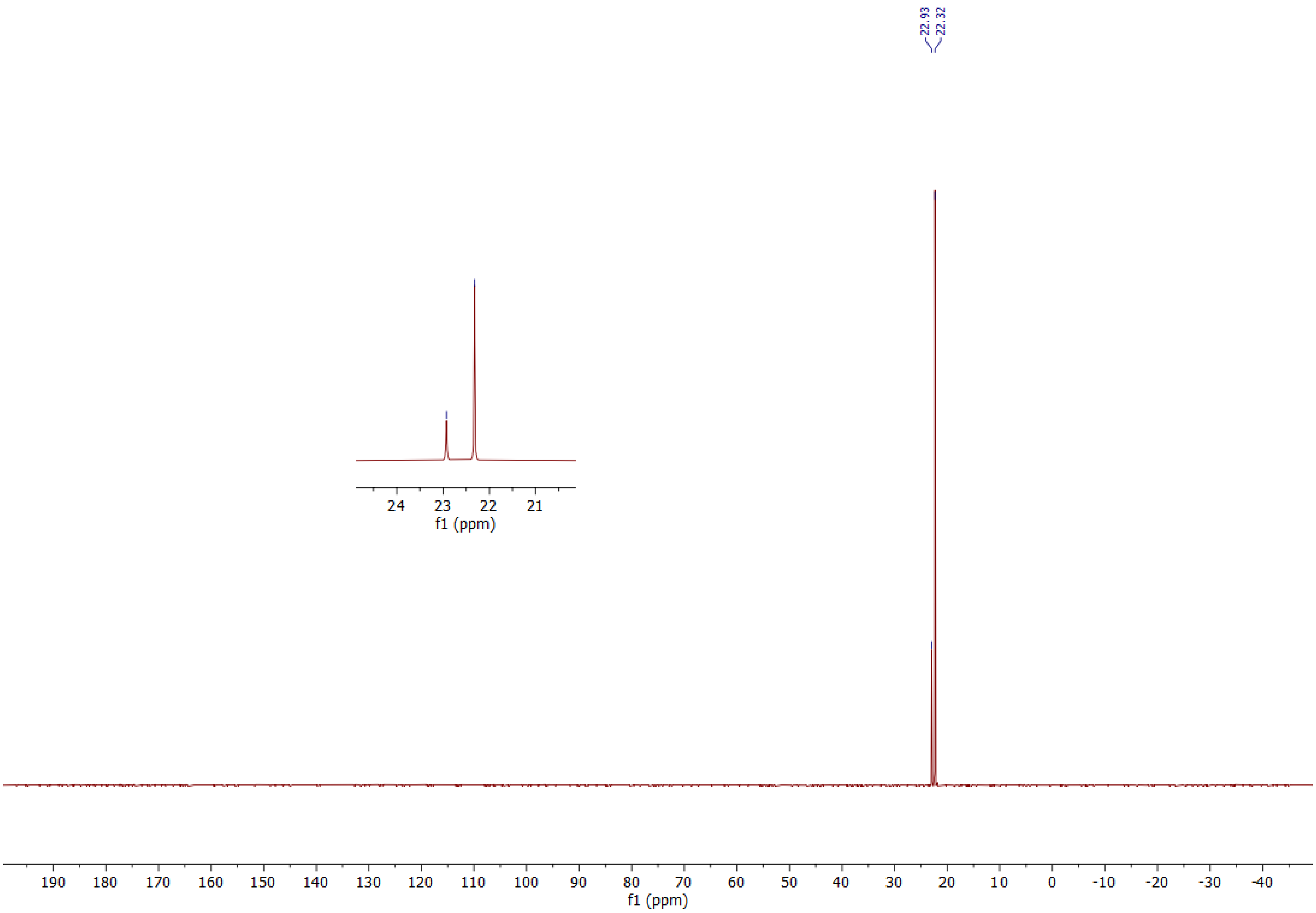

Figure S83. ^{31}P NMR spectrum of **4l** diastereomer 2.

((2-(4-Methyl-2-thioxo-2,3-dihydrothiazol-5-yl)acetamido)(piperidin-2-yl)methyl)phosphonic acid hydrochloride (**5a**)

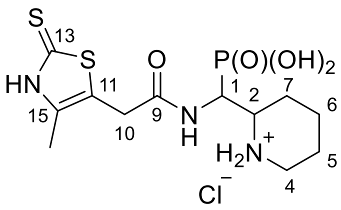

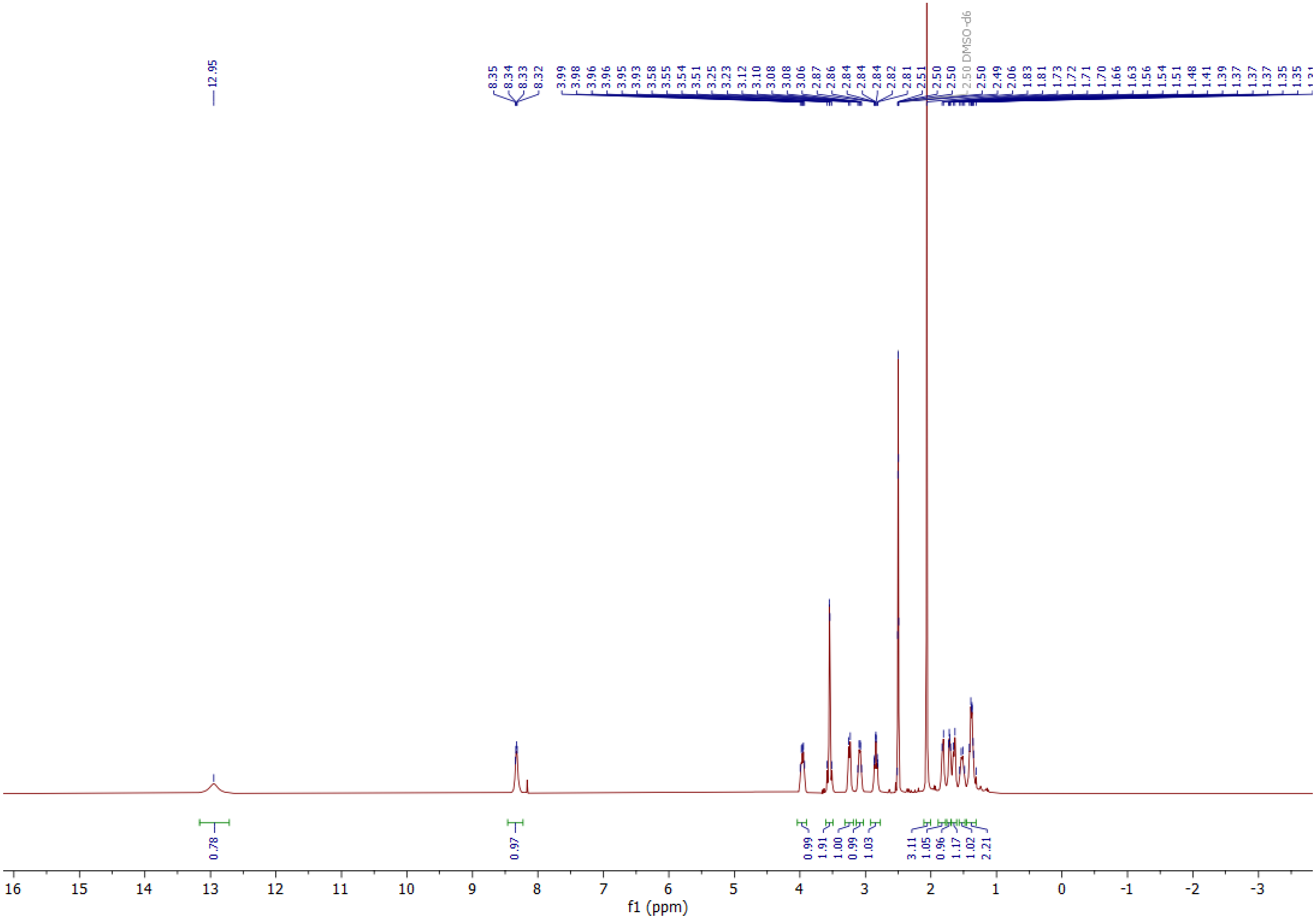

Figure S84. ^{1}H NMR spectrum of **5a** diastereomer 1.

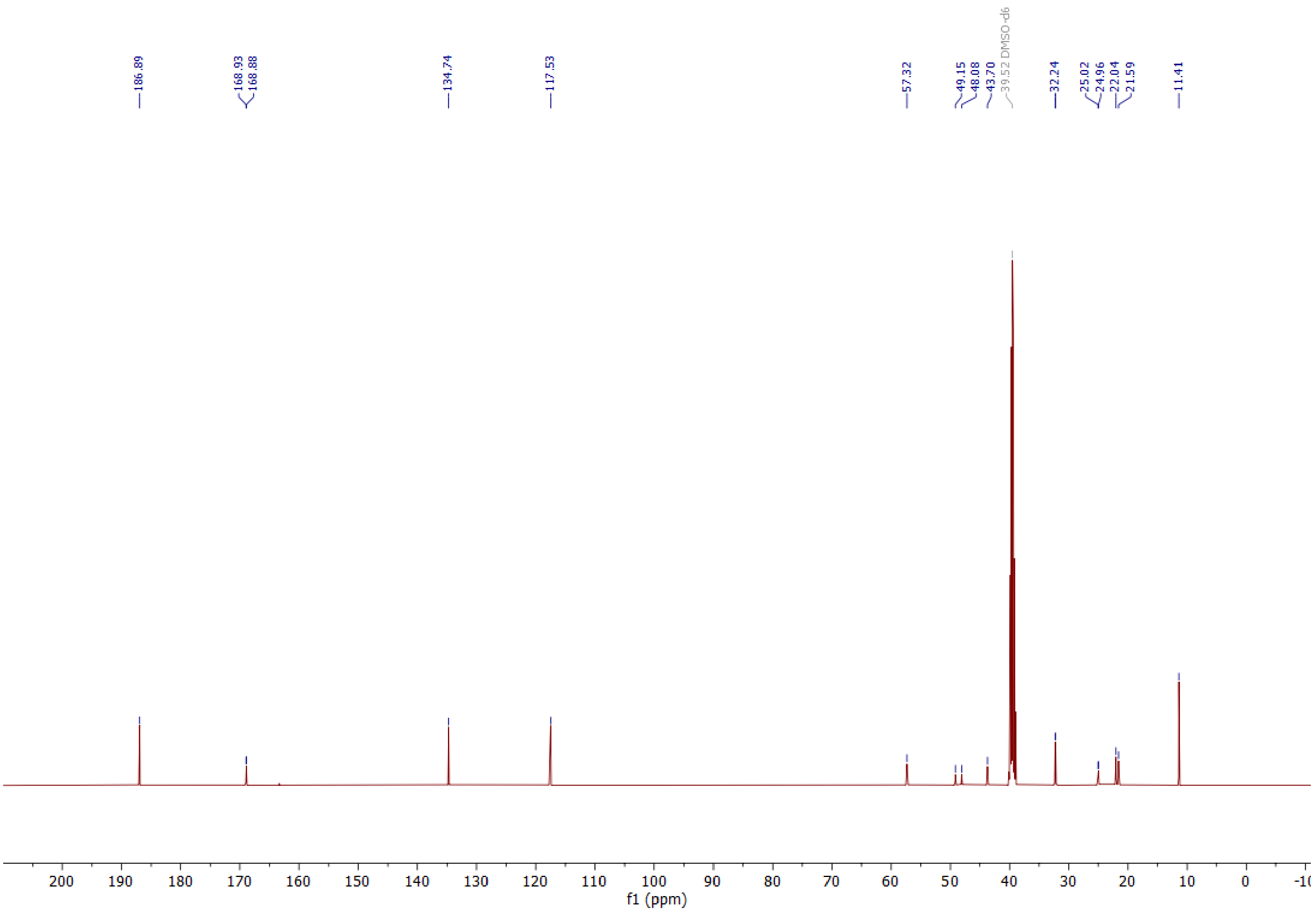

Figure S85. ^{13}C NMR spectrum of **5a** diastereomer 1.

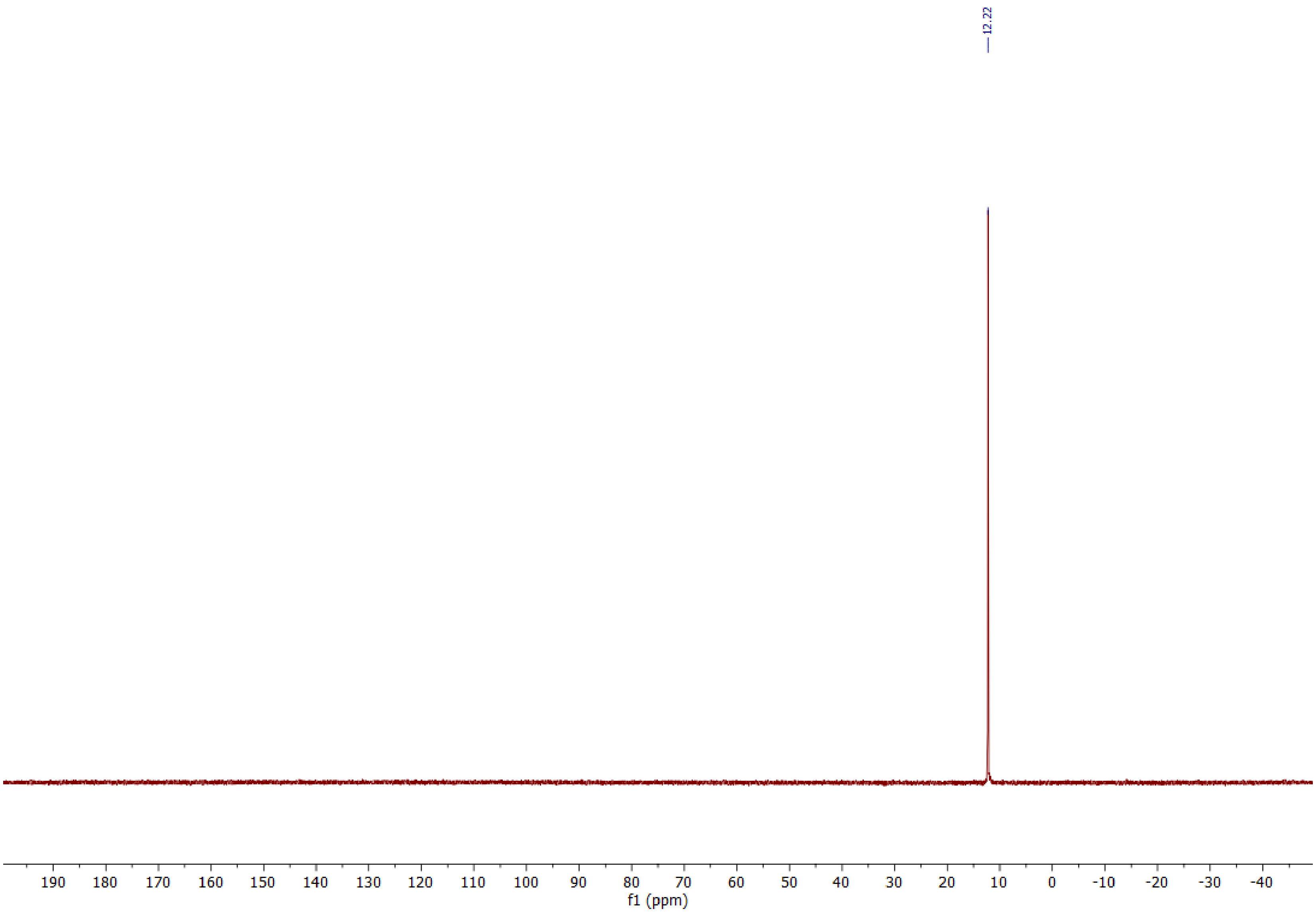

Figure S86. ^{31}P NMR spectrum of **5a** diastereomer 1.

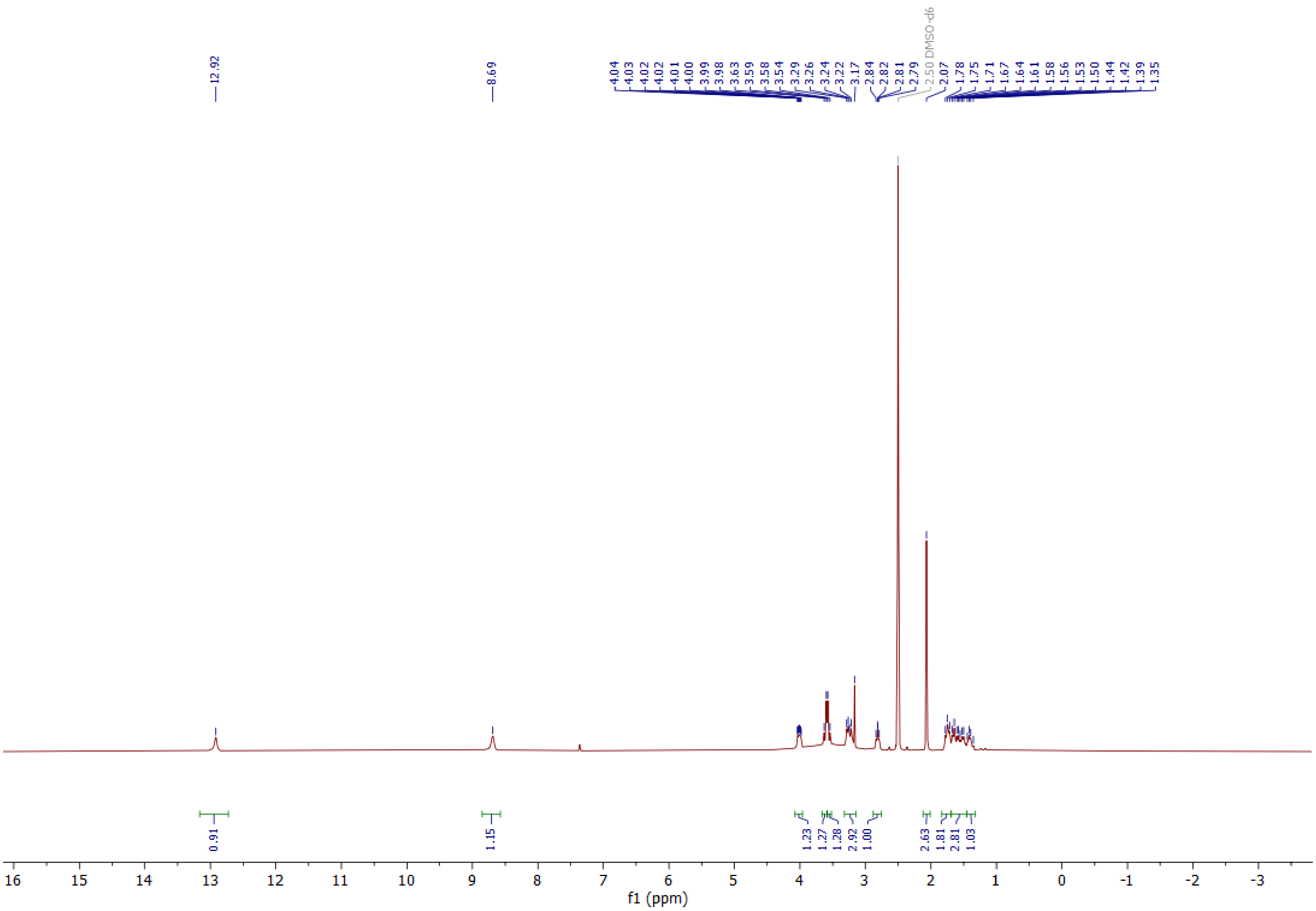

Figure S87. ^{1}H NMR spectrum of **5a** diastereomer 2.

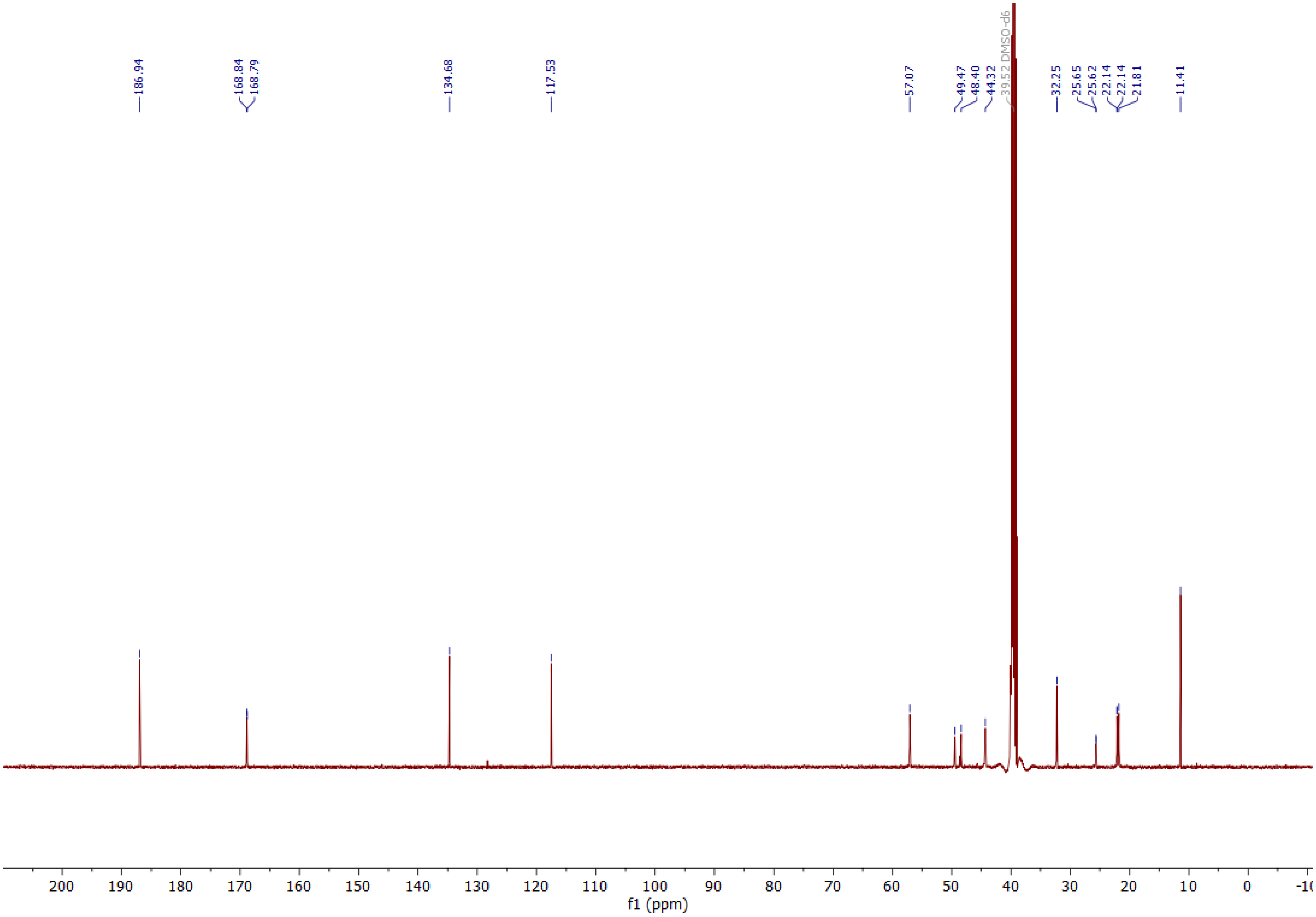

Figure S88. ^{13}C NMR spectrum of **5a** diastereomer 2.

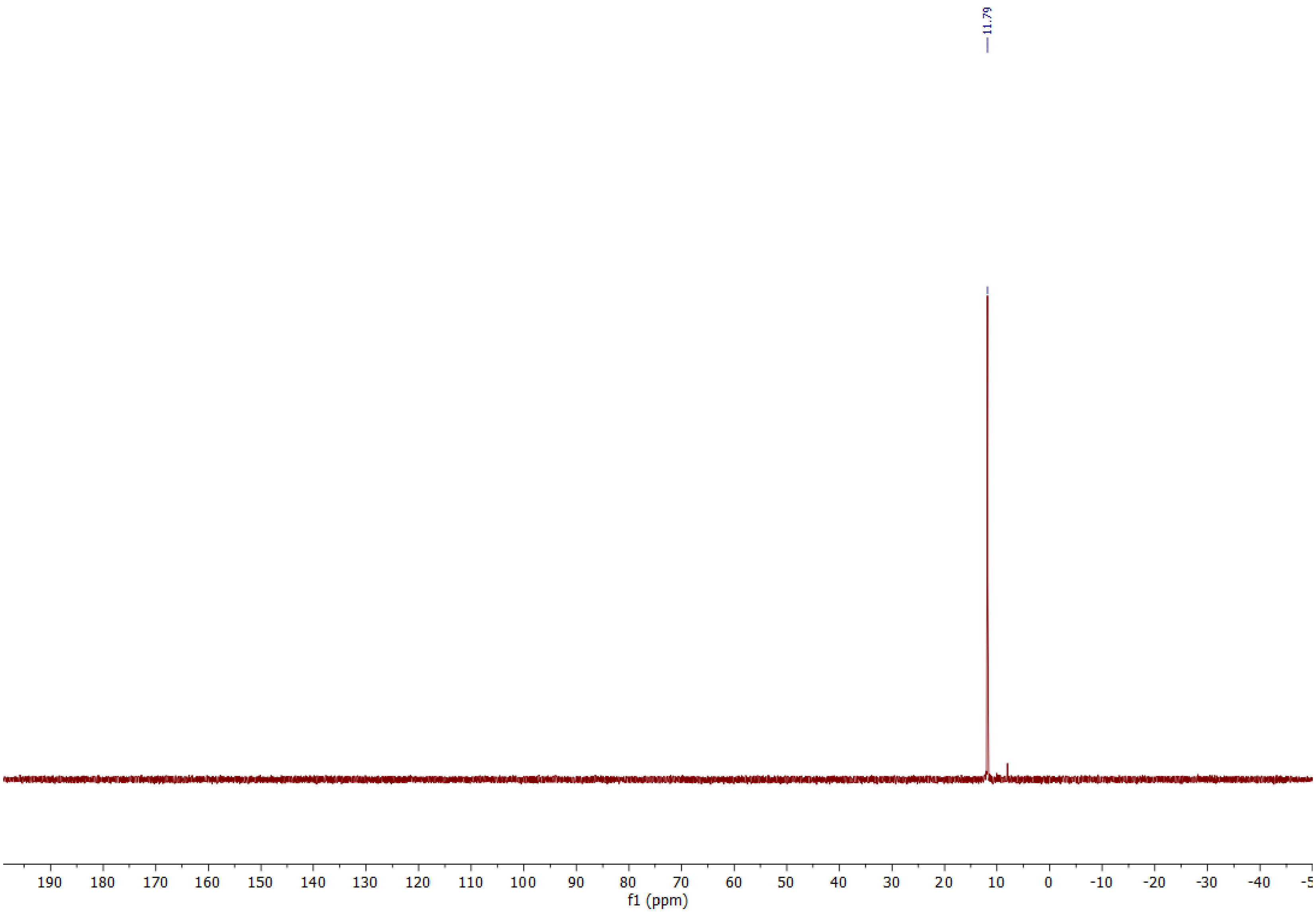

Figure S89. ^{31}P NMR spectrum of **5a** diastereomer 2.

(Piperidin-2-yl(thiophene-2-carboxamido)methyl)phosphonic acid hydrochloride (**5b**)

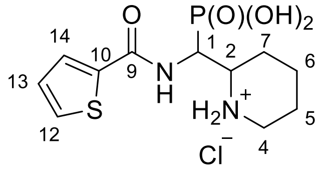

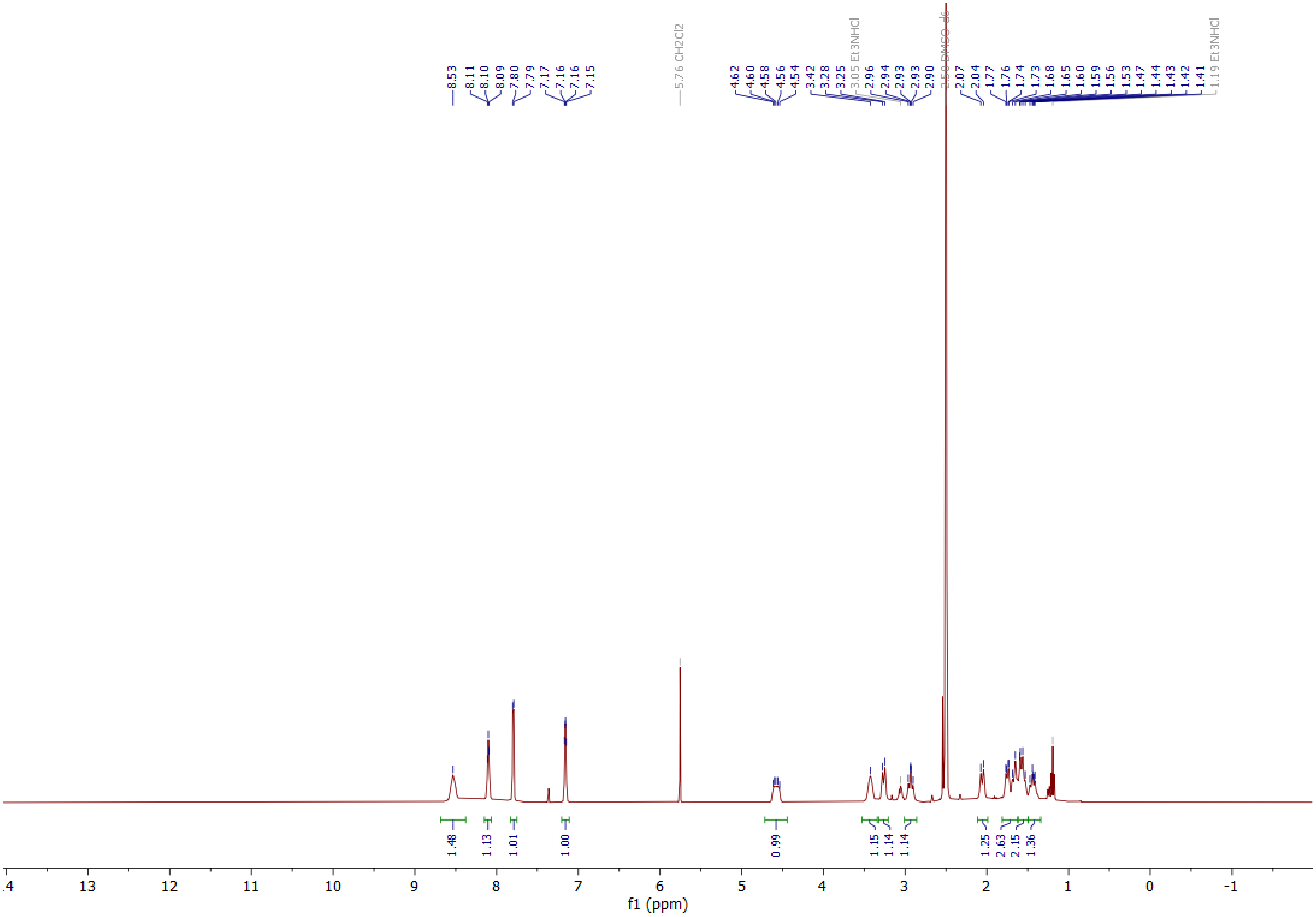

Figure S90. ^{1}H NMR spectrum of **5b** diastereomer 1.

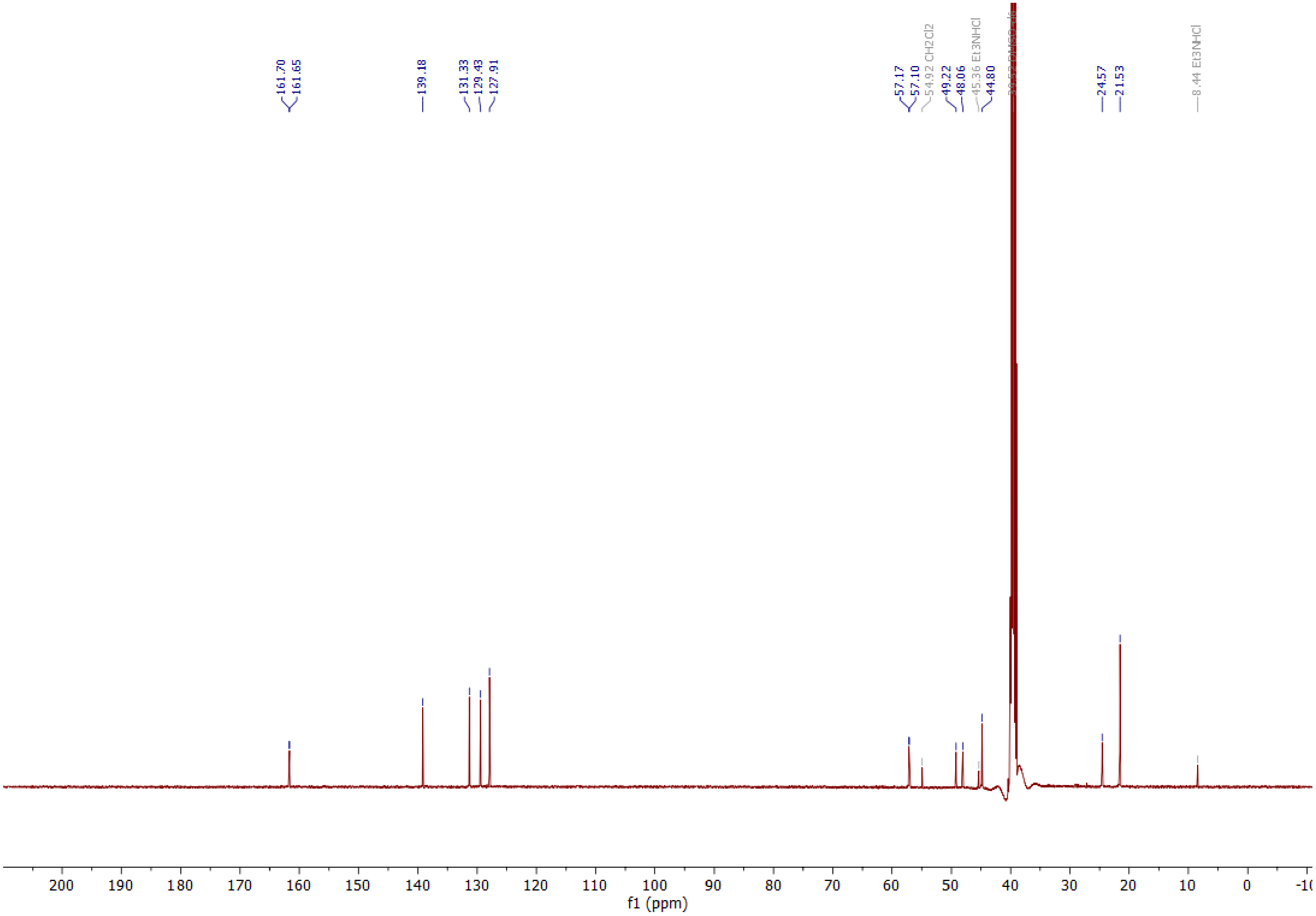

Figure S91. ^{13}C NMR spectrum of **5b** diastereomer 1.

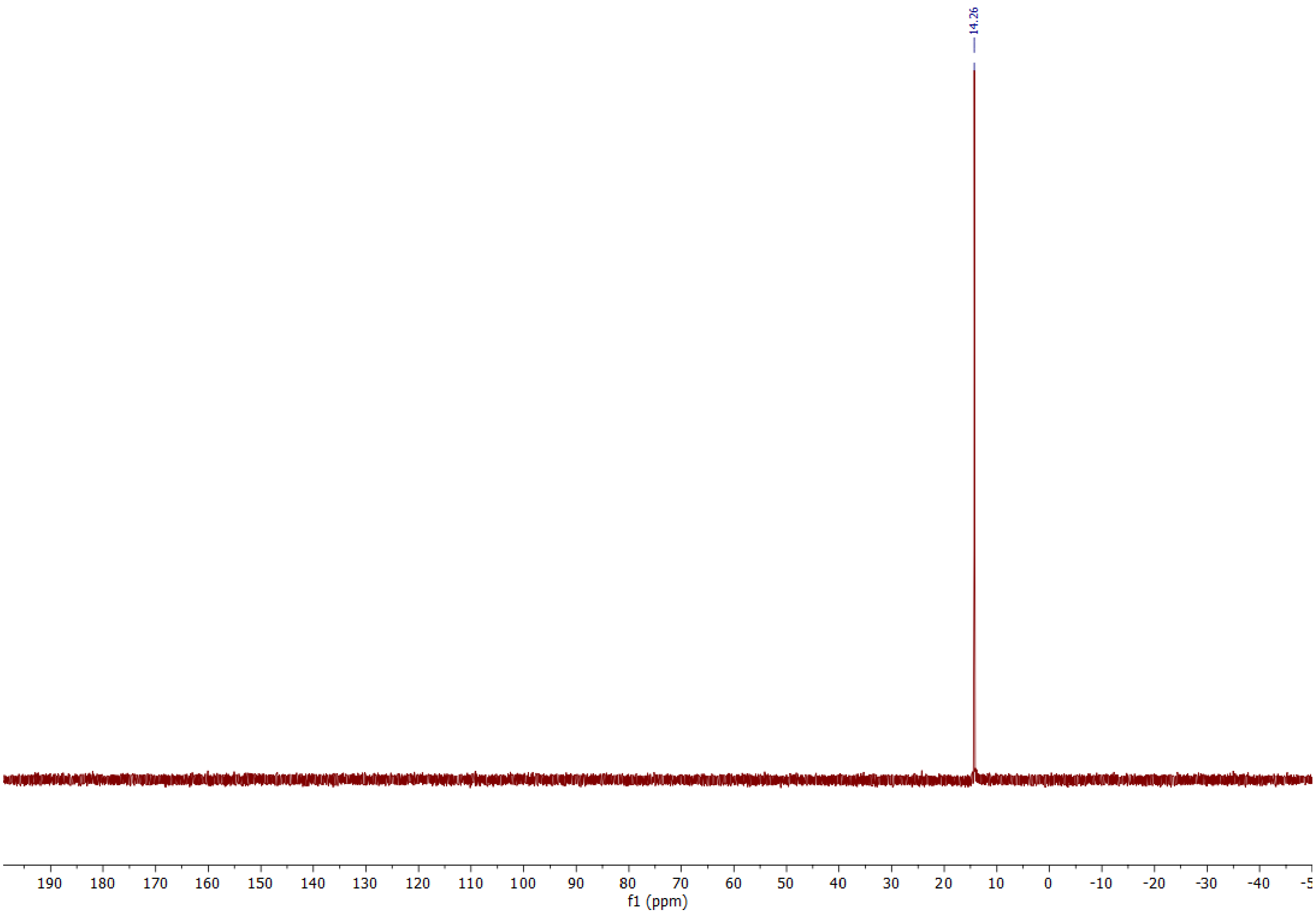

Figure S92. ^{31}P NMR spectrum of **5b** diastereomer 1.

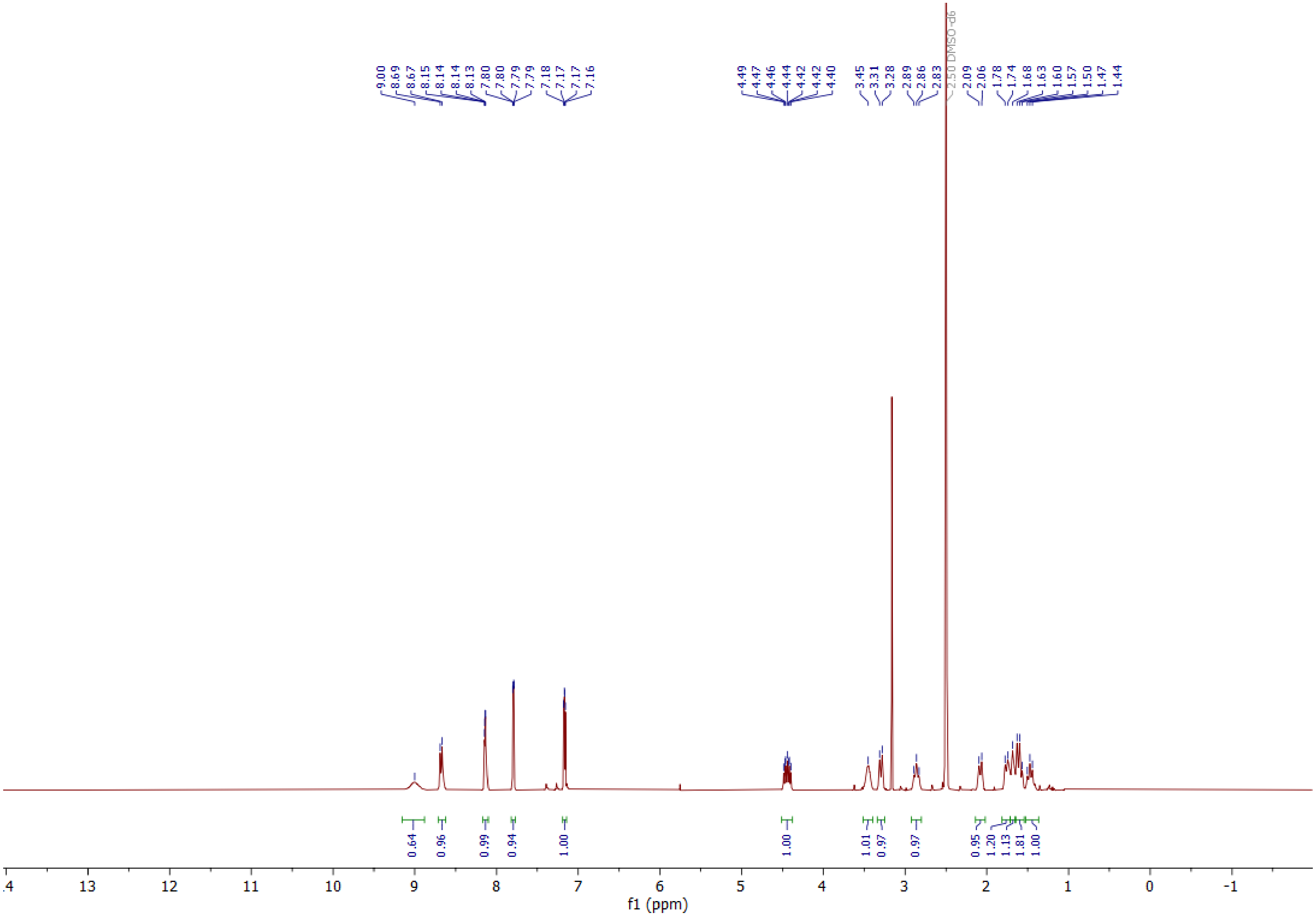

Figure S93. ^{1}H NMR spectrum of **5b** diastereomer 2.

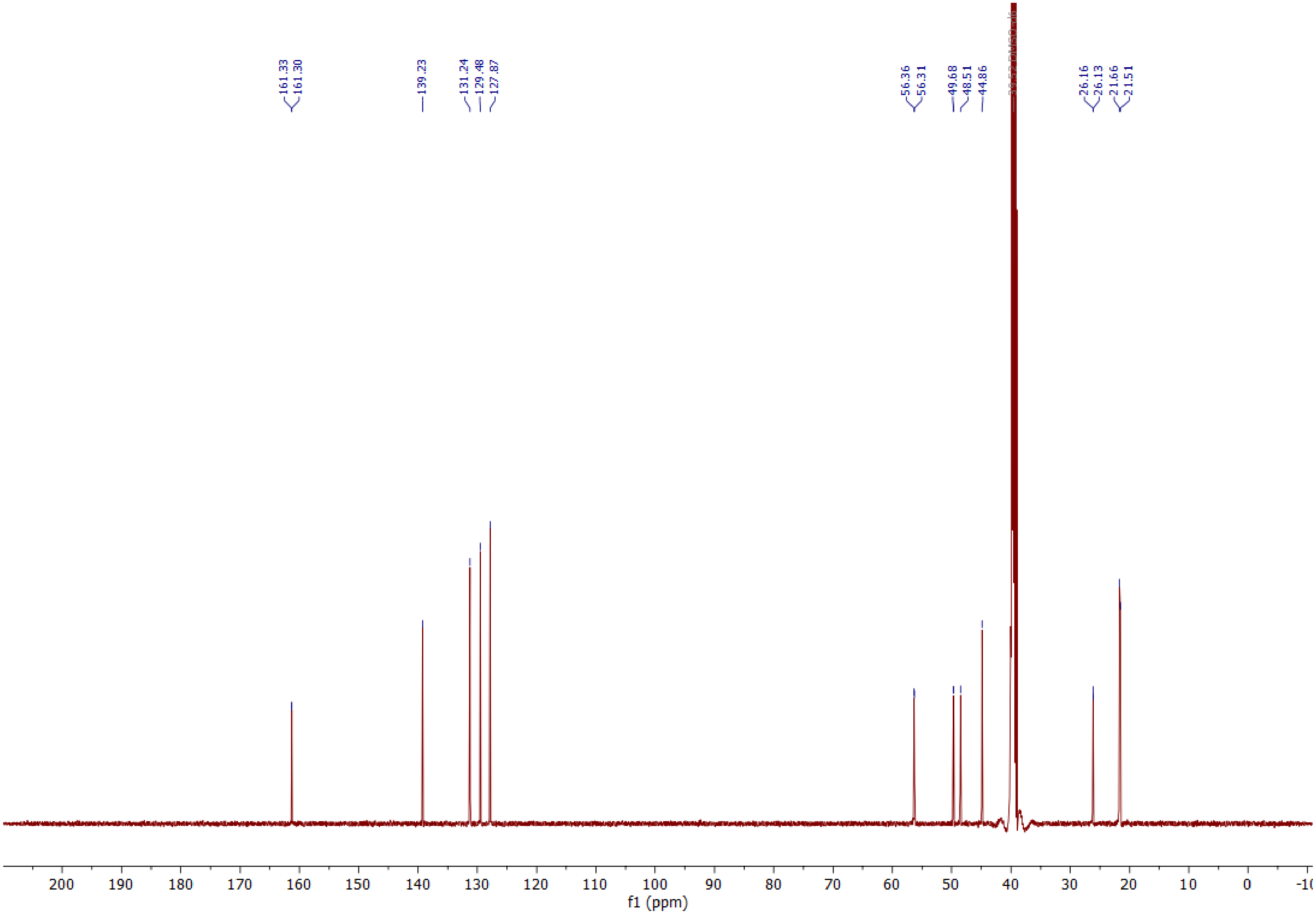

Figure S94. ^{13}C NMR spectrum of **5b** diastereomer 2.

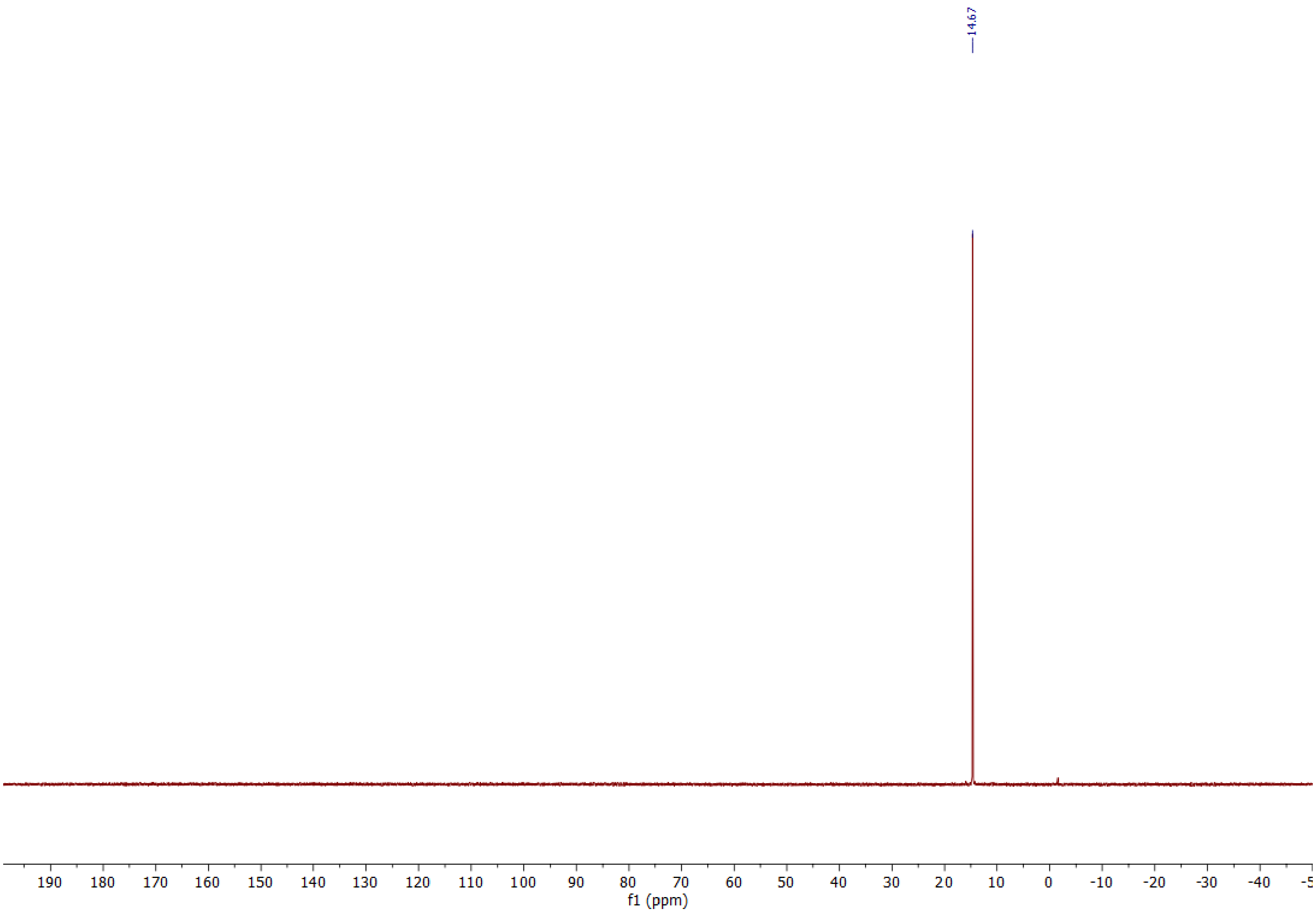

Figure S95. ^{31}P NMR spectrum of **5b** diastereomer 2.

(Piperidin-2-yl(thiophene-3-carboxamido)methyl)phosphonic acid hydrochloride (**5c**)

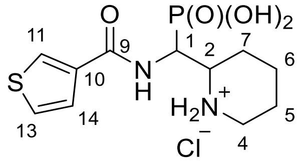

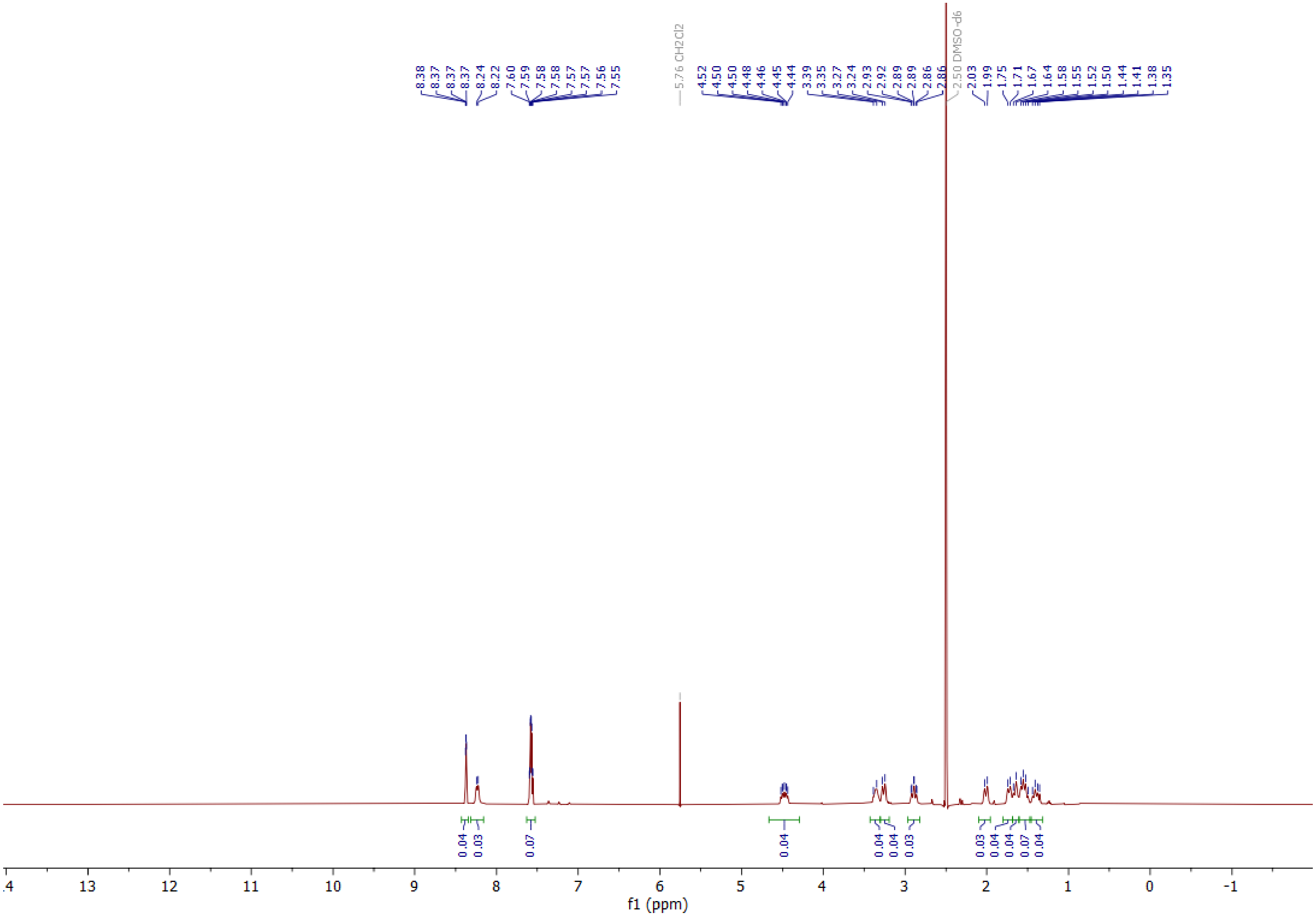

Figure S96. ^{1}H NMR spectrum of **5c** diastereomer 1.

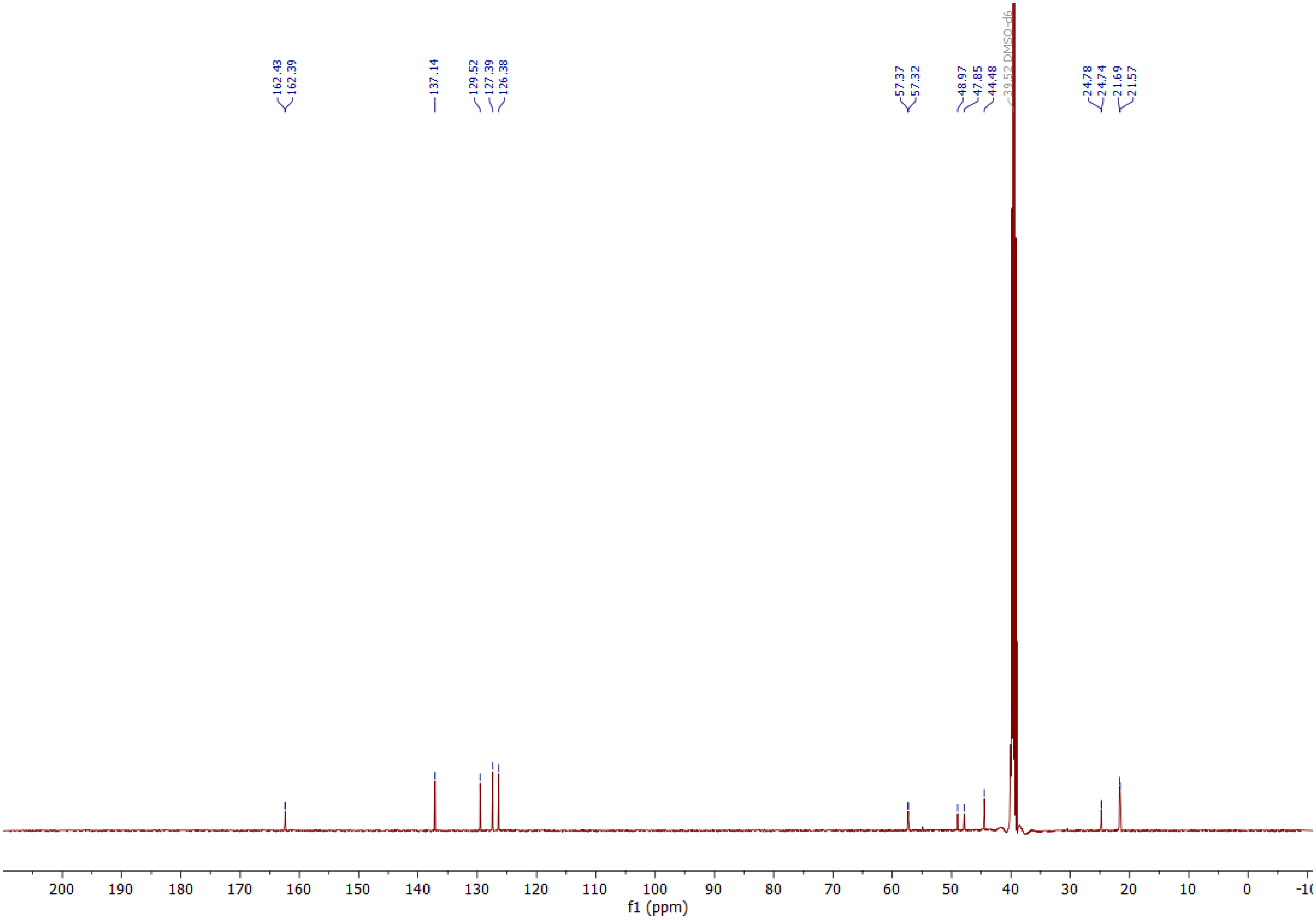

Figure S97. ^{13}C NMR spectrum of **5c** diastereomer 1.

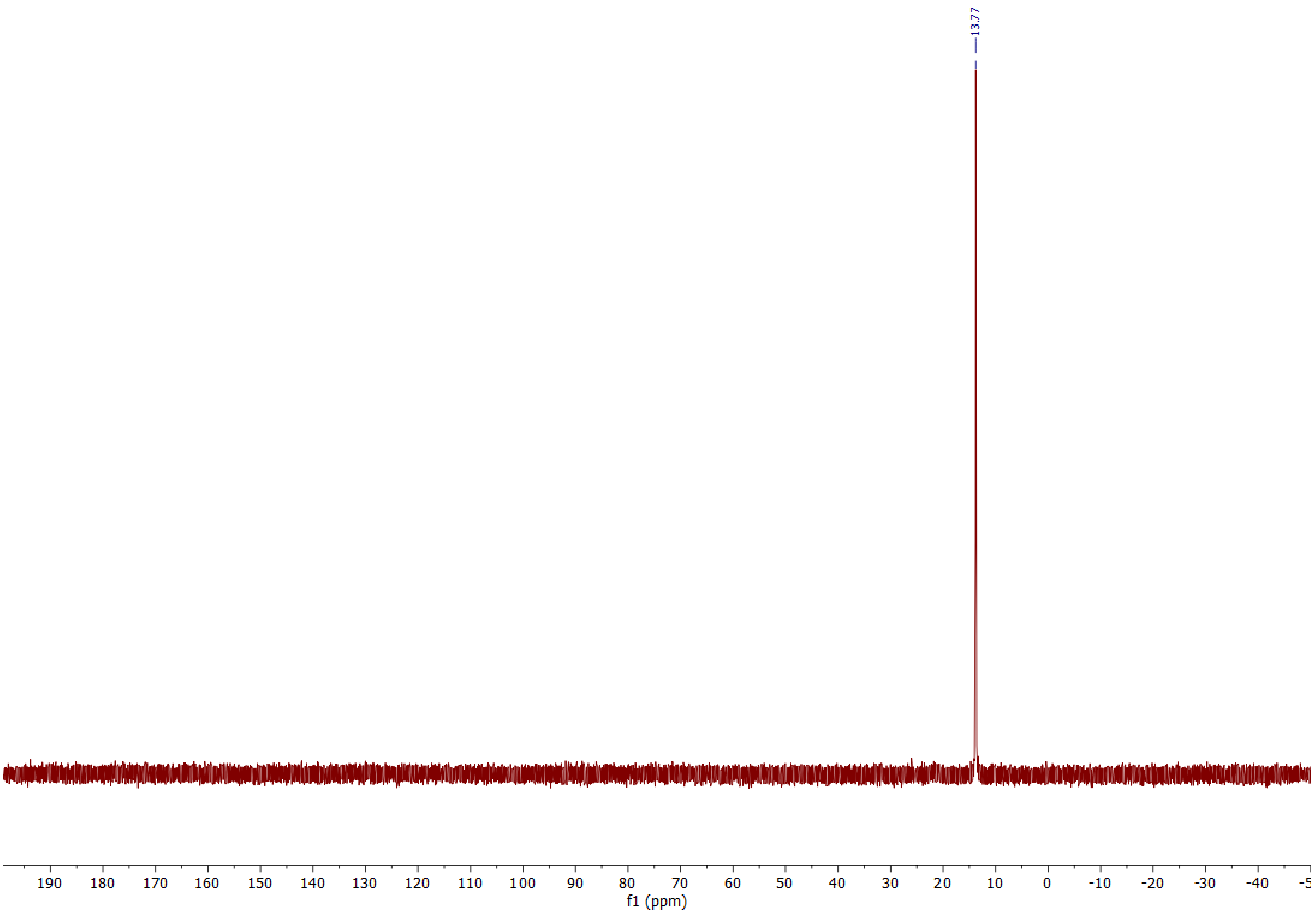

Figure S98. ^{31}P NMR spectrum of **5c** diastereomer 1.

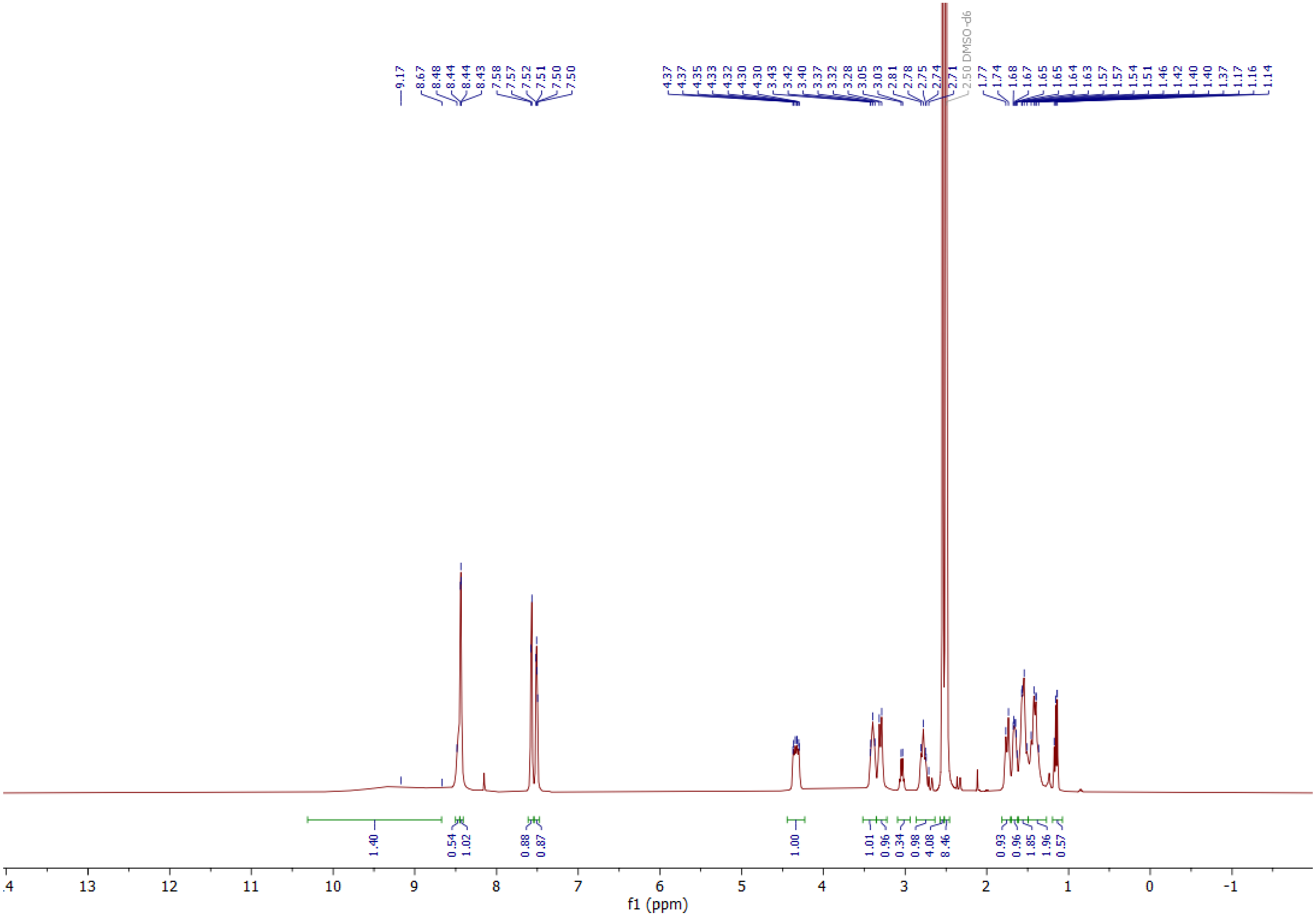

Figure S99. ^{1}H NMR spectrum of **5c** diastereomer 2.

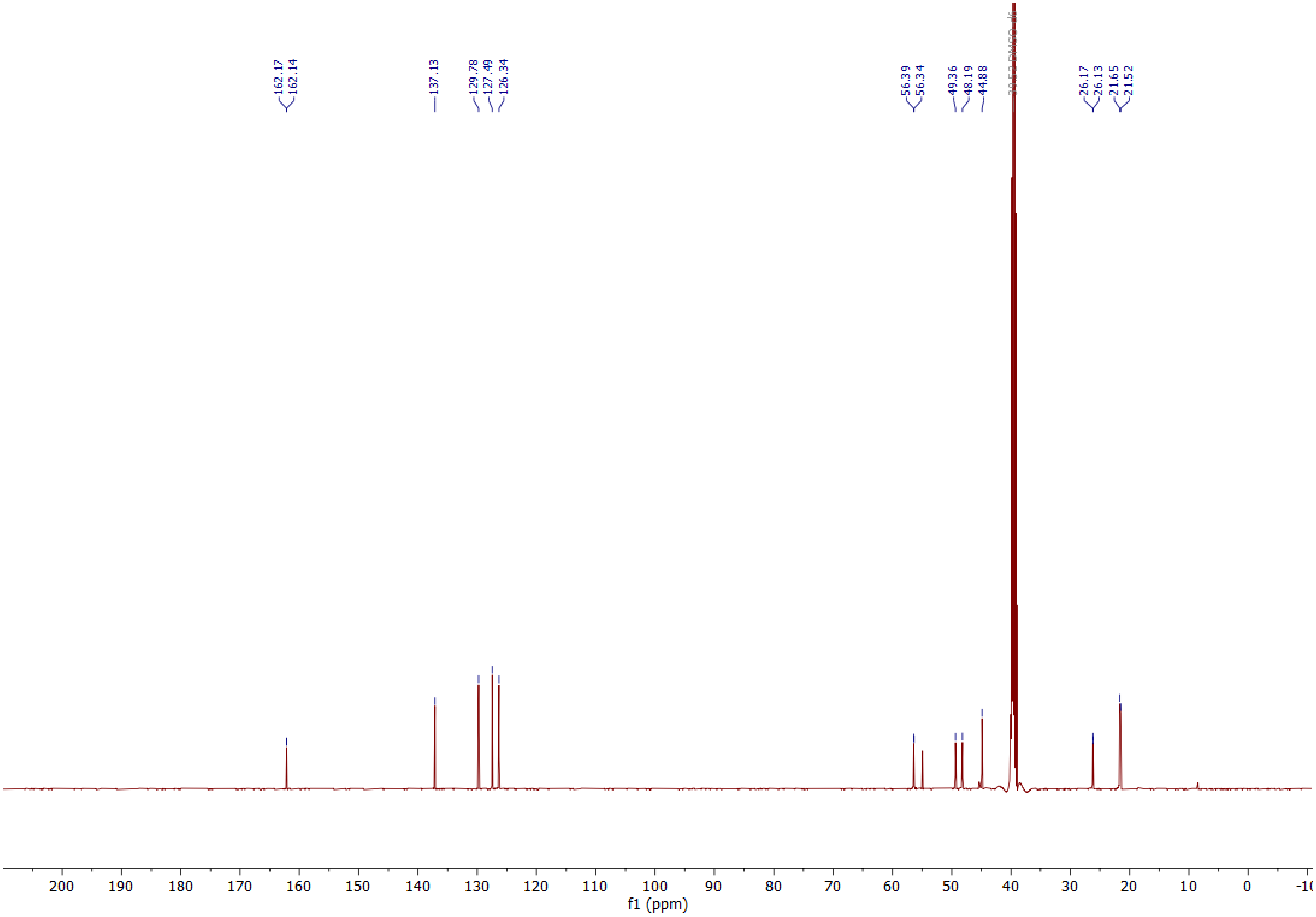

Figure S100. ^{13}C NMR spectrum of **5c** diastereomer 2.

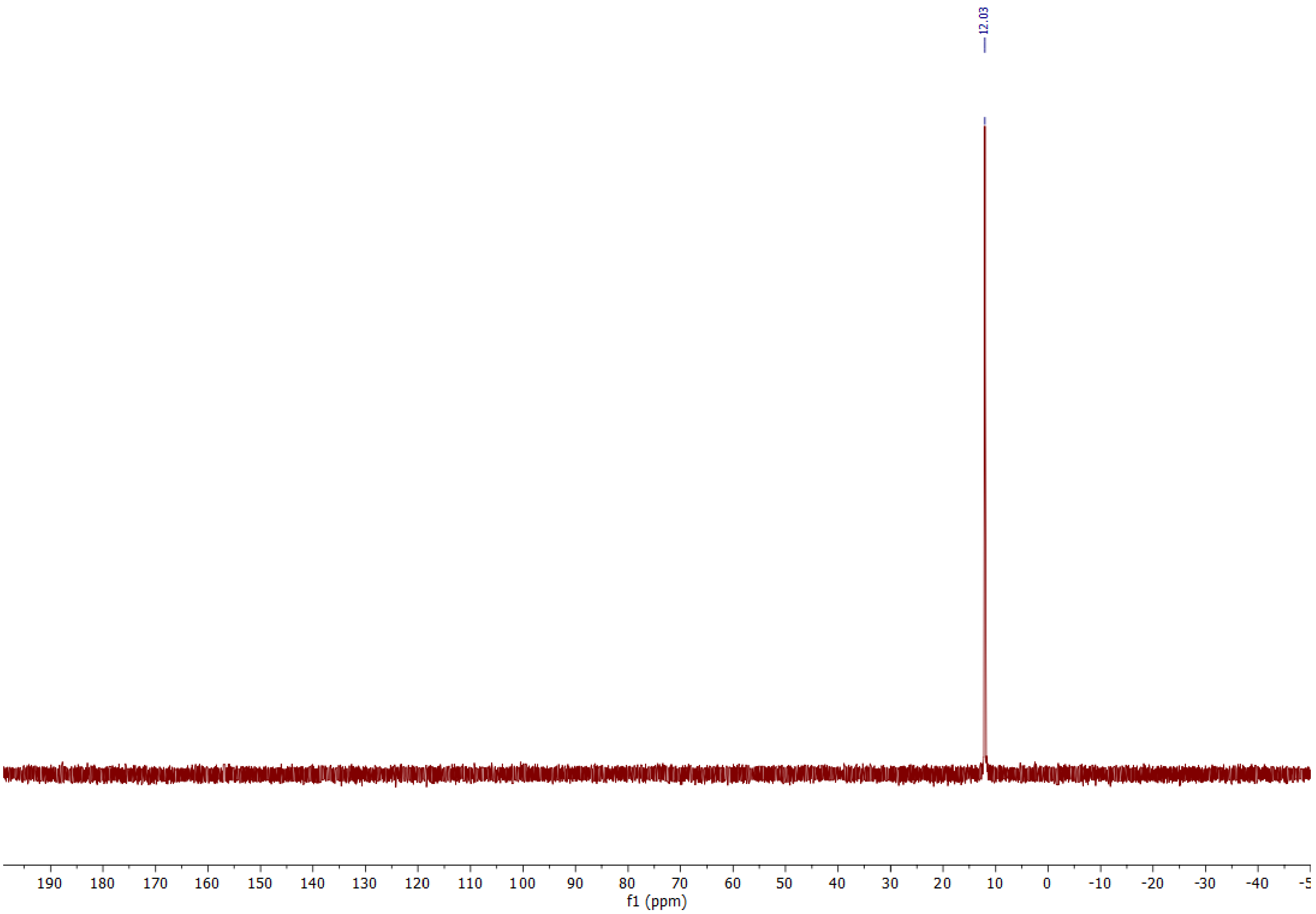

Figure S101. ^{31}P NMR spectrum of **5c** diastereomer 2.

(Piperidin-2-yl(4,5,6,7-tetrahydrobenzo[*b*]thiophene-2-carboxamido)methyl)phosphonic acid hydrochloride (**5d**)

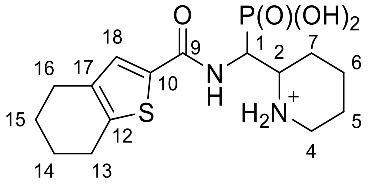

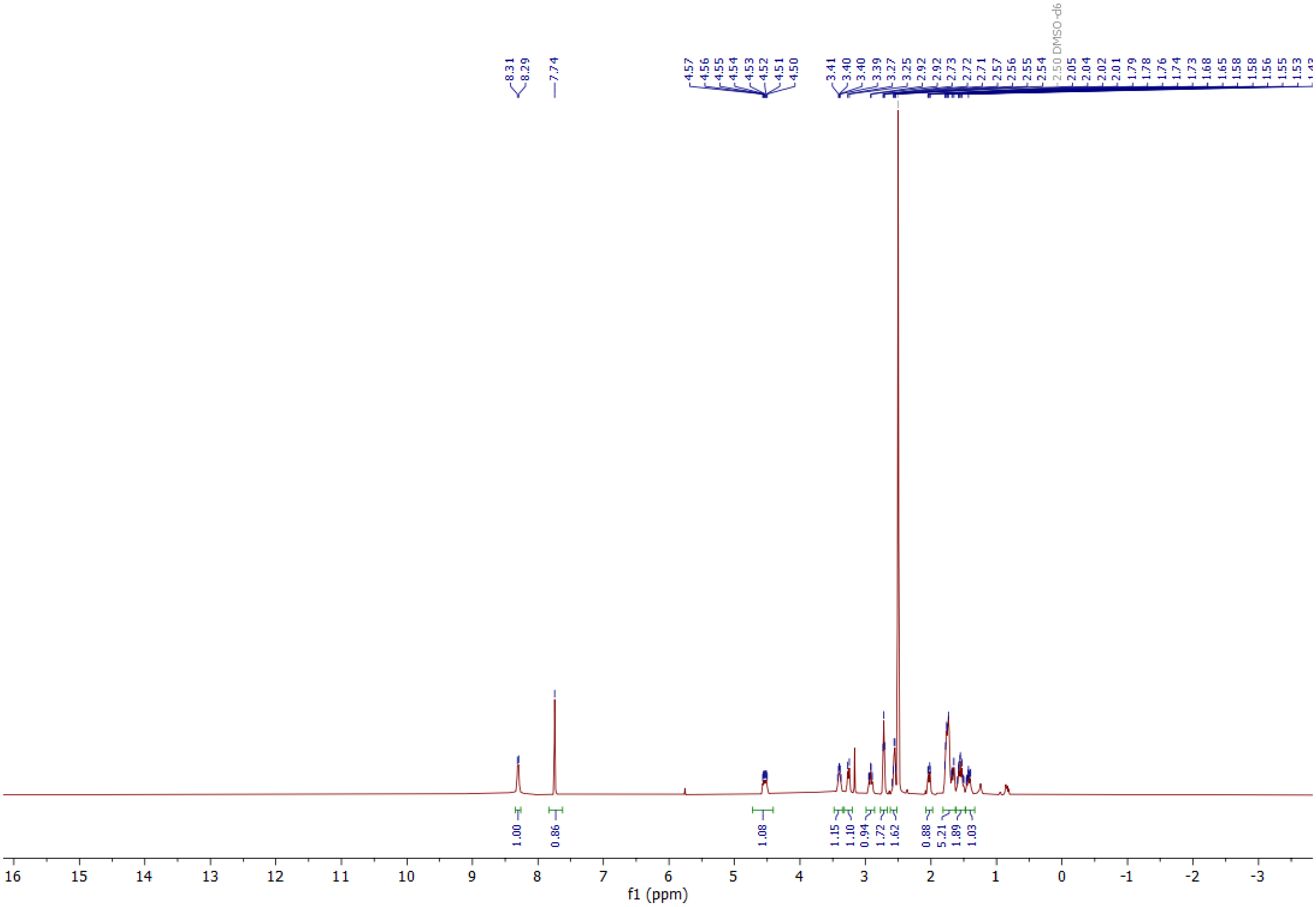

Figure S102. ^{1}H NMR spectrum of **5d** diastereomer 1.

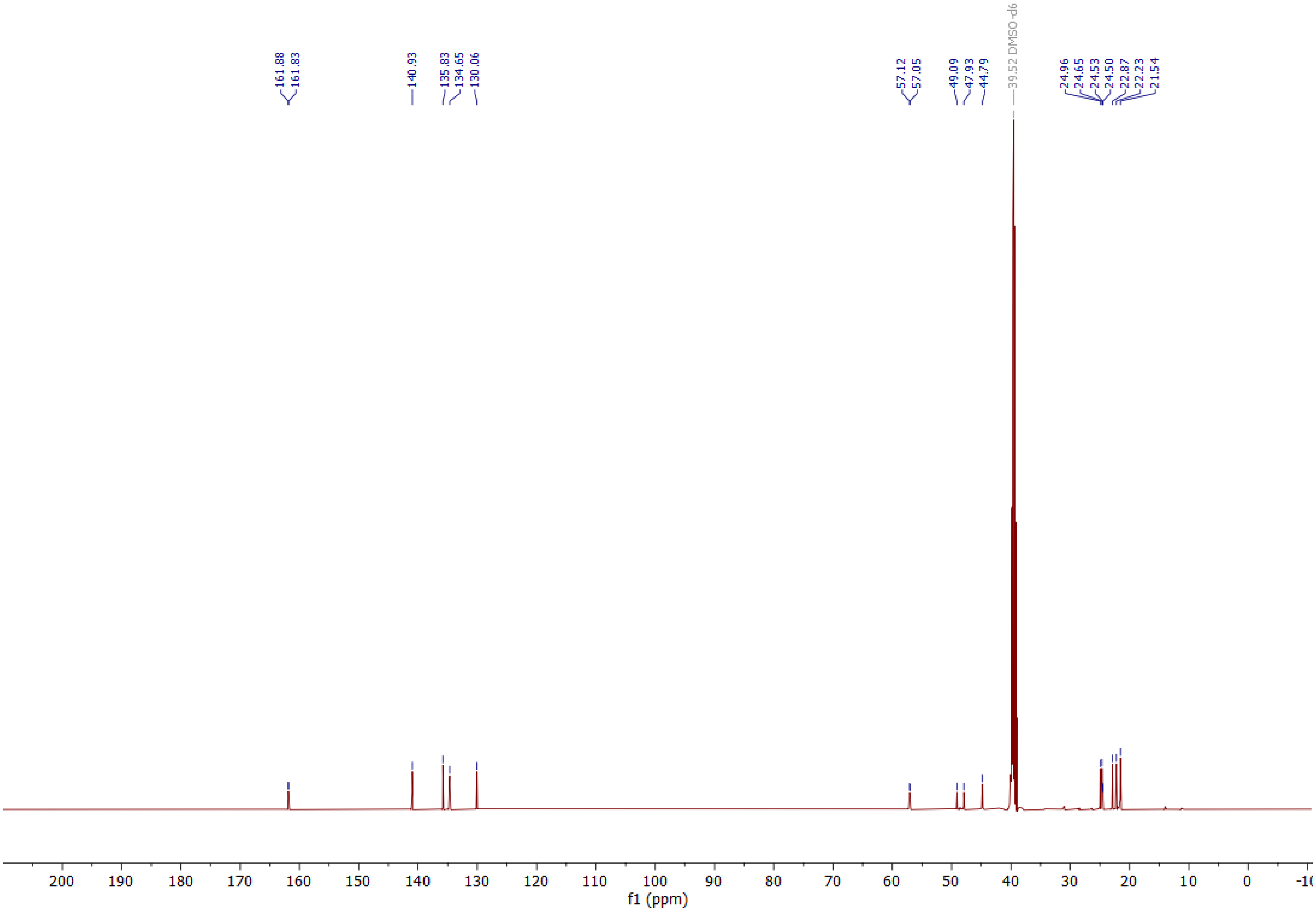

Figure S103. ^{13}C NMR spectrum of **5d** diastereomer 1.

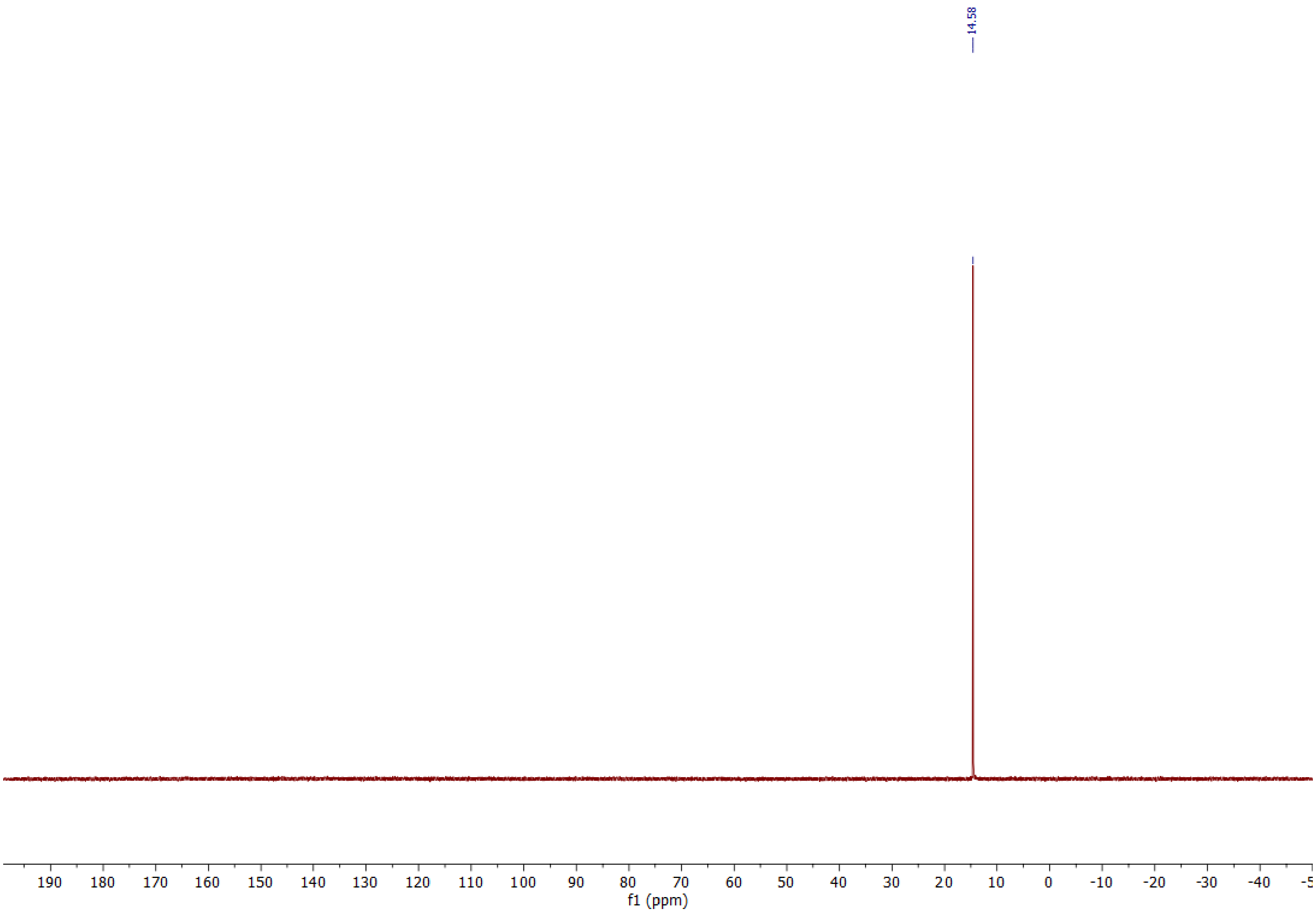

Figure S104. ^{31}P NMR spectrum of **5d** diastereomer 1.

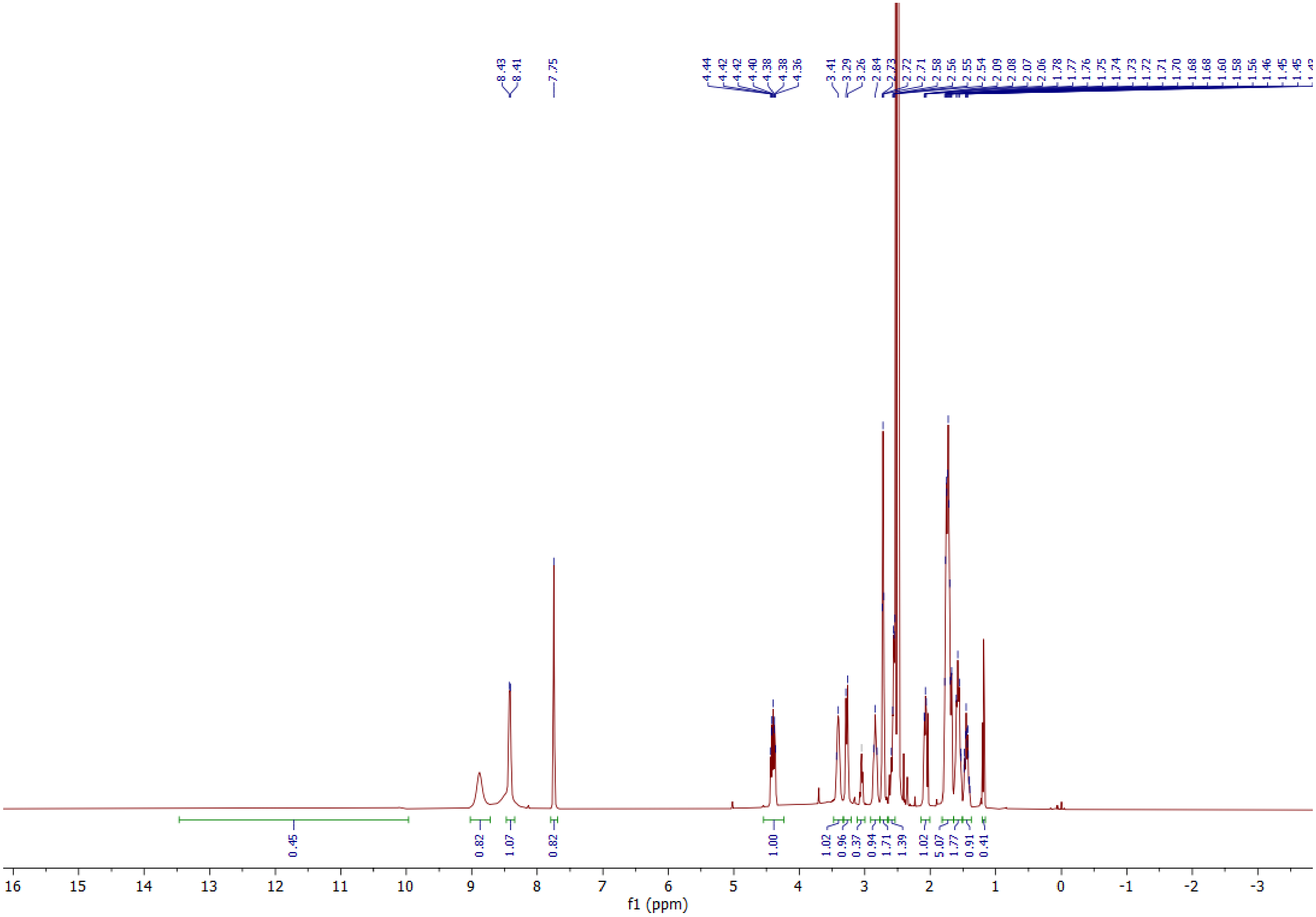

Figure S105. ^{1}H NMR spectrum of **5d** diastereomer 2.

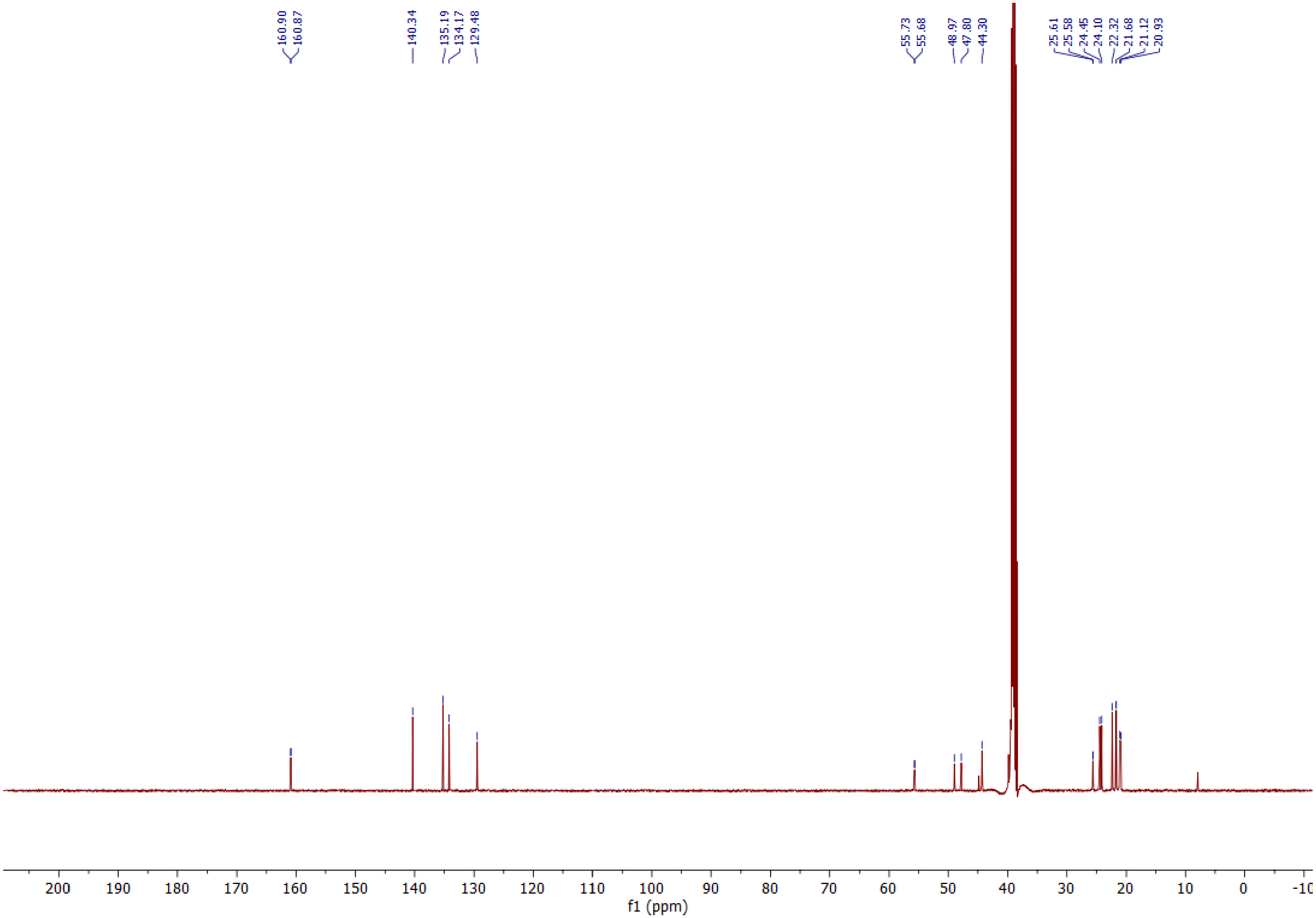

Figure S106. ^{13}C NMR spectrum of **5d** diastereomer 2.

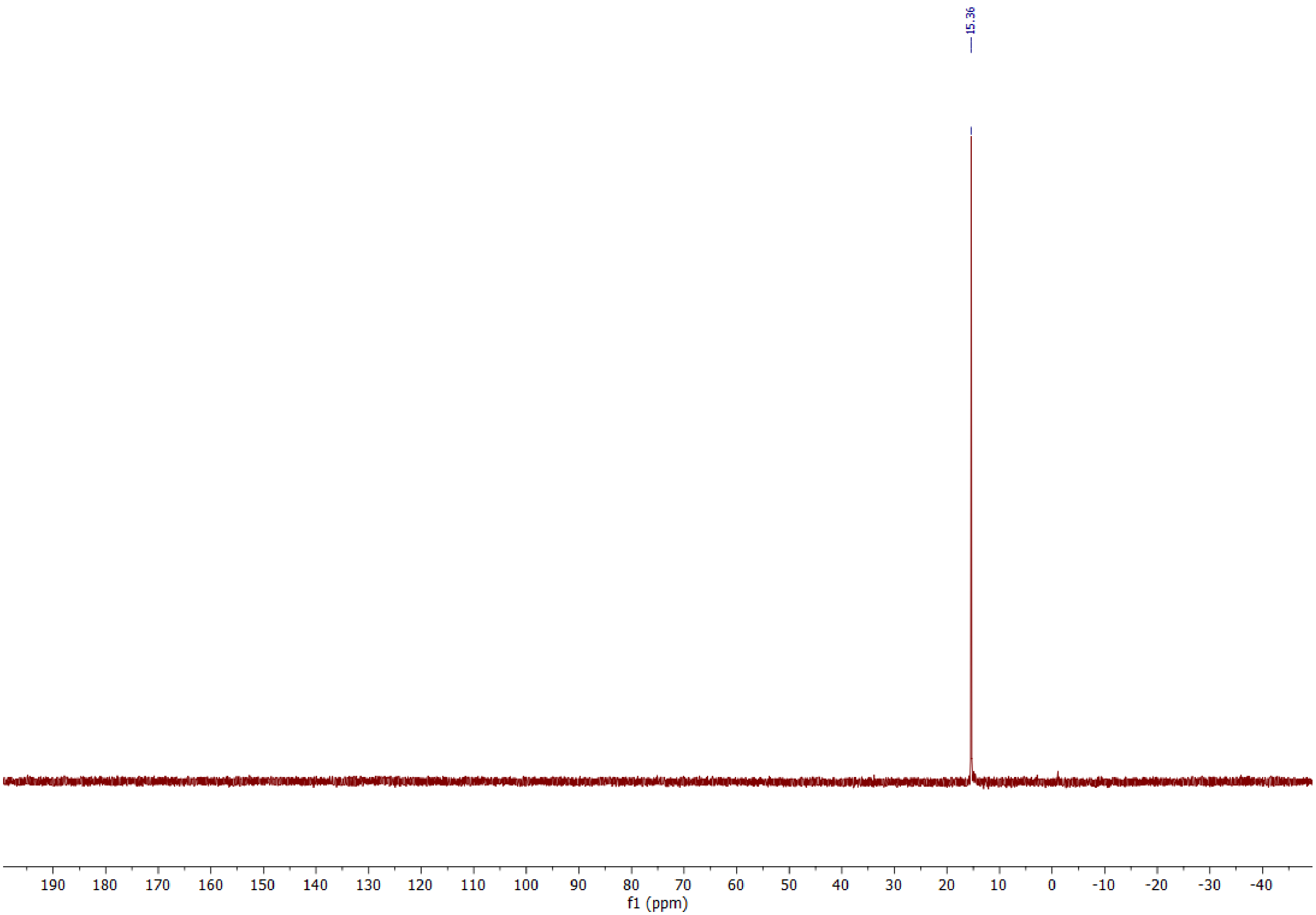

Figure S107. ^{31}P NMR spectrum of **5d** diastereomer 2.

(Piperidin-2-yl(4,5,6,7-tetrahydrobenzo[*b*]thiophene-3-carboxamido)methyl)phosphonic acid hydrochloride (**5e**)

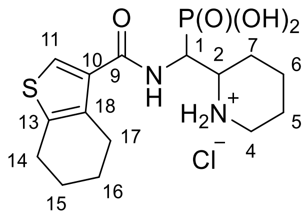

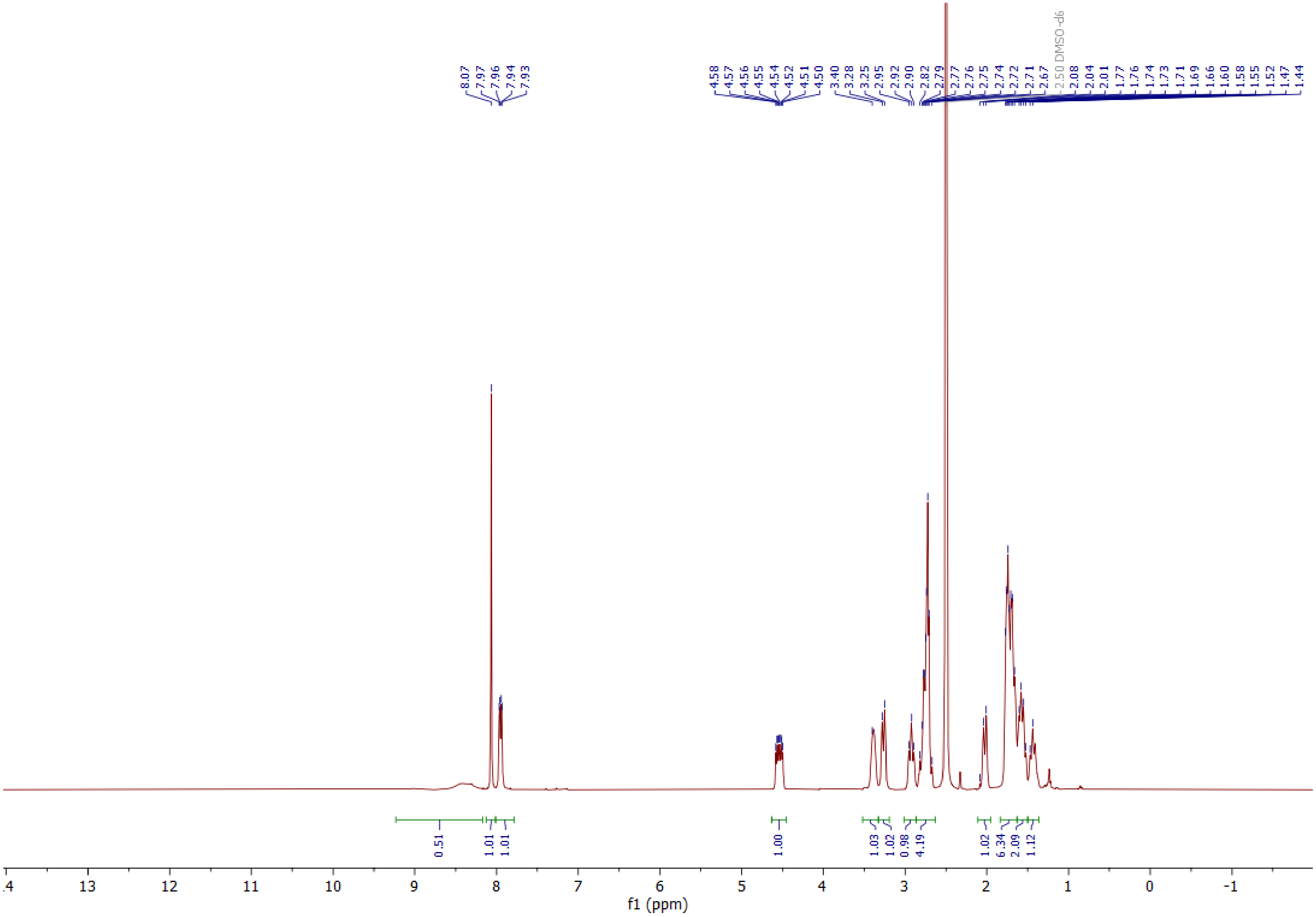

Figure S108. ^{1}H NMR spectrum of **5e** diastereomer 1.

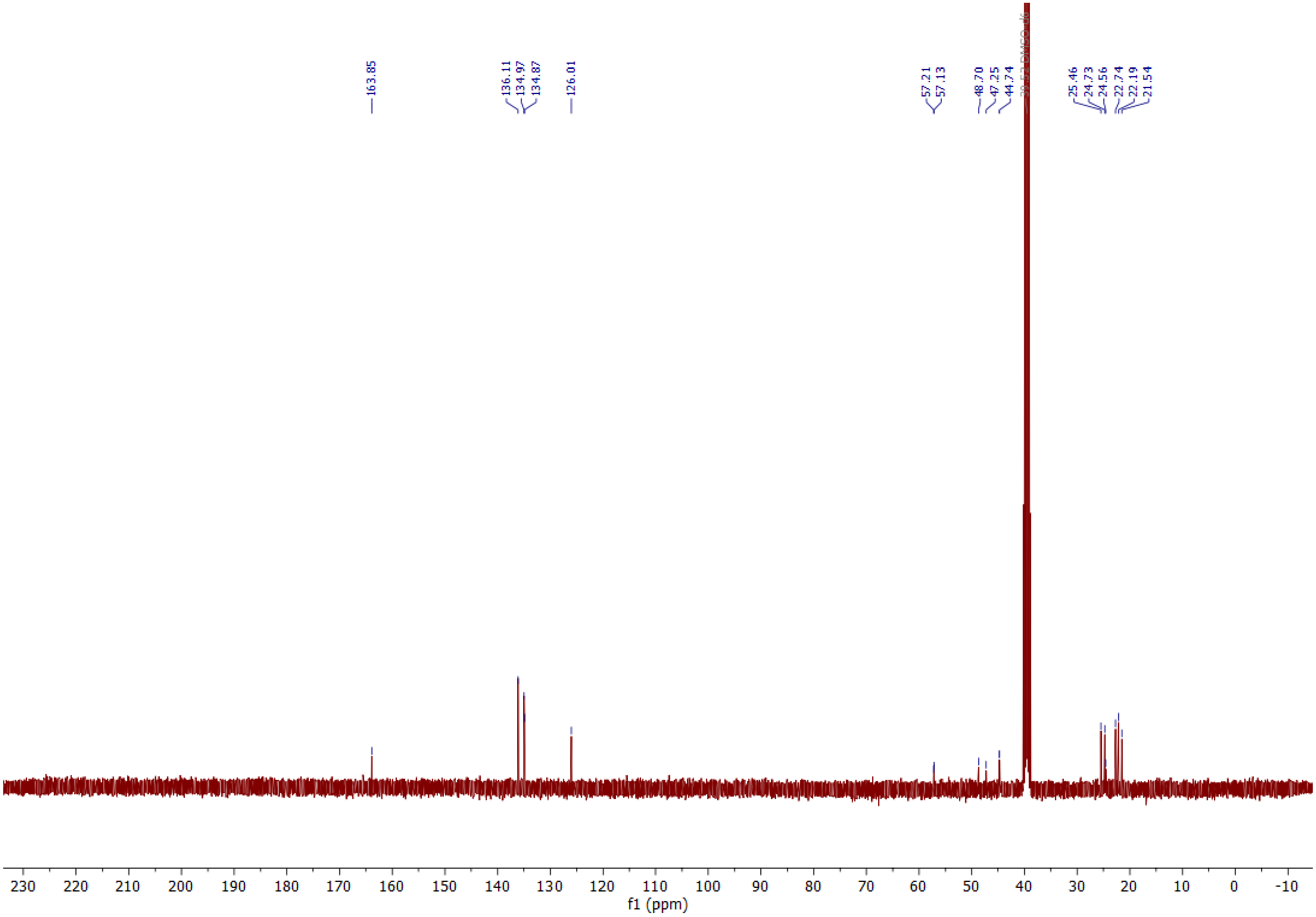

Figure S109. ^{13}C NMR spectrum of **5e** diastereomer 1.

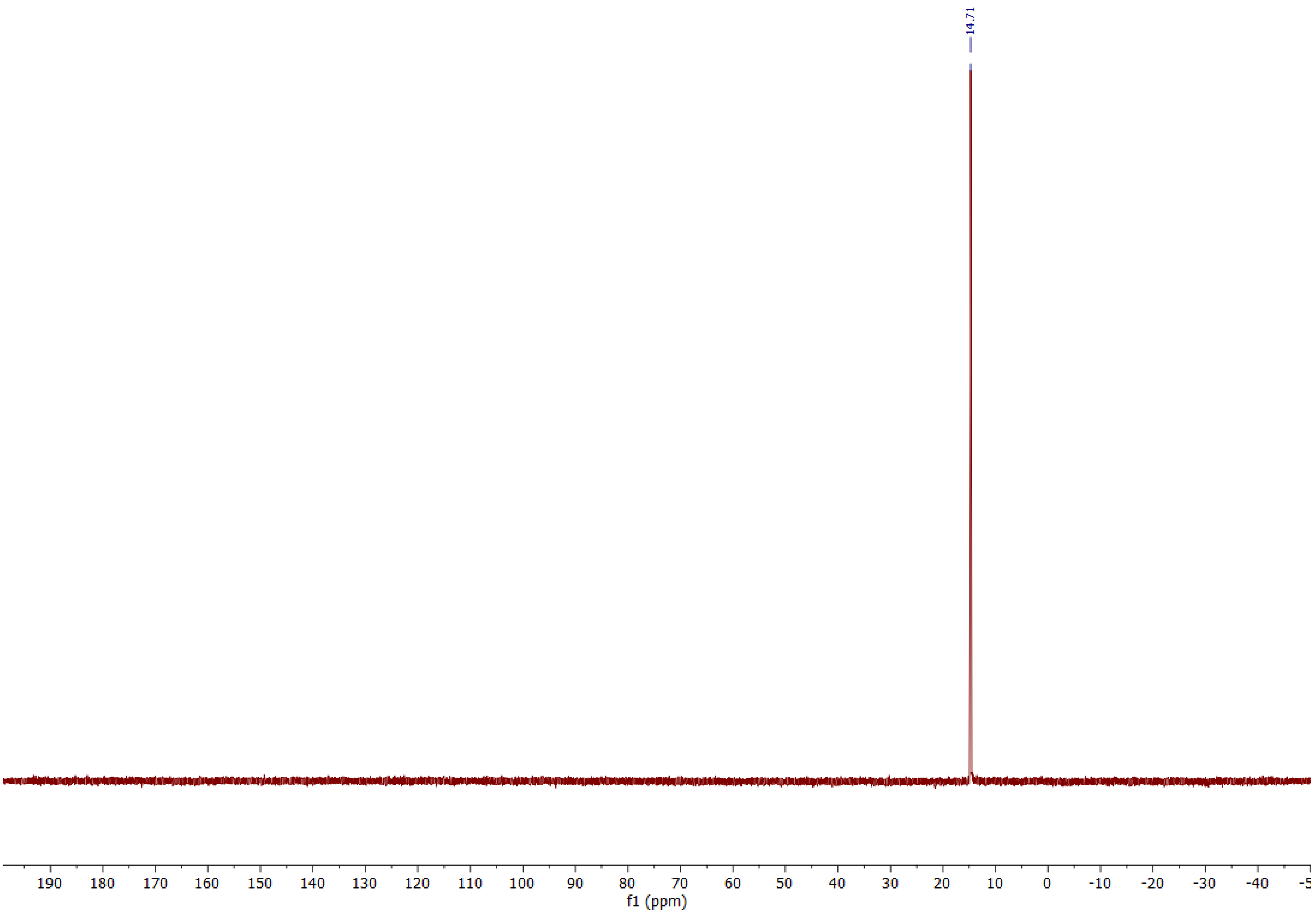

Figure S110. ^{31}P NMR spectrum of **5e** diastereomer 1.

(Piperidin-2-yl(4,5,6,7-tetrahydrobenzo[*c*]thiophene-1-carboxamido)methyl)phosphonic acid hydrochloride (**5f**)

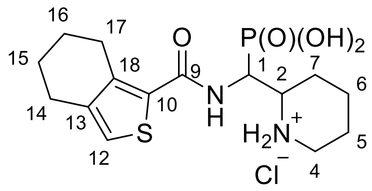

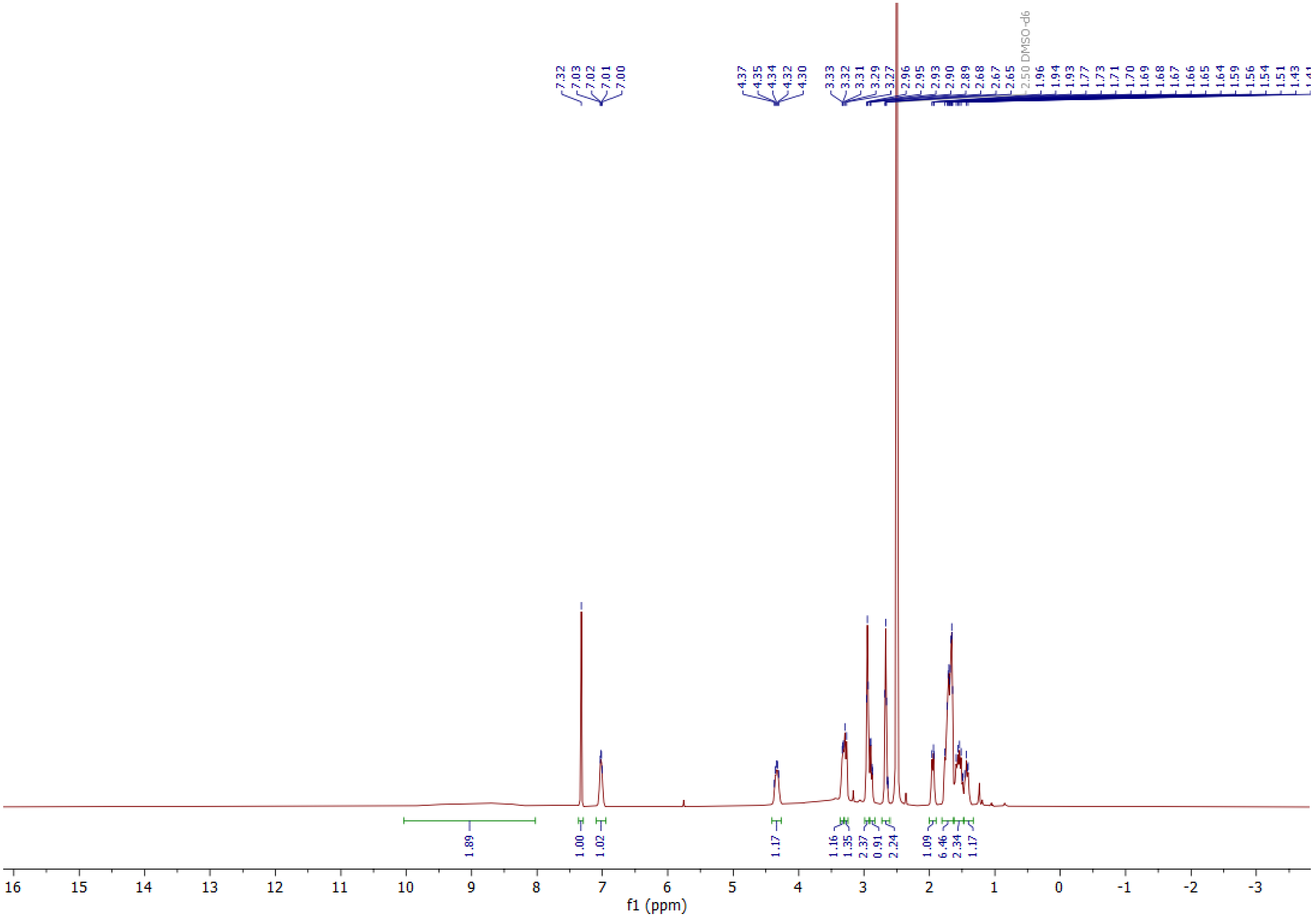

Figure S111. ^{1}H NMR spectrum of **5f** diastereomer 1.

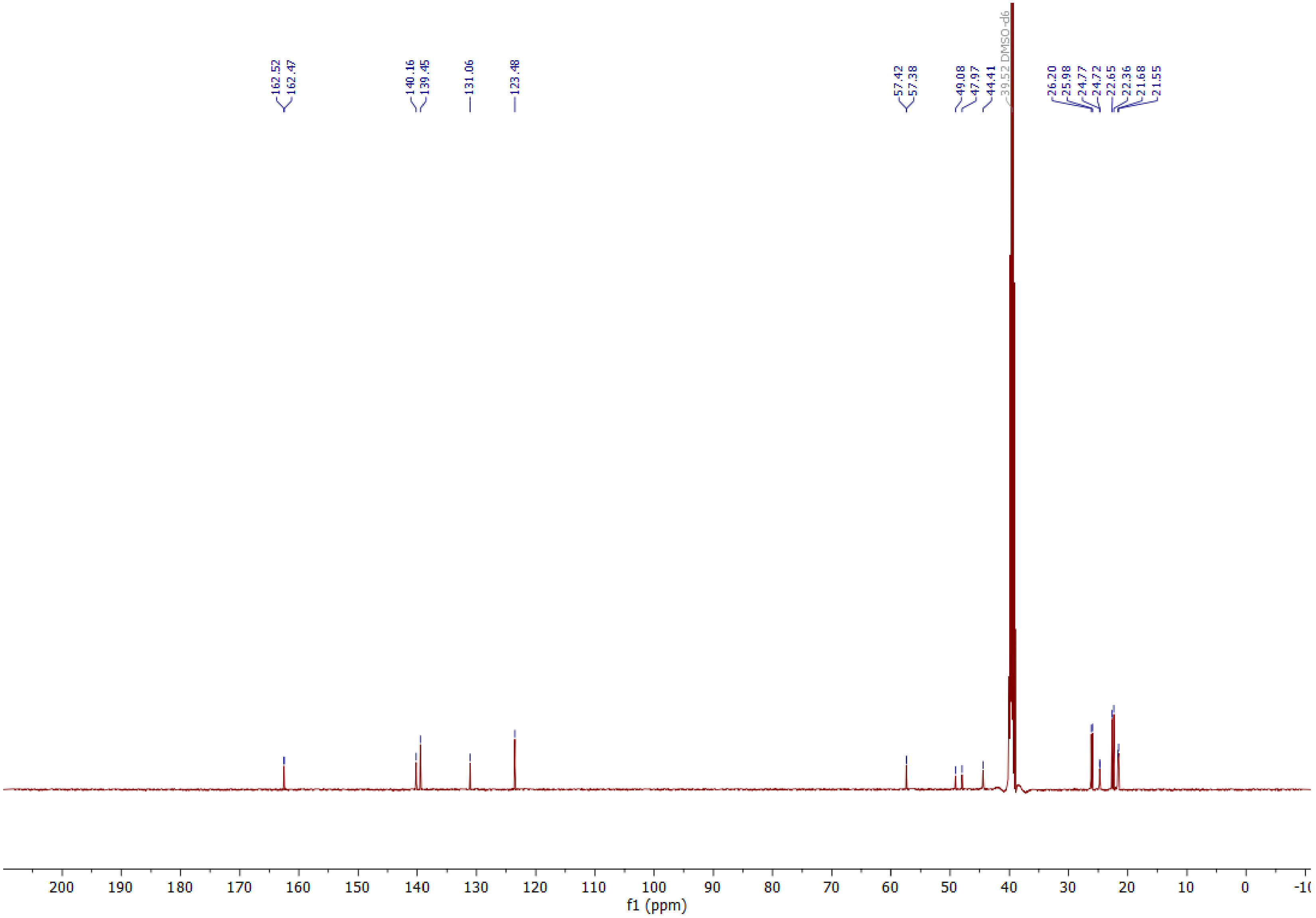

Figure S112. ^{13}C NMR spectrum of **5f** diastereomer 1.

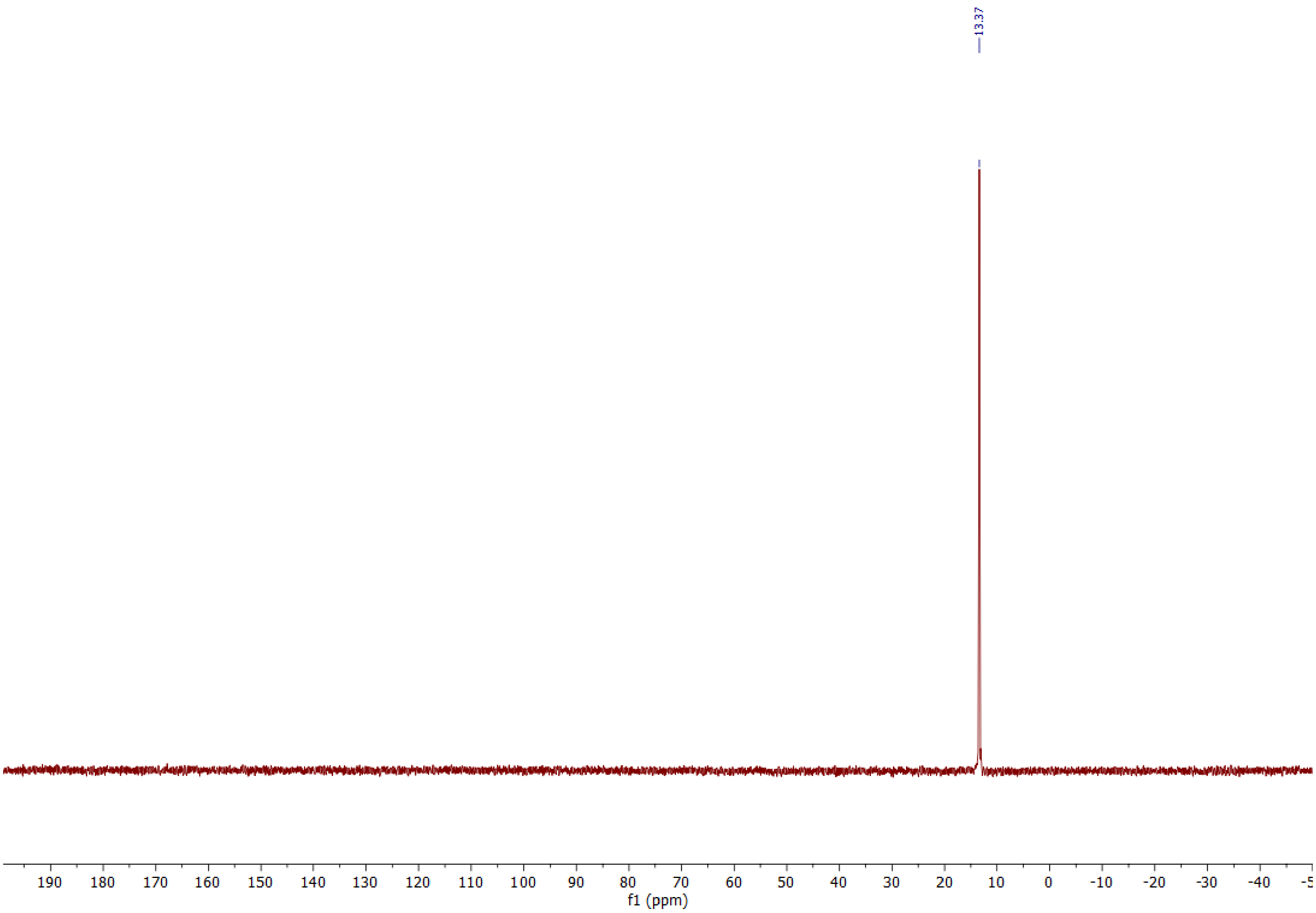

Figure S113. ^{31}P NMR spectrum of **5f** diastereomer 1.

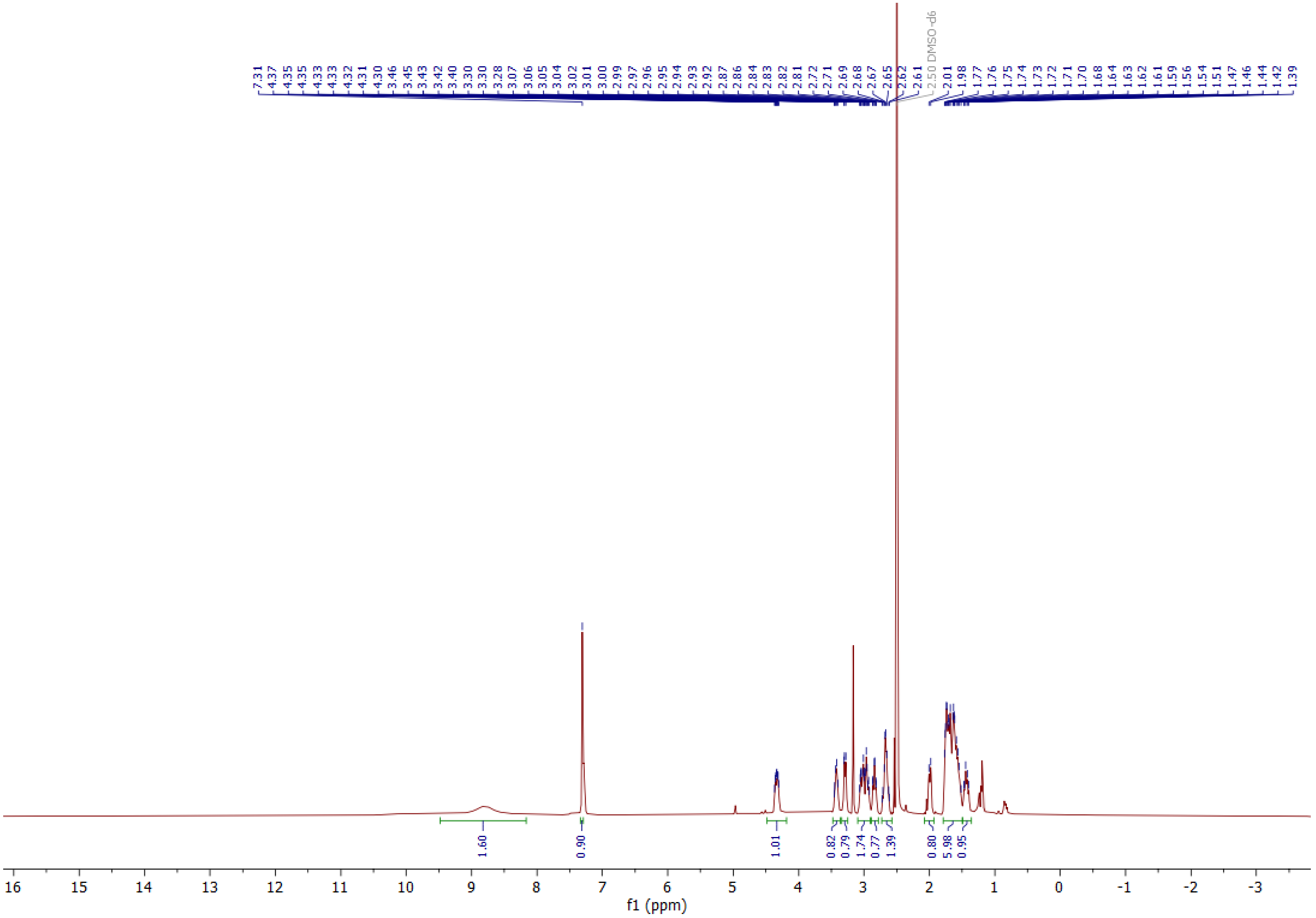

Figure S114. ^{1}H NMR spectrum of **5f** diastereomer 2.

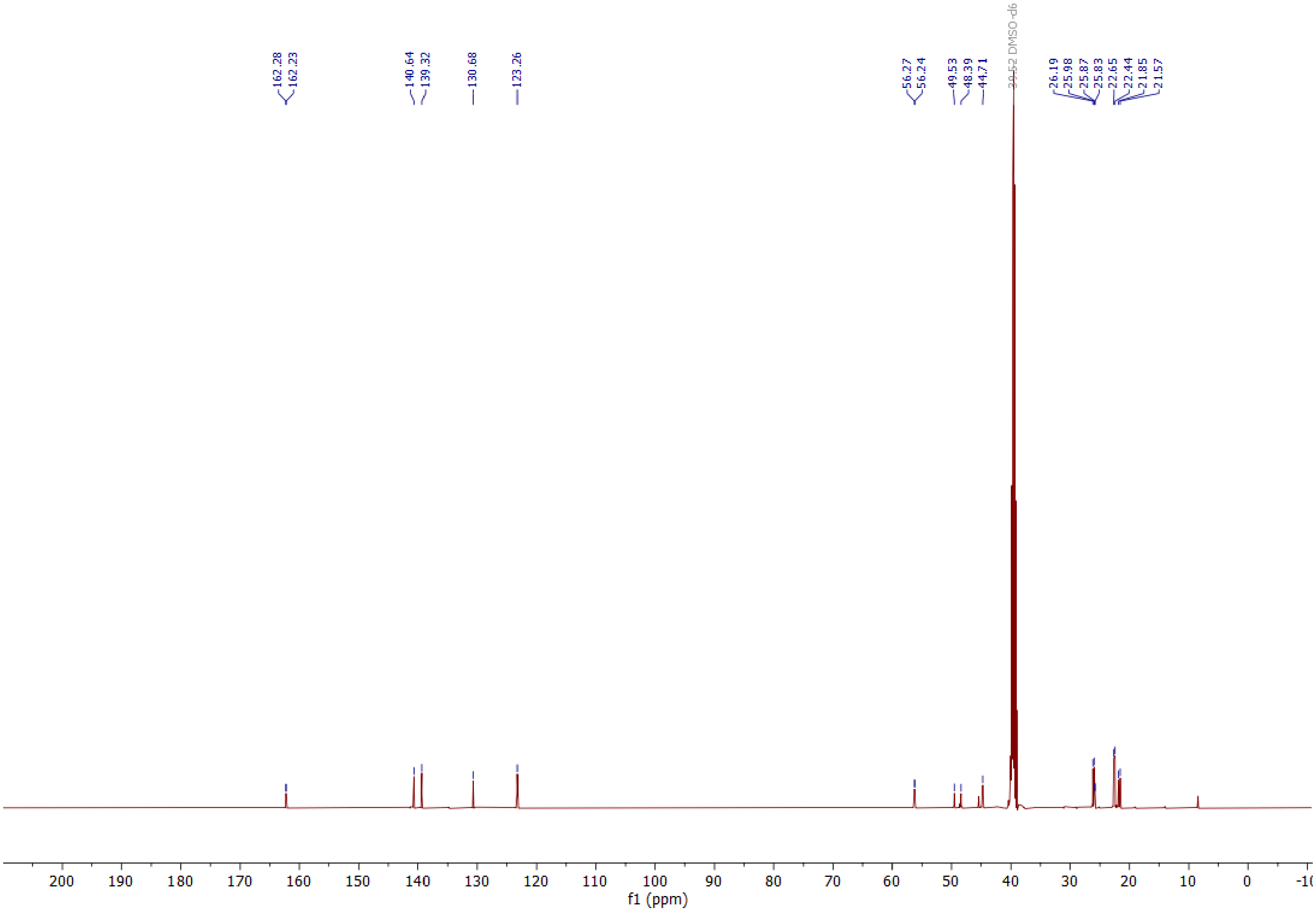

Figure S115. ^{13}C NMR spectrum of **5f** diastereomer 2.

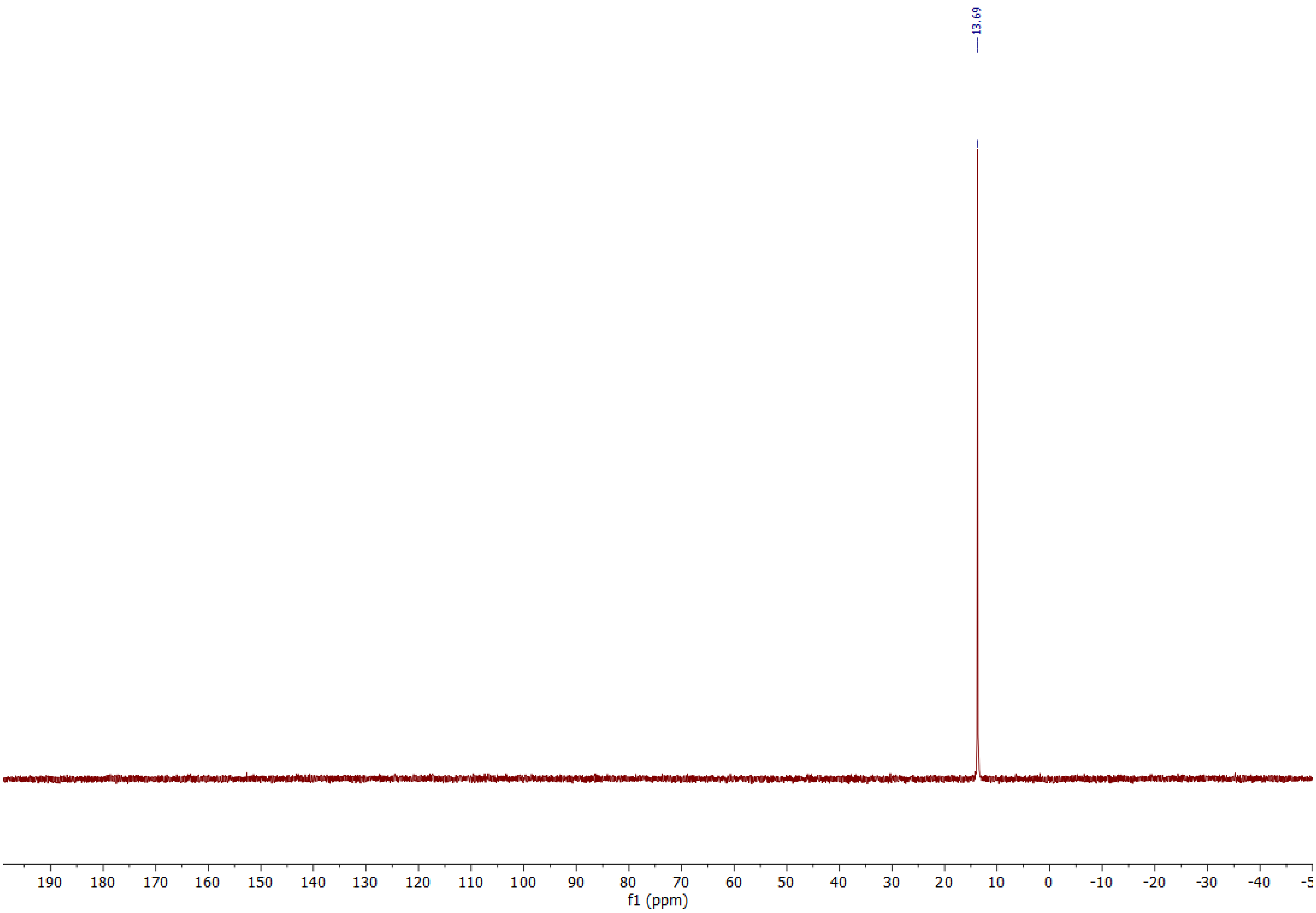

Figure S116. ^{31}P NMR spectrum of **5f** diastereomer 2.

((Benzo[*b*]thiophene-2-carboxamido)(piperidin-2-yl)methyl)phosphonic acid hydrochloride (**5g**)

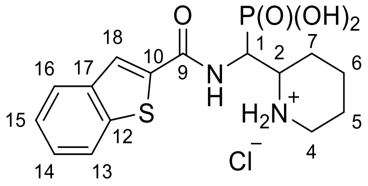

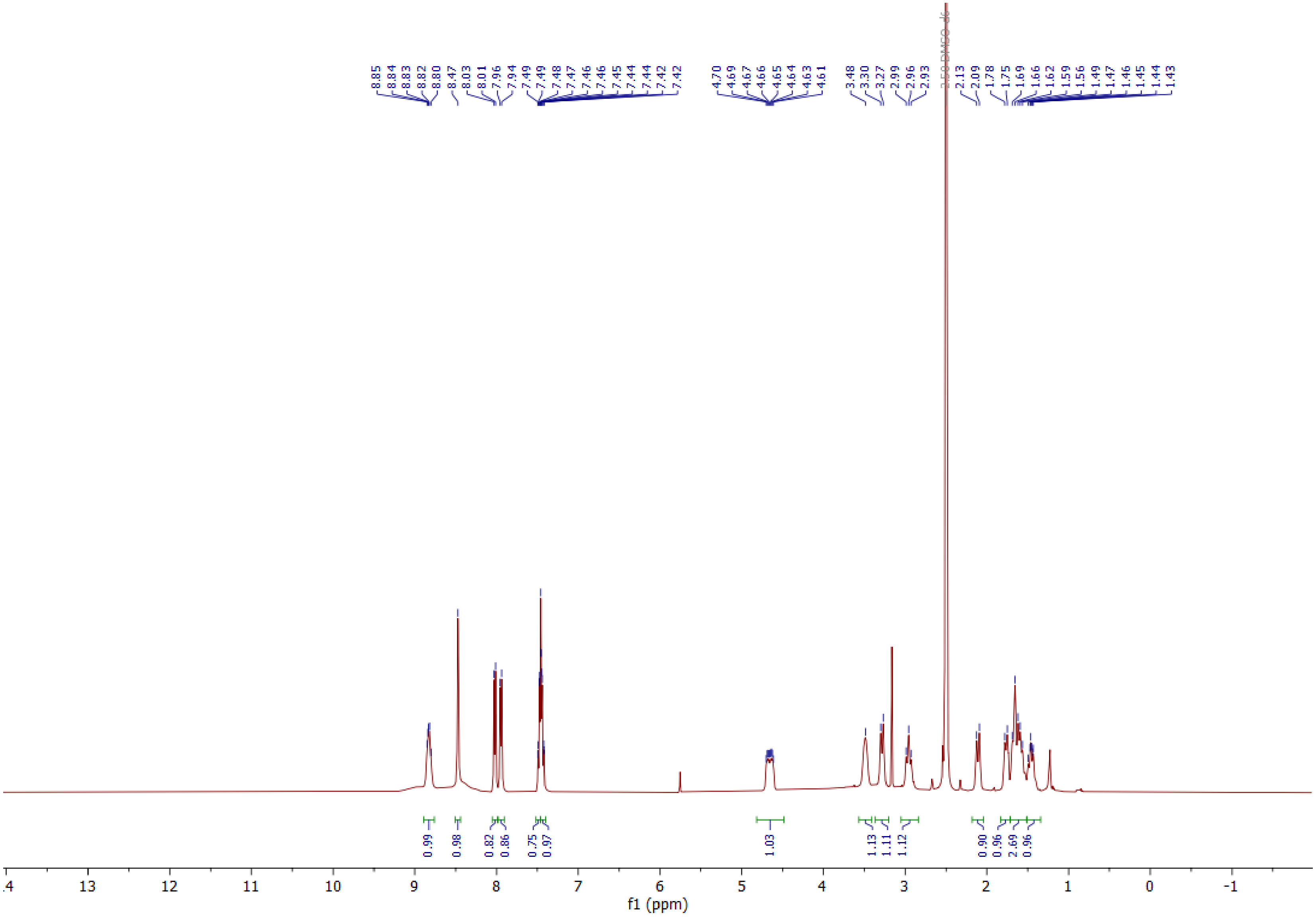

Figure S117. ^{1}H NMR spectrum of **5g** diastereomer 1.

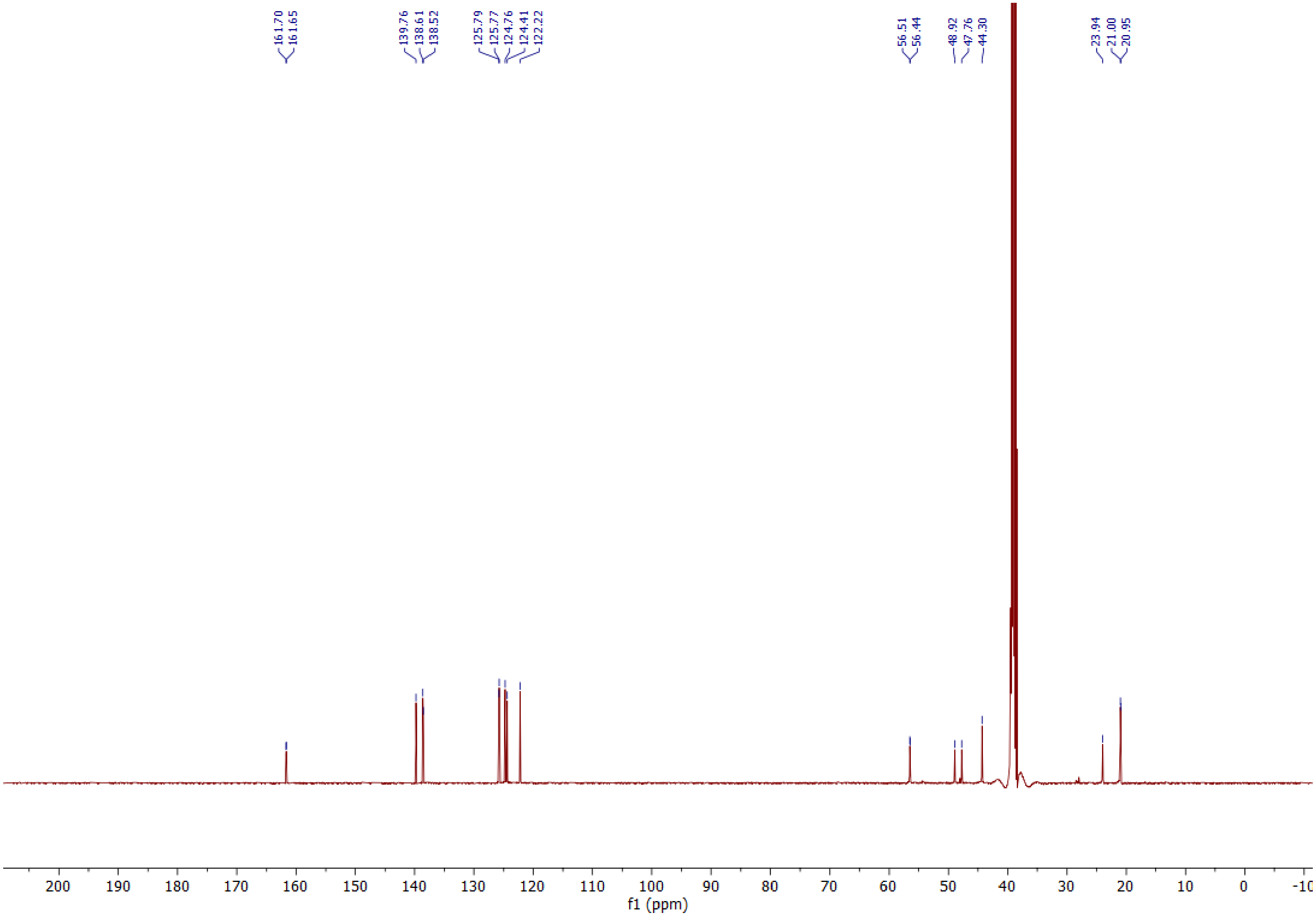

Figure S118. ^{13}C NMR spectrum of **5g** diastereomer 1.

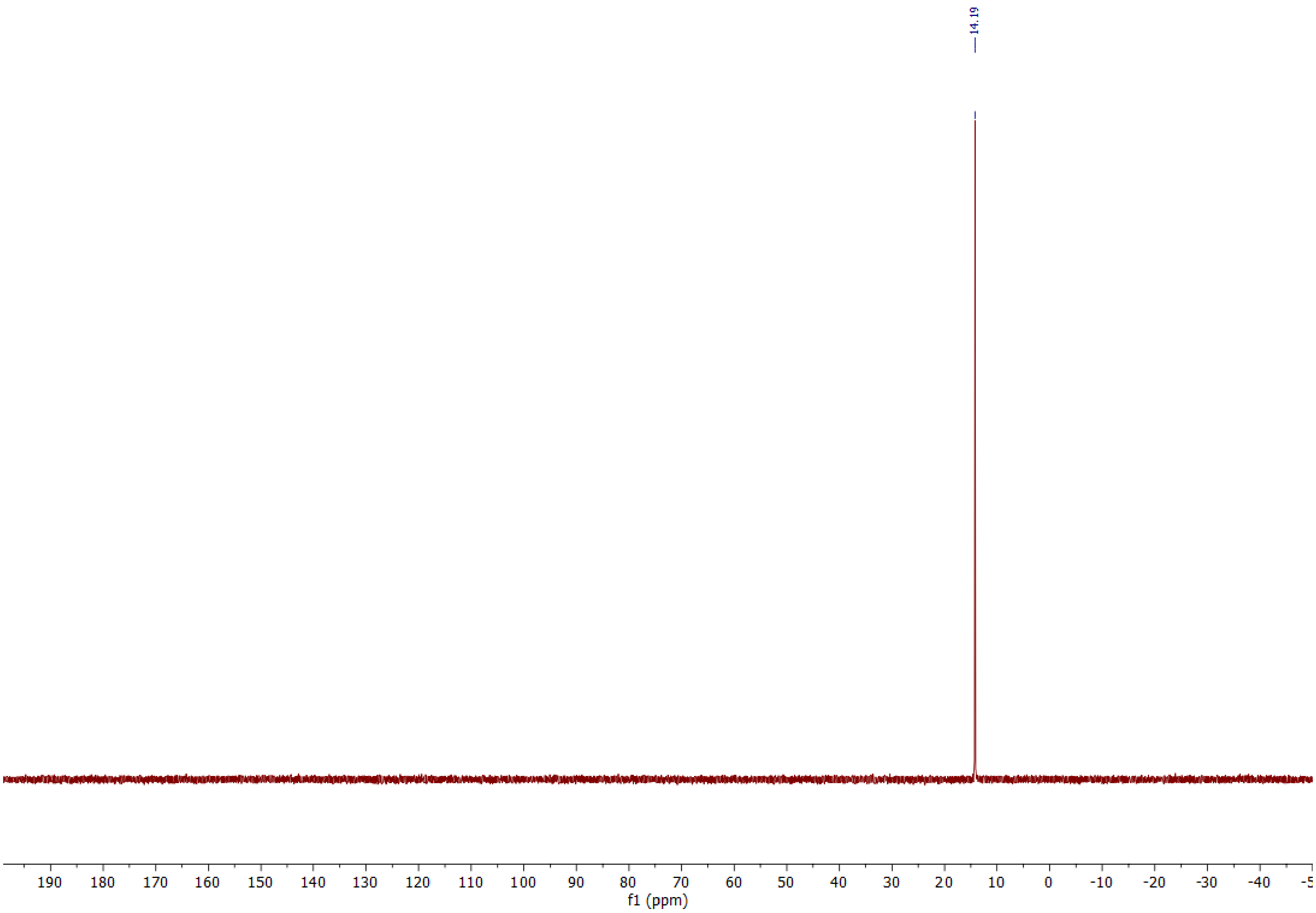

Figure S119. ^{31}P NMR spectrum of **5g** diastereomer 1.

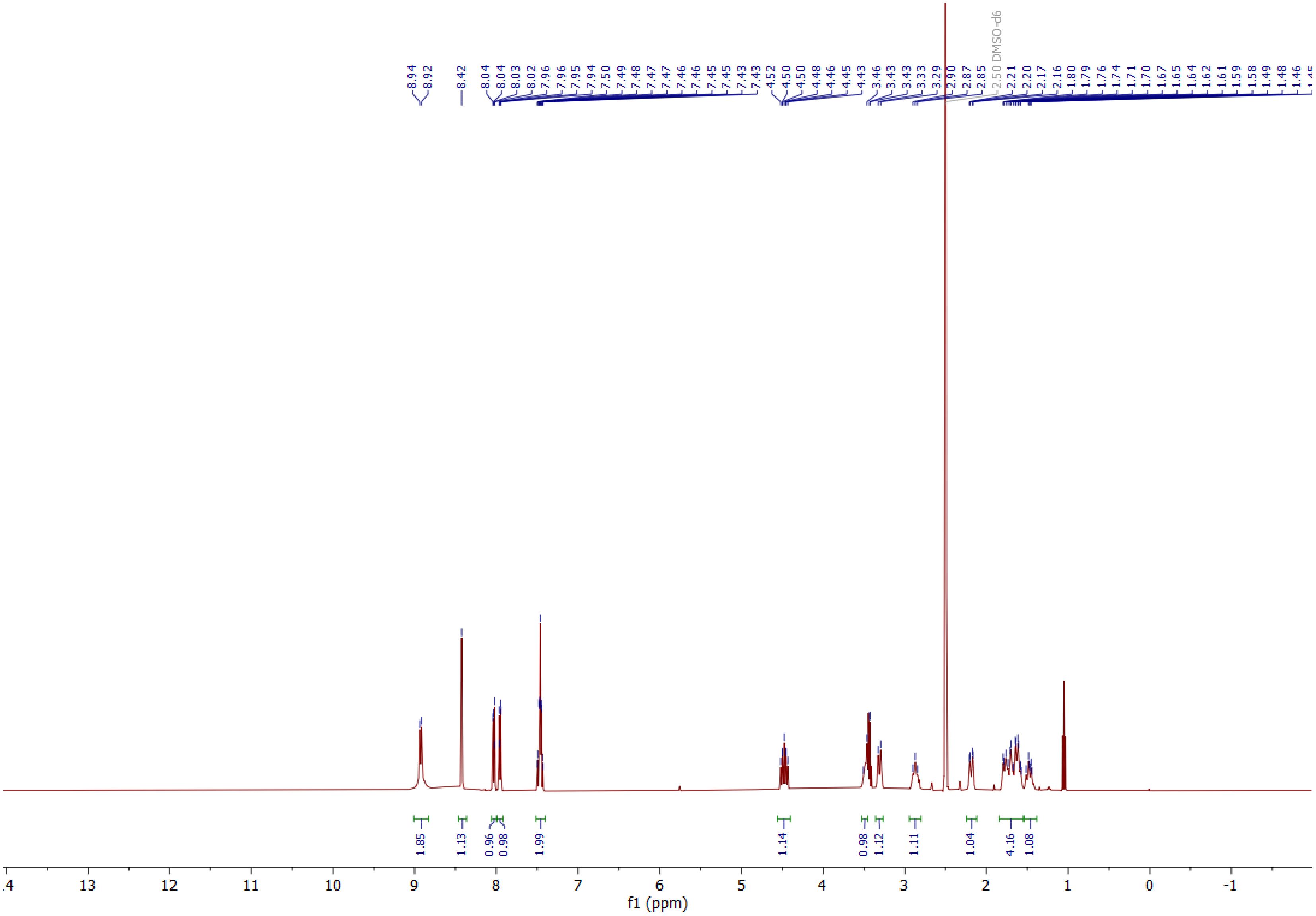

Figure S120. ^{1}H NMR spectrum of **5g** diastereomer 2.

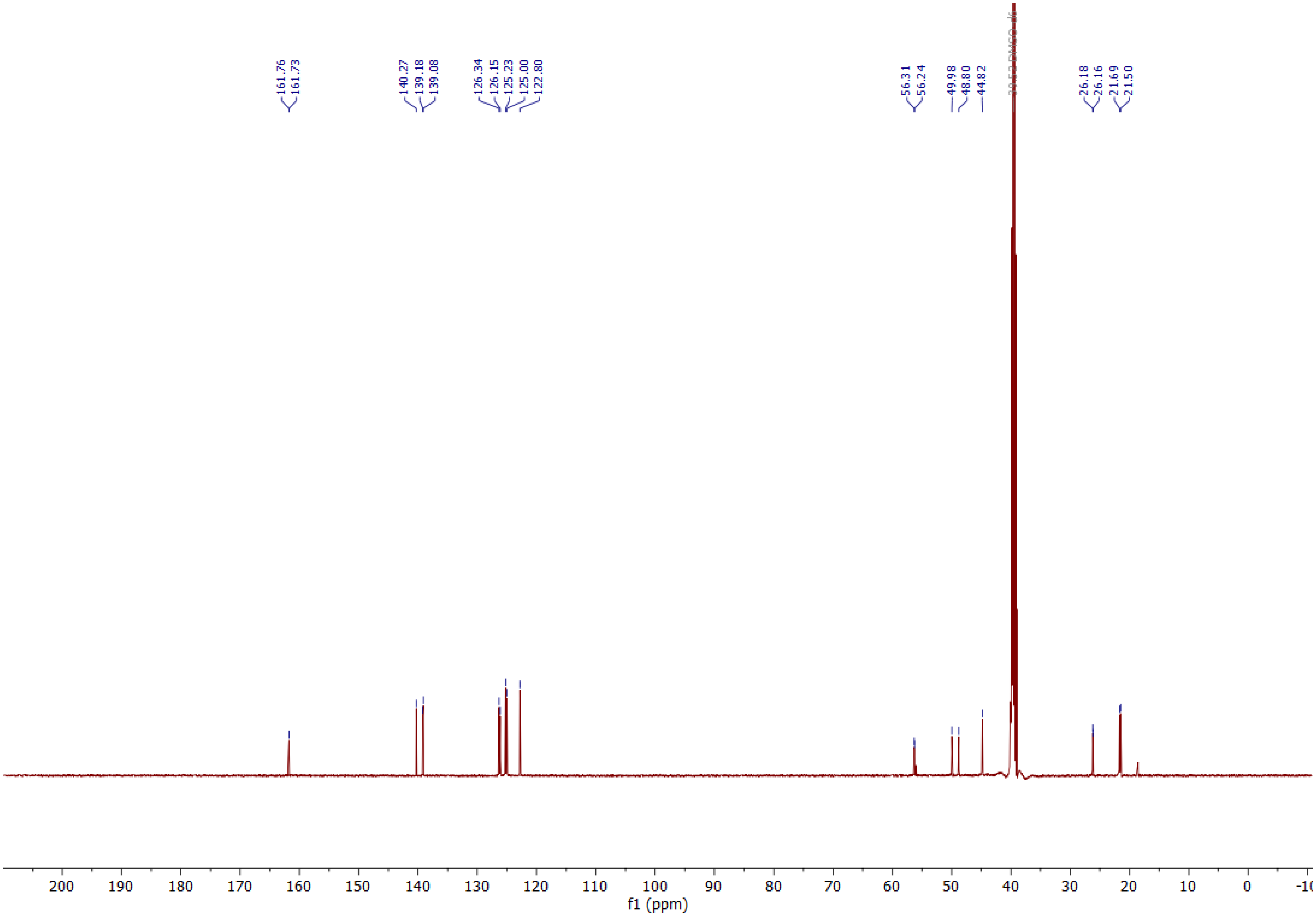

Figure S121. ^{13}C NMR spectrum of **5g** diastereomer 2.

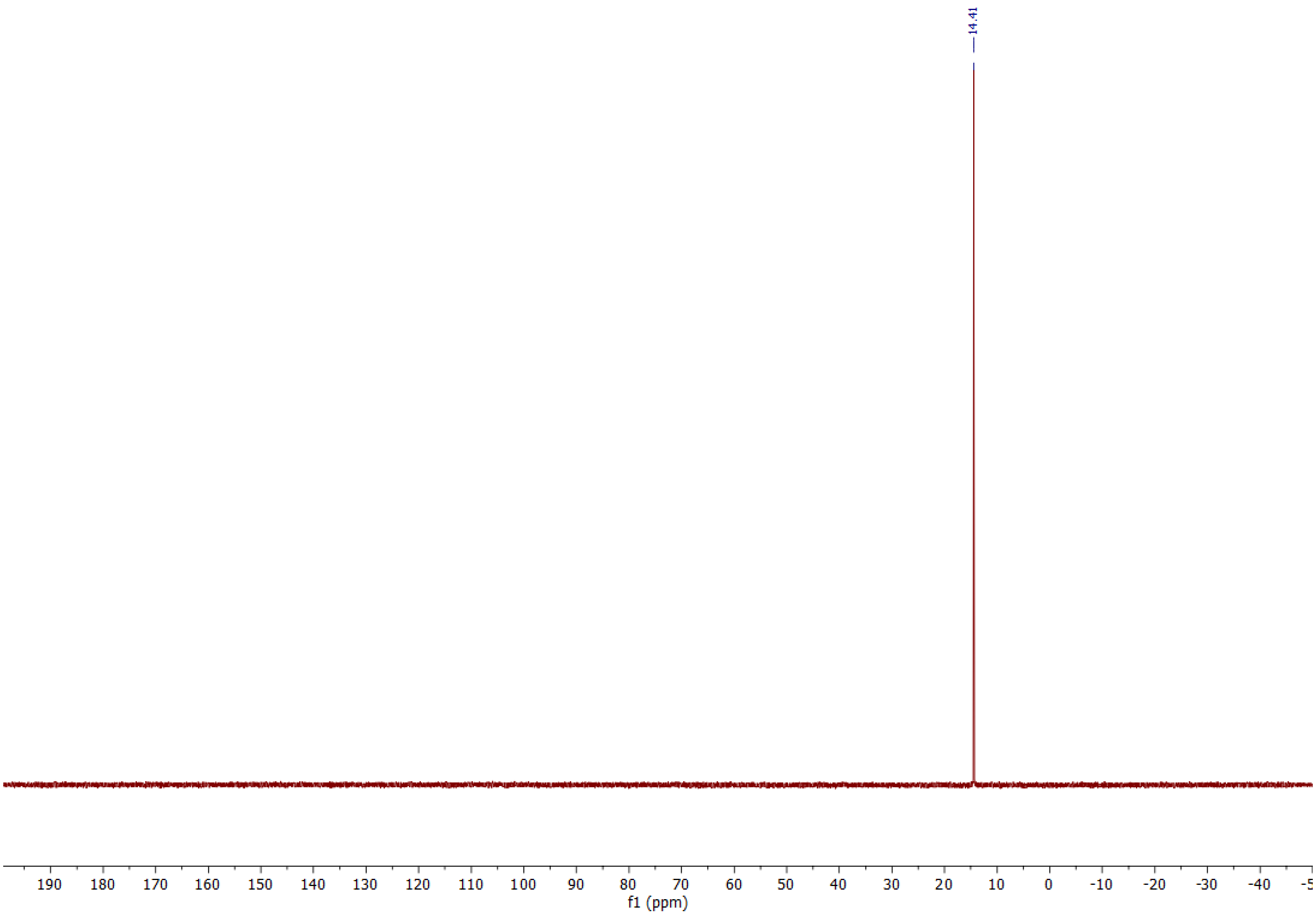

Figure S122. ^{31}P NMR spectrum of **5g** diastereomer 2.

((Benzo[*b*]thiophene-3-carboxamido)(piperidin-2-yl)methyl)phosphonic acid hydrochloride (**5h**)

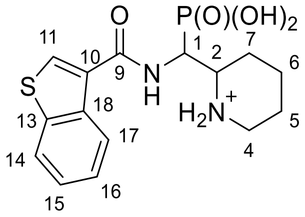

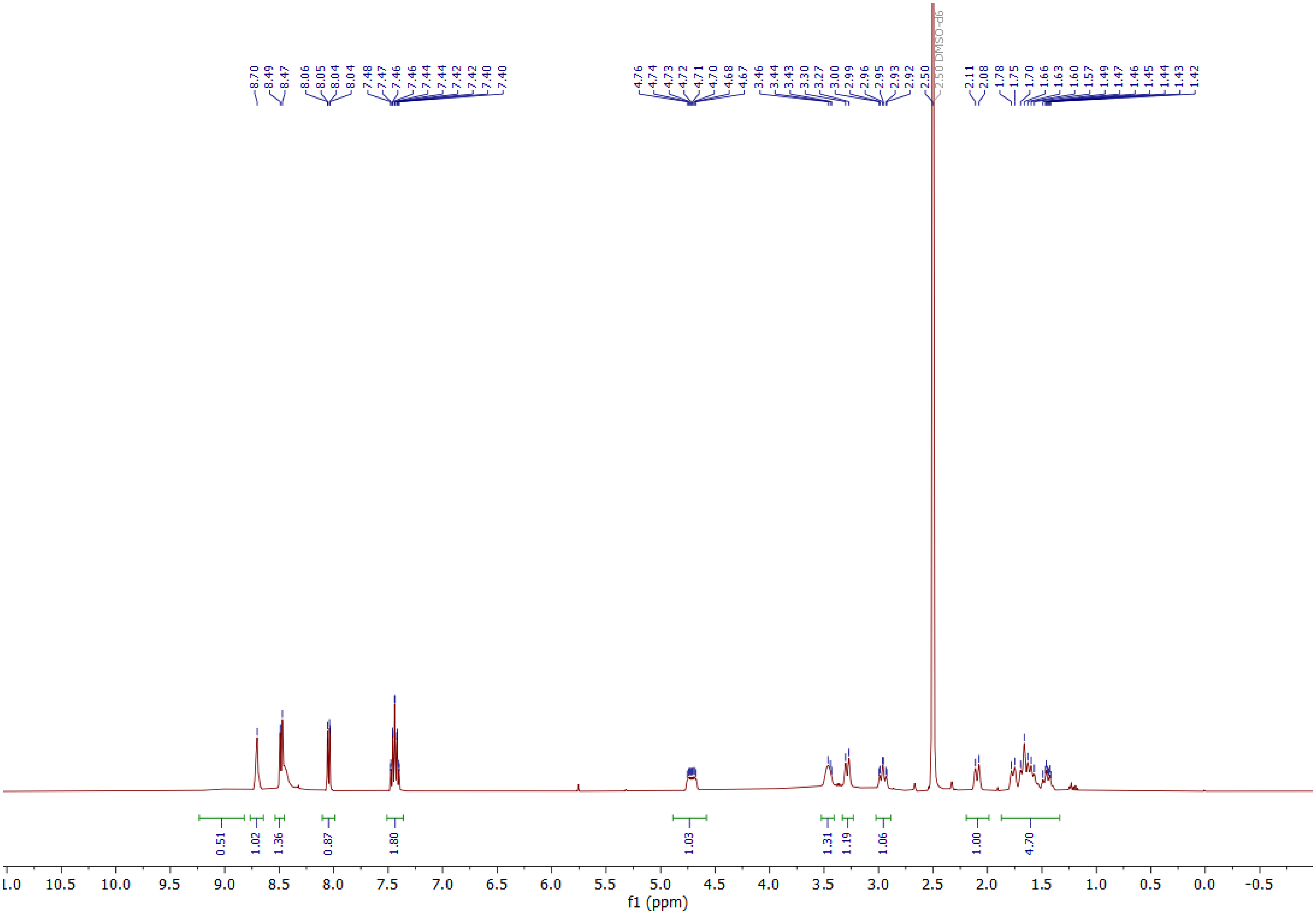

Figure S123. ^{1}H NMR spectrum of **5h** diastereomer 1.

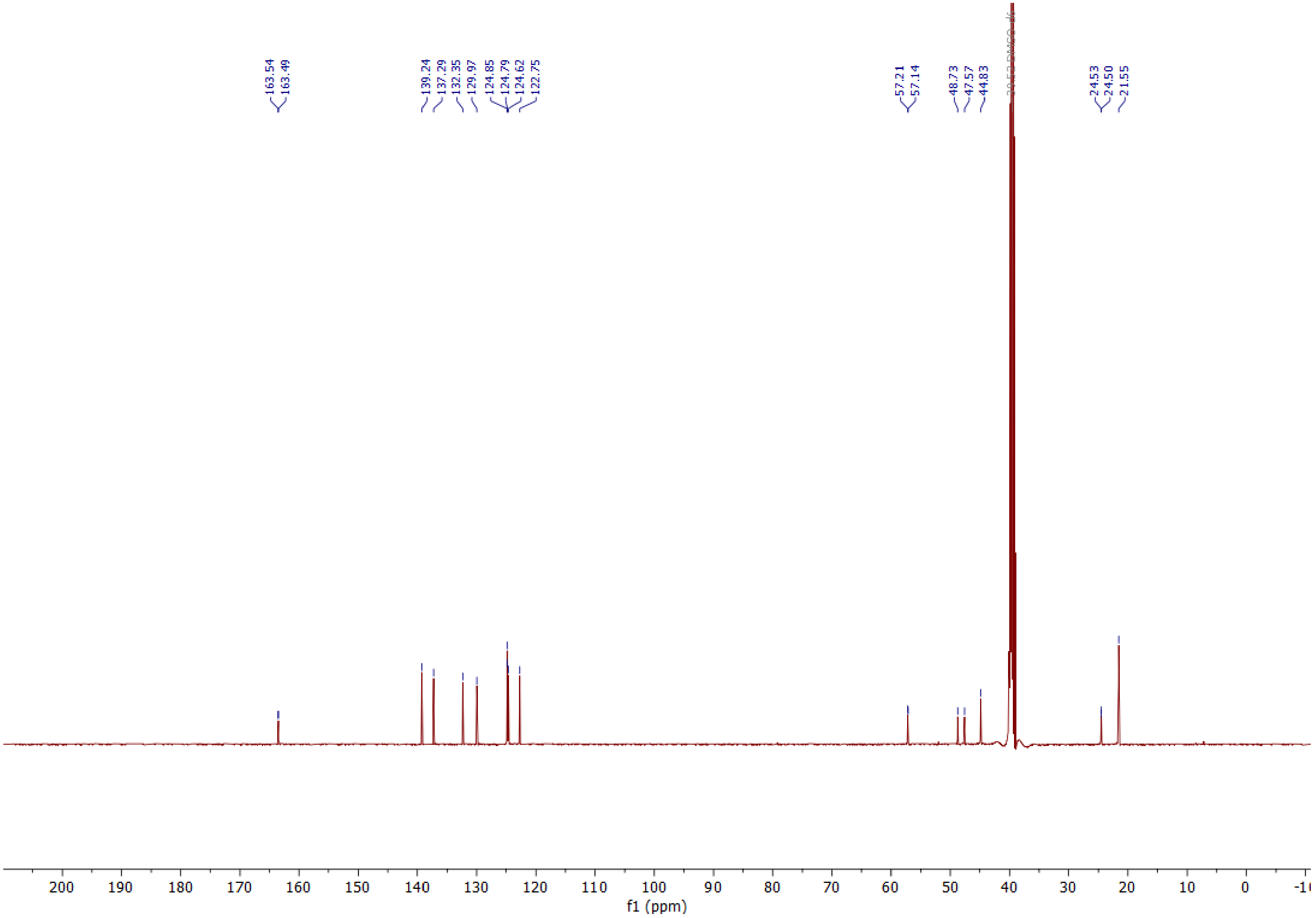

Figure S124. ^{13}C NMR spectrum of **5h** diastereomer 1.

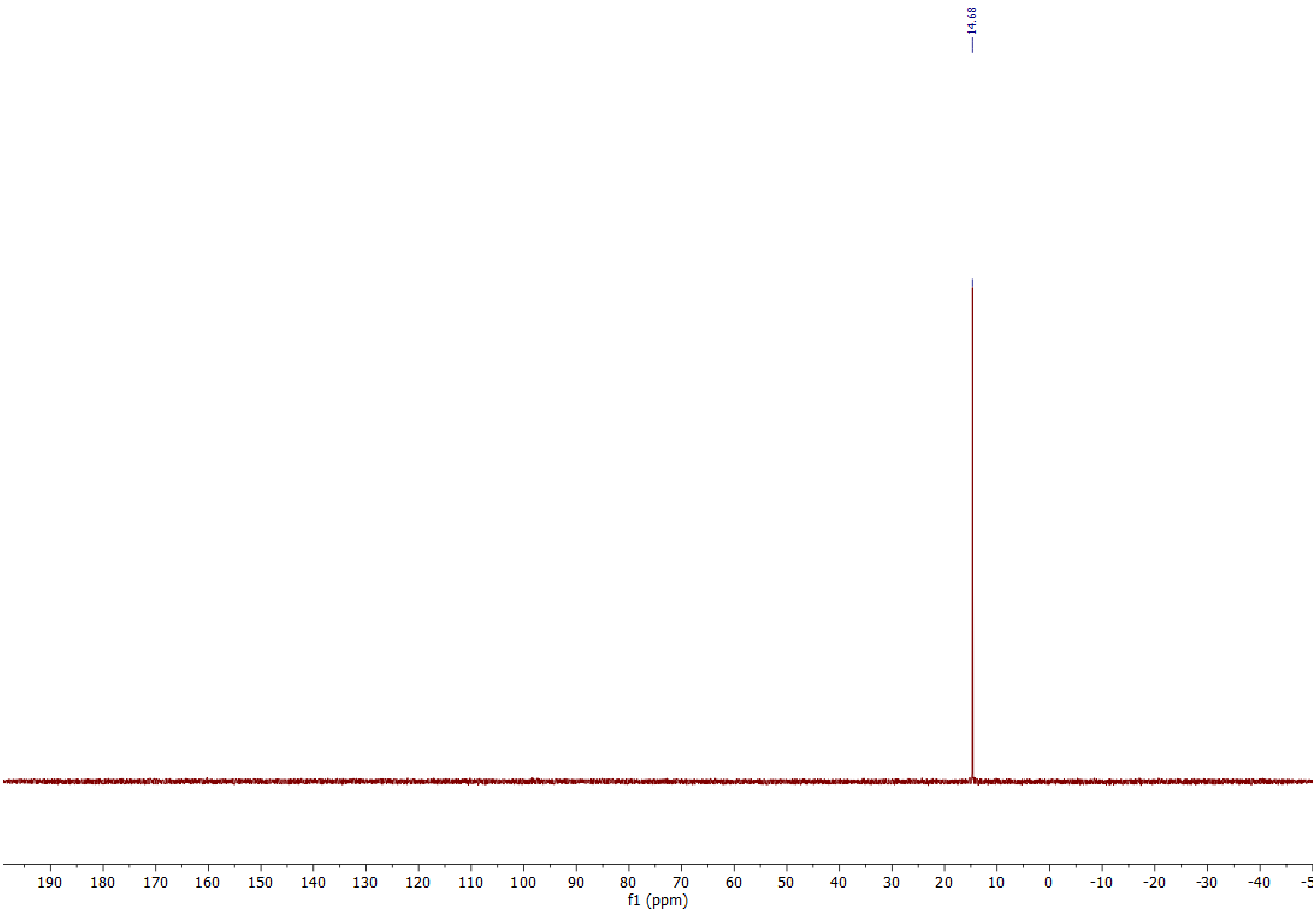

Figure S125. ^{31}P NMR spectrum of **5h** diastereomer 1.

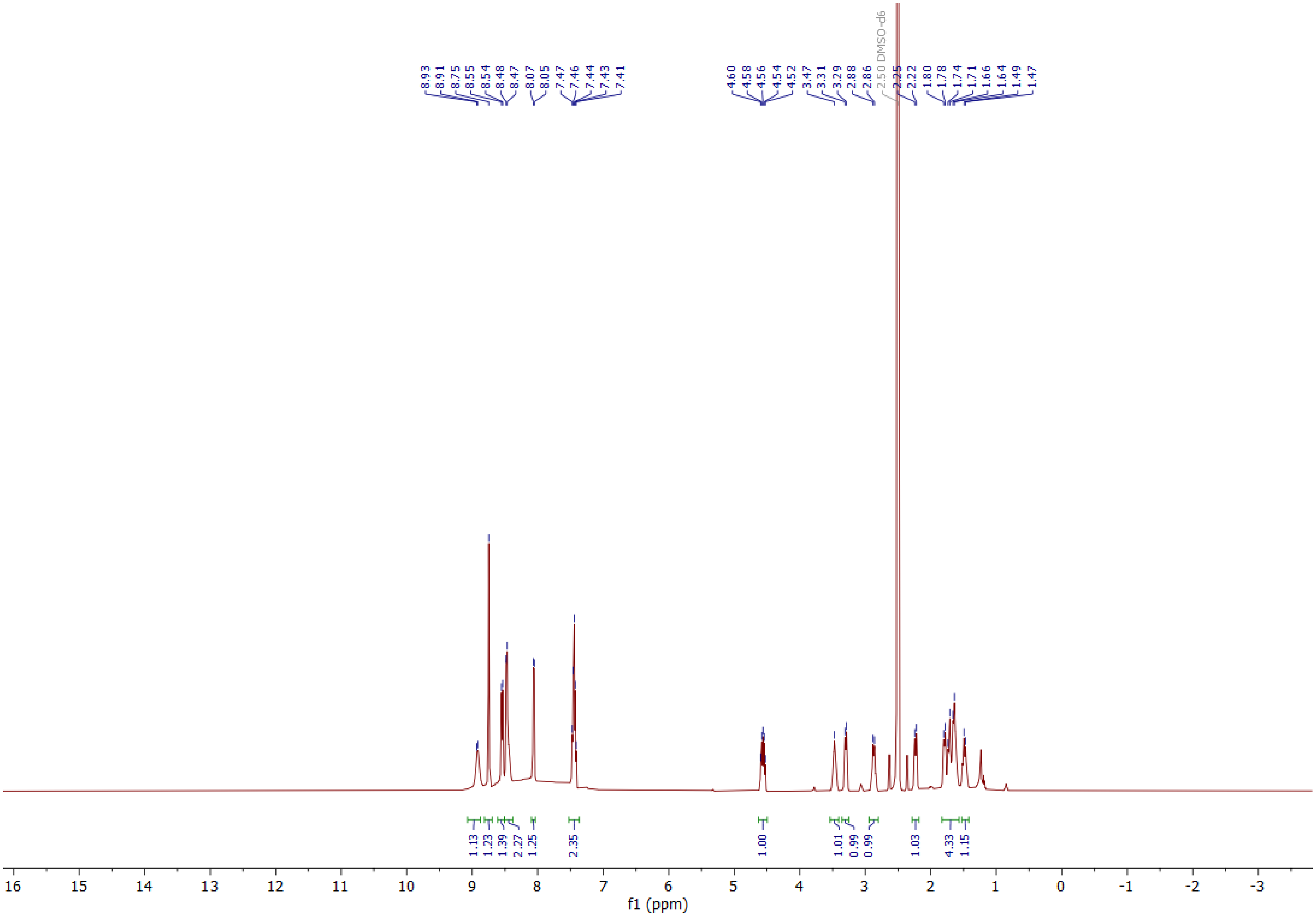

Figure S126. ^{1}H NMR spectrum of **5h** diastereomer 2.

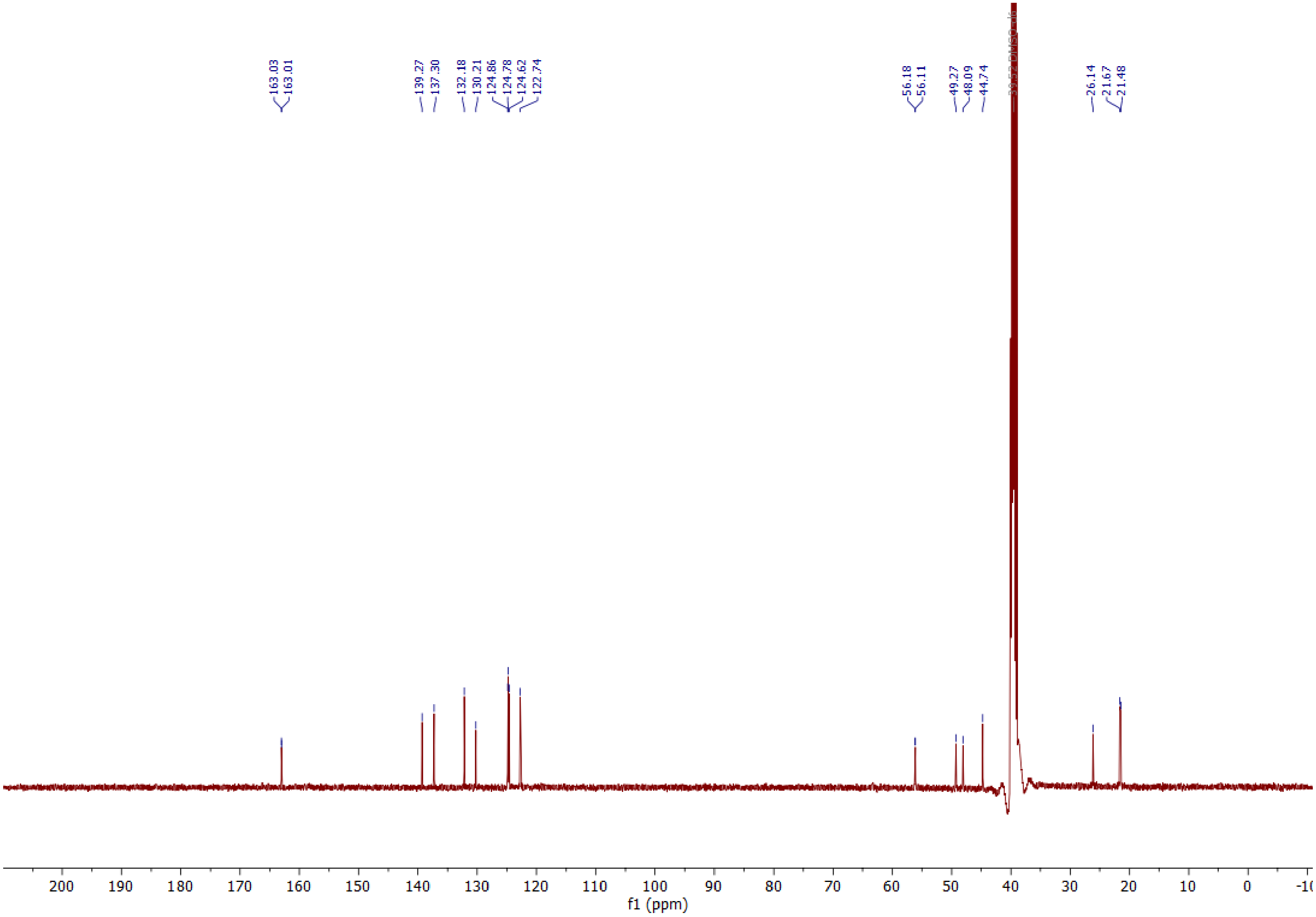

Figure S127. ^{13}C NMR spectrum of **5h** diastereomer 2.

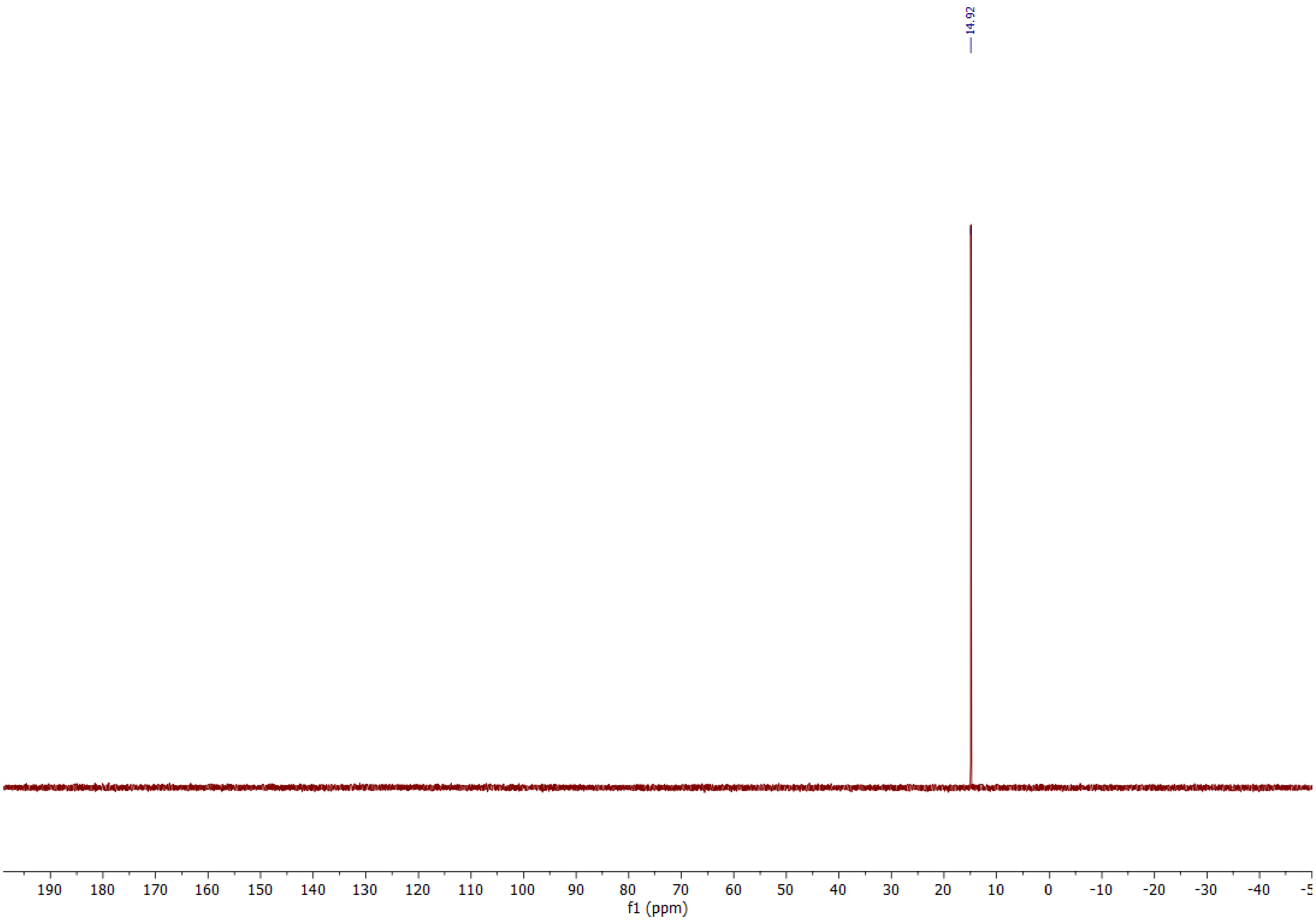

Figure S128. ^{31}P NMR spectrum of **5h** diastereomer 2.

(Piperidin-2-yl(2-(thiophen-2-yl)acetamido)methyl)phosphonic acid hydrochloride (**5i**)

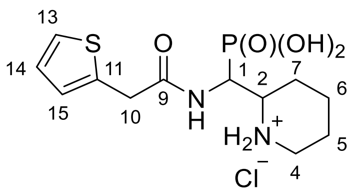

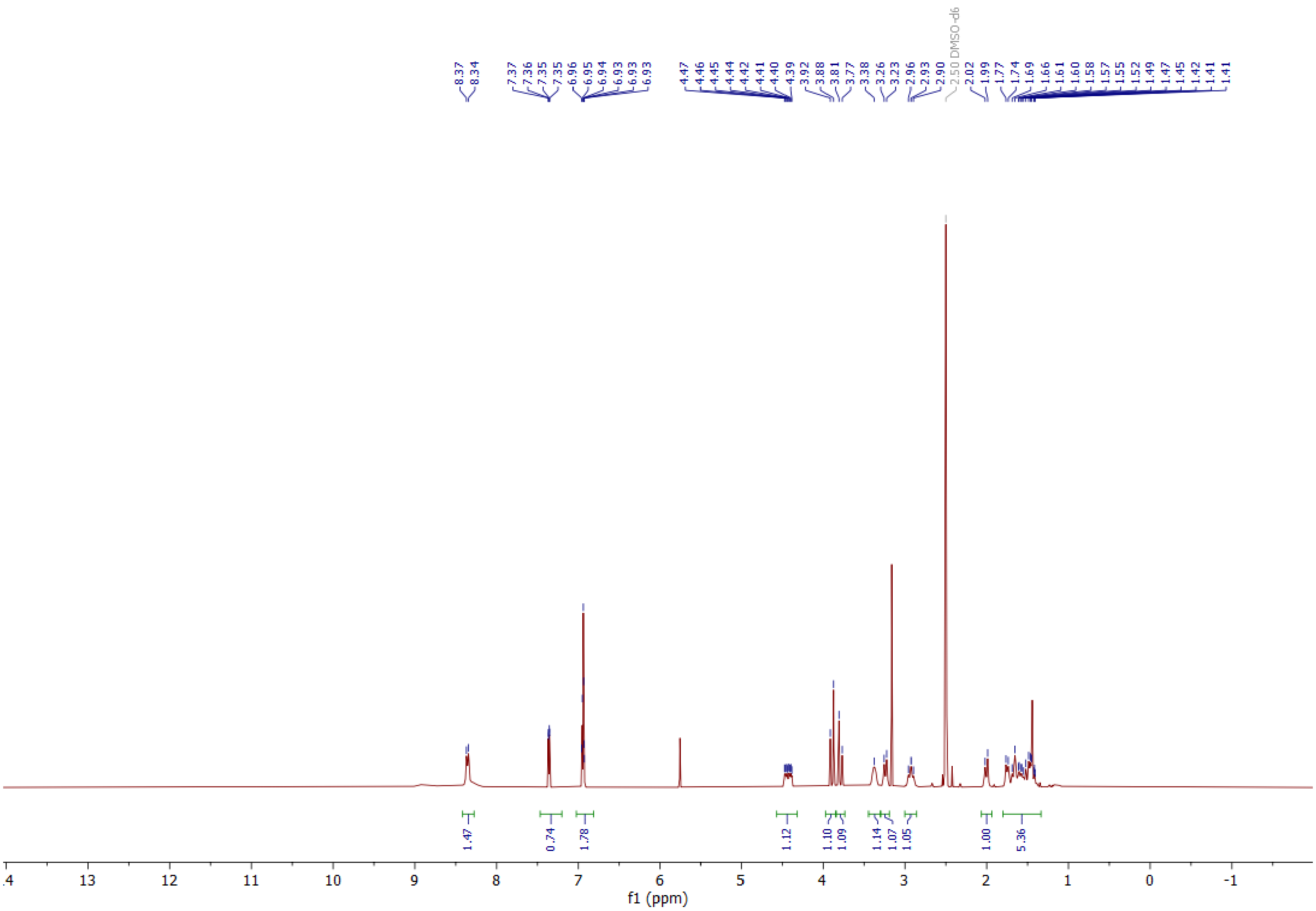

Figure S129. ^{1}H NMR spectrum of **5i** diastereomer 1.

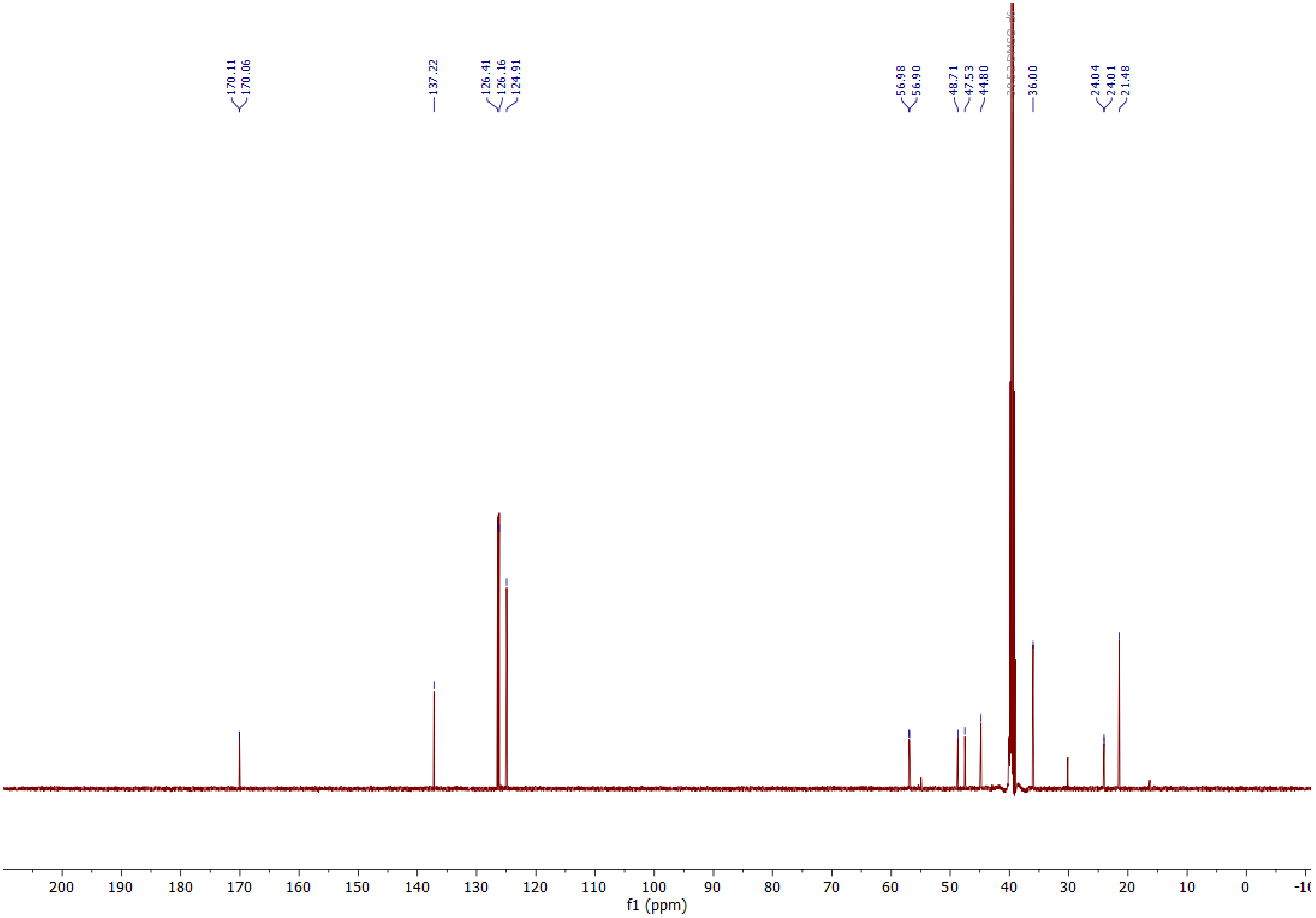

Figure S130. ^{13}C NMR spectrum of **5i** diastereomer 1.

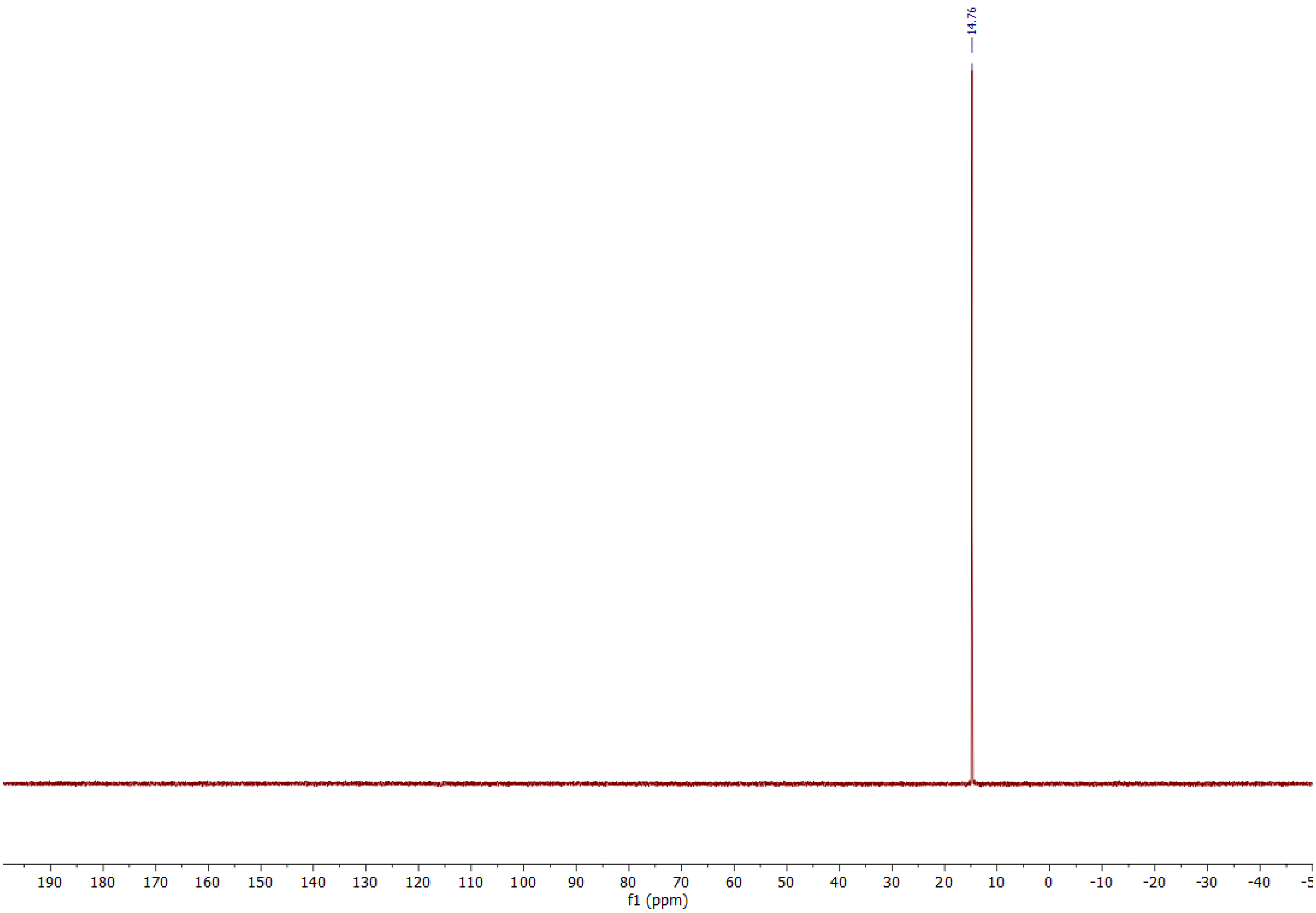

Figure S131. ^{31}P NMR spectrum of **5i** diastereomer 1.

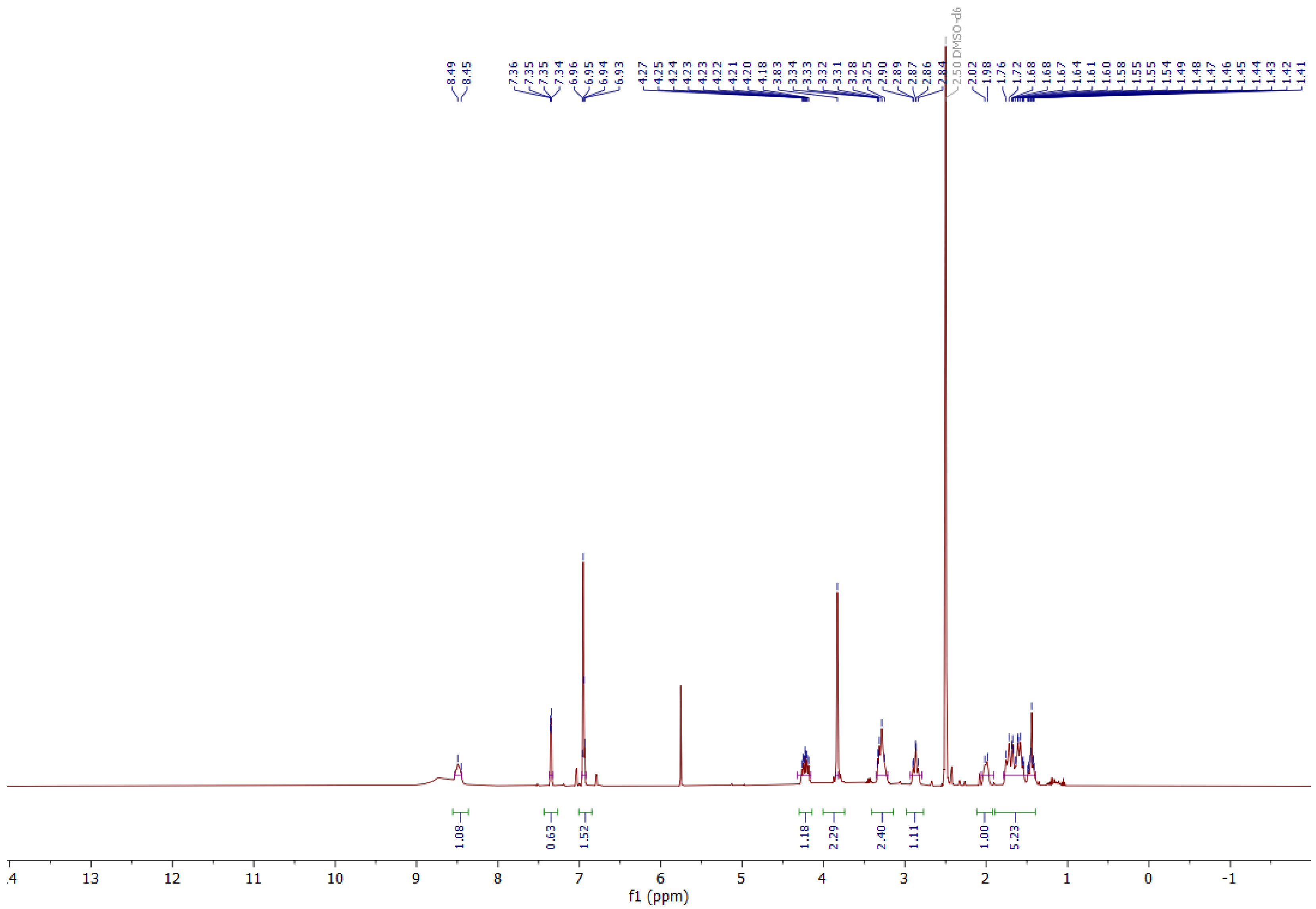

Figure S132. ^{1}H NMR spectrum of **5i** diastereomer 2.

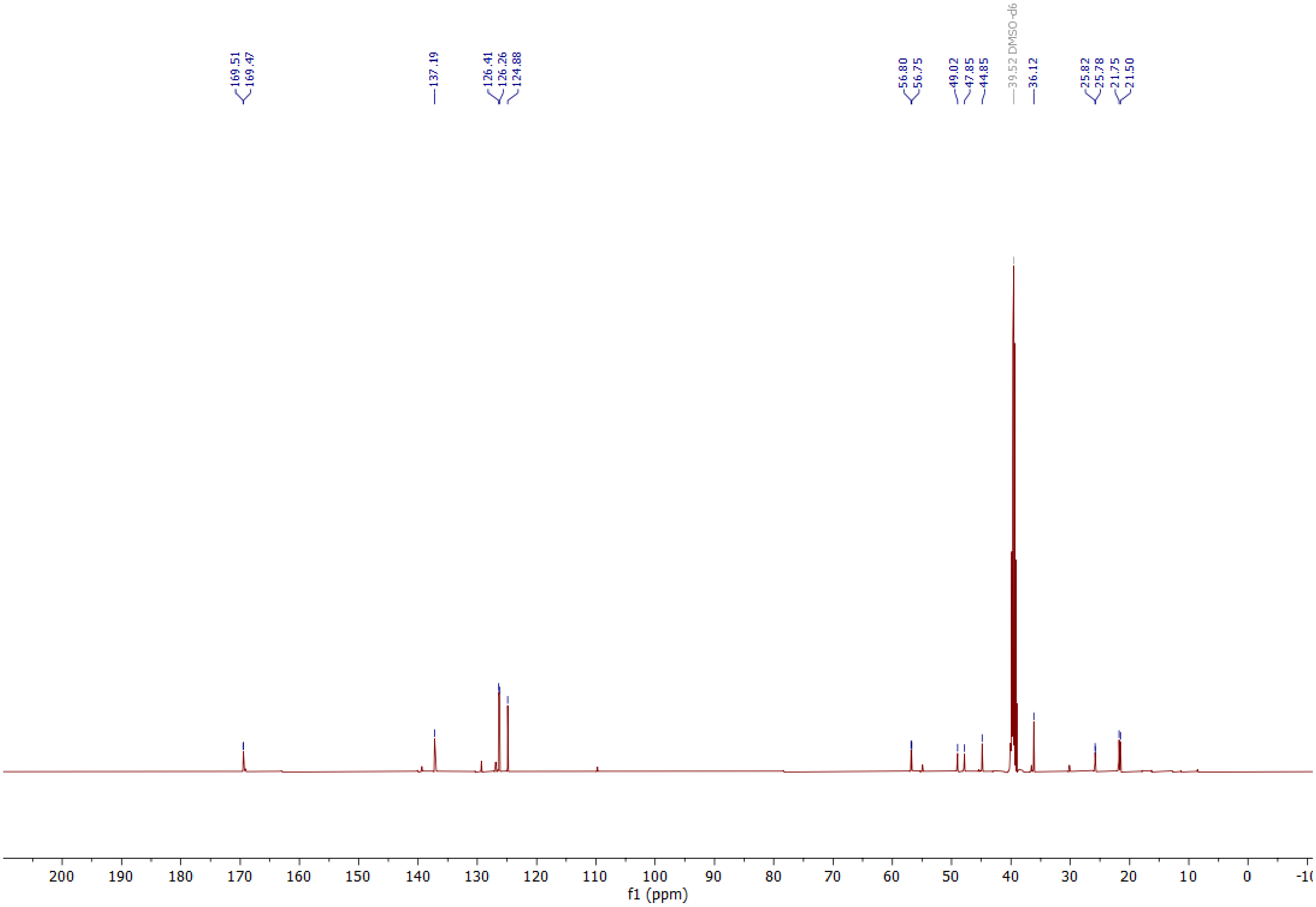

Figure S133. ^{13}C NMR spectrum of **5i** diastereomer 2.

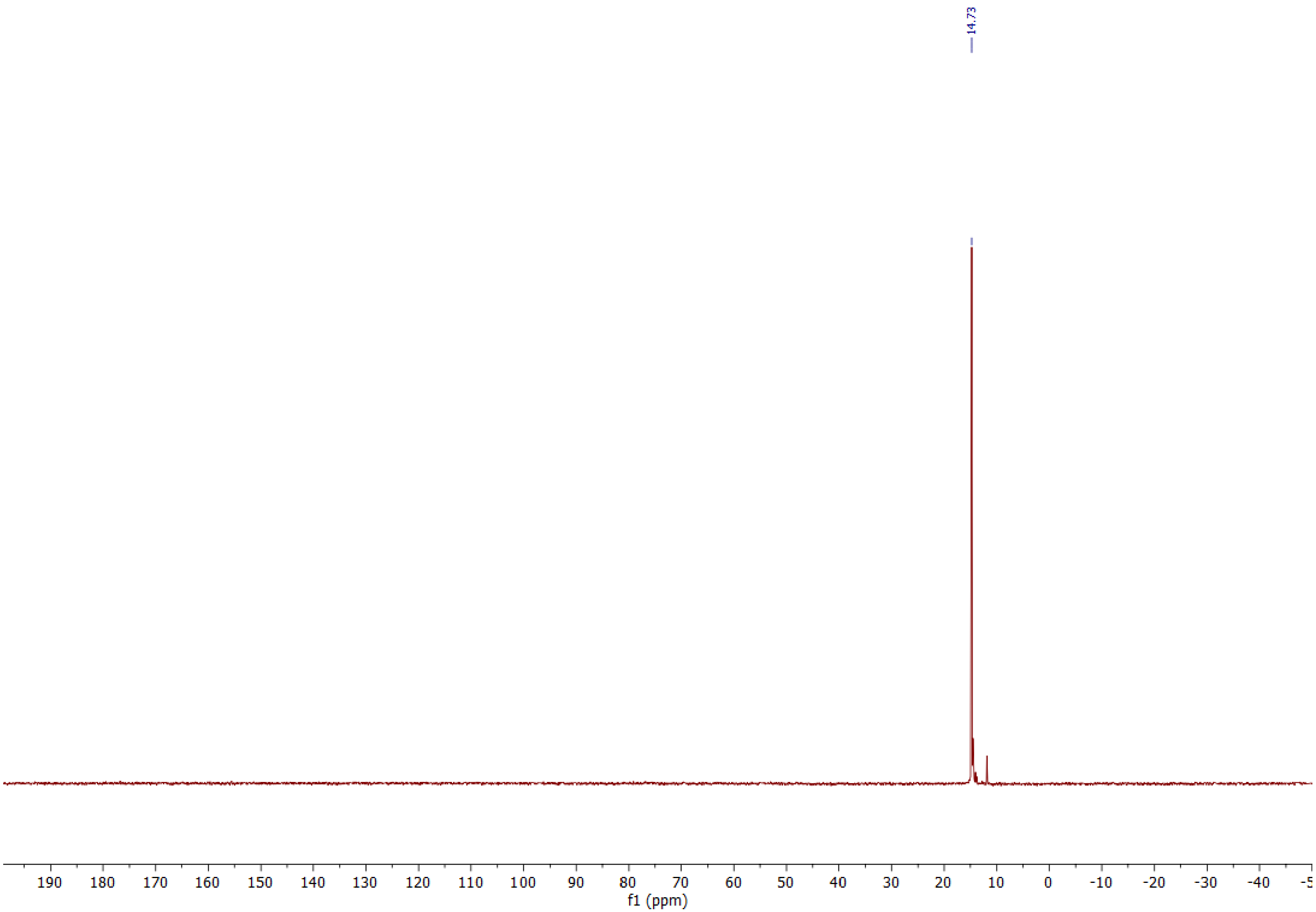

Figure S134. ^{31}P NMR spectrum of **5i** diastereomer 2.

(Piperidin-2-yl(2-(thiophen-3-yl)acetamido)methyl)phosphonic acid hydrochloride (**5j**)

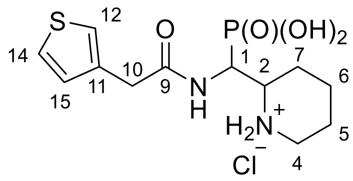

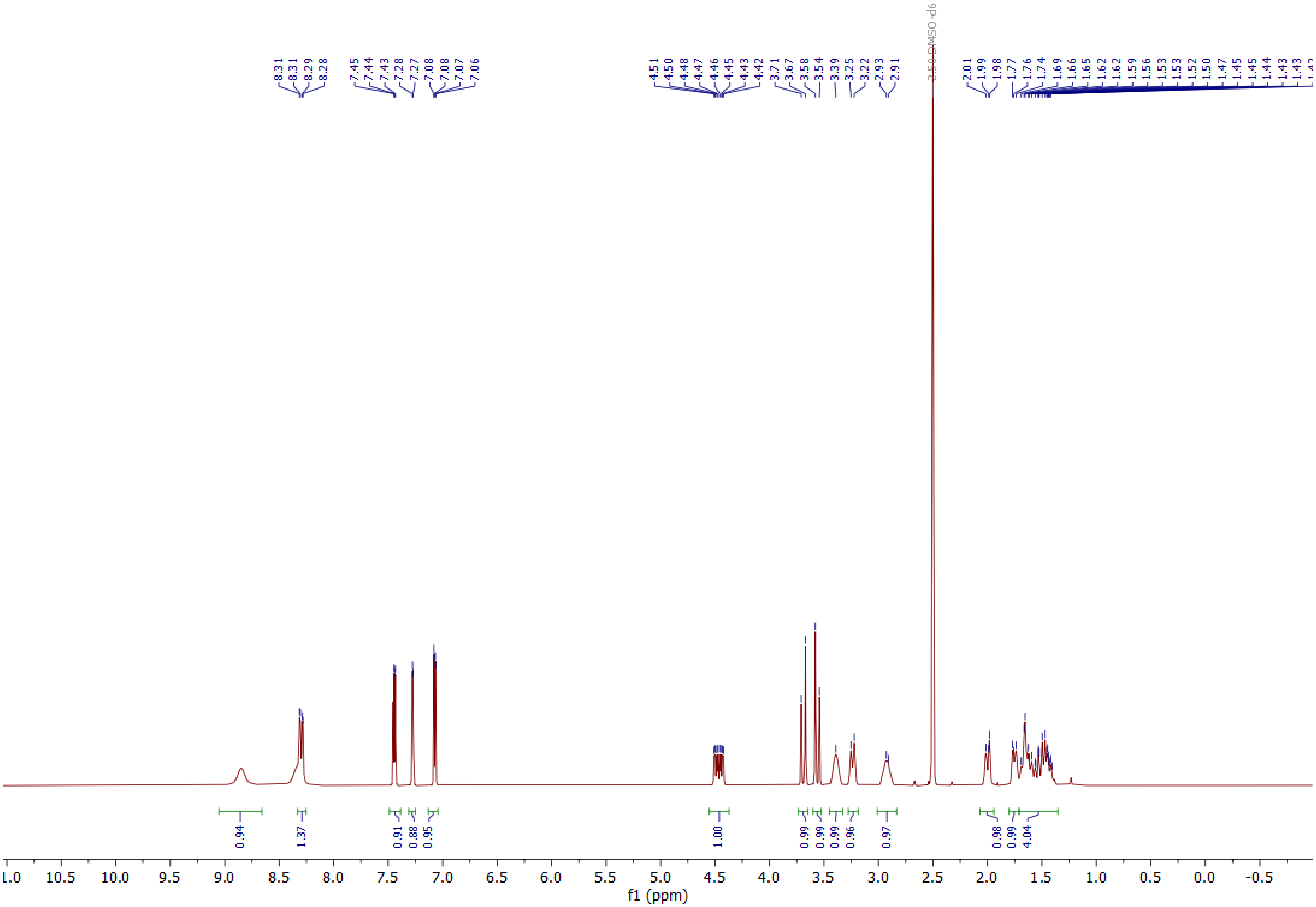

Figure S135. ^{1}H NMR spectrum of **5j** diastereomer 1.

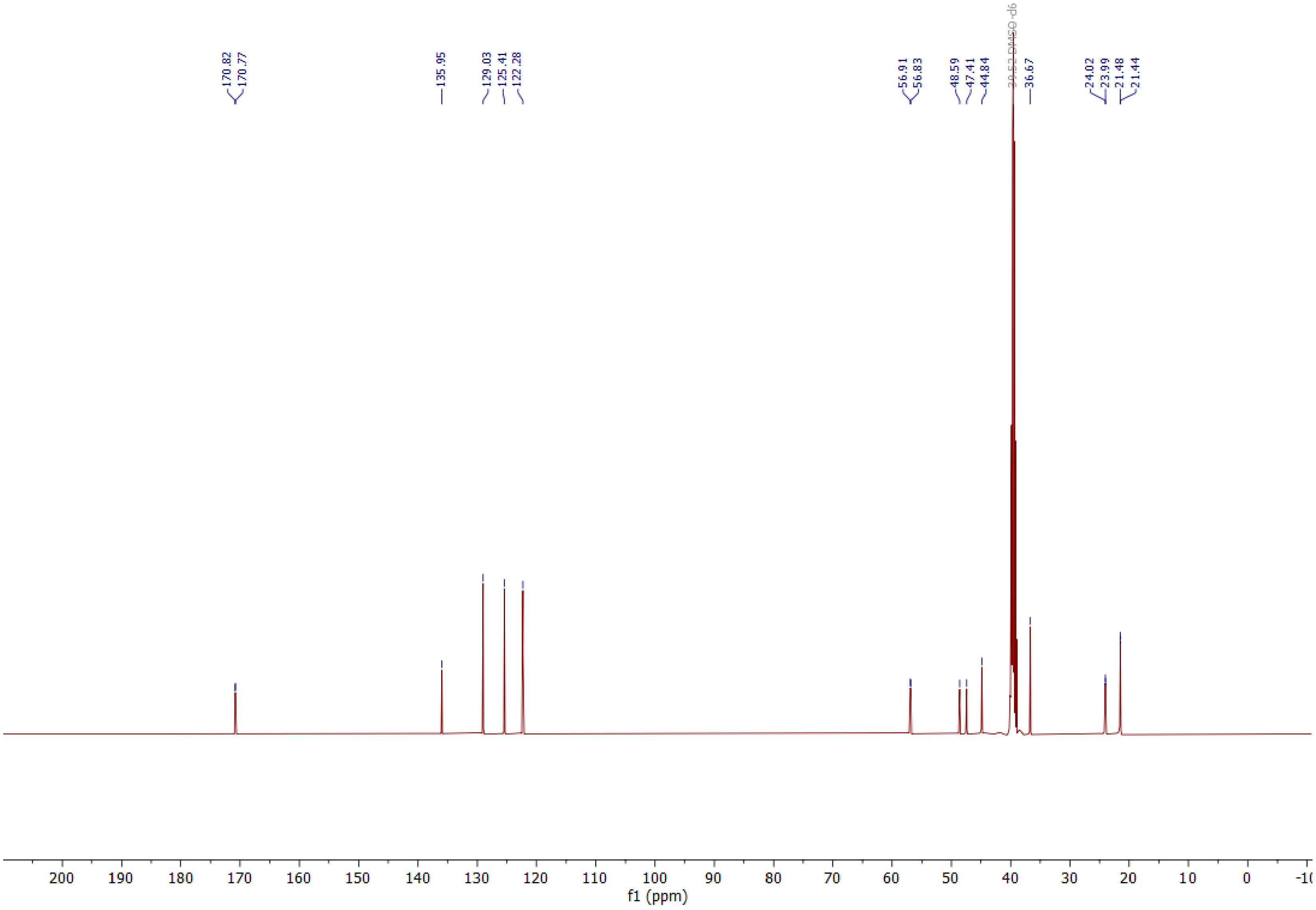

Figure S136. ^{13}C NMR spectrum of **5j** diastereomer 1.

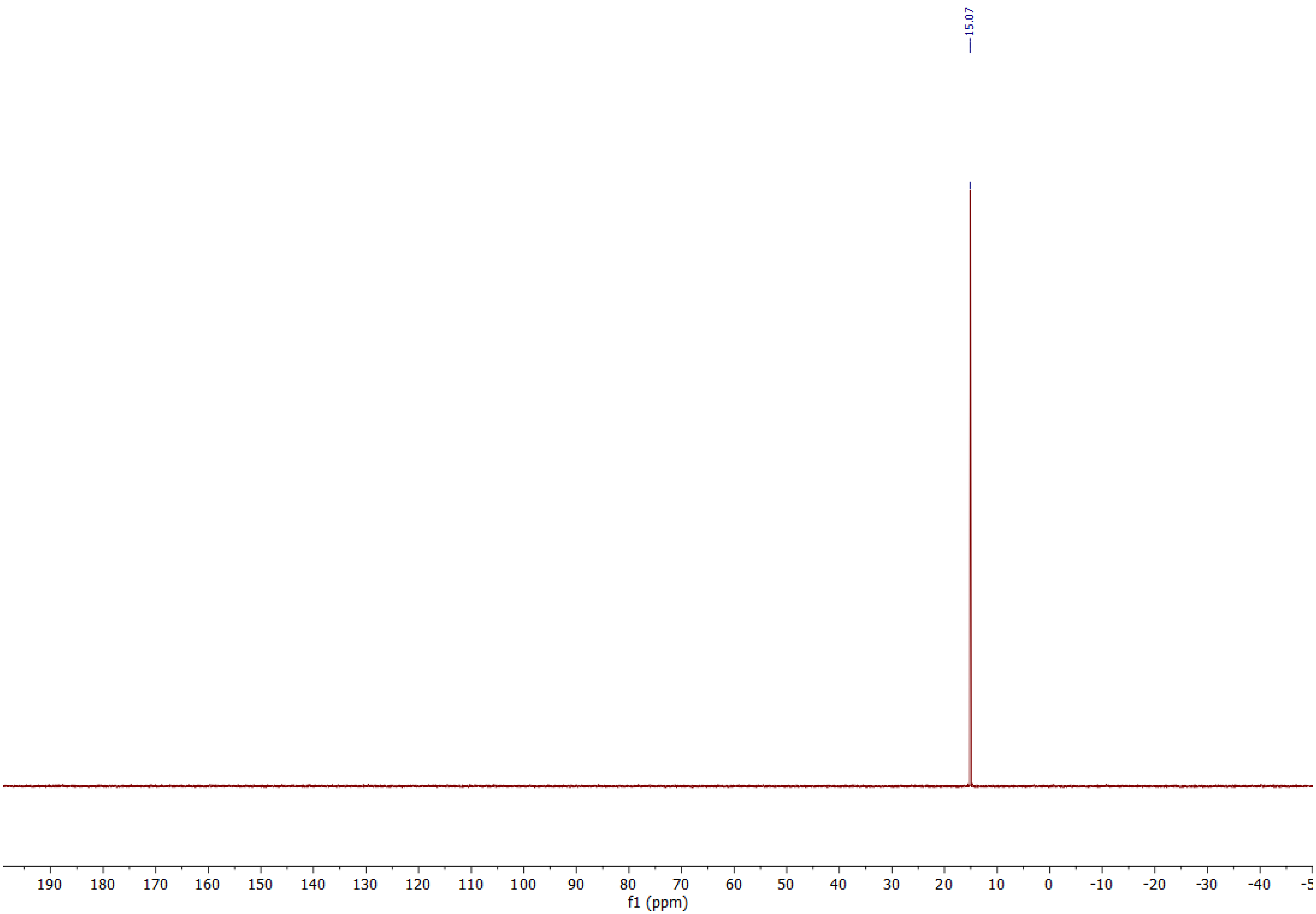

Figure S137. ^{31}P NMR spectrum of **5j** diastereomer 1.

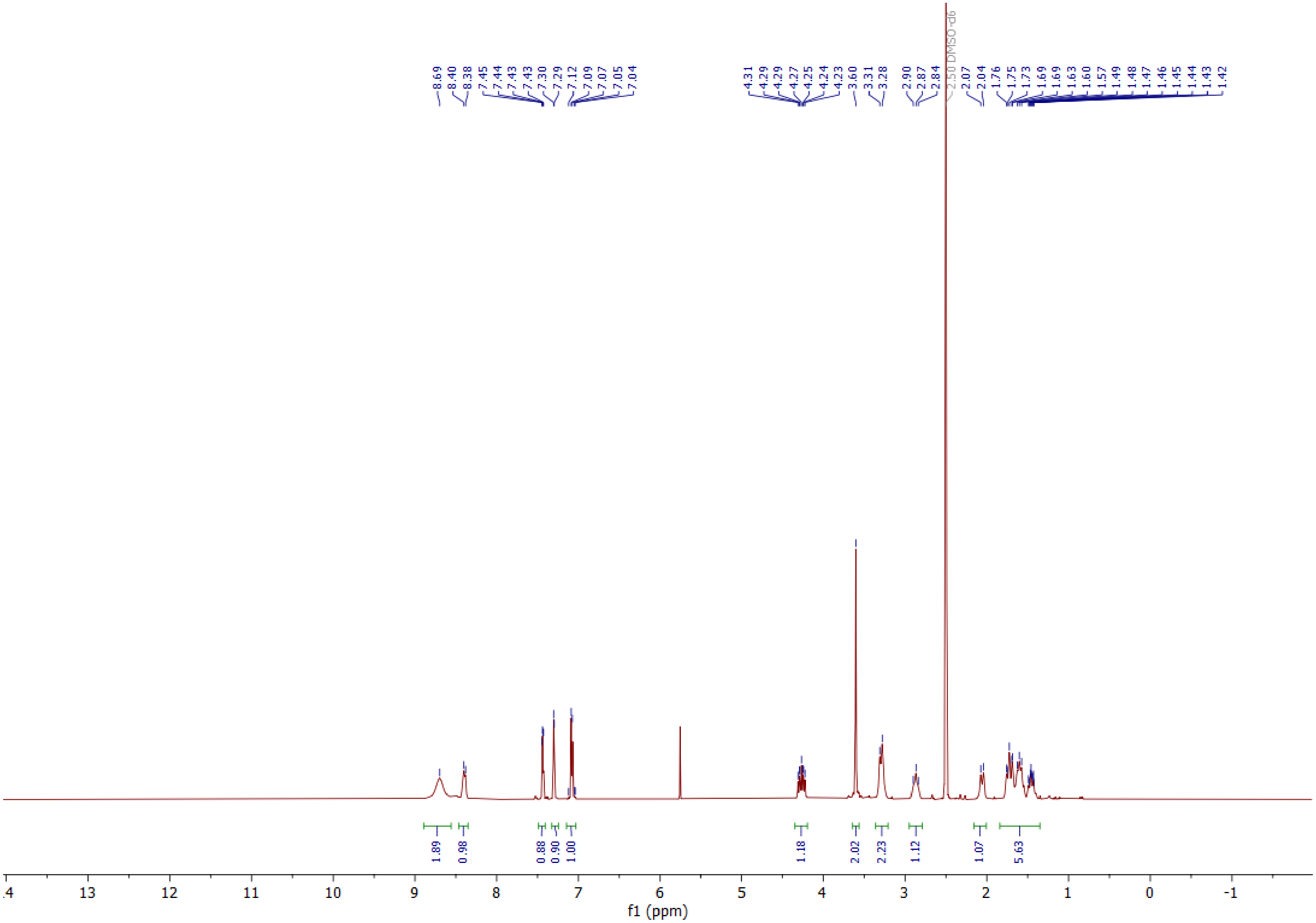

Figure S138. ^{1}H NMR spectrum of **5j** diastereomer 2.

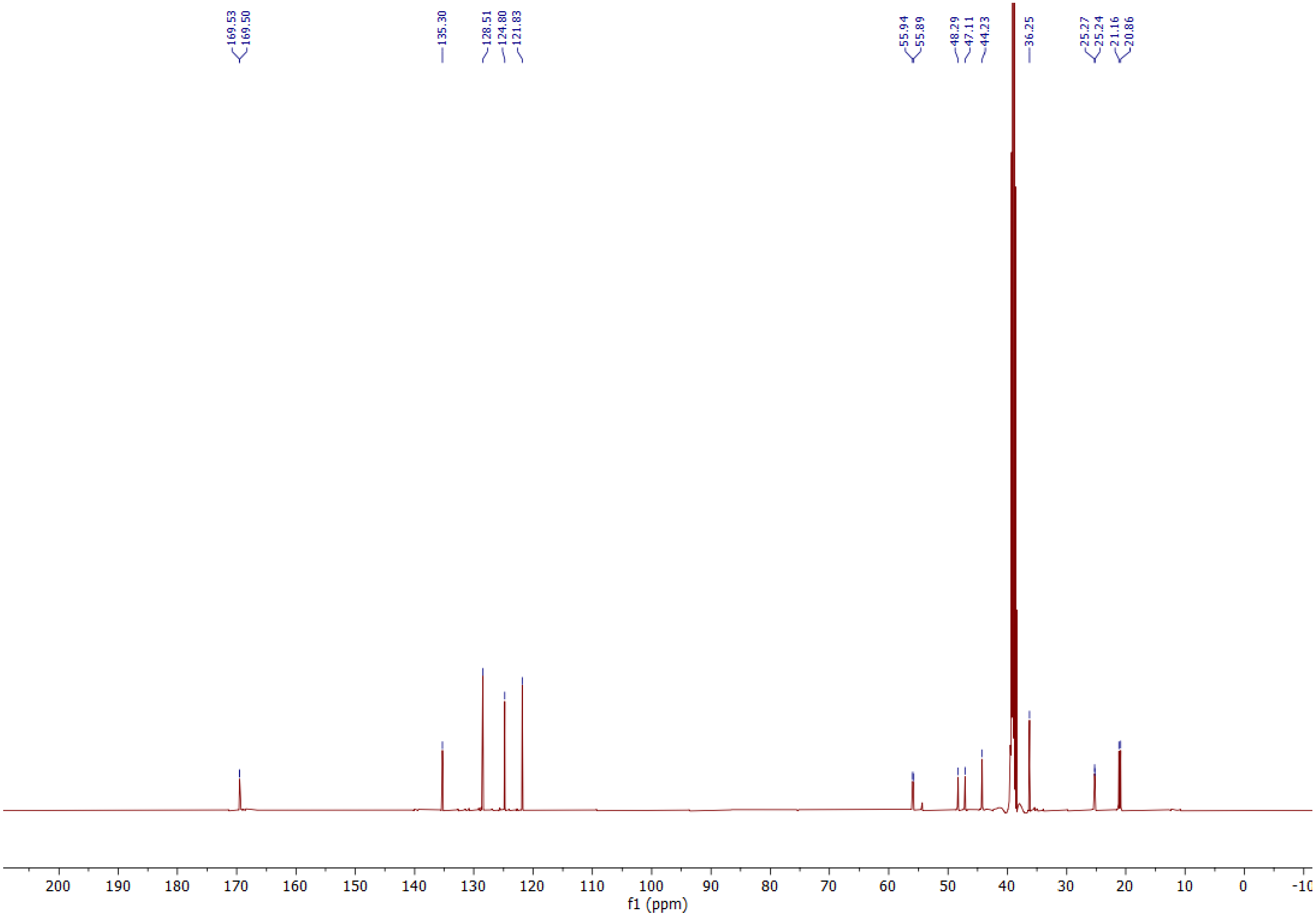

Figure S139. ^{13}C NMR spectrum of **5j** diastereomer 2.

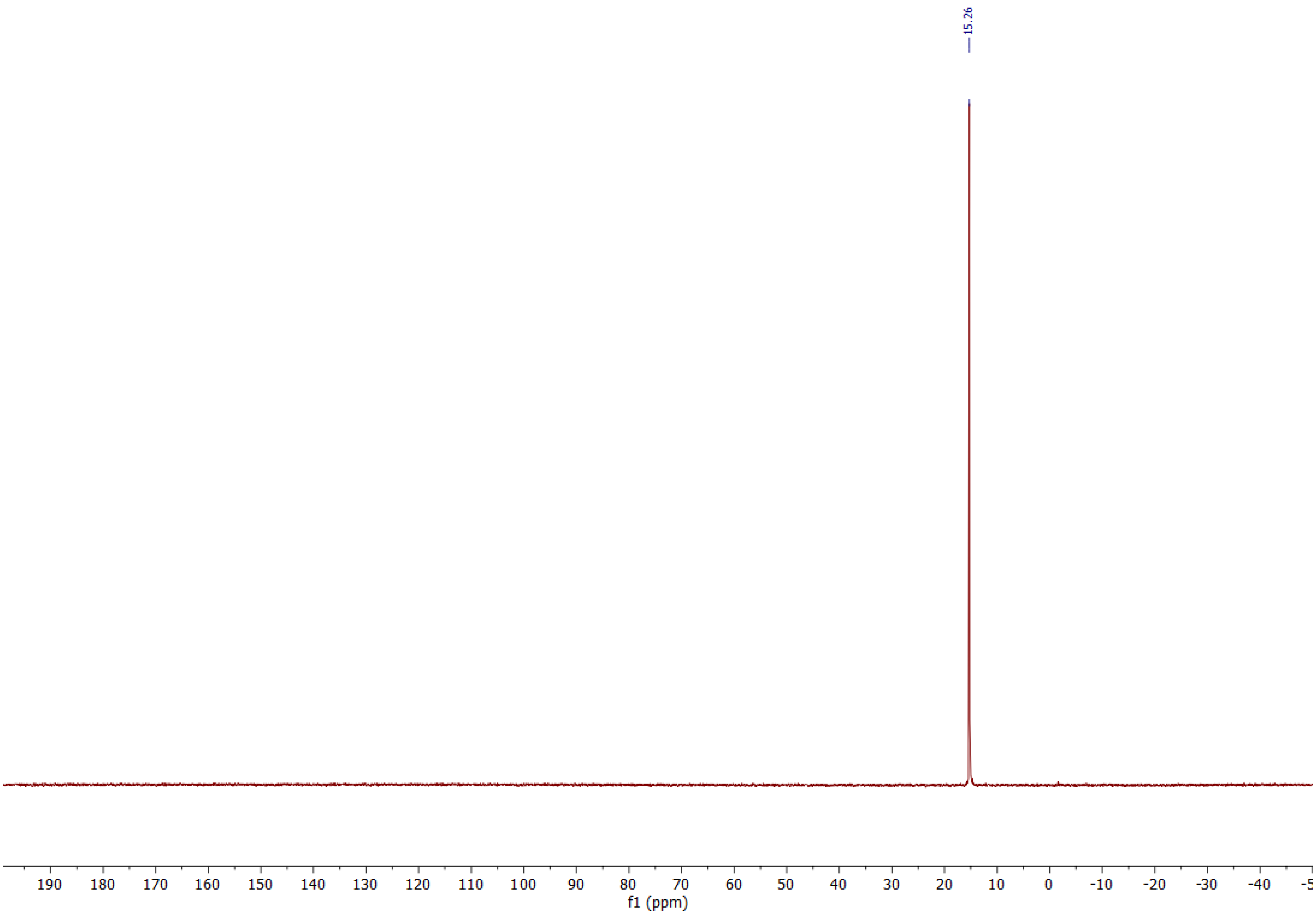

Figure S140. ^{31}P NMR spectrum of **5j** diastereomer 2.

((2-(Benzo[*b*]thiophen-2-yl)acetamido)(piperidin-2-yl)methyl)phosphonic acid hydrochloride (**5k**)

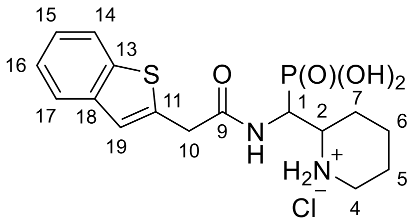

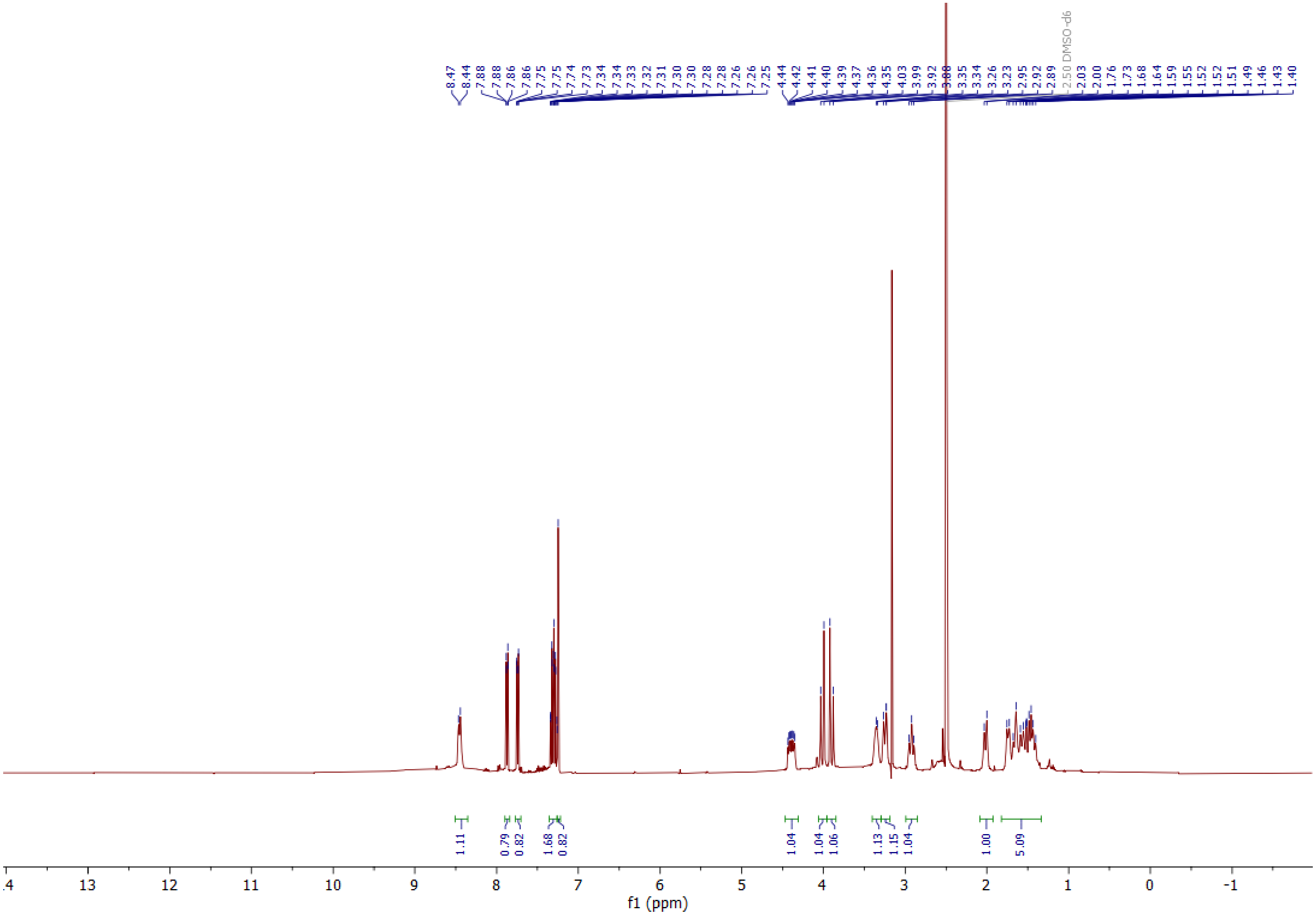

Figure S141. ^{1}H NMR spectrum of **5k** diastereomer 1.

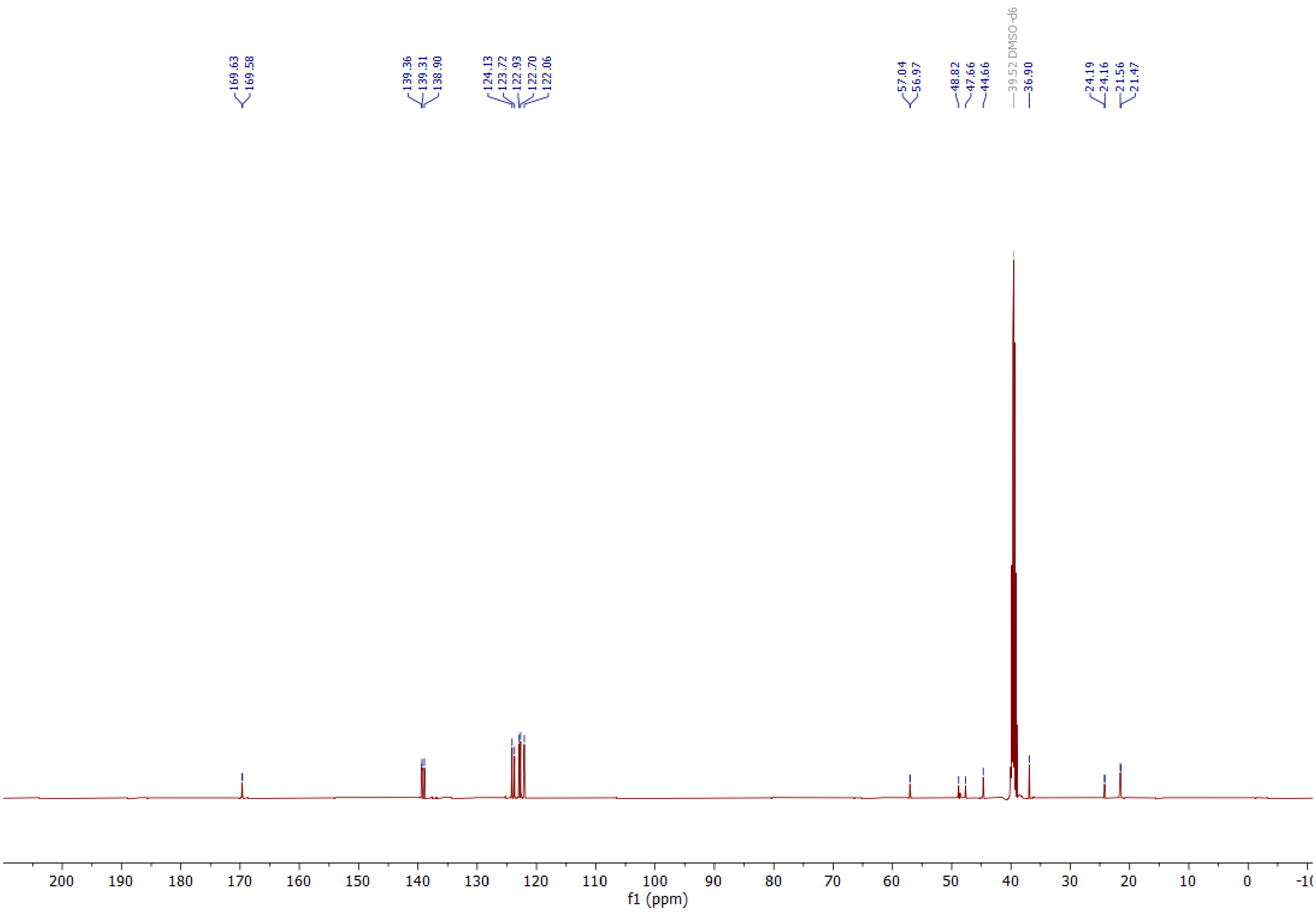

Figure S142. ^{13}C NMR spectrum of **5k** diastereomer 1.

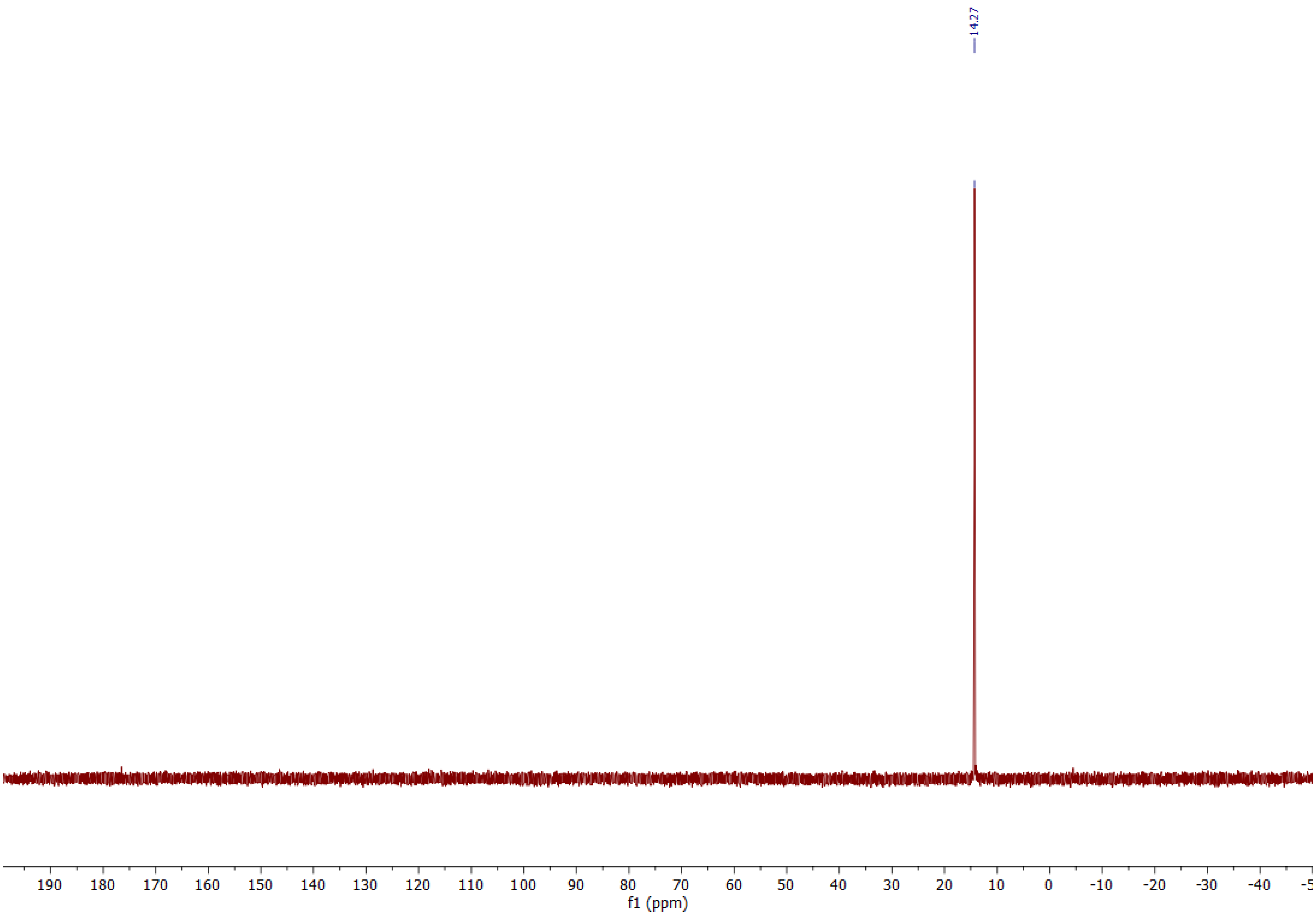

Figure S143. ^{31}P NMR spectrum of **5k** diastereomer 1.

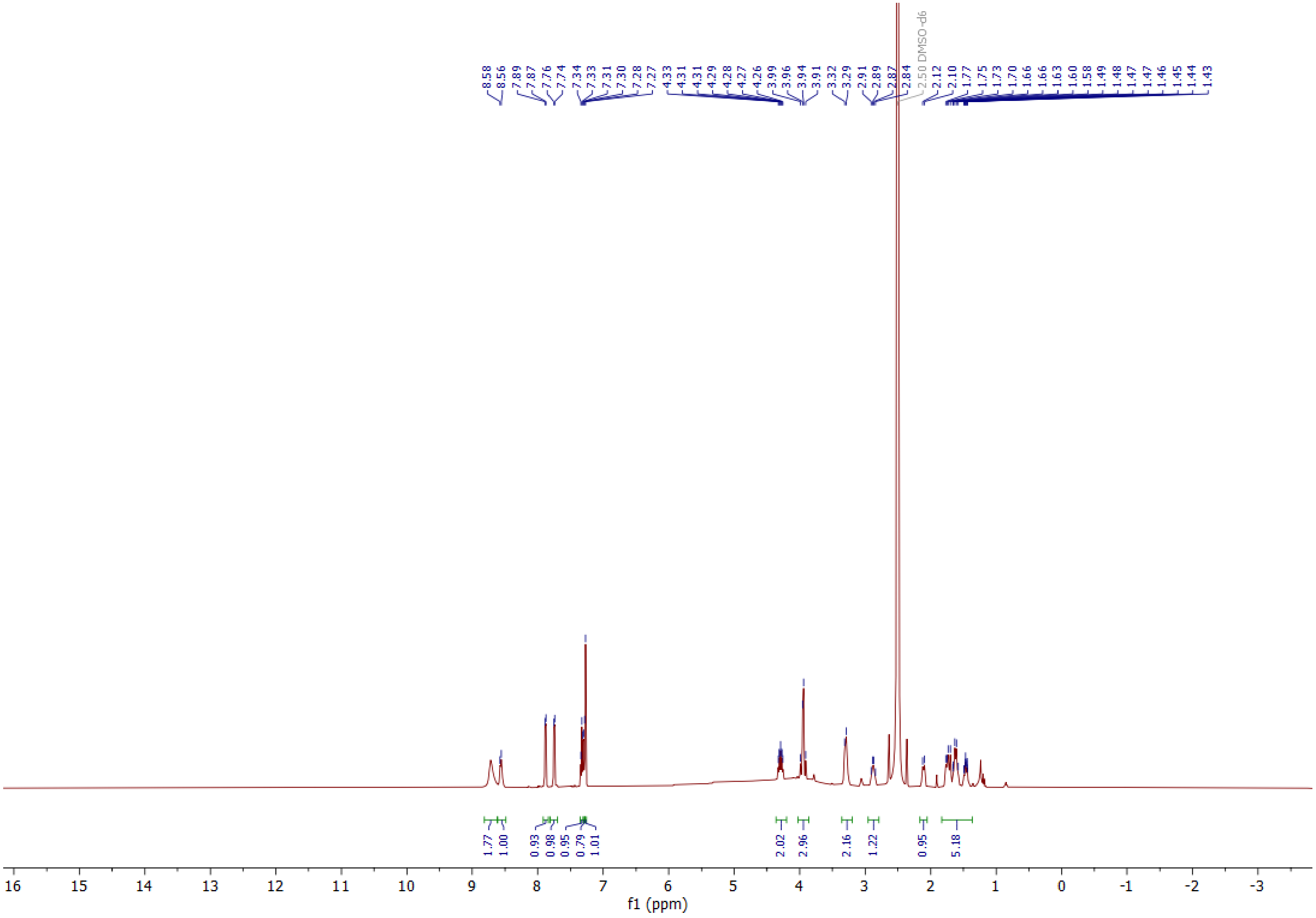

Figure S144. ^{1}H NMR spectrum of **5k** diastereomer 2.

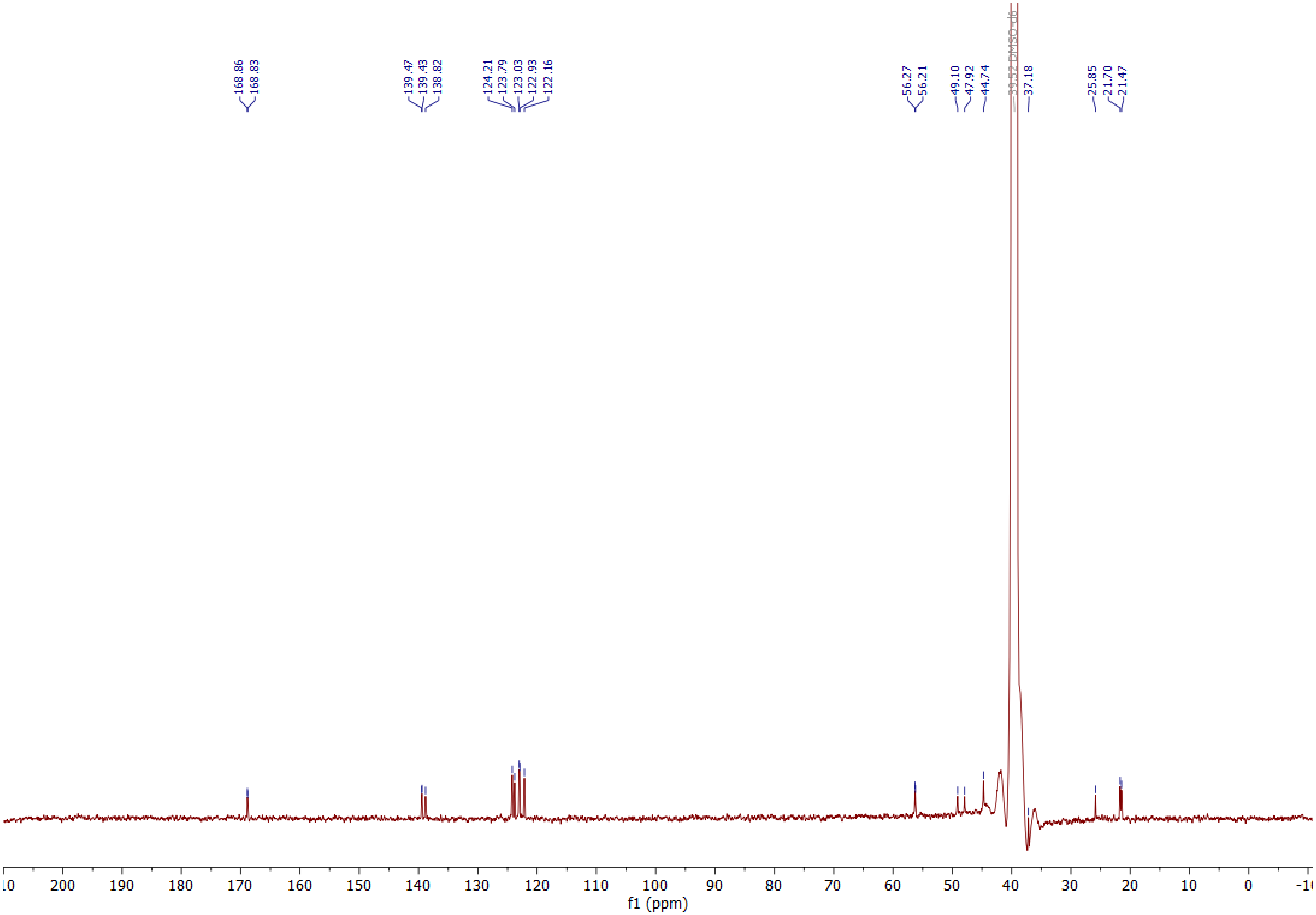

Figure S145. ^{13}C NMR spectrum of **5k** diastereomer 2.

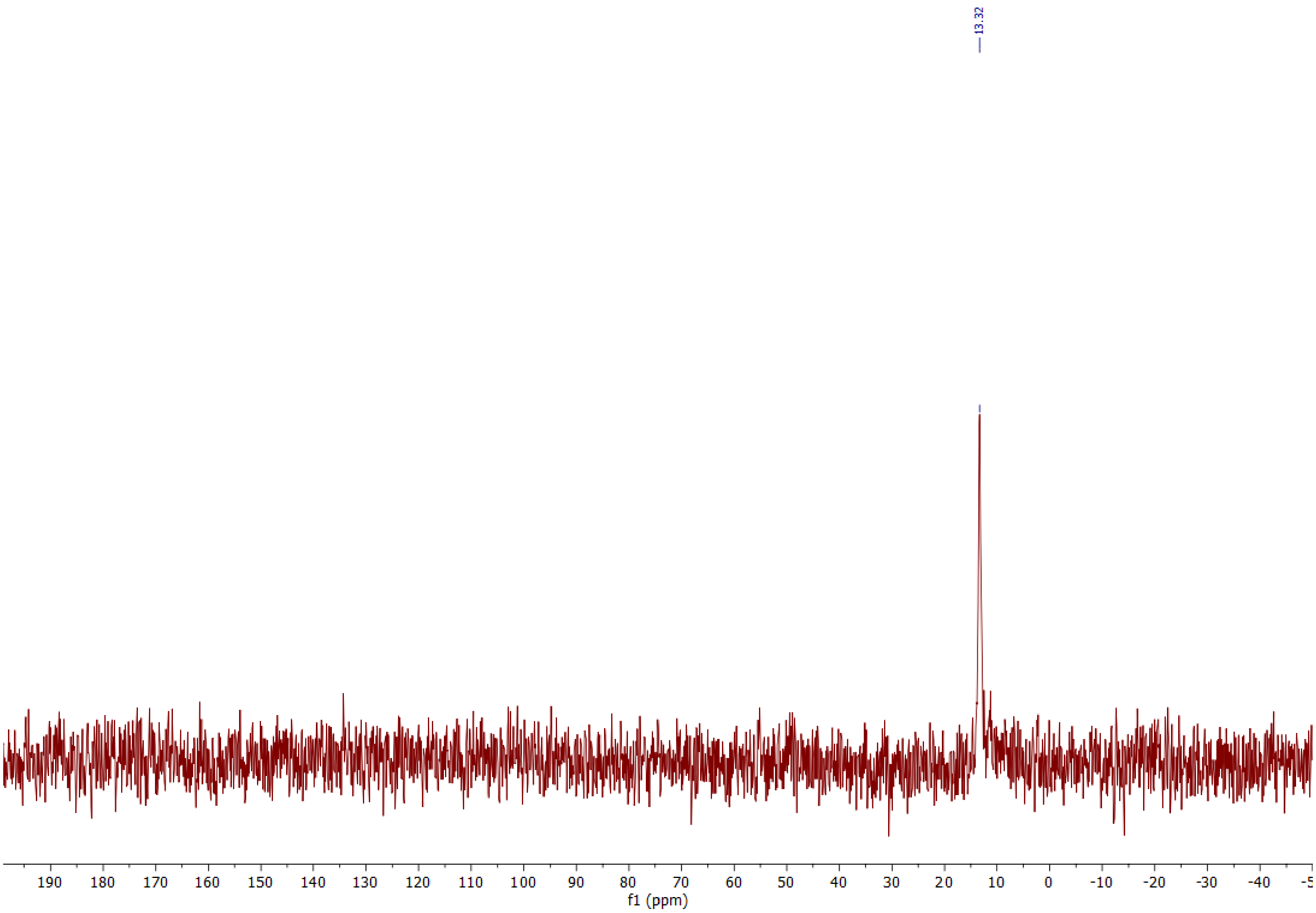

Figure S146. ^{31}P NMR spectrum of **5k** diastereomer 2.

((2-(Benzo[b]thiophen-3-yl)acetamido)(piperidin-2-yl)methyl)phosphonic acid hydrochloride (**5l**)

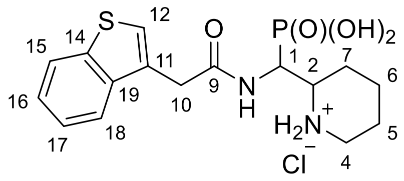

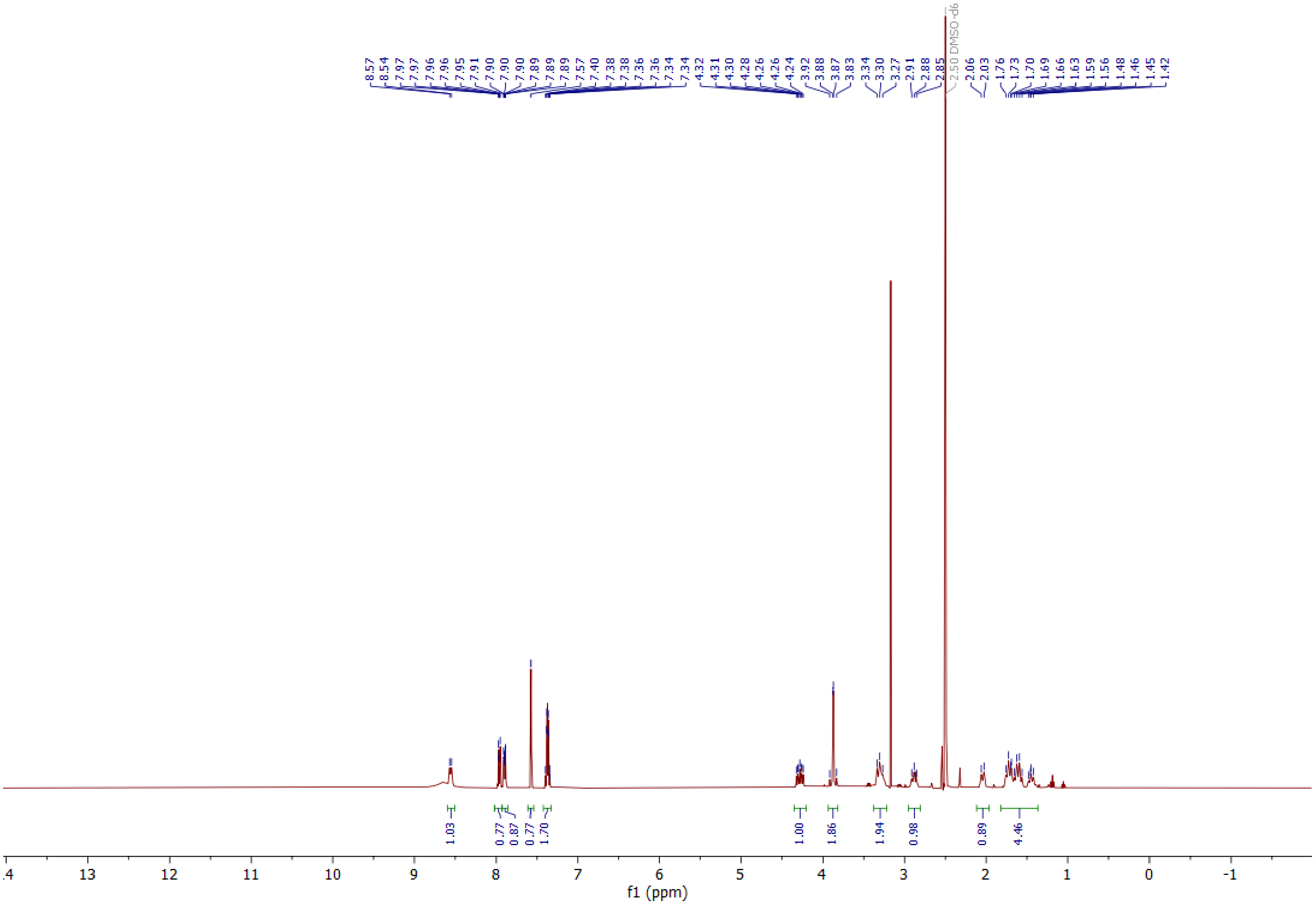

Figure S147. ^{1}H NMR spectrum of **5l** diastereomer 1.

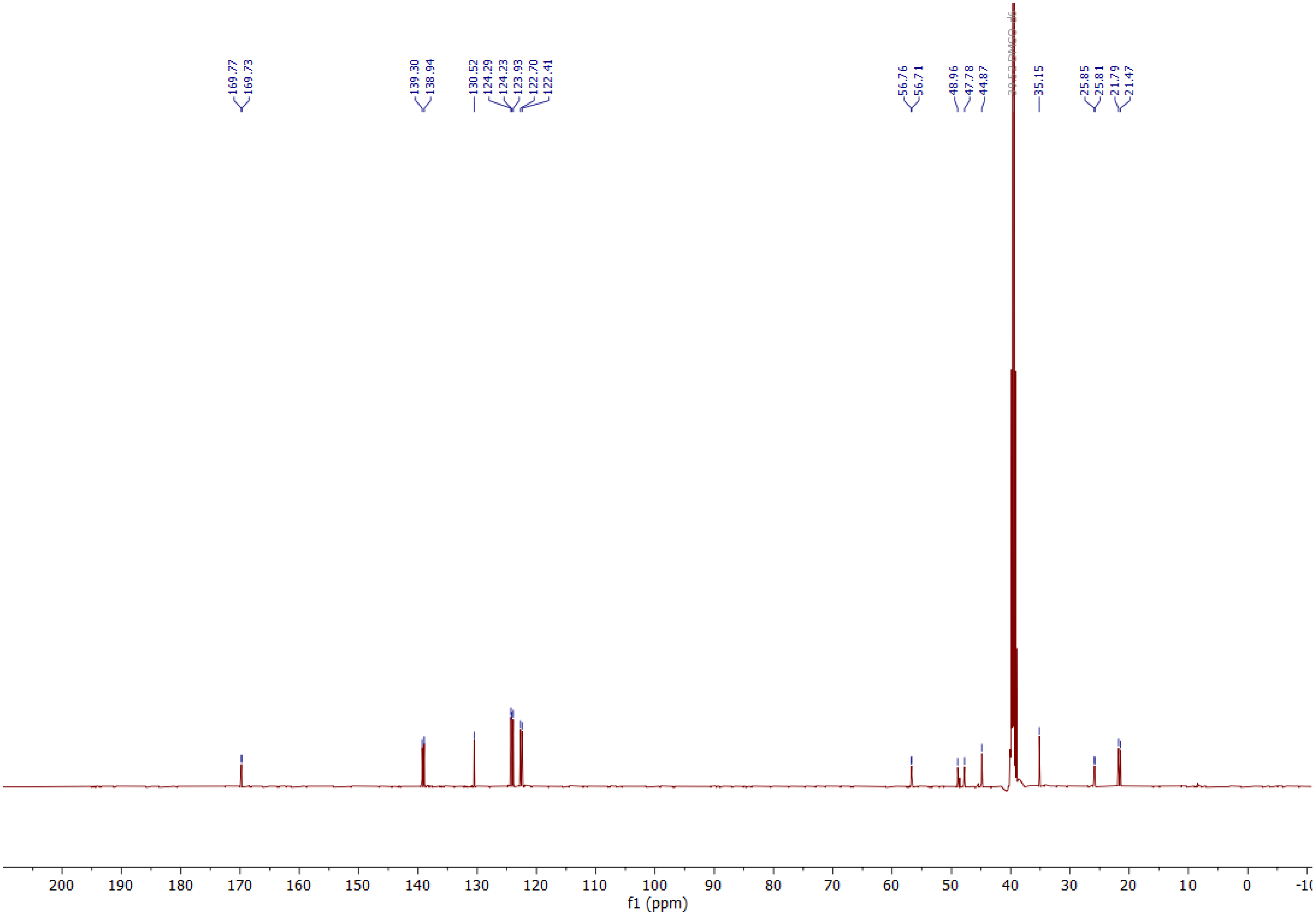

Figure S148. ^{13}C NMR spectrum of **5l** diastereomer 1.

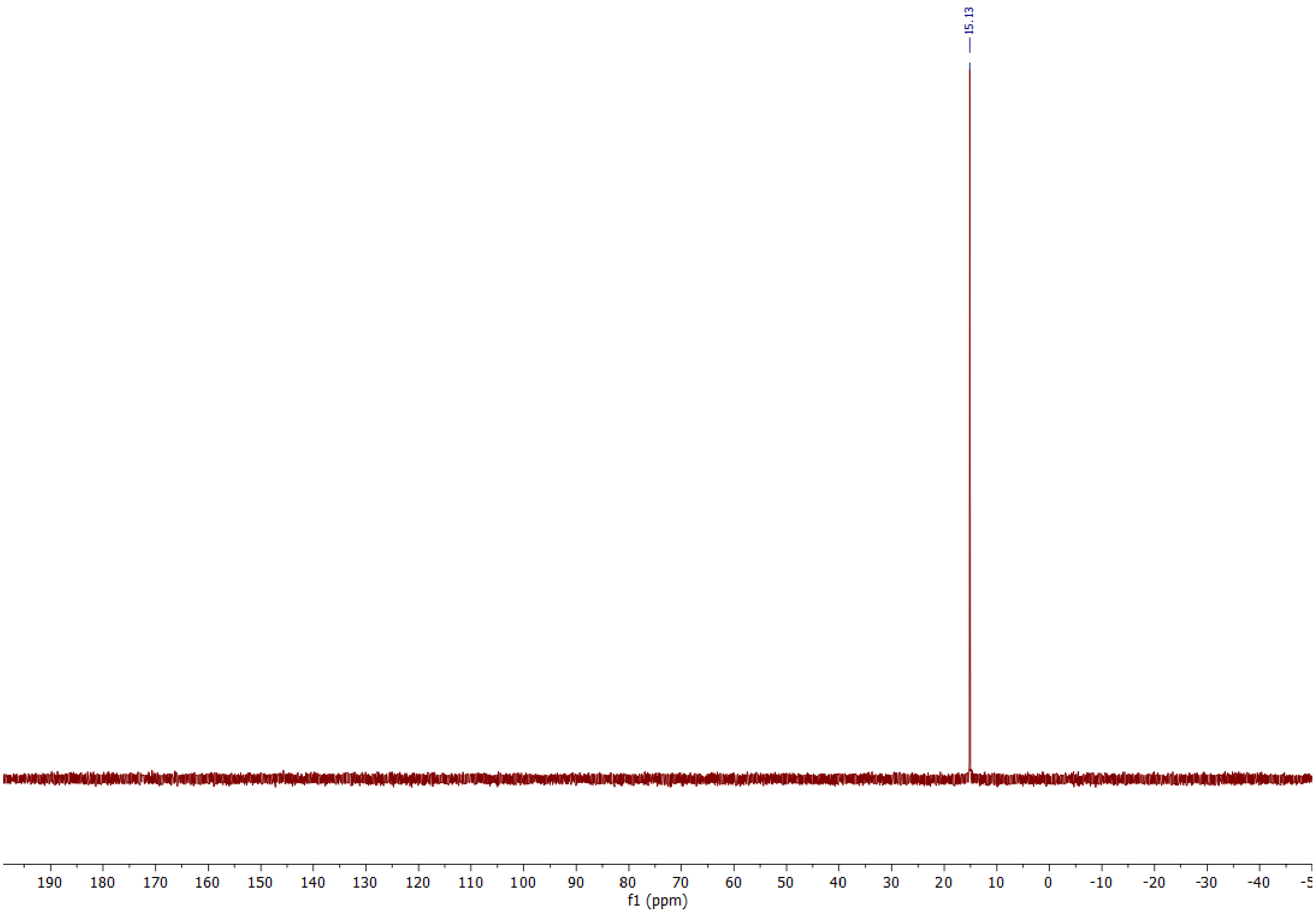

Figure S149. ^{31}P NMR spectrum of **5l** diastereomer 1.

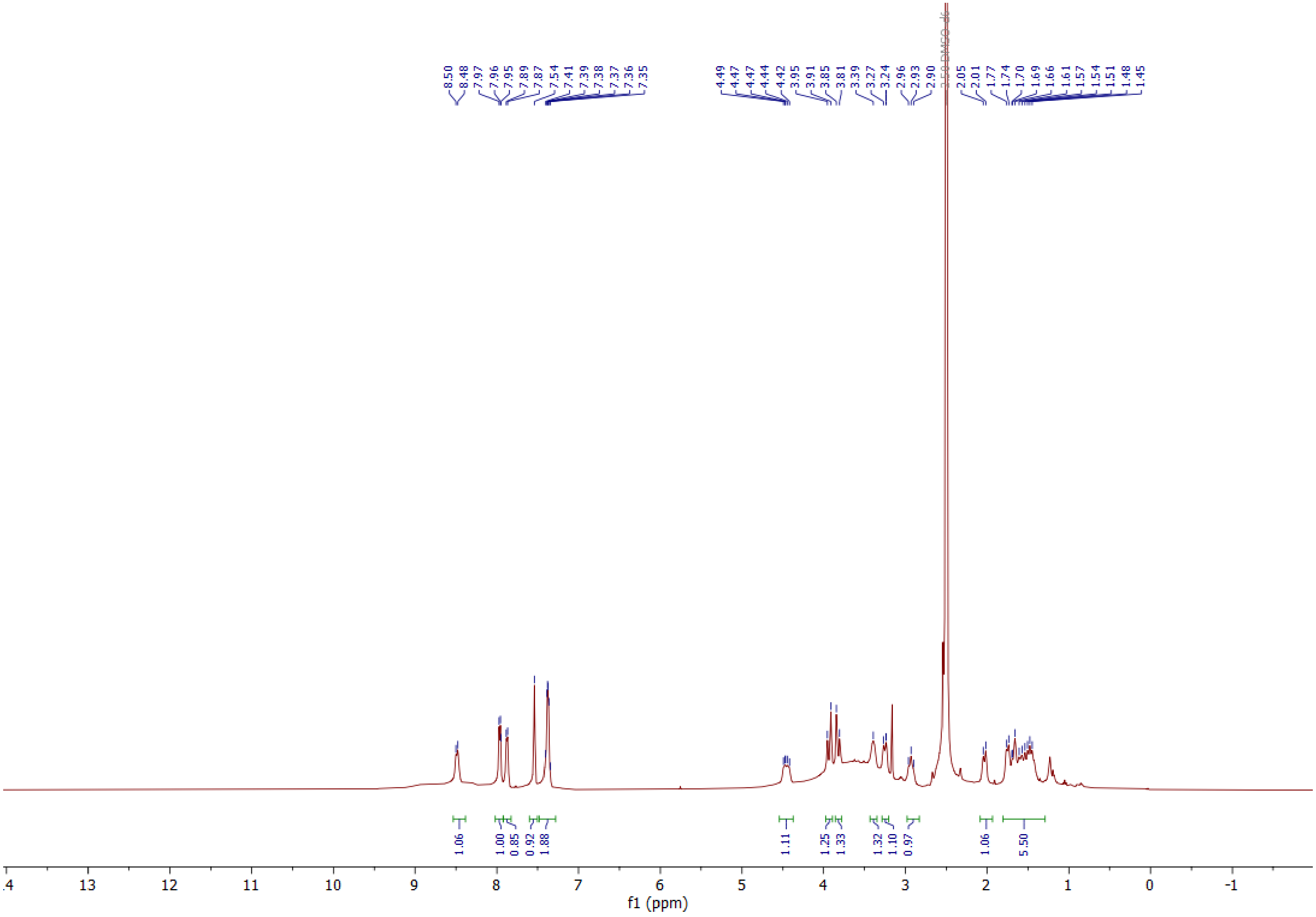

Figure S150. ^{1}H NMR spectrum of **5l** diastereomer 2.

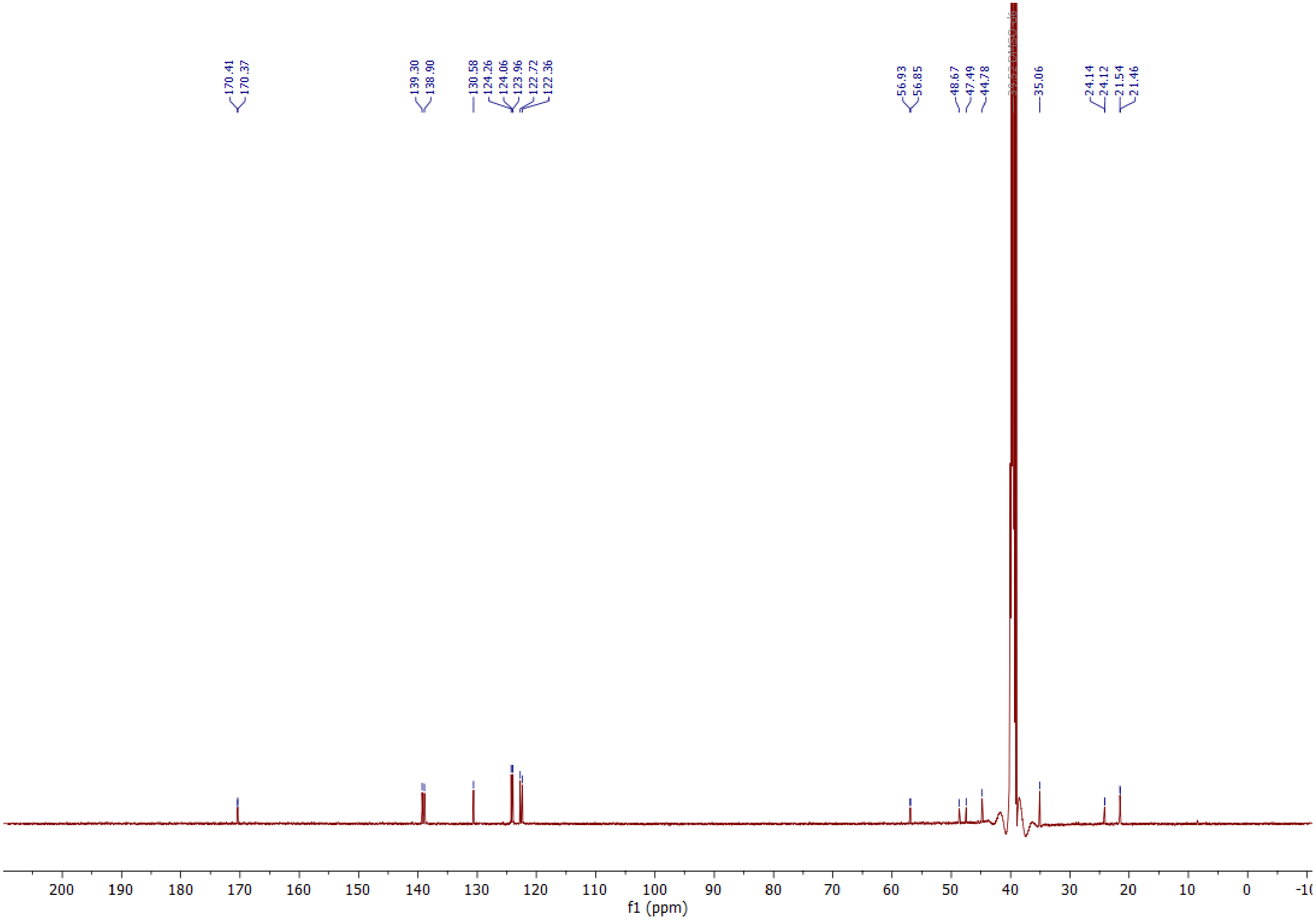

Figure S151. ^{13}C NMR spectrum of **5l** diastereomer 2.

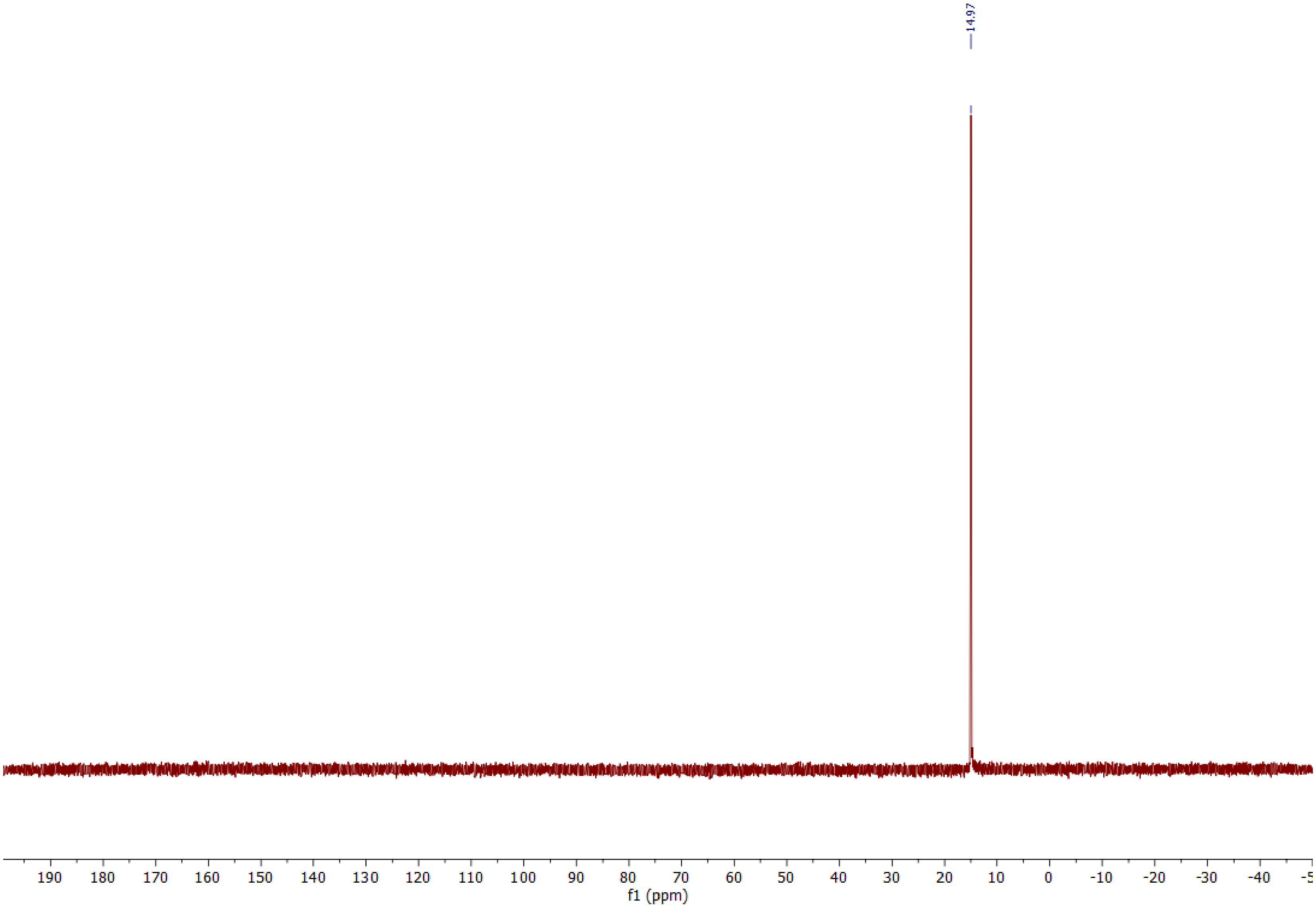

Figure S152. ^{31}P NMR spectrum of **5l** diastereomer 2.

5. Crystallographic data

4a-2 (*R*,*R* / *S*,*S*)

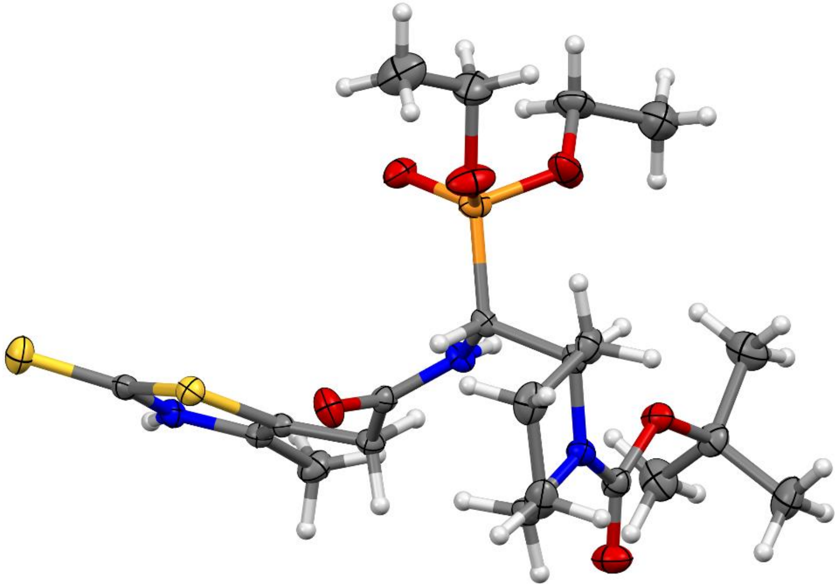

Figure S153. Thermal ellipsoid diagram (50% probability) of **4a-2** (*R*,*R* presented), CCDC 2203659.

Chemical Formula: $C_{21}H_{36}N_3O_6PS_2$

Formula weight: 521.62

Crystal system: monoclinic

Space Group: $P2_1/n$

Cell lengths / Å: **a** 11.8864(6) **b** 17.0353(6) **c** 13.0083(5)

Cell angles / deg: **α** 90 **β** 93.201(4) **γ** 90

Cell Volume / Å^3: 2629.92

R indices [I>2σ(I)]: R_1 = 0.0597, wR_2 = 0.1056

R indices (all data): R_1 = 0.0989, wR_2 = 0.1199

Goodness-of-fit on F^2: 1.052

Z, Z': **Z**: 4 **Z'**: 1

T / K: 120

Dcalc / g cm^{-3}: 1.317

Packing coefficient: 0.665897

Radiation type: micro-focus sealed X-ray tube, Mo-radiation

Habit: prism

Color: colorless

4b-1 (*R*,*S* / *S*,*R*)

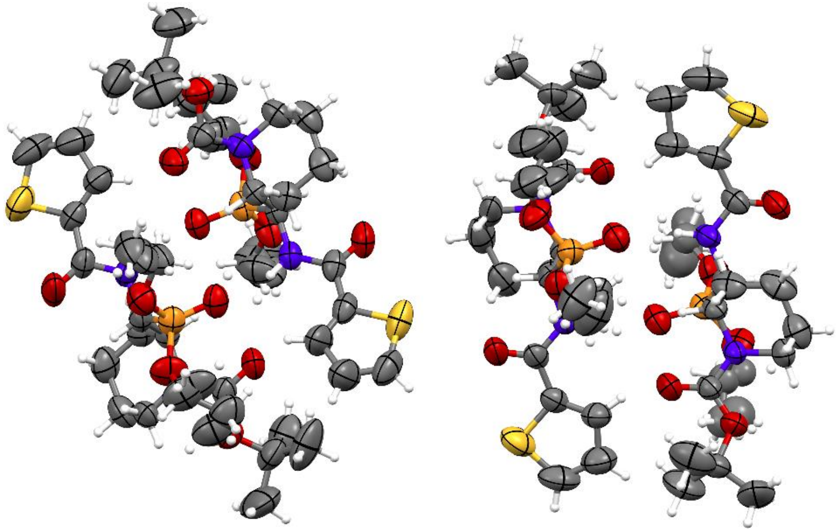

Figure S154. Thermal ellipsoid diagram (50% probability) of **4b-2** (asymmetric unit presented), CCDC 2203660.

Chemical Formula: $C_{20}H_{33}N_2O_6PS$

Formula weight: 460.51

Crystal system: triclinic

Space Group: $P\bar{1}$

Cell lengths / Å: **a** 15.5581(5) **b** 16.4388(5) **c** 19.9240(5)

Cell angles / deg: **α** 94.181(2) **β** 98.983(2) **γ** 90.241(2)

Cell Volume / Å^3: 5019.17

R indices [$I>2\sigma(I)$]: $R_1 = 0.0680$, w$R_2 = 0.1662$

R indices (all data): $R_1 = 0.1386$, w$R_2 = 0.2105$

Goodness-of-fit on F^2: 1.022

Z, Z': **Z**: 8 **Z'**: 4

T / K: 298

Dcalc / g cm^{-3}: 1.219

Packing coefficient: 0.644038

Radiation type: micro-focus sealed X-ray tube, Mo-radiation

Habit: block

Color: colorless

4c-2 (*R*,*R* / *S*,*S*)

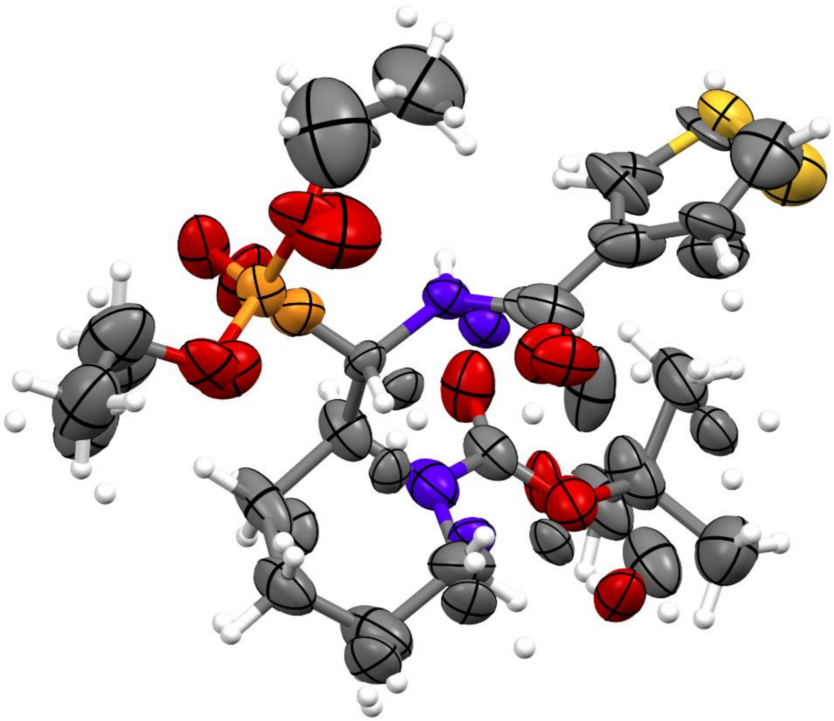

Figure S155. Thermal ellipsoid diagram (50% probability) of **4c-2** (*S*,*S* presented), CCDC 2203661.

Chemical Formula: $C_{20}H_{33}N_2O_6PS$

Formula weight: 460.51

Crystal system: monoclinic

Space Group: $P2_1/n$

Cell lengths / Å: **a** 12.7772(2) **b** 13.5168(2) **c** 13.6871(2)

Cell angles / deg: **α** 90 **β** 94.1990(10) **γ** 90

Cell Volume / Å^3: 2357.51

R indices [$I>2\sigma(I)$]: R_1 = 0.0780, wR_2 = 0.2175

R indices (all data): R_1 = 0.0846, wR_2 = 0.2255

Goodness-of-fit on F^2: 1.048

Z, Z’: **Z**: 4 **Z'**: 1

T / K: 120

Dcalc / g cm^{-3}: 1.297

Packing coefficient: 0.788776

Radiation type: micro-focus sealed X-ray tube, Cu-radiation

Habit: prism

Color: colorless

4d-2 (*R*,*R* / *S*,*S*)

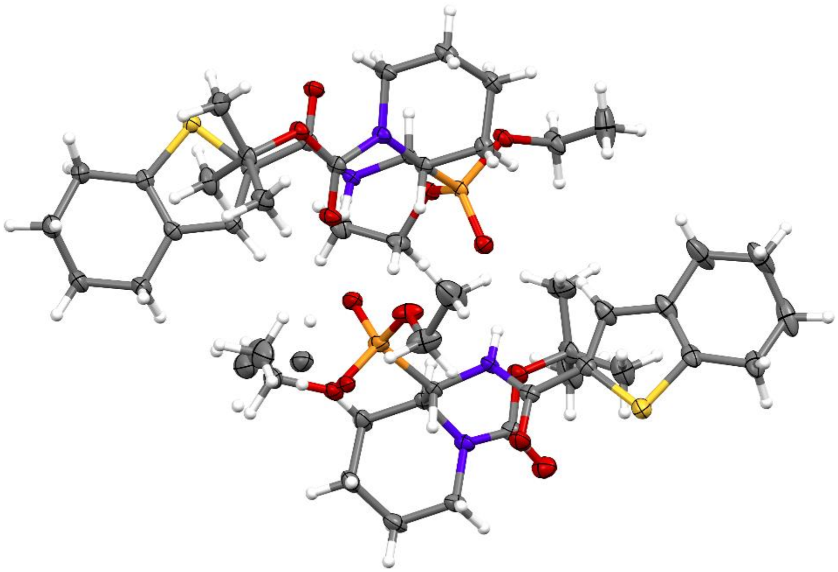

Figure S156. Thermal ellipsoid diagram (50% probability) of **4d-2** (asymmetric unit presented), CCDC 2203662.

Chemical Formula: $C_{24}H_{39}N_2O_6PS$

Formula weight: 514.60

Crystal system: monoclinic

Space Group: $P2_1/c$

Cell lengths / Å: **a** 18.2265(2) **b** 14.9162(2) **c** 19.3281(2)

Cell angles / deg: **α** 90 **β** 91.2810(10) **γ** 90

Cell Volume / Å^3: 5253.42

R indices [I>2σ(I)]: R_1 = 0.0447, wR_2 = 0.1110

R indices (all data): R_1 = 0.0537, wR_2 = 0.1191

Goodness-of-fit on F^2: 1.029

Z, *Z'*: **Z**: 8 **Z'**: 2

T / K: 120

Dcalc / g cm^{-3}: 1.301

Packing coefficient: 0.686541

Radiation type: micro-focus sealed X-ray tube, Cu-radiation

Habit: needle

Color: colorless

4e-2 (*R*,*R* / *S*,*S*)*

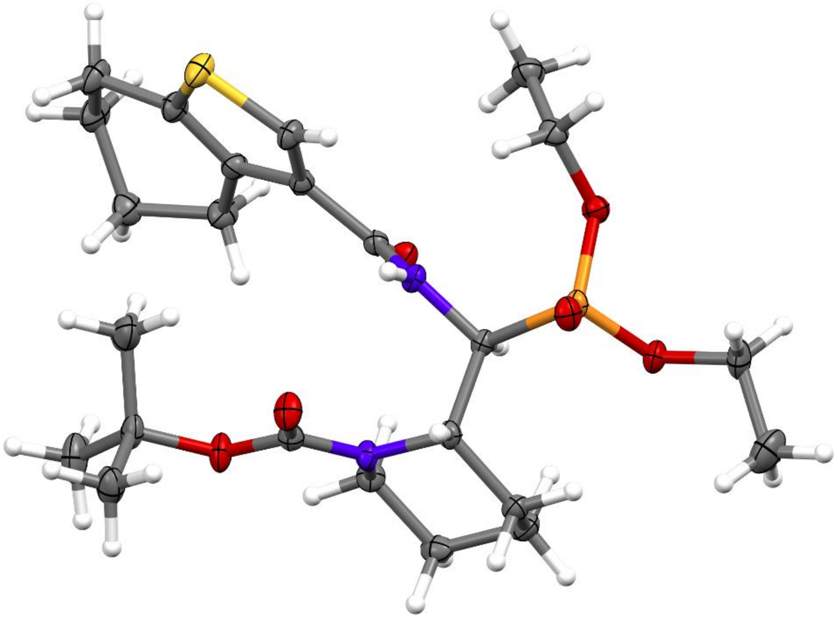

Figure S157. Thermal ellipsoid diagram (50% probability) of **4e-2** (*S*,*S* presented), CCDC 2203663.

Chemical Formula: $C_{24}H_{39}N_2O_6PS$

Formula weight: 514.60

Crystal system: triclinic

Space Group: $P\bar{1}$

Cell lengths / Å: **a** 9.9148(6) **b** 10.9400(6) **c** 14.0426(7)

Cell angles / deg: **α** 83.162(4) **β** 71.761(5) **γ** 64.421(6)

Cell Volume / Å^3: 1304.63

R indices [$I>2\sigma(I)$]: R_1 =0.0416 , wR_2 = 0.0943

R indices (all data): R_1 = 0.0533, wR_2 = 0.1032

Goodness-of-fit on F^2: 1.040

Z, Z': **Z**: 2 **Z'**: 1

T / K: 120

Dcalc / g cm^{-3}: 1.310

Packing coefficient: 0.681854

Radiation type: micro-focus sealed X-ray tube, Mo-radiation

Habit: block

Color: colorless

*Alternative monoclinic structure **4e-2b** was also obtained. Data is found from CSD with identifier CCDC 2203670.

4f-1 (*R*,*S* / *S*,*R*)

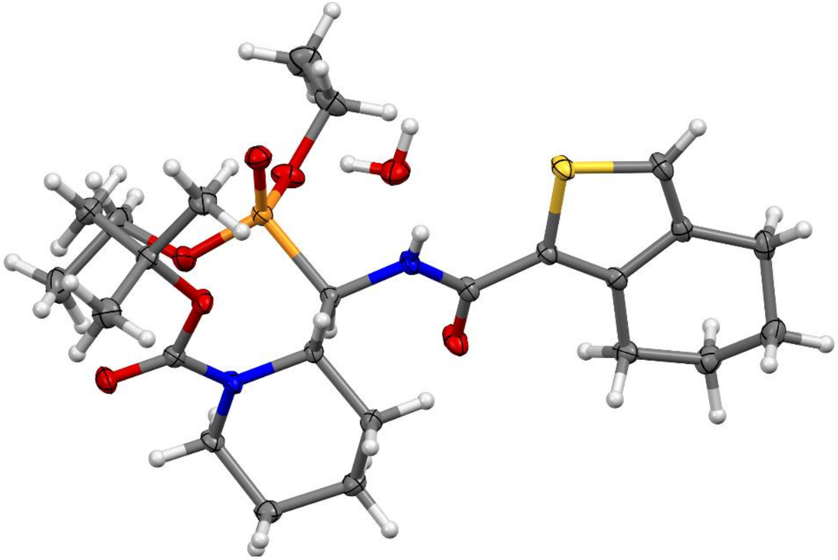

Figure S158. Thermal ellipsoid diagram (50% probability) of **4f-1** (*R*,*S* presented), CCDC 2203664.

Chemical Formula: $C_{24}H_{39}N_2O_6PS$, H_2O

Formula weight: 532.62

Crystal system: triclinic

Space Group: $P\bar{1}$

Cell lengths / Å: **a** 8.9060(3) **b** 12.7308(4) **c** 13.1186(4)

Cell angles / deg: **α** 75.251(13) **β** 76.379(3) **γ** 73.533(3)

Cell Volume / $Å^3$: 1346.58

R indices [$I>2\sigma(I)$]: R_1 = 0.0330, wR_2 =0.0819

R indices (all data): R_1 = 0.0390, wR_2 = 0.0860

Goodness-of-fit on F^2: 1.024

Z, Z’: **Z**: 2 **Z'**: 1

T / K: 120

Dcalc / g cm^{-3}: 1.314

Packing coefficient: 0.688268

Radiation type: micro-focus sealed X-ray tube, Mo-radiation

Habit: block

Color: colorless

4g-1 (*R*,*S* / *S*,*R*)

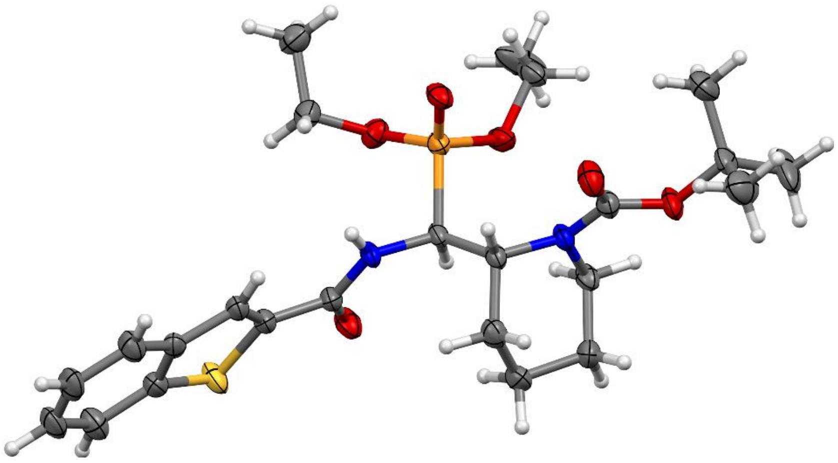

Figure S159. Thermal ellipsoid diagram (50% probability) of **4g-1** (*S*,*R* presented), CCDC 2203665.

Chemical Formula: $C_{24}H_{35}N_2O_6PS$

Formula weight: 510.57

Crystal system: monoclinic

Space Group: $P2_1/n$

Cell lengths / Å: **a** 10.2580(5) **b** 17.1642(5) **c** 14.6705(4)

Cell angles / deg: **α** 90 **β** 90.482(3) **γ** 90

Cell Volume / $Å^3$: 2582.95(16)

R indices [$I>2\sigma(I)$]: R_1 = 0.0782, wR_2 = 0.2331

R indices (all data): R_1 = 0.1065, wR_2 = 0.2910

Goodness-of-fit on F^2: 1.107

Z, Z': **Z**: 4 **Z'**: 1

T / K: 120

Dcalc / g cm^{-3}: 1.313

Packing coefficient: 0.679441

Radiation type: micro-focus sealed X-ray tube, Mo-radiation

Habit: prism

Color: colorless

4h-2 (*R*,*R* / *S*,*S*)

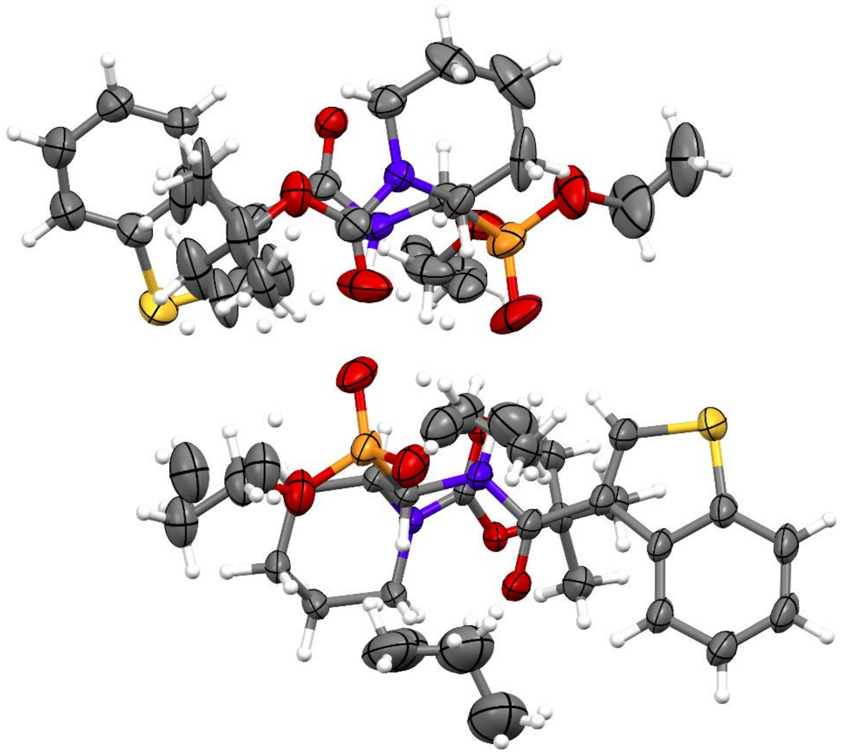

Figure S160. Thermal ellipsoid diagram (50% probability) of **4h-2** (asymmetric unit presented), CCDC 2203666.

Chemical Formula: 2($C_{24}H_{35}N_2O_6PS$), 0.5C_6H_{12})

Formula weight: 1063.21

Crystal system: monoclinic

Space Group: *C*2/*c*

Cell lengths / Å: **a** 48.4808(11) **b** 12.1659(6) **c** 18.9571(5)

Cell angles / deg: **α** 90 **β** 96.648(2) **γ** 90

Cell Volume / $Å^3$: 11106

R indices [$I>2\sigma(I)$]: R_1 = 0.0687, wR_2 = 0.1777

R indices (all data): R_1 = 0.1001, wR_2 = 0.2005

Goodness-of-fit on F^2: 1.031

Z, Z’: **Z**: 8 **Z'**: 1

T / K: 120

Dcalc / g cm^{-3}: 1.272

Packing coefficient: 0.696741

Radiation type: micro-focus sealed X-ray tube, Mo-radiation

Habit: prism

Color: colorless

4i-1 (*S*,*R* / *R*,*S*)

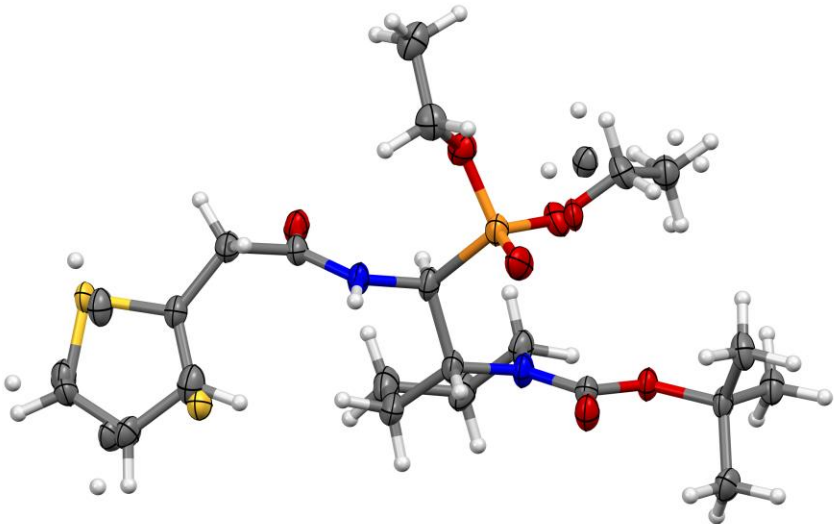

Figure S161. Thermal ellipsoid diagram (50% probability) of **4i-1** (asymmetric unit presented), CCDC 2209101.

Chemical Formula: $C_{21}H_{35}N_2O_6PS$

Formula weight: 474.55

Crystal system: triclinic

Space Group: $P\bar{1}$

Cell lengths / Å: **a** 9.2861(6) **b** 11.2061(9) **c** 12.3620(6)

Cell angles / deg: **α** 103.022(5) **β** 100.213(5) **γ** 102.017(6)

Cell Volume / $Å^3$: 1191.44

R indices [$I>2\sigma(I)$]: R_1 = 0.0702, wR_2 = 0.1820

R indices (all data): R_1 = 0.0915, wR_2 = 0.2123

Goodness-of-fit on F^2: 1.050

Z, Z': **Z**: 2 **Z'**: 1

T / K: 120

Dcalc / g cm^{-3}: 1.323

Packing coefficient: 0.715794

Radiation type: micro-focus sealed X-ray tube, Mo-radiation

Habit: stacked plate

Color: colorless

4j-1 (*R*,*S* / *S*,*R*)

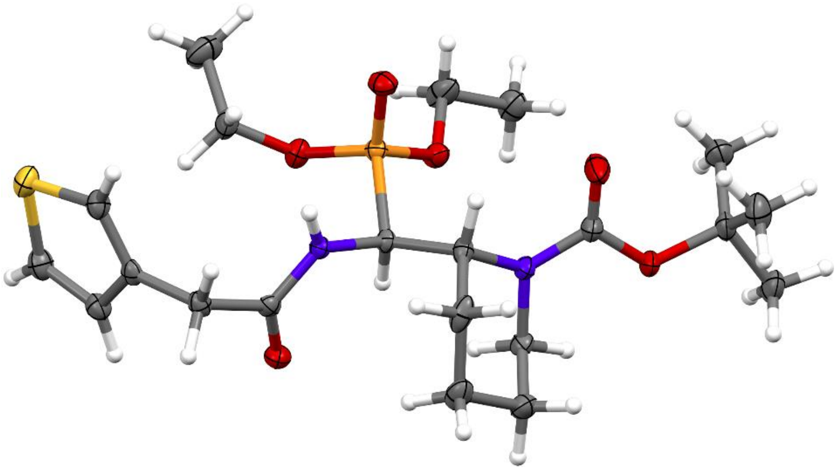

Figure S162. Thermal ellipsoid diagram (50% probability) of **4j-1** (*S*,*R* presented), CCDC 2203667.

Chemical Formula: $C_{21}H_{35}N_2O_6PS$

Formula weight: 474.54

Crystal system: triclinic

Space Group: $P\bar{1}$

Cell lengths / Å: **a** 8.8287(4) **b** 10.6334(6) **c** 14.2157(7)

Cell angles / deg: **α** 109.022(5) **β** 90.103(4) **γ** 99.548(4)

Cell Volume / Å^3: 1241.97

R indices [I>2σ(I)]: R_1 = 0.0431, wR_2 = 0.0934

R indices (all data): R_1 = 0.0603, wR_2 = 0.1023

Goodness-of-fit on F^2: 1.024

Z, *Z'*: **Z**: 2 **Z'**: 1

T / K: 120

Dcalc / g cm^{-3}: 1.269

Packing coefficient: 0.660928

Radiation type: micro-focus sealed X-ray tube, Mo-radiation

Habit: prism

Color: colorless

4k-1 (*R*,*S* / *S*,*R*)

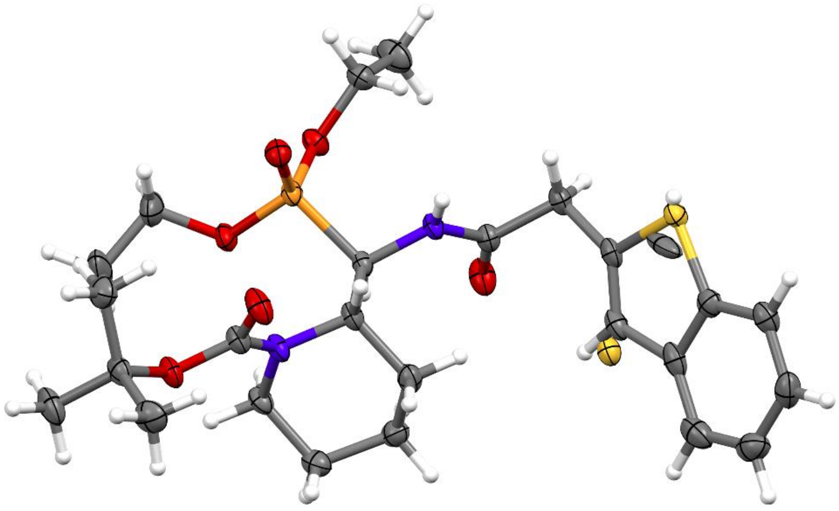

Figure S163. Thermal ellipsoid diagram (50% probability) of **4k-1** (*R*,*S* presented), CCDC 2203668.

Chemical Formula: $C_{25}H_{37}N_2O_6PS$

Formula weight: 524.59

Crystal system: triclinic

Space Group: $P\bar{1}$

Cell lengths / Å: **a** 9.0460(3) **b** 10.9706(4) **c** 13.8583(3)

Cell angles / deg: **α** 86.282(2) **β** 87.202(2) **γ** 79.542(3)

Cell Volume / Å^3: 1348.64

R indices [$I>2\sigma(I)$]: R_1 = 0.0417, wR_2 = 0.1033

R indices (all data): R_1 =0.0519 , wR_2 = 0.1105

Goodness-of-fit on F^2:Z, Z': **Z**: 2 **Z'**: 1

T / K: 120

Dcalc / g cm^{-3}: 1.292

Packing coefficient: 0.675088

Radiation type: micro-focus sealed X-ray tube, Mo-radiation

Habit: block

Color: colorless

4l-1 (*R*,*S* / *S*,*R*)

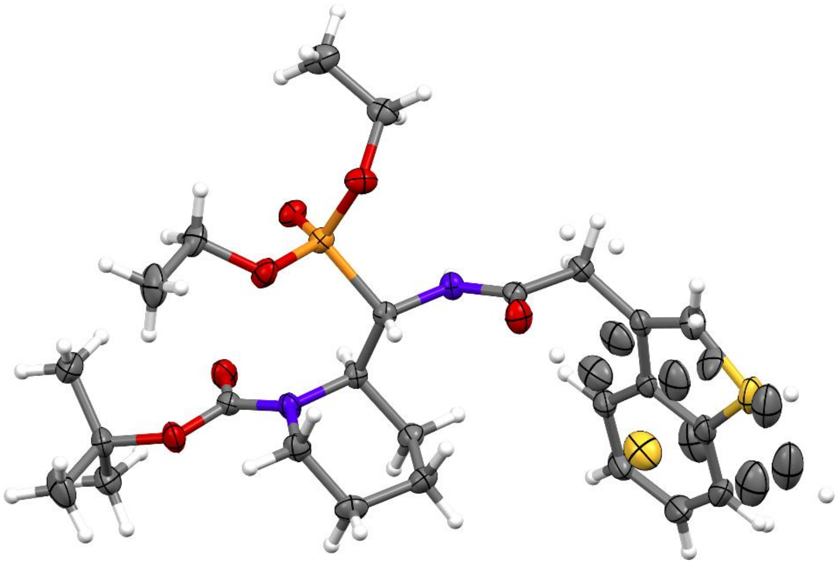

Figure S164. Thermal ellipsoid diagram (50% probability) of **41-1** (S,*R* presented), CCDC 2203669.

Chemical Formula: $C_{25}H_{37}N_2O_6PS$

Formula weight: 524.59

Crystal system: monoclinic

Space Group: $P2_1/c$

Cell lengths / Å: **a** 9.3831(3) **b** 26.8231(6) **c** 11.5306(3)

Cell angles / deg: **α** 90 **β** 107.972(3) **γ** 90

Cell Volume / Å^3: 2760.47

R indices [I>2σ(I)]: R_1 =0.0464 , wR_2 = 0.1011

R indices (all data): R_1 = 0.0632, wR_2 = 0.1110

Goodness-of-fit on F^2: 1.061

Z, *Z*’: **Z**: 4 **Z'**: 1

T / K: 120

Dcalc / g cm^{-3}: 1.262

Packing coefficient: 0.691595

Radiation type: micro-focus sealed X-ray tube, Mo-radiation

Habit: prism

Color: colorless

6. Chemical shift perturbation (CSP) throughout the titration of NDM-1 and of VIM-2 with inhibitors

Table S3. Chemical shift changes for the titrated inhibitors with NDM-1

**AA NDM-1**	**5i-2**	**5i-1**	**5b-1**	**5c-1**	**5j-1**
039M_113V	0,00328	0,00147	0,00691	0,00127	0,00116
042G	0,01175	0,01218	**0,03491**	**0,02388**	0,01481
043D	0,00106	0,00183	2,02724E-4	0,00273	0,00299
044Q	0,00296	6,99625E-4	0,00729	0,00129	0,00101
045R_099A	0,01151	0,00688	0,00895	0,00605	0,00342
046F	0,00398	0,00177	0,00691	0,0033	4,05921E-4
047G_084G	0,01881	0,00816	0,02125	0,00624	0,01625
048D	0,00434	0,00284	0,01143	0,0041	0,00858
049L	0,01065	0,00708	0,00753	0,00171	0,0086
050V	0,0113	0,0152	0,01529	0,0087	0,00998
051F	0,00411	0,00422	0,0085	0,00383	0,00817
052R	0,01034	0,00432	0,00282	0,00385	0,00813
053Q	0,00752	0,00664	2,49956E-4	0,00397	0,00456
054L_183F	0,00719	0,00877	9,81603E-4	0,00113	0,00651

055A	0,01371	8,41347E-5	0,00744	0,00801	0,00691
057N	0,0016	0,00503	0,00692	0,00593	0,00275
058V	0,00583	9,63014E-5	0,0016	0,00118	0,00687
059W	0,00689	0,00165	4,35102E-4	0,00148	0,00328
060Q	0,00297	0,00388	0,00277	0,00284	0,00569
061H	0,00715	0,00326	1,8125E-4	0,00305	0,00206
062T_158Q	0,00318	0,01713	0,00306	0,00562	0,00633
063S	0,0028	0,00147	0,01107	0,00676	0,0079
064Y	0,00543	0,00204	0,0015	0,00157	0,00194
065L	0,01085	0,00947	0,0062	0,00687	0,01444
066D	0,0038	0,0034	0,003	0,00159	0,00136
067M_245M	0,00388	0,00178	0,00198	0,00115	2,14963E-4
070F	**0,02197**	**0,02939**	**0,03529**	**0,02712**	**0,04048**
071G	0,0035	0,00165	0,00687	0,00119	6,96313E-4
072A	0,00325	0,00162	3,7803E-4	0,00115	6,87315E-4
073V	0,00133	0,00822	9,07252E-4	0,00585	0,00222
074A	0,01125	0,01409	0,00714	0,01269	0,01373
075S	0,01338	0,0175	0,01654	0,01628	0,01287
076N_114A	0,00625	0,01077	0,0046	0,00638	0,00708

077G	0,01041	0,00884	0,01209	0,00521	0,0088
078L	0,00417	0,00821	0,00721	0,0014	0,00736
079I	0,00585	8,03244E-4	0,00131	0,00708	0,00132
080V	0,00503	0,00181	0,00115	0,0035	0,00121
081R	0,00587	0,005	0,00351	0,00413	0,00728
082D	0,00712	0,00733	0,00778	0,00338	0,00884
083G	0,00994	0,00108	0,0042	0,00686	0,01515
085R	0,00321	0,00701	2,64053E-4	0,00574	0,00794
086V	0,00352	8,02338E-4	0,00733	0,0024	0,00749
087L	0,00617	0,00224	0,00209	0,00241	0,00138
088V	0,00717	4,77773E-4	3,64557E-4	0,00167	0,00641
089V	0,00444	0,00241	0,00185	0,0064	0,00164
090D	0,00783	0,0096	0,00762	0,00583	0,00856
091T	0,0193	0,02013	**0,02268**	**0,02061**	**0,02246**
092A	0,00691	0,00686	0,01382	0,01262	0,01371
093W	0,004	0,0016	9,22173E-4	0,00593	3,80916E-4
094T	0,0139	0,01372	0,01062	0,01048	0,01036
095D	0,01334	**0,02087**	0,00385	**0,02072**	0,00863
096D	0,00664	0,00824	0,00944	0,00675	0,0078

097Q	0,00685	0,00846	0,00561	0,00489	0,00983
098T	0,00491	0,00113	5,61495E-4	0,00604	0,00332
100Q	0,00297	3,70412E-4	0,00165	0,00582	5,47039E-4
101I	0,00422	0,00464	0,00391	0,00719	0,00455
102L	0,00331	9,63959E-4	0,00741	0,0016	0,00275
103N	0,00909	0,00826	0,00843	0,00705	0,01047
104W	0,00799	0,00266	0,00788	0,00332	0,00608
105I	0,00433	0,00695	0,00315	0,00252	0,00347
106K_238A	0,00706	0,00198	2,92216E-4	0,00577	0,00142
107Q	0,00405	0,00295	0,00717	0,00273	0,00201
108E	**0,03552**	0,01038	**0,03425**	**0,03377**	**0,02934**
109I_146N	0,0049	0,00764	0,00183	0,00258	0,00686
110N_176N	0,00394	3,65623E-4	0,00219	0,00182	0,00145
111L	0,003	0,00253	0,00687	0,0016	1,3149E-4
115L	0,00309	0,00136	0,00689	0,00141	5,52205E-6
116A	0,00341	8,78262E-4	0,00707	0,00511	0,00373
117V	0,00706	0,00573	0,01078	0,004	0,00866
118V	0,00651	0,01332	0,00689	0,00812	0,00233
119T	**0,03146**	**0,02807**	**0,02832**	**0,02659**	**0,03443**

120H	0,00809	0,00803	0,0067	0,00856	0,01036
121A	0,01131	0,01038	0,01806	0,00913	0,00868
122H	0,01753	0,01469	0,01569	0,01413	0,02164
123Q	0,0212	0,01577	0,01861	**0,02677**	0,02181
124D	**0,03672**	**0,03582**	**0,03533**	**0,03601**	**0,03832**
127G	**0,0265**	**0,02746**	**0,02444**	**0,02353**	**0,02973**
128G	0,01091	0,01967	0,01989	0,01876	0,0183
129M	0,00645	0,00645	0,00883	0,00781	0,0094
130D	0,00472	0,00686	0,00348	0,00636	0,00481
131A_168W	0,00342	7,29518E-4	0,00708	0,00113	0,00796
132L	0,00248	4,18024E-4	0,00692	0,00594	0,00721
133H_225D	0,01828	0,01852	0,02033	0,0141	**0,0233**
134A	0,00542	0,00268	0,0047	0,00661	0,00493
135A	0,00313	0,00686	5,73616E-4	0,00117	0,00697
136G	0,00333	0,00695	0,00298	0,00218	0,00334
137I	0,00699	0,0078	0,00387	0,00905	0,00393
138A	0,00359	0,00702	0,00709	0,00845	0,00686
139T	0,02004	0,01809	0,01761	0,01445	0,01646
140Y	0,00661	0,00821	0,00265	0,00725	0,0069

141A	0,01046	0,00559	0,01394	0,01435	0,00859
142N	0,01907	0,01618	0,0181	0,01374	0,01587
143A	0,01866	0,01812	0,02239	**0,02124**	0,01999
144L	0,00956	0,00464	0,00617	0,0093	0,0055
145S	0,00781	0,00697	0,00825	0,00684	0,002
147Q	0,01451	0,0147	0,01494	0,01497	0,01637
148L	0,01045	0,00689	0,00704	0,00801	0,00703
149A	0,02005	0,01582	**0,02277**	**0,02485**	**0,02404**
151Q	0,01425	0,01297	0,01119	0,01182	0,01314
152E	**0,02471**	**0,02452**	**0,02827**	**0,02585**	**0,02502**
153G	0,02147	0,01508	0,01865	0,01671	**0,02448**
154M	0,01768	0,01557	0,02038	0,0104	0,01602
155V	0,0115	0,00972	0,00837	0,00701	0,00792
156A	0,00754	0,01408	0,01387	0,01264	0,00733
157A	0,00306	0,00697	6,22152E-4	0,00113	3,58282E-4
159H	**0,03966**	**0,03557**	**0,03734**	**0,03077**	**0,03724**
160S	**0,03221**	**0,02465**	**0,02633**	**0,02219**	**0,04038**
161L	0,01124	0,00734	0,00784	0,00598	0,00813
162T	0,006	0,00473	0,00823	0,00868	0,00781

163F	0,00338	0,00188	0,00695	0,00158	5,30436E-4
164A	0,00393	0,00686	0,00732	0,0059	0,00616
166N	0,0035	0,00163	8,36377E-4	0,00599	2,56897E-4
167G	1,13827E-4	0,00321	0,0016	0,0034	0,00332
169V	0,00421	0,00375	0,00538	0,00572	0,00575
170E	0,00383	0,008	0,00434	0,00164	0,00103
172A	0,00791	0,00807	0,00818	0,00119	0,01936
173T	0,00754	0,00688	0,00297	0,00307	0,00326
174A	0,01084	0,01031	0,00895	0,00895	0,01023
177F	0,00741	0,00192	0,0021	0,00181	0,00686
178G	0,00315	0,00702	7,51766E-4	0,00595	3,50574E-4
180L	0,01137	0,00752	0,00711	0,01297	0,014
181K	0,0043	0,00829	7,52211E-4	0,00583	0,0069
182V	0,00428	0,00694	0,00708	0,008	0,01392
184Y	0,00849	0,00823	0,00976	0,00994	0,00558
188G	0,01501	**0,02383**	**0,02301**	**0,02183**	**0,02398**
189H	0,0108	0,00688	0,00351	0,00178	0,00894
190T	0,01569	0,01592	0,00984	0,01364	0,00842
191S	**0,0252**	**0,03196**	**0,03868**	**0,03392**	0,01971

192D	0,01537	0,01504	0,01569	0,01031	0,018
193N	0,00959	0,01039	0,00888	0,00802	0,01123
194I	**0,05979**	**0,05492**	**0,05714**	**0,05002**	**0,05633**
195T	0,01398	0,01384	0,01534	0,00799	0,01054
196V	0,01667	0,01372	0,02059	0,01484	0,02111
197G	**0,02697**	**0,02382**	**0,02395**	**0,0229**	**0,02681**
198I	0,01394	0,00349	0,00694	0,00228	0,00747
199D	0,00869	0,0095	0,00684	0,00722	0,00574
201T	0,00557	0,00236	0,00163	0,00577	1,01632E-4
202D	0,00334	0,00776	0,00698	0,00451	0,00435
203I	0,00616	0,00526	0,00711	0,00656	0,0052
204A	0,00392	6,17058E-4	0,00713	0,00574	0,00702
205F	**0,02357**	**0,02064**	**0,02773**	**0,02199**	**0,02785**
206G	**0,02548**	0,01797	0,02219	**0,02095**	**0,02288**
209L	0,00298	7,10977E-4	3,0759E-4	0,0063	0,00233
210I	0,00688	0,00776	0,0117	0,01494	0,00719
211K	**0,03075**	**0,02095**	**0,02843**	**0,02653**	**0,02112**
212D	0,00724	0,01425	0,0142	0,01259	0,01429
213S	0,01422	**0,02152**	0,02129	**0,0195**	**0,02154**

214K	**0,04444**	**0,04873**	**0,04233**	**0,03652**	**0,0268**
215A	0,0072	0,0015	0,00742	0,00172	0,00765
217S	**0,04884**	**0,03748**	**0,04157**	**0,03443**	**0,03916**
222G	0,00396	0,00746	0,01371	0,01261	0,00686
223D	0,00976	0,00686	0,00146	0,00799	0,00692
224A	0,01692	0,01554	0,01766	0,01696	0,01793
226T	0,01446	0,01268	0,01714	0,01604	0,01646
227E	0,02059	0,01391	0,0213	0,01536	0,01403
228H	**0,0539**	**0,0555**	**0,06221**	**0,04304**	**0,06244**
229Y	**0,0293**	**0,02733**	**0,03299**	**0,0256**	**0,03282**
230A	**0,02392**	**0,02615**	**0,02342**	**0,02284**	**0,02361**
231A	0,00854	0,00965	0,00896	0,00809	0,0081
232S	**0,02671**	**0,02974**	**0,02898**	**0,02208**	**0,03321**
233A	0,01813	0,01527	0,01803	0,01338	0,01975
234R	0,01152	0,01227	0,0107	0,00915	0,0118
235A	0,00452	0,0078	0,00451	0,00185	0,00958
236F_265M	0,00571	0,0034	0,00886	0,00798	0,00636
237G	0,00777	0,00788	0,00547	0,00244	0,00412
239A	1,554E-4	0,00689	0,00686	0,00142	2,89366E-4

240F	0,00686	0,00153	0,00688	0,00798	0,00321
242K	0,00769	0,00889	0,0026	0,01289	0,00437
243A	0,00905	0,00753	0,00748	0,00776	0,0065
244S	0,0087	0,00905	0,01437	0,00678	0,01601
246I	0,01331	0,01208	0,02109	0,01194	0,0197
247V	0,01598	0,0163	0,01566	0,01381	0,01559
248M	**0,02787**	**0,02319**	**0,03054**	**0,02324**	**0,026**
249S	**0,02416**	**0,0262**	0,02216	0,01925	**0,02555**
251S	**0,0328**	**0,03069**	**0,03024**	**0,03136**	**0,03107**
252A	0,02116	**0,02469**	**0,03138**	0,01771	0,02062
254D	**0,04253**	**0,03809**	**0,04566**	**0,03534**	**0,04751**
255S	**0,02905**	**0,02557**	**0,02997**	**0,02434**	**0,02898**
256R	0,00886	0,01069	0,00989	0,01557	0,01048
258A	0,00705	0,00355	0,00827	0,00739	0,00814
259I	0,00805	0,00712	0,01024	0,01637	0,01508
260T	0,01175	0,01113	0,00903	0,01124	0,01026
261H	0,01742	**0,02043**	0,01659	0,01102	0,01801
262T	0,00599	0,0077	0,00707	0,00326	7,0326E-4
263A_266A	**0,02201**	0,01094	0,01421	0,01234	0,01709

264R	0,01216	0,01115	0,01314	0,00972	0,01098
267D	0,00708	0,0088	0,00907	0,00818	0,00829
268K	0,00544	0,00107	0,00508	0,00812	0,00564
269L	0,00421	0,00146	0,00468	0,00134	0,00687
270R	0,00334	0,00357	0,00692	0,00118	0,00724

Table S4. Chemical shift changes for the titrated inhibitors withVIM-2.

**AA VIM-2**	**5i-2**	**5b-2**	**5b-1**	**5c-1**	**5a-1**	**5c-2**	**5j-1**
026D	0,002973	0,002374	0,004266	0,01009	0,00301	0,00742	0,00155
027S	0,004049	0,005151	0,008728	0,014481	0,014824	0,005142	0,005841
028S	0,003402	0,005551	0,004507	0,013653	0,012943	0,015541	0,002415
029G	0,00475	0,005688	0,002988	0,006731	0,006704	0,015704	0,006573
030E	0,009145	0,020563	0,011619	0,004271	0,005114	0,009965	0,005876
031Y	**0,040506**	0,081188	**0,069448**	**0,074381**	0,056682	0,075268	0,024127
033T	**0,067571**	**0,095838**	**0,109287**	**0,142233**	**0,126043**	**0,111111**	**0,058233**
034V	0,033483	**0,09828**	**0,07954**	0,066596	0,019325	0,073338	0,024558
035S	0,00271	0,011615	0,002821	0,001	0,005156	0,006257	0,007004
036E	0,007527	0,030459	0,009571	0,02107	0,034886	0,018545	0,023578
037I	0,033192	0,072923	**0,058786**	**0,07622**	0,047687	**0,096537**	0,029601
041E	0,004106	0,004375	0,007019	0,013908	0,024781	0,012408	0,013606
042V	0,010957	0,013137	0,030407	0,018234	0,040356	0,039208	0,0195
043R	0,012244	0,055998	**0,051314**	0,067691	0,034865	0,044237	0,007352
044L	**0,043284**	**0,111383**	**0,08678**	**0,069636**	**0,062277**	**0,092986**	**0,036377**
045Y	**0,05605**	**0,117025**	**0,070109**	0,061274	0,02935	0,091104	0,033778
046Q	0,017104	0,022354	0,016147	0,013555	0,012872	0,022986	0,007646

047I	0,012806	0,033795	0,019364	0,028574	0,020151	0,027131	0,009946
048A	0,001135	0,010103	0,01168	0,014061	0,016183	0,009876	0,002939
049D	0,010628	0,015544	0,013669	0,012848	0,001993	0,005009	0,004762
050G	0,006207	0,012803	0,011394	0,008705	0,002893	0,005555	0,001639
051V	0,010953	0,012208	0,013288	0,021531	0,012241	0,015713	0,012669
052W	0,006824	0,019829	0,015853	0,016153	0,005072	0,011253	0,008898
053S	0,022513	0,032606	0,026447	0,016766	0,012157	0,020938	0,007511
054H	0,014652	0,039484	0,031504	0,019595	0,023802	0,027867	0,01185
055I	0,006423	0,050126	0,013109	0,010743	0,027903	0,041117	0,009458
056A	0,014644	0,018883	0,008659	0,067368	0,04363	0,081645	0,018093
062G	**0,06996**	**0,129042**	**0,111774**	**0,119491**	**0,077834**	**0,129824**	**0,060616**
063A	0,028186	0,084147	**0,054143**	**0,070824**	0,052148	**0,094945**	0,026509
064V	**0,057641**	0,077233	**0,081178**	**0,104408**		0,082165	**0,06919**
065Y		**0,145667**		**0,164978**	**0,152405**	**0,171454**	**0,08554**
069G		**0,255943**					
071I	0,018772	0,046539	0,013623	0,01462	0,00268	0,047132	0,010154
072V	0,000703	0,012913	0,004175	0,004809	0,006845	0,002106	0,005796
073R	0,002822	0,001208	0,009626	0,005984	0,005368	0,003668	0,004682
074D	0,003871	0,002357	0,011285	0,013262	0,011314	0,002137	0,010393

075G	0,004719	0,004884	0,006427	0,004385	0,002809	0,007426	0,00591
076D	0,008625	0,008413	0,00712	0,019334	0,025974	0,019896	0,012086
077E	0,002748	0,003303	0,00438	0,010961	0,014751	0,015577	0,001848
078L	0,006615	0,004408	0,006527	0,006005	0,010003	0,005957	0,002621
079L	0,01641	0,024431	0,006254	0,014798	0,015587	0,035433	0,013243
080L	0,031617	0,063568	0,022898	0,027555	0,017361	0,073234	0,014174
081I	0,030389	0,049649	0,019983	0,028531	0,009345	0,060695	0,018425
082D	0,013483	0,06851	0,010901	0,012911	0,012136	0,062022	0,006376
083T		0,089636	**0,053851**	0,043977	0,014967	**0,094755**	0,016341
095A	0,029149	0,037568	0,020229	0,015283	0,030284	0,028614	0,026282
096E	0,024452	0,053129	0,030945	0,031179	0,028841	0,050627	0,009891
097I	0,018059	0,044402	0,025656	0,019599	0,009892	0,026088	0,006076
098E	0,008194	0,0185	0,010051	0,019158	0,024451	0,026022	0,010781
099K	0,011036	0,021349	0,008088	0,009804	0,00775	0,022953	0,009632
100Q	0,008815	0,030649	0,014094	0,009482	0,009278	0,013672	0,004512
101I	**0,073724**	**0,089852**	**0,076444**	**0,114798**	**0,104096**	**0,172367**	**0,080592**
102G	0,011629	0,030173	0,005001	0,005231	0,015538	0,010491	0,009092
103L	0,000677	0,001221	0,004502	0,010123	0,006803	0,007488	0,010724
105V	0,001992	0,00939	0,001842	0,011563	0,016301	0,014736	0,009741

106T	0,005578	0,00823	0,010958	0,022379	0,025389	0,008923	0,016571
107R	0,016527	0,02548	0,016464	0,027142	0,023902	0,035236	0,018586
108A	0,011259	0,021044	0,012007	0,028216	0,026514	0,040132	0,010151
109V	0,013104	0,036509	0,009012	0,017393	0,015597	0,057382	0,00616
110S	0,023445	0,040402	0,01299	0,033479	0,00328	0,023449	0,009872
122D	0,021098	0,033279	0,031029	0,037776	0,041901	0,040634	0,026319
123V/196C	0,006988	0,027599	0,004948	0,00597	0,021467	0,033551	0,011719
124L	**0,043437**	0,031458	0,036273	0,048948	0,054082	0,047004	**0,054218**
125R	**0,045216**	0,131377	**0,073883**	**0,087608**	**0,059982**	**0,146099**	0,034068
126A	0,005397	0,011653	0,009799	0,004918	0,00561	0,010333	0,002918
127A	0,009934	0,018069	0,011824	0,009125	0,013193	0,015917	0,007756
128G	0,012067	0,023268	0,01872	0,018619	0,02129	0,026264	0,013381
129V	0,002813	0,003355	0,004624	0,004414	0,006808	0,007984	0,003019
130A	0,006266	0,003488	0,009118	0,015732	0,016217	0,021156	0,011241
131T	0,023211	0,016183	0,027814	0,034977	0,044598	0,029667	0,029748
132Y	0,015242	0,030132	0,03197	0,027675	0,033401	0,029275	0,016146
133A	0,009508	0,026967	0,011482	0,003444	0,011997	0,014749	0,004648
134S	0,013756	0,042401	0,040134	0,052254	0,018758	0,061067	0,023245
136S	0,005524	0,016022	0,013895	0,0122	0,013366	0,018207	0,008389

137T	0,010733	0,030904	0,017196	0,028569	0,014777	0,043972	0,009273
138R	0,012897	0,033901	0,019172	0,031839	0,012693	0,044067	0,010364
139R	0,005013	0,016464	0,012019	0,01966	0,008979	0,047187	0,006353
141V	0,007545	0,010967	0,009133	0,023059	0,023037	0,019615	0,010062
142E	0,003003	0,008869	0,012045	0,01364	0,005067	0,0069	0,009273
143V	**0,035942**	0,023007	0,016194	0,017298	0,023902	0,010254	0,016514
144E	0,007828	0,005328	0,025639	0,024895	0,027804	0,029715	0,007246
145G	0,000731	0,005083	0,002781	0,009353	0,009058	0,00851	0,002616
146N	0,018636	0,040054	0,009649	0,011241	0,012234	0,048382	0,00651
147E/156L	0,003725	0,014573	0,005208	0,004029	0,006184	0,001891	0,00578
148I	0,017989	0,032568	0,018741	0,01617	0,029329	0,023958	0,009019
150T	**0,052157**	0,063459	**0,060975**	**0,108881**	**0,108274**	**0,126474**	**0,060623**
151H	0,035255	0,019151	0,035542	**0,071313**	**0,121312**	0,081973	**0,048962**
152S	0,030727	0,032011	0,038888	0,058471	**0,070571**	0,05545	0,027879
153L	0,015754	0,020317	0,010701	0,026421	0,020282	0,035753	0,015483
154E	0,004578	0,013542	0,009062	0,007965	0,005742	0,017177	0,002389
155G	0,000922	0,012326	0,005035	0,014593	0,012962	0,022371	0,007444
157S	0,004205	0,008144	0,003613	0,006045	0,004834	0,016248	0,005777
158S	0,009075	0,01177	0,011167	0,026771	0,042583	0,035855	0,015776

159S	0,004215	0,007161	0,003502	0,004394	0,001604	0,008872	0,002912
160G	0,008776	0,035247	0,023517	0,03727	0,021728	0,043087	0,010177
161D	0,006113	0,015657	0,010415	0,00841	0,004981	0,011467	0,001717
162A	0,004867	0,001093	0,00654	0,002468	0,014133	0,005124	0,001485
163V	0,010355	0,010747	0,012478	0,014111	0,02566	0,02182	0,010844
164R	0,003485	0,011368	0,006147	0,005578	0,013529	0,0175	0,0049
165F	0,002754	0,001719	0,006386	0,003553	0,01434	0,008867	0,005033
166G	0,011936	0,015149	0,008143	0,016367	0,013254	0,034167	0,017493
168V	0,002843	0,003087	0,004695	0,008137	0,015031	0,002314	0,010658
169E	0,006604	0,011328	0,008054	0,015621	0,011385	0,007512	0,013359
170L	0,001548	0,004569	0,002756	0,001755	0,002271	0,008068	0,01075
171F	0,004674	0,009277	0,008357	0,023078	0,025775	0,013476	0,009526
172Y	0,002665	0,009148	0,004144	0,010722	0,003943	0,020373	0,006132
175A	**0,03716**	0,07463	**0,052224**	0,057451	0,051889	0,066486	0,028472
176A	**0,06898**	**0,128966**	**0,07953**	**0,110744**	0,051816	**0,133607**	**0,050529**
177H		**0,167176**				**0,17603**	
178S	0,028787	**0,093884**	**0,057523**	0,059381	0,022172	**0,094605**	0,030062
179T	0,025985	**0,10105**	0,021811	0,010417	**0,068121**	**0,10226**	0,026689
180D	0,031028	0,01753	0,019014	0,024004	0,042528	0,022002	**0,036214**

181N	0,009347	0,025518	0,009412	0,002301	0,008382	0,013273	0,002941
182L	0,020189	0,049514	0,016435	0,031325	0,015791	0,066494	0,009215
183V	0,018225	0,036744	0,018541	0,063469	0,021358	0,053241	0,022119
184V	0,00824	0,018773	0,011467	0,028361	0,016666	0,01995	0,020775
185Y	0,006491	0,011886	0,002188	0,005872	0,002465	0,02112	0,004456
186V	0,002793	0,022721	0,012534	0,019947	0,002527	0,020527	0,001686
188S	0,029211	0,034287	0,041425	0,051265	0,054976	**0,116339**	0,021668
189A	0,00438	0,010534	0,005585	0,008069	0,009891	0,012435	0,008529
190S	0,004258	0,009071	0,00094	0,010617	0,016442	0,015942	0,002696
191V	0,001646	0,001453	0,005829	0,01118	0,006229	0,012255	0,013722
192L	0,001934	0,021854	0,005489	0,00251	0,005612	0,024777	0,002523
193Y	0,018315	0,043128	0,015126	0,048383	0,017316	0,067339	0,023095
194G	0,026419	0,047473	0,02689	0,049271	0,022444	0,064142	**0,039684**
197A	0,007939	0,06897	0,029523	0,031642	0,031867	0,06288	0,013918
198I	0,028533	0,071123	0,032267	**0,07626**	0,015589	**0,109746**	**0,036026**
199Y	**0,044513**	0,078923	**0,069905**	**0,094554**		**0,098069**	**0,045896**
202S	0,016552	0,020928	0,013982	0,041349		0,076887	0,015485
203R	**0,058631**	**0,114009**	**0,090294**	**0,116826**	0,057754	**0,179825**	**0,085399**
205S		**0,12945**				**0,178816**	

206A		**0,40036**					
209V	**0,037114**	0,067559	**0,052485**	0,050412	0,036294	0,039819	0,032138
210A	**0,046718**	**0,096764**	**0,076376**	0,060636	0,047348	0,089644	**0,042486**
213D	0,001997	0,016334	0,010378	0,004539	0,006695	0,007987	0,009214
214L	**0,08359**	**0,170652**	**0,113551**	0,05304	**0,062269**		**0,054132**
215A	0,011081	0,023254	0,009705	0,020806	0,021413	0,027703	0,007856
216E	0,028231	0,02018	0,020381	0,036218	0,045217	0,023449	0,016579
219T	0,023717	0,03154	0,024229	0,039932	0,03612	0,040052	0,014951
220S	0,013855	0,023436	0,015531	0,026756	0,029226	0,032328	0,007205
221I	0,009658	0,026842		0,007007	0,006671	0,008296	0,006199
222E	0,000778	0,020978	0,008324	0,018551	0,024613	0,034588	0,004107
223R	0,01645	0,042802	0,022123	0,038277	0,035327	0,053092	0,005557
224I	0,002984	0,005874	0,004294	0,009354	0,009	0,002304	0,005659
225Q	0,017633	0,02746	0,019109	0,023623	0,006661	0,040604	0,012229
226Q	0,004036	0,015601	0,006304	0,008349	0,005702	0,024723	0,008745
227H	0,030884	0,02842	0,027951	0,037024	0,044194	0,03967	0,031518
228Y	0,013362	0,015903	0,015557	0,021549	0,013007	0,017612	0,005383
230E	0,015911	0,009047	0,017016	0,032463	0,055203	0,035039	0,020203
231A	0,004235	0,007342	0,009201	0,018803	0,022469	0,015874	0,00656

232Q	0,002688	0,020405	0,021285	0,026119	0,028431	0,045891	0,019988
233F	0,007843	0,014342	0,037	0,052603	0,029187	0,01538	0,030598
234V	0,007808	0,011852	0,033663	0,042419	0,033094	0,01607	0,023347
235I	0,012633	0,033069	0,01528	0,052707	0,009962	0,067972	0,015123
237G	**0,040999**	**0,102416**	0,045399	0,06643	0,029587	**0,174503**	0,0105
238H		**0,239213**		**0,169212**		**0,244523**	
239G						**0,229935**	
242G	0,005509	0,028218	0,021967	0,033923	0,010422	0,030419	0,012083
244L	0,006505	0,032666	0,011945	0,019858	0,00963	0,046146	0,005641
245D	0,02536	0,022431	0,022718	0,037661	0,029649	0,043312	0,024623
246I	0,014947	0,024015	0,016772	0,031655	**0,058786**	0,026061	0,002671
247L	0,007598	0,039377	0,020254	0,032013	0,026367	0,048405	0,004716
248K	0,01434	0,042156	0,019462	0,040919	0,023411	0,08184	0,026817
249H	0,006226	0,016536	0,011188	0,010224	0,010226	0,009815	0,004831
250T	**0,038213**	0,062577	**0,052246**	**0,069534**	**0,06674**	0,079488	**0,041326**
252N	**0,039376**	0,050465	0,048748	**0,081378**	**0,086444**	0,079245	0,033173
254V	0,025472	0,042417	0,020048	0,042275	**0,096544**	0,01512	0,021309
255K	**0,058836**	0,069111	**0,064704**	**0,08856**	**0,115756**	0,083204	**0,042943**
256A	**0,059839**	0,090646	**0,072985**	**0,105599**	**0,099834**	**0,126824**	**0,041908**

257H	**0,044528**	0,022529	0,04823	0,06183	**0,100049**	0,065491	**0,036703**
258T	**0,050975**	0,084446	**0,072017**	**0,119376**	**0,137014**	0,076343	**0,036448**
259N	0,017116	0,027377	0,027716	0,01918	0,021037	0,035188	0,005902
260R	**0,072629**	**0,104093**	**0,092009**	**0,128579**	**0,132502**	**0,125824**	**0,054315**
261S	0,033188	0,051217	0,038979	0,048606	0,047937	0,042688	0,014222
262V	0,015436	0,018649	0,013393	0,024955	0,025504	0,029532	0,012623
263V	0,014485	0,021663	0,013062	0,026388	0,026794	0,030837	0,010219
264E	0,013323	0,004688	0,003171	0,012729	0,018111	0,016396	0,007985

7. Chemical shift perturbation histograms

Chemical shift perturbation was calculated according equation (1) [2]

$$\text{CSP} = \Delta\delta_{(1\text{H},15\text{N})} = \sqrt{(\Delta\delta_{1H}{}^{2} + ({}^{1}/_{R_{scale}} \times \Delta\delta_{15N}{}^{2})} \qquad R_{scale} = 6.5 \quad (1)$$

Where $\Delta\delta_{1H}$ is the chemical shift change for proton dimension, $\Delta\delta_{15N}$ is the chemical shift change for nitrogen dimension, R_{scale} is a scaling factor.

The chemical shift perturbations (CSP) were classified as significant (SSP) when the observed $\Delta\delta_{(1H,15N)}$ was greater than the population mean plus the standard deviation (μ+1σ) – solid line; (μ+2σ) – dashed line, and (μ+3σ) – dashed line.

Further on, K_d was calculated according: [3]

$$\Delta\delta_{max}\Delta\delta_{obs} = \Delta\delta_{max}\frac{([P]+[L]+K_d)-\sqrt{([P]+[L]+K_d)^2-4[P][L]}}{2[P]} \quad (2)$$

Where Kd is the dissociation constant, [P] and [L] are protein and ligand concentrations, respectively. $\Delta\delta_{max}$ were estimated in MestReNova by extrapolation of the observed chemical shift differences $\Delta\delta_{obs}$.

The histograms for the titrated compounds (indicated in the y axis title) with NDM-1

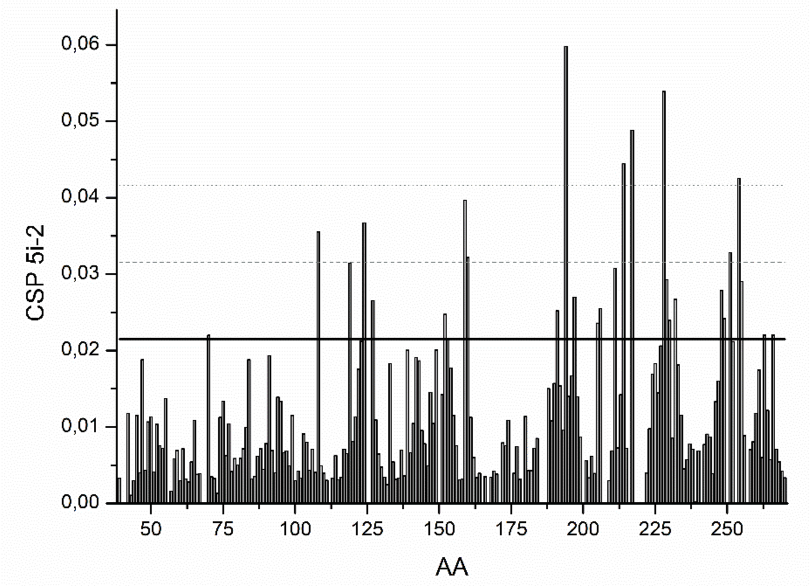

Figure S165. The chemical shift perturbations observed for the backbone amides of ^{15}N-labelled NDM-1 upon addition of **5i-2**.

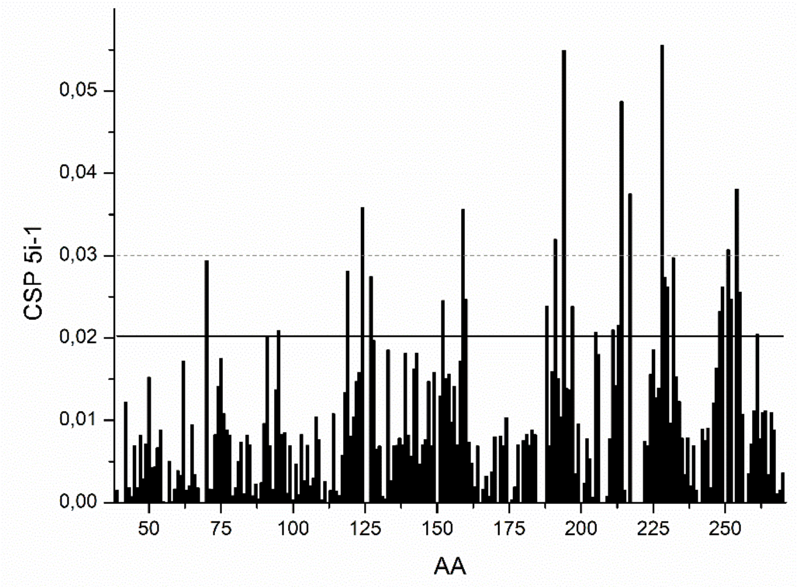

Figure S166. The chemical shift perturbations observed for the backbone amides of ^{15}N-labelled NDM-1 upon addition of **5i-1**.

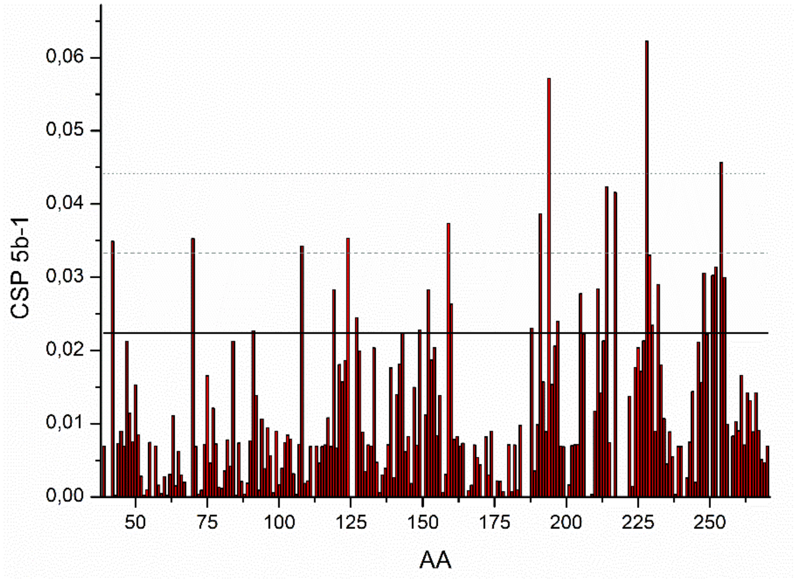

Figure S167. The chemical shift perturbations observed for the backbone amides of ^{15}N-labelled NDM-1 upon addition of **5b-1**.

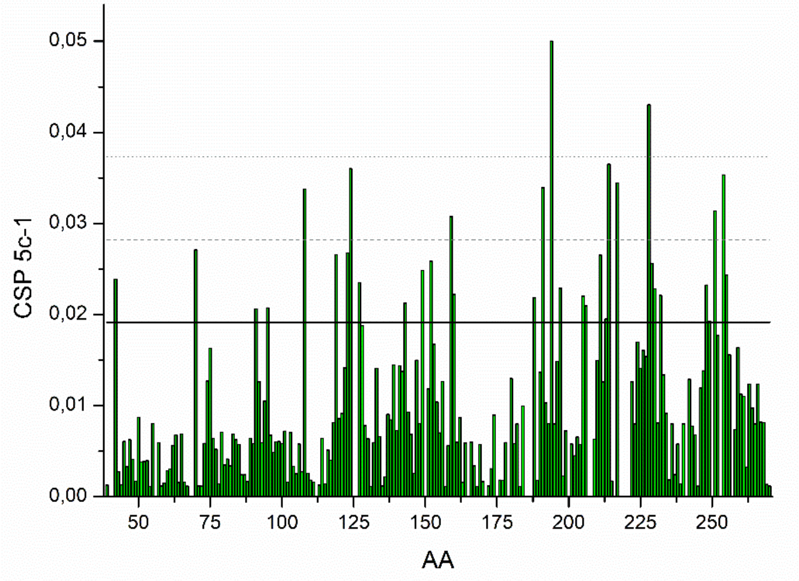

Figure S168. The chemical shift perturbations observed for the backbone amides of ^{15}N-labelled NDM-1 upon addition of **5c-1**.

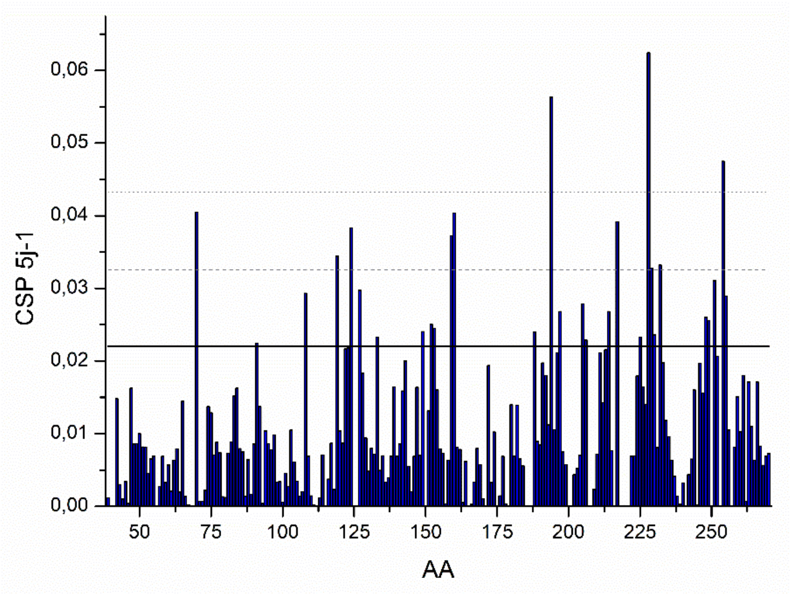

Figure S169. The chemical shift perturbations observed for the backbone amides of ^{15}N-labelled NDM-1 upon addition of **5j-1**.

Below are presented all histograms for compounds titrated (indicated in the y axis title) with VIM-2

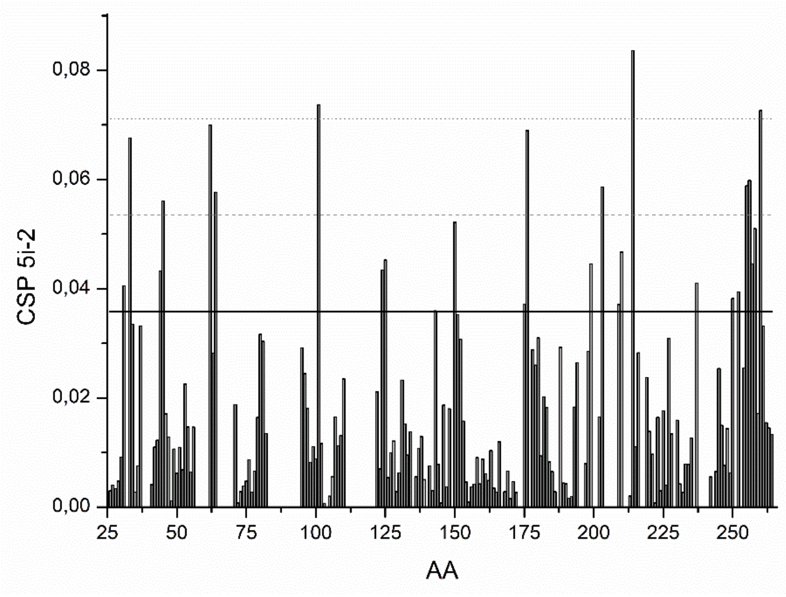

Figure S170. The chemical shift perturbations observed for the backbone amides of ^{15}N-labelled VIM-2 upon addition of **5i-2**.

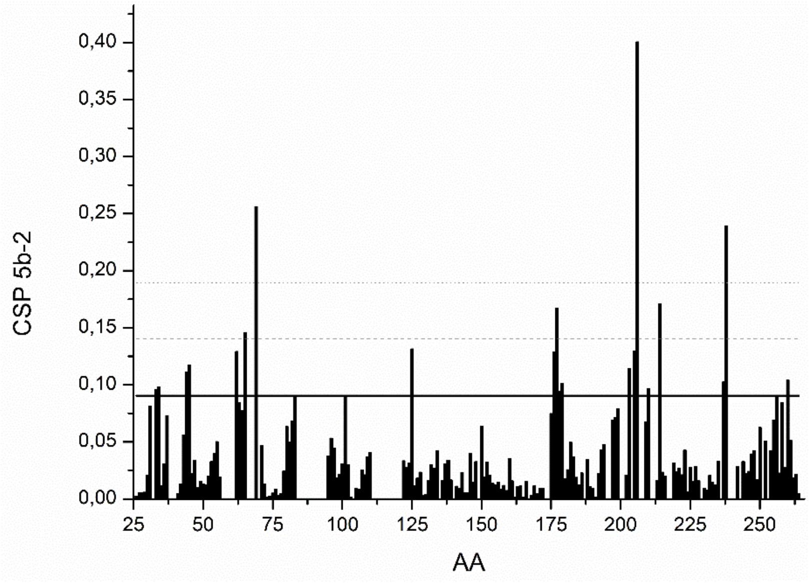

Figure S171. The chemical shift perturbations observed for the backbone amides of ^{15}N-labelled VIM-2 upon addition of **5b-2**.

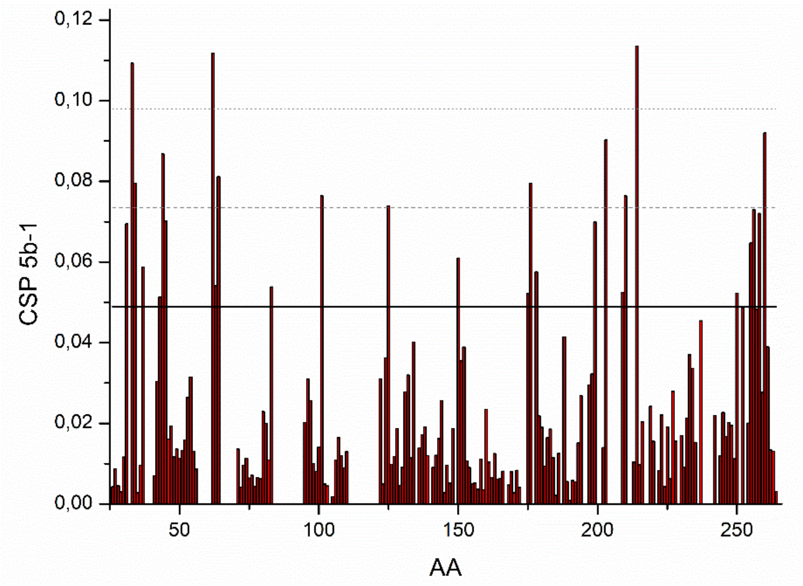

Figure S172. The chemical shift perturbations observed for the backbone amides of ^{15}N-labelled VIM-2 upon addition of **5b-1**.

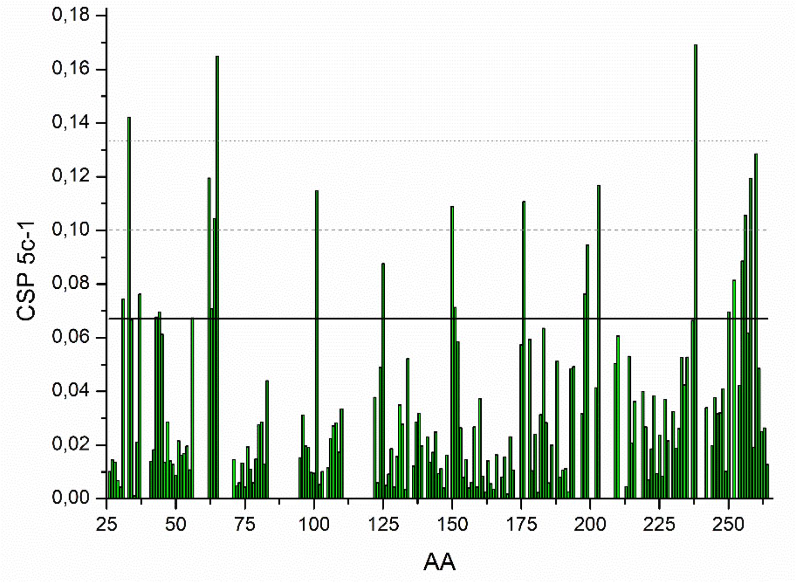

Figure S173. The chemical shift perturbations observed for the backbone amides of ^{15}N-labelled VIM-2 upon addition of **5c-1**.

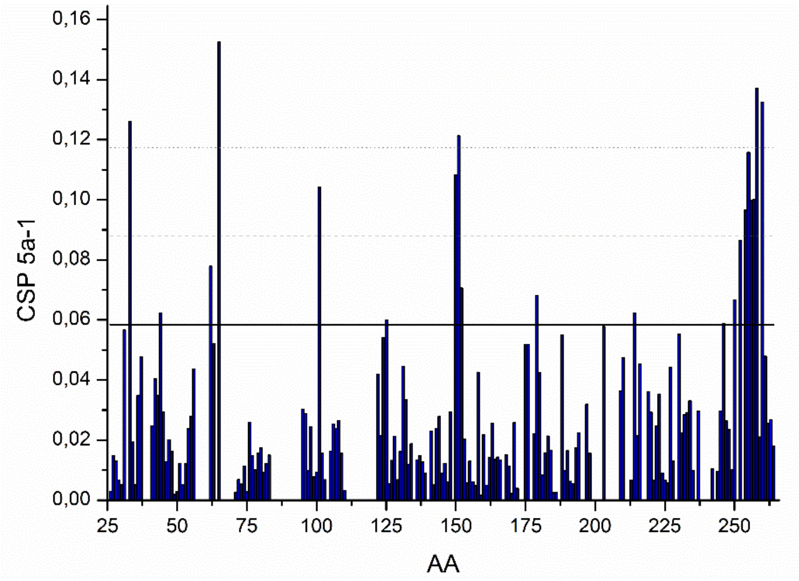

Figure S174. The chemical shift perturbations observed for the backbone amides of ^{15}N-labelled VIM-2 upon addition of **5a-1**.

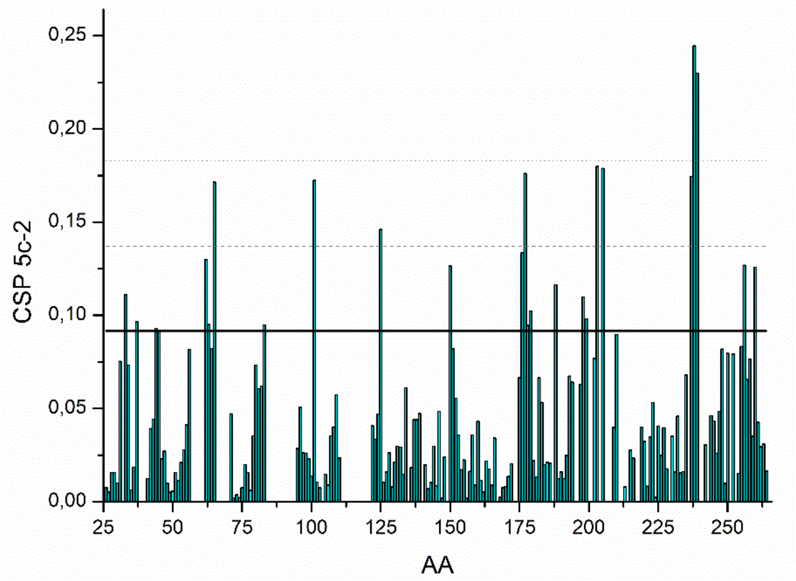

Figure S175. The chemical shift perturbations observed for the backbone amides of ^{15}N-labelled VIM-2 upon addition of **5c-2**.

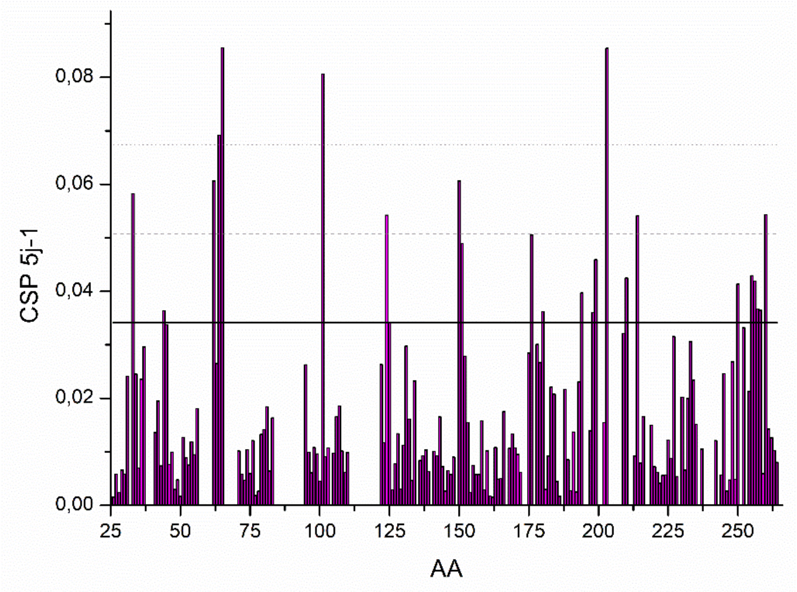

Figure S176. The chemical shift perturbations observed for the backbone amides of ^{15}N-labelled VIM-2 upon addition of **5j-1**.

8. Estimation of binding constants (K_d)

Table S5. Estinmated binding constants

AA NDM-1	5i-2	5i-1	5b-1	5c-1	5j-1
070F	4.90		2.92	15.21	2.54
091T			5.76	4.17	2.11
108E	2.03		6.63	4.50	3.54
119T	26.36	0.93	18.13	10.67	7.97
123Q				1.69	
124D	14.14	3.53	3.17	5.76	12.39
127G	1.76	8.94	8.34	10.76	12.39
133H_225D					1.42
143A				8.49	
149A			1.71	1.76	3.18
152E	1.18		3.52	3.85	32.94
159H	8.30	2.47	21.48	11.67	8.39
160S	17.62	0.25	8.97	5.40	15.69
188G		0.49	3.40	15.77	6.63
191S		0.12	0.85	0.48	

194I	2.49	4.99	2.93	6.26	6.10
197G	15.23	1.27	10.41	1.51	3.05
205F	6.84	2.30	10.98	3.19	8.41
206G	6.81			3.95	7.72
211K	17.1	0.14	3.49	10.05	
213S		0.39		9.53	
214K	0.78	0.57	1.357	6.40	1.96
217S	16.44	3.06	10.45	11.85	8.15
228H	10.14	3.08	11.04	17.90	4.94
229Y	2.30	0.34	2.241	6.74	1.31
230A	2.71	0.18	4.33	11.50	8.81
232S	5.57	0.93	12.07	10.25	21.28
248M	2.58	0.78	12.90	36.59	8.64
249S	8.45		1.71	4.15	34.01
251S	3.38	1.82	6.07	15.43	12.27
252A		0.48	3.48		
254D	5.01	7.53	9.74	5.56	9.29
255S	1.66	9.91	7.37	10.55	18.64
263A_266A	0.69				

9. Linear correlation of chemical shift perturbation

Table S6. Parameters from linear regration for titrated compounds with NDM-1.

Equation	y = a + b*x			
Plot	5i-1	5b-1	5c-1	5j-1
Weight	No Weighting			
Intercept	2,52095E-4 ± 5,09129E-4	3,49794E-4 ± 4,91977E-4	9,96625E-4 ± 4,358E-4	4,78525E-4 ± 5,20324E-4
Slope	0,91064 ± 0,0325	0,97999 ± 0,03148	0,80359 ± 0,02789	0,94623 ± 0,0333
Residual Sum of Squares	0,00406	0,00384	0,00301	0,0043
Pearson's r	0,89774	0,91436	0,90209	0,89974
R-Square (COD)	0,80593	0,83605	0,81377	0,80953
Adj. R-Square	0,8049	0,83519	0,81279	0,80853

The linear regression of the chemical shift perturbations of ligands **5i-1**, **5b-1**, **5c-1** and **5j-1** as a function of the chemical shift perturbations of the amino acids of **5i-2**.

The linear correaltions of the chemical shift perturbations observed for a specific ligand in comparison with those observed for 5i-2 (x-axis) for NDM-1 titrations

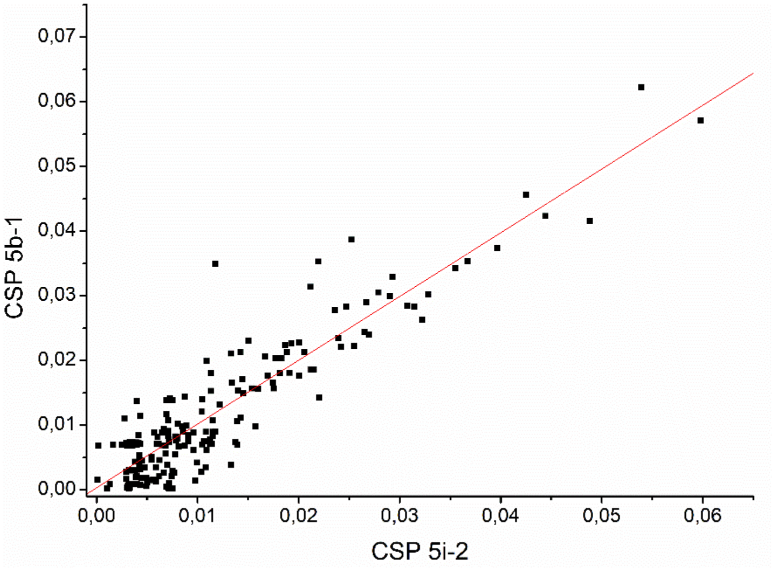

Figure S177. The linear regression of the chemical shift perturbations of ligands **5b-1** as a function of the chemical shift perturbations of the amino acids of **5i-2**.

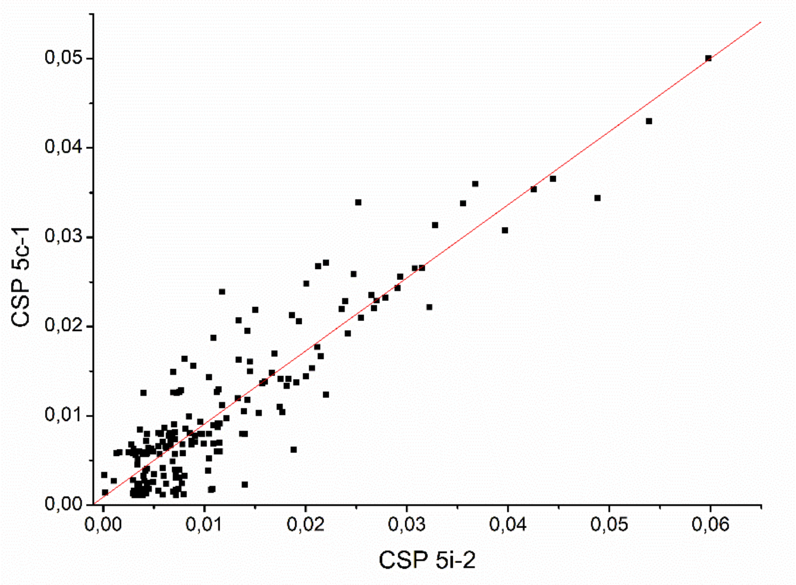

Figure S178. The linear regression of the chemical shift perturbations of ligands **5c-1** as a function of the chemical shift perturbations of the amino acids of **5i-2**.

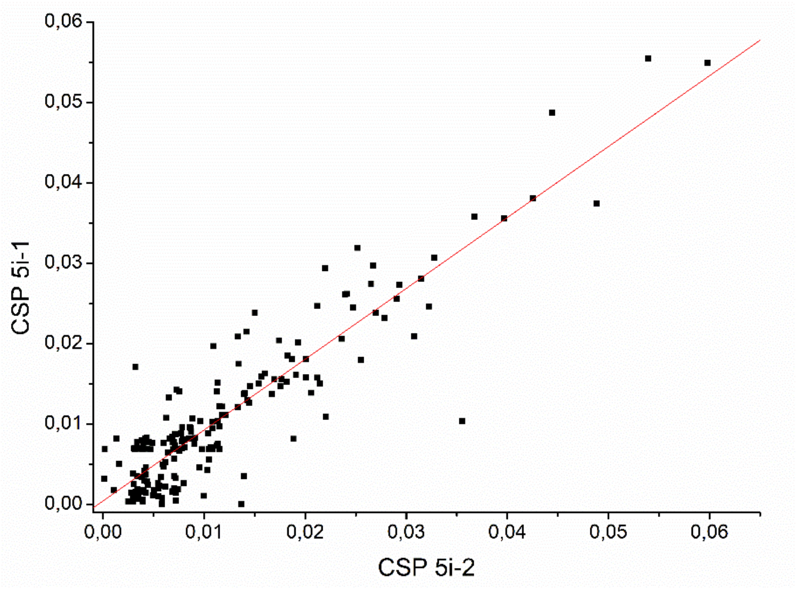

Figure S179. The linear regression of the chemical shift perturbations of ligands **5i-1** as a function of the chemical shift perturbations of the amino acids of **5i-2**.

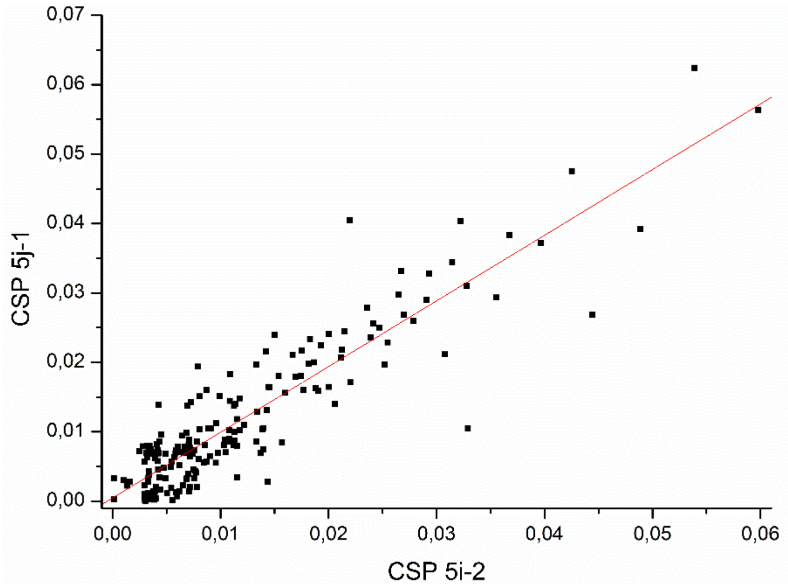

Figure S180. The linear regression of the chemical shift perturbations of ligands **5j-1** as a function of the chemical shift perturbations of the amino acids of **5i-2**.

Table S7. Parameters from linear regration for titrated compounds with VIM-2.

Equation	y = a + b*x					
Plot	5b-2	5b-1	5c-1	5a-1	5c-2	5j-1
Weight	No Weighting					
Intercept	0,00622 ± 0,00189	0,00153 ± 0,00115	0,00579 ± 0,0017	0,00619 ± 0,00196	0,00925 ± 0,00237	0,00289 ± 8,81227E-4
Slope	1,53935 ± 0,0744	1,25313 ± 0,04543	1,46721 ± 0,06694	1,24922 ± 0,07875	1,79865 ± 0,09657	0,78234 ± 0,03477
Residual Sum of Squares	0,04847	0,01794	0,03924	0,05102	0,07453	0,01059
Pearson's r	0,84817	0,90605	0,86142	0,77811	0,8224	0,86716
R-Square (COD)	0,71939	0,82093	0,74205	0,60545	0,67634	0,75197
Adj. R-Square	0,71771	0,81985	0,7405	0,60304	0,67439	0,75048

The linear regression of the chemical shift perturbations pf ligands **5i-1**, **5b-1**, **5c-1** and **5j-1** as a function of the chemical shift perturbations of the amino acids of **5i-2**.

The linear correaltions of the chemical shift perturbations observed for a specific ligand in comparison with those observed for 5i-2 (x-axis) for VIM-2 titrations.

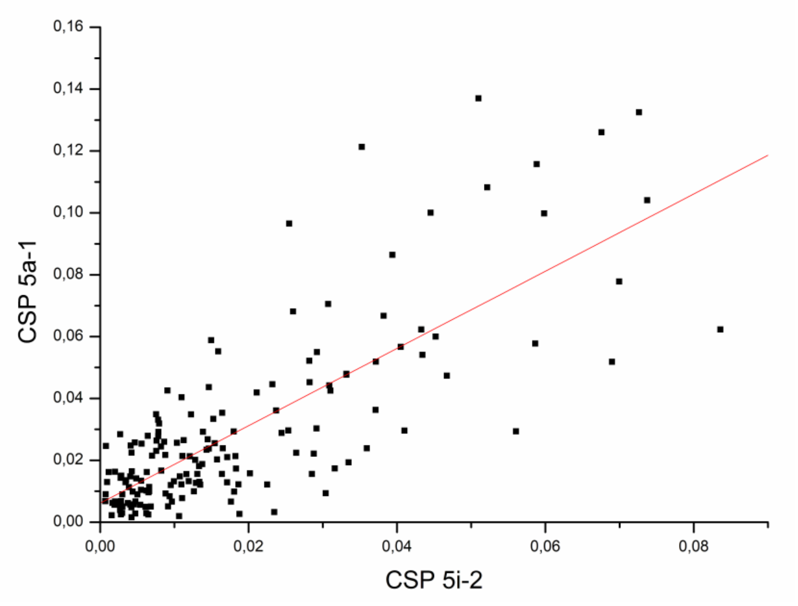

Figure S181. The linear regression of the chemical shift perturbations of ligands **5a-1** as a function of the chemical shift perturbations of the amino acids of **5i-2**.

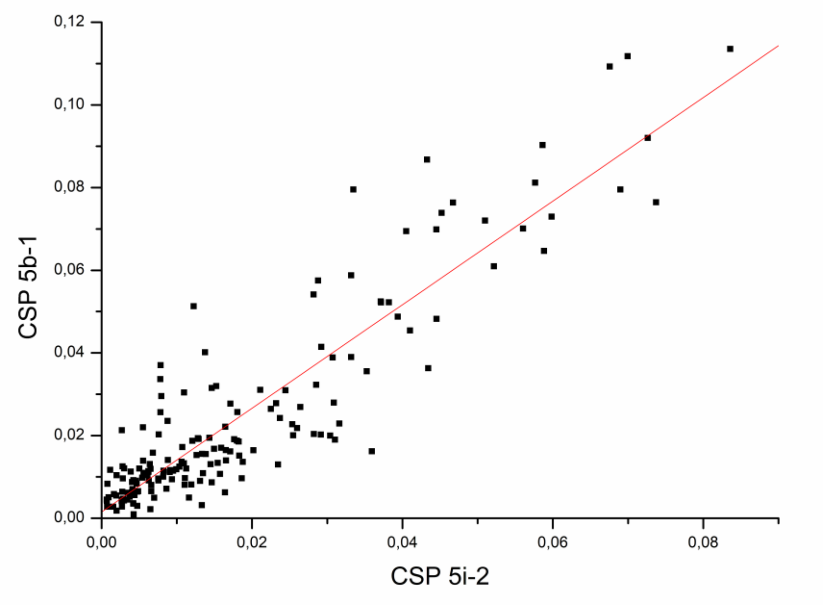

Figure S182. The linear regression of the chemical shift perturbations of ligands **5b-1** as a function of the chemical shift perturbations of the amino acids of **5i-2**.

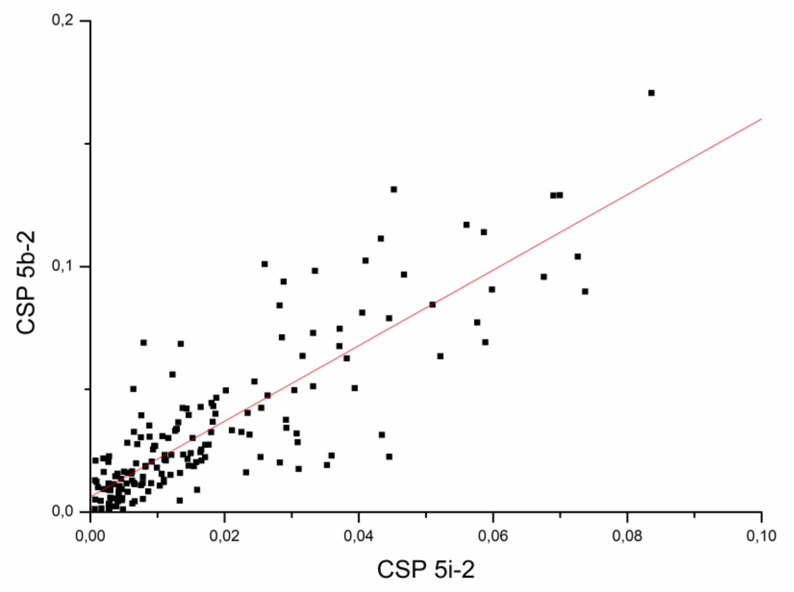

Figure S183. The linear regression of the chemical shift perturbations of ligands **5b-2** as a function of the chemical shift perturbations of the amino acids of **5i-2**.

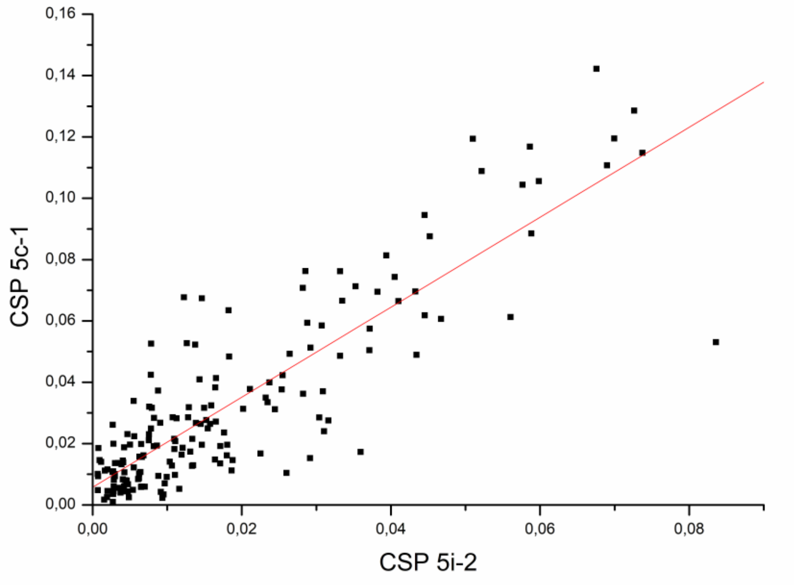

Figure S184. The linear regression of the chemical shift perturbations of ligands **5c-1** as a function of the chemical shift perturbations of the amino acids of **5i-2**.

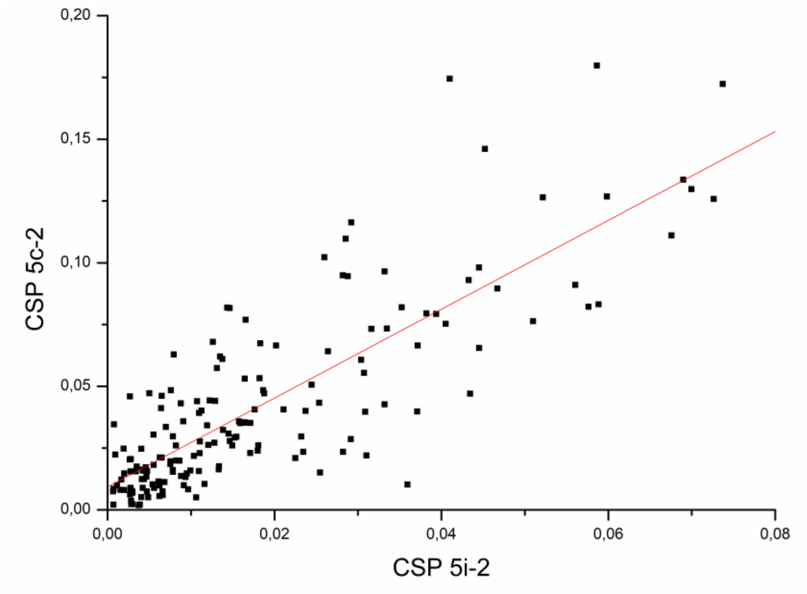

Figure S185. The linear regression of the chemical shift perturbations of ligands **5c-2** as a function of the chemical shift perturbations of the amino acids of **5i-2**.

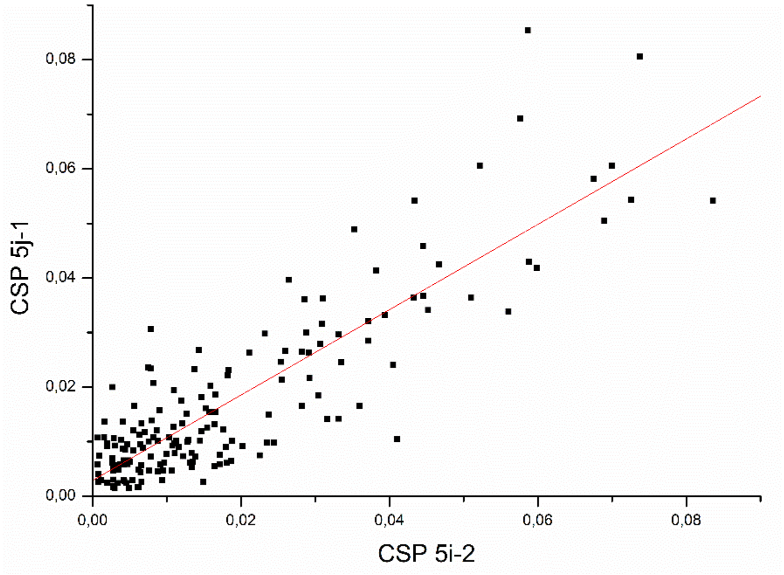

Figure S186. The linear regression of the chemical shift perturbations of ligands **5j-1** as a function of the chemical shift perturbations of the amino acids of **5i-2**.

10. Evaluation of the binding energies of the docking poses

Table S8. Docking poses of ligands to VIM-2[1]

Compound code	π-cation [kcal/mol]	π-π [kcal/mol]
5a-1-RS	**-42.8**	-36.6
5a-1-SR	n.b.[2]	-24.1
5b-1-SR	-32.2	-32.4
5b-1-RS	**-43.8**	-33.0
5b-2-SS	-30.3	-11.7
5b-2-RR	**-30.7**	-20.1
5c-1-RS	**-48.5**	-33.6
5c-1-SR	-33.5	-4.2
5c-2-RR	**-37.2**	-27.4
5c-2-SS	n.b.	-28.1
5i-2-SS*	-47.0	-43.0
5i-2-RR*	**-49.2**	-38.1
5j-1-RS*	**-46.2**	-38.8
5j-1-SR*	n.b.	-10.4

[1]Compounds marked with a * are more flexible due to a CH_2 being incorporated between the amide and the aromatic ring. Energies highlighted in green correspond to the binding modes with the lowest energy among the possible stsreoisomers. The docking poses correspond to the orientations that provide stabilization through a π-cation or a π-π interaction, as described in the main text. [2] n.b., the docking did not provide any feasible binding mode for this stereoisomer.

Table S9. Docking poses of ligands of RS/SR (left) and SS/RR (right) configurations. Energies are shown for the preferred binding mode that has a π-cation interaction to VIM-2[1]

	RS	*SR*
**5a-1**	**-42.9**	n.b.
**5b-1**	**-43.8**	-32.2
**5c-1**	**-48.5**	-33.5
**5j-1**	**-46.2**	n.b.

n.b.: the docking did not provide any feasible binding mode for this stereoisomer.

	RR	*SS*
**5b-2**	-30.7	-30.3
**5c-2**	-37.2	n.b.
**5i-2**	-49.2	-46.9

n.b.: the docking did not provide any feasible binding mode for this stereoisomer.

Dominating π-cation binding mode always has lower binding energy for the *RS* as compared to the *SR*, and for the *RR* as compared to the *SS* stereoisomer.

Table S10. Docking poses of ligands to NDM-1[1]

Compound code	π-cation [kcal/mol]	π-π [kcal/mol]
**5b-1-RS**	**-36.9**	n.b.
**5b-1-SR**	-32.2	-35.8
**5c-1-RS**	**-44.6**	n.b.
**5c-1-SR**	-32.2	-38.3
**5i-2-SS***	-13.9	-14.9
**5i-2-RR***	-29.3	**-43.3**
**5i-1-RS***	n.b.	n.b.
**5i-1-SR***	**-45.0**	-22.7
**5j-1-RS***	-30.0	n.b.
**5j-1-SR***	**-41.5**	-33.5

[1]Compounds marked with a * are more flexible due to a CH_2 being incorporated between the amide and the aromatic ring. Energies highlighted in green correspond to the binding modes with the lowest energy among the possible stereoisomers. The docking poses correspond to the orientations that provide stabilization through a π-cation or a π-π interaction, as described in the main text. [2] n.b., the docking did not provide any feasible binding mode for this stereoisomer.

For the more rigid type of ligand, lacking a CH_2 group in between the aromatic moiety and the amide, the *RS* configuration provides a better binding energy than *SR*, as a rule. This configuration shows a preference for the binding mode participating in π-cation interaction. The *RR* isomer provides better binding energy than the *SS* isomer, and it shows a π-π interaction.’

11. The root-mean-square deviation of atomic positions of the binding energies of the docking poses

Table S11. The root-mean-square deviation of atomic positions of the binding energies of the docking poses

Compound	pi-cation NDM-1 [kcal/mol]	pi-cation VIM-2 [kcal/mol]
**5a-1-RS**	-	0
**5b-1-RS**	2.65	3.78
**5b-2-RR**	-	3.83
**5c-1-RS**	0	3.21
**5c-2-RR**	-	3.58
**5i-RR**	2.68	3.43
**5i-SR**	2.39	3.59
**5j-1-SR**	2.59	3.70
**Mean**	2.58±0.12	3.57±0.21

12. SDS-Page Analysis for ^{15}N-labelled NDM-1 and VIM-2

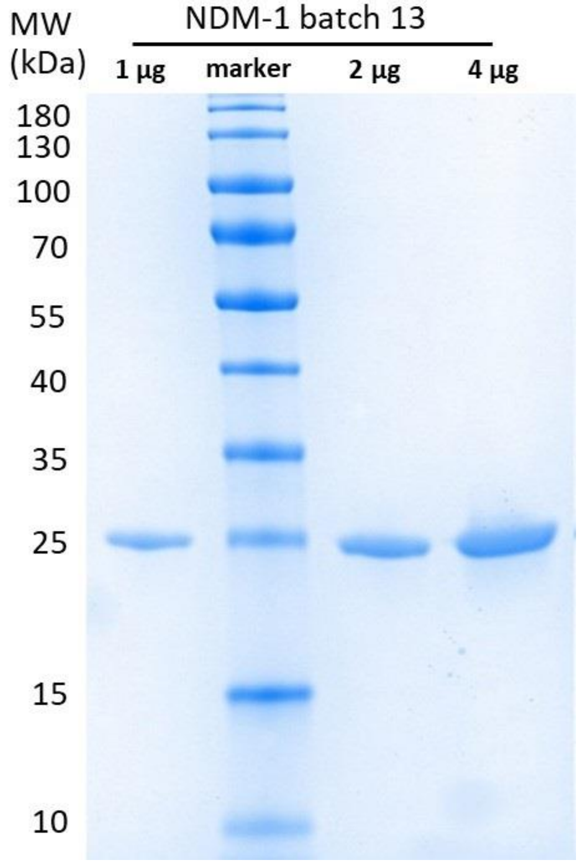

Figure S187. SDS-PAGE analysis of samples of the NDM-1 batch 13, final product.

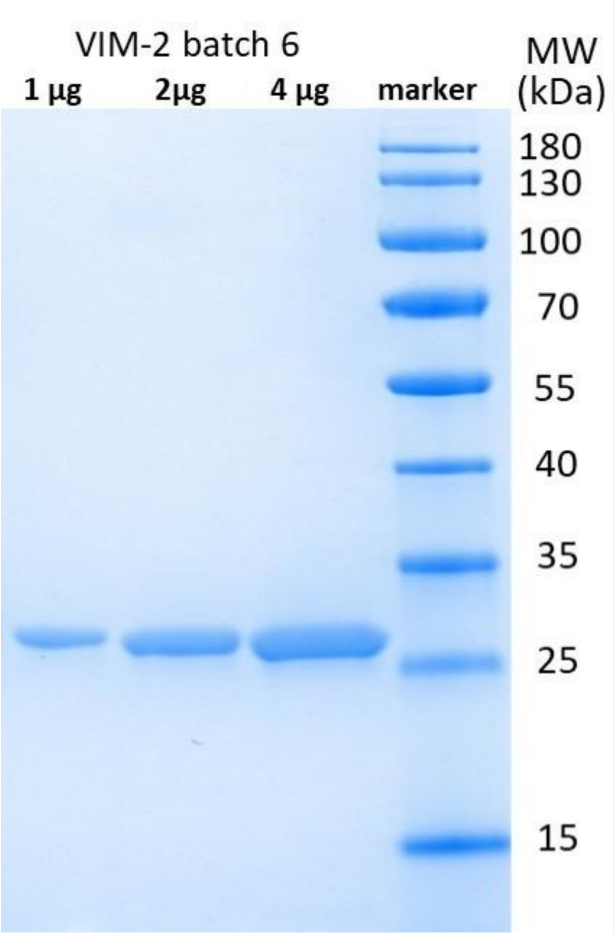

Figure S188. SDS-PAGE analysis of samples of the VIM-2 batch 6, final product.

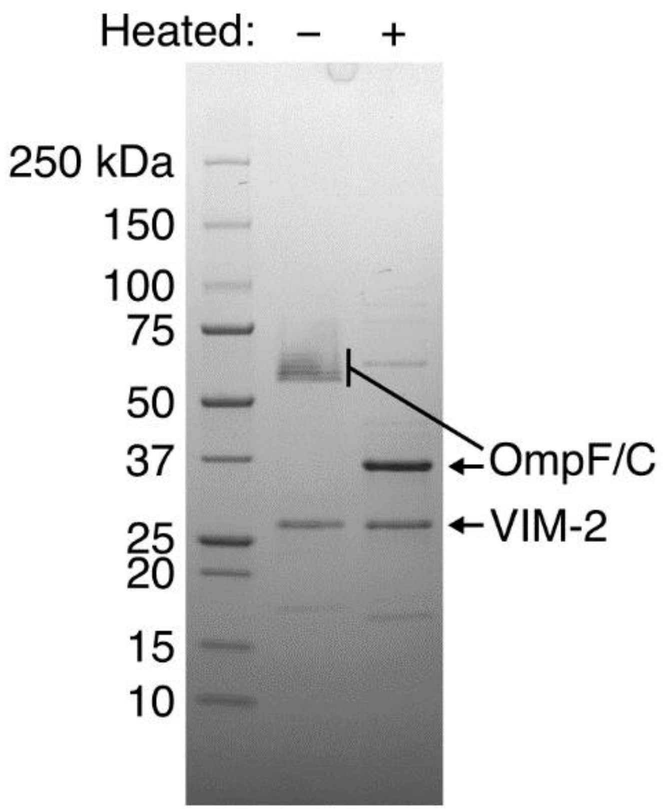

Figure S189. SDS-page analysis of OMVs carrying VIM-2 inside their lumen. Samples were left at room temperature (-) or heat-denatured at 98 °C (+) for 15 minutes prior to separation.

13. Long term stability

The stability of the α-aminophosphonate inhibitors was evaluated by LC-MS, following 3 months after the preparation of the stock solutions (prepared in the buffer 20 mM KPO_4, 0.1 mM $ZnCl_2$, pH 7.0 with 10% D_2O and 2.5% DMSO-d_6). The UV chromatograms and mass spectra of selected samples is shown.

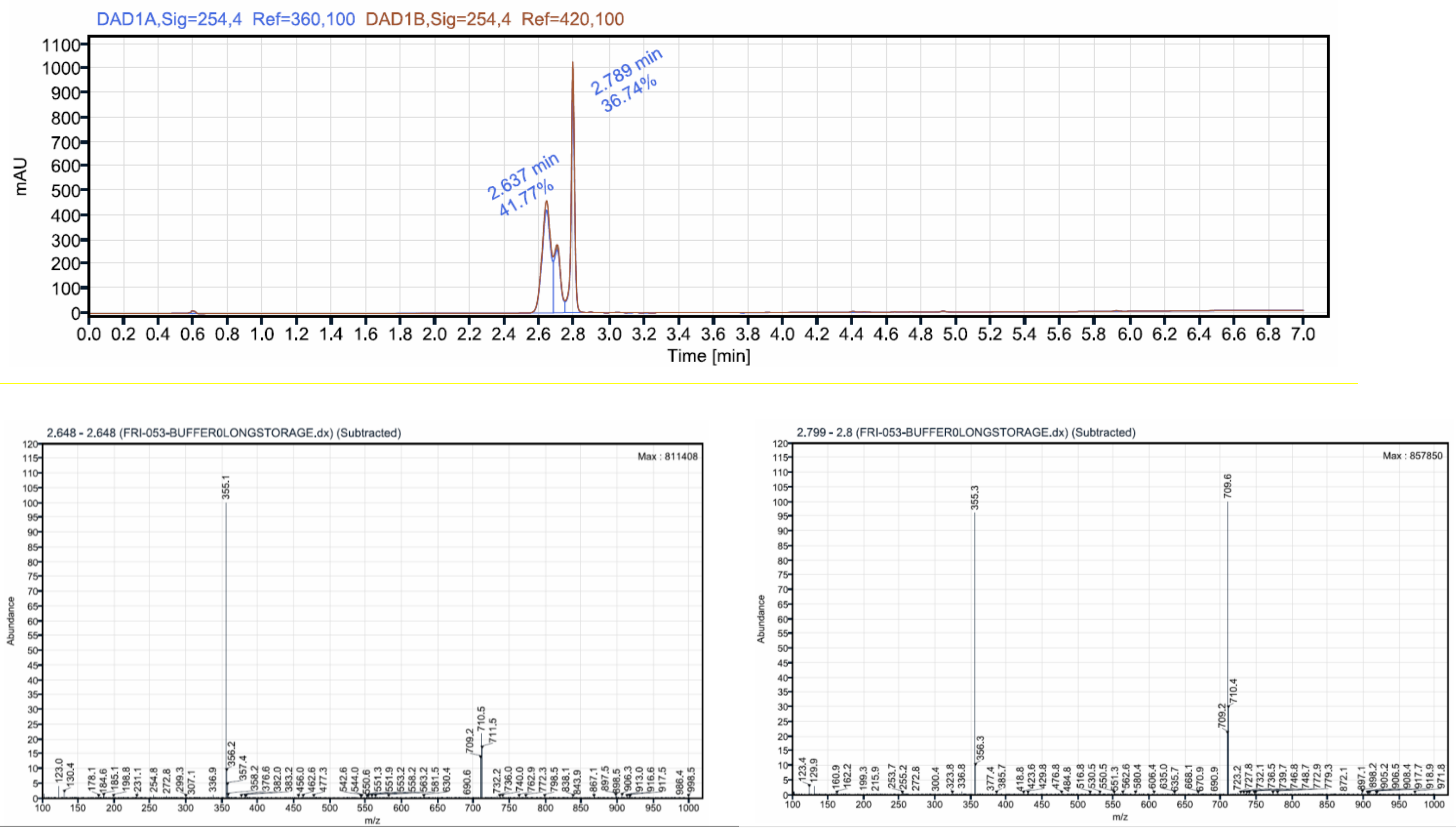

Figure S190. LC-MS results for **5g-1** after ~3 months.

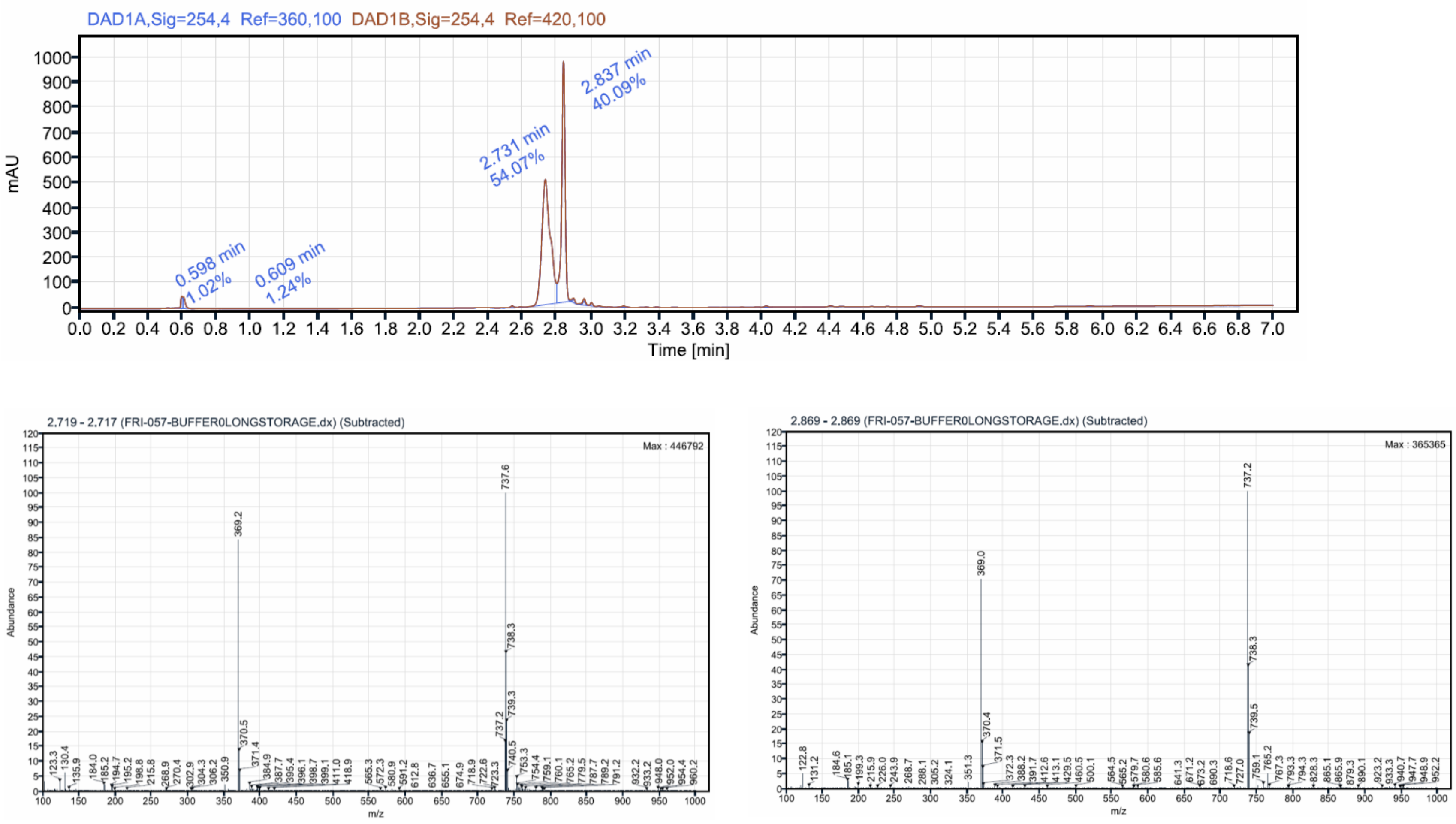

Figure S191. LC-MS results for **5l-2** after ~3 months.

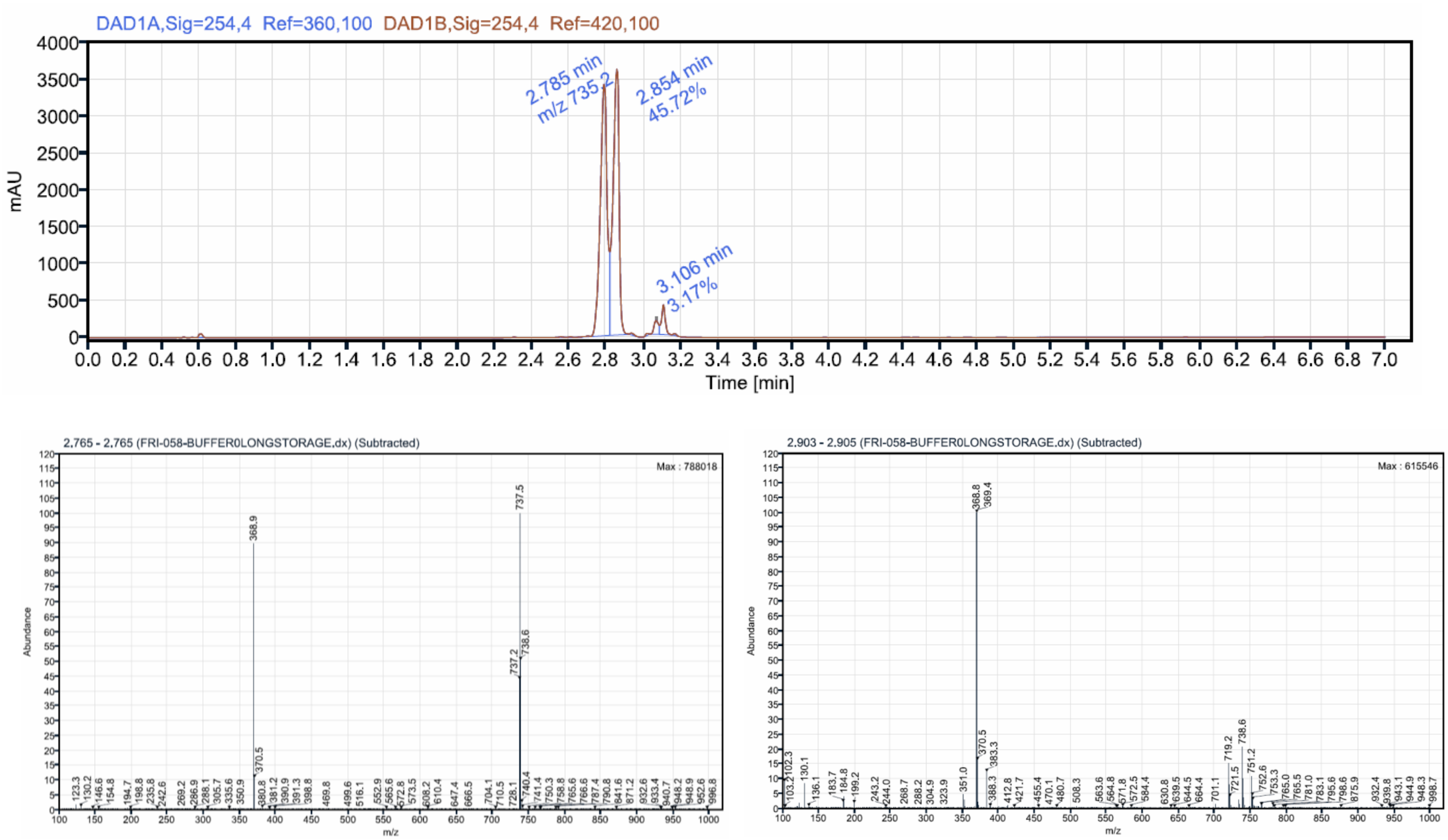

Figure S192. LC-MS results for **5l-1** after ~3 months.

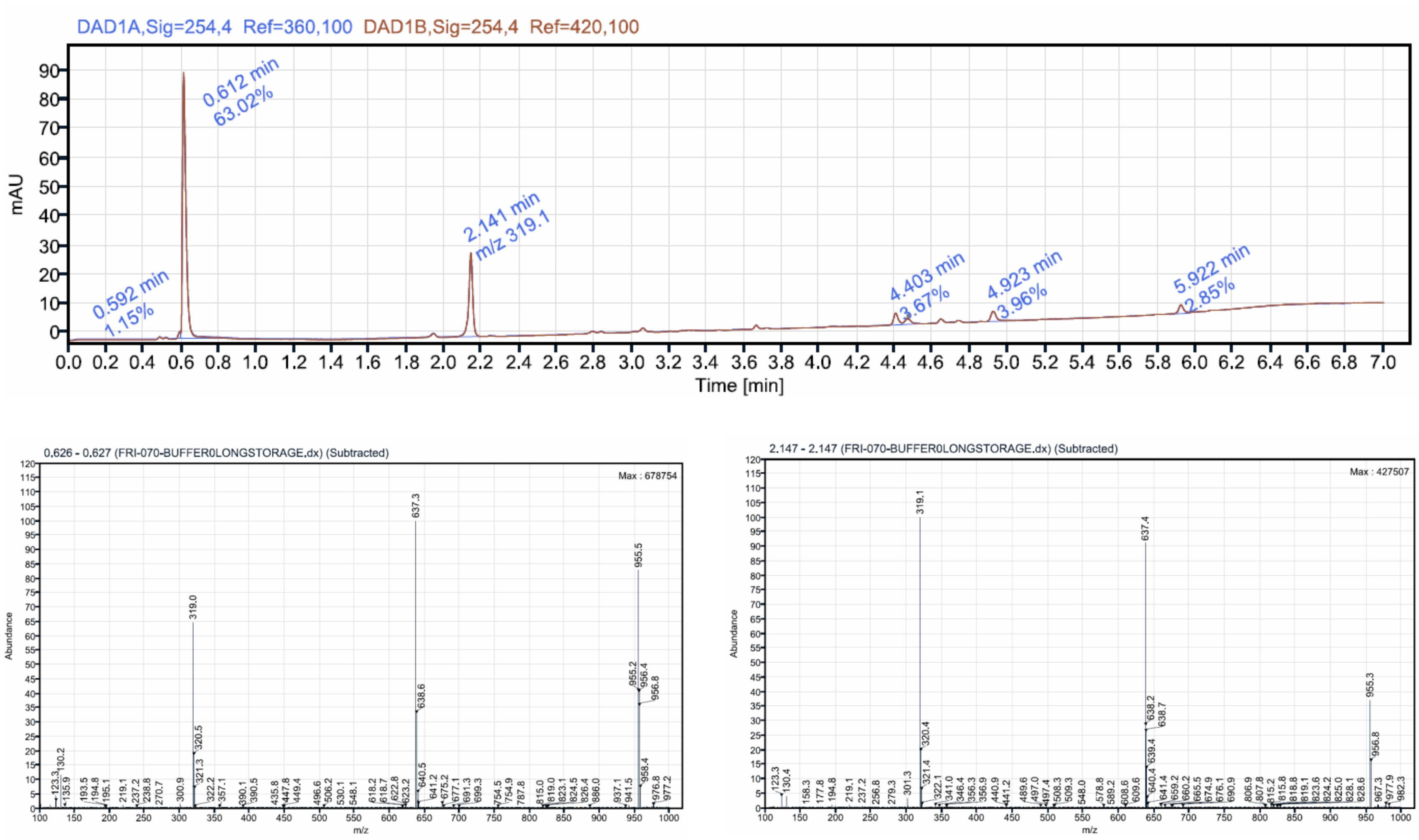

Figure S193. LC-MS results for **5j-1** after ~3 months.

14. References

1. Sievers, F.; Wilm, A.; Dineen, D.; Gibson, T. J.; Karplus, K.; Li, W.; Lopez, R.; McWilliam, H.; Remmert, M.; Söding, J.; Thompson, J. D.; Higgins, D. G., Fast, scalable generation of high-quality protein multiple sequence alignments using Clustal Omega. *Mol. Syst. Biol.* **2011,** *7* (1), 539.
2. Williamson, M. P., Using chemical shift perturbation to characterise ligand binding. *Progr. Nucl. Magn. Reson. Spectr*. **2013,** *73*, 1-16.
3. Fielding, L., NMR methods for the determination of protein-ligand dissociation constants. *Curr. Top Med. Chem.* **2003,** *3* (1), 39-53.
